# Screening for the primary prevention of fragility fractures among adults aged 40 years and older in primary care: systematic reviews of the effects and acceptability of screening and treatment, and the accuracy of risk prediction tools

**DOI:** 10.1186/s13643-023-02181-w

**Published:** 2023-03-21

**Authors:** Michelle Gates, Jennifer Pillay, Megan Nuspl, Aireen Wingert, Ben Vandermeer, Lisa Hartling

**Affiliations:** Department of Pediatrics, Alberta Research Centre for Health Evidence, University of Alberta, Edmonton Clinic Health Academy, 11405-87 Avenue NW, Edmonton, Alberta T6G 1C9 Canada

**Keywords:** Screening, Risk prediction, Calibration, Treatment, Acceptability, Fracture, Osteoporosis, Systematic review, Meta-analysis, Guideline

## Abstract

**Background:**

To inform recommendations by the Canadian Task Force on Preventive Health Care, we reviewed evidence on the benefits, harms, and acceptability of screening and treatment, and on the accuracy of risk prediction tools for the primary prevention of fragility fractures among adults aged 40 years and older in primary care.

**Methods:**

For screening effectiveness, accuracy of risk prediction tools, and treatment benefits, our search methods involved integrating studies published up to 2016 from an existing systematic review. Then, to locate more recent studies and any evidence relating to acceptability and treatment harms, we searched online databases (2016 to April 4, 2022 [screening] or to June 1, 2021 [predictive accuracy]; 1995 to June 1, 2021, for acceptability; 2016 to March 2, 2020, for treatment benefits; 2015 to June 24, 2020, for treatment harms), trial registries and gray literature, and hand-searched reviews, guidelines, and the included studies. Two reviewers selected studies, extracted results, and appraised risk of bias, with disagreements resolved by consensus or a third reviewer. The overview of reviews on treatment harms relied on one reviewer, with verification of data by another reviewer to correct errors and omissions. When appropriate, study results were pooled using random effects meta-analysis; otherwise, findings were described narratively. Evidence certainty was rated according to the GRADE approach.

**Results:**

We included 4 randomized controlled trials (RCTs) and 1 controlled clinical trial (CCT) for the benefits and harms of screening, 1 RCT for comparative benefits and harms of different screening strategies, 32 validation cohort studies for the calibration of risk prediction tools (26 of these reporting on the Fracture Risk Assessment Tool without [i.e., clinical FRAX], or with the inclusion of bone mineral density (BMD) results [i.e., FRAX + BMD]), 27 RCTs for the benefits of treatment, 10 systematic reviews for the harms of treatment, and 12 studies for the acceptability of screening or initiating treatment.

In females aged 65 years and older who are willing to independently complete a mailed fracture risk questionnaire (referred to as “selected population”), 2-step screening using a risk assessment tool with or without measurement of BMD probably (*moderate certainty*) reduces the risk of hip fractures (3 RCTs and 1 CCT, *n* = 43,736, absolute risk reduction [ARD] = 6.2 fewer in 1000, 95% CI 9.0–2.8 fewer, number needed to screen [NNS] = 161) and clinical fragility fractures (3 RCTs, *n* = 42,009, ARD = 5.9 fewer in 1000, 95% CI 10.9–0.8 fewer, NNS = 169). It probably does not reduce all-cause mortality (2 RCTs and 1 CCT, *n* = 26,511, ARD = no difference in 1000, 95% CI 7.1 fewer to 5.3 more) and may (*low certainty*) not affect health-related quality of life. Benefits for fracture outcomes were not replicated in an offer-to-screen population where the rate of response to mailed screening questionnaires was low. For females aged 68–80 years, population screening may not reduce the risk of hip fractures (1 RCT, *n* = 34,229, ARD = 0.3 fewer in 1000, 95% CI 4.2 fewer to 3.9 more) or clinical fragility fractures (1 RCT, *n* = 34,229, ARD = 1.0 fewer in 1000, 95% CI 8.0 fewer to 6.0 more) over 5 years of follow-up. The evidence for serious adverse events among all patients and for all outcomes among males and younger females (<65 years) is very uncertain. We defined overdiagnosis as the identification of high risk in individuals who, if not screened, would never have known that they were at risk and would never have experienced a fragility fracture. This was not directly reported in any of the trials. Estimates using data available in the trials suggest that among “selected” females *offered* screening, 12% of those meeting age-specific treatment thresholds based on clinical FRAX 10-year hip fracture risk, and 19% of those meeting thresholds based on clinical FRAX 10-year major osteoporotic fracture risk, may be overdiagnosed as being at high risk of fracture. Of those identified as being at high clinical FRAX 10-year hip fracture risk and who were *referred for BMD assessment*, 24% may be overdiagnosed. One RCT (*n* = 9268) provided evidence comparing 1-step to 2-step screening among postmenopausal females, but the evidence from this trial was very uncertain.

For the calibration of risk prediction tools, evidence from three Canadian studies (*n* = 67,611) without serious risk of bias concerns indicates that clinical FRAX-Canada may be well calibrated for the 10-year prediction of hip fractures (observed-to-expected fracture ratio [O:E] = 1.13, 95% CI 0.74–1.72, *I*^2^ = 89.2%), and is probably well calibrated for the 10-year prediction of clinical fragility fractures (O:E = 1.10, 95% CI 1.01–1.20, *I*^2^ = 50.4%), both leading to some underestimation of the observed risk. Data from these same studies (*n* = 61,156) showed that FRAX-Canada with BMD may perform poorly to estimate 10-year hip fracture risk (O:E = 1.31, 95% CI 0.91-2.13, *I*^2^ = 92.7%), but is probably well calibrated for the 10-year prediction of clinical fragility fractures, with some underestimation of the observed risk (O:E 1.16, 95% CI 1.12–1.20, *I*^2^ = 0%). The Canadian Association of Radiologists and Osteoporosis Canada Risk Assessment (CAROC) tool may be well calibrated to predict a category of risk for 10-year clinical fractures (low, moderate, or high risk; 1 study, *n* = 34,060). The evidence for most other tools was limited, or in the case of FRAX tools calibrated for countries other than Canada, very uncertain due to serious risk of bias concerns and large inconsistency in findings across studies.

Postmenopausal females in a primary prevention population defined as <50% prevalence of prior fragility fracture (median 16.9%, range 0 to 48% when reported in the trials) and at risk of fragility fracture, treatment with bisphosphonates as a class (median 2 years, range 1–6 years) probably reduces the risk of clinical fragility fractures (19 RCTs, *n* = 22,482, ARD = 11.1 fewer in 1000, 95% CI 15.0–6.6 fewer, [number needed to treat for an additional beneficial outcome] NNT = 90), and may reduce the risk of hip fractures (14 RCTs, *n* = 21,038, ARD = 2.9 fewer in 1000, 95% CI 4.6–0.9 fewer, NNT = 345) and clinical vertebral fractures (11 RCTs, *n* = 8921, ARD = 10.0 fewer in 1000, 95% CI 14.0–3.9 fewer, NNT = 100); it may not reduce all-cause mortality. There is low certainty evidence of little-to-no reduction in hip fractures with any individual bisphosphonate, but all provided evidence of decreased risk of clinical fragility fractures (moderate certainty for alendronate [NNT=68] and zoledronic acid [NNT=50], low certainty for risedronate [NNT=128]) among postmenopausal females. Evidence for an impact on risk of clinical vertebral fractures is very uncertain for alendronate and risedronate; zoledronic acid may reduce the risk of this outcome (4 RCTs, *n* = 2367, ARD = 18.7 fewer in 1000, 95% CI 25.6–6.6 fewer, NNT = 54) for postmenopausal females. Denosumab probably reduces the risk of clinical fragility fractures (6 RCTs, *n* = 9473, ARD = 9.1 fewer in 1000, 95% CI 12.1–5.6 fewer, NNT = 110) and clinical vertebral fractures (4 RCTs, *n* = 8639, ARD = 16.0 fewer in 1000, 95% CI 18.6–12.1 fewer, NNT=62), but may make little-to-no difference in the risk of hip fractures among postmenopausal females. Denosumab probably makes little-to-no difference in the risk of all-cause mortality or health-related quality of life among postmenopausal females. Evidence in males is limited to two trials (1 zoledronic acid, 1 denosumab); in this population, zoledronic acid may make little-to-no difference in the risk of hip or clinical fragility fractures, and evidence for all-cause mortality is very uncertain. The evidence for treatment with denosumab in males is very uncertain for all fracture outcomes (hip, clinical fragility, clinical vertebral) and all-cause mortality.

There is moderate certainty evidence that treatment causes a small number of patients to experience a non-serious adverse event, notably non-serious gastrointestinal events (e.g., abdominal pain, reflux) with alendronate (50 RCTs, *n* = 22,549, ARD = 16.3 more in 1000, 95% CI 2.4–31.3 more, [number needed to treat for an additional harmful outcome] NNH = 61) but not with risedronate; influenza-like symptoms with zoledronic acid (5 RCTs, *n* = 10,695, ARD = 142.5 more in 1000, 95% CI 105.5–188.5 more, NNH = 7); and non-serious gastrointestinal adverse events (3 RCTs, *n* = 8454, ARD = 64.5 more in 1000, 95% CI 26.4–13.3 more, NNH = 16), dermatologic adverse events (3 RCTs, *n* = 8454, ARD = 15.6 more in 1000, 95% CI 7.6–27.0 more, NNH = 64), and infections (any severity; 4 RCTs, *n* = 8691, ARD = 1.8 more in 1000, 95% CI 0.1–4.0 more, NNH = 556) with denosumab. For serious adverse events overall and specific to stroke and myocardial infarction, treatment with bisphosphonates probably makes little-to-no difference; evidence for other specific serious harms was less certain or not available. There was low certainty evidence for an increased risk for the rare occurrence of atypical femoral fractures (0.06 to 0.08 more in 1000) and osteonecrosis of the jaw (0.22 more in 1000) with bisphosphonates (most evidence for alendronate). The evidence for these rare outcomes and for rebound fractures with denosumab was very uncertain.

Younger (lower risk) females have high willingness to be screened. A minority of postmenopausal females at increased risk for fracture may accept treatment. Further, there is large heterogeneity in the level of risk at which patients may be accepting of initiating treatment, and treatment effects appear to be overestimated.

**Conclusion:**

An offer of 2-step screening with risk assessment and BMD measurement to *selected* postmenopausal females with low prevalence of prior fracture probably results in a small reduction in the risk of clinical fragility fracture and hip fracture compared to no screening. These findings were most applicable to the use of clinical FRAX for risk assessment and were not replicated in the *offer-to-screen* population where the rate of response to mailed screening questionnaires was low. Limited direct evidence on harms of screening were available; using study data to provide estimates, there may be a moderate degree of overdiagnosis of high risk for fracture to consider. The evidence for younger females and males is very limited. The benefits of screening and treatment need to be weighed against the potential for harm; patient views on the acceptability of treatment are highly variable.

**Systematic review registration:**

International Prospective Register of Systematic Reviews (PROSPERO): CRD42019123767.

**Supplementary Information:**

The online version contains supplementary material available at 10.1186/s13643-023-02181-w.

## Background

### Rationale for the systematic reviews

There is no international consensus on the recommended approach to screening and subsequent treatment to prevent fragility fractures [1]. Screening has traditionally focused on measuring bone mineral density (BMD) with intervention in those with low bone mass, often referred to as osteoporosis [2]. More recent evidence suggests that fracture risk prediction may be improved by instead considering an array of clinical risk factors, alone or in addition to BMD, which may be incorporated into risk prediction tools to estimate the absolute short- to mid-term risk of fracture [2].

The 2010 Osteoporosis Canada screening strategy (presence of any of various clinical risk factors) has low sensitivity in identifying females aged 50 to 64 years for BMD testing who later experience a major osteoporotic fracture [3]. In addition, the screening strategy has not been evaluated in a randomized controlled trial (RCT), indicating that updated screening and treatment algorithms that incorporate the most recent evidence are needed. Since 2018, three RCTs have been published that integrate a 2-step approach to screening to prevent fragility fractures (i.e., risk assessment followed by BMD measurement in those exceeding a certain risk threshold, but without shared decision-making) [4–6]. A systematic review published in 2020 [7], after we began this review, reported on the effects of screening from these three trials on fractures and all-cause mortality. The review had slightly different eligibility criteria than ours (thus two studies included in our review are not included), did not address overdiagnosis (defined later), and did not review additional aspects such as alternative screening strategies or patient perspectives related to recommendations about screening in primary care.

Because randomized trials on screening were not anticipated to evaluate all possible screening tools and outcomes (e.g., harms from the treatment provided to those at high risk), we have included reviews on these topics to determine whether certain screening tools may be interchangeable, and whether treatment harms may impact the main screening recommendation.

### Description and burden of the condition

Fragility fractures are those that occur without stimulus during normal daily activities or secondary to minor incidents that in healthy adults would not normally result in a fracture [8]. Major independent risk factors for fragility fracture include low bone density, chronic use of certain medications (e.g., glucocorticoids), older age, female sex, low body weight, a personal or family history of fracture, a history of falls, smoking, higher levels of alcohol use, and living with type 2 diabetes and/or rheumatoid arthritis [9–14]. Advancing age, especially among postmenopausal females and older males [15], and menopausal status [16, 17] are strong predictors of fragility fracture, as is low bone density [18]. A reduction in bone mass and quality is a common consequence of the aging process.

Fragility fractures impose a substantial burden on societies worldwide [19]. By the year 2040, it has been projected that more than 319 million people globally will be considered to be at high risk of fragility fracture (based on the Fracture Risk Assessment Tool without incorporating BMD results [clinical FRAX]) [20]. In Canada in 2015/16, the incidence of hip fractures among people aged 65 to 69 years was 87 per 100,000 and increased steeply with advancing age to a rate of 1156 per 100,000 in 85 to 89-year-olds [21]. Fragility fractures, particularly hip and clinical vertebral fractures, can result in significant morbidity (e.g., decreased mobility, pain, reduced quality of life) and increase the risk of mortality in the 5 years post-fracture [22–24]. Fragility fractures have been noted to result in more hospitalized days than either stroke or myocardial infarction [25].

### Screening for the primary prevention of fragility fractures

Screening in primary care aims to decrease the risk of future fragility fractures among those without a prior fracture, and to reduce fracture-related morbidity, mortality, and costs. Harms may be related to the screening test itself (e.g., minimal radiation exposure from dual X-ray absorptiometry [DXA]) [26] or the psychosocial or physical (if harmed from treatment) consequences of being labelled “at risk” [27, 28], which may be due to an inaccurate estimation of fracture risk (i.e., due to a risk prediction tool that is poorly calibrated), and/or detection of excess risk among people who, had they not been screened, would never have known their risk nor experienced a fracture. Though considered by the Task Force to be the ideal approach, shared decision-making for screening and subsequent treatment may not be the standard of care across Canada; many primary care providers may instead screen all people without a prior fracture for risk (e.g., using available risk prediction tools and/or offer of BMD assessment) and consider patients eligible for treatment when screening places them within pre-specified thresholds of BMD or fracture risk. It may instead be ideal to use shared decision-making during the clinical encounter, allowing patients to make informed decisions about screening and treatment after weighing the possible benefits against the potential harms. Information from screening can then be used, along with patient preferences, to consider preventive treatment among those who consider themselves to be at a high fracture risk.

There is large variation in the screening approaches suggested by international guidelines, which often consider the population burden of fragility fractures and mortality, competing societal priorities, and resource availability [1, 29]. A variety of approaches may be used within a single screening program, with recommendations often differing by population group based on age, sex, or menopausal status [1, 29]. Common approaches include (a) a one-step direct to BMD approach (e.g., in females >65 years old in Canada [30] and the USA [31]); and (b) a 2-step approach incorporating the assessment of absolute fracture risk followed by BMD assessment in individuals exceeding a pre-defined threshold [29]. The findings of BMD assessment may then be used independently or incorporated into revised clinical risk scores. Clinical risk factors alone may be used to estimate risk in circumstances where BMD is unavailable, but this is not recommended by current North American guidelines [30–35]. There are at least 12 published fracture risk prediction tools available [36, 37]; however, not all tools are easily accessible to clinicians nor have all tools been calibrated for Canada or validated in populations outside of their derivation cohort, limiting their use [38].

Treatment thresholds vary considerably across countries [1, 29, 39]. A common threshold for treatment used in Canada [30, 40], the USA [41], and several other countries is a fixed 10-year major osteoporosis-related fracture probability ≥20% [39]. In some countries (not Canada), a 10-year hip fracture probability ≥3% may also be used [39]. Other approaches include the use of variable thresholds based on age [39], and hybrid models that incorporate both age-based and fixed thresholds [42–44]. Few existing guidelines incorporate shared decision-making [45, 46], but ideally this could be applied to determine the point at which an individual patient, informed about the benefits and risks, would want to contemplate treatment. Bisphosphonates (i.e., alendronate, risedronate, or zoledronic acid) are the most commonly used first-line treatments for the prevention of fragility fractures [47, 48]. Denosumab may also sometimes be considered [47, 48], but this is less common due to its higher cost compared to bisphosphonates. Changing lifestyle factors (e.g., diet, exercise) and fall prevention are other approaches to preventing fragility fractures [30] but were not in the scope of these systematic reviews.

According to a systematic review commissioned by the United States Preventive Services Task Force (USPSTF) with a comprehensive search in 2016, compared to placebo, treatment with bisphosphonates probably reduces the risk of nonvertebral and vertebral fractures (moderate certainty), but may make little-to-no difference in the risk of hip fractures (low certainty) in females [37]. There was low certainty evidence for reduction in all fracture types with denosumab in females [37]. Evidence for males was limited across all pharmacologic treatments of interest [37]. The review authors did not rate the certainty for all clinical fractures, as is of interest for the current review, and updating the evidence may change findings for some outcomes. Various harms may be associated with treatment to various degrees, with some such as mild upper gastrointestinal distress being fairly benign. Others such as serious infections or cardiac events, osteonecrosis of the jaw, and atypical femoral fractures are potentially highly concerning [49].

The effectiveness of treatment relies on high uptake and adherence [50]. However, uptake of pharmacologic treatment is often low, and adherence tends to diminish over time [51]. Low uptake and adherence may be related to a variable assessment of the balance of benefits and harms by individual patients. Though shared decision-making is incorporated into few existing screening guidelines [45, 46], a large variation in treatment preferences across patients could support a shared decision-making approach in the place of recommended treatment thresholds based solely on fracture risk [52, 53].

### Objectives of systematic reviews

In these reviews, we have synthesized evidence relevant to screening for the primary prevention of fragility fractures and related mortality and morbidity among adults 40 years and older in primary care. The findings are among several considerations (including consultations with patients on outcome prioritization, information on issues of feasibility, acceptability, costs/resources, and equity) that will be used by the Canadian Task Force on Preventive Health Care (“Task Force”) to inform recommendations on screening for the prevention of fragility fractures among adults 40 years and older in Canada. Our key questions (KQs) were as follows:

*KQ1a*: What are the benefits and harms of screening compared with no screening to prevent fragility fractures and related morbidity and mortality in primary care for adults ≥40 years?

*KQ1b*: Does the effectiveness of screening to prevent fragility fractures vary by screening program type (i.e., 1-step vs 2-step) or risk assessment tool?

*KQ2*: How accurate are screening tests at predicting fractures among adults ≥40 years?

*KQ3a*: What are the benefits of pharmacologic treatments to prevent fragility fractures among adults ≥40 years?

*KQ3b*: What are the harms of pharmacologic treatments to prevent fragility fractures among adults ≥40 years?

*KQ4*: For patients ≥40 years, what is the acceptability (i.e., positive attitudes, intentions, willingness, uptake) of screening and/or initiating treatment to prevent fragility fractures when considering the possible benefits and harms from screening and/or treatment?

Screening and treatment for risk factors related to fractures, such as fall risk, were not considered though the Task Force is currently developing separate recommendations about falls prevention interventions [54].

## Methods

### Terminology

Throughout this report, we refer to “females” and “males”; these terms refer to biological sex (i.e., biological attributes, particularly the reproductive or sexual anatomy at birth) unless otherwise indicated.

### Review conduct

We followed a peer-reviewed protocol [55] for this review which was based on accepted systematic review methodology [56]. The review was registered prospectively in the International Prospective Register of Systematic Reviews (PROSPERO): CRD42019123767. The methods for the systematic review are reported in detail within the protocol [55]; we report on the methods here briefly, focusing on deviations from the original plans. We report the systematic review according to the Preferred Reporting Items for Systematic Reviews and Meta-Analyses 2020 statement [57].

At the protocol stage, members of the Task Force rated outcomes on their importance for clinical decision-making using a 9-point scale according to the Grading of Recommendations Assessment, Development and Evaluation (GRADE) approach [58]. In addition, the findings of surveys and focus groups with patients that were conducted by the Knowledge Translation team at St. Michael’s, Unity Health Toronto, were incorporated into the final outcome ratings. Outcomes rated as critical (7–9/9) were hip fracture, clinical fragility fractures, fracture-related mortality, quality of life or wellbeing, functionality and disability, serious adverse events, and prediction model calibration (KQ2 only). Outcomes rated as important (4–6/9) were all-cause mortality, non-serious adverse events, discontinuation due to adverse events, and overdiagnosis. The outcomes are defined in detail within our protocol [55]. As screening for risk of fracture does not result in a “diagnosis” but rather a risk for a future event, overdiagnosis has not been previously defined in the context of fracture risk assessment. However, as with conditions such as osteoporosis, overdiagnosis generally refers to identifying and labelling people with “problems,” or in this case “risks,” that would never have caused harm [59]. Thus, for the purpose of this review, we defined overdiagnosis as the identification of high risk in individuals who, if not screened, would never have known that they were at risk and would never have experienced a fragility fracture [59]. The systematic review protocol and this report were revised following review by external stakeholders (*n*=7 and *n*=4, respectively). The Task Force and their external clinical experts were involved with developing the scope of the review and the eligibility criteria (*n*=4; see “Acknowledgments”), as well as with interpreting the findings (*n*=2), but were not involved in the selection and risk of bias assessments of studies, data extraction, or analysis.

We reviewed the evidence following a staged approach, beginning by identifying direct evidence from trials (including all controlled trials but prioritizing evidence from RCTs) of primary screening versus no screening (KQ1a). Based on positive evidence from KQ1a, we proceeded to KQ1b, examining the comparative effectiveness of different screening approaches. We reviewed evidence related to the acceptability of screening and/or treatment (KQ4), as well as indirect evidence on the accuracy of screening tests (KQ2), concurrently with KQ1. The accuracy of screening tests was reviewed to better understand whether other well calibrated tools existed outside of those used in the screening trials, which could influence the tool ultimately recommended for screening. Because the Task Force believed that further information on the benefits and harms of pharmacologic treatment could be relevant to their recommendations, we proceeded with KQs 3a (benefits) and 3b (harms). After completing KQ3a on the benefits of treatment, discussions with the Task Force indicated that a rapid overview of reviews approach for KQ3b (harms of treatment) would be adequate to inform decision-making, while reducing the time and resources needed to review the evidence. We therefore amended our planned approach to KQ3b, as described herein.

### Eligibility criteria

Detailed PICOTs for each KQ are shown in Table 1. Here, we report changes from our original plans that occurred during the selection phase. For KQ1 (benefits and harms of screening), we had intended to exclude studies of patients already being treated with anti-fracture drugs and/or with prior fractures at baseline, but some relevant trials included unknown proportions of previously treated and/or fractured patients. The comparator of interest was no screening, but in reality the available trials included some degree of ad hoc screening in the comparison group. We considered these factors within the Grading of Recommendations, Assessment, Development and Evaluation (GRADE) indirectness domain.

**Table 1:** Eligibility criteria for each key question

		Key question 1 a & b (a) Benefits and harms of screening vs no screening (b) Comparative benefits and harms of different screening approaches/tools	Key question 2 Predictive accuracy of screening tests	Key question 3 a & b (a) Benefits of pharmacologic treatments (b) Harms of pharmacologic treatments	Key question 4 Acceptability of screening and/or treatment
**Population**	**Include**	Asymptomatic adults ≥40 years in the general population (≥80% of the sample or mean age -1 standard deviation is ≥40 years) Subgroups for decision-making: age, sex, menopausal status Methods subgroups: diabetes, presence of prior fractures, baseline predicted fracture risk, length of follow-up	Asymptomatic adults ≥40 years in the general population Subgroups for decision-making: age, sex, menopausal status Methods subgroups: treatment with anti-osteoporosis drugs, baseline predicted fracture risk, length of follow-up	**KQ3a:** Adults ≥40 years in the general population who are at risk of fragility fracture **KQ3b:** Adults ≥40 years who are at risk of fragility fracture Subgroups for decision-making: age, sex, menopausal status Methods subgroups (KQ3a): prior fracture, predicted fracture risk, length of follow-up	Adults aged ≥40 years Population subgroups: absolute fracture risk (perceived or actual), prior screening, history of fracture, prior use of anti-osteoporotic medication, prior diagnosis of osteoporosis, level of concern or perceived severity of fractures
	**Exclude**	- Adults <40 years - Treatment with anti-osteoporosis drugs - >50% with prior diagnosis of osteoporosis, prior fragility fracture, endocrine or other disorders related to metabolic bone disease, chronic use of glucocorticoid medications, cancer	- Adults <40 years - >50% with prior diagnosis of osteoporosis, prior fragility fracture, endocrine or other disorders related to metabolic bone disease, chronic use of glucocorticoid medications, cancer	**KQ3a:** - Adults <40 years - > 50% with with prior fragility fracture, endocrine or other disorders related to metabolic bone disease, chronic use of glucocorticoid medications, cancer **KQ3b:** - Adults <40 years - Endocrine or other disorders related to metabolic bone disease, cancer	- Adults <40 years - Current use of anti-osteoporosis drugs (>10% of population) - >50% with prior fragility fracture, endocrine or other disorders related to metabolic bone disease, chronic use of glucocorticoid medications, cancer
**Intervention/ Exposure**	**Include**	Screening^a^ to prevent fragility fracture with any of the following: -Fracture risk assessment alone (validated or non-validated tools) -Bone mineral density (BMD) alone by dual x-ray absorptiometry ± vertebral fracture assessment (VFA)/spinal radiography - Fracture risk assessment followed by BMD if indicated ± vertebral fracture assessment/spinal radiography Treatment is offered for participants meeting “high risk” threshold	Screening tool to prevent fragility fracture using any of the following: - Fracture risk assessment alone (validated or nonvalidated tools) - Bone mineral density (BMD) alone by dual X-ray absorptiometry ± vertebral fracture assessment/spinal radiography - Fracture risk assessment followed by/incorporating BMD ± vertebral fracture assessment (VFA)/spinal radiography Risk assessment tools must be available to clinicians and have been externally validated to predict fragility fractures in a population within a very high human development index country with a fracture rate similar to Canada (i.e., moderate)	Pharmacotherapy currently approved by Health Canada for the treatment of osteoporosis or prevention of fragility fractures that is commonly used in Canada as a first-line treatment: - Bisphosphonates (alendronate, risedronate, zoledronic acid); harms of bisphosphonates as a class will be included when no or very low certainty evidence is available for individual bisphosphonates - Denosumab (exposure is discontinuation of denosumab for rebound fracture outcome) Adjunct calcium and/or vitamin D (but not other drugs) will be included if it is used identically in both the intervention and comparison group	Population may or may not have knowledge of their own fracture risk but must have at least some general scenario or background information on the possible magnitude of benefits and/or harms from screening (same tools as KQ1) or treatment (bisphosphonates or denosumab) for fragility fractures or osteoporosis. OR Investigators solicit the magnitude of benefits and/or harms where screening or treatment is acceptable. Exposure subgroups: different presentation of information
	**Exclude**	- Other screening tests - VFA without BMD	- Tools not externally validated - Tools not available to clinicians - Tools that do not provide absolute fracture risk (CAROC retained due to relevance to Canada) - Other countries (see inclusion) - Other BMD or osteoporosis-related screening tests	- Pharmacotherapies not commonly used in Canada: hormone therapy, etidronate, raloxifene, teriparatide, calcitonin - Drugs used in combination - Off-label pharmaceuticals and dosages - Natural health products, dietary supplements (e.g., vitamins, minerals) - Complex interventions (e.g., pharmacotherapy + exercise)	- Context of screening using other BMD or osteoporosis screening tests - Benefit and harm information about treatments
**Comparator**	**Include**	**KQ1a:** no screening **KQ1b:** another screening strategy or screening using a different risk assessment tool	Not applicable	**KQ3a:** Placebo **KQ3b:** Placebo or no treatment; continuation of denosumab for rebound fracture outcome	− None - Non-active exposure: intervention without information about the possible magnitude of benefits and/or harms of screening or treatment - Information on alternative screening or treatment strategy
	**Exclude**	- Other screening tests - Fracture liaison services	Not applicable	All other comparators	See exposure
**Outcome**	**Include**	**Benefits:** hip fractures, clinical fragility fractures^b^, fracture-related mortality, functionality and disability, quality of life or wellbeing, all-cause mortality **Harms:** serious adverse events^c^ (all serious cardiovascular events, serious cardiac rhythm disturbances, serious gastrointestinal adverse events, gastrointestinal cancer, atypical fractures, osteonecrosis of the jaw, overdiagnosis (defined in Additional file 3), discontinuation due to adverse events, non-serious adverse events (including “any” adverse events)	Calibration (total/average and by differing estimated risks) for 5- and 10-year risk of hip and clinical fragility fractures	**KQ3a:** hip fractures, clinical fragility fractures^b^, fracture-related mortality, functionality and disability, quality of life or wellbeing, all-cause mortality **KQ3b:** serious adverse events^c^ (all serious cardiovascular events, serious cardiac rhythm disturbances, serious gastrointestinal adverse events, gastrointestinal cancer, atypical fractures, osteonecrosis of the jaw, rebound fractures i.e. multiple vertebral fractures, discontinuation due to adverse events, non-serious adverse events (including “any” adverse events; non-serious gastrointestinal adverse events, musculoskeletal pain, dermatologic adverse events, infections)	- Willingness or intentions to screen or initiate treatment - Acceptability of screening or initiating treatment - Uptake of screening or treatment - Absolute risk for fracture to make treatment acceptable - Others as suitable, as reported by authors
	**Exclude**	All other outcomes	Discrimination (we will supplement with findings from 2018 USPSTF systematic review)	All other outcomes	All other outcomes
**Follow-up**	**Include**	≥6 months	Any length of follow-up; to make predictions for 5- or 10-year fracture	≥6 months	Any
	**Exclude**	<6 months	Not applicable	<6 months	Not applicable
**Setting**	**Include**	Primary health care	Primary health care	**KQ3a:** primary health care **KQ3b:** primary health care or long-term care	Primary health care
	**Exclude**	Long-term care	Long-term care	**KQ3a:** long-term care **KQ3b:** all other settings	Long-term care or hospital
**Study design**	**Include**	Randomized controlled trials, clinical controlled trials (if needed)^d^; manuscripts, reports, abstracts, dissertations, clinical trials registers if data are available	Prospective or retrospective cohort studies; single arms of randomized trials; manuscripts, reports, abstracts, dissertations, clinical trials registers if data are available	**KQ3a:** randomized controlled trials; manuscripts, reports, abstracts, dissertations, clinical trials registers if data are available **KQ3b:** systematic reviews of randomized trials or observational studies^e^; primary studies for rebound fractures after denosumab discontinuation	Any quantitative primary study design, quantitative data from mixed methods studies
	**Exclude**	Systematic reviews, meta-analyses and pooled analyses; all other primary study designs; non-research; studies only available as gray literature if data are inadequate to assess study design and risk of bias	Systematic reviews, meta-analyses and pooled analyses; all other primary study designs; non-research; studies only available as gray literature if data are inadequate to assess study design and risk of bias	**KQ3a:** Systematic reviews, meta-analyses and pooled analyses; all other primary study designs; non-research; studies only available as gray literature if data are inadequate to assess study design and risk of bias **KQ3b:** primary research, overviews of reviews	Systematic reviews, meta-analyses and pooled analyses; qualitative studies; non-research; studies only available as gray literature if data are inadequate to assess study design and risk of bias
**Language**	**Include**	English or French	English or French	English or French	English or French
	**Exclude**	All other languages	All other languages	All other languages	All other languages
**Date of publication**	**Include**	Any	Any	**KQ3a:** any **KQ3b:** 2015–present; 2020 for primary studies of rebound fractures	1995–present (introduction of bisphosphonates)
	**Exclude**	Not applicable	Not applicable	**KQ3a:** not applicable **KQ3b:** pre-2015 (with exception of previously identified AHRQ review)	Pre-1995

*AHRQ* Agency for Healthcare Research and Quality, *BMD* Bone mineral density, *USPSTF* United States Preventive Services Task Force, *VFA* Vertebral fracture assessment

^a^ Screening includes the intervention, follow-up, referral and/or treatment. Fracture risk assessment tools are considered to be any paper or electronic tool or set of questions using ≥2 demographic and/or clinical risk factors to assess risk of future fracture

^b^ Clinical fragility fractures include only symptomatic and radiologically confirmed fractures; sites per author definition, and may be defined as major osteoporotic fracture

^c^ A serious adverse event is any untoward medical occurrence that at any dose (a) results in death, (b) is life-threatening, (c) requires inpatient hospitalization or prolongation of existing hospitalization, (d) results in persistent or significant disability/incapacity, (e) is a congenital anomaly/birth defect, (f) is a medically important event or reaction

^d^ If certainty in the evidence is a barrier to the development of recommendations, and the CTFPHC believes that further evidence from CCTs may influence their recommendations

^e^ We will select one systematic review for each outcome comparison of interest

For KQ2 (predictive accuracy of screening tests), based on clinical expert input, we decided to exclude tools that (a) are not freely available for use by clinicians or (b) do not provide an absolute risk prediction (e.g., provide only a risk categorization; Canadian Association of Radiologists and Osteoporosis Canada Risk Assessment [CAROC] tool retained due to relevance to Canada). We also considered external validations of FRAX-Canada to be most relevant, in comparison to FRAX tools calibrated for other countries. Though our original eligibility included studies from multiple countries, because of the applicability of Canadian studies (when tools are calibrated to this population) and those from Canada in our original search (in 2019) were among the highest quality, our search update in 2021 focused on finding new Canadian studies for which we limited our inclusion. Though not a deviation from our protocol, it is important to note that the discriminative ability of risk prediction tools was not rated as a critical or important outcome by the Task Force. For this reason, we did not review this information systematically within KQ2, but included data reported in a 2018 USPSTF review [60] within our GRADE Summary of Findings Tables for information purposes.

For KQ3a (benefits of treatment), we had planned to exclude the 5 mg/day dosage of alendronate but later included it as well as mixed doses (e.g., 5 mg followed by 10 mg) based on clinical expert input. This decision was supported by the apparent uncertainty about the superiority of the 10mg/day dose and the likelihood of some variability in the doses used in practice. For KQ3b (harms of treatment), we relied on systematic reviews published since 2015 rather than primary studies, as originally intended (see Review Conduct). We included the one most appropriate systematic review per outcome comparison by considering comprehensiveness (likelihood that the search captured all relevant studies, informed by domain 2 in the Risk Of Bias In Systematic reviews [ROBIS] tool [61]); recency (date of last search); and other relevant features (e.g., availability of subgroup and/or adjusted analyses; availability of absolute event rates for the pooled effect). We included systematic reviews of bisphosphonates as a class only for serious adverse events where findings were very uncertain for individual drugs (i.e., additional data may be useful). For rebound fractures (i.e., fractures resulting from increased bone turnover and reductions in BMD after stopping treatment) from denosumab, we compared discontinuation of denosumab to persistence of denosumab or discontinuation of placebo, based on Task Force input about this being the most relevant available comparison. We also added “multiple vertebral fractures” as the most valid potential outcome to capture the effects of rebound fractures. Further, because the reviews were limited on reporting rebound fractures, we added a search for recent (2020 onwards) primary studies for this outcome. For non-serious adverse events, we included: non-serious gastrointestinal adverse events, musculoskeletal pain, dermatologic adverse events, and infections. There were no changes to the original eligibility criteria for KQ4 (acceptability of screening/treatment).

### Literature search and selection of studies

The approach and dates used to search for and select studies for inclusion in the systematic reviews for each KQ are shown in Table 2. Briefly, for KQs 1 (benefits and harms of screening), 2 (predictive accuracy of screening tests), and 3a (benefits of treatment), we integrated eligible studies published up to 2016 from an existing systematic review by the USPSTF [60]. Due to differences in eligibility criteria, we also checked the USPSTF’s excluded studies list and the reference lists of other systematic reviews and major guidelines to identify studies published before 2016 that would have been excluded from the USPSTF review but met our inclusion criteria (e.g., studies that the USPSTF judged to have serious risk of bias concerns, and those examining the comparative effectiveness of screening approaches). We did not integrate studies from existing reviews for KQs 3b (harms of treatment) or 4 (acceptability) and instead relied solely on our search strategies.

**Table 2:** Approach to search and selection of studies for each key question

	Key question 1 a & b (a) Benefits and harms of screening vs no screening (b) Comparative benefits and harms of screening approaches/ tools	Key question 2 Predictive accuracy of screening tests	Key question 3 a & b (a) Benefits of pharmacologic treatments (b) Harms of pharmacologic treatments	Key question 4 Acceptability of screening and/or treatment
**Search**				
Approach	Integration of eligible studies from 2018 USPSTF review, with search from 2016 onward	Integration of eligible studies from 2018 USPSTF review, with search from 2016 onward	**KQ3a:** integration of eligible studies from 2018 USPSTF review, with search update from 2016 onward **KQ3b:** (i) search for reviews from 2015 onward; (ii) search for primary studies on rebound fractures from discontinuation of denosumab from 2020	Search from 1995 (introduction of bisphosphonates)
Search Strategy	See protocol	See protocol	**KQ3a:** see protocol **KQ3b:** Additional file 2	See protocol
Concepts	Bone health, fractures, osteoporosis, screening	Bone health, fractures, osteoporosis, screening tests, risk assessment, prognosis	Osteoporosis, drug treatment, harms (in general and specific harms; KQ3b only)	Bone health, fractures, screening, risk assessment, osteoporosis - diagnosis, prevention, treatment, risk, decision-making, acceptability
Databases	Ovid Medline, Ovid Embase, Wiley Cochrane Library	Ovid Medline, Ovid Embase, Wiley Cochrane Library	Ovid Medline, Ovid Embase (KQ3a only), Wiley Cochrane Library	Ovid Medline, Ovid Embase, Wiley Cochrane Library, PsycINFO
Additional sources	Clinicaltrials.gov; WHO ICTRP; scanned reference lists of systematic reviews, included studies, major guidelines; contacting authors	Clinicaltrials.gov; WHO ICTRP; scanned reference lists of systematic reviews, included studies, major guidelines; contacting authors	**KQ3a:** Clinicaltrials.gov; WHO ICTRP; scanned reference lists of systematic reviews, included studies, major guidelines; contacting authors **KQ3b:** PROSPERO, Epistemonikos	Scanned reference lists of systematic reviews, included studies; contacting authors
Date of search(es)	2016 to 8 July 2019 Update in databases 4 April 2022	2016 to 5 July 2019 Update in databases 1 June 2021	**KQ3a:** 2016 to 2 March 2020 **KQ3b:** (i) 2015 to 24 June 2020; (ii) 2020 to 18 June 2021	1995 to 4 July 2019 Update in databases 1 June 2021
**Selection**				
Titles and abstracts	Liberal-accelerated (i.e., only requiring one reviewer to include to full text review)	Liberal-accelerated	**KQ3a:** liberal-accelerated **KQ3b:** single reviewer	Liberal-accelerated
Full Text	Duplicate	Duplicate	**KQ3a:** duplicate **KQ3b:** single reviewer	Duplicate
Final inclusion	By consensus, with consultation of a third reviewer if needed	By consensus, with consultation of a third reviewer if needed	By consensus, with consultation of a third reviewer if needed	Single reviewer, with consultation of a second reviewer if needed

*KQ* Key question, *WHO ICTRP* World Health Organization International Clinical Trials Registry Platform, *USPSTF* United States Preventive Services Task Force

A research librarian developed and implemented comprehensive peer-reviewed [62] electronic search strategies for each KQ (see protocol [55]; Additional file 2 for KQ3b on harms of treatment). We also searched clinical trials registries and scanned the reference lists of relevant systematic reviews and the included studies. We exported the database results to EndNote (version X7 or X9, Clarivate Analytics, Philadelphia, PA) and removed duplicates before screening the records in DistillerSR (Evidence Partners Inc., Ottawa, Canada).

### Data extraction and risk of bias assessment

We had initially planned to rely (with verification) on data from the USPSTF systematic review [60] for older studies. However, during review conduct differences in outcome definitions, subgroups of interest, and methodology (e.g., updated version of the PROBAST tool became available) became apparent. Therefore, following a pilot round (with two reviewers), one reviewer independently extracted data from all included studies into a standardized form in Excel (Microsoft Corporation, Redmond, WA). Study characteristics were then verified by a second reviewer and outcome data were extracted in duplicate, with final data based on consensus. The full list of data extraction items is available in our protocol [55]. Since we altered our approach to rely on systematic reviews for KQ3b (harms of treatment), we additionally collected the following: databases searched and date of last search, scope of systematic review and selection criteria for the included studies, number and design of primary studies included, number of participants and summary characteristics, summary of interventions and comparators included, risk of bias/quality appraisal tool used to appraise included studies, analyses methods, and summary statistics for outcomes of interest.

Outcome-level risk of bias was appraised for each included study in duplicate (one reviewer with verification for KQ3b [harms of treatment]) using published design-specific tools as applicable (Cochrane risk of bias tool version 2011 for KQs 1 and 3a [63], PROBAST for KQ2 [64], AMSTAR 2 for KQ3b [65]), with final ratings determined by consensus. For KQ3b (harms of treatment), we also extracted information on the risk of bias of the systematic reviews’ included studies, but if these were missing, we did not perform these appraisals anew. Since there is no commonly used or accepted tool to assess risk of bias in studies of acceptability, we assessed risk of bias in the studies included for KQ4 (acceptability of screening/treatment) by considering the risk of bias subdomains within the GRADE guidance for assessing the certainty of evidence in studies of the importance of outcomes or patient values and preferences [66] (adapted to be suitable to acceptability). Assessments of risk of bias informed the study limitations domain of our assessments of the certainty of the body of evidence.

### Synthesis

We performed meta-analyses when appropriate based on clinical and methodological similarity across studies. For KQ1 (benefits and harms of screening), we pooled data for each outcome via pairwise meta-analysis using the DerSimonian and Laird random effects model [67] in Review Manager (version 5.3, The Cochrane Collaboration, Copenhagen, Denmark). Due to differences in the populations analyzed across studies, we pooled data from different population perspectives separately. The perspectives analyzed were (a) *offer-to-screen*, which included all those randomized and offered (by mail), but not necessarily completing any screening, and in the group they were originally assigned; (b) *offer-to-screen in selected populations*, which included those who independently completed a mailed clinical Fracture Risk Assessment (FRAX) questionnaire, in the group they were originally assigned (randomized before or after completion, depending on the trial); and (c) *acceptors*, which included those randomized who ultimately completed the entire screening process (i.e., clinical FRAX and BMD if meeting the risk threshold). In one study [68], hip fractures were presented only as counts (rather than number of participants with ≥1 fracture); we included this study among the others in meta-analysis based on clinical and statistical expert input indicating that the outcome was sufficiently rare that count and rate data would be similar. As described previously, we defined overdiagnosis as the identification of high risk in individuals who, if not screened, would never have known that they were at risk and would never have experienced a fragility fracture [59]. As this was not reported directly in any trial, we estimated this using available data from two trials, considering the proportion of participants exceeding the risk threshold in the study and the mean risk in these patients (see Additional file 3). For KQ3a (benefits of treatment), we pooled data by outcome as in KQ1; in several studies, there were zero events reported in one or both groups, and in these cases, we performed random effects meta-analysis using the reciprocal of the opposite treatment arm size correction for pooled odds ratio [69] in Stata (StataCorp, College Station, TX). We pooled data by sex and for each drug separately, but also performed an “all bisphosphonates” analysis including data from studies reporting on either of alendronate, risedronate, and zoledronic acid. For KQ3b, we report pooled effects directly as they were presented within the included systematic reviews and did not perform any re-analyses of data from primary studies.

We calculated absolute effects for each outcome comparison by applying the relative risk or odds ratio from the meta-analysis to the median control group event rates from the included studies [70]. For KQ1, we also incorporated a sensitivity analysis by calculating absolute effects using an assumed risk based on the general population in Canada (45 to 54 years and ≥65 years) [15, 71]. If statistically significant, we calculated the number needed to screen for an additional beneficial outcome (NNS), number needed to treat for an additional beneficial outcome (NNT), or number needed to treat for an additional harmful outcome (NNH) [72].

For KQ2 (predictive accuracy of screening tests), we chose not to pool the overall findings on calibration for most tools due to high levels of heterogeneity that could not be explained by a priori subgroups (age, sex, baseline risk within and across studies). We present the calibration findings by tool for both the population overall (average) and a summary of calibration within categories (e.g., quintiles, deciles) of baseline risk. We did pool data for the studies without high risk of bias reporting on the FRAX-Canada tool; we considered data from this subgroup to be most reliable and most directly applicable to Canada. These studies presented no major risk of bias concerns that would reduce our certainty in the findings, whereas all others generally had multiple major reasons to be seriously concerned about risk of bias. In all cases, we used the restricted maximum likelihood estimation approach and the Hartun-Knapp-Sidnick-Jonkman correction to derive 95% CIs [73, 74]. We rescaled total observed versus expected fracture event ratios (O:E) and their variance (standard error) on the natural log scale prior to entering these into meta-analysis (or displaying on forest plots) to achieve approximate normality [75–77].

For KQ4 (acceptability of screening/treatment), we performed a narrative synthesis following the guidance of Popay et al. [78], recognizing that our question of acceptability differs to some extent from questions about interventions or implementation factors.

Across KQs, we considered several potential population and intervention/exposure subgroups of interest, for example in KQ1 analyses were stratified by age, while in KQ3a we analyzed data for postmenopausal females separately from males. In several cases, data on characteristics of interest were unavailable in the included study reports (e.g., baseline fracture risk). We also considered within-study subgroup analyses when these were available. We performed sensitivity analyses by risk of bias, applicability concerns (e.g., high-risk population in KQ1), and outcome ascertainment methods (e.g., clinical fragility fractures in KQ3a). When analyses for interventions contained at least eight trials of varying size, we assessed for small study bias using funnel plots and Harbord’s test (KQ3a) [79].

### Rating certainty of evidence and drawing conclusions

Two reviewers rated the certainty in the evidence for each outcome comparison of interest and agreed on the final rating and conclusion statements. Our certainty of evidence appraisals for effects of interventions were based on the absolute effects and considered only the direction of effect and not its magnitude. For KQ1 (benefits and harms of screening), KQ3 (benefits and harms of treatment), and KQ4 (acceptability of screening/treatment), we assessed the certainty of the evidence following the GRADE approach [80–86]. In the absence of published guidance on GRADE for reviews of risk prediction models, for KQ2 (predictive accuracy of screening tests) calibration outcomes, we considered input from an expert in GRADE to modify existing guidance [87] and assist in rating the evidence and developing conclusions. We decided a priori to consider tools to be well calibrated when the O:E ratio across the study populations consistently fell between 0.8 and 1.2 (20% over- or underestimation, respectively) [88]. We then rated certainty for one of four possible conclusions: well calibrated (O:E ratio consistently between 0.8 and 1.2), underestimation (O:E ratio >1.2 and adequately precise to draw clinically meaningful conclusions), overestimation (O:E ratio <0.8 and adequately precise to draw clinically meaningful conclusions), or poorly calibrated (wide variation across studies including over- and underestimation; unable to draw a clinically meaningful conclusion) (Additional file 4). For KQ3b, we relied preferentially on the certainty of evidence ratings presented by the included systematic reviews, with modifications if needed to align with our other appraisals. When these were not reported by the included systematic reviews, we performed our own GRADE appraisals, relying on the data available in the systematic reviews. When the data required to perform full evidence appraisals were missing from the included systematic reviews, we collected data from the included primary studies (if ≤5 studies) and/or made assumptions, as described in Additional file 4.

We developed informative statements based on our certainty in the evidence for each outcome comparison [89]. We adopted standard wording to describe our findings, using the word “may” together with the direction of effect to describe findings of low certainty and “probably” for those of moderate certainty. When our certainty in the evidence was very low, we describe the evidence only as “very uncertain” [89].

## Results

### KQ1a: What are the benefits and harms of screening compared with no screening to prevent fragility fractures and related morbidity and mortality in primary care for adults ≥40 years?

**Fig. 1:**
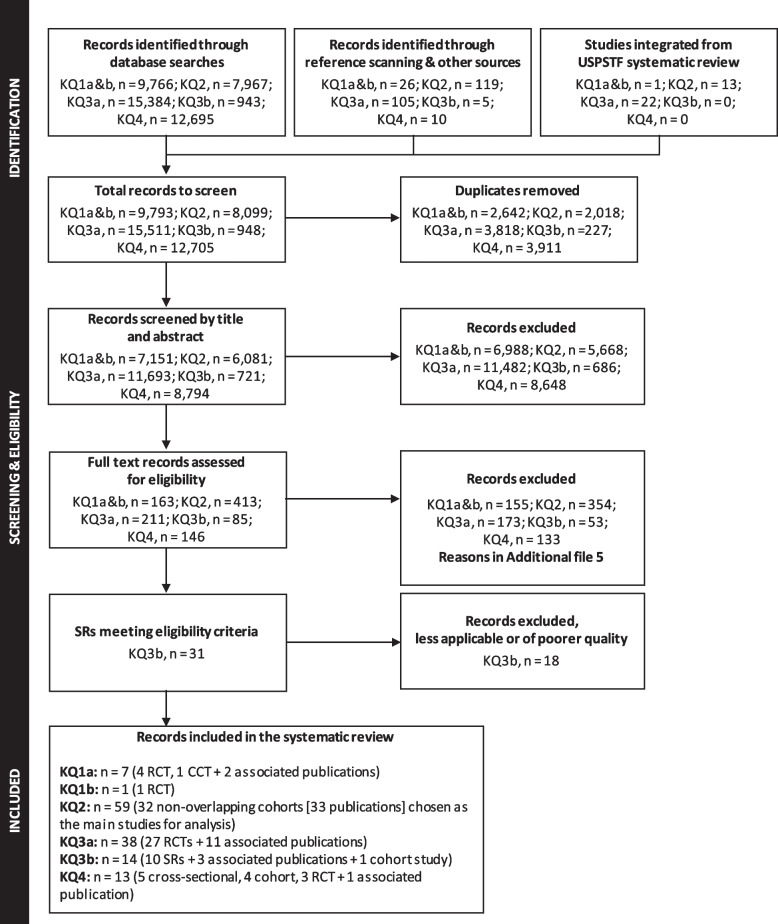
Flow of records through the selection process. Legend: not applicable

Of 7151 unique records retrieved by the searches for KQ1a and b, we assessed 163 for eligibility by full text and included five trials (4 randomized controlled trials [RCT] [4–6, 90], 1 controlled clinical trial [CCT] [68]) and two associated publications [91, 92] for KQ1a, and one RCT for KQ1b [93] (Fig. 1). Studies excluded after full text appraisal are listed with reasons in Additional file 5.

#### Study characteristics

Table 3 shows the characteristics of the included trials for KQ1a. The trials were conducted in countries with a moderate-to-high baseline fracture risk [94]: Denmark (ROSE [5]), the Netherlands (SALT [4]), the UK (SCOOP [6] and APOSS [90]), and the USA (Kern CCT [68]). Aside from the Kern CCT, which included a relatively equal proportion of males and females ≥65 years old [68], the trials included populations of exclusively peri-menopausal (aged 45 to 54 years) [90] or postmenopausal (mean ages 70 to 75.5 years; range 65 to 90 years) [4–6] females. When reported, between 10 and 44% of the study population had a prior fracture [4–6]. The proportion of participants with a prior fracture was highest in the SALT trial (44%), which enrolled females who reported at least one clinical risk factor on the clinical FRAX tool [4]. Participants were not treatment-naïve in all trials; in particular, the APOSS trial allowed enrollment by females with past use of hormone replacement therapy [90] and 11% of participants in the ROSE trial were taking anti-osteoporosis medications at baseline [5].

**Table 3:** Characteristics of studies included for key questions 1a&b on the benefits and harms of screening versus no screening, and the comparative benefits and harms of different screening approaches

Study; design; country; funding; analysis	Population characteristics	Screening approach (***n*** randomized)	Treatment threshold Risk in those meeting threshold	Above treatment threshold; Treatment uptake	Outcomes; follow-up
**KQ1a: benefits and harms of screening versus no screening**					
**Merlijn 2019 (SALT)** [4] RCT Netherlands Foundation, industry, academic Analysis: selected population (high risk)	11,032 (20.5% of 53,794 in age-based sample) females aged 65 to 90 y with ≥1 clinical risk factor; 47% of original sample completed FRAX, but 56% of these were ineligible or did not have a risk factor. Mean (SD) 75.0 (6.7) y; 44% prior fracture (location NR); 1% type 1 diabetes	**Screening (*n*=5575):** 2-step - Completed FRAX-UK - BMD + VFA if ≥ 1 risk factor - 76% of eligible for BMD participated **Usual care (*n*=5457):** completed FRAX-UK; advised to visit GP if ≥ 1 risk factor; 6% underwent DXA and VFA	**Treatment threshold:** any of a) lumbar/thoracic fracture with vertebral height reduction, b) exceeding age-specific FRAX + BMD MOF risk threshold, or c) risk score ≥4 according to Dutch guidelines^a^ **Mean (SD) FRAX + BMD:** 10-y MOF risk: 23.9 (9.6%) 10-y hip fracture risk: 10.6 (10.1)%	**Above treatment threshold:** 1417/4228 (34%) who underwent screening; 25% for the screening group **Self-report of any osteoporosis medication:** 21% in screened (69% with treatment indication); 5% in usual care (mainly bisphosphonates)	**≥1 hip fractures:** self-reported and verified **≥1 MOF (hip, clinical vertebral, wrist, humerus):** self-reported and verified **All-cause mortality:** reported by relatives **Follow-up:** ≥36 months
**Rubin 2018 (ROSE)** [5] RCT Denmark Government, academic Analysis: offer-to-screen; selected population (completed FRAX)	34,229 (18,605 with FRAX; 54.4% of eligible) females aged 65 to 80 y Median (IQR) 71 (8) y; 10% prior fracture (location NR) in those with FRAX; diabetes NR	**Screening (*n*=17,072; 9279 with FRAX):** 2-step - Completed FRAX-Denmark - BMD + VFA when 10-y risk of MOF was ≥15% - 71% of eligible for BMD participated **Usual care (*n*=17,157; 9326 with FRAX):** completed FRAX, risk not calculated; 25% had DXA scan after the index date	**Treatment threshold:** BMD *T*-score at any site ≤2.5; vertebral fracture on VFA. **Median (IQR) FRAX + BMD:** NR in those meeting threshold. Screened group (*n*=5009) with DXA: 10-y MOF risk: 22 (15, 29)% 10-y hip fracture risk: 8.1 (5.6, 13)%	**Above treatment threshold:** 1236/9279 (13%) who completed FRAX; 7% for the screening group **Any osteoporosis medication (pharmacy records):** 23% in screened (80% with treatment indication); 18% in controls	**≥1 hip fracture:** records (ICD-10 codes) **≥1 MOF (hip, clinical vertebral, wrist, humerus):** records (ICD-10 codes) **Follow-up:** median (IQR) 5 (1.3) y
**Shepstone 2018 (SCOOP)** [6, 91] RCT Government, foundation United Kingdom Analysis: selected population (completed FRAX)	12,483 (32.3% of eligible) females aged 70 to 85 y Mean (SD) 75.5 (4.2) y; 24% prior fracture (location NR); diabetes NR	**Screening (*n*=6233):** 2-step - Completed FRAX - BMD when 10-y risk of hip fracture met high risk threshold based on age - 92% of eligible for BMD participated **Usual care (*n*=6250):** completed FRAX, fracture probability not calculated; GP received letter stating patient’s involvement	**Treatment threshold:** exceeding age-specific 10-y hip fracture risk (FRAX-BMD) threshold **Mean (SD) FRAX (no BMD):** 10-y MOF risk: 30.0 (10.7)% 10-y hip fracture risk: 17.9 (10.9)%	**Above treatment threshold:** 898/2790 (32%) who completed FRAX + BMD; 14% for the screening group **Any osteoporosis prescription (GP records):** 1486/6233 (24%) in screened (78% with treatment indication in first 6 months); 16% in controls	**≥1 hip fracture:** self-report, records **≥1 osteoporosis-related fracture (not hands, feet, nose, skull, vertebrae):** self-report; records **All-cause mortality:** registry data, family members, GPs **Health-related quality of life:** self-report via EuroQol-5D, Short-Form 12 Health Survey **Serious AEs:** GPs recorded serious AEs **Follow-up:** 5 y
**Barr 2010 (APOSS)** [90, 92] RCT United Kingdom Foundation, industry Analysis: offer-to-screen; acceptors of screening (completed BMD)	4800 (3128 attended / had complete follow-up; 65% of eligible); peri-menopausal females aged 45 to 54 y Mean (SD) 58.4 (3.7) y; prior fractures and diabetes NR	**Screening (*n*=2400; 1764 attended):** 1-step invitation to be screened by BMD via DXA **Usual care (*n*=2400; 1364 with complete follow-up):** not invited to be screened	**Treatment threshold:** BMD at any site within the lowest quartile of first 1000 women screened **Baseline risk:** NR	**Above treatment threshold:** NR; lowest quartile **Self-reported uptake of any osteoporosis medication >3 months (bisphosphonates, raloxifene, hormone replacement therapy):** 69% in screened (% with treatment indication NR); 59% in controls	**≥1 hip fracture:** self-reported and verified **≥1 MOF (hip, wrist, vertebrae, humerus):** self-report and verified **General health:** self-reported **Health status (2-y follow-up):** self-reported on Short-Form 36 Survey **All-cause mortality:** NR **Follow-up:** median 9.1 y in screened, 8.8 y in controls
**Kern 2005** [68] CCT (non-random allocation based on availability of screening) United States Government, foundation Analysis: selected population (enrolled in another study)	3107 adults ≥65 years (87% of eligible study participants offered screening) Mean (SD) 76.2 (4.9) y; 56% female; <0.1% with radius or ulna fracture in past 5 y, other fractures NR; 1% diabetes	**Screening (*n*=1422):** 1-step offer to be screened by BMD via DXA; 97% completed scans **Usual care (*n*=1685):** not offered BMD scan	**Risk definition:** BMD below age-matched mean of densitometer manufacturer’s reference group **Risk in those meeting threshold:** NR	**Above treatment threshold:** 33% of those completing a DXA scan (392 females, 69 males); 32% in the screening group **Any bone-enhancing medication (includes calcium, multi-vitamins, estrogen, calcitonin, bisphosphonates):** 27% in screened (31% with treatment indication); NR in controls	**Total number of hip fractures:** hospital records (ICD-9 codes); verified against Medicare claims data **All-cause mortality:** surveillance of hospital records and verified against Medicare claims data **Follow-up:** mean 4.9 y
**KQ1b: comparative benefits and harms of different screening approaches**					
**LaCroix 2005 (OPRA)** [93] RCT (3-arm) United States Industry Analysis: offer-to-screen	9268 (3167 [34%] participated) females aged 60 to 80 y Mean (SD) 70.0 (5.6) y; 17% prior fracture; diabetes NR	**Universal screen (*n*=1986; 415 participated):** 1-step invitation to be screened by BMD via DXA **SCORE-based screen (*n*=1940; 576 participated):** 2-step - All completed SCORE - BMD offered if score ≥7 - 74% were eligible for BMD **SOF-based screen (*n*=5342; 2176 participated):** - All completed SCORE - BMD offered if ≥5 clinical risk factors - 7% were eligible for BMD	**Risk definition:** ≥5 fracture risk factors and/or BMD *T*-score <−2.5 for 60–64 y or *z*-score <-0.43 for ≥65 y; prior fracture after age 50 (SOF-based group only) **Risk in those meeting threshold:** NR	**Above treatment threshold:** 28% of those screened in the universal group (6% of allocated); 32% of those completing the SCORE-based tool (7% of allocated); 18% of completing the SOF-based tool (7% of allocated) **Any dispensed prescription for osteoporosis medication (includes alendronate, hormone replacement therapy, calcitonin, raloxifene):** 13% in universal screening (21% of screened), 14% in SCORE-based (20% of screened), 13% in SOF-based (17% of screened) group	**Total number of hip fractures; all non-pathologic (osteoporotic) fractures:** hospitalization and outpatient visit records (ICD-9 codes) **Follow-up:** mean (range) 28 (24–33) months

*AE* Adverse events, *BMD* Bone mineral density, *CCT* Clinical controlled trial, *DXA* Dual-energy X-ray absorptiometry, *FRAX* Fracture Risk Assessment Tool, *GP* General practitioner, *IQR* Interquartile range, *VFA* Vertebral fracture assessment, *MOF* Major osteoporotic fracture, *NR* Not reported, *RCT* Randomized controlled trial, *SD* Standard deviation, *y* years

^a^Bone densitometry and VFA is indicated if the total risk score is ≥4 points (composite of vertebral fracture (4 points), recent fracture after age 50 years (4), age ≥60 years (1), age ≥70 years (1), non-recent fracture after age 50 years (1), additional non-recent fracture after age 50 years on a separate occasion (1), parental hip fracture (1), body weight <60 kg (1), severe immobility or 1 or more falls in the past year (1)). Bisphosphonate treatment is advised if BMD of either femoral neck or lumbar spine shows a *T*-score ≤ −2.5 or if a prevalent vertebral fracture (≥25% height reduction) is present

The three more recent trials (published 2018–2019) [4–6] employed a 2-step approach to screening, whereby all participants completed a mailed questionnaire including data to assess risk with the clinical FRAX tool, and only those surpassing certain risk thresholds were offered BMD assessment. The threshold for BMD assessment varied across trials; in the SALT trial, the entire population had ≥1 risk factor and were offered BMD and vertebral fracture assessment [4], whereas ROSE offered BMD for those with a clinical FRAX-based 10-year major osteoporotic fracture risk ≥15% [6], and SCOOP used age-based thresholds of 10-year hip fracture risk [5]. The two older trials (APOSS [2010] and Kern CCT [2005]) used a one-step direct to BMD screening approach [68, 90]. No trials included a true “no screening” comparator; in all cases, the comparator was usual care, with evidence of varying levels of ad hoc screening and treatment (median 17% treatment rate when this was reported, range 5 to 59% [4–6, 90]) within the comparison groups.

Thresholds for treatment were also variable across the trials. In both the SALT and SCOOP trials, BMD assessment was used to recalculate the 10-year FRAX fracture risk with inclusion of BMD, and treatment was offered when participants exceeded age-specific thresholds [4, 6]; the SALT trial also allowed for several other treatment indications according to Dutch guidelines (e.g., vertebral fracture) [4]. Of note, in the SCOOP trial, only 898 females exceeded a treatment threshold despite 3064 being considered at elevated risk based on fairly similar thresholds but without incorporation of the BMD results into the risk prediction by clinical FRAX. In the ROSE trial, treatment was offered when the BMD *T*-score at any measured site was ≤2.5, and/or a fracture was detected on vertebral fracture assessment [5]. In the two 1-step screening trials, treatment was offered to those in the lowest quartile of BMD, based on the first 1000 participants screened (APOSS) [90], and to those below the age-matched mean of the reference group according to the densitometer’s manufacturer (Kern CCT) [68]. Across the trials, between 7 and 25% of those assigned to screening had indications for treatment; the proportion was highest in the SALT trial, where higher-risk patients were enrolled [4]. The rate of treatment was lowest in the Kern CCT (31% of those with a treatment indication) [68]; among the remaining trials, more than two-thirds (69 to 80%) of those with a treatment indication reported using some form of anti-osteoporosis drugs during follow-up (variable treatments across studies, and sometimes including those such as calcitonin and hormone replacement therapy, which are no longer recommended; see Table 3). It was apparent that most of the treatment provided in the recent RCTs was pharmacologic, though at least one protocol (SALT) mentioned calcium and vitamin D supplementation, as well as notification of a high fall risk, that may have been acted upon by the primary care practitioner.

The trials provided data for hip fractures [4–6, 68, 90], clinical fragility fractures (described as major osteoporotic [4, 5, 90] or osteoporosis-related fractures [6]), serious adverse events [6], all-cause mortality [4, 6, 68, 90], and quality of life or wellbeing [6, 90, 92]; no trials reported on fracture-related mortality, functionality and disability, discontinuation due to adverse events, or non-serious adverse events. Though not directly reported, data were available in two trials to estimate the potential extent of overdiagnosis (see Additional file 3 for calculations) [4, 6]. Because of differences in design and reporting across the trials, we considered three possible population perspectives in our analyses. Two trials (APOSS and ROSE) provided data for an *offer-to-screen* population, whereby all eligible people invited for screening by mail, regardless of actual participation in any screening, were analyzed [5, 90]. The APOSS study also provided data for *acceptors of screening*, where the analysis included only those who attended for BMD measurement and thus completed screening. The SALT, ROSE, and SCOOP trials provided data for what we considered an *offer-to-screen in selected population* approach, because the analyses only included people who independently completed a mailed clinical FRAX questionnaire as part of 2-step screening [4–6]. The Kern CCT [68] also contributed data for this approach, as the sample population for screening was those already enrolled in the Cardiovascular Health Study (i.e., not the general population) [95]. We considered the “selected population” approach to be the one to be most applicable to primary care—where healthcare providers would complete risk assessment tools during the patient visit and then discuss the findings—although the participants in these trials are likely to be more accepting and compliant with screening, and possibly with treatment, than the general population presenting to primary care.

The risk of bias ratings for the included trials for KQ1a are in Table 4. The main risk of bias concerns were related to participant awareness of group assignments and contamination of the control groups in all trials (aforementioned ad hoc screening and treatment, likely to bias the findings toward the null) [4–6, 68, 90], and a high risk of attrition bias in the APOSS trial (42% lost to follow-up) in the offer-to-screen population [90]. The Kern CCT was not randomized, however patients were invited based on age- and sex-stratified random sampling and analyses were adjusted for baseline differences between groups [68]. We rated this trial, as well as the “acceptors” population for the APOSS and the “selected population” in the ROSE trial, to be at unclear risk of selection bias [5, 90], because in these analyses, the participants no longer represented the initially randomized population.

**Table 4:**
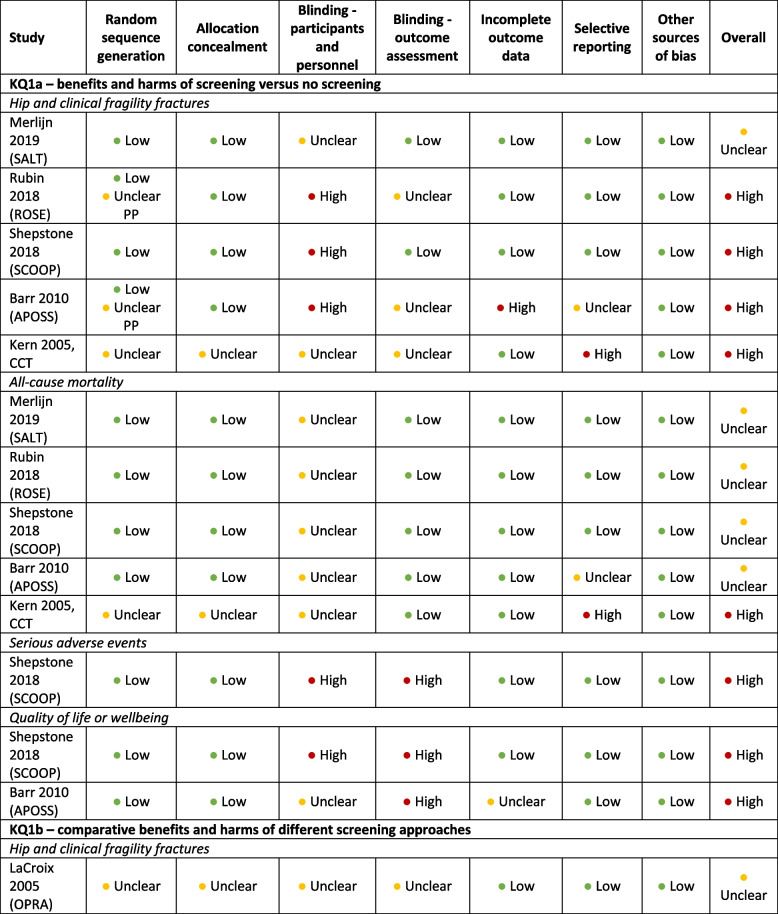
Risk of bias assessments for trials included for KQ1a on the benefits and harms of screening vs. no screening, and KQ1b on the comparative benefits and harms of different screening approaches

*CCT* Clinical controlled trial, *PP* Per protocol

#### Findings

Table 5 summarizes the main findings for KQ1a; Additional file 3 contains the full GRADE Evidence Profiles and Summary of Findings Tables, with explanations for each rating as well as the forest plots, which include the results of statistical tests for subgroup differences. Among females aged 68–80 years, data from one trial showed that a mailed *offer of screening* in the general population may not reduce the risk of hip fractures, clinical fragility fractures, or all-cause mortality during 5 years of follow-up [5]. The evidence is very uncertain for all outcomes from a mailed *offer of screening* with BMD among females aged 45–54 years during 9 years of follow-up (1 trial) [90].

**Table 5:** Summary of findings for KQ1a on the benefits and harms of screening compared with no screening

Study approach	Population; studies; sample size	Follow-up (y)	Assumed population risk^e^	Absolute effects	Certainty^f^	What happens?
**Hip fractures**						
All eligible / offer-to-screen	Females 45–54 y [90]	The evidence from 1 RCT (*n*=2979) is very uncertain.			VERY LOW^a-d^	Very uncertain
	Females 68–80 y 1 RCT; 34,229 [5]	5	Study data: 25 per 1000	0.3 fewer in 1000 (4.2 fewer to 3.9 more)	LOW^a-c^	May not reduce
			General: 20 per 1000	0.2 fewer in 1000 (2.4 fewer to 2.2 more)		
Acceptors of screening	Females 45–54 y [90]	The evidence from 1 RCT (*n*=2604) is very uncertain.			VERY LOW^a-d^	Very uncertain
Offer-to-screen in selected population^g^	Females ≥65 y 3 RCT+1 CCT; 43,736 [4–6, 68, 91]	3 to 5	Study data: 31 per 1000	6.2 fewer in 1000 (9.0 fewer to 2.8 fewer)	MODERATE^c^	Probably reduces
			General: 20 per 1000	4.0 fewer in 1000 (5.8 fewer to 1.8 fewer)		
	Males ≥70 y [68]	The evidence from 1 CCT (*n*=1380) is very uncertain.			VERY LOW^a-d^	Very uncertain
**Clinical fragility fractures**						
All eligible / offer-to-screen	Females 45–54 y [90]	The evidence from 1 RCT (*n*=2979) is very uncertain.			VERY LOW^a-d^	Very uncertain
	Females 68–80 y 1 RCT; 34,229 [5]	5	Study data: 100 per 1000	1.0 fewer in 1000 (8.0 fewer to 6.0 more)	LOW^a-c^	May not reduce
			General: 168 per 1000	1.7 fewer in 1000 (13.4 fewer to 10.1 more)		
Acceptors of screening	Females 45–54 y [90]	The evidence from 1 RCT (*n*=2604) is very uncertain.			VERY LOW^a-d^	Very uncertain
Offer-to-screen in selected population^g^	Females ≥65 y 3 RCT; 42,009 [4–6, 91]	3 to 5	Study data: 84 per 1000	5.9 fewer in 1000 (10.9 fewer to 0.8 fewer)	MODERATE^c^	Probably reduces
			General: 168 per 1000	11.8 fewer in 1000 (21.8 fewer to 1.7 fewer)		
**All-cause mortality**						
All eligible / offer-to-screen	Females 45–54 y 1 RCT; 4800 [90]	9	Study data: The evidence is very uncertain.		VERY LOW^b,d^	Very uncertain
			General: 3 per 1000	No difference in 1000 (0.8 fewer to 1.1 more)	LOW^b,d^	May not reduce
	Females 68–80 y 1 RCT; 34,229 [5]	5	Study data: 118 per 1000	3.5 fewer per 1000 (9.4 fewer to 3.5 more)	LOW^b,d^	May not reduce
			General: 57 per 1000	1.7 fewer per 1000 (4.6 fewer to 1.7 more)		
Offer-to-screen in selected population^g^	Females ≥65 y^h^ 2 RCT+1 CCT; 26,511 [4, 6, 68]	3 to 5	Study data: 89 per 1000	No difference in 1000 (7.1 fewer to 5.3 more)	MODERATE^d^	Probably does not reduce
			General: 57 per 1000	No difference in 1000 (4.6 fewer to 5.1 more)		
**Serious adverse events**						
Offer-to-screen in selected population^g^	Females 70–85 y [6]	The evidence from 1 RCT (*n*=12,483) is very uncertain.			VERY LOW^a,b,d^	Very uncertain
**Health-related quality of life/Wellbeing**						
All eligible / offer-to-screen	Females 45–54 y [90, 92]	9 (self-rated health) 2 (SF-36)	NA	The evidence from 1 RCT (*n*=2979) is very uncertain.	VERY LOW^a-c^	Very uncertain
Offer-to-screen in selected population^g^	Females 70–85 y 1 RCT; 10,661 [6]	5	NA	SF-12 (range 0–100): Mental health: MD −0.30, 95% CI −0.86 to 0.26 Physical health: MD 0.30, 95% CI −0.21 to 0.81 EuroQol-5D (range 0–1): MD 0, 95% CI −0.07 to 0.07	LOW^a,b^	May be little to no difference
**Overdiagnosis**						
Offer-to-screen in selected population^g^	Females 70–85 y (1 RCT; 6,233) [6] 14.4 × (100 − 17.9) /100 = 11.8% overdiagnosed					
	Females 65–90 y (1 RCT; 5575) [4] 25.4 × (100 − 23.9) / 100 = 19.3% overdiagnosed (selected higher-risk population)					
Among those considered at high risk	Females 70–85 y (1 RCT; 3064) [6] 29.3 × (100 − 17.9) / 100 = 24.1% overdiagnosed					

*CCT* Clinical controlled trial, *CI* Confidence interval, *RCT* Randomized controlled trial, *MD* Mean difference, *NA* Not applicable, *y* years

^a^Risk of bias; ^b^inconsistency; ^c^indirectness; ^d^imprecision

^e^ Study data refers to the median control events rates across trials, which is the main analysis. A sensitivity analysis used the effects without screening for the general risk population in Canada, estimated from PRIOR et al. (Bone. 2015;71:237-43) based on 10-year follow-up

^f^ When our assessment of the certainty of evidence fell between levels, we assigned the level that best represented our actual certainty

^g^ Selected population defined as those who completed the initial risk assessment tool (as part of 2-step screening). This population may be more accepting of screening and have higher compliance than the general (intention-to-screen) population

^h^ This analysis included 1379 men from Kern 2005, representing 5.4% of the total sample

Among a *selected population* of females aged ≥65 years who are willing to independently complete a mailed fracture risk questionnaire, 2-step screening with risk assessment (clinical FRAX or FRAX-like tool) and BMD probably reduces the risk of hip fractures (3 RCTs + 1 CCT; *n*=43,736; 6.2 fewer in 1000, 95% confidence interval [CI] 9.0 fewer to 2.8 fewer; NNS=161) [4–6, 68] and clinical fragility fractures (3 RCTs; *n*=42,009; 5.9 fewer in 1000, 95% CI 10.9 fewer to 0.8 fewer; NNS=169) [4–6]. However, screening in this *selected population* probably does not reduce the risk of all-cause mortality (note: 1379 males were included in this analysis from the Kern CCT, representing 5.4% of the total sample) [4, 6, 68]. Our sensitivity analyses using assumed/baseline risks from a general Canadian population (age roughly corresponding to that of the trials) suggest that the effects for clinical fragility fracture may be larger than found in the trial populations, but these analyses are considered exploratory (Table 5). Post hoc subgroup analyses from the SCOOP study showed that the effectiveness of screening on hip fracture risk was greater in females with higher baseline clinical FRAX 10-year hip fracture risk (HR [95% CI] 0.67 [0.53–0.84] in the 90th percentile of risk vs. 0.93 [0.71–1.23] in the 10th percentile, *p*=0.021) and with prior fracture (HR [95% CI] 0.55 [0.38–0.79] vs. 0.87 [0.68–1.12], *p*=0.040 without prior fracture) [91]. The evidence for the effect of an offer of screening in a *selected population* of males is very uncertain [68]. In females aged 70–85 years, screening may make little-to-no difference in health-related quality of life [6]. Between 11.8% [6] and 19.3% [4] of females in a *selected population* offered 2-step screening may be overdiagnosed, but the magnitude of these estimates is of low certainty due to serious concerns of indirectness from lack of data provided as required for the proposed equation (e.g., mean risk in the high-risk population in SCOOP was limited to results of clinical FRAX without incorporation of BMD as used for treatment decisions) and from use of data from the SALT trial where participants were all at increased risk. Among females aged 70–85 years who are considered at high-risk by FRAX 10-year hip fracture risk alone and are referred to BMD assessment, data from one trial indicate that 24.1% may be overdiagnosed [6], but there is low certainty about this due to serious concerns about indirectness.

The evidence for hip and clinical fragility fractures among females aged 45–54 who accept 1-step screening with BMD measurement is very uncertain.

### KQ1b: Does the effectiveness of screening to prevent fragility fractures vary by screening program type (i.e., 1-step vs. 2-step) or risk assessment tool?

#### Study characteristics

As indicated in the findings for KQ1a, one RCT (OPRA) [93] was included for the comparative effectiveness of different screening approaches. Characteristics of the OPRA trial are in Table 3. The trial included a mailed *offer-to-screen* population (acceptors of screening also available but less relevant to the primary care population). Eligible (*n*=9268; 34% participated) postmenopausal females were randomized to one of three screening approaches: 1-step screening using BMD via DXA; 2-step screening using the Simple Calculated Osteoporosis Risk Estimation (SCORE)-based tool, with BMD assessment offered when the score was ≥7 (74% eligible); and 2-step screening using the Study of Osteoporotic Fractures (SOF)-based tool, with BMD assessment offered to those with ≥5 clinical risk factors (7% eligible) [93]. Patients were eligible for potential treatment if they had ≥5 risk factors and/or BMD *T*-score below age-specific thresholds, or if they had a prior fracture after age 50 years (SOF-based group only) [93]. The proportion of patients dispensed a prescription (including alendronate, hormone replacement therapy, calcitonin, raloxifene) was similar across groups (13 to 14% of those offered screening) [93]. The two outcomes reported by the trial were the total number of hip fractures, and clinical fragility fractures (reported as non-pathologic [osteoporotic] fractures) [93].

The risk of bias assessment for the OPRA trial is in Table 4. The trial was rated at unclear risk of bias due to the potential for selection bias (randomization and allocation concealment not clearly defined) and patient awareness of group assignment (those in the SCORE- and SOF-based groups not assigned to BMD testing would have increased awareness of risk and could seek further care) [93]. The trial was not powered to detect a difference in fracture outcomes across groups.

#### Findings

Additional file 3 contains the full analysis details for KQ1b, including the GRADE Summary of Findings Tables, with explanations for each rating and forest plots. The evidence from a single RCT showed that, among females aged 60–80 years, the evidence comparing 1-step (BMD) versus 2-step screening (risk assessment + BMD) and comparing different 2-step screening strategies (i.e., SCORE-based vs. SOF-based for the risk assessment) for risk of hip and clinical fragility fractures is very uncertain [93].

### KQ2: How accurate are screening tests at predicting fractures among adults ≥40 years?

Of 6081 unique records retrieved by the searches for KQ2, we assessed 413 for eligibility by full text, and 59 external validation cohort studies [96–154] taking place in very high human development index countries with moderate fracture risk, met eligibility criteria for inclusion in the review (Fig. 1). From our search update in June 2021 when we changed our eligibility to Canadian reports of unique cohorts or that added data to that previously included, we included one study [154] and excluded 18 other reports [148, 155–171]. Studies excluded after full text appraisal are listed with reasons in Additional file 5. Among the initial set of included studies from our search in July 2019, there were several that analyzed cohorts with substantial overlap in participants. To prevent double-counting in the analysis, when cohorts were overlapping for a given tool-outcome comparison, we selected a single primary cohort study for analysis (*n*=32) [98–100, 104, 106–113, 116, 117, 119, 128, 129, 134, 136, 138, 140, 142–146, 148–151, 153, 154]. We primarily considered recency in our choice of cohorts, but also considered the size of cohorts, quality of the methods (primarily more available data on predictors), and available outcomes. The remaining publications were then used for any reported supplementary data (e.g., calibration plots, subgroups of interest).

#### Study characteristics

Additional file 6 shows the characteristics of the included studies and their associated publications. Half (16/32, 50%) of the included studies were composed of participants from the USA (*n*=9) [104, 109, 110, 113, 136, 140, 143, 144, 153] and Canada (*n*=7) [106, 111, 119, 128, 129, 134, 154]; the remaining studies took place in Spain (*n*=4) [98, 99, 145, 150], Japan (*n*=3) [117, 148, 149], France (*n*=2) [146, 151], Israel (*n*=2) [108, 112], Poland (*n*=2) [107, 142], Australia (*n*=1) [116], New Zealand (*n*=1) [100], and Portugal (*n*=1) [138]. The studies analyzed data from a total of 1,491,968 participants (median 3305, range 91 to 1,054,815), with mean age ranging from 51 to 74.2 years. In more than half of the studies, only females were included (17/32, 53%) [98–100, 104, 106, 107, 112, 134, 136, 142–144, 146, 148, 150, 151]; the remaining were equally split between including only males (*n*=7, 22%; one cohort [129] included females but only the male population was used for analysis) [109, 110, 113, 116, 117, 153], and a mix of males and females (*n*=8, 25%) [108, 111, 119, 128, 138, 140, 145, 154]. Participants were often recruited from patient, insurance, or resident (e.g., electoral rolls) registries (*n*=16/32, 50%) [98, 108–113, 116, 119, 138, 140, 142, 143, 146, 148, 149]; ten (31%) studies enrolled all those presenting for BMD assessment (potentially at higher risk depending on local practices) [99, 106, 107, 128, 129, 136, 144, 145, 150, 151], five (16%) included patients already enrolled in other studies [100, 104, 117, 134, 154], and one (3.2%) enrolled only veterans [153]. Studies most commonly provided findings for the calibration of clinical FRAX (i.e., without incorporation of BMD) or with incorporation of BMD results (i.e., FRAX + BMD; *n*=26/32, 81%) [98–100, 104, 106–109, 111, 112, 116, 117, 129, 134, 138, 140, 142–144, 146, 148–151, 153, 154] and Garvan with or without BMD (*n*=8, 25%) [100, 104, 108, 113, 119, 142, 145, 154]; there were few external validation studies reporting on QFracture (*n*=3) [108, 113, 154], the Fracture Risk Calculator (FRC; *n*=2) [110, 136], CAROC (*n*=1) [128], and the Fracture and Immobilization Score (FRISC; *n*=1) [149].

The risk of bias ratings for the included studies for KQ2 are in Additional file 7. Almost all of the studies were at high overall risk of bias; only four [106, 111, 128, 129] were lacking serious risk of bias concerns (rated at unclear risk of bias because proxy variables were used for some predictors, e.g., chronic obstructive pulmonary disease instead of smoking status). The primary risk of bias concerns across the included studies were related to predictor ascertainment (missing predictor data, predictors not handled as intended by the tool), outcome ascertainment (self-reported or including high trauma fractures), and the analysis (large losses to follow-up and/or competing risk of mortality not accounted for, inadequate number [<100] of fracture outcomes, follow-up duration not matching the prediction period [e.g., substantially shorter or longer than 10 years without adjustment]). Many studies did not account for the effect of treatment prior to risk assessment or during follow-up.

#### Findings

Additional file 4 contains the full analysis details for KQ2, including GRADE Summary of Findings Tables, with explanations for each rating, and forest plots. Within the Summary of Findings Tables, discrimination findings from the USPSTF’s review are shown. Due to a high degree of heterogeneity that could not be well explained by a priori subgroup analyses, we generally did not pool data on calibration, and instead present the findings descriptively. The exception was FRAX-Canada, where we pooled (and relied on primarily) data from the three Canadian studies without serious risk of bias concerns. This decision was based on recognition that FRAX is considered as a suite of tools (algorithm calibrated to various countries) rather than a single tool; therefore, these Canadian studies without serious risk of bias would provide the most directly applicable evidence.

**Fig. 2:**
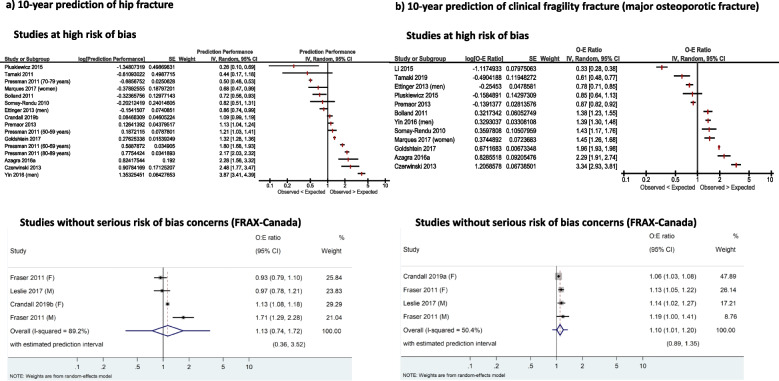
Calibration of clinical FRAX for the 10-year prediction of hip and clinical fragility fractures. Legend: Forest plots show the calibration ratios reported across the included studies; these were not pooled for the high risk of bias studies, and pooled for the studies without high risk of bias (reporting on FRAX-Canada)

**Fig. 3:**
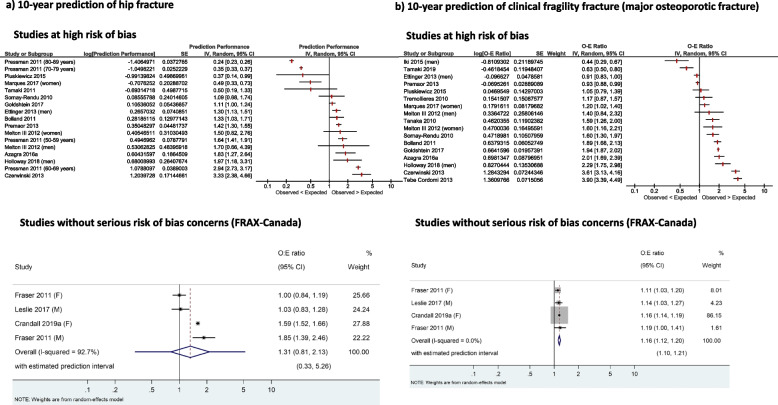
Calibration of FRAX with the incorporation of bone mineral density for the 10-year prediction of hip and clinical fragility fractures. Legend: Forest plots show the calibration ratios reported across the included studies; these were not pooled for the high risk of bias studies, and pooled for the studies without high risk of bias (reporting on FRAX-Canada)

Forest plots for the calibration of clinical FRAX and FRAX + BMD across studies with and without serious risk of bias concerns are in Figs. 2 and 3, respectively. For both the 10-year prediction of hip and clinical fragility fractures, there was a high degree of heterogeneity in O:E estimates across studies that was not well explained by subgroup analyses by age, sex, and baseline risk (Additional file 4). Most studies were at high risk of bias and did not use FRAX-Canada. We judged the performance of FRAX (with and without BMD) to be poor in these studies, but the evidence was rated as very uncertain due to concerns across all GRADE domains. Pooled data from three Canadian studies (*n* = 67,611) [106, 111, 129] without serious risk of bias indicate that clinical FRAX-Canada may be well calibrated for the 10-year prediction of hip fractures (O:E = 1.13, 95% CI 0.74–1.72, *I*^2^ = 89.2%) and is probably well calibrated for the 10-year prediction of clinical fragility fractures (O:E = 1.10, 95% CI 1.01–1.20, *I*^2^ = 50.4%), both with some underestimation of the observed risk. Data from these same studies (*n* = 61,156) [106, 111, 129] showed that FRAX-Canada with BMD may perform poorly to estimate 10-year hip fracture risk (O:E = 1.31, 95% CI 0.91–2.13, *I*^2^ = 92.7%), but is probably well calibrated for the 10-year prediction of clinical fragility fractures, with some underestimation of the observed risk (O:E 1.16, 95% CI 1.12–1.20, *I*^2^ = 0%). Within-study data from calibration plots (e.g., using deciles of baseline risk) were heterogeneous (7 studies for 10-year prediction of hip fractures [99, 100, 104, 109, 112, 143, 148] and 8 for clinical fragility fractures [99, 100, 104, 109, 112, 134, 143, 148] with clinical FRAX; 8 studies for the 10-year prediction of hip fractures [99, 100, 106, 109, 111, 125, 143, 148] and 10 for clinical fractures [99, 100, 106, 109, 111, 117, 140, 143, 148, 150] with FRAX + BMD), but two Canadian studies without serious concerns for risk of bias showed acceptable calibration of clinical FRAX-Canada in females at a baseline predicted risk above 5% [106], and FRAX-Canada with BMD in females at a baseline predicted risk above 6 or 12%, depending on the study [106, 111].

There is evidence to suggest acceptable calibration of FRAX to predict the 5-year risk of hip (FRAX + BMD only) and clinical fragility fractures (clinical FRAX and FRAX + BMD) (low certainty; most applicable to females) [129], but the prediction of 5-year risk is not a well-accepted or intended purpose of the tool. Findings on discrimination from Viswanathan 2018 [60] show an area under the curve (AUC) for the 10-year prediction of hip fractures in females of 0.76 (95% CI 0.72–0.81) for clinical FRAX and 0.79 (95% CI 0.76–0.81) for FRAX + BMD. The AUC for clinical fragility fractures in females was 0.67 (95% CI 0.65–0.68) for clinical FRAX and 0.70 (0.68–0.71) for FRAX + BMD [60]. Reported findings for males are in Additional file 4.

We are very uncertain about the ability of clinical Garvan (2 cohort; *n*=67,923) [104, 113] and Garvan + BMD (5 cohort; *n*=11,869) [100, 113, 119, 142, 145] to predict the 10-year risk of hip fractures and the 10-year risk of clinical fragility fractures [100, 113, 119, 142, 145]. Clinical Garvan (1 cohort; 1,054,815) [108] may underestimate the 5-year risk of hip fractures (O:E 2.17, 95% CI 2.16 to 2.17; low certainty); evidence for calibration for 5-year risk of clinical fragility fractures is very uncertain [154]. The AUC for 10-year prediction of hip fractures reported by the USPSTF was 0.68 (95% CI not reported) for clinical Garvan and 0.73 for Garvan + BMD [60]. For clinical fragility fractures in females, the AUC was 0.66 (95% CI 0.61–0.72) for clinical Garvan and 0.68 for Garvan + BMD [60]. Data for males are in Additional file 4. There is evidence from one study (*n*=34,060) to suggest that CAROC [128] (includes BMD) may be adequately calibrated to predict a category of 10-year risk of clinical fragility fracture; observed fracture risk (95% CI) was 6.4 (6.0–6.8)% in the low risk (<10%) group, 13.8 (13.1–14.5)% in the moderate risk group (10–20%), and 23.8 (22.5–25.0)% in the high-risk group (>20%). The discrimination of this tool was not reported by the USPSTF [60]. There was very limited evidence for the remaining tools (QFracture [108, 113, 154], FRISC [149], FRC [110, 136] with and without BMD).

### KQ3a: What are the benefits of pharmacologic treatments to prevent fragility fractures among adults ≥40 years?

Of 11,693 unique records retrieved by the searches for KQ3a, we assessed 211 for eligibility by full text and included 27 RCTs [172–198] (one trial of alendronate was open-label [185]) and 11 associated publications [199–209] (Fig. 1). Studies excluded after full text appraisal are listed with reasons in Additional file 5.

#### Study characteristics

Detailed study characteristics are in Additional file 6. In total, there were 10 trials of alendronate (5 or 10 mg/day, or mixed doses, or 70 mg/week for 12 to 48 months) [172, 173, 176, 177, 183–185, 187, 193, 197], 7 trials of risedronate (2.5 or 5 mg/day for 12 to 36 months) [179, 182, 183, 186, 189, 190, 196], 6 trials of zoledronic acid (1 to 5 mg/year [5 mg/year most commonly] for 12 to 72 months) [175, 180, 181, 188, 194, 195], and 6 trials of denosumab (60 mg/6 months, or mixed doses for 12 to 36 months) [174, 178, 185, 191, 192, 198]. About half (14/27, 52%) of the trials were multi-country [172, 175, 178, 179, 183, 184, 187–191, 193, 194, 196], with the remaining taking place in the USA (*n*=4) [173, 176, 177, 185], New Zealand (*n*=3) [180, 181, 195], China (*n*=3) [186, 197, 198], Australia (*n*=1) [182], India (*n*=1) [192], or the USA and Canada (*n*=1) [174].

The trials included a total of 34,317 participants (median 398, range 50 to 9931), primarily postmenopausal females with low BMD (definition variable across trials). The prevalence of prior fracture was median 16.9% (range 0 to 48%) when specified in the trials. There were only two trials of males with low BMD, one for zoledronic acid [175] and one for denosumab [191]. Most of the trials were small and probably underpowered to detect differences in fracture incidence, especially for hip fractures; analyses generally relied on one large trial per drug. Most (23/27, 82%) trials included adjunct calcium and/or vitamin D supplements in both groups (treatment and placebo). Length of follow-up for outcomes ranged from 0.5 to 6 years, which in almost all cases corresponded with the duration of treatment; rarely, the follow-up period extended 1 year beyond the end of treatment. The trials provided data for hip fractures [172, 175–178, 180, 181, 184, 186, 187, 189–193, 195–198], clinical fragility fractures [172–175, 177–196, 198], clinical vertebral fractures [172, 174, 176–178, 180, 181, 184, 186, 191, 192, 194–197], all-cause mortality [174–178, 180, 185, 188, 191, 192, 195–198], and health-related quality of life [178]; no trials reported on fracture-related mortality or functionality and disability. Discontinuation due to adverse events, serious and non-serious adverse events are addressed in KQ3b.

The risk of bias ratings for the trials included for KQ3a are in Additional file 7. One of the main risk of bias concerns was selective reporting, as many trials lacked protocols and did not pre-specify fractures as an outcome of interest (either in a protocol or in the “Methods” section); instead, these were often collected as potential harms. In these cases, it was often unclear whether the fracture outcomes were collected prospectively or systematically [172, 173, 176, 180, 181, 185, 190–192, 197, 198]. Several trials were at high risk of attrition bias, due to large or imbalanced losses to follow-up for various outcomes [172, 173, 179, 180, 186, 189–191]. One trial of alendronate was open-label [185] and therefore was at high risk of performance and detection biases. When applicable (“all bisphosphonates” analyses), we assessed for small study bias and this was not detected.

#### Findings

Additional file 4 contains the full analysis details for KQ3a, including GRADE Evidence Profiles and Summary of Findings Tables, with explanations for each rating and forest plots.

##### Bisphosphonates

In postmenopausal females at risk of fragility fractures, the risk of hip fractures may be reduced by median 2 (range 1 to 6) years of treatment with bisphosphonates as a class (alendronate, risedronate, or zoledronic acid; 14 RCTs; *n*=21,038; 2.9 fewer in 1000, 95% CI 4.6 fewer to 0.9 fewer; NNT=345; low certainty) compared to placebo [48, 172, 176, 177, 180, 181, 184, 186, 187, 189, 190, 193, 195–197, 201, 209]. Data for individual bisphosphonates showed that median 3 (range 1 to 3) years of treatment with risedronate may reduce the risk of hip fractures (4 RCTs; *n*=9,672; 7.9 fewer in 1000, 95% CI 13.0 fewer to 1.5 fewer; NNT=127; low certainty), but median 2 (range 1 to 4) years of treatment with alendronate and median 2 (range 2 to 6) years of treatment with zoledronic acid may not reduce the risk of hip fractures (low certainty). Within-study subgroup analyses were available for alendronate [177] and risedronate [189] (1 trial each) by age and baseline risk (BMD, prevalent fractures). These were not considered to be credible as they were available only in single trials (no evidence of consistency), may not have been adequately powered, and were not necessarily pre-specified (Additional file 4). One trial in males (*n* = 1199) showed that 2 years of treatment with zoledronic acid may not reduce the risk of hip fractures [175].

The risk of clinical fragility fractures in postmenopausal females is probably reduced by median 2 (range 1 to 6) years of treatment with bisphosphonates as a class (19 RCTs; *n*=22,482; 11.1 fewer in 1000, 95% CI 15.0 fewer to 6.6 fewer; NNT=90; moderate certainty) [172, 173, 177, 179–184, 186–190, 193–196, 200, 201, 203, 206, 209], median 2 (range 1 to 4) years of treatment with alendronate (8 RCTs; *n*=8854; 14.7 fewer in 1000, 95% CI 24.5 fewer to 2.6 fewer; NNT=68; moderate certainty) [172, 173, 177, 183, 184, 187, 193, 200, 203, 206, 209] and median 2 (range 1 to 6) years of treatment with zoledronic acid (5 RCTs; *n*=3,218; 20.1 fewer in 1000, 95% CI 27.6 fewer to 9.9 fewer; NNT=50; moderate certainty) compared to placebo [180, 181, 188, 194, 195, 201]. Median 2 (range 1 to 3) years of treatment with risedronate may reduce the risk of clinical fragility fractures (7 RCTs; *n*=10,572; 7.8 fewer in 1000, 95% CI 12.5 fewer to 2.3 fewer; NNT=128; low certainty) [179, 182, 183, 186, 189, 190, 196]. The analyses were robust to sensitivity analysis using only “nonvertebral fractures” in one trial of zoledronic acid where nonvertebral and vertebral fractures had been summed to determine the total number of people with fractures (could overestimate) [195]. One trial in males (*n* = 1199) showed that 2 years of treatment with zoledronic acid may not reduce the risk of clinical fragility fractures [175].

The risk of clinical vertebral fractures among postmenopausal females may be reduced by median 2 (range 1 to 6) years of treatment with bisphosphonates as a class (11 RCTs; *n*=8921; 10.0 fewer in 1000, 95% CI 14.0 fewer to 3.9 fewer; NNT=100; low certainty) [172, 176, 177, 179–181, 184, 194–197, 201, 203] and median 2 (range 1 to 6) years of treatment with zoledronic acid (4 RCTs; *n*=2367; 18.7 fewer in 1000, 95% CI 25.6 fewer to 6.6 fewer; NNT=53; low certainty) [180, 181, 194, 195]. The evidence for alendronate [172, 176, 177, 184, 197, 203] and risedronate [179, 196] is very uncertain. There were no studies in males that reported on clinical vertebral fractures.

Bisphosphonates as a class may not reduce the risk of all-cause mortality in postmenopausal females compared to placebo over 1 to 6 years of follow-up [176, 177, 180, 185, 188, 195–197, 202, 206]. Evidence for individual bisphosphonates is very uncertain (including for zoledronic acid in males).

##### Denosumab

In postmenopausal females the risk of hip fractures may not be reduced by median 1 (range 0.5 to 3) years of treatment with denosumab compared to placebo [178, 192, 198, 199, 207]. Within-study subgroup analyses were available by age, baseline BMD and FRAX score from one trial [178], but were not considered credible because there is no evidence that the effects are consistent as they have not been replicated in other trials (Additional file 4). The risk of clinical fragility fractures is probably reduced by median 1.5 (range 0.5 to 3) years of treatment with denosumab (6 RCTs; *n*=9473; 9.1 fewer in 1000, 95% CI 12.1 fewer to 5.6 fewer; NNT=110; moderate certainty) [174, 178, 185, 192, 198, 206, 207]. This analysis was robust to sensitivity analysis using only “nonvertebral” fractures for one trial [178] where vertebral and nonvertebral were summed to determine the total number of people with fractures. The risk of clinical vertebral fractures is probably reduced by median 1.5 (range 0.5 to 3) years of treatment with denosumab (4 RCTs; *n*=8639; 16.0 fewer in 1000, 95% CI 18.6 fewer to 12.1 fewer; NNT=62; moderate certainty) [174, 178, 192, 204, 205]. Denosumab probably does not reduce the risk of all-cause mortality over 0.5 to 3 years of follow-up [174, 178, 185, 192, 198, 205–207], and probably makes little-to-no difference in health-related quality of life over 3 years of follow-up [208]. The evidence for the effect of denosumab on the incidence of fractures (hip, clinical fragility, clinical vertebral) and all-cause mortality from one trial in males (*n*=242) [191] is very uncertain.

### KQ3b: What are the harms of pharmacologic treatments to prevent fragility fractures among adults ≥40 years?

Of 721 unique records retrieved by the searches for KQ3b, we assessed 85 for eligibility by full text with 31 systematic reviews and one primary study meeting our eligibility criteria (Fig. 1). After reviewing these for key characteristics, we included 10 systematic reviews [60, 210–218], 3 associated publications [37, 48, 49], and one primary study on rebound fractures after discontinuation of denosumab [219]. Reviews excluded after full text appraisal, as well as systematic reviews that met inclusion criteria but were not selected for the overview, are listed with reasons in Additional file 5.

#### Study characteristics

Characteristics of the systematic reviews and primary study are in Additional file 6. The systematic reviews were published between 2014 and 2020 and included either only RCTs [212, 216, 217] or a mix of RCTs and observational studies [60, 211, 213–215, 218]; occasionally, only observational studies were included when there existed no RCTs for rare harms [210]. The systematic reviews were generally focused on patients (males or females) with low BMD (often referred to as osteoporosis) or who had risk factors for fracture, though some included wider populations (e.g., patients with chronic use of glucocorticoids); in many cases, patients with other disorders of bone metabolism were excluded. Across the systematic reviews, risk of bias was usually not assessed specific to harm outcomes (assessed in 3 reviews [210, 216, 217]), and certainty of evidence was assessed for selected outcomes in only three of the systematic reviews [60, 211, 215]. Notably, no evidence (either no systematic reviews, or the systematic reviews located no primary studies) was located for the following outcome comparisons: serious stroke and thromboembolic events, atypical femoral fractures, osteonecrosis of the jaw, or myalgia, cramps, and limb pain with risedronate; serious gastrointestinal adverse events, gastrointestinal cancer, pulmonary embolism, and thromboembolic events with zoledronic acid; osteonecrosis of jaw with long-term bisphosphonates as a class; serious gastrointestinal adverse events, gastrointestinal cancer, thromboembolic events, cardiac death, and rebound hip fractures with denosumab. The primary study on rebound fractures (multiple vertebral fractures) after discontinuation versus persistence of denosumab was a retrospective cohort study of 3110 individuals (91% females; mean age 72 years; 42% with prior fracture; denosumab as first-line therapy for 5.4%) conducted in Israel.

The appraisal of the quality of the systematic reviews and primary study included for KQ3b are shown in Additional file 7. Common methodological concerns across the reviews were potential errors in data extraction (because data were not collected in duplicate), limited description of the characteristics of the included studies, and lack of risk of bias appraisal (or risk of bias was assessed for benefits but not for harms). The primary study did not adjust findings for potential confounders, though there was demonstration of comparability across multiple characteristics between groups.

#### Findings

Additional file 4 contains the full analysis details for KQ3b, including GRADE Evidence Profiles and Summary of Findings Tables, with explanations for each rating.

##### Bisphosphonates

The evidence was very uncertain for many adverse events, for example gastrointestinal cancers and several of the serious cardiovascular events. Compared to no treatment, alendronate may increase the risk of atypical subtrochanteric (0.08 more in 1000, 95% CI 0.05 more to 0.14 more; systematic review of 1 cohort; *n*=220,360; NNH=12,500; low certainty) [215] and femoral shaft fractures (0.06 more in 1000, 95% CI 0.03 to 0.10; systematic review of 1 cohort; *n*=220,360; NNH=16,667; low certainty) [215], and osteonecrosis of the jaw (systematic review of 1 cohort; *n*=220,360; 0.22 more in 1000, 95% CI 0.04 more to 0.59 more; NNH=4545; low certainty) [215]. The evidence for bisphosphonates as a class showed similar findings [48, 49, 211, 215]. The risk of “any serious adverse event” (composite outcome) is probably not increased with risedronate [37, 60] and zoledronic acid [37, 60] and may not be increased with alendronate [37, 60]. The risk of certain serious gastrointestinal adverse events (perforations, ulcers, and bleeds; serious esophageal) may not be increased with alendronate [48, 49, 211]. The risk of stroke and myocardial infarction probably does not increase with bisphosphonates as a class [216]; certainty was low for little-to-no difference in other serious cardiovascular events from individual drugs and from the drug class [48, 49, 211, 216].

The risk of non-serious gastrointestinal adverse events is probably increased by treatment with alendronate (systematic review of 50 RCTs; *n*=22,549; 16.3 more in 1000, 95% CI 2.4 more to 31.3 more; NNH=61; moderate certainty) [48, 49, 211], but probably not by treatment with risedronate [48, 49, 211]. Non-serious adverse events (composite outcome) are probably increased by treatment with zoledronic acid (systematic review of 6 RCTs; *n*=9575; 51.8 more in 1000, 95% CI no difference to 112.2 more; NNH=19; moderate certainty) [212], related to the potential increased risk of multiple influenza-like symptoms [48, 49, 211] including pyrexia [212], headache [212], chills [48, 49, 211], arthritis and arthralgia [48, 49, 211], and myalgia [48, 49, 211] (low-to-moderate certainty). With the exception of zoledronic acid, the risk of “any non-serious adverse event” (composite outcome) [212] and discontinuation due to adverse events [37, 60] do not appear to be increased by treatment with bisphosphonates (low-to-moderate certainty).

##### Denosumab

The evidence was very uncertain for many adverse events, including serious infections [37, 60], venous thromboembolism [213], and rebound fractures after denosumab discontinuation [219]. Treatment with denosumab may not increase the risk of “any serious adverse event” (composite outcome) [37, 60] and does not appear to increase the risk of serious cardiovascular outcomes (stroke and various composite outcomes) [48, 49, 211, 213, 217] (low certainty).

The risks of non-serious gastrointestinal adverse events (systematic review of 3 RCTs; *n*=8454; 64.5 more in 1000, 95% CI 26.4 more to 113.3 more; NNH=16; moderate certainty) [48, 49, 211], rash or eczema (systematic review of 3 RCTs; *n*=8454; 15.8 more in 1000, 95% CI 7.6 more to 27.0 more; NNH=63; moderate certainty) [37, 60], and infections (any serious or non-serious; systematic review of 4 RCTs; *n*=8691; 1.8 more per 1000, 95% CI 0.1 more to 4.0 more; NNH=556; moderate certainty) [48, 49, 211] are probably increased by treatment with denosumab. Risks of any non-serious adverse event (composite outcome) [212, 213] and discontinuation due to adverse events [37, 60] do not appear to be increased by treatment with denosumab (moderate and low certainty, respectively).

### KQ4: For adults ≥40 years, what is the acceptability of screening and/or initiating treatment to prevent fragility fractures when considering the possible benefits and harms from screening and/or treatment?

Of 8794 unique records retrieved by the searches for KQ4, we assessed 146 for eligibility by full text and included 12 studies (5 cross-sectional [220–224], 4 cohort [225–228], 3 RCTs [229–231]) and one associated publication of another study [53] (Fig. 1). Studies excluded after full text appraisal are listed with reasons in Additional file 5.

#### Study characteristics

Detailed study characteristics are in Additional file 6. Half of the 12 studies were conducted in the USA (6/12, 50%) [221, 223, 225–227, 231]; the remaining were conducted in New Zealand (*n*=3) [222, 229, 230], Canada (*n*=1), the Netherlands (*n*=1) [220], and China (*n*=1) [224]. Across all studies, a total of 2188 participants (median 204, range 30 to 393) were included, primarily postmenopausal females. In three studies [222, 224, 230], both males and females were included. One study reported on the acceptability of screening among females who would be considered to be at low risk based on age (mean 57 years, range 50 to 65 years) [231]. The remaining 11 studies elicited patients’ views on the acceptability of initiating pharmacologic treatments. In four (36%) of these studies, patients who were at risk for fracture based on BMD (*T*-score in osteoporosis or osteopenia range, definitions varied across studies) and were aware of their 10-year major osteoporotic and/or hip fracture probability were provided decision aids and were in the position to make real-life decisions about starting treatment. In the remaining studies, the decisions about starting treatment were based on hypothetical scenarios; patients in these studies were not always made aware of their fracture risk and would not necessarily have been eligible for treatment [220–224, 229–231].

The risk of bias assessments for studies included in KQ4 are in Additional file 7. Four studies were at high risk of bias due to low participation rates (<40% of those eligible) [222, 223, 229, 231]. Three studies were at high risk of bias because they provided participants no or inaccurate (based on our comparison to currently available evidence) information on the potential benefits or harms of treatment—we required information on at least one of benefits or harms for inclusion [222, 227, 230]. Two studies were considered to be at high risk of bias because they did not present findings for important subgroups of interest (e.g., baseline fracture risk) for whom results may be expected to differ [225, 227]. Other risk of bias concerns were infrequent.

#### Findings

Additional file 4 shows the full analysis details for KQ4, including GRADE Summary of Findings Tables, with explanations for each rating. One RCT (*n*=258) [231] that included females aged 50–65 years (low risk based on age), revealed that this population had a strong intention to be screened over the next 5 years (mean [standard deviation] intention score 3.74 [0.96]/5). Participants were then provided a 1-page decision support sheet containing information on benefits in one of four formats (words, numbers, narrative, or framed narrative in terms of benefits of *not* screening). The sheet indicated that screening and treatment would be associated with a reduction in the risk of hip fractures by 2 per 1000 or “very few” females, and a reduction in other fractures in “few” females over 10 years. Risks were described as the potential for worry, minor stomach upset, and muscle or joint pain. Serious harms were described as rare—osteonecrosis of the jaw in 1 to 10 per 1000 or “very few” females and atypical fractures in 5 per 1000 or “very few” females over 10 years. Overdiagnosis was presented by showing that the incidence of low bone density (labelled as osteoporosis) exceeded important fracture outcomes. After reviewing the decision support sheet, participants’ intention to screen did not change substantially and also did not differ based on the format of information provided (1 study, *n*=258; low certainty) [231].

Seven observational studies and two RCTs (*n*=1930; sample size uncertain in one study) [220, 221, 224–230] reported on the acceptability of treatment. In five studies (*n*=1010) [220, 221, 224, 229, 230], adults (primarily females) ≥50 years old were provided information on the benefits and harms of treatment in various formats; not all participants in these studies were considered to be at high fracture risk or eligible for treatment. In these studies, patients were asked to make hypothetical treatment decisions, with results of three studies showing that patients’ preference for treatment versus no treatment may be highly variable (3 studies, *n*=317; low certainty) [220, 221, 224]. Two other studies showed that after receiving information on their personal fracture risk (median [IQR] 10-year hip fracture risk 2.2 [0.5–2.7%] in one study, 5-year hip fracture risk 1.4 [0.8–3.0%] in the other), relatively few (19 to 39%) patients may be willing to accept treatment (2 studies, *n*=593; low certainty) [229, 230]. In the four remaining studies (*n*=324; sample size uncertain in one study), postmenopausal females with low bone density (labelled as osteoporosis or osteopenia) who were in the position to make real-life decisions about treatment were provided decision aids outlining the potential benefits and harms of treatment. These studies showed that few (5–20%) eligible patients who read decision aids and are aware of their fracture risk are willing to initiate treatment (2 studies, *n*=240; sample size uncertain in one study [227, 228], but that somewhat more may be willing to start treatment when the decision aid is used during a clinical encounter (4–44% acceptance; 2 studies, *n*=84 [225, 226] or when they have had a previous fracture or are at higher fracture risk (32–45%; 1 study, *n*=208) [53, 228]. Overall, a minority of postmenopausal females at increased risk for fracture may accept treatment (moderate certainty).

Three observational studies (*n*=741) [220, 222, 224] reported on the minimum acceptable benefit of treatment among adults ≥50 years (mean 60 to 72 years) provided hypothetical scenarios about the benefits and harms of anti-osteoporosis treatment. These studies indicated that about two-thirds (64%) of adults ≥50 years may have overly optimistic views of the benefits of treatment (1 study, *n*=354) [222] and that these views may be highly variable (3 studies, *n*=741; low certainty) [220, 222, 224]. For example, one study reported that patients may require a reduction of 20 to 200 fractures per 1000 to consider 10 years of bisphosphonate treatment with no major side effects to be acceptable (1 study, *n*=354; low certainty) [222].

Six observational studies (*n*=1091) [53, 220, 223, 226, 229, 230] reported on the level of risk at which treatment would be considered acceptable among adults (97% female) ≥45 years old who were aware of their personal fracture risk but not necessarily at high risk or making real-life treatment decisions. These studies reported that there is large heterogeneity in the level of risk at which treatment would be considered to be acceptable (6 studies, *n*=1091; low certainty) [53, 220, 223, 226, 229, 230]. Many patients (19 to 51%) are willing to accept treatment even at low levels of fracture risk (5 to 20%); meanwhile, a large proportion (44 to 68%) of high-risk females (≥3% hip or ≥20% osteoporotic fracture risk; ≥30% in one study) would choose not to be treated (3 studies, *n*=378; low certainty) [53, 226, 229].

## Discussion

### Summary of principal findings for screening

In this review, we found that among a *selected population* of females aged 65 years and older who are willing to independently complete a mailed questionnaire about personal risk factors, an offer of 2-step screening using a fracture risk assessment tool (clinical FRAX) followed by assessment of BMD in those at increased risk (and treatment initiated based on various criteria) probably reduces the risk of hip (6.2 fewer in 1000, NNS=161) [4–6, 68, 91] and clinical fragility fractures (5.9 fewer in 1000, NNS=169) [4–6, 91] over 3 to 5 years of follow-up. The evidence is very uncertain for younger females [90] and for males [68]. A mailed offer of screening to females aged 68 to 80 years, where 54% returned a completed questionnaire and were eligible, may not reduce the risk for hip or clinical fragility fractures over 5 years of follow-up [5]. Screening does not appear to make any difference in the risk of all-cause mortality nor wellbeing (very uncertain for younger females). The findings for the selected population (willing to independently complete clinical FRAX) are similar to those of a 2020 systematic review that pooled data only from the three most recent trials [7]. Minimal evidence related to the potential harms of screening is available; in one trial [6] no serious adverse events were reported but these did not appear to be collected systematically. Among *selected* females offered screening, 12% of those meeting age-specific treatment thresholds based on FRAX 10-year hip fracture risk, and 19% of those meeting thresholds based on FRAX 10-year major osteoporotic fracture risk, may be overdiagnosed according to our definition [4, 6, 59]. We did not locate convincing evidence to recommend one method of screening over another, although the evidence from the trials supports the use of clinical FRAX followed by BMD assessment in those at increased risk.

### Clinical considerations and implications

There appeared to be a considerable amount of ad hoc screening (and subsequent treatment; median 17%) in the control groups of the included trials; it is possible that the magnitude of effect would have been larger with a true “no screening” comparator. In all of the trials, the rate of completion of mailed risk assessment tools was low (generally less than two-thirds of those who were sent the tool), and 8 to 29% of those eligible for BMD did not attend [4–6]. There appeared to be a healthy selection bias in several of the trials. For example, in the SALT trial 25% of those who were offered DXA did not participate, and non-participants were among those at the highest fracture risk on clinical FRAX [4]. In the ROSE trial, the majority of fractures occurred in those who did not return the initial mailed risk assessment questionnaire [5]. In our review of the acceptability of screening, we similarly found that low risk (based on age) females have a high intention to be screened [231], but unfortunately we found no studies reporting on the intentions of higher-risk females. An analysis of non-participants in the ROSE trial showed that those who declined DXA scans were older, more likely to have comorbid conditions, had lower socioeconomic status, and were more likely to smoke and have high alcohol consumption [232]. Many of these factors may also place a person at increased risk for fracture. There are multiple reasons for which a person may choose not to be screened. For example, lack of interest may be related to a low perception (and perhaps underestimation) of personal fracture risk [232], the belief that low bone density is not a serious health issue [233], and fears of the potential serious harms of treatment despite their rare occurrence [234]. If screening for fracture risk is believed to be important, there may be a need to improve its accessibility for those at highest risk, and to attempt shared decision-making on the benefits and harms.

The mechanism by which the small reductions in fracture risk were achieved by screening is uncertain in light of other findings of this review. For example, among postmenopausal females, we found that treatment with bisphosphonates as a class may result in small reductions in the risk of hip (2.9 fewer in 1000; NNT=345) and clinical fragility fractures (11.1 fewer in 1000; NNT=90), of a magnitude similar to that seen in the screening trials, where only a small proportion of females were eligible for treatment and treated for a clinically meaningful length of time. In the screening context, we also observed an absolute risk reduction for hip fractures (6.2 per 1000) that was of similar magnitude to the reduction in clinical fragility fractures (5.9 per 1000) among females who independently completed the FRAX tool. The plausibility of this finding is difficult to ascertain. Notably, the one trial finding a statistically significant reduction in hip fracture risk with screening (SCOOP) did not find a similar reduction in the risk of clinical fractures [6], an outcome that occurs more frequently than hip fractures. It is possible that participants in this trial were better selected to benefit in terms of hip fracture reduction, because FRAX 10-year hip fracture risk was used in treatment thresholds, as opposed to 10-year major osteoporotic fracture risk used in the other trials. It is also possible that the treatments used in the trials were more effective at reducing hip rather than other clinical fractures, or simply that hip fractures were more reliably reported and ascertained than other fractures. Uncertainty remains because the trials do not provide information on which particular participants sustained fractures (i.e., those at increased risk or otherwise). Females in the screening trials may have been at higher risk overall than in the treatment trials due to older age (e.g., in SCOOP all were ≥70 years), though this is difficult to ascertain.

The effectiveness of screening may depend on uptake and persistence with anti-fracture treatments among those at high risk [50], but this tends to be suboptimal and declines with longer durations of treatment [51]. In the three more recent screening trials, uptake of anti-fracture drugs ranged from 69 to 80% of those with a treatment indication [4–6]; however, these values could be overestimates as they were based on self-reports and prescription records. Longer-term follow-up from the SALT trial showed that by 36 months less than half (43%) of those at high risk reported using anti-osteoporosis drugs [4]. In the larger treatment trials, full compliance with treatment was somewhat higher, ranging from about 50 to 80% [177, 178, 189, 195]. One hypothesis is that the benefits seen from screening might be the result of unmeasured variables. For example, participation in screening may have provoked alterations in health behavior that helped participants to avoid fractures [235], like increasing weight-bearing exercise, stopping smoking, or taking preventive action to reduce the risk of falls. Post hoc analyses from the SCOOP trial showed, however, that screening had no significant impact on the risk of falls [236], and that the intervention was most beneficial in those at highest baseline hip fracture risk and those with prior fracture [91]. These findings suggest that the reduction in fracture risk seen with screening may be more related to treatment uptake and adherence (even if suboptimal) than other risk-reducing behaviors. It remains unclear from the trials whether the patients who sustained fractures were those who undertook treatment. It should be noted that decreased fracture risk in our review was only seen among highly motivated participants (those completing the clinical FRAX independently or accepting screening with BMD) who are probably more likely to adhere to treatment than the general population. The recent screening RCTs focused on treatment using first-line pharmacologic treatment and it is unclear what the impact may have been, if any, if they replaced this with or added therapies including vitamin D and calcium and/or interventions designed to prevent falls (e.g., exercise) or fractures from falls (e.g., hip protectors).

### Predictive value of screening strategies

If screening, overall, is believed to offer net benefit, there is limited certainty about which strategy to use. Two-step with risk assessment followed by BMD in those meeting a pre-determined risk threshold appears effective for reducing fractures, and the variable screening methods and treatment criteria in the trials suggest that some variation between strategies may be acceptable. The evidence from one comparative effectiveness trial suggests that BMD alone may be more effective than 2-step screening but we rated this evidence to be of very low certainty. The trials are most applicable to use of clinical FRAX for risk assessment and FRAX with BMD for treatment thresholds, and the evidence from KQ2 indicates that FRAX-Canada (with or without BMD) is probably well calibrated, with some underestimation, for the 10-year prediction of clinical fragility fractures [106, 111, 129]. Clinical FRAX-Canada may also be well calibrated, with some underestimation, to predict the 10-year risk of hip fracture, but the calibration of FRAX + BMD for this outcome may be poor [106, 111, 129]. One potential reason for the underestimation is lack of ability to incorporate a history of previous falls in FRAX; clinicians should be aware that those with previous falls may be at higher risk than estimated with FRAX [237]. The CAROC tool seemed to be adequately calibrated to predict a category of risk; however, it was not used in any of the included trials and requires the inclusion of BMD results. It was beyond the scope of this review to compare screening tools directly (e.g., with vs. without BMD); however, the evidence from this review showed clinical FRAX-Canada to be adequately calibrated without the addition of BMD. A review by Kanis et al. showed high concordance between risk categorization using either FRAX scores or BMD alone; people with higher scores are also generally those with a low BMD [238]. Also of interest is that in one of the trials (SCOOP) [6], only about one-third of those considered at high risk for 10-year hip fracture with clinical FRAX (using criteria suggested for treatment initiation in some cases [239]) were eligible for treatment (using only slightly different criteria) after their BMD results were incorporated into the predictions. Though not a focus of the current review, it is important to consider that the calibration of FRAX may vary by ethnicity. In a study using data from the Manitoba Bone Mineral Density Program registry, FRAX-Canada substantially overestimated 10-year risk of fracture in females who identified as Black or Asian as compared to White [240].

### Treatment effects

We found that treatment of postmenopausal females in a primary prevention population (<50% with prior fracture, but who are at risk of fragility fracture) with bisphosphonates as a class probably reduces the risk of clinical fragility fractures. Notably, our conclusion for the effect of bisphosphonates on the risk of hip fractures differs from the USPSTF who in 2018 reported low certainty evidence of no benefit [37]. We included additional trials in our analysis (including one large trial of zoledronic acid published after the USPSTF’s review was completed) and found a similar estimate of effect as the USPSTF but with improved precision, allowing for us to conclude that bisphosphonates may reduce the risk of hip fracture. Denosumab probably reduces the risk of clinical fragility fractures and clinical vertebral fractures, but may not reduce the risk of hip fractures. The limited evidence showed that zoledronic acid may not reduce the risk of hip or clinical fragility fractures in males with low BMD, and evidence for the use of denosumab in males was very uncertain. As reported in a recent review of risedronate for primary and secondary prevention of fractures [241], the trials for individual drugs are hampered by lack of power, as most studies focused on the impact of treatment on BMD as their main outcome of interest, rather than fractures which are then reported only as adverse events. Selection into treatment studies was often based on BMD, and no study used clinical risk scores to select patients. Similar to the screening trials, participants with prior fracture were often included, which differs somewhat from primary prevention where screening would be aimed at those without prior fracture. This review’s focus was determining estimates for the effects from the treatments used as first-line therapy in the RCTs on screening (mostly from anticipation of poor reporting on the harms), which largely employed pharmacologic treatment. Nevertheless, considering that most hip fractures occur as a direct result of a fall [242], preventing falls may be of value for people at high risk for fracture. The Task Force is currently developing recommendations about interventions for preventing falls [54].

### Patient perspectives

Though pharmacologic treatments appear to be beneficial, the magnitude of benefit may not be felt to be important enough to make treatment acceptable to patients. The most important findings of our acceptability review were that despite a high willingness to be screened among younger females, a minority of eligible older females may be willing to undergo treatment. Additionally, there was a large degree of variability in the level of risk at which individual patients would be willing to accept treatment (given information on benefits and/or harms). Many older adults have unrealistic views about the effectiveness of treatment and may require a reduction of 20 to 200 fractures per 1000 to consider 10 years of treatment with a bisphosphonate with no major side effects; this is at least double the magnitude of reduction in risk that was observed in our meta-analyses. Overall, though it was outside the scope of our review to determine the optimal length of treatment, a recent systematic review by Fink et al. found evidence of moderate certainty for no difference in the risk of clinical fragility fracture with 5 versus 10 years of treatment with alendronate and 3 versus 6 years of zoledronic acid [215]. There appeared to be some benefit of longer (10 vs. 5 years) treatment with alendronate on the risk of clinical vertebral fractures [215].

### Consideration of treatment harms and shared decision-making

Patients considering treatment should be able to weigh the proposed benefits with potential harms. We found increased risk for some non-serious adverse events; namely non-serious gastrointestinal events with alendronate; influenza-like symptoms with zoledronic acid; and non-serious gastrointestinal adverse events, dermatologic adverse events, and infections with denosumab. There was also low certainty evidence for an increased risk for the rare occurrence of atypical femoral fractures and osteonecrosis of the jaw with bisphosphonates (most evidence for alendronate). A concern about the risk of rebound fractures, and in particular multiple vertebral fractures, after cessation of treatment with denosumab has been raised by clinical experts [218, 243]. This requires more research focus as to date there is only minimal empiric evidence of very low certainty addressing these concerns; this finding was based on one available trial that compared discontinuation of denosumab with discontinuation of placebo (FREEDOM and its extension) [178, 244]. In this study, findings from patients initially randomized to denosumab or placebo who participated in the extension were analyzed for the occurrence of fractures after voluntary discontinuation (i.e., non-random sample). Ideally, trials would follow randomized participants from treatment initiation through an adequate time period after discontinuation to fully understand the net impact of denosumab treatment and subsequent discontinuation on the risk of fractures. The findings of our review also substantiate the large heterogeneity in the level of risk at which patients may accept treatment [52]. The finding that patients’ decisions about treatment may not correspond with guideline-recommended treatment thresholds [53, 225–227], and awareness of the complexity of decisions about treatment [245], supports the importance of shared decision-making about screening and subsequent treatment. A recent study of decision-making for osteoporosis treatment showed that allowing patients to make autonomous decisions after being provided information on the benefits and harms of treatment can result in better persistence with medication [246]. Most (91%) of the females in this study who started pharmacotherapy continued to be treated after 1 year of follow-up [246].

### Strengths and limitations

We comprehensively reviewed evidence related to the benefits and harms of screening for the primary prevention of fragility fractures by first considering direct evidence from screening trials, and supplementing this by reviews on the accuracy of risk assessment, benefits and harms of treatment, and patient perceptions of the acceptability of screening and treatment. To our knowledge, this is the first systematic review to synthesize evidence on the calibration of fracture risk assessment tools. We implemented rigorous searches to locate all potentially relevant studies; though our searches were limited to English and French language studies, this has been shown not to bias the effect estimates from meta-analyses [247]. We limited our update search for the accuracy of risk assessment tools to Canadian studies because these were thought to be the most relevant; the studies included for other tools were all affected by serious risk of bias (among), such that conclusions were unlikely to be impacted by this limit. We did not update the evidence for KQ3a on the benefits of treatment because this data did not weigh heavily into the Task Force’s decision making for their guideline on screening, for which there were several RCTs. Since we took a rapid approach to KQ3b (harms of treatment), there is the small possibility that relevant systematic reviews were missed or that minor errors were overlooked; by using an experienced reviewer, we reduced the likelihood of major omissions that would impact the findings [248]. It is also possible that the evidence for this KQ was less up to date (versus using primary studies) or did not examine all outcomes of interest that could be available in primary studies; moderate certainty of evidence would suggest stable findings for several outcomes. For KQ2 (accuracy of risk prediction tools), we did not review discrimination as it was not rated as critical or important by the Task Force; reported findings from the USPSTF review [60] are therefore less up to date.

There was some indirectness in our findings due to populations, interventions, comparators, and outcomes differing from those of primary interest. Our findings focus mainly on a *selected population* of patients who completed a mailed clinical FRAX tool independently and who are likely to be more compliant with screening and potentially treatment than the general population. This differs to some extent from clinical practice, where ideally decisions about screening would be made in shared decision-making with between patients and providers, after which patients would have the opportunity to consider their level of risk, along with their perceived benefits and harms of treatment. In addition, some participants in the screening trials had previously used anti-osteoporosis drugs, and the comparator included ad hoc treatment. Across all KQs, the ascertainment of clinical fragility fractures was problematic; definitions differed across studies and in some cases could have included non-clinical vertebral fractures, or other fractures that were not related to fragility (e.g., due to trauma). Our findings were robust to sensitivity analyses removing studies with unclear ascertainment of outcomes, or including only a single type of fracture (e.g., if multiple were added to determine a total number, rather than number of patients with ≥1 fracture). There was concern for selective reporting across some outcomes. Minimal discussion of potential harms was included across the screening trials; in the treatment trials, it was often unclear whether fracture data were collected systematically, and many did not report on clinical vertebral fractures (though this information should have been available).

The evidence in this review is most applicable to postmenopausal females aged 65 and over. We located very limited evidence for males and younger females, and there were no screening trial data specific to females aged 55 to 65 years. In addition, though one trial provided evidence of increased effectiveness of screening among those at higher baseline risk, there is a need for analyses from other trials to substantiate these findings. There is a need for robust comparative effectiveness trials to inform the most effective screening strategy. Examining whether different treatment approaches may positively impact effects for those at high risk based on screening for fracture risk, especially for those individuals nonadherent or uninterested in anti-osteoporosis medications, may also be of value.

## Conclusion

Screening in primary care using clinical FRAX, followed by BMD assessment in those at increased risk, among selected females aged 65 years and older who are likely to be more compliant with screening (as ascertained by their willingness to independently complete a risk assessment questionnaire) probably results in a small reduction in the risk of clinical fragility fracture and hip fracture compared to no screening. This may differ to some extent from clinical practice, where healthcare providers would ideally engage in shared decision-making about screening and discuss the results of fracture risk estimation, as well as the risks and benefits of treatment, during the patient consultation. A mailed offer of screening in the general population, where uptake was relatively low, did not improve any patient-important outcomes. Minimal information on harms is available, although our calculated estimates of overdiagnosis were 12 and 19% for hip and major osteoporotic fractures, respectively. The mechanism of the reduction in risk with screening is not fully clear, though there is some evidence to suggest it may be attributed to pharmacologic treatment rather than a reduction in falls or other risk behaviors. It is not clear which screening strategy would be most beneficial. The screening trials used diverse criteria when deciding for whom to offer treatment. There is some evidence for clinical FRAX and FRAX + BMD being adequately calibrated (particularly for clinical fragility fractures), with some underestimation, among Canadian studies; CAROC seems adequately calibrated to predict a category of risk and requires a BMD measurement. Treatment with bisphosphonates in primary prevention populations (at risk, but without prior fracture) probably reduces the risk of clinical fragility fractures and may reduce the risk of hip fractures and clinical vertebral fractures among postmenopausal females, to a similar magnitude as seen in the screening trials. Denosumab probably reduces the risk of clinical fragility fractures and clinical vertebral fractures but may not reduce the risk of hip fractures in postmenopausal females; evidence for males is very uncertain. Females at low risk seem to have a high willingness to be screened but there is large heterogeneity in the level of risk at which higher-risk patients would accept treatment, supporting a shared decision-making approach. The findings of this review will be used, among several other considerations (e.g., information on issues of feasibility, acceptability, costs/resources, and equity) by the Canadian Task Force on Preventive Health Care to inform recommendations on screening for the prevention of fragility fractures among adults 40 years and older in primary care in Canada.

## Supplementary Information

**Additional file 1.** PRISMA checklist. This file shows the completed reporting checklist for the systematic reviews.

**Additional file 2.** Search strategy for KQ3b. This file shows the full search strategy for key question 3b on the harms of pharmacologic treatment.

**Additional file 3.** Evidence sets for KQ1a-b. GRADE Evidence Profiles and Summary of Findings for KQ1. This file shows the analyses and GRADE Evidence Profiles, Summary of Findings, and contributing data for key question 1a on the benefits and harms of screening, and key question 1b on the comparative benefits and harms of different screening approaches.

**Additional file 4.** Evidence sets for KQs 2-4. GRADE Summary of Findings for KQs 2-4. This file shows the analyses and GRADE Summary of Findings for key questions 2 (accuracy of screening tests), 3a (benefits of treatment), 3b (harms of treatment), and 4 (acceptability of screening/treatment).

**Additional file 5.** Excluded studies list. This file lists all studies excluded after full text appraisal, with reasons.

**Additional file 6.** KQ 2-4 study characteristics. This file shows the descriptive characteristics of the included studies for key questions 2 (accuracy of screening tests), 3a (benefits of treatment), 3b (harms of treatment), and 4 (acceptability of screening/treatment).

**Additional file 7.** KQ 2-4 Risk of bias assessments. This file shows the risk of bias/quality appraisals for key questions 2 (accuracy of screening tests), 3a (benefits of treatment), 3b (harms of treatment), and 4 (acceptability of screening/treatment).

**Additional file 8.** Responses to stakeholder comments. This file contains responses to the comments submitted by stakeholder during their review of a draft of this manuscript. Written informed consent to publish was obtained from the stakeholders who provided the stakeholder reviews. A copy of the written consent is available for review by the Editors-in-Chief of this journal. The stakeholder reviews have been anonymized.

## Data Availability

The data generated during this study are available within the manuscript or its supplementary files.

## Abbreviations

AUC: Area under the curve
BMD: Bone mineral density
CAROC: Canadian Association of Radiologists and Osteoporosis Canada tool
CI: Confidence interval
DXA: Dual-energy X-ray absorptiometry
FRAX: Fracture Risk Assessment Tool
FRC: Fracture risk calculator
FRISC: Fracture and Immobilization Score
GRADE: Grading of Recommendations Assessment, Development and Evaluation
KQ: Key question
NNH: Number needed to treat for an additional harmful outcome
NNS: Number needed to screen
NNT: Number needed to treat for an additional beneficial outcome
PROSPERO: International prospective register of systematic reviews
SCORE: Simple Calculated Osteoporosis Risk Estimation
SOF: Study of Osteoporotic Fractures

## References

[1] Leslie WD, Schousboe JT (2011). A review of osteoporosis diagnosis and treatment options in new and recently updated guidelines on case finding around the world. Curr Osteoporos Rep..

[2] Kanis JA (2002). Diagnosis of osteoporosis and assessment of fracture risk. Lancet..

[3] Crandall CJ, Larson J, Manson JE, Cauley JA, LaCroix AZ, Wactawski-Wende J (2019). A comparison of US and Canadian osteoporosis screening and treatment strategies in postmenopausal women. J Bone Miner Res..

[4] Merlijn T, Swart KMA, van Schoor NM, Heymans MW, van der Zwaard BC, van der Heijden AA (2019). The effect of a screening and treatment program for the prevention of fractures in older women: a randomized pragmatic trial. J Bone Miner Res..

[5] Rubin KH, Rothmann MJ, Holmberg T, Hoiberg M, Moller S, Barkmann R (2018). Effectiveness of a two-step population-based osteoporosis screening program using FRAX: the randomized Risk-stratified Osteoporosis Strategy Evaluation (ROSE) study. Osteoporos Int..

[6] Shepstone L, Lenaghan E, Cooper C, Clarke S, Fong-Soe-Khioe R, Fordham R (2018). Screening in the community to reduce fractures in older women (SCOOP): a randomised controlled trial. Lancet..

[7] Merlijn T, Swart KMA, van der Horst HE, Netelenbos JC, Elders PJM (2020). Fracture prevention by screening for high fracture risk: a systematic review and meta-analysis. Osteoporos Int..

[8] Lentle B, Cheung AM, Hanley DA, Leslie WD, Lyons D, Papaioannou A (2011). Osteoporosis Canada 2010 guidelines for the assessment of fracture risk. Can Assoc Radiol J..

[9] Friedman SM, Mendelson DA (2014). Epidemiology of fragility fractures. Clin Geriatr Med..

[10] McCloskey EV, Vasireddy S, Threlkeld J, Eastaugh J, Parry A, Bonnet N (2008). Vertebral fracture assessment (VFA) with a densitometer predicts future fractures in elderly women unselected for osteoporosis. J Bone Miner Res..

[11] Morin SN, Lix LM, Leslie WD (2014). The importance of previous fracture site on osteoporosis diagnosis and incident fractures in women. J Bone Miner Res..

[12] Hodsman AB, Leslie WD, Tsang JF, Gamble GD (2008). 10-year probability of recurrent fractures following wrist and other osteoporotic fractures in a large clinical cohort: an analysis from the manitoba bone density program. Arch Intern Med..

[13] Hippisley-Cox J, Coupland C (2009). Predicting risk of osteoporotic fracture in men and women in England and Wales: prospective derivation and validation of QFractureScores. BMJ..

[14] Robbins J, Aragaki AK, Kooperberg C (2007). Factors associated with 5-year risk of hip fracture in postmenopausal women. JAMA..

[15] Prior JC, Langsetmo L, Lentle BC, Berger C, Goltzman D, Kovacs CS (2015). Ten-year incident osteoporosis-related fractures in the population-based Canadian Multicentre Osteoporosis Study—comparing site and age-specific risks in women and men. Bone..

[16] Langdahl BL (2017). Osteoporosis in premenopausal women. Curr Opin Rheumatol..

[17] Cohen A (2017). Premenopausal osteoporosis. Endocrinol Metab Clin North Am..

[18] World Health Organization. WHO Technical Report Series: assessment of fracture risk and its application to screening for postmenopausal osteoporosis. Geneva: WHO; 1994. 7941614

[19] Curtis EM, Moon RJ, Harvey NC, Cooper C (2017). The impact of fragility fracture and approaches to osteoporosis risk assessment worldwide. Bone..

[20] Odén A, McCloskey EV, Kanis JA, Harvey NC, Johansson H (2015). Burden of high fracture probability worldwide: secular increases 2010-2040. Osteoporos Int..

[21] Public Health Agency of Canada. Public Health Infobase: Canadian Chronic Disease Indicators. Ottawa: PHAC; 2018.

[22] Adachi JD, Adami S, Gehlbach S, Anderson FA, Boonen S, Chapurlat RD (2010). Impact of prevalent fractures on quality of life: baseline results from the global longitudinal study of osteoporosis in women. Mayo Clin Proc..

[23] Ioannidis G, Papaioannou A, Hopman WM, Akhtar-Danesh N, Anastassiades T, Pickard L (2009). Relation between fractures and mortality: results from the Canadian Multicentre Osteoporosis Study. CMAJ..

[24] Papaioannou A, Kennedy CC, Ioannidis G, Sawka A, Hopman WM, Pickard L (2009). The impact of incident fractures on health-related quality of life: 5 years of data from the Canadian Multicentre Osteoporosis Study. Osteoporos Int..

[25] Tarride JE, Hopkins RB, Leslie WD, Morin S, Adachi JD, Papaioannou A (2012). The burden of illness of osteoporosis in Canada. Osteoporos Int..

[26] Njeh CF, Fuerst T, Hans D, Blake GM, Genant HK (1999). Radiation exposure in bone mineral density assessment. Appl Radiat Isot..

[27] Hansen CA, Abrahamsen B, Konradsen H, Pedersen BD (2017). Women’s lived experiences of learning to live with osteoporosis: a longitudinal qualitative study. BMC Womens Health..

[28] Barker KL, Toye F, Lowe CJM (2016). A qualitative systematic review of patients’ experience of osteoporosis using meta-ethnography. Arch Osteoporos..

[29] Hayawi LM, Graham ID, Tugwell P, Yousef AS (2018). Screening for osteoporosis: a systematic assessment of the quality and content of clinical practice guidelines, using the AGREE II instrument and the IOM Standards for Trustworthy Guidelines. PLoS one..

[30] Papaioannou A, Morin S, Cheung AM, Atkinson S, Brown JP, Feldman S (2010). 2010 clinical practice guidelines for the diagnosis and management of osteoporosis in Canada: summary. CMAJ..

[31] United States Preventive Services Task Force (2018). Screening for osteoporosis to prevent fractures: US Preventive Services Task Force recommendation statement. JAMA..

[32] North American Menopause Society (2006). Management of osteoporosis in postmenopausal women: 2006 position statement of The North American Menopause Society. Menopause (New York, NY)..

[33] Siminoski K, O'Keeffe M, Brown JP, Burrell S, Coupland D, Dumont M (2013). Canadian Association of Radiologists technical standards for bone mineral densitometry reporting. Can Assoc Radiol J..

[34] The International Society for Clinical Densitometry. ISCD official position: Adults 2015 [Available from: https://www.iscd.org/official-positions/2015-iscd-official-positions-adult/].

[35] Watts NB, Adler RA, Bilezikian JP, Drake MT, Eastell R, Orwoll ES (2012). Osteoporosis in men: an Endocrine Society clinical practice guideline. J Clin Endocrinol Metab..

[36] Rubin KH, Friis-Holmberg T, Hermann AP, Abrahamsen B, Brixen K (2013). Risk assessment tools to identify women with increased risk of osteoporotic fracture: complexity or simplicity? A systematic review. J Bone Miner Res..

[37] Viswanathan M, Reddy S, Berkman N, Cullen K, Middleton JC, Nicholson WK (2018). Screening to prevent osteoporotic fractures: updated evidence report and systematic review for the US Preventive Services Task Force. JAMA..

[38] Moons KGM, de Groot JAH, Bouwmeester W, Vergouwe Y, Mallett S, Altman DG (2014). Critical appraisal and data extraction for systematic reviews of prediction modelling studies: The CHARMS checklist. PLOS Med..

[39] Kanis JA, Harvey NC, Cooper C, Johansson H, Oden A, McCloskey EV (2016). A systematic review of intervention thresholds based on FRAX : a report prepared for the National Osteoporosis Guideline Group and the International Osteoporosis Foundation. Arch Osteoporos..

[40] Siminoski K, Leslie WD, Frame H, Hodsman A, Josse RG, Khan A (2005). Recommendations for bone mineral density reporting in Canada. Can Assoc Radiol J..

[41] Cosman F, de Beur SJ, LeBoff MS, Lewiecki EM, Tanner B, Randall S (2014). Clinician's guide to prevention and treatment of osteoporosis. Osteoporos Int..

[42] Compston J, Cooper A, Cooper C, Gittoes N, Gregson C, Harvey N (2017). UK clinical guideline for the prevention and treatment of osteoporosis. Arch Osteoporos..

[43] Chakhtoura M, Baddoura R, El-Hajj Fuleihan G (2013). Lebanese FRAX-Based Osteoporosis Guidelines.

[44] Kanis JA, Cooper C, Rizzoli R, Reginster JY (2019). European guidance for the diagnosis and management of osteoporosis in postmenopausal women. Osteoporos Int..

[45] Beithon J, Gallenberg M, Johnson K, Kildahl P, Krenik J, Liebow M (2017). Institute for Clinical Systems Improvement: diagnosis and treatment of osteoporosis.

[46] Eastell R, Rosen CJ, Black DM, Cheung AM, Murad MH, Shoback D (2019). Pharmacological management of osteoporosis in postmenopausal women: An Endocrine Society Clinical Practice Guideline. J Clin Endocrinol Metab..

[47] Camacho PM, Petak SM, Binkley N, Clarke BL, Harris ST, Hurley DL (2016). American Association of Clinical Endocrinologists and American College of Endocrinology clinical practice guidelines for the diagnosis and treatment of postmenopausal osteoporosis—2016. Endocr Pract..

[48] Qaseem A, Forciea M, McLean RM, Denberg TD (2017). Treatment of low bone density or osteoporosis to prevent fractures in men and women: a clinical practice guideline update from the American College of Physicians. Ann Intern Med..

[49] Crandall CJ, Newberry SJ, Diamant A, Lim YW, Gellad WF, Suttorp MJ (2012). Treatment to prevent fractures in men and women with low bone density or osteoporosis: Update of a 2007 Report.

[50] Keshishian A, Boytsov N, Burge R, Krohn K, Lombard L, Zhang X (2017). Examining the effect of medication adherence on risk of subsequent fracture among women with a fragility fracture in the U.S. Medicare Population. J Manag Care Spec Pharm.

[51] Fatoye F, Smith P, Gebrye T, Yeowell G (2019). Real-world persistence and adherence with oral bisphosphonates for osteoporosis: a systematic review. BMJ Open..

[52] Hiligsmann M, Bours SPG, Boonen A (2015). A review of patient preferences for osteoporosis drug treatment. Curr Rheumatol Rep..

[53] Billington EO, Feasel AL, Kline GA. At odds about the odds: women’s choices to accept osteoporosis medications do not closely agree with physician-set treatment thresholds. J Gen Intern Med. 2020;35(1):276-82. 10.1007/s11606-019-05384-x PMC6957614 31625042

[54] Pillay J, Riva JJ, Tessier LA, Colquhoun H, Lang E, Moore AE (2021). Fall prevention interventions for older community-dwelling adults: systematic reviews on benefits, harms, and patient values and preferences. Syst Rev..

[55] Gates M, Pillay J, Thériault G, Limburg H, Grad R, Klarenbach S (2019). Screening to prevent fragility fractures among adults 40 years and older in primary care: protocol for a systematic review. Syst Rev..

[56] Canadian Task Force on Preventive Health Care. Procedure Manual 2014 [Available from: https://canadiantaskforce.ca/methods/].

[57] Page MJ, McKenzie JE, Bossuyt PM, Boutron I, Hoffmann TC, Mulrow CD (2021). The PRISMA 2020 statement: an updated guideline for reporting systematic reviews. BMJ..

[58] Guyatt GH, Oxman AD, Kunz R, Atkins D, Brozek J, Vist G (2011). GRADE guidelines: 2. Framing the question and deciding on important outcomes. J Clin Epidemiol..

[59] Theriault G, Reynolds D, Pillay J, Limburg H, Grad R, Gates M, et al. Expanding the measurement of overdiagnosis in the context of disease precursors and risk factors. BMJ Evid Based Med. 2023;bmjebm-2022-112117. 10.1136/bmjebm-2022-112117. 10.1136/bmjebm-2022-112117 36627178

[60] Viswanathan M, Reddy S, Berkman N, Cullen K, Middleton JC, Nicholson WK (2018). Screening to prevent osteoporotic fractures: an evidence review for the US Preventive Services Task Force.

[61] Whiting P, Jelena S, Higgins LPT, Caldwell DM, Reeves BC, Shea B (2016). ROBIS: a new tool to assess risk of bias in systematic reviews was developed. J Clin Epidemiol..

[62] McGowan J, Sampson M, Salzwedel DM, Cogo E, Foerster V, Lefebvre C (2016). PRESS peer review of electronic search strategies: 2015 guideline statement. J Clin Epidemiol..

[63] Higgins JPT, Altman DG, Gøtzsche PC, Jüni P, Moher D, Oxman AD (2011). The Cochrane Collaboration’s tool for assessing risk of bias in randomised trials. BMJ..

[64] Moons KM, Wolff RF, Riley RD (2019). Probast: a tool to assess risk of bias and applicability of prediction model studies: explanation and elaboration. Ann Intern Med..

[65] Shea BJ, Reeves BC, Wells G, Thuku M, Hamel C, Moran J (2017). AMSTAR 2: a critical appraisal tool for systematic reviews that include randomised or non-randomised studies of healthcare interventions, or both. BMJ..

[66] Zhang Y, Alonso-Coello P, Guyatt GH, Yepes-Nuñez JJ, Akl EA, Hazlewood G (2019). GRADE Guidelines: 19. Assessing the certainty of evidence in the importance of outcomes or values and preferences-Risk of bias and indirectness. J Clin Epidemiol..

[67] DerSimonian R, Laird N (1986). Meta-analysis in clinical trials. Control Clin Trials..

[68] Kern LM, Powe NR, Levine MA, Fitzpatrick AL, Harris TB, Robbins J (2005). Association between screening for osteoporosis and the incidence of hip fracture. Ann Intern Med..

[69] Sweeting M, Sutton A, Lambert P (2004). What to add to nothing? Use and avoidance of continuity corrections in meta-analysis of sparse data. Stat Med..

[70] Schünemann HJ, Higgins J, Vist G, Glasziou P, Akl E, Skoetz N (2020). Chapter 14: Completing ‘Summary of findings’ tables and grading the certainty of the evidence. Cochrane Handbook for Systematic Reviews of Interventions version 61, Cochrane.

[71] Statistics Canada (2018). Table 13-10-0710-01: Deaths and mortality rate, by age group.

[72] Schünemann HJ, Vist G, Higgins J, Santesso N, Deeks JJ, Glasziou P (2020). Chapter 15: Interpreting results and drawing conclusions. Cochrane Handbook for Systematic Reviews of Interventions version 61: Cochrane.

[73] Cornell JE, Mulrow CD, Localio R, Stack CB, Meibohm AR, Guallar E (2014). Random-effects meta-analysis of inconsistent effects: a time for change. Ann Intern Med..

[74] IntHout J, Ioannidis JP, Borm GF (2014). The Hartung-Knapp-Sidik-Jonkman method for random effects meta-analysis is straightforward and considerably outperforms the standard DerSimonian-Laird method. BMC Med Res Methodol..

[75] Snell KIE (2015). Development and application of statistical methods for prognosis research (Doctoral Thesis).

[76] van Klaveren D, Steyerberg EW, Perel P, Vergouwe Y (2014). Assessing discriminative ability of risk models in clustered data. BMC Med Res Methodol..

[77] Qin G, Hotilovac L (2008). Comparison of non-parametric confidence intervals for the area under the ROC curve of a continuous-scale diagnostic test. Stat Methods Med Res..

[78] Popay J, Roberts H, Sowden A, Petticrew M, Arai L, Rodgers M (2006). Guidance on the conduct of narrative synthesis in systematic reviews: a product from the ESRC Methods Programme.

[79] Harbord RM, Egger M, Sterne JA (2006). A modified test for small-study effects in meta-analyses of controlled trials with binary endpoints. Stat Med..

[80] Guyatt GH, Oxman AD, Kunz R, Brozek J, Alonso-Coello P, Rind D (2011). GRADE guidelines 6. Rating the quality of evidence—imprecision. J Clin Epidemiol..

[81] Guyatt GH, Oxman AD, Kunz R, Woodcock J, Brozek J, Helfand M (2011). GRADE guidelines: 8. Rating the quality of evidence—indirectness. J Clin Epidemiol..

[82] Guyatt GH, Oxman AD, Kunz R, Woodcock J, Brozek J, Helfand M (2011). GRADE guidelines: 7. Rating the quality of evidence--inconsistency. J Clin Epidemiol..

[83] Guyatt GH, Oxman AD, Montori V, Vist G, Kunz R, Brozek J (2011). GRADE guidelines: 5. Rating the quality of evidence—publication bias. J Clin Epidemiol..

[84] Guyatt GH, Oxman AD, Vist G, Kunz R, Brozek J, Alonso-Coello P (2011). GRADE guidelines: 4. Rating the quality of evidence—study limitations (risk of bias). J Clin Epidemiol..

[85] Guyatt GH, Oxman AD, Vist GE, Kunz R, Falck-Ytter Y, Alonso-Coello P (2008). GRADE: an emerging consensus on rating quality of evidence and strength of recommendations. BMJ..

[86] Hultcrantz M, Rind D, Akl EA, Treweek S, Mustafa RA, Iorio A (2017). The GRADE Working Group clarifies the construct of certainty of evidence. J Clin Epidemiol..

[87] Murad MH, Mustafa RA, Schünemann HJ, Sultan S, Santesso N (2017). Rating the certainty in evidence in the absence of a single estimate of effect. Evid Based Med..

[88] Debray TPA, Damen JAAG, Snell KIE, Ensor J, Hooft L, Reitsma JB (2017). A guide to systematic review and meta-analysis of prediction model performance. BMJ..

[89] Santesso N, Glenton C, Dahm P, Garner P, Akl EA, Alper B (2020). GRADE guidelines 26: informative statements to communicate the findings of systematic reviews of interventions. J Clin Epidemiol..

[90] Barr RJ, Stewart A, Torgerson DJ, Reid DM (2010). Population screening for osteoporosis risk: a randomised control trial of medication use and fracture risk. Osteoporos Int..

[91] McCloskey E, Johansson H, Harvey NC, Shepstone L, Lenaghan E, Fordham R (2018). Management of patients with high baseline hip fracture risk by FRAX reduces hip fractures-a post hoc analysis of the SCOOP study. J Bone Miner Res..

[92] Torgerson DJ, Thomas RE, Campbell MK, Reid DM (1997). Randomized trial of osteoporosis screening. Use of hormone replacement therapy and quality-of-life results. Arch Intern Med..

[93] Lacroix AZ, Buist DS, Brenneman SK, Abbott TA (2005). Evaluation of three population-based strategies for fracture prevention: results of the osteoporosis population-based risk assessment (OPRA) trial. Med Care..

[94] Kanis JA, Odén A, McCloskey EV, Johansson H, Wahl DA, Cooper C (2012). A systematic review of hip fracture incidence and probability of fracture worldwide. Osteoporos Int..

[95] Fried LP, Borhani NO, Enright P, Furberg CD, Gardin JM, Kronmal RA (1991). The Cardiovascular Health Study: design and rationale. Ann Epidemiol..

[96] Azagra R, Roca G, Encabo G, Aguye A, Zwart M, Guell S (2012). FRAX(R) tool, the WHO algorithm to predict osteoporotic fractures: the first analysis of its discriminative and predictive ability in the Spanish FRIDEX cohort. BMC Musculoskelet Disord..

[97] Azagra R, Roca G, Martin-Sanchez JC, Casado E, Encabo G, Zwart M (2015). FRAX(R) thresholds to identify people with high or low risk of osteoporotic fracture in Spanish female population. Med Clin..

[98] Azagra R, Zwart M, Aguye A, Martin-Sanchez JC, Casado E, Diaz-Herrera MA (2016). Fracture experience among participants from the FROCAT study: what thresholding is appropriate using the FRAX tool?. Maturitas..

[99] Azagra R, Zwart M, Encabo G, Aguye A, Martin-Sanchez JC, Puchol-Ruiz N (2016). Rationale of the Spanish FRAX model in decision-making for predicting osteoporotic fractures: an update of FRIDEX cohort of Spanish women. BMC Musculoskelet Disord..

[100] Bolland MJ, Siu AT, Mason BH, Horne AM, Ames RW, Grey AB (2011). Evaluation of the FRAX and Garvan fracture risk calculators in older women. J Bone Miner Res..

[101] Bolton JM, Morin SN, Majumdar SR, Sareen J, Lix LM, Johansson H (2017). Association of mental disorders and related medication use with risk for major osteoporotic fractures. JAMA Psychiatry..

[102] Brennan SL, Leslie WD, Lix LM, Johansson H, Oden A, McCloskey E (2014). FRAX provides robust fracture prediction regardless of socioeconomic status. Osteoporos Int..

[103] Buehring B, Hansen KE, Lewis BL, Cummings SR, Lane NE, Binkley N (2018). Dysmobility syndrome independently increases fracture risk in the osteoporotic fractures in men (MrOS) prospective cohort study. J Bone Miner Res..

[104] Crandall CJ, Larson J, LaCroix A, Cauley JA, LeBoff MS, Li W (2019). Predicting fracture risk in younger postmenopausal women: comparison of the Garvan and FRAX Risk Calculators in the Women's Health Initiative Study. J Gen Intern Med..

[105] Crandall CJ, Larson JC, Watts NB, Gourlay ML, Donaldson MG, LaCroix A (2014). Comparison of fracture risk prediction by the US Preventive Services Task Force strategy and two alternative strategies in women 50-64 years old in the Women's Health Initiative. J Clin Endocrinol Metab..

[106] Crandall CJ, Schousboe JT, Morin SN, Lix LM, Leslie W (2019). Performance of FRAX and FRAX-based treatment thresholds in women aged 40 years and older: the Manitoba BMD Registry. J Bone Miner Res..

[107] Czerwinski E, Borowy P, Kumorek A, Amarowicz J, Gorkiewicz M, Milert A (2013). Fracture risk prediction in outpatients from Krakow Region using FRAX tool versus fracture risk in 11-year follow-up. Ortop Traumatol Rehabil..

[108] Dagan N, Cohen-Stavi C, Leventer-Roberts M, Balicer RD (2017). External validation and comparison of three prediction tools for risk of osteoporotic fractures using data from population based electronic health records: retrospective cohort study. BMJ..

[109] Ettinger B, Ensrud KE, Blackwell T, Curtis JR, Lapidus JA, Orwoll ES (2013). Performance of FRAX in a cohort of community-dwelling, ambulatory older men: the Osteoporotic Fractures in Men (MrOS) study. Osteoporos Int..

[110] Ettinger B, Liu H, Blackwell T, Hoffman AR, Ensrud KE, Orwoll ES (2012). Validation of FRC, a fracture risk assessment tool, in a cohort of older men: the Osteoporotic Fractures in Men (MrOS) Study. J Clin Densitom..

[111] Fraser LA, Langsetmo L, Berger C, Ioannidis G, Goltzman D, Adachi JD (2011). Fracture prediction and calibration of a Canadian FRAX(R) tool: a population-based report from CaMos. Osteoporos Int..

[112] Goldshtein I, Gerber Y, Ish-Shalom S, Leshno M (2018). Fracture risk assessment with FRAX using real-world data in a population-based cohort from Israel. Am J Epidemiol..

[113] Gourlay ML, Ritter VS, Fine JP, Overman RA, Schousboe JT, Cawthon PM (2017). Comparison of fracture risk assessment tools in older men without prior hip or spine fracture: the MrOS study. Arch Osteoporos..

[114] Harvey NC, Oden A, Orwoll E, Lapidus J, Kwok T, Karlsson MK (2018). Falls predict fractures independently of FRAX probability: a Meta-Analysis of the Osteoporotic Fractures in Men (MrOS) study. J Bone Miner Res..

[115] Hillier TA, Cauley JA, Rizzo JH, Pedula KL, Ensrud KE, Bauer DC (2011). WHO absolute fracture risk models (FRAX): do clinical risk factors improve fracture prediction in older women without osteoporosis?. J Bone Miner Res..

[116] Holloway KL, Mohebbi M, Betson AG, Hans D, Hyde NK, Brennan-Olsen SL (2018). Prediction of major osteoporotic and hip fractures in Australian men using FRAX scores adjusted with trabecular bone score. Osteoporos Int..

[117] Iki M, Fujita Y, Tamaki J, Kouda K, Yura A, Sato Y (2015). Trabecular bone score may improve FRAX(R) prediction accuracy for major osteoporotic fractures in elderly Japanese men: the Fujiwara-kyo Osteoporosis Risk in Men (FORMEN) Cohort Study. Osteoporos Int..

[118] Kalvesten J, Lui LY, Brismar T, Cummings S (2016). Digital X-ray radiogrammetry in the study of osteoporotic fractures: Comparison to dual energy X-ray absorptiometry and FRAX. Bone..

[119] Langsetmo L, Nguyen TV, Nguyen ND, Kovacs CS, Prior JC, Center JR (2011). Independent external validation of nomograms for predicting risk of low-trauma fracture and hip fracture. CMAJ..

[120] Langsetmo L, Peters KW, Burghardt AJ, Ensrud KE, Fink HA, Cawthon PM (2018). Volumetric bone mineral density and failure load of distal limbs predict incident clinical fracture independent HR-pQCT BMD and failure load predicts incident clinical fracture of FRAX and clinical risk factors among older men. J Bone Miner Res..

[121] Leslie WD, Berger C, Langsetmo L, Lix LM, Adachi JD, Hanley DA (2011). Construction and validation of a simplified fracture risk assessment tool for Canadian women and men: results from the CaMos and Manitoba cohorts. Osteoporos Int..

[122] Leslie WD, Brennan SL, Lix LM, Johansson H, Oden A, McCloskey E (2013). Direct comparison of eight national FRAX(R) tools for fracture prediction and treatment qualification in Canadian women. Arch Osteoporos..

[123] Leslie WD, Johansson H, Kanis JA, Lamy O, Oden A, McCloskey EV (2014). Lumbar spine texture enhances 10-year fracture probability assessment. Osteoporos Int..

[124] Leslie WD, Lix LM (2010). Simplified 10-Year Absolute Fracture Risk Assessment: A Comparison of Men and Women. J Clin Densitom..

[125] Leslie WD, Lix LM, Johansson H, Oden A, McCloskey E, Kanis JA (2010). Independent clinical validation of a Canadian FRAX tool: fracture prediction and model calibration. J Bone Miner Res..

[126] Leslie WD, Lix LM, Johansson H, Oden A, McCloskey E, Kanis JA (2012). A comparative study of using non-hip bone density inputs with FRAX(R). Osteoporos Int..

[127] Leslie WD, Lix LM, Majumdar SR, Morin SN, Johansson H, Oden A (2017). Total hip bone area affects fracture prediction with FRAX in Canadian white women. J Clin Endocrinol Metab..

[128] Leslie WD, Majumdar SR, Lix LM, Josse RG, Johansson H, Oden A (2016). Direct comparison of FRAX(R) and a simplified fracture risk assessment tool in routine clinical practice: a registry-based cohort study. Osteoporos Int..

[129] Leslie WD, Majumdar SR, Morin SN, Lix LM, Johansson H, Oden A (2017). FRAX for fracture prediction shorter and longer than 10 years: the Manitoba BMD registry. Osteoporos Int..

[130] Leslie WD, Majumdar SR, Morin SN, Lix LM, Schousboe JT, Ensrud KE (2018). Performance of FRAX in clinical practice according to sex and osteoporosis definitions: the Manitoba BMD registry. Osteoporos Int..

[131] Leslie WD, Morin S (2011). Fracture burden in relation to low bone mineral density and FRAX((R)) probability. J Clin Densitom..

[132] Leslie WD, Morin S, Lix LM, Johansson H, Oden A, McCloskey E (2012). Fracture risk assessment without bone density measurement in routine clinical practice. Osteoporos Int..

[133] Leslie WD, Tsang JF, Lix LM (2009). Simplified system for absolute fracture risk assessment: clinical validation in Canadian women. J Bone Miner Res..

[134] Li G, Thabane L, Papaioannou A, Adachi JD (2015). Comparison between frailty index of deficit accumulation and fracture risk assessment tool (FRAX) in prediction of risk of fractures. Bone..

[135] Lix LM, Leslie WD, Majumdar SR (2018). Measuring improvement in fracture risk prediction for a new risk factor: a simulation. BMC Res Notes..

[136] Lo JC, Pressman AR, Chandra M, Ettinger B (2011). Fracture risk tool validation in an integrated healthcare delivery system. Am J Manag Care..

[137] Majumdar SR, Leslie WD, Lix LM, Morin SN, Johansson H, Oden A (2016). Longer duration of diabetes strongly impacts fracture risk assessment: the Manitoba BMD Cohort. J Clin Endocrinol Metab..

[138] Marques A, Lucas R, Simoes E, Verstappen SMM, Jacobs JWG, da Silva JAP (2017). Do we need bone mineral density to estimate osteoporotic fracture risk? A 10-year prospective multicentre validation study. RMD Open..

[139] Martineau P, Leslie WD, Johansson H, Oden A, McCloskey EV, Hans D (2017). Clinical utility of using lumbar spine trabecular bone score to adjust fracture probability: the Manitoba BMD Cohort. J Bone Miner Res..

[140] Melton LJ, Atkinson EJ, Achenbach SJ, Kanis JA, Therneau TM, Johansson H (2012). Potential Extensions of the US FRAX Algorithm. J Osteoporos..

[141] Orwoll ES, Lapidus J, Wang PY, Vandenput L, Hoffman A, Fink HA (2017). The limited clinical utility of testosterone, estradiol, and sex hormone binding globulin measurements in the prediction of fracture risk and bone loss in older men. J Bone Miner Res..

[142] Pluskiewicz W, Adamczyk P, Czekajlo A, Grzeszczak W, Drozdzowska B (2015). High fracture probability predicts fractures in a 4-year follow-up in women from the RAC-OST-POL study. Osteoporos Int..

[143] Premaor M, Parker RA, Cummings S, Ensrud K, Cauley JA, Lui LY (2013). Predictive value of FRAX for fracture in obese older women. J Bone Miner Res..

[144] Pressman AR, Lo JC, Chandra M, Ettinger B (2011). Methods for assessing fracture risk prediction models: experience with FRAX in a large integrated health care delivery system. J Clin Densitom..

[145] Reyes Dominguez AI, Sosa Cabrera N, Saavedra Santana P, de Tejada Romero MJG, Jodar Gimeno E, Sosa HM (2017). Assessment of the predictive capacity of the garvan calculator of 10 year risk of fracture in a Spanish population. Rev de Osteoporos Metab Miner..

[146] Sornay-Rendu E, Munoz F, Delmas PD, Chapurlat RD (2010). The FRAX tool in French women: how well does it describe the real incidence of fracture in the OFELY cohort?. J Bone Miner Res..

[147] Tamaki J, Iki M, Kadowaki E, Sato Y, Kajita E, Kagamimori S (2011). Fracture risk prediction using FRAX(R): a 10-year follow-up survey of the Japanese Population-Based Osteoporosis (JPOS) Cohort Study. Osteoporos Int..

[148] Tamaki J, Iki M, Sato Y, Winzenrieth R, Kajita E, Kagamimori S (2019). Does Trabecular Bone Score (TBS) improve the predictive ability of FRAX for major osteoporotic fractures according to the Japanese Population-Based Osteoporosis (JPOS) cohort study?. J Bone Miner Metab..

[149] Tanaka S, Yoshimura N, Kuroda T, Hosoi T, Saito M, Shiraki M (2010). The Fracture and Immobilization Score (FRISC) for risk assessment of osteoporotic fracture and immobilization in postmenopausal women--A joint analysis of the Nagano, Miyama, and Taiji Cohorts. Bone..

[150] Tebe Cordomi C, Del Rio LM, Di Gregorio S, Casas L, Estrada MD, Kotzeva A (2013). Validation of the FRAX predictive model for major osteoporotic fracture in a historical cohort of Spanish women. J Clin Densitom..

[151] Tremollieres FA, Pouilles JM, Drewniak N, Laparra J, Ribot CA, Dargent-Molina P (2010). Fracture risk prediction using BMD and clinical risk factors in early postmenopausal women: sensitivity of the WHO FRAX tool. J Bone Miner Res..

[152] Yang S, Leslie WD, Morin SN, Lix LM (2019). Administrative healthcare data applied to fracture risk assessment. Osteoporos Int..

[153] Yin MT, Shiau S, Rimland D, Gibert CL, Bedimo RJ, Rodriguez-Barradas MC (2016). Fracture prediction with Modified-FRAX in older HIV-infected and uninfected men. J Acquir Immune Defic Syndr..

[154] Desbiens L-C, Mac-Way F, Sidibe A, Beaudoin C, Jean S (2020). Comparison of fracture prediction tools in individuals without and with early chronic kidney disease: a population-based analysis of CARTaGENE. J Bone Miner Res..

[155] Chapurlat R, Bui M, Sornay-Rendu E, Zebaze R, Delmas PD, Liew D (2020). Deterioration of cortical and trabecular microstructure identifies women with osteopenia or normal bone mineral density at imminent and long-term risk for fragility fracture: a prospective study. J Bone Miner Res..

[156] Crandall CJ, Larson J, Cauley JA, Schousboe JT, LaCroix AZ, Robbins JA (2019). Do additional clinical risk factors improve the performance of fracture risk assessment tool (FRAX) among postmenopausal women? Findings from the Women's Health Initiative Observational Study and Clinical Trials. JBMR Plus..

[157] Dagan N, Elnekave E, Barda N, Bregman-Amitai O, Bar A, Orlovsky M (2020). Automated opportunistic osteoporotic fracture risk assessment using computed tomography scans to aid in FRAX underutilization. Nat Med..

[158] de Abreu LLF, Holloway-Kew KL, Sajjad MA, Kotowicz MA, Pasco JA (2019). FRAX (Australia) scores in women with impaired fasting glucose and diabetes. Bone Rep..

[159] Harvey NC, Kanis JA, Liu E, Cooper C, Lorentzon M, Bea JW (2021). Predictive value of DXA appendicular lean mass for incident fractures, falls, and mortality, independent of prior falls, FRAX, and BMD: findings from the Women's Health Initiative (WHI). J Bone Miner Res..

[160] Holloway-Kew KL, Zhang Y, Betson AG, Anderson KB, Hans D, Hyde NK (2019). How well do the FRAX (Australia) and Garvan calculators predict incident fractures? Data from the Geelong Osteoporosis Study. Osteoporos Int..

[161] Kim H, Kim JH, Kim MJ, Hong AR, Choi H, Ku E (2020). Low predictive value of FRAX adjusted by trabecular bone score for osteoporotic fractures in Korean women: a community-based cohort study. Endocrinol Metab (Seoul, Korea)..

[162] Lu T, Forgetta V, Keller-Baruch J, Nethander M, Bennett D, Forest M (2021). Improved prediction of fracture risk leveraging a genome-wide polygenic risk score. Genome Med..

[163] Pickhardt PJ, Graffy PM, Zea R, Lee SJ, Liu J, Sandfort V (2020). Automated abdominal CT imaging biomarkers for opportunistic prediction of future major osteoporotic fractures in asymptomatic adults. Radiology..

[164] Pluskiewicz W, Adamczyk P, Drozdzowska B (2021). Height loss in postmenopausal women-do we need more for fracture risk assessment? Results from the GO Study. Osteoporos Int..

[165] Xiao X, Wu Q (2021). The utility of genetic risk score to improve performance of FRAX for fracture prediction in US postmenopausal women. Calcif Tissue Int..

[166] Bisson EJ, Finlayson M, Ekuma O, Marrie RA, Leslie WD (2018). Accuracy of FRAX in people with multiple sclerosis: a Manitoba BMD registry-based cohort study. J Bone Miner Res..

[167] Leslie WD, Lix LM, Binkley N (2020). Targeted vertebral fracture assessment for optimizing fracture prevention in Canada. Arch Osteoporos..

[168] Leslie WD, Morin SN, Lix LM, Binkley N (2019). Comparison of treatment strategies and thresholds for optimizing fracture prevention in Canada: a simulation analysis. Arch Osteoporos..

[169] Leslie WD, Morin SN, Lix LM, Binkley N (2020). Impact of spine-hip discordance on fracture risk assessment and treatment qualification in Canada: the Manitoba BMD registry. Arch Osteoporos..

[170] Leslie WD, Morin SN, Lix LM, Martineau P, Bryanton M, McCloskey EV (2019). Fracture prediction from self-reported falls in routine clinical practice: a registry-based cohort study. Osteoporos Int..

[171] Li G, Leslie WD, Kovacs CS, Prior J, Josse RG, Towheed T (2020). Combining frailty and trabecular bone score did not improve predictive accuracy in risk of major osteoporotic fractures. J Bone Miner Res..

[172] Ascott-Evans BH, Guañabens N, Kivinen S, Stuckey BGA, Magaril CH, Vandormael K (2003). Alendronate prevents loss of bone density associated with discontinuation of hormone replacement therapy: a randomized controlled trial. Arch Intern Med..

[173] Bell NH, Bilezikian JP, Bone Iii HG, Kaur A, Maragoto A, Santora AC (2002). Alendronate increases bone mass and reduces bone markers in postmenopausal African-American women. J Clin Endocrinol Metab..

[174] Bone HG, Bolognese MA, Yuen CK, Kendler DL, Wang H, Liu Y (2008). Effects of denosumab on bone mineral density and bone turnover in postmenopausal women. J Clin Endocrinol Metab..

[175] Boonen S, Reginster J-Y, Kaufman J-M, Lippuner K, Zanchetta J, Langdahl B (2012). Fracture risk and zoledronic acid therapy in men with osteoporosis. N Engl J Med..

[176] Chesnut CH, McClung MR, Ensrud KE, Bell NH, Genant HK, Harris ST (1995). Alendronate treatment of the postmenopausal osteoporotic woman: effect of multiple dosages on bone mass and bone remodeling. Am J Med..

[177] Cummings SR, Black DM, Thompson DE, Applegate WB, Barrett-Connor E, Musliner TA (1998). Effect of alendronate on risk of fracture in women with low bone density but without vertebral fractures results from the Fracture Intervention Trial. JAMA..

[178] Cummings SR, Martin JS, McClung MR, Siris ES, Eastell R, Reid IR (2009). Denosumab for prevention of fractures in postmenopausal women with osteoporosis. N Engl J Med..

[179] Fogelman I, Ribot C, Smith R, Ethgen D, Sod E, Reginster JY (2000). Risedronate reverses bone loss in postmenopausal women with low bone mass: results from a multinational, double-blind, placebo-controlled trial. BMD-MN Study Group. J Clin Endocrinol Metab..

[180] Grey A, Bolland M, Mihov B, Wong S, Horne A, Gamble G (2014). Duration of antiresorptive effects of low-dose zoledronate in osteopenic postmenopausal women: a randomized, placebo-controlled trial. J Bone Miner Res..

[181] Grey A, Bolland MJ, Wattie D, Horne A, Gamble G, Reid IR (2009). The antiresorptive effects of a single dose of zoledronate persist for two years: a randomized, placebo-controlled trial in osteopenic postmenopausal women. Obstet Gynecol Surv..

[182] Hooper MJ, Ebeling PR, Roberts AP, Graham JJ, Nicholson GC, D'Emden M (2005). Risedronate prevents bone loss in early postmenopausal women: a prospective randomized, placebo-controlled trial. Climacteric..

[183] Hosking D, Adami S, Felsenberg D, Andia JC, Valimaki M, Benhamou L (2003). Comparison of change in bone resorption and bone mineral density with once-weekly alendronate and daily risedronate: a randomised, placebo-controlled study. Curr Med Res Opin..

[184] Hosking D, Chilvers CE, Christiansen C, Ravn P, Wasnich R, Ross P (1998). Prevention of bone loss with alendronate in postmenopausal women under 60 years of age. Early Postmenopausal Intervention Cohort Study Group. N Engl J Med..

[185] Lewiecki EM, Miller PD, McClung MR, Cohen SB, Bolognese MA, Liu Y (2007). Two-year treatment with denosumab (AMG 162) in a randomized phase 2 study of postmenopausal women with low BMD. J Bone Miner Res..

[186] Li Y, Zhang Z, Deng X, Chen L (2005). Efficacy and safety of risedronate sodium in treatment of postmenopausal osteoporosis. J Huazhong Univ Sci Technol Med Sci..

[187] Liberman UA, Weiss SR, Bröll J, Minne HW, Quan H, Bell NH (1995). Effect of oral alendronate on bone mineral density and the incidence of fractures in postmenopausal osteoporosis. N Engl J Med..

[188] McClung M, Miller P, Recknor C, Mesenbrink P, Bucci-Rechtweg C, Benhamou CL (2009). Zoledronic acid for the prevention of bone loss in postmenopausal women with low bone mass: a randomized controlled trial. Obstet Gynecol..

[189] McClung MR, Geusens P, Miller PD, Zippel H, Bensen WG, Roux C (2001). Effect of risedronate on the risk of hip fracture in elderly women. N Engl J Med..

[190] Mortensen L, Charles P, Bekker PJ, Digennaro J, Johnston CC (1998). Risedronate increases bone mass in an early postmenopausal population: two years of treatment plus one year of follow-up. J Clin Endocrinol Metab..

[191] Orwoll E, Teglbjaerg CS, Langdahl BL, Chapurlat R, Czerwinski E, Kendler DL (2012). A randomized, placebo-controlled study of the effects of denosumab for the treatment of men with low bone mineral density. J Clin Endocrinol Metab..

[192] Pitale S, Thomas M, Rathi G, Deshmukh V, Kumar P, Reddy S (2015). A randomized placebo-controlled trial of the efficacy of denosumab in Indian postmenopausal women with osteoporosis. Indian J Endocrinol Metab..

[193] Pols HAP, Felsenberg D, Hanley DA, Štepán J, Muñoz-Torres M, Wilkin TJ (1999). Multinational, placebo-controlled, randomized trial of the effects of alendronate on bone density and fracture risk in postmenopausal women with low bone mass: results of the FOSIT Study. Osteoporos Int..

[194] Reid IR, Brown JP, Burckhardt P, Horowitz Z, Richardson P, Trechsel U (2002). Intravenous zoledronic acid in postmenopausal women with low bone mineral density. N Engl J Med..

[195] Reid IR, Horne AM, Mihov B, Stewart A, Garratt E, Wong S (2018). Fracture prevention with zoledronate in older women with osteopenia. N Engl J Med..

[196] Välimäiki MJ, Farrerons-Minguella J, Halse J, Kröger H, Maroni M, Mulder H (2007). Effects of risedronate 5 mg/d on bone mineral density and bone turnover markers in late-postmenopausal women with osteopenia: a multinational, 24-month, randomized, double-blind, placebo-controlled, parallel-group, phase III trial. Clin Ther..

[197] Yan Y, Wang W, Zhu H, Li M, Liu J, Luo B (2009). The efficacy and tolerability of once-weekly alendronate 70 mg on bone mineral density and bone turnover markers in postmenopausal Chinese women with osteoporosis. J Bone Miner Metab..

[198] Zhu HM, Tang H, Cheng Q, He L, Li PQ, Xue QY, et al. Efficacy and safety of denosumab in Chinese postmenopausal women with osteoporosis at increased risk of fracture: Results from a 12-month, randomized, double-blind, placebo-controlled phase III study. J Bone Miner Res. 2017;31(Suppl 1):S160.

[199] Boonen S, Adachi JD, Man Z, Cummings SR, Lippuner K, Törring O (2011). Treatment with denosumab reduces the incidence of new vertebral and hip fractures in postmenopausal women at high risk. J Clin Endocrinol Metab..

[200] Donaldson MG, Palermo L, Ensrud KE, Hochberg MC, Schousboe JT, Cummings SR (2012). Effect of alendronate for reducing fracture by FRAX score and femoral neck bone mineral density: the Fracture Intervention Trial. J Bone Miner Res..

[201] Grey A, Bolland M, Wong S, Horne A, Gamble G, Reid IR (2012). Low-dose zoledronate in osteopenic postmenopausal women: a randomized controlled trial. J Clin Endocrinol Metab..

[202] Grey A, Bolland MJ, Horne A, Mihov B, Gamble G, Reid IR (2017). Duration of antiresorptive activity of zoledronate in postmenopausal women with osteopenia: a randomized, controlled multidose trial. CMAJ..

[203] Hochberg MC, Thompson DE, Black DM, Quandt SA, Cauley J, Geusens P (2005). Effect of alendronate on the age-specific incidence of symptomatic osteoporotic fractures. J Bone Miner Res..

[204] McCloskey EV, Johansson H, Oden A, Austin M, Siris E, Wang A (2012). Denosumab reduces the risk of osteoporotic fractures in postmenopausal women, particularly in those with moderate to high fracture risk as assessed with FRAX. J Bone Miner Res..

[205] McClung MR, Boonen S, Törring O, Roux C, Rizzoli R, Bone HG (2012). Effect of denosumab treatment on the risk of fractures in subgroups of women with postmenopausal osteoporosis. J Bone Miner Res..

[206] McClung MR, Lewiecki EM, Cohen SB, Bolognese MA, Woodson GC, Moffett AH (2006). Denosumab in postmenopausal women with low bone mineral density. N Engl J Med..

[207] NCT (2016). Denosumab China Phase III Study. Clincaltrials.gov.

[208] Silverman S, Viswanathan HN, Yang YC, Wang A, Boonen S, Ragi-Eis S (2012). Impact of clinical fractures on health-related quality of life is dependent on time of assessment since fracture: results from the FREEDOM trial. Osteoporos Int..

[209] Tucci JR, Tonino RP, Emkey RD, Peverly CA, Kher U, Santora AC (1996). Effect of three years of oral alendronate treatment in postmenopausal women with osteoporosis. Am J Med..

[210] Chen LX, Ning GZ, Zhou ZR, Li YL, Zhang D, Wu QL (2015). The carcinogenicity of alendronate in patients with osteoporosis: evidence from cohort studies. PLOS ONE..

[211] Crandall CJ, Newberry SJ, Diamant A, Lim YW, Gellad WF, Booth MJ (2014). Comparative effectiveness of pharmacologic treatments to prevent fractures: an updated systematic review. Ann Intern Med..

[212] Davis S, Martyn-St James M, Sanderson J, Stevens J, Goka E, Rawdin A (2016). A systematic review and economic evaluation of bisphosphonates for the prevention of fragility fractures. Health Technol Assess (Winchester, England)..

[213] Davis S, Simpson E, Hamilton J, Martyn-St James M, Rawdin A, Wong R (2020). Denosumab, raloxifene, romosozumab and teriparatide to prevent osteoporotic fragility fractures: a systematic review and economic evaluation. Health Technol Assess..

[214] Diedhiou D, Cuny T, Sarr A, Norou Diop S, Klein M, Weryha G (2015). Efficacy and safety of denosumab for the treatment of osteoporosis: a systematic review. Ann Endocrinol..

[215] Fink HA, MacDonald R, Forte ML, Rosebush CE, Ensrud KE, Schousboe JT (2019). Long-term drug therapy and drug holidays for osteoporosis fracture prevention: a systematic review.

[216] Kranenburg G, Bartstra JW, Weijmans M, de Jong PA, Mali WP, Verhaar HJ (2016). Bisphosphonates for cardiovascular risk reduction: a systematic review and meta-analysis. Atherosclerosis..

[217] Lv F, Cai X, Yang W, Gao L, Chen L, Wu J (2020). Denosumab or romosozumab therapy and risk of cardiovascular events in patients with primary osteoporosis: systematic review and meta- analysis. Bone..

[218] Tsourdi E, Zillikens MC, Meier C, Body J-J, Rodriguez EG, Anastasilakis AD, et al. Fracture risk and management of discontinuation of denosumab therapy: a systematic review and position statement by ECTS. J Clin Endocrinol Metab. 2020;dgaa756. 10.1210/clinem/dgaa756. 10.1210/clinem/dgaa756 33103722

[219] Tripto-Shkolnik L, Fund N, Rouach V, Chodick G, Shalev V, Goldshtein I (2020). Fracture incidence after denosumab discontinuation: real-world data from a large healthcare provider. Bone..

[220] de Bekker-Grob EW, Essink-Bot ML, Meerding WJ, Pols HA, Koes BW, Steyerberg EW (2008). Patients’ preferences for osteoporosis drug treatment: a discrete choice experiment. Osteoporos Int..

[221] Fuzzell LN, Fraenkel L, Stark SL, Seehra SS, Nelson C, Keleman A (2020). A mixed methods study exploring older womens’ attitudes toward osteoporosis medications: adapting a health communication framework. Womens Health Rep (New Rochelle, NY)..

[222] Hudson B, Zarifeb A, Young L, Wells J (2012). Patients’ expectations of screening and preventive treatments. Ann Fam Med..

[223] Neuner JM, Schapira MM (2014). Patient perceptions of osteoporosis treatment thresholds. J Rheumatol..

[224] Si L, Tu L, Xie Y, Palmer AJ, Gu Y, Zheng X (2019). Chinese patients’ preference for pharmaceutical treatments of osteoporosis: a discrete choice experiment. Arch Osteoporos..

[225] LeBlanc A, Wang AT, Wyatt K, Branda ME, Shah ND, Van Houten H (2015). Encounter decision aid vs. clinical decision support or usual care to support patient-centered treatment decisions in osteoporosis: The Osteoporosis Choice Randomized Trial II. PLoS One..

[226] Montori VM, Shah ND, Pencille LJ, Branda ME, Van Houten HK, Swiglo BA (2011). Use of a decision aid to improve treatment decisions in osteoporosis: the osteoporosis choice randomized trial. Am J Med..

[227] Smallwood AJ, Schapira MM, Fedders M, Neuner JM (2017). A pilot randomized controlled trial of a decision aid with tailored fracture risk tool delivered via a patient portal. Osteoporos Int..

[228] Liu CS, Feasel AL, Kline GA, Billington EO (2021). Pharmacotherapy decisions among postmenopausal women attending a group medical consultation or a one-on-one specialist consultation at an osteoporosis center: an observational cohort study. Osteoporos Int..

[229] Hudson B, Toop L, Mangin D, Pearson J (2011). Risk communication methods in hip fracture prevention: a randomised trial in primary care. Br J Gen Pract..

[230] Kalluru R, Petrie KJ, Grey A, Nisa Z, Horne AM, Gamble GD (2017). Randomised trial assessing the impact of framing of fracture risk and osteoporosis treatment benefits in patients undergoing bone densitometry. BMJ Open..

[231] Sheridan SL, Sutkowi-Hemstreet A, Barclay C, Brewer NT, Dolor RJ, Gizlice Z (2016). A comparative effectiveness trial of alternate formats for presenting benefits and harms information for low-value screening services: a randomized clinical trial. JAMA Intern Med..

[232] Rothmann MJ, Möller S, Holmberg T, Højberg M, Gram J, Bech M (2017). Non-participation in systematic screening for osteoporosis—the ROSE trial. Osteoporos Int..

[233] Rothmann MJ, Huniche L, Ammentorp J, Barkmann R, Glüer CC, Hermann AP (2014). Women’s perspectives and experiences on screening for osteoporosis (Risk-stratified Osteoporosis Strategy Evaluation, ROSE). Arch Osteoporos..

[234] Jha S, Wang Z, Laucis N, Bhattacharyya T (2015). Trends in media reports, oral bisphosphonate prescriptions, and hip fractures 1996-2012: an ecological analysis. J Bone Miner Res..

[235] Deutekom M, Vansenne F, McCaffery K, Essink-Bot M-L, Stronks K, Bossuyt PMM (2011). The effects of screening on health behaviour: a summary of the results of randomized controlled trials. J Public Health..

[236] Condurache CI, Chiu S, Chotiyarnwong P, Johansson H, Shepstone L, Lenaghan E (2020). Screening for high hip fracture risk does not impact on falls risk: a post hoc analysis from the SCOOP study. Osteoporos Int..

[237] Masud T, Binkley N, Boonen S, Hannan MT (2011). Official Positions for FRAX® clinical regarding falls and frailty: can falls and frailty be used in FRAX®? From Joint Official Positions Development Conference of the International Society for Clinical Densitometry and International Osteoporosis Foundation on FRAX®. J Clin Densitometry.

[238] Kanis JA, McCloskey E, Johansson H, Oden A, Leslie WD (2012). FRAX® with and without Bone Mineral Density. Calcif Tissue Int..

[239] Kanis JA, Borgstrom F, Zethraeus N, Johnell O, Oden A, Jönsson B (2005). Intervention thresholds for osteoporosis in the UK. Bone..

[240] Leslie WD, Morin SN, Lix LM, McCloskey EV, Johansson H, Harvey NC (2021). Fracture prediction from FRAX for Canadian ethnic groups: a registry-based cohort study. Osteoporos Int..

[241] Wells GA, Hsieh SC, Zheng C, Peterson J, Tugwell P, Liu W (2022). Risedronate for the primary and secondary prevention of osteoporotic fractures in postmenopausal women. Cochrane Database Syst Rev..

[242] Parkkari J, Kannus P, Palvanen M, Natri A, Vainio J, Aho H (1999). Majority of hip fractures occur as a result of a fall and impact on the greater trochanter of the femur: a prospective controlled hip fracture study with 206 consecutive patients. Calcified tissue international..

[243] Symonds C, Kline G (2018). Warning of an increased risk of vertebral fracture after stopping denosumab. CMAJ..

[244] Cummings SR, Ferrari S, Eastell R, Gilchrist N, Jensen JB, McClung M (2018). Vertebral fractures after discontinuation of denosumab: a post hoc analysis of the randomized placebo-controlled FREEDOM trial and its extension. J Bone Miner Res..

[245] Yeam CT, Chia S, Tan HCC, Kwan YH, Fong W, Seng JJB (2018). A systematic review of factors affecting medication adherence among patients with osteoporosis. Osteoporos Int..

[246] Wilton-Clark MS, Feasel AL, Kline GA, Billington EO (2020). Autonomy begets adherence: decisions to start and persist with osteoporosis treatment after group medical consultation. Arch Osteoporos..

[247] Morrison A, Polisena J, Husereau D, Moulton K, Clark M, Fiander M (2012). The effect of English-language restriction on systematic review-based meta-analyses: a systematic review of empirical studies. Int J Technol Assess Health Care..

[248] Waffenschmidt S, Knelangen M, Sieben W, Bühn S, Pieper D (2019). Single screening versus conventional double screening for study selection in systematic reviews: a methodological systematic review. BMC Med Res Methodol..

