# Supplementary material for: Screening for the primary prevention of fragility fractures among adults aged 40 years and older in primary care: systematic reviews of the effects and acceptability of screening and treatment, and the accuracy of risk prediction tools
Source: Syst Rev. 2023 Mar 21;12:51. doi: 10.1186/s13643-023-02181-w (PMC10029308; doi:10.1186/s13643-023-02181-w)
Supplement: Supplementary file 2 — Additional file 2. Search strategy for KQ3b. This file shows the full search strategy for key question 3b on the harms of pharmacologic treatment. [file 13643_2023_2181_MOESM2_ESM.pdf]

Screening for the primary prevention of fragility fractures among adults aged 40 years and older in primary care: systematic reviews of the effects and acceptability of screening and treatment, and the accuracy of risk prediction tools

Authors: Michelle Gates, Jennifer Pillay (corresponding), Megan Nuspl, Aireen Wingert, Ben Vandermeer, Lisa Hartling
Affiliation: Alberta Research Centre for Health Evidence, Department of Pediatrics, University of Alberta. Edmonton Clinic Health Academy, 11405-87 Avenue NW, Edmonton, Alberta, Canada T6G 1C9. E-mail: MG: michellegates85@gmail.com; JP: jpillay@ualberta.ca; MN: megan.nuspl@ualberta.ca; AW: awingert@ualberta.ca; BV: ben.vandermeer@ualberta.ca; LH: hartling@ualberta.ca

Additional file 2. Search strategies for KQ3b on the harms of pharmacologic treatment, from (i) systematic reviews and (ii) primary studies on rebound fractures after discontinuation of denosumab

Systematic reviews

Database: Ovid MEDLINE(R) and Epub Ahead of Print, In-Process & Other Non-Indexed Citations and Daily 1946 to June 23, 2020
Date: 24 June 2020
Search Strategy:

#	Searches	Results
1	Alendronate/	3662
2	Bone Density Conservation Agents/	13808
3	Denosumab/	1620
4	Diphosphonates/	15951
5	Osteoporosis/dt [Drug Therapy]	9420
6	Osteoporosis, Postmenopausal/dt [Drug Therapy]	4591
7	Osteoporotic Fractures/pc [Prevention and control]	1556
8	Risedronate Sodium/	1182
9	alendron*.mp.	5505
10	bisphosphonate*.mp.	17442
11	denosumab*.mp.	3061
12	diphosphonate*.mp.	19383
13	risedron*.mp.	1995
14	zoledron*.mp.	5328
15	or/1-14 [Combined MeSH & text words for osteoporosis treatments]	43682
16	ae.fs. [adverse effects free-floating subheading]	1727258
17	ci.fs. [chemically induced free-floating subheading]	585834
18	co.fs. [complications free-floating subheading]	1968777
19	to.fs. [toxicity free-floating subheading]	423665
20	Risk/	122023
21	(adrs or safe or safety or side effect* or tolerability or toxicity or treatment emergent or undesirable effect*).ti,ab.	1323033
22	(adverse adj2 (effect or effects or event or events or outcome or outcomes or reaction or reactions)).ti,ab.	430669
23	harm*.ti,ab.	180450

24	or/16-23 [Combined free-floating subheadings, MeSH & text words for general harms]	5246296
25	Arrhythmias, Cardiac/ep, et, ci [Epidemiology, Etiology, Chemically Induced]	23995
26	Atrial Fibrillation/ep, et, ci [Epidemiology, Etiology, Chemically Induced]	12955
27	Cardiovascular Diseases/ep, et, ci [Epidemiology, Etiology, Chemically Induced]	65185
28	Colorectal Neoplasms/ep, et, ci [Epidemiology, Etiology, Chemically Induced]	11238
29	Esophageal Neoplasms/ep, et, ci [Epidemiology, Etiology, Chemically Induced]	6420
30	Femoral Fractures/ep, et, ci [Epidemiology, Etiology, Chemically Induced]	2941
31	Gastrointestinal Diseases/ep, et, ci [Epidemiology, Etiology, Chemically Induced]	13291
32	Jaw Diseases/ep, et, ci [Epidemiology, Etiology, Chemically Induced]	1576
33	Osteonecrosis/ep, et, ci [Epidemiology, Etiology, Chemically Induced]	3713
34	Musculoskeletal Pain/ep, et, ci [Epidemiology, Etiology, Chemically Induced]	1666
35	exp Myocardial Ischemia/ep, et, ci [Epidemiology, Etiology, Chemically Induced]	96261
36	Stomach Neoplasms/ep, et, ci [Epidemiology, Etiology, Chemically Induced]	10650
37	Stroke/ep, et, ci [Epidemiology, Etiology, Chemically Induced]	35319
38	arrhythmia*.ti,ab.	84361
39	atrial fib*.ti,ab.	69770
40	((cancer* or neoplasm*) adj3 (colon or colorectal* or esoph* or gastro* or rectal* or rectum or stomach)).ti,ab.	208699
41	(cardiovascular adj2 (death* or disease* or event*)).ti,ab.	205474
42	((femoral* or femur*) adj2 fracture*).ti,ab.	17644
43	((infarct* or ischemi*) adj1 myocardial).ti,ab.	214966
44	((jaw or mandib*) adj3 (osteo-necro* or osteonecro*)).ti,ab.	2517
45	rash*.ti,ab.	30958
46	stroke*.ti,ab.	245634
47	or/25-46 [Combined MeSH & text words for established drug complications]	1092790
48	or/24,47 [Combination of general & specific harms concepts]	5913572
49	and/15,48 [Combined concepts for osteoporosis treatments and harms]	20165
50	Bisphosphonate-Associated Osteonecrosis of the Jaw/	1202
51	49 or 50 [Combined coordinated concept & combined concepts for treatments and harms]	20272
52	limit 51 to systematic reviews	417
53	animals/ not humans/	4676988
54	52 not 53 [Exclude animal studies]	416
55	limit 54 to (english or french)	411
56	(2015* or 2016* or 2017* or 2018* or 2019* or 2020*).dt.	6741761
57	55 and 56	200
58	limit 55 to yr="2015-Current"	205
59	57 or 58 [Date range limit applied]	207
60	remove duplicates from 59	206

Database: Wiley Cochrane Library (inception to present)
Date: 24 June 2020
Search strategy:

ID	Search	Hits
#1	[mh ^Alendronate]	732
#2	[mh ^"Bone Density Conservation Agents"]	1548

#3	[mh ^Denosumab]	304
#4	[mh ^Diphosphonates]	1231
#5	[mh ^Osteoporosis/DT]	573
#6	[mh ^"Osteoporosis, Postmenopausal"/DT]	1075
#7	[mh ^"Osteoporotic Fractures"/PC]	147
#8	[mh ^"Risedronate Sodium"]	250
#9	alendron*:ti,ab,kw	1546
#10	bisphosphonate*:ti,ab,kw	2257
#11	denosumab*:ti,ab,kw	928
#12	diphosphonate*:ti,ab,kw	1385
#13	risedron*:ti,ab,kw	719
#14	zoledron*:ti,ab,kw	1590
#15	{or #1-#14}	6907
#16	MeSH descriptor: [] explode all trees and with qualifier(s): [adverse effects - AE]	127621
#17	MeSH descriptor: [] explode all trees and with qualifier(s): [chemically induced - CI]	23580
#18	MeSH descriptor: [] explode all trees and with qualifier(s): [complications - CO]	52939
#19	MeSH descriptor: [] explode all trees and with qualifier(s): [toxicity - TO]	1629
#20	[mh ^Risk]	3237
#21	(adrs or safe or safety or "side effect*" or tolerability or toxicity or treatment emergent or "undesirable effect*"):ti,ab,kw	336391
#22	(adverse near/2 (effect or effects or event or events or outcome or outcomes or reaction or reactions)):ti,ab,kw	275950
#23	harm*:ti,ab,kw	13601
#24	{or #16-#23}	508121
#25	[mh ^"Arrhythmias, Cardiac"/EP,ET,CI]	866
#26	[mh ^"Atrial Fibrillation"/EP,ET,CI]	729
#27	[mh ^"Cardiovascular Diseases"/EP,ET,CI]	2884
#28	[mh ^"Colorectal Neoplasms"/EP,ET,CI]	272
#29	[mh ^"Esophageal Neoplasms"/EP,ET,CI]	43
#30	[mh ^"Femoral Fractures"/EP,ET,CI]	22
#31	[mh ^"Gastrointestinal Diseases"/EP,ET,CI]	896
#32	[mh ^"Jaw Diseases"/EP,ET,CI]	14
#33	[mh ^Osteonecrosis/EP,ET,CI]	44
#34	[mh ^"Musculoskeletal Pain"/EP,ET,CI]	110
#35	[mh ^"Stomach Neoplasms"/EP,ET,CI]	74
#36	[mh ^Stroke/EP,ET,CI]	1669
#37	arrhythmia*:ti,ab	7799
#38	"atrial fib*":ti,ab	4
#39	((cancer* or neoplasm*) near/3 (colon or colorectal* or esoph* or gastro* or rectal* or rectum or stomach)):ti,ab	20623
#40	(cardiovascular near/2 (death* or disease* or event*)):ti,ab	29814
#41	((femoral* or femur*) near/2 fracture*):ti,ab	1724
#42	((infarct* or ischemi*) near/1 myocardial):ti,ab	29742
#43	((jaw or mandib*) near/3 ("osteo-necro*" or osteonecro*)):ti,ab	229
#44	rash*:ti,ab	4850
#45	stroke*:ti,ab	49759
#46	{or #25-#45}	129492
#47	#24 or #46	586183
#48	#15 and #47	3654

#49	[mh ^"Bisphosphonate-Associated Osteonecrosis of the Jaw"]	21
#50	#48 or #49	3654
#51	#48 or #49 with Publication Year from 2015 to 2020, with Cochrane Library publication date Between Jan 2015 and Jul 2020	19 (17 reviews / 2 protocols)

Other Source: PROSPERO
Platform/URL: https://www.crd.york.ac.uk/prospero/
Date: 24 June 2020
Strategy:

(alendron* OR denosumab* OR disphosphonate* OR risedron* OR zolendron*) AND ("adverse effect*" OR "adverse event*" OR "adverse outcome*" OR complicat* OR harm* OR risk* OR safe OR safety) (112)

Other Source: EPISTEMONIKOS
Platform/URL: https://www.epistemonikos.org
Date: 24 June 2020
Strategy:

(alendron* OR denosumab* OR disphosphonate* OR risedron* OR zolendron*) AND ("adverse effect*" OR "adverse event*" OR "adverse outcome*" OR complicat* OR harm* OR risk* OR safe OR safety)<br>Filters:<br>Publication year: 2015 to 2020<br>Publication type: Systematic Review (163)

Primary studies on rebound fractures from discontinuation of denosumab

Ovid MEDLINE(R) ALL <1946 to June 18, 2021>

#	Searches	Results
1	Bone Density Conservation Agents/	14670
2	Denosumab/ or 4EQZ6YO2HI.rn.	1859
3	(amg162 or amg-162).tw,kf.	38
4	denosumab.tw,kf.	3273
5	dmab.tw,kf.	471
6	prolia.tw,kf.	52
7	xgeva.tw,kf.	28
8	or/1-7	17000
9	Deprescriptions/	619

10	Drug Administration Schedule/	101965
11	Withholding Treatment/	12286
12	ceas*.tw,kf.	25171
13	cessation.tw,kf.	78183
14	(deprescri* or de-prescri*).tw,kf.	1345
15	(discontinu* or dis-continu*).tw,kf.	134782
16	holiday*.tw,kf.	5762
17	stop*.tw,kf.	140187
18	withdraw*.tw,kf.	132161
19	withhold*.tw,kf.	8361
20	or/9-19	588245
21	8 and 20	1867
22	limit 21 to (comment or editorial or letter)	71
23	21 not 22	1796
24	limit 23 to yr="2020 -Current"	182
25	limit 24 to (english or french)	178
26	remove duplicates from 25	177

Embase <1974 to 2021 June 18>

#	Searches	Results
1	bone density conservation agent/	3732
2	denosumab/ or 615258-40-7.rn.	10081
3	(amg162 or amg-162).tw,kw.	248
4	denosumab.tw,kw.	6396
5	dmab.tw,kw.	892

6	prolia.tw,kw.	428
7	xgeva.tw,kw.	298
8	or/1-7	14364
9	deprescription/	635
10	drug withdrawal/	212735
11	treatment withdrawal/	20569
12	ceas*.tw,kw.	32385
13	cessation.tw,kw.	105493
14	(deprescri* or de-prescri*).tw,kw.	1857
15	(discontinu* or dis-continu*).tw,kw.	221242
16	holiday*.tw,kw.	8604
17	stop*.tw,kw.	211219
18	withdraw*.tw,kw.	186305
19	withhold*.tw,kw.	11157
20	or/9-19	853036
21	8 and 20	1879
22	limit 21 to (conference abstract or conference paper or "conference review" or editorial or letter or note)	664
23	21 not 22	1215
24	limit 23 to yr="2020 -Current"	239
25	limit 24 to (english or french)	235
26	remove duplicates from 25	231

Strategy: Cochrane

ID	SEARCH	HITS
#1	(amg162 or amg-162):ti,ab,kw	54
#2	denosumab:ti,ab,kw	1074

#3	dmab:ti,ab,kw	142
#4	prolia:ti,ab,kw	55
#5	xgeva:ti,ab,kw	26
#6	{OR #1-#5}	1089
#7	ceas*:ti,ab,kw	1962
#8	cessation:ti,ab,kw	17443
#9	(deprescri* or de-prescri*):ti,ab,kw	270
#10	(discontinu* or dis-continu*):ti,ab,kw	39138
#11	holiday*:ti,ab,kw	547
#12	stop*:ti,ab,kw	25095
#13	withdraw*:ti,ab,kw	44772
#14	withhold*:ti,ab,kw	1255
#15	{OR #7-#14}	110888
#16	#6 AND #15	172
#17	#6 and #15 with Publication Year from 2020 to present, in Trials	18
#18	#6 and #15 with Cochrane Library publication date from Jan 2020 to present	35
#19	#17 OR #18	35
