# Supplementary material for: Screening for the primary prevention of fragility fractures among adults aged 40 years and older in primary care: systematic reviews of the effects and acceptability of screening and treatment, and the accuracy of risk prediction tools
Source: Syst Rev. 2023 Mar 21;12:51. doi: 10.1186/s13643-023-02181-w (PMC10029308; doi:10.1186/s13643-023-02181-w)
Supplement: Supplementary file 3 — Additional file 3. Evidence sets for KQ1a-b. GRADE Evidence Profiles and Summary of Findings for KQ1. This file shows the analyses and GRADE Evidence Profiles, Summary of Findings, and contributing data for key question 1a on the benefits and harms of screening, and key question 1b on the comparative benefits and harms of different screening approaches. [file 13643_2023_2181_MOESM3_ESM.pdf]

Screening for the primary prevention of fragility fractures among adults aged 40 years and older in primary care: systematic reviews of the effects and acceptability of screening and treatment, and the accuracy of risk prediction tools

Authors: Michelle Gates, Jennifer Pillay (corresponding), Megan Nuspl, Aireen Wingert, Ben Vandermeer, Lisa Hartling

Affiliation: Alberta Research Centre for Health Evidence, Department of Pediatrics, University of Alberta. Edmonton Clinic Health Academy, 11405-87 Avenue NW, Edmonton, Alberta, Canada T6G 1C9. E-mail: MG: michellegates85@gmail.com; JP: jpillay@ualberta.ca; MN: megan.nuspl@ualberta.ca; AW: awingert@ualberta.ca; BV: ben.vandermeer@ualberta.ca; LH: hartling@ualberta.ca

Additional file 3. GRADE Evidence Profiles and Summary of Findings for KQ1a (benefits and harms of screening vs. no screening) and KQ1b (comparative benefits and harms of different screening approaches)

Contents

Evidence Set	Page
**Evidence set 1:** screening vs. no screening; hip fractures (all eligible/offer-to-screen)	3
**Evidence set 2:** Screening vs. no screening; hip fractures (offer-to-screen in selected populations & acceptors of screening)	8
**Evidence Set 3:** Screening vs. no screening; clinical fragility fractures (all eligible/offer-to-screen)	14
**Evidence Set 4:** Screening vs. no screening; clinical fragility fractures (offer-to-screen in selected populations & acceptors of screening)	19
**Evidence Set 5:** Screening vs. no screening; all-cause mortality (all eligible/offer-to-screen)	24
**Evidence Set 6:** Screening vs. no screening; serious adverse events (offer-to-screen in selected populations)	32
**Evidence Set 7:** Screening vs. no screening; wellbeing outcomes (all eligible/offer-to-screen & offer-to-screen in selected populations)	34
**Calculations for overdiagnosis**	38
**Evidence Set 8:** 1-step vs. 2-step screening; hip fractures (all eligible/offer-to-screen)	39
**Evidence Set 9:** 1-step vs. 2-step screening; hip fractures (acceptors of screening)	42
**Evidence Set 10:** 1-step vs. 2-step screening; clinical fragility fractures (all eligible/offer-to-screen)	45
**Evidence Set 11:** 1-step vs. 2-step screening; clinical fragility fractures (acceptors of screening)	48
**Evidence Set 12:** BMD vs. SCORE tool + BMD; hip fractures (all eligible/offer-to-screen)	51

Evidence Set 13: BMD vs. SCORE tool + BMD; hip fractures (acceptors of screening) 54

Evidence Set 14: BMD vs. SCORE tool + BMD; clinical fragility fractures (all eligible/offer-to-screen) 57

Evidence Set 15: BMD vs. SCORE tool + BMD; clinical fragility fractures (acceptors of screening) 60

Evidence Set 16: BMD vs. SOF-based tool + BMD; hip fractures (all eligible/offer-to-screen) 63

Evidence Set 17: BMD vs. SOF-based tool + BMD; hip fractures (acceptors of screening) 66

Evidence Set 18: BMD vs. SOF-based tool + BMD; clinical fragility fractures (all eligible/offer-to-screen) 69

Evidence Set 19: BMD vs. SOF-based tool + BMD; clinical fragility fractures (acceptors of screening) 72

Evidence Set 20: SCORE tool + BMD vs. SOF-based tool + BMD; hip fractures (all eligible/offer-to-screen) 75

Evidence Set 21: SCORE tool + BMD vs. SOF-based tool + BMD; hip fractures (acceptors of screening) 78

Evidence Set 22: SCORE tool + BMD vs. SOF-based tool + BMD; clinical fragility fractures (all eligible/offer-to-screen) 81

Evidence Set 23: SCORE tool + BMD vs. SOF-based tool + BMD; clinical fragility fractures (acceptors of screening) 84

Evidence Set 1: Screening vs. no screening; hip fractures (All eligible/Offer-to-screen)

Included studies: Barr 2010 - APOSS (women 45-54 years), Rubin 2018 - ROSE (women 68-80 years)

1A. GRADE Evidence Profile Table

Population	Certainty assessment							№ of patients		Effect		Certainty*
	№ of studies	Study design	Risk of bias	Inconsistency	Indirectness	Imprecision	Other considerations	Screen-ing	Usual care	Relative HR (95% CI)	Absolute (95% CI)	
**Women 45-54 years of age (Barr 2010 - APOSS)**												
**Control event rate (0.2% or 2 per 1000)**	1	RCT	-1[a]	-0.5[b]	-1[c]	-1[d]	NC	1433	1364	0.95 (0.19 to 4.71)	0.1 fewer in 1000 (1.6 fewer to 7.4 more)	VERY LOW ⨁⊖⊖⊖
**General population risk (0.8% or 8 per 1000)**[**]	1	RCT	-1[a]	-0.5[b]	-1[e]	-1[d]	NC	1433	1364	0.95 (0.19 to 4.71)	0.4 fewer in 1000 (6.5 fewer to 29.7 more)	VERY LOW ⨁⊖⊖⊖
**Women 68-80 years of age (Rubin 2018 - ROSE)**												
**Control event rate (3.5% or 35 per 1000)**	1	RCT	-0.5[f]	-0.5[b]	-1[g]	NC	NC	17072	17157	0.99 (0.88 to 1.11)	0.3 fewer in 1000 (4.2 fewer to 3.9 more)	LOW ⨁⨁⊖⊖
**General population risk (2.0% or 20 per 1000)**[**]	1	RCT	-0.5[f]	-0.5[b]	-1[h]	NC	NC	17072	17157	0.99 (0.88 to 1.11)	0.2 fewer in 1000 (2.4 fewer to 2.2 more)	LOW ⨁⨁⊖⊖

CI: confidence interval; HR: hazard ratio; NC: no serious concerns; RCT: randomized controlled trial

[*] When our assessment of the certainty of evidence fell between levels, we assigned the level that best represented our actual certainty in the evidence, and based our conclusions on this the level of certainty.

[**] The effects without screening for the general risk population are estimated from PRIOR et al., based on 10-year follow-up [1]

Explanations:

[a] Risk of bias: Serious concerns about contamination of the control group (21.6% were taking anti-osteoporosis medications, not including hormone replacement therapy, at follow-up vs. 36.6% in the screened group) and attrition bias due to a high proportion of losses to follow-up (42%).

[b] Inconsistency: Some concern about lack of evidence of consistency because there is only one study in the analysis.

Evidence Set 1: Screening vs. no screening; hip fractures (All eligible/Offer-to-screen)
Included studies: Barr 2010 - APOSS (women 45-54 years), Rubin 2018 - ROSE (women 68-80 years)

[c] Indirectness: Serious concern that the population differs from the population of interest because a relatively large proportion of participants may have been taking anti-osteoporosis drugs at baseline. The comparator differs from that of interest (no screening), because it includes ad-hoc screening. Recruitment method not typical (via letters) but effect on the outcome may be limited.
[d] Imprecision: The total number of events does not meet the optimal information size (<300).
[e] Indirectness: Serious concern that the population differs from the population of interest because a relatively large proportion of participants may have been taking anti-osteoporosis drugs at baseline. The comparator differs from that of interest (no screening), because it includes ad-hoc screening. Recruitment method not typical (via letters) but effect on the outcome may be limited. There is uncertainty about whether the relative HR can be applied across populations with differing levels of baseline risk, however the baseline risk is similar enough to not rate down further.
[f] Risk of bias: Serious concern about contamination of the control group. 25% of the control group had a DXA scan after the index date vs. 48% in the screening group. Use of anti-osteoporosis medications was 18% in the control group vs. 23% in the screening group. Rated down 0.5 because these concerns overlap with concerns about indirectness, for which we have already rated down.
[g] Indirectness: Serious concern that the population differs from the population of interest because 11% of participants who returned complete questionnaires were on osteoporotic treatment at baseline. The comparator differs from that of interest (no screening), because it includes ad-hoc screening. Recruitment method (via letters) and educational materials for GPs not typical but effect on the outcome may be limited.
[h] Indirectness: Serious concern that the population differs from the population of interest because 11% of participants who returned complete questionnaires were on osteoporotic treatment at baseline. The comparator differs from that of interest (no screening), because it includes ad-hoc screening. Recruitment method (via letters) and educational materials for GPs not typical but effect on the outcome may be limited. There is uncertainty about whether the relative HR can be applied across populations with differing levels of baseline risk, however the baseline risk is similar enough to not rate down further.

Evidence Set 1: Screening vs. no screening; hip fractures (All eligible/Offer-to-screen)
Included studies: Barr 2010 - APOSS (women 45-54 years), Rubin 2018 - ROSE (women 68-80 years)

1B. GRADE Summary of Findings Table

Outcome No. participants (studies)	Relative effects, HR (95% CI)	Anticipated absolute effects* (95% CI)			Certainty of evidence (GRADE)**	What happens?
		Without screening	With screening	Difference		
**Women 45-54 years of age (Barr 2010 – APOSS)**						
Hip fractures<br><br>Follow-up: 9 years<br><br>2,979 (1 RCT)	0.95 (0.19 to 4.71)	Control event rate			VERY LOW<br>⊕⊖⊖⊖<br>(control event rate)[a-d] due to risk of bias, inconsistency, indirectness, and imprecision	The evidence about the effects on hip fractures from offering screening to women 45-54 years of age is very uncertain.
		2 per 1000	1.9 per 1000 (0.4 to 9.42)	0.1 fewer in 1000 (1.6 fewer to 7.4 more)		
		General population risk†			VERY LOW<br>⊕⊖⊖⊖<br>(general population risk estimate)[a,b,d,e] due to risk of bias, indirectness, inconsistency, and imprecision	
		8 per 1000	7.6 per 1000 (1.5 to 37.7)	0.4 fewer in 1000 (6.5 fewer to 29.7 more)		
**Women 68-80 years of age (Rubin 2018 – ROSE)**						
Hip fractures<br><br>Follow-up: 5 years<br><br>34,229 (1 RCT)	0.99 (0.88 to 1.11)	Control event rate			LOW<br>⊕⊕⊖⊖<br>(control event rate – high risk)[b,f, g] due to risk of bias, inconsistency, and indirectness	Offering screening to women 68-80 years of age may not reduce the risk of hip fracture compared to no offer of screening, but the evidence is uncertain.
		35 per 1000	34.7 per 1000 (30.8 to 38.9)	0.3 fewer in 1000 (4.2 fewer to 3.9 more)		

Evidence Set 1: Screening vs. no screening; hip fractures (All eligible/Offer-to-screen)
Included studies: Barr 2010 - APOSS (women 45-54 years), Rubin 2018 - ROSE (women 68-80 years)

		General population risk†			LOW ⊕⊕⊖⊖ (general population risk estimate)[b,f,h] due to risk of bias, inconsistency, and indirectness	
		20 per 1000	19.8 per 1000 (17.6 to 22.2)	0.2 fewer in 1000 (2.4 fewer to 2.2 more)		

CI: confidence interval; HR: hazard ratio; RCT: randomized controlled trial
* The absolute effect (and its 95% CI) without screening (i.e. baseline rate) is based on the estimated risk in the comparison group; the effect with screening is based on applying the relative effect of the intervention (and its 95% CI) to the effect without screening.
** When our assessment of the certainty of evidence fell between levels, we assigned the level that best represented our actual certainty in the evidence, and based our conclusions on this the level of certainty.
† The effects without screening for the general risk population are estimated from PRIOR et al., based on 10-year follow-up [1]

Explanations:
[a] Risk of bias: Serious concerns about contamination of the control group (21.6% were taking anti-osteoporosis medications, not including hormone replacement therapy, at follow-up vs. 36.6% in the screened group) and attrition bias due to a high proportion of losses to follow-up (42%).
[b] Inconsistency: Some concern about lack of evidence of consistency because there is only one study in the analysis.
[c] Indirectness: Serious concern that the population differs from the population of interest because a relatively large proportion of participants may have been taking anti-osteoporosis drugs at baseline. The comparator differs from that of interest (no screening), because it includes ad-hoc screening. Recruitment method not typical (via letters) but effect on the outcome may be limited.
[d] Imprecision: The total number of events does not meet the optimal information size (<300).
[e] Indirectness: Serious concern that the population differs from the population of interest because a relatively large proportion of participants may have been taking anti-osteoporosis drugs at baseline. The comparator differs from that of interest (no screening), because it includes ad-hoc screening. Recruitment method not typical (via letters) but effect on the outcome may be limited. There is uncertainty about whether the relative HR can be applied across populations with differing levels of baseline risk, however the baseline risk is similar enough to not rate down further.
[f] Risk of bias: Serious concern about contamination of the control group. 25% of the control group had a DXA scan after the index date vs. 48% in the screening group. Use of anti-osteoporosis medications was 18% in the control group vs. 23% in the screening group. Rated down 0.5 because these concern overlap with concerns about indirectness, for which we have already rated down.
[g] Indirectness: Serious concern that the population differs from the population of interest because 11% of participants who returned complete questionnaires were on osteoporotic treatment at baseline. The comparator differs from that of interest (no screening), because it includes ad-hoc screening. Recruitment method (via letters) and educational materials for GPs not typical but effect on the outcome may be limited.
[h] Indirectness: Serious concern that the population differs from the population of interest because 11% of participants who returned complete questionnaires were on osteoporotic treatment at baseline. The comparator differs from that of interest (no screening), because it includes ad-hoc screening. Recruitment

method (via letters) and educational materials for GPs not typical but effect on the outcome may be limited. There is uncertainty about whether the relative HR can be applied across populations with differing levels of baseline risk, however the baseline risk is similar enough to not rate down further.

1C. Forest Plot

Study or Subgroup	log[Hazard Ratio]	SE	Screening Total	Usual care Total	Weight	Hazard Ratio IV, Random, 95% CI	Risk of Bias A B C D E F G
**1.1.1 Women 45-54 years of age**							
Barr 2010 (APOSS)	-0.04935	0.81561982	1433	1364	100.0%	0.95 [0.19, 4.71]	+ + − ? − ? +
**Subtotal (95% CI)**			**1433**	**1364**	**100.0%**	**0.95 [0.19, 4.71]**	
Heterogeneity: Not applicable							
Test for overall effect: Z = 0.06 (P = 0.95)							
**1.1.2 Women 68-80 years of age**							
Rubin 2018 (ROSE)	-0.01106095	0.06	17072	17157	100.0%	0.99 [0.88, 1.11]	+ + − ? + + +
**Subtotal (95% CI)**			**17072**	**17157**	**100.0%**	**0.99 [0.88, 1.11]**	
Heterogeneity: Not applicable							
Test for overall effect: Z = 0.18 (P = 0.85)							

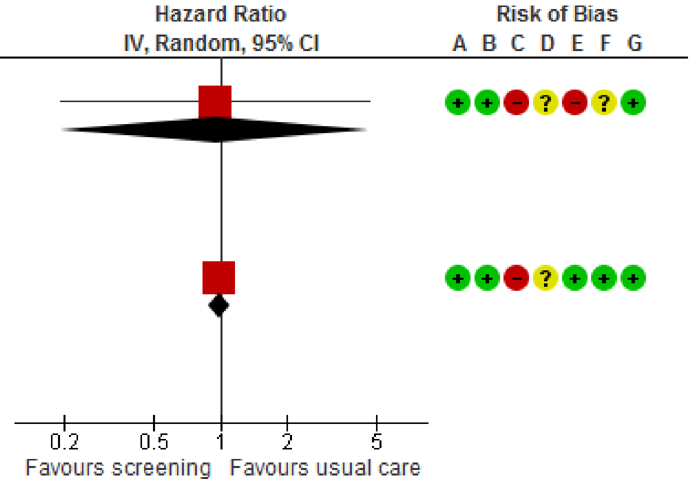

Test for subgroup differences: Chi² = 0.00, df = 1 (P = 0.96), I² = 0%

Risk of bias legend
(A) Random sequence generation (selection bias)
(B) Allocation concealment (selection bias)
(C) Blinding of participants and personnel (performance bias): Fracture outcomes
(D) Blinding of outcome assessment (detection bias): Fracture outcomes
(E) Incomplete outcome data (attrition bias): Fracture outcomes
(F) Selective reporting (reporting bias)
(G) Other bias

* The relative risk was used for Barr 2010 because a hazard ratio was not presented in the study. The hazard ratio in the Rubin 2018 analysis takes into account competing risk of death and handles emigration as a censoring event.

Evidence Set 2: Screening vs. usual care; hip fractures (Offer-to-screen in selected populations & Acceptors of screening)

Included studies: Acceptors: Barr 2010 – APOSS (women 45-64 years); Offer-to-screen in selected: Merlijn 2019 - SALT, Rubin 2018 - ROSE, Shepstone 2018 – SCOOP (women ≥65 years), Kern 2005 [CCT] (men and women ≥70 years)

2A. GRADE Evidence Profile Table

Population	Certainty assessment							№ of patients		Effect		Certainty[*]
	№ of studies	Study design	Risk of bias	Inconsistency	Indirectness	Imprecision	Other consideration	Screen-ing	Usual care	Relative HR (95% CI)	Absolute (95% CI)	
**Women 45-54 years of age who accept screening (Barr 2010 – APOSS)**												
**Control event rate (0.2% or 2 per 1000)**	1	RCT	-1[a]	-0.5[b]	-1[c]	-1[d]	NC	1240	1364	0.37 (0.04 to 3.52)	1.3 fewer per 1000 (1.9 fewer to 5.0 more)	VERY LOW ⨁⊖⊖⊖
**General population risk (0.8% or 8 per 1000)[**]**	1	RCT	-1[a]	-0.5[b]	-1[e]	-1[d]	NC	1240	1364	0.37 (0.04 to 3.52)	5.0 fewer per 1000 (7.7 fewer to 20.2 more)	VERY LOW ⨁⊖⊖⊖
**Women ≥65 years of age (Merlijn 2019 – SALT, Shepstone 2018 – SCOOP, Rubin 2018 – ROSE, Kern 2005 [CCT])**												
**Median control event rate (3.1% or 31 per 1000)**	4	3 RCT, 1 CCT	NC[f]	NC	-0.5[g]	NC	NC	21796	21940	0.80 (0.71 to 0.91)	6.2 fewer per 1000 (9.0 fewer to 2.8 fewer)	MODERATE ⨁⨁⨁⊖
**General population risk (2.0% or 20 per 1000)[**]**	4	3 RCT, 1 CCT	NC[f]	NC	-0.5[h]	NC	NC	21796	21940	0.80 (0.71 to 0.91)	4.0 fewer per 1000 (5.8 fewer to 1.8 fewer)	MODERATE ⨁⨁⨁⊖
**Men ≥70 years of age (Kern 2005 [CCT])** [†]												
**Control event rate (3.0% or 30 per 1000)**	1	CCT	-0.5[i]	-0.5[b]	-0.5[g]	-1[d]	NC	654	726	0.68 (0.32 to 1.43)	9.6 fewer per 1000 (20.4 fewer to 12.9 more)	VERY LOW ⨁⊖⊖⊖
**General population risk (1.6% or 16 per 1000)[**]**	1	CCT	-0.5[i]	-0.5[b]	-0.5[h]	-1[d]	NC	654	726	0.68 (0.32 to 1.43)	5.1 fewer per 1000 (10.9 fewer to 6.9 more)	VERY LOW ⨁⊖⊖⊖

Evidence Set 2: Screening vs. usual care; hip fractures (Offer-to-screen in selected populations & Acceptors of screening)

Included studies: Acceptors: Barr 2010 – APOSS (women 45-64 years); Offer-to-screen in selected: Merlijn 2019 - SALT, Rubin 2018 - ROSE, Shepstone 2018 – SCOOP (women ≥65 years), Kern 2005 [CCT] (men and women ≥70 years)

CCT: clinical controlled trial; CI: confidence interval; HR: hazard ratio; NC: no serious concerns; RCT: randomized controlled trial

* When our assessment of the certainty of evidence fell between levels, we assigned the level that best represented our actual certainty in the evidence, and based our conclusions on this the level of certainty.

** The effects without screening for the general risk population are estimated from PRIOR et al., based on 10 year follow-up [1]

† Started at low certainty due to study design

Explanations:

[a] Risk of bias: Serious concerns about attrition bias due to a high proportion of losses to follow-up (42%), contamination of the control group (21.6% were taking anti-osteoporosis medications, not including hormone replacement therapy, at follow-up vs. 36.6% in the screened group), and selection bias because the analysis is per protocol.

[b] Inconsistency: Some concern about lack of evidence of consistency because there is only one study in the analysis.

[c] Indirectness: Serious concern that the population differs from the population of interest because a relatively large proportion of participants may have been taking anti-osteoporosis drugs at baseline. The comparator differs from that of interest (no screening), because it includes ad-hoc screening. Recruitment method (via letters) not typical but effect on the outcome may be limited.

[d] Imprecision: The total number of events does not meet the optimal information size (<300).

[e] Indirectness: Serious concern that the population differs from the population of interest because a relatively large proportion of participants may have been taking anti-osteoporosis drugs at baseline. The comparator differs from that of interest (no screening), because it includes ad-hoc screening. Recruitment method (via letters) not typical but effect on the outcome may be limited. There is uncertainty about whether the relative HR can be applied across populations with differing levels of baseline risk, however the baseline risk is similar enough to not rate down further.

[f] Risk of bias: Did not rate down because a significant benefit of screening was observed despite concerns (contamination of the control group [Rubin 2018, Shepstone 2018, Merlijn 2019]; selective reporting [Kern 2005]; design [Kern 2005]) that are likely to bias the findings toward the null.

[g] Indirectness: The comparator differs from that of interest (no screening), because it includes ad-hoc screening. Recruitment methods and education of GPs not typical but effect on the outcomes is unclear. For analysis of women ≥65 years, Merlijn 2019 enrolls a high risk population, but removal of this study does not change the findings.

[h] Indirectness: The comparator differs from that of interest (no screening), because it includes ad-hoc screening. Recruitment methods and education of GPs not typical but effect on the outcomes is unclear. For analysis of women ≥65 years, Merlijn 2019 enrolls a high risk population, but removal of this study does not change the findings. There is uncertainty about whether the relative HR can be applied across populations with differing levels of baseline risk, however the baseline risk is similar enough to not rate down further.

[i] Risk of bias: Some concerns about performance and detection bias. Potential for bias related to the hypothesis being generated after data were collected.

Evidence Set 2: Screening vs. usual care; hip fractures (Offer-to-screen in selected populations & Acceptors of screening)

Included studies: Acceptors: Barr 2010 – APOSS (women 45-64 years); Offer-to-screen in selected: Merlijn 2019 - SALT, Rubin 2018 - ROSE, Shepstone 2018 – SCOOP (women ≥65 years), Kern 2005 [CCT] (men and women ≥70 years)

2B. GRADE Summary of Findings Table

Outcome<br>No. participants (studies)	Relative effects, HR (95% CI)	Anticipated absolute effects* (95% CI)			Certainty of evidence (GRADE)**	What happens?
		Without screening	With screening	Difference		
**Women 45-54 years of age (Barr 2010 – APOSS)**						
Hip fractures<br><br>Follow-up: 9 years<br><br>2,604 (1 RCT)	0.37 (0.04 to 3.52)	Control event rate				The evidence about the effects of accepting screening on hip fractures in women 45-54 years of age is very uncertain.
		2 per 1000	0.7 per 1000 (0.1 to 7.0)	1.3 fewer per 1000 (1.9 fewer to 5.0 more)	VERY LOW<br>⊕⊖⊖⊖<br>(control event rate)[a-d] due to risk of bias, inconsistency, indirectness, and imprecision	
		General population risk†				
		8 per 1000	3.0 per 1000 (0.3 to 28.2)	5.0 fewer per 1000 (7.7 fewer to 20.2 more)	VERY LOW<br>⊕⊖⊖⊖<br>(control event rate)[a,b,d,e] due to risk of bias, inconsistency, indirectness, and imprecision	
**Women ≥65 years of age (Merlijn 2019 – SALT, Shepstone 2018 – SCOOP, Rubin 2018 – ROSE, Kern 2005 [CCT])**						
Hip fractures<br><br>Follow-up: 3-5 years<br><br>43,736 (3 RCT, 1CCT)	0.80 (0.71 to 0.91)	Median control event rate				Offering screening probably slightly reduces the risk of hip fracture compared to no offer of screening among selected populations of women ≥65 years of age among which compliance might be higher than the general population.
		31 per 1000	24.8 per 1000 (22.0 to 28.2)	6.2 fewer per 1000 (9.0 fewer to 2.8 fewer)	MODERATE<br>⊕⊕⊕⊖<br>(median control event rate – high risk)[f,g] due to indirectness	
		General population risk †				
		20 per 1000	16.0 per 1000 (14.2 to 18.2)	4.0 fewer (5.8 fewer to 1.8 fewer)	MODERATE<br>⊕⊕⊕⊖<br>(general population risk)[f,h] indirectness	

Evidence Set 2: Screening vs. usual care; hip fractures (Offer-to-screen in selected populations & Acceptors of screening)

Included studies: Acceptors: Barr 2010 – APOSS (women 45-64 years); Offer-to-screen in selected: Merlijn 2019 - SALT, Rubin 2018 - ROSE, Shepstone 2018 – SCOOP (women ≥65 years), Kern 2005 [CCT] (men and women ≥70 years)

Outcome No. participants (studies)	Relative effects, HR (95% CI)	Anticipated absolute effects* (95% CI)			Certainty of evidence (GRADE)**	What happens?
		Without screening	With screening	Difference		
**Men ≥70 years (Kern 2005 [CCT])**						
Hip fractures<br><br>Follow-up: 4.9 years<br><br>1,380 (1 CCT)	0.68 (0.32 to 1.43)	Control event rate			VERY LOW ⊕⊖⊖⊖ (control event rate – high risk)[b,d,g,i] due to risk of bias, inconsistency, indirectness, and imprecision	The evidence about the effects on hip fractures from offering screening to selected populations of men ≥70 years of age is very uncertain.
		30 per 1000	20.4 per 1000 (9.6 to 42.9)	9.6 fewer per 1000 (20.4 fewer to 12.9 more)		
		General population risk †			VERY LOW ⊕⊖⊖⊖ (general population risk)[b,d,h,i] due to risk of bias, inconsistency, indirectness, and imprecision	
		16 per 1000	10.9 per 1000 (5.1 to 22.9)	5.1 fewer per 1000 (10.9 fewer to 6.9 more)		

CCT: clinical controlled trial; CI: confidence interval; HR: hazard ratio; RCT: randomized controlled trial

* The absolute effect (and its 95% CI) without screening (i.e. baseline rate) is based on the estimated risk in the comparison group; the effect with screening is based on applying the relative effect of the intervention (and its 95% CI) to the effect without screening.

** When our assessment of the certainty of evidence fell between levels, we assigned the level that best represented our actual certainty in the evidence, and based our conclusions on this the level of certainty.

† The effects without screening for the general risk population are estimated from PRIOR et al., based on 10 year follow-up [1]

Explanations:

[a] Risk of bias: Serious concerns about attrition bias due to a high proportion of losses to follow-up (42%), contamination of the control group (21.6% were taking anti-osteoporosis medications, not including hormone replacement therapy, at follow-up vs. 36.6% in the screened group), and selection bias because the analysis is per protocol.

[b] Inconsistency: Some concern about lack of evidence of consistency because there is only one study in the analysis.

Evidence Set 2: Screening vs. usual care; hip fractures (Offer-to-screen in selected populations & Acceptors of screening)

Included studies: Acceptors: Barr 2010 – APOSS (women 45-64 years); Offer-to-screen in selected: Merlijn 2019 - SALT, Rubin 2018 - ROSE, Shepstone 2018 – SCOOP (women ≥65 years), Kern 2005 [CCT] (men and women ≥70 years)

[c] Indirectness: Serious concern that the population differs from the population of interest because a relatively large proportion of participants may have been taking anti-osteoporosis drugs at baseline. The comparator differs from that of interest (no screening), because it includes ad-hoc screening. Recruitment method (via letters) not typical but effect on the outcome may be limited.

[d] Imprecision: The total number of events does not meet the optimal information size (<300).

[e] Indirectness: Serious concern that the population differs from the population of interest because a relatively large proportion of participants may have been taking anti-osteoporosis drugs at baseline. The comparator differs from that of interest (no screening), because it includes ad-hoc screening. Recruitment method (via letters) not typical but effect on the outcome may be limited. There is uncertainty about whether the relative HR can be applied across populations with differing levels of baseline risk, however the baseline risk is similar enough to not rate down further.

[f] Risk of bias: Did not rate down because a significant benefit of screening was observed despite concerns (contamination of the control group [Rubin 2018, Shepstone 2018, Merlijn 2019; selective reporting [Kern 2005]; design [Kern 2005]) that are likely to bias the findings toward the null.

[g] Indirectness: The comparator differs from that of interest (no screening), because it includes ad-hoc screening. Recruitment methods and education of GPs not typical but effect on the outcomes is unclear. For analysis of women ≥65 years, Merlijn 2019 enrolls a high risk population, but removal of this study does not change the findings.

[h] Indirectness: The comparator differs from that of interest (no screening), because it includes ad-hoc screening. Recruitment methods and education of GPs not typical but effect on the outcomes is unclear. For analysis of women ≥65 years, Merlijn 2019 enrolls a high risk population, but removal of this study does not change the findings. There is uncertainty about whether the relative HR can be applied across populations with differing levels of baseline risk, however the baseline risk is similar enough to not rate down further.

[i] Risk of bias: Some concerns about performance and detection bias. Potential for bias related to the hypothesis being generated after data were collected.

Evidence Set 2: Screening vs. usual care; hip fractures (Offer-to-screen in selected populations & Acceptors of screening)

Included studies: Acceptors: Barr 2010 – APOSS (women 45-64 years); Offer-to-screen in selected: Merlijn 2019 - SALT, Rubin 2018 - ROSE, Shepstone 2018 – SCOOP (women ≥65 years), Kern 2005 [CCT] (men and women ≥70 years)

2C. Forest Plot

Study or Subgroup	log[Hazard Ratio]	SE	Screening Total	Usual care Total	Weight	Hazard Ratio IV, Random, 95% CI	Risk of Bias A	B	C	D	E	F	G
**1.2.1 Women 45-54 years of age**													
Barr 2010 (APOSS)	-1.0033	1.15403368	1240	1364	100.0%	0.37 [0.04, 3.52]	?	+	-	?	-	?	+
**Subtotal (95% CI)**			**1240**	**1364**	**100.0%**	**0.37 [0.04, 3.52]**							
Heterogeneity: Not applicable													
Test for overall effect: Z = 0.87 (P = 0.38)													
**1.2.2 Women ≥ 65 years of age**													
Kern 2005	-0.49	0.28	768	959	5.0%	0.61 [0.35, 1.06]	?	?	?	?	+	-	+
Merlijn 2019 (SALT)	-0.09431068	0.11946	5516	5405	25.9%	0.91 [0.72, 1.15]	+	+	?	+	+	+	+
Rubin 2018 (ROSE)	-0.17673718	0.1043	9279	9326	33.2%	0.84 [0.68, 1.03]	?	+	-	?	+	+	+
Shepstone 2018 (SCOOP)	-0.32	0.1	6233	6250	35.9%	0.73 [0.60, 0.88]	+	+	-	+	+	+	+
**Subtotal (95% CI)**			**21796**	**21940**	**100.0%**	**0.80 [0.71, 0.91]**							
Heterogeneity: Tau² = 0.00; Chi² = 3.21, df = 3 (P = 0.36); I² = 6%													
Test for overall effect: Z = 3.53 (P = 0.0004)													
**1.2.3 Men ≥ 70 years of age**													
Kern 2005	-0.39	0.38	654	726	100.0%	0.68 [0.32, 1.43]	?	?	?	?	+	-	+
**Subtotal (95% CI)**			**654**	**726**	**100.0%**	**0.68 [0.32, 1.43]**							
Heterogeneity: Not applicable													
Test for overall effect: Z = 1.03 (P = 0.30)													

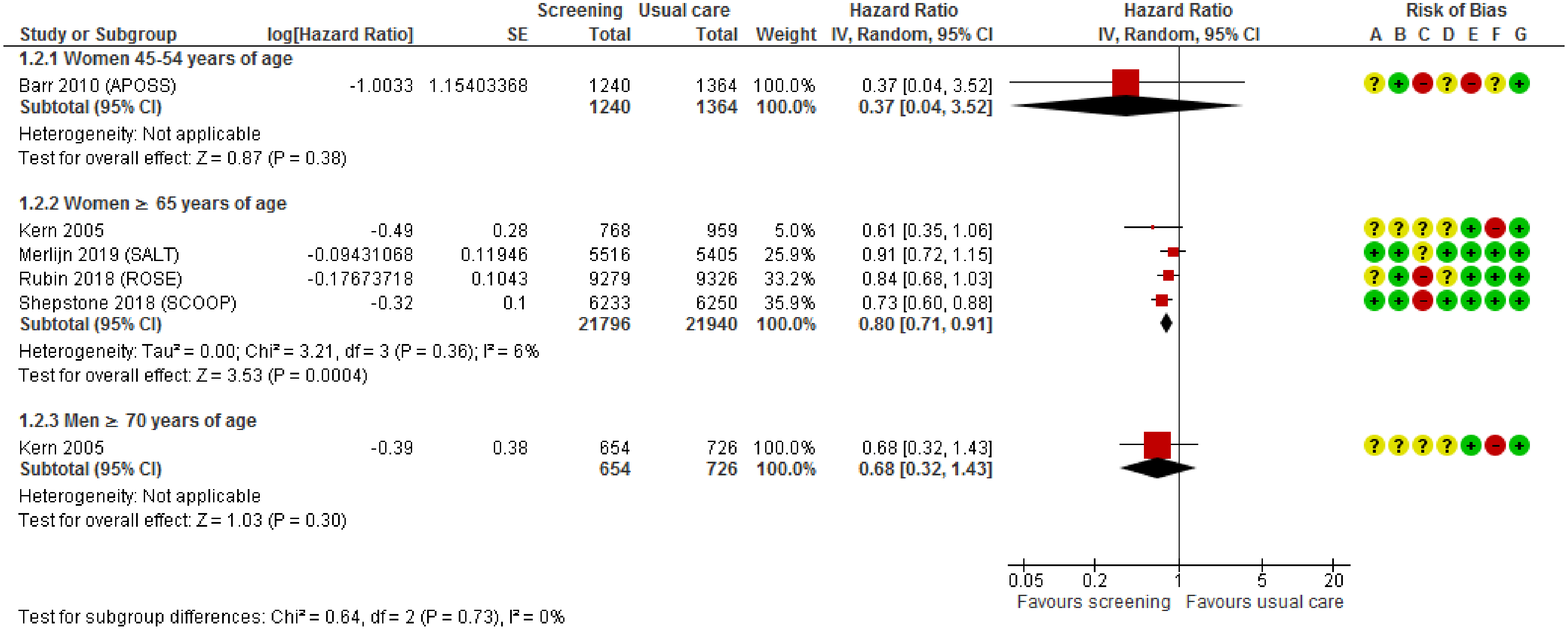

Test for subgroup differences: Chi² = 0.64, df = 2 (P = 0.73), I² = 0%

Risk of bias legend

(A) Random sequence generation (selection bias)
(B) Allocation concealment (selection bias)
(C) Blinding of participants and personnel (performance bias): Fracture outcomes
(D) Blinding of outcome assessment (detection bias): Fracture outcomes
(E) Incomplete outcome data (attrition bias): Fracture outcomes
(F) Selective reporting (reporting bias)
(G) Other bias

* The relative risk was used for Barr 2010 because a hazard ratio was not presented in the study. The hazard ratio in the Rubin 2018 analysis takes into account competing risk of death and handles emigration as a censoring event. The hazard ratio in the Shepstone 2018 analysis regarded death or withdrawal from the study as a censoring event, and included recruiting region, baseline FRAX, and self-reported falls in the model (prognostic factors agreed on before analysis). The Kern 2005 analysis was adjusted for propensity to be screened; we used the adjusted analysis because the study was non-randomized.

Evidence Set 3: Screening vs. usual care; clinical fragility fractures (All eligible for screening/Offer-to-screen)
Included studies: Barr 2010 - APOSS (women 45-54 years), Rubin 2018 – ROSE (women 68-80 years)

3A. GRADE Evidence Profile Table

Population	Certainty assessment							№ of patients		Effect		Certainty*
	№ of studies	Study design	Risk of bias	Inconsistency	Indirectness	Imprecision	Other consideration	Screen-ing	Usual care	Relative HR (95% CI)	Absolute (95% CI)	
**Women 45-54 years of age (Barr 2010 - APOSS)**												
**Control event rate (3.4% or 34 per 1000)**	1	RCT	-1[a]	-0.5[b]	-1[c]	-1[d]	NC	1433	1364	1.01 (0.68 to 1.50)	0.3 more per 1000 (10.9 fewer to 17.0 more)	VERY LOW ⊕⊖⊖⊖
**General population risk (6.7% or 67 per 10000)**[**]	1	RCT	-1[a]	-0.5[b]	-1[e]	-1[d]	NC	1433	1364	1.01 (0.68 to 1.50)	0.7 more per 1000 (21.4 fewer to 33.5 more)	VERY LOW ⊕⊖⊖⊖
**Women ≥65 years (Rubin 2018 - ROSE)**												
**Control event rate (10.0% or 100 per 1000)**	1	RCT	-0.5[f]	-0.5[b]	-1[g]	NC	NC	17072	17157	0.99 (0.92 to 1.06)	1.0 fewer per 1000 (8.0 fewer to 6.0 more)	LOW ⊕⊕⊖⊖
**General population risk (16.8% or 168 per 1000)**[**]	1	RCT	-0.5[f]	-0.5[b]	-1[h]	NC	NC	17072	17157	0.99 (0.92 to 1.06)	1.7 fewer per 1000 (13.4 fewer to 10.1 more)	LOW ⊕⊕⊖⊖

CI: confidence interval; HR: hazard ratio; NC: no serious concerns; RCT: randomized controlled trial

[*] When our assessment of the certainty of evidence fell between levels, we assigned the level that best represented our actual certainty in the evidence, and based our conclusions on this the level of certainty.

[**] The effects without screening for the general risk population are estimated from PRIOR et al., based on 10 year follow-up [1]

Explanations:

[a] Risk of bias: Serious concerns about contamination of the control group (21.6% were taking anti-osteoporosis medications, not including hormone replacement therapy, at follow-up vs. 36.6% in the screened group), and about potential attrition bias due to a high proportion of losses to follow-up (42%).

[b] Inconsistency: Some concern about lack of evidence of consistency because there is only one study in the analysis.

Evidence Set 3: Screening vs. usual care; clinical fragility fractures (All eligible for screening/Offer-to-screen)
Included studies: Barr 2010 - APOSS (women 45-54 years), Rubin 2018 – ROSE (women 68-80 years)

[c] Indirectness: Serious concern that the population differs from the population of interest because a relatively large proportion of participants may have been taking anti-osteoporosis drugs at baseline. The comparator differs from that of interest (no screening), because it includes ad-hoc screening. Recruitment method not typical (via letters) but effect on the outcome may be limited.
[d] Imprecision: The total number of events does not meet the optimal information size (<300).
[e] Indirectness Serious concern that the population differs from the population of interest because a relatively large proportion of participants may have been taking anti-osteoporosis drugs at baseline. The comparator differs from that of interest (no screening), because it includes ad-hoc screening. Recruitment method not typical (via letters) but effect on the outcome may be limited. There is uncertainty about whether the relative HR can be applied across populations with differing levels of baseline risk, however the baseline risk is similar enough to not rate down further.
[f] Risk of bias: Serious concern about contamination of the control group. 25% of the control group had a DXA scan after the index date vs. 48% in the screening group. Use of antiosteoporosis medications was 18% in the control group vs. 23% in the screening group. Rated down 0.5 because these concern overlap with concerns about indirectness, for which we have already rated down.
[g] Indirectness: Serious concern that the population differs from the population of interest because 11% of participants who returned complete questionnaires were on osteoporotic treatment at baseline. The comparator differs from that of interest (no screening), because it includes ad-hoc screening. The comparator differs from that of interest (no screening), because it includes ad-hoc screening. Recruitment method (via letters) and educational materials for GPs not typical but effect on the outcome may be limited.
[h] Indirectness: Serious concern that the population differs from the population of interest because 11% of participants who returned complete questionnaires were on osteoporotic treatment at baseline. The comparator differs from that of interest (no screening), because it includes ad-hoc screening. Recruitment method (via letters) and educational materials for GPs not typical but effect on the outcome may be limited. There is uncertainty about whether the relative HR can be applied across populations with differing levels of baseline risk, however the baseline risk is similar enough to not rate down further.

3B. GRADE Summary of Findings Table

Outcome No. participants (studies)	Relative effects, HR (95% CI)	Anticipated absolute effects* (95% CI)			Certainty of evidence (GRADE)**	What happens?
		Without screening	With screening	Difference		
**Women <65 years (Barr 2010 - APOSS)**						
Major osteoporotic fractures (hip, clinical vertebral, humerus, wrist)<br><br>Follow-up: 9 years	1.01 (0.68 to 1.50)	Control event rate			VERY LOW ⊕⊖⊖⊖ (control event rate)[a-d] due to risk of bias, inconsistency, indirectness, and imprecision	The evidence about the effects on clinical fragility fractures from offering screening to women 45-54 years of age is very uncertain.
		34 per 1000	34.3 per 1000 (23.1 to 51.0)	0.3 more per 1000 (10.9 fewer to 17.0 more)		

Evidence Set 3: Screening vs. usual care; clinical fragility fractures (All eligible for screening/Offer-to-screen)
Included studies: Barr 2010 - APOSS (women 45-54 years), Rubin 2018 – ROSE (women 68-80 years)

2,979 (1 RCT)		General population risk†			VERY LOW ⊕⊝⊝⊝ (control event rate)[a,b,d,e] due to risk of bias, inconsistency, indirectness, and imprecision	
		67 per 1000	67.7 per 1000 (45.6 to 100.5)	0.7 more per 1000 (21.4 fewer to 33.5 more)		
**Women ≥65 years (Rubin 2018 - ROSE)**						
Major osteoporotic fractures (hip, clinical vertebral, humerus, wrist)<br><br>Follow-up: 5 years<br><br>34,229 (1 RCT)	0.99 (0.92 to 1.06)	Control event rate			LOW ⊕⊕⊝⊝ (general population risk estimate)[b,f,g] due to risk of bias, inconsistency, and indirectness	Offering screening to women ≥65 years may not reduce the risk of clinical fragility fracture compared to no offer of screening, but the evidence is uncertain.
		100 per 1000	99.0 per 1000 (92.0 to 106.0)	1.0 fewer per 1000 (8.0 fewer to 6.0 more)		
		General population risk†			LOW ⊕⊕⊝⊝ (general population risk)[b,f,h] due to risk of bias, inconsistency, and indirectness	
		168 per 1000	166.3 per 1000 (154.6 to 178.1)	1.7 fewer per 1000 (13.4 fewer to 10.1 more)		

CCT: clinical controlled trial; CI: confidence interval; HR: hazard ratio; RCT: randomized controlled trial; ROB: risk of bias

*The absolute effect (and its 95% CI) without screening (i.e. baseline rate) is based on the estimated risk in the comparison group; the effect with screening is based on applying the relative effect of the intervention (and its 95% CI) to the effect without screening.

** When our assessment of the certainty of evidence fell between levels, we assigned the level that best represented our actual certainty in the evidence, and based our conclusions on this the level of certainty.

† The effects without screening for the general risk population are estimated from PRIOR et al., based on 10 year follow-up [1]

Explanations:

[a] Risk of bias: Serious concerns about contamination of the control group (21.6% were taking anti-osteoporosis medications, not including hormone replacement therapy, at follow-up vs. 36.6% in the screened group), and about potential attrition bias due to a high proportion of losses to follow-up (42%).

[b] Inconsistency: Some concern about lack of evidence of consistency because there is only one study in the analysis.

Evidence Set 3: Screening vs. usual care; clinical fragility fractures (All eligible for screening/Offer-to-screen)
Included studies: Barr 2010 - APOSS (women 45-54 years), Rubin 2018 – ROSE (women 68-80 years)

[c] Indirectness: Serious concern that the population differs from the population of interest because a relatively large proportion of participants may have been taking anti-osteoporosis drugs at baseline. The comparator differs from that of interest (no screening), because it includes ad-hoc screening. Recruitment method not typical (via letters) but effect on the outcome may be limited.
[d] Imprecision: The total number of events does not meet the optimal information size (<300).
[e] Indirectness Serious concern that the population differs from the population of interest because a relatively large proportion of participants may have been taking anti-osteoporosis drugs at baseline. The comparator differs from that of interest (no screening), because it includes ad-hoc screening. Recruitment method not typical (via letters) but effect on the outcome may be limited. There is uncertainty about whether the relative HR can be applied across populations with differing levels of baseline risk, however the baseline risk is similar enough to not rate down further.
[f] Risk of bias: Serious concern about contamination of the control group. 25% of the control group had a DXA scan after the index date vs. 48% in the screening group. Use of antiosteoporosis medications was 18% in the control group vs. 23% in the screening group. Rated down 0.5 because these concern overlap with concerns about indirectness, for which we have already rated down.
[g] Indirectness: Serious concern that the population differs from the population of interest because 11% of participants who returned complete questionnaires were on osteoporotic treatment at baseline. The comparator differs from that of interest (no screening), because it includes ad-hoc screening. The comparator differs from that of interest (no screening), because it includes ad-hoc screening. Recruitment method (via letters) and educational materials for GPs not typical but effect on the outcome may be limited.
[h] Indirectness: Serious concern that the population differs from the population of interest because 11% of participants who returned complete questionnaires were on osteoporotic treatment at baseline. The comparator differs from that of interest (no screening), because it includes ad-hoc screening. Recruitment method (via letters) and educational materials for GPs not typical but effect on the outcome may be limited. There is uncertainty about whether the relative HR can be applied across populations with differing levels of baseline risk, however the baseline risk is similar enough to not rate down further.

Evidence Set 3: Screening vs. usual care; clinical fragility fractures (All eligible for screening/Offer-to-screen)

Included studies: Barr 2010 - APOSS (women 45-54 years), Rubin 2018 – ROSE (women 68-80 years)

3C. Forest Plot

Study or Subgroup	log[Hazard Ratio]	SE	Screening Total	Usual care Total	Weight	Hazard Ratio IV, Random, 95% CI	A	B	C	D	E	F	G
**1.3.1 Women 45-54 years of age**													
Barr 2010 (APOSS)	0.012527	0.19961368	1433	1364	100.0%	1.01 [0.68, 1.50]	+	+	-	?	-	?	+
**Subtotal (95% CI)**			**1433**	**1364**	**100.0%**	**1.01 [0.68, 1.50]**							
Heterogeneity: Not applicable													
Test for overall effect: Z = 0.06 (P = 0.95)													
**1.3.2 Women 68-80 years of age years**													
Rubin 2018 (ROSE)	-0.01106095	0.03431	17072	17157	100.0%	0.99 [0.92, 1.06]	+	+	-	?	+	+	+
**Subtotal (95% CI)**			**17072**	**17157**	**100.0%**	**0.99 [0.92, 1.06]**							
Heterogeneity: Not applicable													
Test for overall effect: Z = 0.32 (P = 0.75)													

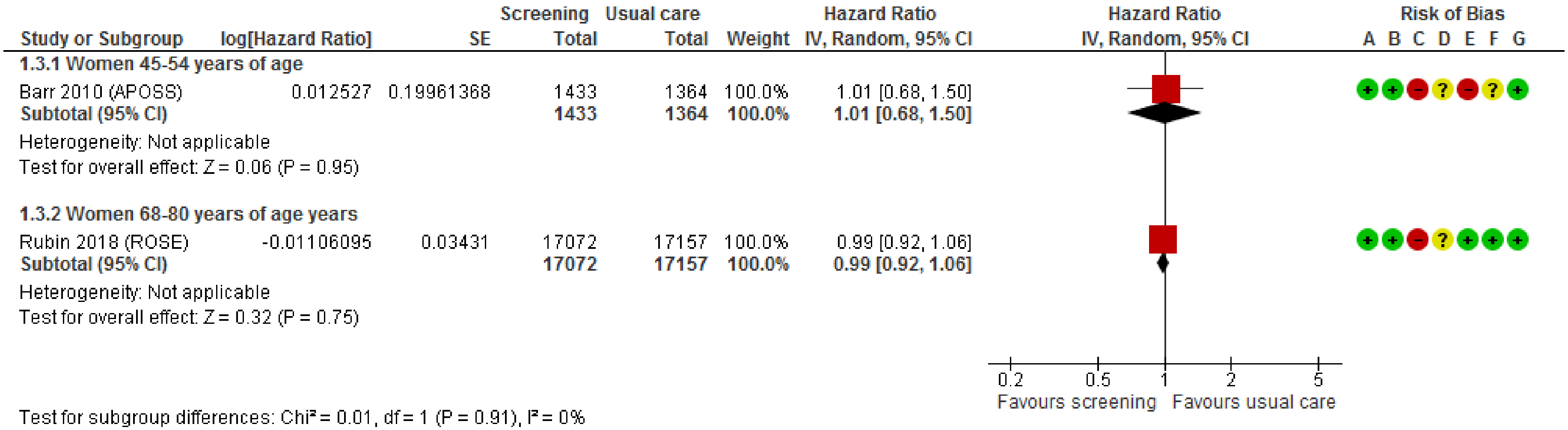

Test for subgroup differences: Chi² = 0.01, df = 1 (P = 0.91), I² = 0%

Risk of bias legend

(A) Random sequence generation (selection bias)
(B) Allocation concealment (selection bias)
(C) Blinding of participants and personnel (performance bias): Fracture outcomes
(D) Blinding of outcome assessment (detection bias): Fracture outcomes
(E) Incomplete outcome data (attrition bias): Fracture outcomes
(F) Selective reporting (reporting bias)
(G) Other bias

* The relative risk was used for Barr 2010 because a hazard ratio was not presented in the study. The hazard ratio in the Rubin 2018 analysis takes into account competing risk of death and handles emigration as a censoring event.

Evidence Set 4: Screening vs. usual care; clinical fragility fractures (Offer-to-screen in selected populations & Acceptors of screening)
Included studies: Acceptors: Barr 2010 – APOSS (women 45-54 years); Offer-to-screen in selected: Merlijn 2019 - SALT, Rubin 2018 - ROSE, Shepstone 2018 – SCOOP (women ≥65 years)

4A. GRADE Evidence Profile Table

Population	Certainty assessment							№ of patients		Effect		Certainty[*]
	№ of studies	Study design	Risk of bias	Inconsistency	Indirectness	Imprecision	Other consideration	Screen-ing	Usual care	Relative HR (95% CI)	Absolute (95% CI)	
**Women 45-54 years of age (Barr 2010 - APOSS)**												
**Control event rate (3.4% or 34 per 1000)**	1	RCT	-1[a]	-0.5[b]	-1[c]	-1[d]	NC	1240	1364	0.73 (0.46 to 1.14)	9.2 fewer per 1000 (18.4 fewer to 4.8 more)	VERY LOW ⊕⊖⊖⊖
**General population risk (6.7% or 67 per 1000)[**]**	1	RCT	-1[a]	-0.5[b]	-1[e]	-1[d]	NC	1240	1364	0.73 (0.46 to 1.14)	18.1 fewer per 1000 (36.2 fewer to 9.4 more)	VERY LOW ⊕⊖⊖⊖
**Women ≥65 years of age (Merlijn 2019 – SALT, Rubin 2018 – ROSE, Shepstone 2018 – SCOOP)**												
**Control event rate (8.4% or 84 per 1000)**	3	RCT	NC[f]	NC	-1[g]	NC	NC	21028	20981	0.93 (0.87 to 0.99)	5.9 fewer per 1000 (10.9 fewer to 0.8 fewer)	MODERATE ⊕⊕⊕⊖
**General population risk (16.8% or 168 per 1000)[**]**	3	RCT	NC[f]	NC	-1[h]	NC	NC	21028	20981	0.93 (0.87 to 0.99)	11.8 fewer per 1000 (21.8 fewer to 1.7 fewer)	MODERATE ⊕⊕⊕⊖

CI: confidence interval; HR: hazard ratio; NC: no serious concerns; RCT: randomized controlled trial

[*] When our assessment of the certainty of evidence fell between levels, we assigned the level that best represented our actual certainty in the evidence, and based our conclusions on this the level of certainty.

[**] The effects without screening for the general risk population are estimated from PRIOR et al., based on 10 year follow-up [1]

Evidence Set 4: Screening vs. usual care; clinical fragility fractures (Offer-to-screen in selected populations & Acceptors of screening)
Included studies: Acceptors: Barr 2010 – APOSS (women 45-54 years); Offer-to-screen in selected: Merlijn 2019 - SALT, Rubin 2018 - ROSE, Shepstone 2018 – SCOOP (women ≥65 years)

Explanations:
[a] Risk of bias: Serious concerns about attrition bias due to a high proportion of losses to follow-up (42%), contamination of the control group (21.6% were taking anti-osteoporosis medications, not including hormone replacement therapy, at follow-up vs. 36.6% in the screened group), and selection bias because the analysis is per protocol.
[b] Inconsistency: Some concern about lack of evidence of consistency because there is only one study in the analysis.
[c] Indirectness: Serious concern that the population differs from the population of interest because a relatively large proportion of participants may have been taking anti-osteoporosis drugs at baseline. The comparator differs from that of interest (no screening), because it includes ad-hoc screening. Recruitment method (via letters) not typical but effect on the outcome may be limited.
[d] Imprecision: The total number of events does not meet the optimal information size (<300).
[e] Indirectness Serious concern that the population differs from the population of interest because a relatively large proportion of participants may have been taking anti-osteoporosis drugs at baseline. The comparator differs from that of interest (no screening), because it includes ad-hoc screening. Recruitment method (via letters) not typical but effect on the outcome may be limited.
[f] Risk of bias: Did not rate down because a significant benefit of screening was observed despite concerns (contamination of the control group [Rubin 2018, Shepstone 2018, Merlijn 2019]) that are likely to bias the findings toward the null.
[g] Indirectness: The comparator differs from that of interest (no screening), because it includes ad-hoc screening. Due to the ascertainment method, the outcome may include an unknown number of non-clinical vertebral fractures. Recruitment methods and education of GPs not typical but effect on the outcomes is unclear. Merlijn 2019 enrolls a high risk population, but removal of this study does not change the findings.
[h] Indirectness: The comparator differs from that of interest (no screening), because it includes ad-hoc screening. Recruitment methods and education of GPs not typical but effect on the outcomes is unclear. Due to the ascertainment method, the outcome may include an unknown number of non-clinical vertebral fractures. Merlijn 2019 enrolls a high risk population, but removal of this study does not change the findings. There is uncertainty about whether the relative HR can be applied across populations with differing levels of baseline risk, however the baseline risk is similar enough to not rate down further.

Evidence Set 4: Screening vs. usual care; clinical fragility fractures (Offer-to-screen in selected populations & Acceptors of screening)

Included studies: Acceptors: Barr 2010 – APOSS (women 45-54 years); Offer-to-screen in selected: Merlijn 2019 - SALT, Rubin 2018 - ROSE, Shepstone 2018 – SCOOP (women ≥65 years)

4B. GRADE Summary of Findings Table

Outcome<br>No. participants<br>(studies)	Relative effects, HR (95% CI)	Anticipated absolute effects* (95% CI)			Certainty of evidence (GRADE)**	What happens?
		Without screening	With screening	Difference		
**Women 45-54 years of age, all eligible who completed screening (Barr 2010 - APOSS)**						
Major osteoporotic fractures (hip, clinical vertebral, humerus, wrist)<br><br>Follow-up: 9 years<br><br>2,604 (1 RCT)	0.73 (0.46 to 1.14)	Control event rate			VERY LOW<br>⊕⊖⊖⊖<br>(control event rate)[a-d] due to risk of bias, inconsistency, indirectness, and imprecision	The evidence about the effects on clinical fragility fractures in women 45-54 years of age who accept screening is very uncertain.
		34 per 1000	24.8 per 1000 (15.6 to 38.8)	9.2 fewer per 1000 (18.4 fewer to 4.8 more)		
		General population risk †			VERY LOW<br>⊕⊖⊖⊖<br>(control event rate)[a,b,d,e] due to risk of bias, inconsistency, indirectness, and imprecision	
		67 per 1000	48.9 per 1000 (30.8 to 76.4)	18.1 fewer per 1000 (36.2 fewer to 9.4 more)		
**Women ≥65 years of age, all eligible who completed screening (Merlijn 2019 – SALT, Rubin 2018 – ROSE, Shepstone 2018 – SCOOP)**						
Major osteoporotic fractures (hip, clinical vertebral, humerus, wrist) ‡<br><br>Follow-up: 3-5 years<br><br>42,009 (3 RCT)	0.93 (0.87 to 0.99)	Median control event rate			MODERATE<br>⊕⊕⊕⊖<br>(median control event rate)[f,g] due to indirectness	Offering screening to selected populations of women ≥65 years of age among which compliance might be higher than the general population (filled in a FRAX questionnaire) probably reduces the risk of clinical fragility fracture compared to no offer of screening.
		84 per 1000	78.1 per 1000 (73.1 to 83.2)	5.9 fewer per 1000 (10.9 fewer to 0.8 fewer)		

Evidence Set 4: Screening vs. usual care; clinical fragility fractures (Offer-to-screen in selected populations & Acceptors of screening)
Included studies: Acceptors: Barr 2010 – APOSS (women 45-54 years); Offer-to-screen in selected: Merlijn 2019 - SALT, Rubin 2018 - ROSE, Shepstone 2018 – SCOOP (women ≥65 years)

		General population risk†			MODERATE ⨁⨁⨁⊖ (median control event rate)f,h due to indirectness	
		168 per 1000	156.2 (146.2 to 166.3)	11.8 fewer per 1000 (21.8 fewer to 1.7 fewer)		

CI: confidence interval; HR: hazard ratio; RCT: randomized controlled trial
* The absolute effect (and its 95% CI) without screening (i.e. baseline rate) is based on the estimated risk in the comparison group; the effect with screening is based on applying the relative effect of the intervention (and its 95% CI) to the effect without screening.
** When our assessment of the certainty of evidence fell between levels, we assigned the level that best represented our actual certainty in the evidence, and based our conclusions on this the level of certainty.
† The effects without screening for the general risk population are estimated from PRIOR et al., based on 10 year follow-up [1]
‡ Shepstone 2018 defined these as 'osteoporosis related fractures', which included all except hands, feet, nose, skull, cervical vertebrae, and vertebral fractures documented within 6 months of randomization.

Explanations:
a Risk of bias: Serious concerns about attrition bias due to a high proportion of losses to follow-up (42%), contamination of the control group (21.6% were taking anti-osteoporosis medications, not including hormone replacement therapy, at follow-up vs. 36.6% in the screened group), and selection bias because the analysis is per protocol.
b Inconsistency: Some concern about lack of evidence of consistency because there is only one study in the analysis.
c Indirectness: Serious concern that the population differs from the population of interest because a relatively large proportion of participants may have been taking anti-osteoporosis drugs at baseline. The comparator differs from that of interest (no screening), because it includes ad-hoc screening. Recruitment method (via letters) not typical but effect on the outcome may be limited.
d Imprecision: The total number of events does not meet the optimal information size (<300).
e Indirectness Serious concern that the population differs from the population of interest because a relatively large proportion of participants may have been taking anti-osteoporosis drugs at baseline. The comparator differs from that of interest (no screening), because it includes ad-hoc screening. Recruitment method (via letters) not typical but effect on the outcome may be limited.
f Risk of bias: Did not rate down because a significant benefit of screening was observed despite concerns (contamination of the control group [Rubin 2018, Shepstone 2018, Merlijn 2019]) that are likely to bias the findings toward the null.
g Indirectness: The comparator differs from that of interest (no screening), because it includes ad-hoc screening. Due to the ascertainment method, the outcome may include an unknown number of non-clinical vertebral fractures. Recruitment methods and education of GPs not typical but effect on the outcomes is unclear. Merlijn 2019 enrolls a high risk population, but removal of this study does not change the findings.

Evidence Set 4: Screening vs. usual care; clinical fragility fractures (Offer-to-screen in selected populations & Acceptors of screening)
Included studies: Acceptors: Barr 2010 – APOSS (women 45-54 years); Offer-to-screen in selected: Merlijn 2019 - SALT, Rubin 2018 - ROSE, Shepstone 2018 – SCOOP (women ≥65 years)

[h] Indirectness: The comparator differs from that of interest (no screening), because it includes ad-hoc screening. Recruitment methods and education of GPs not typical but effect on the outcomes is unclear. Due to the ascertainment method, the outcome may include an unknown number of non-clinical vertebral fractures. Merlijn 2019 enrolls a high risk population, but removal of this study does not change the findings. There is uncertainty about whether the relative HR can be applied across populations with differing levels of baseline risk, however the baseline risk is similar enough to not rate down further.

4C. Forest Plot

Study or Subgroup	log[Hazard Ratio]	SE	Screening Total	Usual care Total	Weight	Hazard Ratio IV, Random, 95% CI	Risk of Bias A B C D E F G
**1.4.1 Women 45-54 years**							
Barr 2010 (APOSS)	-0.32	0.23	1240	1364	100.0%	0.73 [0.46, 1.14]	? + − ? − ? +
**Subtotal (95% CI)**			**1240**	**1364**	**100.0%**	**0.73 [0.46, 1.14]**	
Heterogeneity: Not applicable							
Test for overall effect: Z = 1.39 (P = 0.16)							
**1.4.2 Women ≥ 65 years**							
Merlijn 2019 (SALT)	-0.08338161	0.0662	5516	5405	22.7%	0.92 [0.81, 1.05]	+ + ? + + + +
Rubin 2018 (ROSE)	-0.08121006	0.05136	9279	9326	37.6%	0.92 [0.83, 1.02]	? + − ? + + +
Shepstone 2018 (SCOOP)	-0.06	0.05	6233	6250	39.7%	0.94 [0.85, 1.04]	+ + − + + + +
**Subtotal (95% CI)**			**21028**	**20981**	**100.0%**	**0.93 [0.87, 0.99]**	
Heterogeneity: Tau² = 0.00; Chi² = 0.12, df = 2 (P = 0.94); I² = 0%							
Test for overall effect: Z = 2.33 (P = 0.02)							

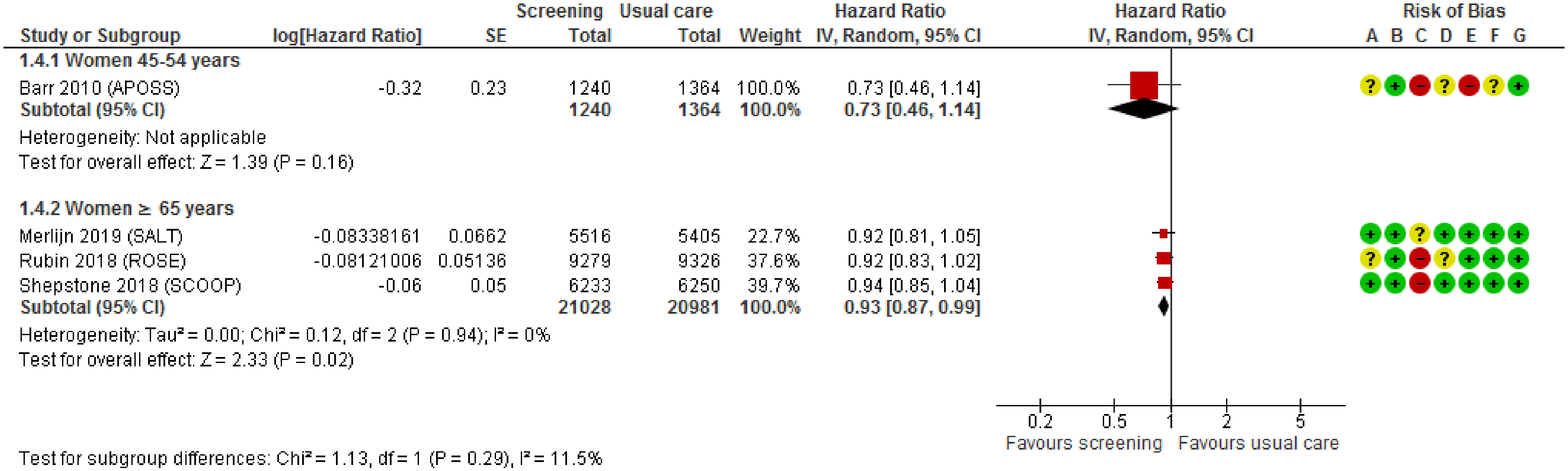

Test for subgroup differences: Chi² = 1.13, df = 1 (P = 0.29), I² = 11.5%

Risk of bias legend
(A) Random sequence generation (selection bias)
(B) Allocation concealment (selection bias)
(C) Blinding of participants and personnel (performance bias): Fracture outcomes
(D) Blinding of outcome assessment (detection bias): Fracture outcomes
(E) Incomplete outcome data (attrition bias): Fracture outcomes
(F) Selective reporting (reporting bias)
(G) Other bias

* The relative risk was used for Barr 2010 because a hazard ratio was not presented in the study. The hazard ratio in the Rubin 2018 analysis takes into account competing risk of death and handles emigration as a censoring event. The hazard ratio in the Shepstone 2018 analysis regarded death or withdrawal from the study as a censoring event, and included recruiting region, baseline FRAX, and self-reported falls in the model (prognostic factors agreed on before analysis).

Evidence Set 5: Screening vs. no screening; all-cause mortality (All eligible for screening/Offer-to-screen; offer-to-screen in selected population)
Included studies: Barr 2010 - APOSS (women <65 years), Rubin 2018 – ROSE (women 68-80 years); Kern 2005 [CCT], Merlijn 2019, Shepstone 2018 (women ≥65 years)

5A. GRADE Evidence Profile Table

Population	Certainty assessment							№ of patients		Effect		Certainty*
	№ of studies	Study design	Risk of bias	Inconsistency	Indirectness	Imprecision	Other consideration	Screen-ing	Usual care	Relative Risk (95% CI)	Absolute (95% CI)	
**Women 45-54 years of age, offer-to-screen (Barr 2010 - APOSS)**												
**Control event rate (3.3% or 33 per 1000)**	1	RCT	NC[a]	-0.5[b]	NC[c]	-2[d]	NC	2400	2400	0.99 (0.72 to 1.35)	0.3 fewer per 1000 (9.2 fewer to 11.6 more)	VERY LOW ⊕⊖⊖⊖
**General population risk (0.3% or 3 per 1000)****	1	RCT	NC[a]	-0.5[b]	NC[e]	-1[f]	NC	2400	2400	0.99 (0.72 to 1.35)	No difference per 1000 (0.8 fewer to 1.1 more)	LOW ⊕⊕⊖⊖
**Women 68-80 years of age, offer-to-screen (Rubin 2018 – ROSE) – offer-to-screen**												
**Control event rate (11.8% or 118 per 1000)**	1	RCT	NC[g]	-0.5[b]	NC[h]	-1[i]	NC	17,072	17,157	0.97 (0.92 to 1.03)	3.5 fewer per 1000 (9.4 fewer to 3.5 more)	LOW ⊕⊖⊖⊖
**General population risk (5.7% or 57 per 1000)****	1	RCT	NC[g]	-0.5[b]	NC[j]	-1[i]	NC	17,072	17,157	0.97 (0.92 to 1.03)	1.7 fewer per 1000 (4.6 fewer to 1.7 more)	LOW ⊕⊖⊖⊖
**Women ≥65 years of age, offer-to-screen in selected population (Merlijn 2019 – SALT, Shepstone 2018 – SCOOP, Kern 2005 [CCT])** †												
**Control event rate (8.9% or 89 per 1000)**	3	RCT/CCT	NC[k]	NC	NC[l]	-1[i]	NC	13,171	13,340	1.00 (0.92 to 1.09)	No difference per 1000 (7.1 fewer to 5.3 more)	MODERATE ⊕⊕⊕⊖

Evidence Set 5: Screening vs. no screening; all-cause mortality (All eligible for screening/Offer-to-screen; offer-to-screen in selected population)
Included studies: Barr 2010 - APOSS (women <65 years), Rubin 2018 – ROSE (women 68-80 years); Kern 2005 [CCT], Merlijn 2019, Shepstone 2018 (women ≥65 years)

Population	Certainty assessment							№ of patients		Effect		Certainty[*]
	№ of studies	Study design	Risk of bias	Inconsistency	Indirectness	Imprecision	Other consideration	Screen-ing	Usual care	Relative Risk (95% CI)	Absolute (95% CI)	
**General population risk (5.7% or 57 per 1000)****	3	RCT/CCT	NC[k]	NC	NC[m]	-1[i]	NC	13,171	13,340	1.00 (0.92 to 1.09)	No difference per 1000 (4.6 fewer to 5.1 more)	MODERATE ⊕⊕⊖⊖

CI: confidence interval; HR: hazard ratio; NC: no serious concerns; RCT: randomized controlled trial

[*] When our assessment of the certainty of evidence fell between levels, we assigned the level that best represented our actual certainty in the evidence, and based our conclusions on this the level of certainty.

[**] Estimated from 2017 population data available from Statistics Canada for women 40-65 years and >65 years [2]

[†] There were 1379 men in this analysis from Kern 2005 (5.4%)

Explanations:

[a] Risk of bias: Some concern about contamination of the control group, but it is unclear how this might affect the mortality outcome.

[b] Inconsistency: Some concern about lack of evidence of consistency because there is only one study in the analysis.

[c] Indirectness: Some concern that the population differs from the population of interest because a relatively large proportion of participants may have been taking anti-osteoporosis drugs at baseline (potentially high risk), but unlikely to affect the mortality outcome. Recruitment method not typical (via letters) but effect on the outcome may be limited.

[d] Imprecision: The number of events does not meet the optimal information size (<300). The confidence interval includes potential for both important benefit and harm.

[e] Indirectness: Serious concern that the population differs from the population of interest because a relatively large proportion of participants may have been taking anti-osteoporosis drugs at baseline, but effect on mortality outcome is unclear. Recruitment method (via letters) and education of GPs not typical but effect on the outcome may be limited. There is uncertainty about whether the relative HR can be applied across populations with differing levels of baseline risk, however the baseline risk is similar enough to not rate down further.

[f] Imprecision: The number of events does not meet the optimal information size (<300). Though the confidence interval for absolute effects includes potential for small benefit and harm, it is relatively narrow.

[g] Risk of bias: Some concern about contamination of the control group. 25% of the control group had a DXA scan after the index date vs. 48% in the screening group. Use of anti-osteoporosis medications was 18% in the control group vs. 23% in the screening group. Effect on mortality is unclear.

Evidence Set 5: Screening vs. no screening; all-cause mortality (All eligible for screening/Offer-to-screen; offer-to-screen in selected population)
Included studies: Barr 2010 - APOSS (women <65 years), Rubin 2018 – ROSE (women 68-80 years); Kern 2005 [CCT], Merlijn 2019, Shepstone 2018 (women ≥65 years)

[h] Indirectness: Some concern that the population differs from the population of interest because 11% of participants who returned complete questionnaires were on osteoporotic treatment at baseline, but unlikely to affect the mortality outcome.
[i] Imprecision: Serious concern that the confidence interval includes the potential for both important benefit and harm. However, the sample size is large.
[j] Indirectness: Serious concern that the population differs from the population of interest because 11% of participants who returned complete questionnaires were on osteoporotic treatment at baseline, but unlikely to affect the mortality outcome. There is uncertainty about whether the relative HR can be applied across populations with differing levels of baseline risk, however the baseline risk is similar enough to not rate down further.
[k] Risk of bias: Some concern about contamination of the control groups, and selective reporting in Kern 2005, but it is unclear how it might affect the mortality outcome.
[l] Indirectness: Merlijn 2019 enrolls a high risk population, but removal of this study does not change the findings. Kern 2005 is a CCT, but removal of this study does not change the findings. There were a small proportion of men (5.4%) in the analysis from Kern 2005; unlikely that this affected the findings.
[m] Indirectness Merlijn 2019 enrolls a high risk population, but removal of this study does not change the findings. Kern 2005 is a CCT, but removal of this study does not change the findings. There were a small proportion of men (5.4%) in the analysis from Kern 2005; unlikely that this affected the findings.
There is uncertainty about whether the relative HR can be applied across populations with differing levels of baseline risk, however the level of risk is similar enough to not rate down.

Evidence Set 5: Screening vs. no screening; all-cause mortality (All eligible for screening/Offer-to-screen; offer-to-screen in selected population)
Included studies: Barr 2010 - APOSS (women <65 years), Rubin 2018 – ROSE (women 68-80 years); Kern 2005 [CCT], Merlijn 2019, Shepstone 2018 (women ≥65 years)

5B. GRADE Summary of Findings Table

Outcome<br>No. participants (studies)	Relative effects, RR (95% CI)	Anticipated absolute effects* (95% CI)<br>Without screening	With screening	Difference	Certainty of evidence (GRADE)**	What happens?
**Women <65 years, offer-to-screen (Barr 2010 - APOSS)**						
All-cause mortality<br><br>Follow-up: 9 years<br><br>4,800 (1 RCT)	0.99 (0.72 to 1.35)	Control event rate			VERY LOW<br>⊕⊖⊖⊖<br>(median control event rate)[a-d] due to inconsistency and imprecision	The evidence about the effects on all-cause mortality from offering screening to women 45-54 years of age is very uncertain.
		33 per 1000	32.7 per 1000 (23.8 to 44.6)	0.3 fewer per 1000 (9.2 fewer to 11.6 more)		
		General population risk†			LOW<br>⊕⊕⊖⊖<br>(general population risk)[a,b,e,f] due to inconsistency and imprecision	Offering screening to women 45-54 years of age may not reduce the risk of all-cause mortality compared to no offer of screening, but the evidence is uncertain.
		3 per 1000	3.0 per 1000 (2.2 to 4.1)	No difference per 1000 (0.8 fewer to 1.1 more)		
**Women 68-80 years, offer-to-screen (Rubin 2018 – ROSE)**						
All-cause mortality<br><br>Follow-up: 5 years<br><br>34,299 (1 RCT)	0.97 (0.92 to 1.03)	Control event rate			LOW<br>⊕⊖⊖⊖<br>(median control event rate)[b, g-i] due to inconsistency and imprecision	Offering screening to women 68-80 years of age may not reduce the risk of all-cause mortality compared to no offer of screening, but the evidence is uncertain.
		118 per 1000	114.5 per 1000 (108.6 to 121.5)	3.5 fewer per 1000 (9.4 fewer to 3.5 more)		
		General population risk†			LOW<br>⊕⊖⊖⊖<br>(general population risk)[b, g,i,j] due to inconsistency and imprecision	
		57 per 1000	55.3 per 1000 (52.4 to 58.7)	1.7 fewer per 1000 (4.6 fewer to 1.7 more)		

Evidence Set 5: Screening vs. no screening; all-cause mortality (All eligible for screening/Offer-to-screen; offer-to-screen in selected population)
Included studies: Barr 2010 - APOSS (women <65 years), Rubin 2018 – ROSE (women 68-80 years); Kern 2005 [CCT], Merlijn 2019, Shepstone 2018 (women ≥65 years)

Outcome<br>No. participants (studies)	Relative effects, RR (95% CI)	Anticipated absolute effects* (95% CI)			Certainty of evidence (GRADE)**	What happens?
		Without screening	With screening	Difference		
**Women ≥65 years, offer-to-screen in selected population (Merlijn 2019 – SALT, Shepstone 2018 – SCOOP, Kern 2005 [CCT])** ‡						
All-cause mortality<br><br>Follow-up: 3-5 years<br><br>26,511 (2 RCT, 1 CCT)	1.00 (0.92 to 1.09)	Median control event rate			MODERATE<br>⊕⊕⊕⊖<br>(median control event rate)[l,k,l] due to imprecision	Offering screening to women ≥65 years probably does not reduce the risk of all-cause mortality compared to no offer of screening.
		89 per 1000	89.0 per 1000 (81.9 to 94.3)	No difference in 1000 (7.1 fewer to 5.3 more)		
		General population risk†			MODERATE<br>⊕⊕⊕⊖<br>(general population risk)[l,k,m] due to imprecision	
		57 per 1000	57.0 per 1000 (52.4 to 62.1)	No difference in 1000 (4.6 fewer to 5.1 more)		

CI: confidence interval; HR: hazard ratio; RCT: randomized controlled trial
* The absolute effect (and its 95% CI) without screening (i.e. baseline rate) is based on the estimated risk in the comparison group; the effect with screening is based on applying the relative effect of the intervention (and its 95% CI) to the effect without screening.
** When our assessment of the certainty of evidence fell between levels, we assigned the level that best represented our actual certainty in the evidence, and based our conclusions on this the level of certainty.
† Rates in the control group are estimated from 2017 population data available from Statistics Canada for women 40-65 years and >65 years [2]
‡ There were 1379 men in this analysis from Kern 2005 (5.4% of the total sample)

Explanations:
[a] Risk of bias: Some concern about contamination of the control group, but it is unclear how this might affect the mortality outcome.
[b] Inconsistency: Some concern about lack of evidence of consistency because there is only one study in the analysis.
[c] Indirectness: Some concern that the population differs from the population of interest because a relatively large proportion of participants may have been taking anti-osteoporosis drugs at baseline (potentially high risk), but unlikely to affect the mortality outcome. Recruitment method not typical (via letters) but effect on the outcome may be limited.
[d] Imprecision: The number of events does not meet the optimal information size (<300). The confidence interval includes potential for both important benefit and harm.
[e] Indirectness: Serious concern that the population differs from the population of interest because a relatively large proportion of participants may have been taking anti-osteoporosis drugs at baseline, but effect on mortality outcome is unclear. Recruitment method (via letters) and education of GPs not typical but

effect on the outcome may be limited. There is uncertainty about whether the relative HR can be applied across populations with differing levels of baseline risk, however the baseline risk is similar enough to not rate down further.

[f] Imprecision: The number of events does not meet the optimal information size (<300). Though the confidence interval for absolute effects includes potential for small benefit and harm, it is relatively narrow.

[g] Risk of bias: Some concern about contamination of the control group. 25% of the control group had a DXA scan after the index date vs. 48% in the screening group. Use of anti-osteoporosis medications was 18% in the control group vs. 23% in the screening group. Effect on mortality outcome is unclear.

[h] Indirectness: Some concern that the population differs from the population of interest because 11% of participants who returned complete questionnaires were on osteoporotic treatment at baseline, but unlikely to affect the mortality outcome.

[i] Imprecision: Serious concern that the confidence interval includes the potential for both important benefit and harm. However, the sample size is large.

[j] Indirectness: Serious concern that the population differs from the population of interest because 11% of participants who returned complete questionnaires were on osteoporotic treatment at baseline, but unlikely to affect the mortality outcome. There is uncertainty about whether the relative HR can be applied across populations with differing levels of baseline risk, however the baseline risk is similar enough to not rate down further.

[k] Risk of bias: Some concern about contamination of the control groups, and selective reporting in Kern 2005, but it is unclear how it might affect the mortality outcome.

[l] Indirectness: Merlijn 2019 enrolls a high risk population, but removal of this study does not change the findings. Kern 2005 is a CCT, but removal of this study does not substantially change the findings. There were a small proportion of men (5.4%) in the analysis from Kern 2005; unlikely that this affected the findings.

[m] Indirectness Merlijn 2019 enrolls a high risk population, but removal of this study does not change the findings. Kern 2005 is a CCT, but removal of this study does not substantially change the findings. There were a small proportion of men (5.4%) in the analysis from Kern 2005; unlikely that this affected the findings. There is uncertainty about whether the relative HR can be applied across populations with differing levels of baseline risk, however the level of risk is similar enough to not rate down

Evidence Set 5: Screening vs. no screening; all-cause mortality (All eligible for screening/Offer-to-screen; offer-to-screen in selected population)
Included studies: Barr 2010 - APOSS (women <65 years), Rubin 2018 – ROSE (women 68-80 years); Kern 2005 [CCT], Merlijn 2019, Shepstone 2018 (women ≥65 years)

5C. Forest Plots

Offer-to-screen

Study or Subgroup	Screening Events	Screening Total	No screening Events	No screening Total	Weight	Risk Ratio M-H, Random, 95% CI	Risk of Bias A B C D E F G
**1.9.1 Women 45-50 years of age**							
Barr 2010 (APOSS)	77	2400	78	2400	100.0%	0.99 [0.72, 1.35]	+ + ? + + ? +
**Subtotal (95% CI)**		**2400**		**2400**	**100.0%**	**0.99 [0.72, 1.35]**	
Total events	77		78				
Heterogeneity: Not applicable							
Test for overall effect: Z = 0.08 (P = 0.93)							
**1.9.3 Women 68-80 years of age**							
Rubin 2018 (ROSE)	1968	17072	2038	17157	100.0%	0.97 [0.92, 1.03]	+ + ? + + + +
**Subtotal (95% CI)**		**17072**		**17157**	**100.0%**	**0.97 [0.92, 1.03]**	
Total events	1968		2038				
Heterogeneity: Not applicable							
Test for overall effect: Z = 1.01 (P = 0.31)							

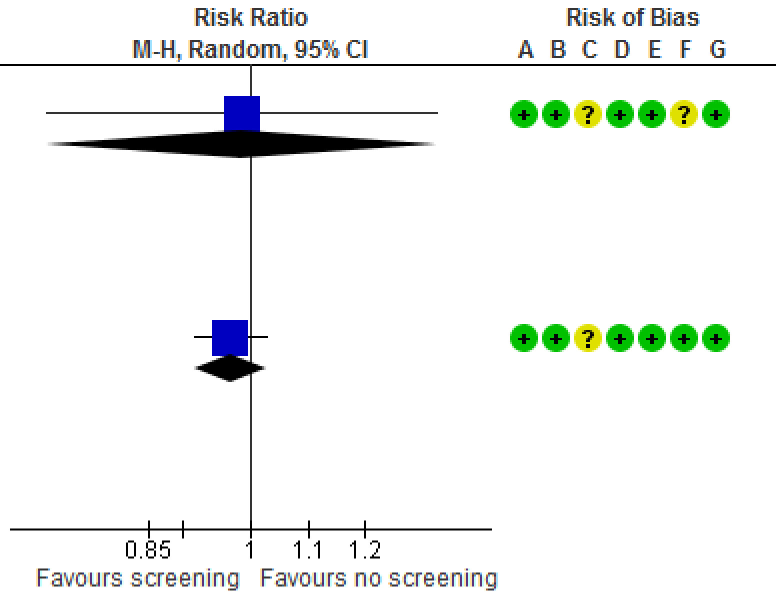

Test for subgroup differences: Chi² = 0.01, df = 1 (P = 0.92), I² = 0%

Risk of bias legend
(A) Random sequence generation (selection bias)
(B) Allocation concealment (selection bias)
(C) Blinding of participants and personnel (performance bias)
(D) Blinding of outcome assessment (detection bias)
(E) Incomplete outcome data (attrition bias)
(F) Selective reporting (reporting bias)
(G) Other bias

*Data for Rose 2018 were provided by the study authors.

Evidence Set 5: Screening vs. no screening; all-cause mortality (All eligible for screening/Offer-to-screen; offer-to-screen in selected population)
Included studies: Barr 2010 - APOSS (women <65 years), Rubin 2018 – ROSE (women 68-80 years); Kern 2005 [CCT], Merlijn 2019, Shepstone 2018 (women ≥65 years)

Offer-to-screen in selected populations*

Study or Subgroup	Screening Events	Screening Total	No screening Events	No screening Total	Weight	Risk Ratio M-H, Random, 95% CI	A	B	C	D	E	F	G
**1.10.2 Women ≥ 65 years of age**													
Kern 2005	256	1422	337	1685	26.5%	0.90 [0.78, 1.04]	?	?	?	+	+	−	+
Merlijn 2019 (SALT)	499	5516	479	5405	35.6%	1.02 [0.91, 1.15]	+	+		+	+	+	+
Shepstone 2018 (SCOOP)	550	6233	525	6250	37.9%	1.05 [0.94, 1.18]	+	+	?	+	+	+	+
**Subtotal (95% CI)**		**13171**		**13340**	**100.0%**	**1.00 [0.92, 1.09]**							
Total events	1305		1341										

Heterogeneity: Tau² = 0.00; Chi² = 2.82, df = 2 (P = 0.24); I² = 29%
Test for overall effect: Z = 0.04 (P = 0.96)

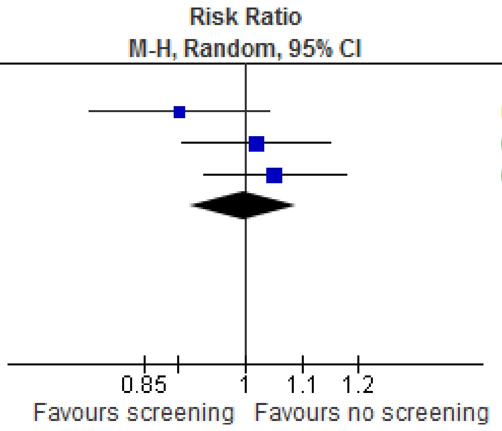

Test for subgroup differences: Not applicable

Risk of bias legend
(A) Random sequence generation (selection bias)
(B) Allocation concealment (selection bias)
(C) Blinding of participants and personnel (performance bias)
(D) Blinding of outcome assessment (detection bias)
(E) Incomplete outcome data (attrition bias)
(F) Selective reporting (reporting bias)
(G) Other bias

* There were 1379 men in this analysis from Kern 2005 (5.4% of the total sample).

Evidence Set 6: Screening vs. no screening; serious adverse events (acceptors of screening)
Included studies: Shepstone 2018 – SCOOP (women ≥65 years)

6A. GRADE Evidence Profile Table

Population	Certainty assessment							№ of patients		Effect		Certainty*
	№ of studies	Study design	Risk of bias	Inconsistency	Indirectness	Imprecision	Other consideration	Screening	Usual care	Relative HR (95% CI)	Absolute (95% CI)	
**Women 70-85 years of age (Shepstone 2018 – SCOOP)**												
**Control event rate NR**	1	RCT	-1[a]	-0.5[b]	NC	-1[c]	NC	6233	6250	Not applicable	General practitioners reported no serious adverse events related to the screening process.	VERY LOW ⨁⊖⊖⊖

CI: confidence interval; HR: hazard ratio; RCT: randomized controlled trial
* When our assessment of the certainty of evidence fell between levels, we assigned the level that best represented our actual certainty in the evidence, and based our conclusions on this the level of certainty.

Explanations:
[a] Risk of bias: Some concern about reporting bias due to unblinded and passive collection of data related to subjective outcomes. Serious concern about selective reporting, since only one of the five included studies reported on adverse events.
[b] Inconsistency: Some concern about lack of evidence for consistency because only one study reported this outcome.
[c] Imprecision: The total number of events does not meet the optimal information size (<300).

Evidence Set 6: Screening vs. no screening; serious adverse events (acceptors of screening)

Included studies: Shepstone 2018 – SCOOP (women ≥65 years)

6B. GRADE Summary of Findings Table

Outcome<br>No. participants (studies)	Relative effects, (95% CI)	Anticipated absolute effects* (95% CI)			Certainty of evidence (GRADE)**	What happens?
		Without screening	With screening	Difference		
**Women 70-85 years of age, all eligible for screening**						
Serious adverse events related to the screening process<br><br>Follow-up: 5 years<br><br>12,483 (1 RCT)	Not applicable	Control event rate			VERY LOW<br>⊕⊖⊖⊖<br>Due to risk of bias, inconsistency, and imprecision[a-c]	The evidence about the effects on serious adverse events from offering screening to women 70-85 years of age is very uncertain.
		NR	0	Assuming no events in controls, there was no difference in the number of events in the screened group.		

CCT: clinical controlled trial; CI: confidence interval; HR: hazard ratio; RCT: randomized controlled trial; ROB: risk of bias

* The absolute effect (and its 95% CI) without screening (i.e. baseline rate) is based on the estimated risk in the comparison group; the effect with screening is based on applying the relative effect of the intervention (and its 95% CI) to the effect without screening.

** When our assessment of the certainty of evidence fell between levels, we assigned the level that best represented our actual certainty in the evidence, and based our conclusions on this the level of certainty.

Explanations:

[a] Risk of bias: Some concern about reporting bias due to unblinded and passive collection of data related to subjective outcomes. Serious concern about selective reporting, since only one of the five included studies reported on adverse events.

[b] Inconsistency: Some concern about lack of evidence for consistency because only one study reported this outcome.

[c] Imprecision: The total number of events does not meet the optimal information size (<300).

Evidence Set 7: Screening vs. no screening; wellbeing outcomes (All eligible/offer-to-screen & offer-to-screen in selected populations)
Included studies: Acceptors of screening: Barr 2010 (women <65 years); offer-to-screen in selected population: Shepstone 2018 (women ≥65 years)

7A. GRADE Evidence Profile Table

Population	Certainty assessment							№ of patients		Findings	Certainty*
	№ of studies	Study design	Risk of bias	Inconsistency	Indirectness	Imprecision	Other consideration	Screen-ing	Usual care		
**Women 45-54 years of age (Barr 2010 - APOSS)**											
**General health, measured on a 5-point scale (very good, good, satisfactory, not so good, poor)**	1	RCT	-1[a]	-0.5[b]	-1[c]	NC	NC	1433	1364	At follow-up (median 9.1 years in screened and 8.8 years in controls), 69.2% of the screened group and 68.0% of the control group reported good or very good general health. 18.0% and 17.7% reported their health as satisfactory, 11.3% and 12.2% as not so good, and 1.5% and 2.1% as poor in screened and control groups, respectively.	VERY LOW ⊕⊖⊖⊖
**Health-related quality of life measured using the SF-36 (range 0-100 with higher scores indicating better quality of life)**	1	RCT	-1[a]	-0.5[b]	-1[c]	NC	NC	611	606	At 2-year follow-up, mean (SD) SF-36 subscale scores were as follows for screened vs. control groups:<br>Physical functioning: 80.4 (23.4) vs. 81.1 (22.0)<br>Social functioning: 85.3 (23.1) vs. 85.0 (22.5)<br>Role-physical: 75.8 (36.9) vs. 78.8 (35.1)<br>Role-emotional: 79.3 (35.6) vs. 78.1 (35.4)<br>Mental health: 71.7 (18.3) vs. 71.4 (18.6)<br>Energy and fatigue: 59.0 (21.0) vs. 58.9 (20.8)<br>Pain: 73.8 (25.8) vs. 73.3 (24.9)<br>General health perception: 69.7 (21.7) vs. 69.8 (20.8)	VERY LOW ⊕⊖⊖⊖

Evidence Set 7: Screening vs. no screening; wellbeing outcomes (All eligible/offer-to-screen & offer-to-screen in selected populations)
Included studies: Acceptors of screening: Barr 2010 (women <65 years); offer-to-screen in selected population: Shepstone 2018 (women ≥65 years)

Population	Certainty assessment							№ of patients		Findings	Certainty*
	№ of studies	Study design	Risk of bias	Inconsistency	Indirectness	Imprecision	Other consideration	Screen-ing	Usual care		
**Women 70-85 years of age (Shepstone 2018 – SCOOP)**											
**Physical and mental health measured using the SF-12 (range 0-100 with higher scores indicating better quality of life)**	1	RCT	-1[d]	-0.5[b]	NC	NC	NC	5334	5327	Mean (SD) change from baseline to 5-years follow-up was -7.1 (15.9) in the screened group vs. -6.8 (15.8) in controls (MD -0.30, 95% CI -0.86, 0.26) for general mental health and -6.7 (14.6) in the screened group and -7.0 (14.5) in controls (MD 0.30, 95% CI -0.21, 0.81) for general physical health.	LOW ⊕⊕⊖⊖
**Health-related quality of life measured using the EuroQol-5D (range 0-1 with higher scores indicating better quality of life)**	1	RCT	-1[d]	-0.5[b]	NC	NC	NC	5334	5327	Mean (SD) change from baseline to 5-years follow-up was -0.11 (0.3) in the screened group vs. -0.11 (0.29) in controls (MD 0, 95% CI -0.07, 0.07).	LOW ⊕⊕⊖⊖

CI: confidence interval; NC: no serious concerns; MD: mean difference; RCT: randomized controlled trial; SD: standard deviation; SF-36 or -12: Short-Form Health Survey

* When our assessment of the certainty of evidence fell between levels, we assigned the level that best represented our actual certainty in the evidence, and based our conclusions on this the level of certainty.

Explanations:

[a] Risk of bias: Serious concerns about contamination of the control group (21.6% were taking anti-osteoporosis medications, not including HRT, at follow-up vs. 36.6% in the screened group), and about reporting bias (the outcome is subjective and self-reported).

Evidence Set 7: Screening vs. no screening; wellbeing outcomes (All eligible/offer-to-screen & offer-to-screen in selected populations)
Included studies: Acceptors of screening: Barr 2010 (women <65 years); offer-to-screen in selected population: Shepstone 2018 (women ≥65 years)

[b] Inconsistency: Some concern about lack of evidence of consistency because there is only one study in the analysis.
[c] Indirectness: Serious concern that a relatively large proportion of participants may have been taking anti-osteoporosis drugs at baseline.
[d] Risk of bias: Serious concern about contamination of the control group (16% of controls reported use of anti-osteoporosis medications at follow-up vs. 24% in the screened group), and about reporting bias (the outcome is subjective and self-reported).

7B. GRADE Summary of Findings Table

Outcome<br>No. participants (studies)	Findings	Certainty of evidence (GRADE)*	What happens?
**Women 45-54 years of age (Barr 2010 – APOSS)**			
General health<br><br>Follow-up: 9 years<br><br>2,797 (1 RCT)	One study of screening (1 step direct to BMD) in women <65 years measured general health on a 5-point scale. At follow-up (median 9.1 years in screened and 8.8 years in controls), 69.2% of the screened group and 68.0% of the control group reported good or very good general health. 18.0% and 17.7% reported their health as satisfactory, 11.3% and 12.2% as not so good, and 1.5 and 2.1% as poor in screened and control groups, respectively.	VERY LOW<br>⊕⊖⊖⊖<br>Due to risk of bias, inconsistency, and indirectness[a-c]	The evidence about the effects on general health from offering screening to women 45-54 years of age is very uncertain.
Health-related quality of life<br><br>Follow-up: 2 years<br><br>1,217 (1 RCT)	One study of screening (1 step direct to BMD) in women <65 years measured health-related quality of life in women using the Short-Form Health Survey-12. At 2-year follow-up, subscale scores were as follows for screening vs. control groups.<br>Physical functioning: 80.4 (23.4) vs. 81.1 (22.0)<br>Social functioning: 85.3 (23.1) vs. 85.0 (22.5)<br>Role-physical: 75.8 (36.9) vs. 78.8 (35.1)<br>Role-emotional: 79.3 (35.6) vs. 78.1 (35.4)<br>Mental health: 71.7 (18.3) vs. 71.4 (18.6)<br>Energy and fatigue: 59.0 (21.0) vs. 58.9 (20.8)<br>Pain: 73.8 (25.8) vs. 73.3 (24.9)<br>General health perception: 69.7 (21.7) vs. 69.8 (20.8)	VERY LOW<br>⊕⊖⊖⊖<br>Due to risk of bias, inconsistency, and indirectness[a-c]	The evidence about the effects on health-related quality of life from offering screening to women 45-54 years of age is very uncertain.

Evidence Set 7: Screening vs. no screening; wellbeing outcomes (All eligible/offer-to-screen & offer-to-screen in selected populations)
Included studies: Acceptors of screening: Barr 2010 (women <65 years); offer-to-screen in selected population: Shepstone 2018 (women ≥65 years)

Outcome<br>No. participants<br>(studies)	Findings	Certainty of evidence (GRADE)[*]	What happens?
**Women 70-85 years of age, all eligible for screening who responded at follow-up (Shepstone 2018 – SCOOP)**			
Health-related quality of life<br><br>Follow-up: 5 years<br><br>10,661 (1 RCT)	One study of screening (2 step: FRAX, then BMD offered if 10-y hip fracture risk using FRAX along met the 'assessment/high risk' threshold) in women ≥65 years measured health-related quality of life using the Short-Form Health Survey-12 and the EuroQol-5D.<br>SF-12: Mean (SD) change from baseline to 5-years follow-up was -7.1 (15.9) in the screened group vs. -6.8 (15.8) in controls (MD -0.30, 95% CI -0.86, 0.26) for general mental health and -6.7 (14.6) in the screened group and -7.0 (14.5) in controls (MD 0.30, 95% CI -0.21, 0.81) for general physical health.<br>EuroQol-5D: Mean (SD) change from baseline to 5-years follow-up was -0.11 (0.3) in the screened group vs. -0.11 (0.29) in controls (MD 0, 95% CI -0.07, 0.07).	LOW<br>⨁⨁⊖⊖<br>Due to risk of bias and inconsistency[b,d]	Offering screening to women 70-85 years of age may not improve health-related quality of life.

BMD: bone mineral density; RCT: randomized controlled trial; MD: mean difference; SD: standard deviation
[**] When our assessment of the certainty of evidence fell between levels, we assigned the level that best represented our actual certainty in the evidence, and based our conclusions on this the level of certainty.

Explanations:
[a] Risk of bias: Serious concerns about contamination of the control group (21.6% were taking anti-osteoporosis medications, not including HRT, at follow-up vs. 36.6% in the screened group), and about reporting bias (the outcome is subjective and self-reported).
[b] Inconsistency: Some concern about lack of evidence of consistency because there is only one study in the analysis.
[c] Indirectness: Serious concern that a relatively large proportion of participants may have been taking anti-osteoporosis drugs at baseline.
[d] Risk of bias: Serious concern about contamination of the control group (16% of controls reported use of anti-osteoporosis medications at follow-up vs. 24% in the screened group), and about reporting bias (the outcome is subjective and self-reported).

Calculations for overdiagnosis

Definition:
In the setting of screening to identify risk, we defined overdiagnosis as the identification of high risk in individuals who, if not screened, would never have known that they were at risk and would never have experienced a fragility fracture.

Calculation:
W = proportion (%) of individuals deemed at high risk (based on threshold) or shared decision making
y = mean % risk in this high risk population
100 – y = % who would theoretically not fracture

Extent of overdiagnosis = W x (100-y) / 100

Overdiagnosis using trial data:

**Trials**	**SCOOP (Shepstone 2018) Females 70-80 years 10-year risk of hip fracture**		**SALT (Merlijn 2019)[a] Females 65-90 years 10-year risk of MOF**
	**Offer-to-screen in select population**	**Screened as high-risk with clinical FRAX and referred for DXA**	**Offer-to-screen in select population**
Number offered screening	6233	3064	5575
Number above treatment threshold	898	898	1417
% above treatment threshold (W)	14.4%	29.3%	25.4%
Mean risk in high risk group (y) [b]	17.9%	17.9%	23.9%
Calculation of overdiagnosis	14.4 x (100-17.9) / 100	29.3 x (100-17.9) / 100	25.4 x (100-23.9) / 100
**% overdiagnosed**	**11.8%**	**24.1%**	**19.3%**

MOF=major osteoporotic fracture
[a] This study included only women with at least one risk factor, so the proportion above the treatment threshold would be expected to be higher than the general population.
[b] Calculated using clinical FRAX (without BMD); note that the trials did not use clinical FRAX for treatment thresholds.

Evidence Set 8: 1-step vs. 2-step screening; hip fractures (All eligible/Offer-to-screen)
Included studies: LaCroix 2005 – OPRA (women 60-80 years)

8A. GRADE Evidence Profile Table

Population	Certainty assessment							№ of patients		Effect		Certainty[‡]
	№ of studies	Study design	Risk of bias	Inconsistency	Indirectness	Imprecision	Other consideration	2-step[*]	1-step[†]	Relative Rate Ratio (95% CI)	Absolute (95% CI)	
**Women 60-80 years (LaCroix 2005 - OPRA)**												
**2-step event rate (1.2% or 12 per 1000)**	1	RCT	-1[a]	-0.5[b]	NC[c]	-1[d]	NC	7282	1986	0.70 (0.42 to 1.18)	3.6 fewer per 1000 (7.0 fewer to 2.1 more)	VERY LOW ⨁⊖⊖⊖

BMD: bone mineral density; CI: confidence interval; NC: no serious concerns; RCT: randomized controlled trial

[*] SCORE-based tool + BMD or SOF-based tool + BMD

[†] BMD only

[‡] When our assessment of the certainty of evidence fell between levels, we assigned the level that best represented our actual certainty in the evidence, and based our conclusions on this the level of certainty.

Explanations:

[a] Risk of bias: Serious concern as the single study in the analysis is at risk of selection, performance, and detection biases.

[b] Inconsistency: Some concern about lack of evidence of consistency because there is only one study in the analysis.

[c] Indirectness: Recruitment method not typical (via letters), but effect on the outcome may be limited.

[d] Imprecision: The number of events does not meet the optimal information size (<300).

Evidence Set 8: 1-step vs. 2-step screening; hip fractures (All eligible/Offer-to-screen)

Included studies: LaCroix 2005 – OPRA (women 60-80 years)

8B. GRADE Summary of Findings Table

Outcome<br>No. participants (studies)	Relative effects, Rate Ratio (95% CI)	Anticipated absolute effects* (95% CI)			Certainty of evidence (GRADE)**	What happens?
		2-step screening†	1-step screening‡	Difference		
**Women 60-80 years (LaCroix 2005, OPRA)**						
Hip fractures<br><br>Follow-up: mean 2.3 years<br><br>9,268 (1 RCT)	0.70 (0.42 to 1.18)	2-step event rate<br>12 per 1000	8.4 per 1000 (5.0 to 14.1)	3.6 fewer per 1000 (7.0 fewer to 2.1 more)	VERY LOW<br>⊕⊖⊖⊖<br>due to risk of bias, inconsistency, and imprecision[a-d]	The evidence is very uncertain.

BMD: bone mineral density; CI: confidence interval; RCT: randomized controlled trial

* The absolute effect (and its 95% CI) with 2-step screening (i.e. baseline rate) is based on the estimated risk in the 2-step screening group; the effect with 1-step screening is based on applying the relative effect of the intervention (and its 95% CI) to the effect in the 1-step group.

† SCORE-based tool + BMD or SOF-based tool + BMD

‡ BMD only

** When our assessment of the certainty of evidence fell between levels, we assigned the level that best represented our actual certainty in the evidence, and based our conclusions on this the level of certainty.

Explanations:

[a] Risk of bias: Serious concern as the single study in the analysis is at risk of selection, performance, and detection biases.

[b] Inconsistency: Some concern about lack of evidence of consistency because there is only one study in the analysis.

[c] Indirectness: Recruitment method not typical (via letters), but effect on the outcome may be limited.

[d] Imprecision: The number of events does not meet the optimal information size (<300).

Evidence Set 8: 1-step vs. 2-step screening; hip fractures (All eligible/Offer-to-screen)
Included studies: LaCroix 2005 – OPRA (women 60-80 years)

8C. Forest Plot

Study or Subgroup	log[Rate Ratio]	SE	1-step Total	2-step Total	Weight	Rate Ratio IV, Random, 95% CI	Rate Ratio IV, Random, 95% CI	Risk of Bias A B C D E F G
LaCroix 2005 (OPRA)	-0.3542	0.265	1986	7282	100.0%	0.70 [0.42, 1.18]		? ? ? ? + + +
**Total (95% CI)**			**1986**	**7282**	**100.0%**	**0.70 [0.42, 1.18]**		

Heterogeneity: Not applicable
Test for overall effect: Z = 1.34 (P = 0.18)

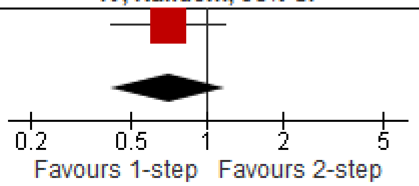

Risk of bias legend
(A) Random sequence generation (selection bias)
(B) Allocation concealment (selection bias)
(C) Blinding of participants and personnel (performance bias): Fracture outcomes
(D) Blinding of outcome assessment (detection bias): Fracture outcomes
(E) Incomplete outcome data (attrition bias): Fracture outcomes
(F) Selective reporting (reporting bias)
(G) Other bias

*The rate ratio is age-adjusted

Evidence Set 9: 1-step vs. 2-step; hip fractures (Acceptors of screening)
Included studies: LaCroix 2005 – OPRA (women 60-80 years)

9A. GRADE Evidence Profile Table

Population	Certainty assessment							№ of patients		Effect		Certainty[‡]
	№ of studies	Study design	Risk of bias	Inconsistency	Indirectness	Imprecision	Other consideration	2-step[*]	1-step[†]	Relative Rate Ratio (95% CI)	Absolute (95% CI)	
**Women 60-80 years (LaCroix 2005 - OPRA)**												
**2-step event rate (0.9% per 9 per 1000)**	1	RCT	-1[a]	-0.5[b]	NC[c]	-1[d]	NC	2752	415	0.38 (0.07 to 2.14)	5.6 fewer per 1000 (8.4 fewer to 10.3 more)	VERY LOW ⊕⊖⊖⊖

BMD: bone mineral density; CI: confidence interval; NC: no serious concerns; RCT: randomized controlled trial
[*] SCORE-based tool + BMD or SOF-based tool + BMD
[†] BMD
[‡] When our assessment of the certainty of evidence fell between levels, we assigned the level that best represented our actual certainty in the evidence, and based our conclusions on this the level of certainty.

Explanations:
[a] Risk of bias: Concern about selection bias because the per protocol population may differ from the randomized sample. Some concern about performance and detection biases.
[b] Inconsistency: Some concern about lack of evidence of consistency because there is only one study in the analysis.
[c] Indirectness: Recruitment method not typical (via letters) but effect on outcomes may be limited.
[d] Imprecision: The number of events does not meet the optimal information size (<300).

Evidence Set 9: 1-step vs. 2-step; hip fractures (Acceptors of screening)
Included studies: LaCroix 2005 – OPRA (women 60-80 years)

9B. GRADE Summary of Findings Table

Outcome No. participants (studies)	Relative effects, Rate Ratio (95% CI)	Anticipated absolute effects* (95% CI)			Certainty of evidence (GRADE)**	What happens?
		2-step screening†	1-step screening‡	Difference		
**Women 60-80 years (LaCroix 2005, OPRA)**						
Hip fractures<br><br>Follow-up: mean 2.3 years<br><br>3,167 (1 RCT)	0.38 (0.07 to 2.14)	2-step event rate<br>9 per 1000	3.4 per 1000 (0.6 to 19.3)	5.6 fewer per 1000 (8.4 fewer to 10.3 more)	VERY LOW<br>⨁⊖⊖⊖<br>due to risk of bias inconsistency, and imprecision[a-d]	The evidence is very uncertain.

BMD: bone mineral density; CI: confidence interval; RCT: randomized controlled trial

* The absolute effect (and its 95% CI) with 2-step screening (i.e. baseline rate) is based on the estimated risk in the 2-step screening group; the effect with 1-step screening is based on applying the relative effect of the intervention (and its 95% CI) to the effect in the 1-step group.

† SCORE-based tool + BMD or SOF-based tool + BMD

‡ BMD

** When our assessment of the certainty of evidence fell between levels, we assigned the level that best represented our actual certainty in the evidence, and based our conclusions on this the level of certainty.

Explanations:

[a] Risk of bias: Concern about selection bias because the per protocol population may differ from the randomized sample. Some concern about performance and detection biases.

[b] Inconsistency: Some concern about lack of evidence of consistency because there is only one study in the analysis.

[c] Indirectness: Recruitment method not typical (via letters) but effect on outcomes may be limited.

[d] Imprecision: The number of events does not meet the optimal information size (<300).

Evidence Set 9: 1-step vs. 2-step; hip fractures (Acceptors of screening)

Included studies: LaCroix 2005 – OPRA (women 60-80 years)

9C. Forest Plot

Study or Subgroup	log[Rate Ratio]	SE	1-step Total	2-step Total	Weight	Rate Ratio IV, Random, 95% CI	Risk of Bias A	B	C	D	E	F	G
LaCroix 2005 (OPRA)	-0.97889	0.8881	415	2752	100.0%	0.38 [0.07, 2.14]	?	?	?	?	+	+	+
**Total (95% CI)**			**415**	**2752**	**100.0%**	**0.38 [0.07, 2.14]**							

Heterogeneity: Not applicable

Test for overall effect: Z = 1.10 (P = 0.27)

Rate Ratio IV, Random, 95% CI

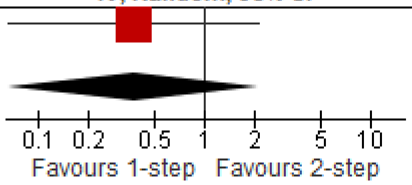

Risk of bias legend

(A) Random sequence generation (selection bias)

(B) Allocation concealment (selection bias)

(C) Blinding of participants and personnel (performance bias): Fracture outcomes

(D) Blinding of outcome assessment (detection bias): Fracture outcomes

(E) Incomplete outcome data (attrition bias): Fracture outcomes

(F) Selective reporting (reporting bias)

(G) Other bias

*The rate ratio is age-adjusted

Evidence Set 10: 1-step vs. 2-step; clinical fragility fractures (All eligible/Offer-to-screen)
Included studies: LaCroix 2005 – OPRA (women 60-80 years)

10A. GRADE Evidence Profile Table

Population	Certainty assessment							№ of patients		Effect		Certainty[‡]
	№ of studies	Study design	Risk of bias	Inconsistency	Indirectness	Imprecision	Other consideration	2-step[*]	1-step[†]	Relative Rate Ratio (95% CI)	Absolute (95% CI)	
**Women 60-80 years (LaCroix 2005 - OPRA)**												
**2-step event rate (9.4% or 94 per 1000)**	1	RCT	-1[a]	-0.5[b]	-1[c]	NC	NC	7282	1986	0.79 (0.66 to 0.94)	20.2 fewer (32.6 fewer to 5.8 fewer)	VERY LOW ⨁⊖⊖⊖

BMD: bone mineral density; CI: confidence interval; NC: no serious concerns; RCT: randomized controlled trial

[*] SCORE-based tool + BMD or SOF-based tool + BMD

[†] BMD

[‡] When our assessment of the certainty of evidence fell between levels, we assigned the level that best represented our actual certainty in the evidence, and based our conclusions on this the level of certainty.

Explanations:

[a] Risk of bias: Serious concern as the single study in the analysis is at risk of selection, performance, and detection biases.

[b] Inconsistency: Some concern about lack of evidence of consistency because there is only one study in the analysis.

[c] Indirectness: Serious concern because the 'non-pathological fractures' outcome is likely to include some non-clinical vertebral fractures due to the ascertainment method, and potentially other non-osteoporotic fractures. Recruitment method not typical (via letters), but effect on outcomes may be limited.

Evidence Set 10: 1-step vs. 2-step; clinical fragility fractures (All eligible/Offer-to-screen)
Included studies: LaCroix 2005 – OPRA (women 60-80 years)

10B. GRADE Summary of Findings Table

Outcome<br>No. participants (studies)	Relative effects, Rate Ratio (95% CI)	Anticipated absolute effects* (95% CI)			Certainty of evidence (GRADE)**	What happens?
		2-step screening†	1-step screening‡	Difference		
**Women 60-80 years (LaCroix 2005, OPRA)**						
Clinical fragility fractures (included all nonpathologic fractures)<br><br>Follow-up: mean 2.3 years<br><br>9,268 (1 RCT)	0.79 (0.66 to 0.94)	2-step event rate<br>96 per 1000	75.8 per 1000 (63.4 to 90.2)	20.2 fewer (32.6 fewer to 5.8 fewer)	VERY LOW<br>⊕⊕⊖⊖<br>due to risk of bias inconsistency, and indirectness[a-c]	The evidence is very uncertain.

BMD: bone mineral density; CI: confidence interval; RCT: randomized controlled trial

* The absolute effect (and its 95% CI) with 2-step screening (i.e. baseline rate) is based on the estimated risk in the 2-step screening group; the effect with 1-step screening is based on applying the relative effect of the intervention (and its 95% CI) to the effect in the 1-step group.

† SCORE-based tool + BMD or SOF-based tool + BMD

‡ BMD

** When our assessment of the certainty of evidence fell between levels, we assigned the level that best represented our actual certainty in the evidence, and based our conclusions on this the level of certainty.

Explanations:

[a] Risk of bias: Serious concern as the single study in the analysis is at risk of selection, performance, and detection biases.

[b] Inconsistency: Some concern about lack of evidence of consistency because there is only one study in the analysis.

[c] Indirectness: Serious concern because the 'non-pathological fractures' outcome is likely to include some non-clinical vertebral fractures due to the ascertainment method, and potentially other non-osteoporotic fractures. Recruitment method not typical (via letters), but effect on outcomes may be limited.

Evidence Set 10: 1-step vs. 2-step; clinical fragility fractures (All eligible/Offer-to-screen)
Included studies: LaCroix 2005 – OPRA (women 60-80 years)

10C. Forest Plot

Study or Subgroup	log[Rate Ratio]	SE	1-step Total	2-step Total	Weight	Rate Ratio IV, Random, 95% CI	Risk of Bias A	B	C	D	E	F	G
LaCroix 2005 (OPRA)	-0.2357	0.0909	1986	7282	100.0%	0.79 [0.66, 0.94]	?	?	?	?	+	+	+
**Total (95% CI)**			**1986**	**7282**	**100.0%**	**0.79 [0.66, 0.94]**							

Heterogeneity: Not applicable
Test for overall effect: Z = 2.59 (P = 0.010)

Rate Ratio
IV, Random, 95% CI

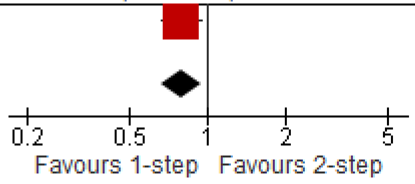

Risk of bias legend
(A) Random sequence generation (selection bias)
(B) Allocation concealment (selection bias)
(C) Blinding of participants and personnel (performance bias): Fracture outcomes
(D) Blinding of outcome assessment (detection bias): Fracture outcomes
(E) Incomplete outcome data (attrition bias): Fracture outcomes
(F) Selective reporting (reporting bias)
(G) Other bias

*The rate ratio is age-adjusted

Evidence Set 11: 1-step vs. 2-step; clinical fragility fractures (Acceptors of screening)
Included studies: LaCroix 2005 – OPRA (women 60-80 years)

11A. GRADE Evidence Profile Table

Population	Certainty assessment							№ of patients		Effect		Certainty[‡]
	№ of studies	Study design	Risk of bias	Inconsistency	Indirectness	Imprecision	Other consideration	2-step[*]	1-step[†]	Relative Rate Ratio (95% CI)	Absolute (95% CI)	
**Women 60-80 years (LaCroix 2005 - OPRA)**												
**2-step event rate (9.8% or 98 per 1000)**	1	RCT	-1[a]	-0.5[b]	-1[c]	-1[d]	NC	2752	415	0.91 (0.65 to 1.29)	8.9 fewer per 1000 (34.3 fewer to 28.4 more)	VERY LOW ⨁⊖⊖⊖

BMD: bone mineral density; CI: confidence interval; NC: no serious concerns; RCT: randomized controlled trial

[*] SCORE-based tool + BMD or SOF-based tool + BMD

[†] BMD

[‡] When our assessment of the certainty of evidence fell between levels, we assigned the level that best represented our actual certainty in the evidence, and based our conclusions on this the level of certainty.

Explanations:

[a] Risk of bias: Serious concern related to the potential for selection, performance and detection biases.

[b] Inconsistency: Some concern about lack of evidence of consistency because there is only one study in the analysis.

[c] Indirectness: Serious concern because the 'non-pathological fractures' outcome is likely to include some non-clinical vertebral fractures due to the ascertainment method, and potentially other non-osteoporotic fractures. Recruitment methods (letters) not typical but effect on the outcome may be limited.

[d] Imprecision: The confidence interval includes the potential for both important benefit and harm.

Evidence Set 11: 1-step vs. 2-step; clinical fragility fractures (Acceptors of screening)

Included studies: LaCroix 2005 – OPRA (women 60-80 years)

11B. GRADE Summary of Findings Table

Outcome<br>No. participants (studies)	Relative effects, Rate Ratio (95% CI)	Anticipated absolute effects* (95% CI)			Certainty of evidence (GRADE)**	What happens?
		2-step screening†	1-step screening‡	Difference		
**Women 60-80 years (LaCroix 2005, OPRA)**						
Hip fractures<br><br>Follow-up: mean 2.3 years<br><br>3,167 (1 RCT)	0.91 (0.65 to 1.29)	2-step event rate<br>98 per 1000	89.1 per 1000 (63.7 to 126.4)	8.9 fewer per 1000 (34.3 fewer to 28.4 more)	VERY LOW<br>⨁⊖⊖⊖<br>due to risk of bias inconsistency, indirectness and imprecision[a-d]	The evidence is very uncertain.

BMD: bone mineral density; CI: confidence interval; RCT: randomized controlled trial

* The absolute effect (and its 95% CI) with 2-step screening (i.e. baseline rate) is based on the estimated risk in the 2-step screening group; the effect with 1-step screening is based on applying the relative effect of the intervention (and its 95% CI) to the effect in the 1-step group.

† SCORE-based tool + BMD or SOF-based tool + BMD

‡ BMD

** When our assessment of the certainty of evidence fell between levels, we assigned the level that best represented our actual certainty in the evidence, and based our conclusions on this the level of certainty.

Explanations:

a Risk of bias: Serious concern related to the potential for selection, performance and detection biases.

b Inconsistency: Some concern about lack of evidence of consistency because there is only one study in the analysis.

c Indirectness: Serious concern because the 'non-pathological fractures' outcome is likely to include some non-clinical vertebral fractures due to the ascertainment method, and potentially other non-osteoporotic fractures. Recruitment methods (letters) not typical but effect on the outcome may be limited.

d Imprecision: The confidence interval includes the potential for both important benefit and harm.

Evidence Set 11: 1-step vs. 2-step; clinical fragility fractures (Acceptors of screening)

Included studies: LaCroix 2005 – OPRA (women 60-80 years)

11C. Forest Plot

Study or Subgroup	log[Rate Ratio]	SE	1-step Total	2-step Total	Weight	Rate Ratio IV, Random, 95% CI	Risk of Bias A	B	C	D	E	F	G
LaCroix 2005 (OPRA)	-0.091	0.1753	415	2752	100.0%	0.91 [0.65, 1.29]	?	?	?	?	+	+	+
**Total (95% CI)**			**415**	**2752**	**100.0%**	**0.91 [0.65, 1.29]**							
Heterogeneity: Not applicable													
Test for overall effect: Z = 0.52 (P = 0.60)													

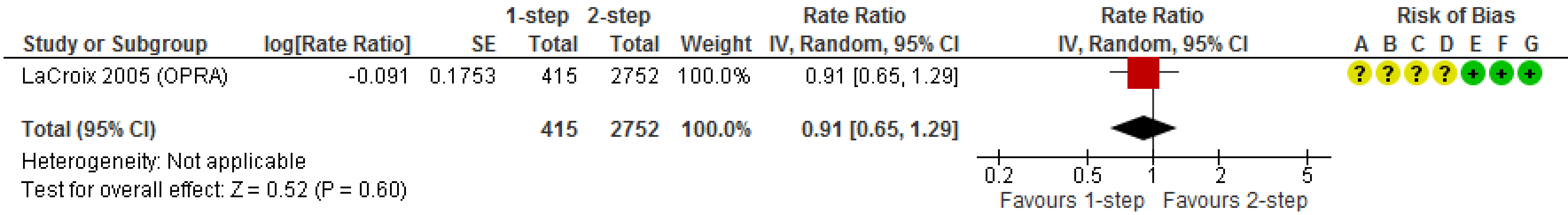

Risk of bias legend
(A) Random sequence generation (selection bias)
(B) Allocation concealment (selection bias)
(C) Blinding of participants and personnel (performance bias): Fracture outcomes
(D) Blinding of outcome assessment (detection bias): Fracture outcomes
(E) Incomplete outcome data (attrition bias): Fracture outcomes
(F) Selective reporting (reporting bias)
(G) Other bias

*The rate ratio is age-adjusted

Evidence Set 12: BMD vs. SCORE + BMD; hip fractures (All eligible/Offer-to-screen)
Included studies: LaCroix 2005 – OPRA (women 60-80 years)

12A. GRADE Evidence Profile Table

Population	Certainty assessment							№ of patients		Effect		Certainty*
	№ of studies	Study design	Risk of bias	Inconsistency	Indirectness	Imprecision	Other consideration	SCORE + BMD	BMD	Relative Rate Ratio (95% CI)	Absolute (95% CI)	
**Women 60-80 years (LaCroix 2005 - OPRA)**												
**SCORE + BMD event rate (0.9% or 9 per 1000)**	1	RCT	-1[a]	-0.5[b]	NC[c]	-1[d]	NC	1940	1986	0.94 (0.48 to 1.84)	0.5 fewer (4.7 fewer to 7.6 more)	VERY LOW ⊕⊖⊖⊖

BMD: bone mineral density; CI: confidence interval; NC: no serious concerns; RCT: randomized controlled trial; SCORE: Simple Calculated Osteoporosis Risk Estimation

* When our assessment of the certainty of evidence fell between levels, we assigned the level that best represented our actual certainty in the evidence, and based our conclusions on this the level of certainty.

Explanations:

[a] Risk of bias: Serious concern because the single study in the analysis is at risk of selection, performance, and detection biases.
[b] Inconsistency: Some concern about lack of evidence of consistency because there is only one study in the analysis.
[c] Indirectness: Recruitment method not typical (via letters), but effect on the outcome may be limited.
[d] Imprecision: The number of events does not meet the optimal information size (<300).

Evidence Set 12: BMD vs. SCORE + BMD; hip fractures (All eligible/Offer-to-screen)

Included studies: LaCroix 2005 – OPRA (women 60-80 years)

12B. GRADE Summary of Findings Table

Outcome<br>No. participants (studies)	Relative effects, Rate Ratio (95% CI)	Anticipated absolute effects* (95% CI)			Certainty of evidence (GRADE)**	What happens?
		SCORE + BMD	BMD	Difference		
**Women 60-80 years (LaCroix 2005, OPRA)**						
Hip fractures<br><br>Follow-up: mean 2.3 years<br><br>3,926 (1 RCT)	0.94 (0.48 to 1.84)	SCORE + BMD event rate			VERY LOW<br>⊕⊖⊖⊖<br>due to risk of bias inconsistency and imprecision[a-d]	The evidence is very uncertain.
		9 per 1000	8.5 per 1000 (4.3 to 16.6)	0.5 fewer (4.7 fewer to 7.6 more)		

BMD: bone mineral density; CI: confidence interval; RCT: randomized controlled trial; SCORE: Simple Calculated Osteoporosis Risk Estimation

* The absolute effect (and its 95% CI) with SCORE + BMD (i.e. baseline rate) is based on the estimated risk in the SCORE + BMD screening group; the effect with BMD only screening is based on applying the relative effect of the intervention (and its 95% CI) to the effect in the BMD only group.

** When our assessment of the certainty of evidence fell between levels, we assigned the level that best represented our actual certainty in the evidence, and based our conclusions on this the level of certainty.

Explanations:

[a] Risk of bias: Serious concern because the single study in the analysis is at risk of selection, performance, and detection biases.

[b] Inconsistency: Some concern about lack of evidence of consistency because there is only one study in the analysis.

[c] Indirectness: Recruitment method not typical (via letters), but unlikely to have affected the outcomes.

[d] Imprecision: The number of events does not meet the optimal information size (<300).

Evidence Set 12: BMD vs. SCORE + BMD; hip fractures (All eligible/Offer-to-screen)

Included studies: LaCroix 2005 – OPRA (women 60-80 years)

12C. Forest Plot

Study or Subgroup	log[Rate Ratio]	SE	BMD Total	SCORE + BMD Total	Weight	Rate Ratio IV, Random, 95% CI	Risk of Bias A	B	C	D	E	F	G
LaCroix 2005 (OPRA)	-0.0569	0.3406	1986	1940	100.0%	0.94 [0.48, 1.84]	?	?	?	?	+	+	+
**Total (95% CI)**			**1986**	**1940**	**100.0%**	**0.94 [0.48, 1.84]**							

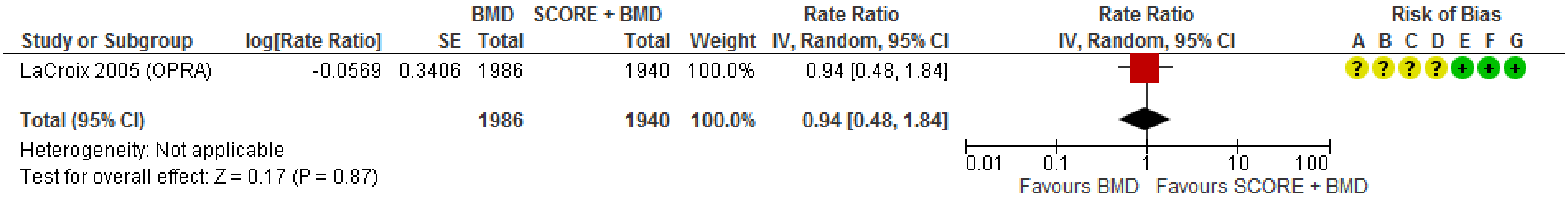

Heterogeneity: Not applicable

Test for overall effect: Z = 0.17 (P = 0.87)

Risk of bias legend

(A) Random sequence generation (selection bias)

(B) Allocation concealment (selection bias)

(C) Blinding of participants and personnel (performance bias): Fracture outcomes

(D) Blinding of outcome assessment (detection bias): Fracture outcomes

(E) Incomplete outcome data (attrition bias): Fracture outcomes

(F) Selective reporting (reporting bias)

(G) Other bias

*The rate ratio is age-adjusted

Evidence Set 13: BMD vs. SCORE + BMD; hip fractures (Acceptors of screening)

Included studies: LaCroix 2005 – OPRA (women 60-80 years)

13A. GRADE Evidence Profile Table

Population	Certainty assessment							№ of patients		Effect		Certainty*
	№ of studies	Study design	Risk of bias	Inconsistency	Indirectness	Imprecision	Other consideration	SCORE + BMD	BMD	Relative Rate Ratio (95% CI)	Absolute (95% CI)	
**Women 60-80 years (LaCroix 2005 - OPRA)**												
**SCORE + BMD event rate (0.8% or per 8 per 1000)**	1	RCT	-1[a]	-0.5[b]	-0.5[c]	-1[c]	NC	576	415	0.40 (0.06 to 2.78)	4.8 fewer (7.5 fewer to 14.2 more)	VERY LOW ⊕⊖⊖⊖

BMD: bone mineral density; CI: confidence interval; NC: no serious concerns; RCT: randomized controlled trial; SCORE: Simple Calculated Osteoporosis Risk Estimation

* When our assessment of the certainty of evidence fell between levels, we assigned the level that best represented our actual certainty in the evidence, and based our conclusions on this the level of certainty.

Explanations:

[a] Risk of bias: Concern about selection bias because the per protocol population may differ from the randomized sample. Some concern about performance and detection biases.

[b] Inconsistency: Some concern about lack of evidence of consistency because there is only one study in the analysis.

[c] Indirectness: Recruitment methods (letters) not typical but effect on the outcome may be limited.

[c] Imprecision: The number of events does not meet the optimal information size (<300).

Evidence Set 13: BMD vs. SCORE + BMD; hip fractures (Acceptors of screening)

Included studies: LaCroix 2005 – OPRA (women 60-80 years)

13B. GRADE Summary of Findings Table

Outcome<br>No. participants (studies)	Relative effects, Rate Ratio (95% CI)	Anticipated absolute effects[*] (95% CI)			Certainty of evidence (GRADE)[**]	What happens?
		SCORE + BMD	BMD	Difference		
**Women 60-80 years (LaCroix 2005, OPRA)**						
Hip fractures<br><br>Follow-up: mean 2.3 years<br><br>991 (1 RCT)	0.40 (0.06 to 2.78)	SCORE + BMD event rate<br>8 per 1000	3.2 per 1000 (0.5 to 22.2)	4.8 fewer (7.5 fewer to 14.2 more)	VERY LOW<br>⊕⊖⊖⊖<br>due to risk of bias inconsistency, indirectness, and imprecision[a-d]	The evidence is very uncertain.

BMD: bone mineral density; CI: confidence interval; RCT: randomized controlled trial; SCORE: Simple Calculated Osteoporosis Risk Estimation

[*] The absolute effect (and its 95% CI) with SCORE + BMD (i.e. baseline rate) is based on the estimated risk in the SCORE + BMD screening group; the effect with BMD only screening is based on applying the relative effect of the intervention (and its 95% CI) to the effect in the BMD only group

[**] When our assessment of the certainty of evidence fell between levels, we assigned the level that best represented our actual certainty in the evidence, and based our conclusions on this the level of certainty.

Explanations:

[a] Risk of bias: Concern about selection bias because the per protocol population may differ from the randomized sample. Some concern about performance and detection biases.

[b] Inconsistency: Some concern about lack of evidence of consistency because there is only one study in the analysis.

[c] Indirectness: Recruitment methods (letters) not typical but effect on the outcome may be limited.

[c] Imprecision: The number of events does not meet the optimal information size (<300).

Evidence Set 13: BMD vs. SCORE + BMD; hip fractures (Acceptors of screening)

Included studies: LaCroix 2005 – OPRA (women 60-80 years)

13C. Forest Plot

Study or Subgroup	log[Rate Ratio]	SE	BMD Total	SCORE + BMD Total	Weight	Rate Ratio IV, Random, 95% CI	Risk of Bias A	B	C	D	E	F	G
LaCroix 2005 (OPRA)	-0.904	0.9821	415	576	100.0%	0.40 [0.06, 2.78]	?	?	?	?	+	+	+
**Total (95% CI)**			**415**	**576**	**100.0%**	**0.40 [0.06, 2.78]**							

Heterogeneity: Not applicable

Test for overall effect: Z = 0.92 (P = 0.36)

Rate Ratio IV, Random, 95% CI

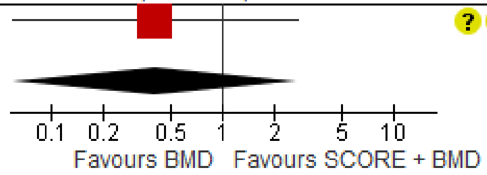

Risk of bias legend

(A) Random sequence generation (selection bias)

(B) Allocation concealment (selection bias)

(C) Blinding of participants and personnel (performance bias): Fracture outcomes

(D) Blinding of outcome assessment (detection bias): Fracture outcomes

(E) Incomplete outcome data (attrition bias): Fracture outcomes

(F) Selective reporting (reporting bias)

(G) Other bias

*The rate ratio is age-adjusted

Evidence Set 14: BMD vs. SCORE + BMD; clinical fragility fractures (All eligible/Offer-to-screen)
Included studies: LaCroix 2005 – OPRA (women 60-80 years)

14A. GRADE Evidence Profile Table

Population	Certainty assessment							№ of patients		Effect		Certainty*
	№ of studies	Study design	Risk of bias	Inconsistency	Indirectness	Imprecision	Other consideration	SCORE + BMD	BMD	Relative Rate Ratio (95% CI)	Absolute (95% CI)	
**Women 60-80 years (LaCroix 2005 - OPRA)**												
**SCORE + BMD event rate (9.9% or 99 per 1000)**	1	RCT	-1[a]	-0.5[b]	-1[c]	NC	NC	1940	1986	0.75 (0.60 to 0.92)	24.7 fewer per 1000 (39.6 fewer to 7.9 fewer)	VERY LOW ⨁⊖⊖⊖

BMD: bone mineral density; CI: confidence interval; NC: no serious concerns; RCT: randomized controlled trial; SCORE: Simple Calculated Osteoporosis Risk Estimation

* When our assessment of the certainty of evidence fell between levels, we assigned the level that best represented our actual certainty in the evidence, and based our conclusions on this the level of certainty.

Explanations:

[a] Risk of bias: Serious concern because the single included study is at risk of selection, performance, and detection biases.

[b] Inconsistency: Some concern about lack of evidence of consistency because there is only one study in the analysis.

[c] Indirectness: Serious concern because the 'non-pathological fractures' outcome is likely to include some non-clinical vertebral fractures due to the ascertainment method, and potentially other non-osteoporotic fractures. Recruitment method not typical (via letters), but effect on the outcomes may be limited.

Evidence Set 14: BMD vs. SCORE + BMD; clinical fragility fractures (All eligible/Offer-to-screen)
Included studies: LaCroix 2005 – OPRA (women 60-80 years)

14B. GRADE Summary of Findings Table

Outcome<br>No. participants (studies)	Relative effects, Rate Ratio (95% CI)	Anticipated absolute effects* (95% CI)			Certainty of evidence (GRADE)**	What happens?
		SCORE + BMD	BMD	Difference		
**Women 60-80 years (LaCroix 2005, OPRA)**						
Hip fractures<br><br>Follow-up: mean 2.3 years<br><br>3,926 (1 RCT)	0.75 (0.60 to 0.92)	SCORE + BMD event rate			VERY LOW<br>⊕⊖⊖⊖<br>due to risk of bias inconsistency, and indirectness [a-c]	The evidence is very uncertain.
		99 per 1000	74.3 per 1000 (59.4 to 91.1)	24.7 fewer per 1000 (39.6 fewer to 7.9 fewer)		

BMD: bone mineral density; CI: confidence interval; RCT: randomized controlled trial; SCORE: Simple Calculated Osteoporosis Risk Estimation

* The absolute effect (and its 95% CI) with SCORE + BMD (i.e. baseline rate) is based on the estimated risk in the SCORE + BMD screening group; the effect with BMD only screening is based on applying the relative effect of the intervention (and its 95% CI) to the effect in the BMD only group

** When our assessment of the certainty of evidence fell between levels, we assigned the level that best represented our actual certainty in the evidence, and based our conclusions on this the level of certainty.

Explanations:

[a] Risk of bias: Serious concern because the single included study is at risk of selection, performance, and detection biases.

[b] Inconsistency: Some concern about lack of evidence of consistency because there is only one study in the analysis.

[c] Indirectness: Serious concern because the 'non-pathological fractures' outcome is likely to include some non-clinical vertebral fractures due to the ascertainment method, and potentially other non-osteoporotic fractures. Recruitment method not typical (via letters), but effect on the outcomes may be limited.

Evidence Set 14: BMD vs. SCORE + BMD; clinical fragility fractures (All eligible/Offer-to-screen)

Included studies: LaCroix 2005 – OPRA (women 60-80 years)

14C. Forest Plot

Study or Subgroup	log[Rate Ratio]	SE	BMD Total	SCORE + BMD Total	Weight	Rate Ratio IV, Random, 95% CI	Risk of Bias A	B	C	D	E	F	G
LaCroix 2005 (OPRA)	-0.294	0.1094	1986	1940	100.0%	0.75 [0.60, 0.92]	?	?	?	?	+	+	+
**Total (95% CI)**			**1986**	**1940**	**100.0%**	**0.75 [0.60, 0.92]**							

Heterogeneity: Not applicable

Test for overall effect: Z = 2.69 (P = 0.007)

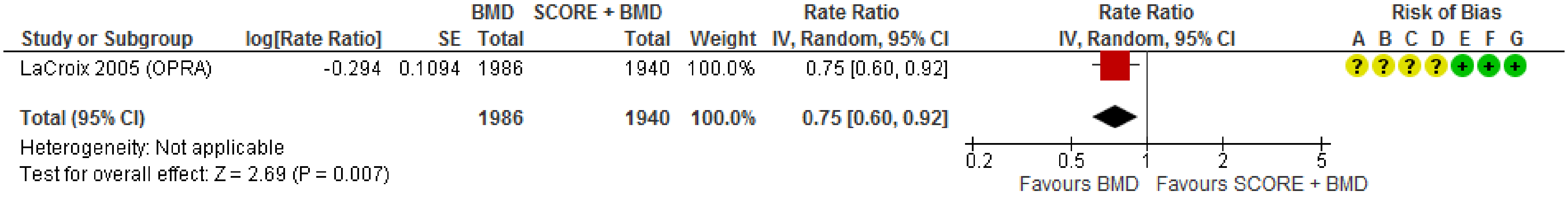

Risk of bias legend

(A) Random sequence generation (selection bias)

(B) Allocation concealment (selection bias)

(C) Blinding of participants and personnel (performance bias): Fracture outcomes

(D) Blinding of outcome assessment (detection bias): Fracture outcomes

(E) Incomplete outcome data (attrition bias): Fracture outcomes

(F) Selective reporting (reporting bias)

(G) Other bias

*The rate ratio is age-adjusted

Evidence Set 15: BMD vs. SCORE + BMD; clinical fragility fractures (Acceptors of screening)
Included studies: LaCroix 2005 – OPRA (women 60-80 years)

15A. GRADE Evidence Profile Table

Population	Certainty assessment							№ of patients		Effect		Certainty*
	№ of studies	Study design	Risk of bias	Inconsistency	Indirectness	Imprecision	Other consideration	SCORE + BMD	BMD	Relative Rate Ratio (95% CI)	Absolute (95% CI)	
**Women 60-80 years (LaCroix 2005 - OPRA)**												
**SCORE + BMD event rate (11.6% or 116 per 1000)**	1	RCT	-1[a]	-0.5[b]	-1[c]	-1[d]	NC	576	415	0.77 (0.51 to 1.15)	26.7 fewer (56.8 fewer to 17.4 more)	VERY LOW ⨁⊖⊖⊖

BMD: bone mineral density; CI: confidence interval; NC: no serious concerns; RCT: randomized controlled trial; SCORE: Simple Calculated Osteoporosis Risk Estimation

* When our assessment of the certainty of evidence fell between levels, we assigned the level that best represented our actual certainty in the evidence, and based our conclusions on this the level of certainty.

Explanations:

[a] Risk of bias: Concern about selection bias because the per protocol population may differ from the randomized sample. Some concern about performance and detection biases.

[b] Inconsistency: Some concern about lack of evidence of consistency because there is only one study in the analysis.

[c] Indirectness: Serious concern because the 'non-pathological fractures' outcome is likely to include some non-clinical vertebral fractures due to the ascertainment method, and potentially other non-osteoporotic fractures. Recruitment method not typical (via letters), effect on the outcome may be limited.

[d] Imprecision: The confidence interval includes the potential for important benefit and harm.

Evidence Set 15: BMD vs. SCORE + BMD; clinical fragility fractures (Acceptors of screening)

Included studies: LaCroix 2005 – OPRA (women 60-80 years)

15B. GRADE Summary of Findings Table

Outcome<br>No. participants (studies)	Relative effects, Rate Ratio (95% CI)	Anticipated absolute effects* (95% CI)			Certainty of evidence (GRADE)**	What happens?
		SOF + BMD	BMD	Difference		
**Women 60-80 years (LaCroix 2005, OPRA)**						
Hip fractures<br><br>Follow-up: mean 2.3 years<br><br>991 (1 RCT)	0.77 (0.51 to 1.15)	SCORE + BMD event rate			VERY LOW<br>⊕⊖⊖⊖<br>due to risk of bias inconsistency, indirectness and imprecision[a-d]	The evidence is very uncertain.
		116 per 1000	89.3 per 1000 (59.2 to 133.4)	26.7 fewer (56.8 fewer to 17.4 more)		

BMD: bone mineral density; CI: confidence interval; RCT: randomized controlled trial; SCORE: Simple Calculated Osteoporosis Risk Estimation

* The absolute effect (and its 95% CI) with SCORE + BMD (i.e. baseline rate) is based on the estimated risk in the SCORE + BMD screening group; the effect with BMD only screening is based on applying the relative effect of the intervention (and its 95% CI) to the effect in the BMD only group

** When our assessment of the certainty of evidence fell between levels, we assigned the level that best represented our actual certainty in the evidence, and based our conclusions on this the level of certainty.

Explanations:

[a] Risk of bias: Concern about selection bias because the per protocol population may differ from the randomized sample. Some concern about performance and detection biases.

[b] Inconsistency: Some concern about lack of evidence of consistency because there is only one study in the analysis.

[c] Indirectness: Serious concern because the 'non-pathological fractures' outcome is likely to include some non-clinical vertebral fractures due to the ascertainment method, and potentially other non-osteoporotic fractures. Recruitment method not typical (via letters), effect on the outcome may be limited.

[d] Imprecision: The confidence interval includes the potential for important benefit and harm.

Evidence Set 15: BMD vs. SCORE + BMD; clinical fragility fractures (Acceptors of screening)

Included studies: LaCroix 2005 – OPRA (women 60-80 years)

15C. Forest Plot

Study or Subgroup	log[Rate Ratio]	SE	BMD Total	SCORE + BMD Total	Weight	Rate Ratio IV, Random, 95% CI	Rate Ratio IV, Random, 95% CI	Risk of Bias A B C D E F G
LaCroix 2005 (OPRA)	-0.265	0.2047	415	576	100.0%	0.77 [0.51, 1.15]		? ? ? ? + + +
**Total (95% CI)**			**415**	**576**	**100.0%**	**0.77 [0.51, 1.15]**		
Heterogeneity: Not applicable								
Test for overall effect: Z = 1.29 (P = 0.20)								

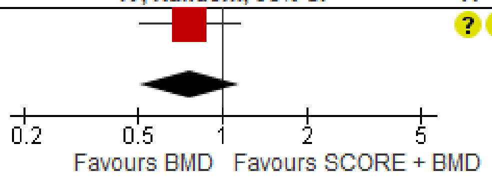

Risk of bias legend

(A) Random sequence generation (selection bias)
(B) Allocation concealment (selection bias)
(C) Blinding of participants and personnel (performance bias): Fracture outcomes
(D) Blinding of outcome assessment (detection bias): Fracture outcomes
(E) Incomplete outcome data (attrition bias): Fracture outcomes
(F) Selective reporting (reporting bias)
(G) Other bias

*The rate ratio is age-adjusted

Evidence Set 16: BMD vs. SOF + BMD; hip fractures (All eligible/Offer-to-screen)
Included studies: LaCroix 2005 – OPRA (women 60-80 years)

16A. GRADE Evidence Profile Table

Population	Certainty assessment							№ of patients		Effect		Certainty*
	№ of studies	Study design	Risk of bias	Inconsistency	Indirectness	Imprecision	Other consideration	SOF + BMD	BMD	Relative Rate Ratio (95% CI)	Absolute (95% CI)	
**Women 60-80 years (LaCroix 2005 - OPRA)**												
**SOF + BMD event rate (1.3% or 13 per 1000)**	1	RCT	-1[a]	-0.5[b]	NC[c]	-1[d]	NC	5342	1986	0.64 (0.38 to 1.09)	4.7 fewer per 1000 (8.1 fewer to 1.2 more)	VERY LOW ⨁⊖⊖⊖

BMD: bone mineral density; CI: confidence interval; NC: no serious concerns; RCT: randomized controlled trial; SOF: Study of Osteoporotic Fractures
* When our assessment of the certainty of evidence fell between levels, we assigned the level that best represented our actual certainty in the evidence, and based our conclusions on this the level of certainty.

Explanations:
[a] Risk of bias: Serious concern because the single included study is at risk of selection, performance, and detection biases.
[b] Inconsistency: Some concern about lack of evidence of consistency because there is only one study in the analysis.
[c] Indirectness: Recruitment method not typical (via letters), but effect on the outcomes may be limited.
[d] Imprecision: The number of events does not meet the optimal information size (<300).

Evidence Set 16: BMD vs. SOF + BMD; hip fractures (All eligible/Offer-to-screen)

Included studies: LaCroix 2005 – OPRA (women 60-80 years)

16B. GRADE Summary of Findings Table

Outcome<br>No. participants (studies)	Relative effects, Rate Ratio (95% CI)	Anticipated absolute effects* (95% CI)			Certainty of evidence (GRADE)**	What happens?
		SOF + BMD	BMD	Difference		
**Women 60-80 years (LaCroix 2005, OPRA)**						
Hip fractures<br><br>Follow-up: mean 2.3 years<br><br>7,328 (1 RCT)	0.64 (0.38 to 1.09)	SOF + BMD event rate			VERY LOW<br>⊕⊖⊖⊖<br>due to risk of bias inconsistency and imprecision[a-d]	The evidence is very uncertain.
		13 per 1000	8.3 per 1000 (4.9 to 14.2)	4.7 fewer per 1000 (8.1 fewer to 1.2 more)		

BMD: bone mineral density; CI: confidence interval; RCT: randomized controlled trial; SOF: Study of Osteoporotic Fractures

* The absolute effect (and its 95% CI) with SOF + BMD (i.e. baseline rate) is based on the estimated risk in the SOF + BMD screening group; the effect with BMD only screening is based on applying the relative effect of the intervention (and its 95% CI) to the effect in the BMD only group.

** When our assessment of the certainty of evidence fell between levels, we assigned the level that best represented our actual certainty in the evidence, and based our conclusions on this the level of certainty.

Explanations:

[a] Risk of bias: Serious concern because the single included study is at risk of selection, performance, and detection biases.

[b] Inconsistency: Some concern about lack of evidence of consistency because there is only one study in the analysis.

[c] Indirectness: Recruitment method not typical (via letters), but effect on the outcomes may be limited.

[d] Imprecision: The number of events does not meet the optimal information size (<300).

Evidence Set 16: BMD vs. SOF + BMD; hip fractures (All eligible/Offer-to-screen)
Included studies: LaCroix 2005 – OPRA (women 60-80 years)

16C. Forest Plot

Study or Subgroup	log[Rate Ratio]	SE	BMD Total	SOF + BMD Total	Weight	Rate Ratio IV, Random, 95% CI	Risk of Bias A	B	C	D	E	F	G
LaCroix 2005 (OPRA)	-0.4438	0.2702	1986	5342	100.0%	0.64 [0.38, 1.09]	?	?	?	?	+	+	+
**Total (95% CI)**			**1986**	**5342**	**100.0%**	**0.64 [0.38, 1.09]**							

Heterogeneity: Not applicable
Test for overall effect: Z = 1.64 (P = 0.10)

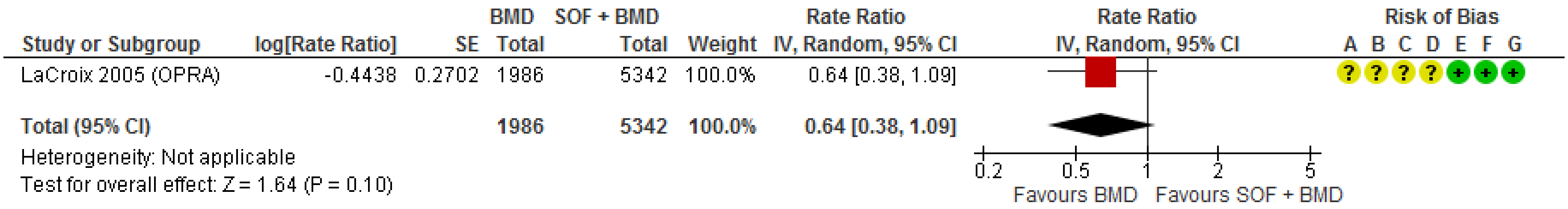

Risk of bias legend
(A) Random sequence generation (selection bias)
(B) Allocation concealment (selection bias)
(C) Blinding of participants and personnel (performance bias): Fracture outcomes
(D) Blinding of outcome assessment (detection bias): Fracture outcomes
(E) Incomplete outcome data (attrition bias): Fracture outcomes
(F) Selective reporting (reporting bias)
(G) Other bias

*The rate ratio is age-adjusted

Evidence Set 17: BMD vs. SOF + BMD; hip fractures (Acceptors of screening)
Included studies: LaCroix 2005 – OPRA (women 60-80 years)

17A. GRADE Evidence Profile Table

Population	Certainty assessment							№ of patients		Effect		Certainty*
	№ of studies	Study design	Risk of bias	Inconsistency	Indirectness	Imprecision	Other consideration	SOF + BMD	BMD	Relative Rate Ratio (95% CI)	Absolute (95% CI)	
**Women 60-80 years (LaCroix 2005 - OPRA)**												
**SOF + BMD event rate (0.9% or 9 per 1000)**	1	RCT	-1[a]	-0.5[b]	NC[c]	-1[d]	NC	2176	415	0.37 (0.06 to 2.12)	5.7 fewer per 1000 (8.5 fewer to 10.1 more)	VERY LOW ⊕⊖⊖⊖

BMD: bone mineral density; CI: confidence interval; NC: no serious concerns; RCT: randomized controlled trial; SOF: Study of Osteoporotic Fractures
* When our assessment of the certainty of evidence fell between levels, we assigned the level that best represented our actual certainty in the evidence, and based our conclusions on this the level of certainty.

Explanations:
[a] Risk of bias: Concern about selection bias because the per protocol population may differ from the randomized sample. Some concern about performance and detection biases.
[b] Inconsistency: Some concern about lack of evidence of consistency because there is only one study in the analysis.
[c] Indirectness: Recruitment method not typical (via letters), but effect on the outcomes may be limited.
[d] Imprecision: The number of events does not meet the optimal information size (<300). The confidence interval includes potential for both important benefit and harm.

Evidence Set 17: BMD vs. SOF + BMD; hip fractures (Acceptors of screening)
Included studies: LaCroix 2005 – OPRA (women 60-80 years)

17B. GRADE Summary of Findings Table

Outcome<br>No. participants (studies)	Relative effects, Rate Ratio (95% CI)	Anticipated absolute effects* (95% CI)			Certainty of evidence (GRADE)**	What happens?
		SOF + BMD	BMD	Difference		
**Women 60-80 years (LaCroix 2005, OPRA)**						
Hip fractures<br><br>Follow-up: mean 2.3 years<br><br>2,591 (1 RCT)	0.37 (0.06 to 2.12)	SOF + BMD event rate			VERY LOW ⨁⊖⊖⊖ due to risk of bias, inconsistency, and imprecision[a-d]	The evidence is very uncertain.
		9 per 1000	3.3 per 1000 (0.5 to 19.1)	5.7 fewer per 1000 (8.5 fewer to 10.1 more)		

BMD: bone mineral density; CI: confidence interval; RCT: randomized controlled trial; SOF: Study of Osteoporotic Fractures

* The absolute effect (and its 95% CI) with SOF + BMD (i.e. baseline rate) is based on the estimated risk in the SOF + BMD screening group; the effect with BMD only screening is based on applying the relative effect of the intervention (and its 95% CI) to the effect in the BMD only group.

** When our assessment of the certainty of evidence fell between levels, we assigned the level that best represented our actual certainty in the evidence, and based our conclusions on this the level of certainty.

Explanations:

[a] Risk of bias: Concern about selection bias because the per protocol population may differ from the randomized sample. Some concern about performance and detection biases.

[b] Inconsistency: Some concern about lack of evidence of consistency because there is only one study in the analysis.

[c] Indirectness: Recruitment method not typical (via letters), but effect on the outcomes may be limited.

[d] Imprecision: The number of events does not meet the optimal information size (<300). The confidence interval includes potential for both important benefit and harm.

Evidence Set 17: BMD vs. SOF + BMD; hip fractures (Acceptors of screening)

Included studies: LaCroix 2005 – OPRA (women 60-80 years)

17C. Forest Plot

Study or Subgroup	log[Rate Ratio]	SE	BMD Total	SOF + BMD Total	Weight	Rate Ratio IV, Random, 95% CI	Rate Ratio IV, Random, 95% CI	Risk of Bias A B C D E F G
LaCroix 2005 (OPRA)	-0.9985	0.8938	415	2176	100.0%	0.37 [0.06, 2.12]		? ? ? ? + + +
**Total (95% CI)**			**415**	**2176**	**100.0%**	**0.37 [0.06, 2.12]**		
Heterogeneity: Not applicable								
Test for overall effect: Z = 1.12 (P = 0.26)								

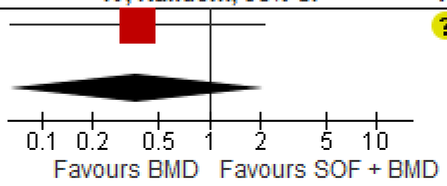

Risk of bias legend
(A) Random sequence generation (selection bias)
(B) Allocation concealment (selection bias)
(C) Blinding of participants and personnel (performance bias): Fracture outcomes
(D) Blinding of outcome assessment (detection bias): Fracture outcomes
(E) Incomplete outcome data (attrition bias): Fracture outcomes
(F) Selective reporting (reporting bias)
(G) Other bias

*The rate ratio is age-adjusted

Evidence Set 18: BMD vs. SOF + BMD; clinical fragility fractures (All eligible/offer-to-screen)
Included studies: LaCroix 2005 – OPRA (women 60-80 years)

18A. GRADE Evidence Profile Table

Population	Certainty assessment							№ of patients		Effect		Certainty*
	№ of studies	Study design	Risk of bias	Inconsistency	Indirectness	Imprecision	Other consideration	SCORE + BMD	BMD	Relative Rate Ratio (95% CI)	Absolute (95% CI)	
**Women 60-80 years (LaCroix 2005 - OPRA)**												
**SOF + BMD event rate (9.2% or 92 per 1000)**	1	RCT	-1[a]	-0.5[b]	-1[c]	NC	NC	5342	1986	0.81 (0.67 to 0.97)	17.5 fewer in 1000 (30.4 fewer to 2.8 fewer)	VERY LOW ⨁⨁⊖⊖

BMD: bone mineral density; CI: confidence interval; NC: no serious concerns; RCT: randomized controlled trial; SCORE: Simple Calculated Osteoporosis Risk Estimation

* When our assessment of the certainty of evidence fell between levels, we assigned the level that best represented our actual certainty in the evidence, and based our conclusions on this the level of certainty.

Explanations:

[a] Risk of bias: Serious concern as the single included study is at risk of selection, performance, and detection biases.

[b] Inconsistency: Some concern about lack of evidence of consistency because there is only one study in the analysis.

[c] Indirectness: Serious concern because the 'non-pathological fractures' outcome is likely to include some non-clinical vertebral fractures due to the ascertainment method, and potentially other non-osteoporotic fractures. Recruitment method not typical (via letters), effect on the outcome may be limited.

Evidence Set 18: BMD vs. SOF + BMD; clinical fragility fractures (All eligible/offer-to-screen)
Included studies: LaCroix 2005 – OPRA (women 60-80 years)

18B. GRADE Summary of Findings Table

Outcome<br>No. participants (studies)	Relative effects, Rate Ratio (95% CI)	Anticipated absolute effects* (95% CI)			Certainty of evidence (GRADE)**	What happens?
		SOF + BMD	BMD	Difference		
**Women 60-80 years (LaCroix 2005, OPRA)**						
All clinical fragility fractures<br><br>Follow-up: mean 2.3 years<br><br>7,328 (1 RCT)	0.81 (0.67 to 0.97)	SOF + BMD event rate<br>92 per 1000	74.5 per 1000 (61.6 to 89.2)	17.5 fewer in 1000 (30.4 fewer to 2.8 fewer)	VERY LOW<br>⊕⊖⊖⊖<br>due to risk of bias inconsistency, and indirectness[a-c]	The evidence is very uncertain.

BMD: bone mineral density; CI: confidence interval; RCT: randomized controlled trial; SOF: Study of Osteoporotic Fractures
* The absolute effect (and its 95% CI) with SOF + BMD (i.e. baseline rate) is based on the estimated risk in the SOF + BMD screening group; the effect with BMD only screening is based on applying the relative effect of the intervention (and its 95% CI) to the effect in the BMD only group
** When our assessment of the certainty of evidence fell between levels, we assigned the level that best represented our actual certainty in the evidence, and based our conclusions on this the level of certainty.

Explanations:
[a] Risk of bias: Serious concern as the single included study is at risk of selection, performance, and detection biases.
[b] Inconsistency: Some concern about lack of evidence of consistency because there is only one study in the analysis.
[c] Indirectness: Serious concern because the 'non-pathological fractures' outcome is likely to include some non-clinical vertebral fractures due to the ascertainment method, and potentially other non-osteoporotic fractures. Recruitment method not typical (via letters), effect on the outcome may be limited.

Evidence Set 18: BMD vs. SOF + BMD; clinical fragility fractures (All eligible/offer-to-screen)

Included studies: LaCroix 2005 – OPRA (women 60-80 years)

18C. Forest Plot

Study or Subgroup	log[Rate Ratio]	SE	BMD Total	SOF + BMD Total	Weight	Rate Ratio IV, Random, 95% CI	Risk of Bias A	B	C	D	E	F	G
LaCroix 2005 (OPRA)	-0.2137	0.094	1986	5342	100.0%	0.81 [0.67, 0.97]	?	?	?	?	+	+	+
**Total (95% CI)**			**1986**	**5342**	**100.0%**	**0.81 [0.67, 0.97]**							

Heterogeneity: Not applicable

Test for overall effect: Z = 2.27 (P = 0.02)

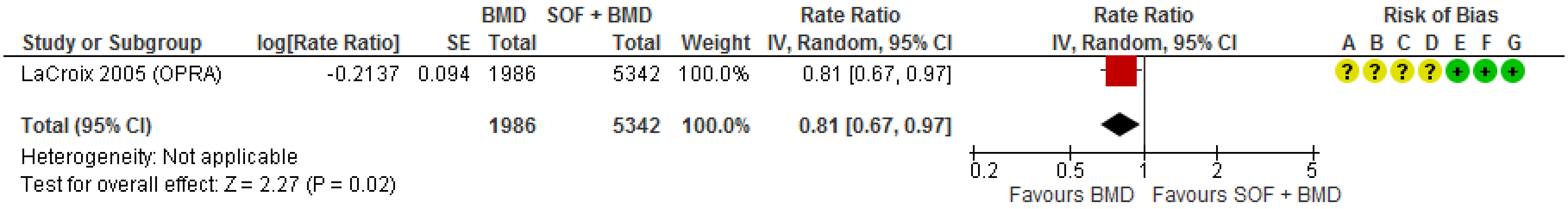

Risk of bias legend

(A) Random sequence generation (selection bias)

(B) Allocation concealment (selection bias)

(C) Blinding of participants and personnel (performance bias): Fracture outcomes

(D) Blinding of outcome assessment (detection bias): Fracture outcomes

(E) Incomplete outcome data (attrition bias): Fracture outcomes

(F) Selective reporting (reporting bias)

(G) Other bias

*The rate ratio is age-adjusted

Evidence Set 19: BMD vs. SOF + BMD; all clinical fractures (Acceptors of screening)
Included studies: LaCroix 2005 – OPRA (women 60-80 years)

19A. GRADE Evidence Profile Table

Population	Certainty assessment							№ of patients		Effect		Certainty*
	№ of studies	Study design	Risk of bias	Inconsistency	Indirectness	Imprecision	Other consideration	SOF + BMD	BMD	Relative Rate Ratio (95% CI)	Absolute (95% CI)	
**Women 60-80 years (LaCroix 2005 - OPRA)**												
**SOF + BMD event rate (9.3% or 93 per 1000)**	1	RCT	-1[a]	-0.5[b]	-1[c]	-1[d]	NC	576	415	0.96 (0.68 to 1.37)	3.8 fewer per 1000 (29.8 fewer to 34.4 more)	VERY LOW ⨁⊖⊖⊖

BMD: bone mineral density; CI: confidence interval; NC: no serious concerns; RCT: randomized controlled trial; SOF: Study of Osteoporotic Fractures
* When our assessment of the certainty of evidence fell between levels, we assigned the level that best represented our actual certainty in the evidence, and based our conclusions on this the level of certainty.

Explanations:
[a] Risk of bias: Serious concern about selection bias because the per protocol population may differ from the randomized sample. Some concern about performance and detection biases.
[b] Inconsistency: Some concern about lack of evidence of consistency because there is only one study in the analysis.
[c] Indirectness: Serious concern because the 'non-pathological fractures' outcome is likely to include some non-clinical vertebral fractures due to the ascertainment method, and potentially other non-osteoporotic fractures. Recruitment method not typical (via letters), effect on the outcome may be limited.
[d] Imprecision: The confidence interval includes potential for both important benefit and harm.

Evidence Set 19: BMD vs. SOF + BMD; all clinical fractures (Acceptors of screening)
Included studies: LaCroix 2005 – OPRA (women 60-80 years)

19B. GRADE Summary of Findings Table

Outcome<br>No. participants (studies)	Relative effects, Rate Ratio (95% CI)	Anticipated absolute effects* (95% CI)			Certainty of evidence (GRADE)**	What happens?
		SOF + BMD	BMD	Difference		
**Women 60-80 years (LaCroix 2005, OPRA)**						
All clinical fragility fractures<br><br>Follow-up: mean 2.3 years<br><br>2,591 (1 RCT)	0.96 (0.68 to 1.37)	SOF + BMD event rate			VERY LOW<br>⊕⊖⊖⊖<br>due to risk of bias inconsistency, indirectness and imprecision[a-d]	The evidence is very uncertain.
		93 per 1000	89.2 per 1000 (63.2 to 127.4)	3.8 fewer per 1000 (29.8 fewer to 34.4 more)		

BMD: bone mineral density; CI: confidence interval; RCT: randomized controlled trial; SOF: Study of Osteoporotic Fractures
* The absolute effect (and its 95% CI) with SOF + BMD (i.e. baseline rate) is based on the estimated risk in the SOF + BMD screening group; the effect with BMD only screening is based on applying the relative effect of the intervention (and its 95% CI) to the effect in the BMD only group
** When our assessment of the certainty of evidence fell between levels, we assigned the level that best represented our actual certainty in the evidence, and based our conclusions on this the level of certainty.

Explanations:
[a] Risk of bias: Serious concern about selection bias because the per protocol population may differ from the randomized sample. Some concern about performance and detection biases.
[b] Inconsistency: Some concern about lack of evidence of consistency because there is only one study in the analysis.
[c] Indirectness: Serious concern because the 'non-pathological fractures' outcome is likely to include some non-clinical vertebral fractures due to the ascertainment method, and potentially other non-osteoporotic fractures. Recruitment method not typical (via letters), effect on the outcome may be limited.
[d] Imprecision: The confidence interval includes potential for both important benefit and harm.

Evidence Set 19: BMD vs. SOF + BMD; all clinical fractures (Acceptors of screening)

Included studies: LaCroix 2005 – OPRA (women 60-80 years)

19C. Forest Plot

Study or Subgroup	log[Rate Ratio]	SE	BMD Total	SOF + BMD Total	Weight	Rate Ratio IV, Random, 95% CI	Risk of Bias A	B	C	D	E	F	G
LaCroix 2005 (OPRA)	-0.0385	0.1788	415	2176	100.0%	0.96 [0.68, 1.37]	?	?	?	?	+	+	+
**Total (95% CI)**			**415**	**2176**	**100.0%**	**0.96 [0.68, 1.37]**							

Heterogeneity: Not applicable

Test for overall effect: Z = 0.22 (P = 0.83)

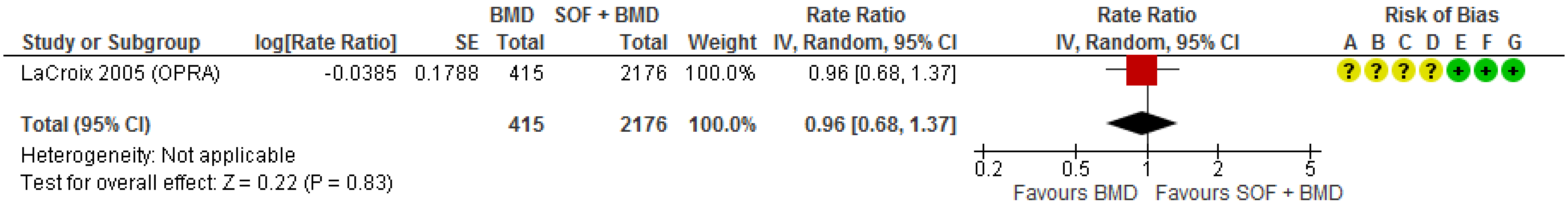

Risk of bias legend

(A) Random sequence generation (selection bias)

(B) Allocation concealment (selection bias)

(C) Blinding of participants and personnel (performance bias): Fracture outcomes

(D) Blinding of outcome assessment (detection bias): Fracture outcomes

(E) Incomplete outcome data (attrition bias): Fracture outcomes

(F) Selective reporting (reporting bias)

(G) Other bias

*The rate ratio is age-adjusted

Evidence Set 20: SOF + BMD vs. SCORE + BMD; hip fractures (All eligible/Offer-to-screen)
Included studies: LaCroix 2005 – OPRA (women 60-80 years)

20A. GRADE Evidence Profile Table

Population	Certainty assessment							№ of patients		Effect		Certainty*
	№ of studies	Study design	Risk of bias	Inconsistency	Indirectness	Imprecision	Other consideration	SOF + BMD	SCORE + BMD	Relative Rate Ratio (95% CI)	Absolute (95% CI)	
**Women 60-80 years (LaCroix 2005 - OPRA)**												
**SOF + BMD event rate (1.3% or 13 per 1000)**	1	RCT	-1[a]	-0.5[b]	NC[c]	-1[d]	NC	5342	1940	0.68 (0.40 to 1.15)	4.2 fewer per 1000 (7.8 fewer to 2.0 more)	VERY LOW ⨁⊖⊖⊖

BMD: bone mineral density; CI: confidence interval; NC: no serious concerns; RCT: randomized controlled trial; SCORE: Simple Calculated Osteoporosis Risk Estimation

* When our assessment of the certainty of evidence fell between levels, we assigned the level that best represented our actual certainty in the evidence, and based our conclusions on this the level of certainty.

Explanations:

[a] Risk of bias: Serious concern as the single included study is at risk of selection, performance, and detection biases.
[b] Inconsistency: Some concern about lack of evidence of consistency because there is only one study in the analysis.
[c] Indirectness: Recruitment method not typical (via letters), but effect on the outcome may be limited.
[c] Imprecision: The number of events does not meet the optimal information size (<300).

Evidence Set 20: SOF + BMD vs. SCORE + BMD; hip fractures (All eligible/Offer-to-screen)
Included studies: LaCroix 2005 – OPRA (women 60-80 years)

20B. GRADE Summary of Findings Table

Outcome No. participants (studies)	Relative effects, Rate Ratio (95% CI)	Anticipated absolute effects* (95% CI)			Certainty of evidence (GRADE)**	What happens?
		SOF + BMD	SCORE + BMD	Difference		
**Women 60-80 years (LaCroix 2005, OPRA)**						
Hip fractures<br><br>Follow-up: mean 2.3 years<br><br>7,282 (1 RCT)	0.68 (0.40 to 1.15)	SOF + BMD event rate<br>13 per 1000	8.8 per 1000 (5.2 per 1000 to 15.0 per 1000)	4.2 fewer per 1000 (7.8 fewer to 2.0 more)	VERY LOW<br>⊕⊖⊖⊖<br>due to risk of bias inconsistency and imprecision[a-d]	The evidence is very uncertain.

BMD: bone mineral density; CI: confidence interval; RCT: randomized controlled trial; SCORE: Simple Calculated Osteoporosis Risk Estimation; SOF: Study of Osteoporotic Fractures

* The absolute effect (and its 95% CI) with SOF + BMD (i.e. baseline rate) is based on the estimated risk in the SOF + BMD screening group; the effect with SCORE + BMD screening is based on applying the relative effect of the intervention (and its 95% CI) to the effect in the SCORE + BMD group.

** When our assessment of the certainty of evidence fell between levels, we assigned the level that best represented our actual certainty in the evidence, and based our conclusions on this the level of certainty.

Explanations:

[a] Risk of bias: Serious concern as the single included study is at risk of selection, performance, and detection biases.
[b] Inconsistency: Some concern about lack of evidence of consistency because there is only one study in the analysis.
[c] Indirectness: Recruitment method not typical (via letters), but effect on the outcome may be limited.
[d] Imprecision: The number of events does not meet the optimal information size (<300).

Evidence Set 20: SOF + BMD vs. SCORE + BMD; hip fractures (All eligible/Offer-to-screen)

Included studies: LaCroix 2005 – OPRA (women 60-80 years)

20C. Forest Plot

Study or Subgroup	log[Rate Ratio]	SE	SCORE + BMD Total	SOF + BMD Total	Weight	Rate Ratio IV, Random, 95% CI	Risk of Bias A	B	C	D	E	F	G
LaCroix 2005 (OPRA)	-0.3869	0.2666	1940	5342	100.0%	0.68 [0.40, 1.15]	?	?	?	?	+	+	+
**Total (95% CI)**			**1940**	**5342**	**100.0%**	**0.68 [0.40, 1.15]**							

Heterogeneity: Not applicable

Test for overall effect: Z = 1.45 (P = 0.15)

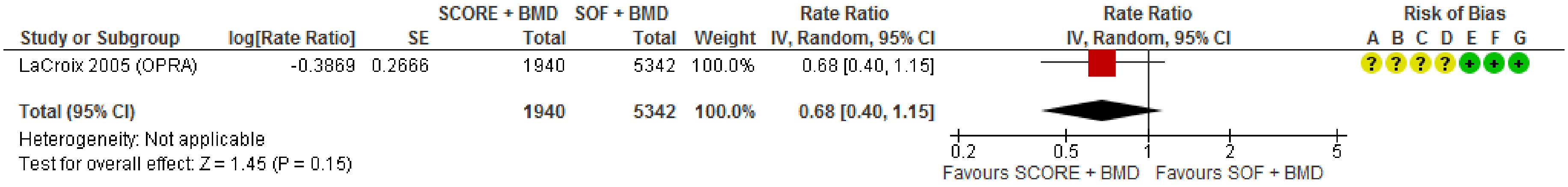

Risk of bias legend

(A) Random sequence generation (selection bias)

(B) Allocation concealment (selection bias)

(C) Blinding of participants and personnel (performance bias): Fracture outcomes

(D) Blinding of outcome assessment (detection bias): Fracture outcomes

(E) Incomplete outcome data (attrition bias): Fracture outcomes

(F) Selective reporting (reporting bias)

(G) Other bias

*The rate ratio is age-adjusted

Evidence Set 21: SOF + BMD vs. SCORE + BMD; hip fractures (Acceptors of screening)

Included studies: LaCroix 2005 – OPRA (women 60-80 years)

21A. GRADE Evidence Profile Table

Population	Certainty assessment							№ of patients		Effect		Certainty*
	№ of studies	Study design	Risk of bias	Inconsistency	Indirectness	Imprecision	Other consideration	SOF + BMD	SCORE + BMD	Relative Rate Ratio (95% CI)	Absolute (95% CI)	
**Women 60-80 years (LaCroix 2005 - OPRA)**												
**SOF + BMD event rate (0.9% or 9 per 1000)**	1	RCT	-1[a]	-0.5[b]	NC[c]	-1[d]	NC	2176	576	0.91 (0.33 to 2.52)	0.8 fewer per 1000 (6.0 fewer to 13.7 more)	VERY LOW ⨁⊖⊖⊖

BMD: bone mineral density; CI: confidence interval; NC: no serious concerns; RCT: randomized controlled trial; SCORE: Simple Calculated Osteoporosis Risk Estimation; SOF: Study of Osteoporotic Fractures

* When our assessment of the certainty of evidence fell between levels, we assigned the level that best represented our actual certainty in the evidence, and based our conclusions on this the level of certainty.

Explanations:

[a] Risk of bias: Serious concern about selection bias because the per protocol population may differ from the randomized sample. Some concern about performance and detection biases.

[b] Inconsistency: Some concern about lack of evidence of consistency because there is only one study in the analysis.

[c] Indirectness: Recruitment method not typical (via letters), but effect on the outcome may be limited.

[d] Imprecision: The number of events does not meet the optimal information size (<300).

Evidence Set 21: SOF + BMD vs. SCORE + BMD; hip fractures (Acceptors of screening)
Included studies: LaCroix 2005 – OPRA (women 60-80 years)

21B. GRADE Summary of Findings Table

Outcome<br>No. participants (studies)	Relative effects, Rate Ratio (95% CI)	Anticipated absolute effects* (95% CI)			Certainty of evidence (GRADE)**	What happens?
		SOF + BMD	SCORE + BMD	Difference		
**Women 60-80 years (LaCroix 2005, OPRA)**						
Hip fractures<br><br>Follow-up: mean 2.3 years<br><br>2,752 (1 RCT)	0.91 (0.33 to 2.52)	SOF + BMD event rate<br>9 per 1000	8.2 per 1000 (3.0 to 22.7)	0.8 fewer per 1000 (6.0 fewer to 13.7 more)	VERY LOW<br>⊕⊖⊖⊖<br>due to risk of bias, inconsistency, and imprecision[a-d]	The evidence is very uncertain.

BMD: bone mineral density; CI: confidence interval; RCT: randomized controlled trial; SCORE: Simple Calculated Osteoporosis Risk Estimation; SOF: Study of Osteoporotic Fractures

* The absolute effect (and its 95% CI) with SOF + BMD (i.e. baseline rate) is based on the estimated risk in the SOF + BMD screening group; the effect with SCORE + BMD screening is based on applying the relative effect of the intervention (and its 95% CI) to the effect in the SCORE + BMD group.

** When our assessment of the certainty of evidence fell between levels, we assigned the level that best represented our actual certainty in the evidence, and based our conclusions on this the level of certainty.

Explanations:

[a] Risk of bias: Serious concern about selection bias because the per protocol population may differ from the randomized sample. Some concern about performance and detection biases.

[b] Inconsistency: Some concern about lack of evidence of consistency because there is only one study in the analysis.

[c] Indirectness: Recruitment method not typical (via letters), but effect on the outcome may be limited.

[d] Imprecision: The number of events does not meet the optimal information size (<300).

Evidence Set 21: SOF + BMD vs. SCORE + BMD; hip fractures (Acceptors of screening)

Included studies: LaCroix 2005 – OPRA (women 60-80 years)

21C. Forest Plot

Study or Subgroup	log[Rate Ratio]	SE	SCORE + BMD Total	SOF + BMD Total	Weight	Rate Ratio IV, Random, 95% CI	Risk of Bias A	B	C	D	E	F	G
LaCroix 2005 (OPRA)	-0.0947	0.5205	576	2176	100.0%	0.91 [0.33, 2.52]	?	?	?	?	+	+	+
**Total (95% CI)**			**576**	**2176**	**100.0%**	**0.91 [0.33, 2.52]**							

Heterogeneity: Not applicable

Test for overall effect: Z = 0.18 (P = 0.86)

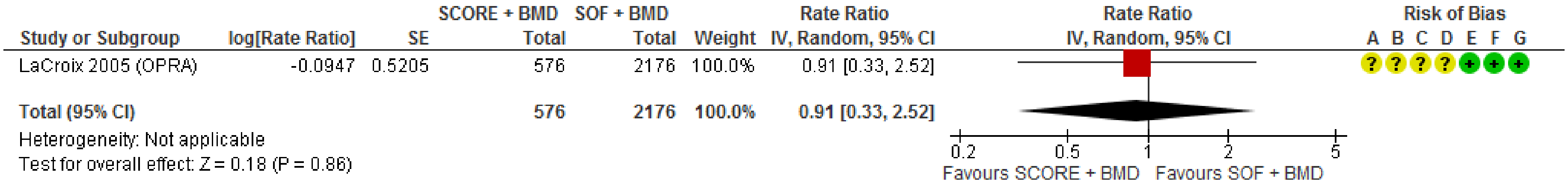

Risk of bias legend

(A) Random sequence generation (selection bias)

(B) Allocation concealment (selection bias)

(C) Blinding of participants and personnel (performance bias): Fracture outcomes

(D) Blinding of outcome assessment (detection bias): Fracture outcomes

(E) Incomplete outcome data (attrition bias): Fracture outcomes

(F) Selective reporting (reporting bias)

(G) Other bias

*The rate ratio is age-adjusted

Evidence Set 22: SOF + BMD vs. SCORE + BMD; all clinical fractures (All eligible/Offer-to-screen)
Included studies: LaCroix 2005 – OPRA (women 60-80 years)

22A. GRADE Evidence Profile Table

Population	Certainty assessment							№ of patients		Effect		Certainty*
	№ of studies	Study design	Risk of bias	Inconsistency	Indirectness	Imprecision	Other consideration	SOF + BMD	SCORE + BMD	Relative Rate Ratio (95% CI)	Absolute (95% CI)	
**Women 60-80 years (LaCroix 2005 - OPRA)**												
**SOF + BMD event rate (9.2% or 92 per 1000)**	1	RCT	-0.5[a]	-0.5[b]	-1[c]	-1[d]	NC	5342	1940	1.08 (0.92 to 1.28)	7.4 more per 1000 (7.4 fewer to 25.8 more)	VERY LOW ⊕⊖⊖⊖

BMD: bone mineral density; CI: confidence interval; NC: no serious concerns; RCT: randomized controlled trial; SCORE: Simple Calculated Osteoporosis Risk Estimation; SOF: Study of Osteoporotic Fractures

* When our assessment of the certainty of evidence fell between levels, we assigned the level that best represented our actual certainty in the evidence, and based our conclusions on this the level of certainty.

Explanations:

[a] Risk of bias: Some concern about potential for selection, performance, and detection biases.

[b] Inconsistency: Some concern about lack of evidence of consistency because there is only one study in the analysis.

[c] Indirectness: Serious concern because the 'non-pathological fractures' outcome is likely to include some non-clinical vertebral fractures due to the ascertainment method, and potentially other non-osteoporotic fractures. Recruitment method not typical (via letters), effect on the outcome may be limited.

[d] Imprecision: The confidence interval includes potential for both important benefit and harm.

Evidence Set 22: SOF + BMD vs. SCORE + BMD; all clinical fractures (All eligible/Offer-to-screen)
Included studies: LaCroix 2005 – OPRA (women 60-80 years)

22B. GRADE Summary of Findings Table

Outcome No. participants (studies)	Relative effects, Rate Ratio (95% CI)	Anticipated absolute effects* (95% CI)			Certainty of evidence (GRADE)**	What happens?
		SOF + BMD	SCORE + BMD	Difference		
**Women 60-80 years (LaCroix 2005, OPRA)**						
Clinical fragility fractures<br><br>Follow-up: mean 2.3 years<br><br>7,282 (1 RCT)	1.08 (0.92 to 1.28)	SOF + BMD event rate			VERY LOW ⊕⊖⊖⊖ due to risk of bias inconsistency, indirectness and imprecision[a-d]	The evidence is very uncertain.
		92 per 1000	99.4 per 1000 (84.6 to 117.8)	7.4 more per 1000 (7.4 fewer to 25.8 more)		

BMD: bone mineral density; CI: confidence interval; RCT: randomized controlled trial; SCORE: Simple Calculated Osteoporosis Risk Estimation; SOF: Study of Osteoporotic Fractures

* The absolute effect (and its 95% CI) with SOF + BMD (i.e. baseline rate) is based on the estimated risk in the SOF + BMD screening group; the effect with SCORE + BMD screening is based on applying the relative effect of the intervention (and its 95% CI) to the effect in the SCORE + BMD group.

** When our assessment of the certainty of evidence fell between levels, we assigned the level that best represented our actual certainty in the evidence, and based our conclusions on this the level of certainty.

Explanations:

[a] Risk of bias: Some concern about potential for selection, performance, and detection biases.

[b] Inconsistency: Some concern about lack of evidence of consistency because there is only one study in the analysis.

[c] Indirectness: Serious concern because the 'non-pathological fractures' outcome is likely to include some non-clinical vertebral fractures due to the ascertainment method, and potentially other non-osteoporotic fractures. Recruitment method not typical (via letters), effect on the outcome may be limited.

[d] Imprecision: The confidence interval includes potential for both important benefit and harm.

Evidence Set 22: SOF + BMD vs. SCORE + BMD; all clinical fractures (All eligible/Offer-to-screen)

Included studies: LaCroix 2005 – OPRA (women 60-80 years)

22C. Forest Plot

Study or Subgroup	log[Rate Ratio]	SE	SCORE + BMD Total	SOF + BMD Total	Weight	Rate Ratio IV, Random, 95% CI	Risk of Bias A	B	C	D	E	F	G
LaCroix 2005 (OPRA)	0.0803	0.085	1940	5342	100.0%	1.08 [0.92, 1.28]	?	?	?	?	+	+	+
**Total (95% CI)**			**1940**	**5342**	**100.0%**	**1.08 [0.92, 1.28]**							

Heterogeneity: Not applicable

Test for overall effect: Z = 0.94 (P = 0.34)

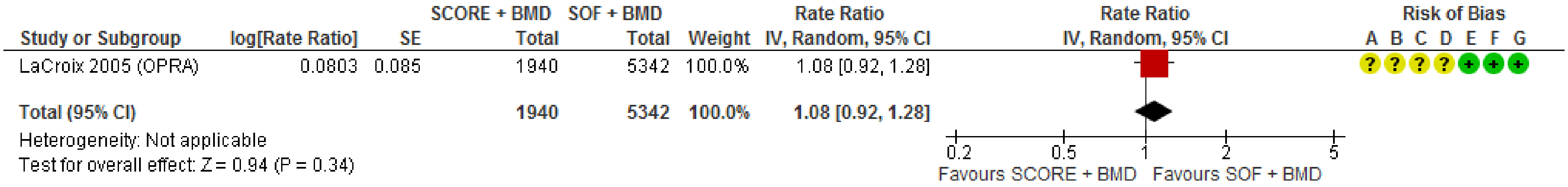

Risk of bias legend

(A) Random sequence generation (selection bias)

(B) Allocation concealment (selection bias)

(C) Blinding of participants and personnel (performance bias): Fracture outcomes

(D) Blinding of outcome assessment (detection bias): Fracture outcomes

(E) Incomplete outcome data (attrition bias): Fracture outcomes

(F) Selective reporting (reporting bias)

(G) Other bias

*The rate ratio is age-adjusted

Evidence Set 23: SOF + BMD vs. SCORE + BMD; clinical fragility fractures (Acceptors of screening)
Included studies: LaCroix 2005 – OPRA (women 60-80 years)

23A. GRADE Evidence Profile Table

Population	Certainty assessment							№ of patients		Effect		Certainty*
	№ of studies	Study design	Risk of bias	Inconsistency	Indirectness	Imprecision	Other consideration	SOF + BMD	SCORE + BMD	Relative Rate Ratio (95% CI)	Absolute (95% CI)	
**Women 60-80 years (LaCroix 2005 - OPRA)**												
**SOF + BMD event rate (9.3% or 93 per 1000)**	1	RCT	-1[a]	-0.5[b]	-1[c]	-0.5[d]	NC	2176	576	1.25 (0.95 to 1.65)	23.3 more per 1000 (4.6 fewer to 60.5 more)	VERY LOW ⨁⊖⊖⊖

BMD: bone mineral density; CI: confidence interval; NC: no serious concerns; RCT: randomized controlled trial; SCORE: Simple Calculated Osteoporosis Risk Estimation; SOF: Study of Osteoporotic Fractures

* When our assessment of the certainty of evidence fell between levels, we assigned the level that best represented our actual certainty in the evidence, and based our conclusions on this the level of certainty.

Explanations:

[a] Risk of bias: Serious concern about selection bias because the per protocol population may differ from the randomized sample. Some concern about performance and detection biases.

[b] Inconsistency: Some concern about lack of evidence of consistency because there is only one study in the analysis.

[c] Indirectness: Serious concern because the 'non-pathological fractures' outcome is likely to include some non-clinical vertebral fractures due to the ascertainment method, and potentially other non-osteoporotic fractures. Recruitment method not typical (via letters), effect on the outcome may be limited.

[d] Imprecision: The entire confidence interval does not cross the threshold of harm.

Evidence Set 23: SOF + BMD vs. SCORE + BMD; clinical fragility fractures (Acceptors of screening)

Included studies: LaCroix 2005 – OPRA (women 60-80 years)

23B. GRADE Summary of Findings Table

Outcome No. participants (studies)	Relative effects, Rate Ratio (95% CI)	Anticipated absolute effects* (95% CI)			Certainty of evidence (GRADE)**	What happens?
		SOF + BMD	SCORE + BMD	Difference		
**Women 60-80 years (LaCroix 2005, OPRA)**						
Clinical fragility fractures<br><br>Follow-up: mean 2.3 years<br><br>2,591 (1 RCT)	1.25 (0.95 to 1.65)	SOF + BMD event rate			VERY LOW ⊕⊖⊖⊖ due to risk of bias inconsistency, indirectness and imprecision[a-d]	The evidence is very uncertain.
		93 per 1000	116.3 per 1000 (88.4 to 153.5)	23.3 more per 1000 (4.6 fewer to 60.5 more)		

BMD: bone mineral density; CI: confidence interval; RCT: randomized controlled trial; SCORE: Simple Calculated Osteoporosis Risk Estimation; SOF: Study of Osteoporotic Fractures

* The absolute effect (and its 95% CI) with SOF + BMD (i.e. baseline rate) is based on the estimated risk in the SOF + BMD screening group; the effect with SCORE + BMD screening is based on applying the relative effect of the intervention (and its 95% CI) to the effect in the SCORE + BMD group.

** When our assessment of the certainty of evidence fell between levels, we assigned the level that best represented our actual certainty in the evidence, and based our conclusions on this the level of certainty.

Explanations:

[a] Risk of bias: Serious concern about selection bias because the per protocol population may differ from the randomized sample. Some concern about performance and detection biases.

[b] Inconsistency: Some concern about lack of evidence of consistency because there is only one study in the analysis.

[c] Indirectness: Serious concern because the 'non-pathological fractures' outcome is likely to include some non-clinical vertebral fractures due to the ascertainment method, and potentially other non-osteoporotic fractures. Recruitment method not typical (via letters), effect on the outcome may be limited.

[d] Imprecision: The entire confidence interval does not cross the threshold of harm.

Evidence Set 23: SOF + BMD vs. SCORE + BMD; clinical fragility fractures (Acceptors of screening)

Included studies: LaCroix 2005 – OPRA (women 60-80 years)

23C. Forest Plot

Study or Subgroup	log[Rate Ratio]	SE	SCORE + BMD Total	SOF + BMD Total	Weight	Rate Ratio IV, Random, 95% CI	Risk of Bias A	B	C	D	E	F	G
LaCroix 2005 (OPRA)	0.2265	0.141	576	2176	100.0%	1.25 [0.95, 1.65]	?	?	?	?	+	+	+
**Total (95% CI)**			**576**	**2176**	**100.0%**	**1.25 [0.95, 1.65]**							

Heterogeneity: Not applicable

Test for overall effect: Z = 1.61 (P = 0.11)

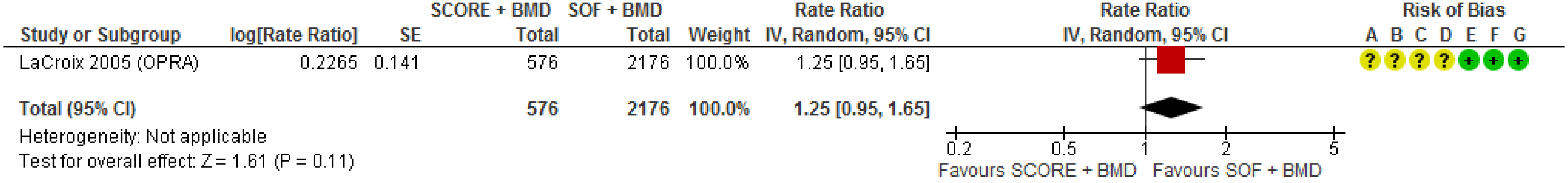

Risk of bias legend

(A) Random sequence generation (selection bias)

(B) Allocation concealment (selection bias)

(C) Blinding of participants and personnel (performance bias): Fracture outcomes

(D) Blinding of outcome assessment (detection bias): Fracture outcomes

(E) Incomplete outcome data (attrition bias): Fracture outcomes

(F) Selective reporting (reporting bias)

(G) Other bias

*The rate ratio is age-adjusted

REFERENCES

1. Prior JC, Langsetmo L, Lentle BC, Berger C, Goltzman D, Kovacs CS, et al. Ten-year incidence osteoporosis-related fractures in the population-based Canadian Multicentre Osteoporosis Study – Comparing site and age-specific risks in women and men. Bone 2015;71:237-243.

2. Statistics Canada. Table 13-10-0710-01 Deaths and mortality rates, by age group; Available at: https://doi.org/10.25318/1310071001-eng. Accessed Sept 30, 2019.

INCLUDED STUDIES

1. Merlijn T, Swart KMA, van Schoor NM, Heymans MW, van der Zwaard BC, van der Heijden AA, et al. The effect of a screening and treatment program for the prevention of fractures in older women: a randomized pragmatic trial. Journal of Bone & Mineral Research 2019;20:20.

2. Shepstone L, Lenaghan, E, Cooper C, Clarke S, Fong-Soe-Khioe R, Fordham R, et al. Screening in the community to reduce fractures in older women (SCOOP): a randomised controlled trial. Lancet 2018;391(10122):741-7.

Associated: McCloskey E, Johansson J, Harvey NC, Shepstone L, Lenaghan E, Fordham R, et al. Management of patients with high baseline hip fracture risk by FRAX reduces hip fractures - a post hoc analysis of the SCOOP Study. Journal of Bone & Mineral Research 2018;33(6):1020-6.

3. Rubin KH, Rothman MJ, Holmberg T, Hoiberg M, Moller S, Barkmann R. Effectiveness of a two-step population-based osteoporosis screening program using FRAX: the randomized Risk-stratified Osteoporosis Strategy Evaluation (ROSE) study. Osteoporosis International 2018;29(3):567-78.

4. Barr RJ, Stewart A, Torgerson DJ, Reid DM. Population screening for osteoporosis risk: a randomised control trial of medication use and fracture risk. Osteoporosis international 2010;21(4):561-8.

Associated: Torgerson DJ, Thomas RE, Campbell MK, Reid DM. Randomized trial of osteoporosis screening. Use of hormone replacement therapy and quality-of-life results. Archives of Internal Medicine 1997;157(18):2121-5.

5. Kern LM, Powe NR, Levine MA, Fitzpatrick AL, Harris TB, Robbins J, et al. Association between screening for osteoporosis and the incidence of hip fracture. Annals of Internal Medicine 2005;142(3):173-81.

6. LaCroix AZ, Buist DSM, Brenneman SK, Abbott TA III. Evaluation of three population-based strategies for fracture prevention: Results of the osteoporosis population-based risk assessment (OPRA) trial. Med Care. 2005;43(3):293-302.
