# Supplementary material for: Screening for the primary prevention of fragility fractures among adults aged 40 years and older in primary care: systematic reviews of the effects and acceptability of screening and treatment, and the accuracy of risk prediction tools
Source: Syst Rev. 2023 Mar 21;12:51. doi: 10.1186/s13643-023-02181-w (PMC10029308; doi:10.1186/s13643-023-02181-w)
Supplement: Supplementary file 4 — Additional file 4. Evidence sets for KQs 2-4. GRADE Summary of Findings for KQs 2-4. This file shows the analyses and GRADE Summary of Findings for key questions 2 (accuracy of screening tests), 3a (benefits of treatment), 3b (harms of treatment), and 4 (acceptability of screening/treatment). [file 13643_2023_2181_MOESM4_ESM.pdf]

Screening for the primary prevention of fragility fractures among adults aged 40 years and older in primary care: systematic reviews of the effects and acceptability of screening and treatment, and the accuracy of risk prediction tools

Authors: Michelle Gates, Jennifer Pillay (corresponding), Megan Nuspl, Aireen Wingert, Ben Vandermeer, Lisa Hartling

Affiliation: Alberta Research Centre for Health Evidence, Department of Pediatrics, University of Alberta. Edmonton Clinic Health Academy, 11405-87 Avenue NW, Edmonton, Alberta, Canada T6G 1C9. E-mail: MG: michellegates85@gmail.com; JP: jpillay@ualberta.ca; MN: megan.nuspl@ualberta.ca; AW: awingert@ualberta.ca; BV: ben.vandermeer@ualberta.ca; LH: hartling@ualberta.ca

Additional file 4. Summary of findings for KQs 2, 3a, 3b, and 4

Contents

Key Question	Page
**KQ2:** predictive accuracy of screening tests	2
**KQ3a:** benefits of pharmacologic treatments	22
**KQ3b:** harms of pharmacologic treatments	62
**KQ4:** acceptability of screening and/or treatment	76
**References**	78

KQ2: How accurate are screening tests at predicting fractures among adults ≥40 years?

EVIDENCE SUMMARY FOR KQ2 ON THE PREDICTIVE ACCURACY OF SCREENING TESTS

Background and approach to GRADE

Observed to expected fracture ratio (O:E) ratio

- The O:E ratio is a measure of model calibration, and indicates the extent of agreement between the expected number of events (i.e., number of fractures predicted by the tool) and the observed number of events (i.e., actual number of individuals with one or more fractures observed during follow-up) [1].

- The O:E ratio may range from 0 to infinity. An ideal tool would have a O:E ratio of 1.0, which means that there are exactly the same number of fractures observed as were predicted by the tool.

- We considered tools to be well calibrated when the O:E ratio is between 0.8 and 1.2 [1]. An O:E ratio <1 indicates that the tool overestimates the observed probability of fractures, while an O:E ratio >1 indicates that the tool underestimates the observed probability of fractures.

Conclusions and interpretation of the evidence

- Due to heterogeneity that was not well explained by our a-priori subgroup analyses, most conclusions are descriptive and not based on a pooled estimate. For the FRAX tool, we pooled data from Canadian studies at lower risk of bias and present these separately from the other studies that were all at high risk of bias.

- When there was no pooled estimate, we rated precision based on the recommendations for assessing the certainty of the evidence in the absence of a single estimate of effect from meta-analysis [2]

- There were serious risk of bias concerns across the majority of studies that could merit rating down twice. We instead usually rated down once, considering that our ratings for inconsistency and imprecision may have been at least in part related to concerns about risk of bias and/or indirectness (i.e., to avoid double-counting when we rated down).

- We considered a range of potential conclusions:

    - **The tool is well calibrated**: most comparisons showing an O:E ratio between 0.8 and 1.2
    - **The tool underestimates fracture risk**: most comparisons showing an O:E ratio >1.2, and the magnitude is adequately consistent and precise to draw clinically meaningful conclusions
    - **The tool overestimates fracture risk**: most comparisons showing an O:E ratio <0.8, and the magnitude is adequately consistent and precise to draw clinically meaningful conclusions
    - **The tool is poorly calibrated**: most comparisons showing an O:E ratio <0.8 or >1.2, but the direction of the calibration (over- or underestimation) is unclear

- Certainty of evidence appraisals were based on the conclusion that is shown in each summary of findings table.

KQ2: How accurate are screening tests at predicting fractures among adults ≥40 years?

1. Clinical FRAX

1.1 GRADE Summary of Findings

Outcome* Studies; sample size	Findings	Certainty†	What does the evidence say?	Discrimination‡ [3] (pooled AUC, 95% CI)
**Clinical FRAX (high risk of bias studies)**				
10-y hip fractures<br>13 cohort; 343,755<br>[4-16]	None of the FRAX tools in this analysis were calibrated for Canada. Most studies show poor calibration and are inconsistent. Most often, the tool over- (n=4 studies, 4 comparisons; O:E estimates from 0.26 to 0.72) or underestimated (n=5 studies, 7 comparisons; O:E 1.21 to 3.87) the observed fracture risk. Inconsistency was not well explained by subgroup analyses.	VERY LOW[a]	Very uncertain for the conclusion of poor performance.	All studies, regardless of risk of bias:<br>F: 0.76 (0.72-0.81)<br>M: 0.73 (0.68-0.77)
10-y clinical fragility fractures<br>12 cohort; 190,116<br>[4, 5, 7-12, 14-20]	Only one of the 12 studies used the FRAX tool calibrated for Canada. Most studies show poor calibration and are inconsistent. Most often, the tool underestimated (n=7 studies, 8 comparisons; O:E 1.33 to 3.34) the observed fracture risk. Inconsistency was not well explained by subgroup analyses.	VERY LOW[b]	Very uncertain for the conclusion of poor performance.	All studies, regardless of risk of bias:<br>F: 0.67 (0.65-0.68)<br>M: 0.62 (0.61-0.64)
5-y hip fractures<br>1 cohort; 1,054,815<br>[21]	A single study that did not use a FRAX tool calibrated to Canada showed underestimation of the observed 5-year risk of hip fracture (O:E 1.74, 95% CI 1.72-1.76).	VERY LOW[c]	Very uncertain for the conclusion of underestimation.	NR
5-y clinical fragility fractures<br>1 cohort; 9,393 [22]	A single study of a FRAX tool calibrated to Canada showed overestimation of the observed 5-year risk of clinical fragility fracture (O:E 0.75, 95% CI 0.68-0.89).	VERY LOW[d]	Very uncertain for the conclusion of overestimation.	NR
**Clinical FRAX (lower risk of bias studies)**				
10-y hip fractures<br>3 cohort; 67,611<br>[17-19]	All studies used the FRAX tool calibrated for Canada. The pooled O:E showed acceptable calibration with some underestimation of the observed fracture risk, and a wide confidence interval (pooled O:E 1.13, 95% CI 0.74-1.72, I^2=89.2%).	LOW[e]	May be well calibrated.	See above.
10-y clinical fragility fractures<br>3 cohort; 67,611<br>[17-19]	All studies used the FRAX tool calibrated for Canada. The pooled O:E showed acceptable calibration with some underestimation of the observed fracture risk (O:E 1.10, 95% CI 1.01-1.20, I^2=50.4%).	MODERATE[f]	Probably well calibrated.	See above.
5-y hip fractures<br>1 cohort; 68,730<br>(62,275 F, 6,445 M)<br>[19]	A single study, which used the FRAX tool calibrated for Canada, showed large overestimation of the observed 5-year risk of hip fracture in females (O:E 0.68, 95% CI 0.62-0.73) and imprecise overestimation in males (O:E 0.82, 95% CI 0.60- 1.03).	LOW[g]	May be poorly calibrated.	NR
5-y clinical fragility fractures<br>1 cohort; 68,730<br>(62,275 F, 6,445 M)<br>[19]	A single study, which used the FRAX tool calibrated for Canada, found acceptable calibration in females (O:E 0.93, 95% CI 0.89-0.96). The tool imprecisely underestimated the observed fracture risk in males (O:E 1.23, 95% CI 1.08-1.38).	LOW[h]	May be well calibrated (most applicable to females).	NR

BMD=bone mineral density; CI=confidence interval; F=female; M=male; NR=not reported; O:E ratio=ratio of observed to expected (predicted) events

*Rows for 5-year fractures have been omitted from the table when no studies were located that reported on this outcome.

† When our assessment of the certainty of evidence fell between levels, we assigned the level that best represented our actual certainty.

‡Extracted directly from the 2018 USPSTF systematic review by Viswanathan et al. [3]

Explanations:

[a] 10-year hip fractures (high risk of bias studies): ***Serious concern about risk of bias***: all studies were at high risk of bias primarily due to concerns about predictor ascertainment (missing predictor data, predictors not handled as intended), outcome ascertainment (self-reported or including high trauma fractures), and analysis (high losses to follow-up/deaths not accounted for, inadequate number of fracture outcomes, short follow-up period, not accounting for competing mortality risk). ***Some concern about inconsistency***: most point estimates agree with conclusions of poor performance, though 5 studies (6 comparisons) suggest acceptable calibration. Inconsistency is not fully explained by *a-priori* subgroups, and the direction of poor performance (under- or overestimating) is unclear. Rated down 0.5 because it is believed that this inconsistency is at

least partly related to risk of bias concerns. ***Some concern about indirectness:*** in 5 (Azagra 2016a, Bolland 2011, Czerwinski 2013, Goldshtein 2017, Pressman 2011) studies participants are likely to be higher risk than the general primary care population because they were referred for BMD testing, one study (Yin 2016) enrolled only veterans, and another (Ettinger 2013) enrolled participants who were likely to be healthier than the general population. Differences between studies do not appear to explain the findings. None of the studies use the FRAX tool calibrated for Canada. ***Serious concern for imprecision***: the confidence intervals of 7 studies cross the upper and/or lower threshold for being well calibrated, and most others are too wide to indicate a clinically meaningful over- or underestimation. ***No serious concerns for other considerations:*** no other concerns that would further impact certainty in the estimates. Publication bias not detected.

[b] <u>10-year clinical fragility fractures (high risk of bias studies):</u> ***Serious concern about risk of bias***: all studies were at high risk of bias primarily due to concerns about predictor ascertainment (missing predictor data, predictors not handled as intended), outcome ascertainment (self-reported or including high trauma fractures), and analysis (high losses to follow-up/deaths not accounted for, inadequate number of fracture outcomes, short follow-up period, not accounting for competing mortality risk). ***No serious concern about inconsistency***: All but two point estimates agree with conclusions of poor performance. The direction of poor performance is unclear. ***Some concern about indirectness***: in 4 (Azagra 2016a, Bolland 2011, Czerwinski 2013, Goldshtein 2017) studies, participants are likely to be higher risk than the general primary care population because they were referred for BMD testing, one study (Yin 2016) enrolled only veterans, and another (Ettinger 2013) enrolled participants who were likely to be healthier than the general population. Only one of the studies (Li 2015) used the FRAX tool calibrated for Canada. Differences between studies do not appear to explain the findings. ***Serious concern for imprecision***: The confidence intervals of 4 studies cross the upper and/or lower threshold for being well calibrated, and most others are too wide to indicate a clinically meaningful over- or underestimation. ***No serious concerns for other considerations:*** no other concerns that would further impact certainty in the estimates. Publication bias not detected.

[c] <u>5-year hip fractures (high risk of bias studies):</u> ***Serious concern about risk of bias:*** the one included study is at high risk of bias because it did not account for competing risk of mortality and use of the tool was not as intended (halved the 10-year risk estimate to obtain a 5-year risk). ***Serious concern about inconsistency:*** there is no evidence of consistency because there was only one study reporting this outcome, and demonstrated inconsistency in other analyses reinforces the need for several studies to support certainty in conclusions. ***Some concern for indirectness:*** The study did not use the FRAX tool calibrated for Canada. ***No serious concerns for imprecision, or other considerations:*** no other concerns that would further impact certainty in the estimates.

[d] <u>5-year clinical fragility fracture (high risk of bias studies):</u> ***Serious concern about risk of bias:*** the one included study is at high risk of bias because it did not have adequate data on several predictors nor account for competing risk of mortality, and use of the tool was not as intended (halved the 10-year risk estimate to obtain a 5-year risk). ***Serious concern about inconsistency:*** there is no evidence of consistency because there was only one study reporting this outcome, and demonstrated inconsistency in other analyses reinforces the need for several studies to support certainty in conclusions. ***No serious concern about indirectness:*** The study used the FRAX tool calibrated for Canada and the population is quite representative. ***Serious concerns for imprecision, or other considerations:*** the confidence interval for the calibration includes values that indicate the tool may perform well.

[e] <u>10-year hip fractures (lower risk of bias studies):</u> ***No serious concern about risk of bias:*** any concerns about risk of bias were quite minimal compared to other analyzed studies. The main potential concern was the use of proxy variables which might be expects to result in underestimation of fracture risk (i.e., the tool would be better calibrated than it appears from the analysis), thus we did not rate down. ***Serious concern for inconsistency:*** Three of the four point estimates fall in the range of acceptable performance, with one (Fraser 2011, men) showing substantial underestimation of the observed risk. The I^2 for the pooled estimate is 89.2%. ***Some concern for imprecision***: The confidence interval of the pooled estimate is wide, including the potential for substantial under- or overestimation of the observed risk. This is partly the result of inconsistency. ***No serious concerns about indirectness:*** participants in one of the studies may be higher risk than the general primary care population because they were all referred for BMD testing. Based on other analyses, the impact on the findings is unclear. All studies used the FRAX tool calibrated for Canada. ***No serious concerns for other considerations***: no other concerns that would further impact certainty in the estimates.

KQ2: How accurate are screening tests at predicting fractures among adults ≥40 years?

[f] <u>10-year clinical fragility fractures (lower risk of bias studies):</u> ***No serious concern about risk of bias:*** any concerns about risk of bias were quite minimal compared to other analyzed studies. The main potential concern was the use of proxy variables which might be expects to result in underestimation of fracture risk (i.e., the tool would be better calibrated than it appears from the analysis), thus we did not rate down. ***Some concern for inconsistency:*** All point estimates fall in the range of acceptable performance, with some inconsistency in the degree of underestimation (6 to 19%). I^2 for the pooled estimate is 50.4%. ***Some concern for imprecision***: The confidence interval includes a wide range of potential O:E estimates, ranging between 1 and 20% underestimation of the observed risk. This is partly the result of inconsistency. ***No serious concerns about indirectness:*** participants in one of the studies may be higher risk than the general primary care population because they were all referred for BMD testing. Based on other analyses, the impact on the findings is unclear. All studies used the FRAX tool calibrated for Canada. ***No serious concerns for other considerations***: no other concerns that would further impact certainty in the estimates.

[g] <u>5-year hip fractures (lower risk of bias studies):</u> ***Some concern about risk of bias:*** Use of the tool was not as intended (halved the 10-year risk estimate to obtain a 5-year risk). ***Serious concern for inconsistency:*** there is no evidence of consistency because there is only one study for this outcome, and demonstrated inconsistency in other analyses reinforces the need for several studies to support certainty in conclusions. ***No serious concerns about indirectness***: participants may be higher risk than the general primary care population because they all were referred for BMD testing. Based on other analyses the impact on the findings is unclear. The study used the FRAX tool calibrated for Canada. ***Some concern about imprecision:*** the 95% confidence interval for males includes the potential that the tool is well calibrated or overestimates the observed fracture risk. ***No serious concerns for other considerations:*** no other concerns that would further impact certainty in the estimates.

[h] <u>5-year clinical fragility fractures (lower risk of bias studies):</u> ***Some concern about risk of bias:*** Use of the tool was not as intended (halved the 10-year estimate to obtain a 5-year risk). ***Serious concern for inconsistency:*** there is no evidence of consistency because there is only one study for this outcome, and demonstrated inconsistency in other analyses reinforces the need for several studies to support certainty in conclusions. ***No serious concerns about indirectness***: participants are likely to be higher risk than the general primary care population because they all were referred for BMD testing. Based on other analyses the impact on the findings is unclear. The study used the FRAX tool calibrated for Canada. ***Some concern about imprecision:*** The 95% confidence interval for males includes the potential that the tool is well calibrated or underestimates the observed fracture risk. ***No serious concerns for other considerations:*** no other concerns that would further impact certainty in the estimates.

KQ2: How accurate are screening tests at predicting fractures among adults ≥40 years?

1.2 Contributing data

Calibration for the 10-year prediction of hip fractures

High risk of bias studies (none Canadian)

Study or Subgroup	log[Prediction Performance]	SE	Prediction Performance IV, Random, 95% CI
Pluskiewicz 2015	-1.34807319	0.49869831	0.26 [0.10, 0.69]
Tamaki 2011	-0.81093022	0.4987715	0.44 [0.17, 1.18]
Pressman 2011 (70-79 years)	-0.6856752	0.0250628	0.50 [0.48, 0.53]
Bolland 2011	-0.32365756	0.12977143	0.72 [0.56, 0.93]
Marques 2017 (men)	-0.21309322	0.125	0.81 [0.63, 1.03]
Sornay-Rendu 2010	-0.20212419	0.24014605	0.82 [0.51, 1.31]
Ettinger 2013 (men)	-0.1541507	0.0740851	0.86 [0.74, 0.99]
Marques 2017 (women)	-0.09855167	0.03571429	0.91 [0.84, 0.97]
Crandall 2019b	0.08468309	0.04605224	1.09 [0.99, 1.19]
Premaor 2013	0.12641392	0.04379517	1.13 [1.04, 1.24]
Pressman 2011 (50-59 years)	0.1872115	0.0787801	1.21 [1.03, 1.41]
Goldshtein 2017	0.27625338	0.01539249	1.32 [1.28, 1.36]
Pressman 2011 (60-69 years)	0.5887872	0.034905	1.80 [1.68, 1.93]
Pressman 2011 (80-89 years)	0.7754424	0.0341893	2.17 [2.03, 2.32]
Azagra 2016a	0.82417544	0.192	2.28 [1.56, 3.32]
Czerwinski 2013	0.90784169	0.17125207	2.48 [1.77, 3.47]
Yin 2016 (men)	1.35325451	0.06427653	3.87 [3.41, 4.39]

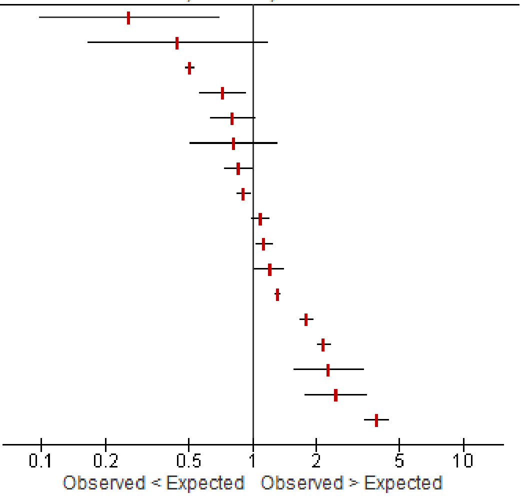

Lower risk of bias studies (all Canadian)

Study	O:E ratio (95% CI)	% Weight
Fraser 2011 (F)	0.93 (0.79, 1.10)	25.84
Leslie 2017 (M)	0.97 (0.78, 1.21)	23.83
Crandall 2019b (F)	1.13 (1.08, 1.18)	29.29
Fraser 2011 (M)	1.71 (1.29, 2.28)	21.04
Overall (I-squared = 89.2%)	1.13 (0.74, 1.72)	100.00
with estimated prediction interval	(0.36, 3.52)	

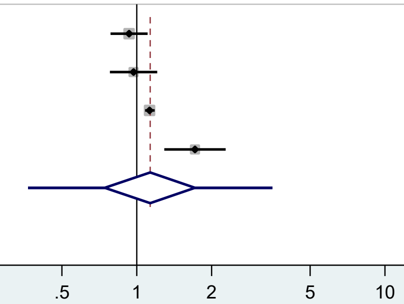

NOTE: Weights are from random-effects model

KQ2: How accurate are screening tests at predicting fractures among adults ≥40 years?

Calibration for the 10-year prediction of clinical fragility fractures (FRAX-defined major osteoporotic fractures)

High risk of bias studies (All but 1 non-Canadian)

Study or Subgroup	log[O-E Ratio]	SE	O-E Ratio IV, Random, 95% CI
Li 2015	-1.1174933	0.07975063	0.33 [0.28, 0.38]
Tamaki 2019	-0.4904188	0.11948272	0.61 [0.48, 0.77]
Ettinger 2013 (men)	-0.25453	0.0478581	0.78 [0.71, 0.85]
Pluskiewicz 2015	-0.1584891	0.14297309	0.85 [0.64, 1.13]
Premaor 2013	-0.1391377	0.02813576	0.87 [0.82, 0.92]
Marques 2017 (men)	0.28163976	0.03030303	1.33 [1.25, 1.41]
Bolland 2011	0.3217342	0.06052749	1.38 [1.23, 1.55]
Yin 2016 (men)	0.3293037	0.03308108	1.39 [1.30, 1.48]
Sornay-Rendu 2010	0.3597808	0.10507959	1.43 [1.17, 1.76]
Marques 2017 (women)	0.39688136	0.00689655	1.49 [1.47, 1.51]
Goldshtein 2017	0.6711683	0.00673348	1.96 [1.93, 1.98]
Azagra 2016a	0.8285518	0.09205476	2.29 [1.91, 2.74]
Czerwinski 2013	1.2058578	0.06738501	3.34 [2.93, 3.81]

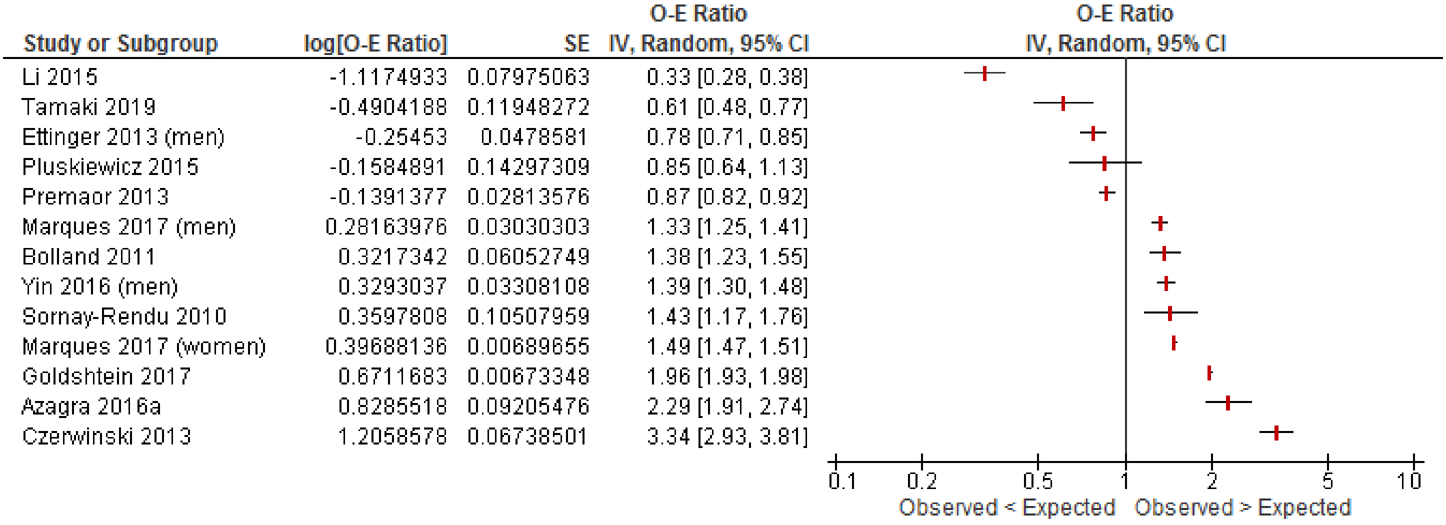

Lower risk of bias studies (All Canadian)

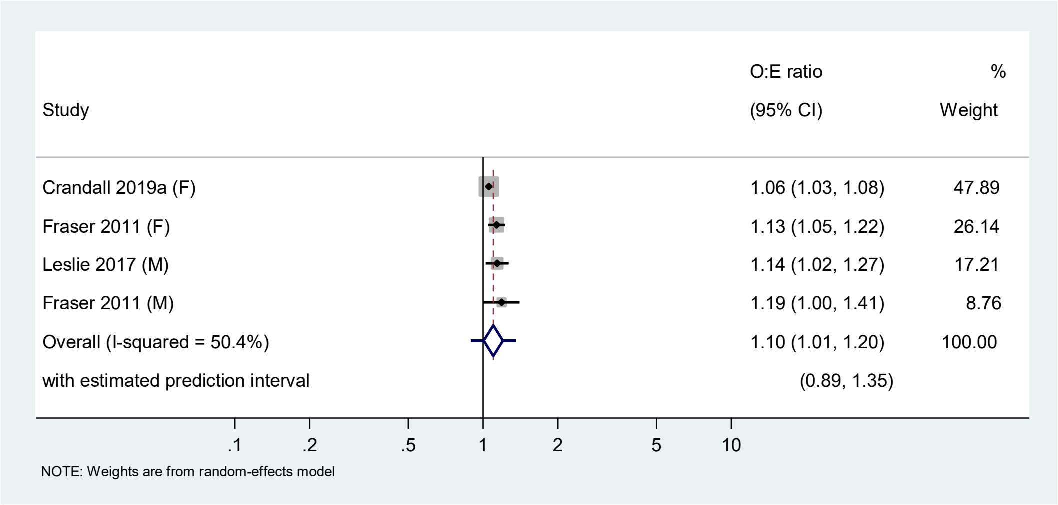

Summary of subgroup analyses

Outcome	N studies; compar-isons	n	O:E ratio (95% CI)[a] or regression coefficient (95% CI)	I^2	Prediction interval
**1.0 10-year clinical fragility fractures**	**15; 17**	**251,272**	**1.18 (0.90, 1.54)[b]**	**100%**	**0.38, 3.66**
1.1 Between study subgroup: mean age (test for subgroup differences, p=0.063)					
<65 years	7; 7	226,572	1.36 (0.92, 2.02)	100%	0.44, 4.26
≥65 years	8; 9	24,700	1.03 (0.65, 1.65)	99%	0.23, 4.67
1.2 Between study subgroup: sex (test for subgroup differences, p=0.397)					
Males	5; 5	32,944	1.14 (0.85, 1.53)	96%	0.51, 2.54
Females	12; 12	218,328	1.19 (0.80, 1.76)	100%	0.29, 4.91
1.3 Between study sensitivity: risk of bias (test for subgroup differences, p=0.003)					
Unclear risk of bias	3; 4	61,156	1.10 (1.01, 1.20)	50%	0.89, 1.35
High risk of bias	12; 12	190,116	1.18 (0.79, 1.76)	100%	0.28, 5.01

KQ2: How accurate are screening tests at predicting fractures among adults ≥40 years?

Outcome	N studies; comparisons	n	O:E ratio (95% CI)[a] or regression coefficient (95% CI)	I^2	Prediction interval
1.4 Subgroup analysis using within-study data					
Females ≥65 years	6; 15	68,368	1.56 (1.18, 2.06)[b]	99%	0.53, 4.50
1.5 Meta-regressions					
Mean age	15; 17	251,272	0.96 (0.92, 1.01), p=0.115		
Mean baseline risk	15; 17	251,272	-0.05 (-0.10, -0.01), p=0.029		
**2.0 10-year hip fractures**	**17; 21**	**404,911**	**1.15 (0.88, 1.49)[b]**	**99%**	**0.37, 3.60**
2.1 Between study subgroup: mean age (test for subgroup differences, p=0.221)					
<65 years	8; 8	271,305	1.25 (0.72, 2.17)	99%	0.25, 6.26
≥65 years	9; 12	133,606	1.09 (0.75, 1.58)	99%	0.32, 3.75
2.2 Between study subgroup: sex (test for subgroup differences, p=0.404)					
Males	5; 5	32,944	1.35 (0.59, 3.10)	98%	0.14, 13.50
Females	13; 16	371,967	1.09 (0.81, 3.27)	99%	0.37. 3.27
2.3 Between study sensitivity: risk of bias (test for subgroup differences, p=0.796)					
Unclear risk of bias	3; 4	343,755	1.13 (0.74, 1.72)	89%	0.36, 3.52
High risk of bias	13; 16	61,156	1.14 (0.80, 1.64)	99%	0.28, 4.59
2.4 Subgroup analysis using within-study data					
Females ≥65 years	7; 18	123,719	1.57 (0.94, 2.62)[b]	100%	0.18, 13.44
2.5 Meta-regressions					
Mean age	17; 21	404,911	0.99 (0.95, 1.03), p=0.57		
Mean baseline risk	17; 21	404,911	-0.01 (-0.11, 0.09), p=0.86		
**3.0 5-year clinical fragility fractures**	**1; 2**	**62,275<br>6,455**	**F: 0.93 (0.89, 0.96)<br>M: 1.23 (1.08, 1.38)**	**NA**	**NA**
**4.0 5-year hip fractures**	**2; 3**	**In one study (n=68,730), the O:E ratio (95% CI) was 0.68 (0.62, 0.73) in females and 0.82 (0.60, 1.03) in males. In another study (n=1,054,815) the O:E ratio was 1.76.**			

F=female; O:E ratio=ratio of observed to expected (predicted) events; M=male; NA=not applicable
[a]Pooled using restricted maximum likelihood (REML) estimation and the Hartung-Knapp-Sidik-Jonkman (HKSJ) correction.
[b]The pooled estimate was suppressed in the summary of findings due to high unexplained heterogeneity.

Summary of data from calibration plots

Within-study data from calibration plots were inconsistent (data available on request). Eight studies provided calibration plot data for 10-year risk of clinical fragility fractures; none fully confirmed the conclusions drawn from between-study meta-regression. Two studies (Azagra 2016, Goldshtein 2017) showed that FRAX without BMD underestimated the observed risk of clinical fractures, one study (Li 2015) showed overestimation, and two (Crandall 2019b, Premaor 2013) showed acceptable calibration at all levels of baseline risk. Two studies (Bolland 2011, Ettinger 2013) showed that FRAX without BMD underestimated the observed risk of fractures in those at lower baseline risk, but may be well calibrated at higher levels of baseline risk (≥7.7% in one study and 15% in another). There was no apparent trend in the remaining study (Tamaki 2019).

Seven studies provided calibration plot data for 10-year risk of hip fractures. Two studies (Crandall 2019b, Premaor 2013) showed that clinical FRAX underestimated the observed risk of hip fracture at lower levels of baseline risk, but appeared to be well calibrated at higher levels of baseline risk (≥1.6% in one study and 3% in another). Two studies (Bolland 2011, Tamaki 2019) showed overestimation of the observed risk of hip fracture at higher levels of baseline risk, but acceptable calibration at lower levels of baseline risk (≤5.6% in one study and 0.2% in another). One study (Ettinger 2013) showed acceptable calibration at all levels of baseline risk and there was no apparent trend in the two remaining studies (Azagra 2016, Goldshtein 2017).

One study (Desbiens 2020) provided calibration plot data for 5-year clinical fragility fractures. Clinical FRAX overestimated, to a similar degree, the observed risk of fracture at all levels of baseline risk. One study (Dagan 2017) reported calibration plot

data for the 5-year prediction of hip fractures. In this study, clinical FRAX underestimated the observed risk of fracture at all levels of baseline risk.

2. FRAX + BMD

2.1 GRADE Summary of Findings

Outcome* Studies; sample size	Findings	Certainty†.	What does the evidence say?	Discrimination‡ [3] (pooled AUC, 95% CI)
**FRAX + BMD (high risk of bias studies)**				
10-y hip fractures<br>13 cohort; 138,606<br>[4, 5, 7-15, 23, 24]	None of the FRAX tools in this analysis were calibrated for Canada. Most studies show poor calibration and are inconsistent. Most often, the tool either over- (n = 4 studies, 6 comparisons; O:E range from 0.24 to 0.68) or underestimated (n = 8 studies, 10 comparisons; O:E 1.30 to 3.33) the observed fracture risk. Inconsistency was not well explained by subgroup analyses.	VERY LOW[a]	Very uncertain for the conclusion of poor performance.	All studies, regardless of risk of bias:<br>F: 0.79 (0.76-0.81)<br>M: 0.76 (0.72-0.80)
10-y clinical fragility fractures<br>16 cohort; 49,235<br>[4, 5, 7-12, 14, 15, 23-28]	None of the FRAX tools in this analysis were calibrated for Canada. Most studies show poor calibration and are inconsistent. Most often (10 studies, 12 comparisons; O:E 1.11 to 3.90), the tool underestimated the observed fracture risk. Inconsistency was not well explained by subgroup analyses.	VERY LOW[b]	Very uncertain for the conclusion of poor performance.	All studies, regardless of risk of bias:<br>F: 0.70 (0.68-0.71)<br>M: 0.67 (0.66-0.68)
**FRAX + BMD (lower risk of bias studies)**				
10-y hip fractures<br>3 cohort; 61,156<br>[17-19]	All studies used the FRAX tool calibrated for Canada. The pooled O:E showed underestimation of the observed risk with a high level of inconsistency (O:E 1.31, 95% CI 0.91-2.13, I^2 = 92.7%); two comparisons showed acceptable calibration while two others showed substantial underestimation of the observed fracture risk.	LOW[c]	May perform poorly.	See above.
10-y clinical fragility fractures<br>3 cohort; 61,156<br>[17-19]	All studies used the FRAX tool calibrated for Canada. The pooled O:E showed acceptable calibration with some underestimation of the observed risk (O:E 1.16, 95% CI 1.12-1.20, I^2 = 0%).	MODERATE[d]	Probably well calibrated.	See above.
5-y hip fractures<br>1 cohort; 68,730<br>(62,275 F, 6,445 M)<br>[19]	A single study, which used the FRAX tool calibrated for Canada, showed acceptable calibration with some overestimation in females (O:E 0.88, 95% CI 0.81-0.95) and males (O:E 0.88, 95% CI 0.65-1.10).	LOW[e]	May be well calibrated (most applicable to females.	NR
5-y clinical fragility fractures<br>1 cohort; 68,730<br>(62,275 F, 6,445 M)<br>[19]	A single, which used the FRAX tool calibrated for Canada, study provided inconsistent findings, showing acceptable calibration in females (O:E 1.00, 95% CI 0.97-1.04). The tool imprecisely underestimated the observed fracture risk in males (O:E 1.22, 95% CI 1.07, 1.37).	LOW[f]	May be well calibrated (most applicable to females).	NR

BMD=bone mineral density; CI=confidence interval; F=female; M=male; NR=not reported; O:E ratio=ratio of observed to expected (predicted) events
*Rows for 5-year fractures have been omitted from the table when no studies were located that reported on this outcome.
† When our assessment of the certainty of evidence fell between levels, we assigned the level that best represented our actual certainty.
‡Extracted directly from the 2018 USPSTF systematic review by Viswanathan et al. [3]

Explanations:

[a] 10-year hip fractures (high risk of bias studies): ***Serious concern about risk of bias:*** all studies were at high risk of bias primarily due to concerns about predictor ascertainment (missing predictor data, predictors not handled as intended), outcome ascertainment (self-reported or including high trauma fractures), and analysis (high losses to follow-up/deaths not accounted for, inadequate number of fracture outcomes, short follow-up period, not accounting for competing mortality risk). ***No serious concern about inconsistency:*** All but two point estimates agree with conclusions of poor performance. The direction of poor performance (under- or overestimating) is unclear. ***Some concern about indirectness:*** in 5 (Azagra 2016a, Bolland 2011, Czerwinski 2013, Goldshtein 2017, Pressman 2011) studies participants are likely to be higher risk than the general primary care population because they were referred for BMD testing, and one study (Ettinger 2013) enrolled

participants who were likely to be healthier than the general population. No studies used the FRAX tool calibrated to Canada. Differences between studies do not appear to explain the findings. ***Serious concerns for imprecision:*** The confidence intervals of 10 studies cross the upper and/or lower threshold for being well calibrated, and most others are too wide to indicate a clinically meaningful over- or underestimation. ***No serious concerns for other considerations:*** no other concerns that would further impact certainty in the estimates. Publication bias not detected.

[b] <u>10-year clinical fragility fractures (high risk of bias studies):</u> ***Serious concern about risk of bias:*** all studies were at high risk of bias primarily due to concerns about predictor ascertainment (missing predictor data, predictors not handled as intended), outcome ascertainment (self-reported or including high trauma fractures), and analysis (high losses to follow-up/deaths not accounted for, inadequate number of fracture outcomes, short follow-up period, not accounting for competing mortality risk). ***Some concern about inconsistency:*** the point estimates for 12 studies (14 comparisons) agree with conclusions of poor performance, though 4 studies (4 comparisons) suggest acceptable calibration. Rated down 0.5 because it is believed that this inconsistency is at least partly related to risk of bias concerns. ***Some concern about indirectness:*** in 6 (Azagra 2016a, Bolland 2011, Czerwinski 2013, Goldshtein 2017, Tebe Cordomi 2013, Tremollieres 2010) studies, participants are likely to be higher risk than the general primary care population because they were referred for BMD testing, and one study (Ettinger 2013) enrolled participants who were likely to be healthier than the general population. None of the studies used the FRAX tool calibrated for Canada. ***Serious concerns for imprecision***: The confidence intervals of 6 studies cross the upper and lower threshold for being well calibrated, and most others are too wide to indicate a clinically meaningful over- or underestimation. ***No serious concerns for other considerations:*** no other concerns that would further impact certainty in the estimates. Publication bias not detected.

[d] <u>10-year hip fractures (lower risk of bias studies):</u> ***No serious concern about risk of bias:*** any concerns about risk of bias were quite minimal compared to other analyzed studies. The main potential concern was the use of proxy variables which might be expects to result in underestimation of fracture risk (i.e., the tool would be better calibrated than it appears from the analysis), thus we did not rate down. ***Serious concern for inconsistency:*** two comparisons are consistent with acceptable calibration while two others show substantial underestimation of the observed fracture risk; I^2 for the pooled effect is 92.7%. ***No serious concern about indirectness:*** the participants may be higher risk than the general primary care population because they all were referred for BMD testing. Based on other analyses the impact on the findings is unclear. All studies used the FRAX tool calibrated for Canada. ***Some concern about imprecision:*** the 95% confidence interval for the pooled effect includes the potential for acceptable calibration and substantial underestimation of observed fracture risk. This is at least partly due to inconsistency. ***No serious concerns for other considerations:*** no other concerns that would further impact certainty in the estimates.

[c] <u>10-year clinical fragility fractures (lower risk of bias studies):</u> ***No serious concern about risk of bias:*** any concerns about risk of bias were quite minimal compared to other analyzed studies. The main potential concern was the use of proxy variables which might be expects to result in underestimation of fracture risk (i.e., the tool would be better calibrated than it appears from the analysis), thus we did not rate down. ***Some concern for imprecision:*** the 95% confidence interval is somewhat wide for clinically meaningful estimates, ranging from 12 to 20% underestimation. ***No serious concern about indirectness:*** the participants may be higher risk than the general primary care population because they all were referred for BMD testing. Based on other analyses the impact on the findings is unclear. All studies used the FRAX tool calibrated for Canada. ***No serious concerns for inconsistency, or other considerations:*** no other concerns that would further impact certainty in the estimates.

[e] <u>5-year hip fractures (lower risk of bias studies):</u> ***Some concern about risk of bias:*** Use of the tool was not as intended (halved the 10-year risk estimate to obtain a 5-year risk). ***Serious concern for inconsistency:*** there is no evidence of consistency because there is only one study for this outcome, and demonstrated inconsistency in other analyses reinforces the need for several studies to support certainty in conclusions. ***No serious concern about indirectness:*** the participants may be higher risk than the general primary care population because they all were referred for BMD testing. Based on other analyses the impact on the findings is unclear. The study used the FRAX tool calibrated for Canada. ***Some concern about imprecision:*** the 95% confidence interval for males includes the potential that the tool is well calibrated or overestimates the observed fracture risk. ***No serious concerns for other considerations:*** no other concerns that would further impact certainty in the estimates.

KQ2: How accurate are screening tests at predicting fractures among adults ≥40 years?

[f] 5-year clinical fragility fractures (lower risk of bias studies): ***Some concern about risk of bias:*** Use of the tool was not as intended (halved the 10-year risk estimate to obtain a 5-year risk). ***Serious concern for inconsistency:*** there is no evidence of consistency because there is only one study for this outcome, and demonstrated inconsistency in other analyses reinforces the need for several studies to support certainty in conclusions. ***No serious concern about indirectness:*** the participants may be higher risk than the general primary care population because they all were referred for BMD testing. Based on other analyses the impact on the findings is unclear. The study used the FRAX tool calibrated for Canada. ***Some concern about imprecision:*** the 95% confidence interval for males includes the potential that the tool is well calibrated or underestimates the observed fracture risk. ***No serious concerns for other considerations:*** no other concerns that would further impact certainty in the estimates.

2.2 Contributing data

Calibration for the 10-year prediction of hip fractures

High risk of bias studies (none Canadian)

Study or Subgroup	log[Prediction Performance]	SE	Prediction Performance IV, Random, 95% CI
Pressman 2011 (80-89 years)	-1.4064971	0.0372765	0.24 [0.23, 0.26]
Pressman 2011 (70-79 years)	-1.0498221	0.0252229	0.35 [0.33, 0.37]
Pluskiewicz 2015	-0.99139824	0.49869961	0.37 [0.14, 0.99]
Marques 2017 (women)	-0.74473455	0.05882353	0.47 [0.42, 0.53]
Tamaki 2011	-0.69314718	0.4987715	0.50 [0.19, 1.33]
Marques 2017 (men)	-0.38623375	0.14285714	0.68 [0.51, 0.90]
Sornay-Rendu 2010	0.08555788	0.24014605	1.09 [0.68, 1.74]
Goldshtein 2017	0.10536052	0.05436657	1.11 [1.00, 1.24]
Ettinger 2013 (men)	0.2657032	0.0740851	1.30 [1.13, 1.51]
Bolland 2011	0.28185115	0.12977143	1.33 [1.03, 1.71]
Premaor 2013	0.35048297	0.04481737	1.42 [1.30, 1.55]
Melton III 2012 (women)	0.40546511	0.31030493	1.50 [0.82, 2.76]
Pressman 2011 (50-59 years)	0.4946962	0.0787791	1.64 [1.41, 1.91]
Melton III 2012 (men)	0.53062825	0.48395918	1.70 [0.66, 4.39]
Azagra 2016a	0.60431597	0.1864509	1.83 [1.27, 2.64]
Holloway 2018 (men)	0.68008993	0.26407674	1.97 [1.18, 3.31]
Pressman 2011 (60-69 years)	1.0788097	0.0389003	2.94 [2.73, 3.17]
Czerwinski 2013	1.2039728	0.17144661	3.33 [2.38, 4.66]

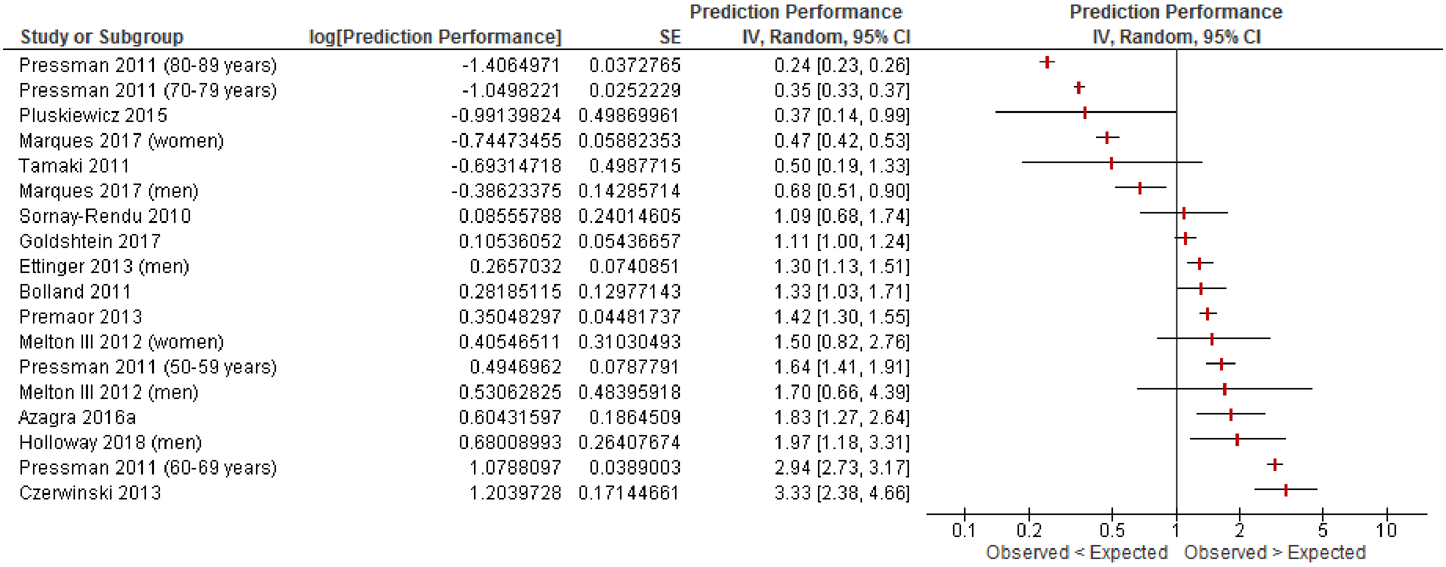

Lower risk of bias studies (all Canadian)

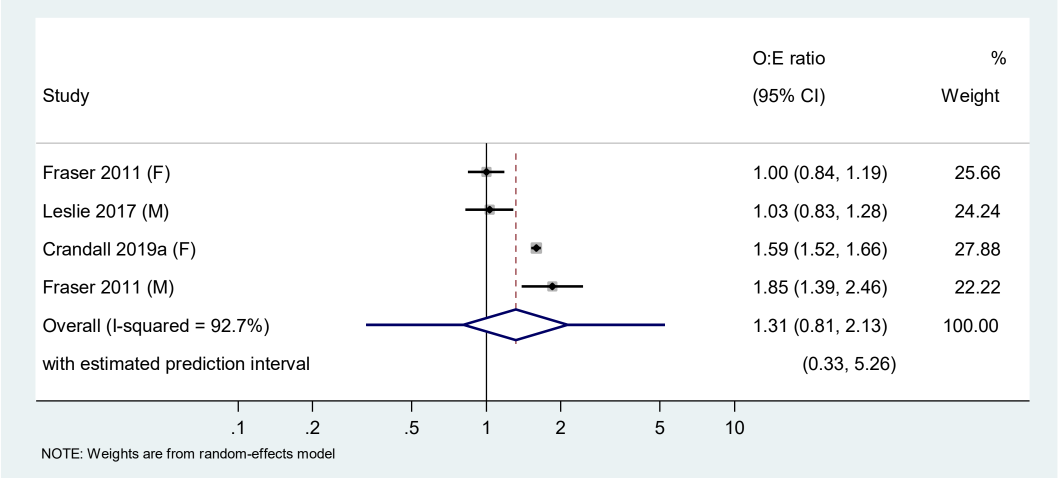

KQ2: How accurate are screening tests at predicting fractures among adults ≥40 years?

Calibration for the 10-year prediction of clinical fragility fractures (FRAX-defined major osteoporotic fractures)

High risk of bias studies (none Canadian)

Study or Subgroup	log[O-E Ratio]	SE	O-E Ratio IV, Random, 95% CI
Iki 2015 (men)	-0.8109302	0.21189745	0.44 [0.29, 0.67]
Tamaki 2019	-0.4618454	0.11948407	0.63 [0.50, 0.80]
Ettinger 2013 (men)	-0.096627	0.0478581	0.91 [0.83, 1.00]
Premaor 2013	-0.0695261	0.02889089	0.93 [0.88, 0.99]
Pluskiewicz 2015	0.0469549	0.14297003	1.05 [0.79, 1.39]
Tremollieres 2010	0.1541507	0.15087577	1.17 [0.87, 1.57]
Marques 2017 (men)	0.19633229	0.04347826	1.22 [1.12, 1.33]
Marques 2017 (women)	0.2394394	0.00862069	1.27 [1.25, 1.29]
Melton III 2012 (men)	0.3364722	0.25806146	1.40 [0.84, 2.32]
Tanaka 2010	0.4620355	0.11902382	1.59 [1.26, 2.00]
Melton III 2012 (women)	0.4700036	0.16495591	1.60 [1.16, 2.21]
Sornay-Rendu 2010	0.4718981	0.10507959	1.60 [1.30, 1.97]
Bolland 2011	0.6379315	0.06052749	1.89 [1.68, 2.13]
Goldshtein 2017	0.6641596	0.01957391	1.94 [1.87, 2.02]
Azagra 2016a	0.6981347	0.08796951	2.01 [1.69, 2.39]
Holloway 2018 (men)	0.8270444	0.13530688	2.29 [1.75, 2.98]
Czerwinski 2013	1.2843294	0.07244346	3.61 [3.13, 4.16]
Tebe Cordomi 2013	1.3609766	0.0715056	3.90 [3.39, 4.49]

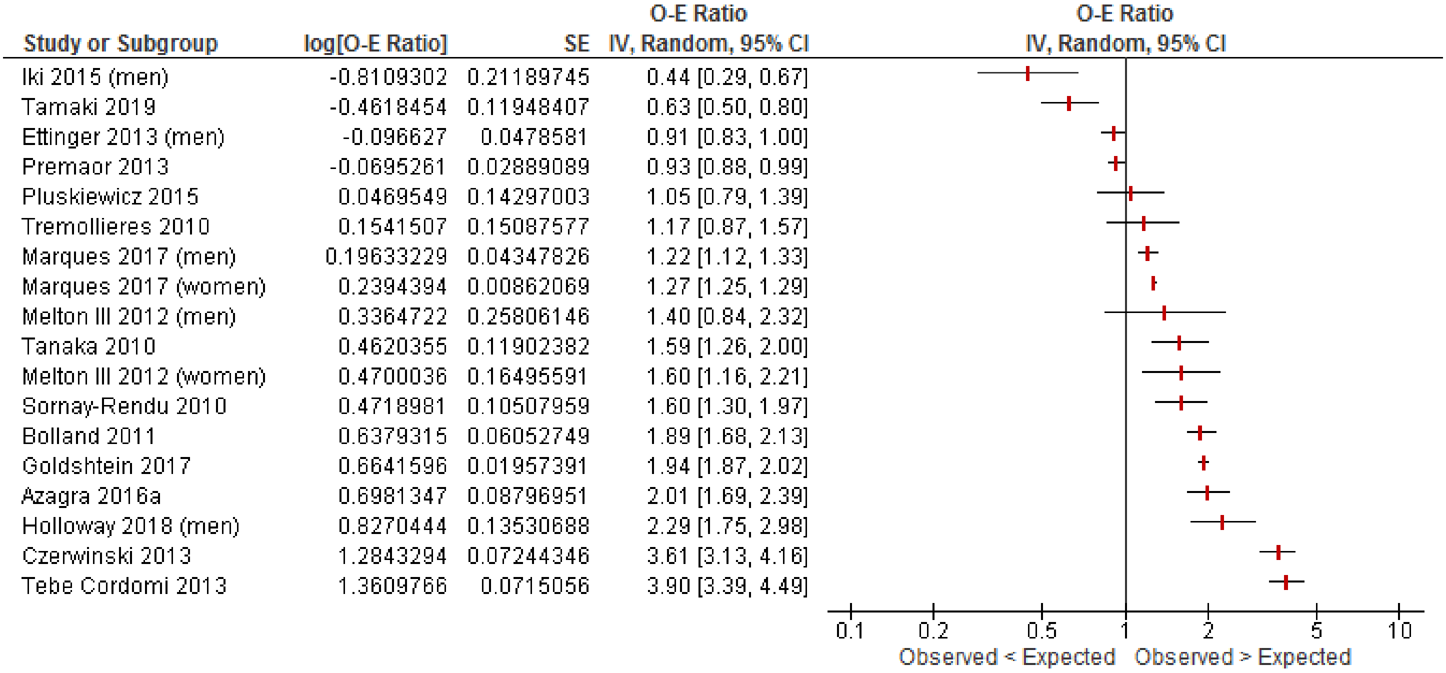

Lower risk of bias studies (all Canadian)

Study	O:E ratio (95% CI)	% Weight
Fraser 2011 (F)	1.11 (1.03, 1.20)	8.01
Leslie 2017 (M)	1.14 (1.03, 1.27)	4.23
Crandall 2019a (F)	1.16 (1.14, 1.19)	86.15
Fraser 2011 (M)	1.19 (1.00, 1.41)	1.61
Overall (I-squared = 0.0%)	1.16 (1.12, 1.20)	100.00
with estimated prediction interval	(1.10, 1.21)	

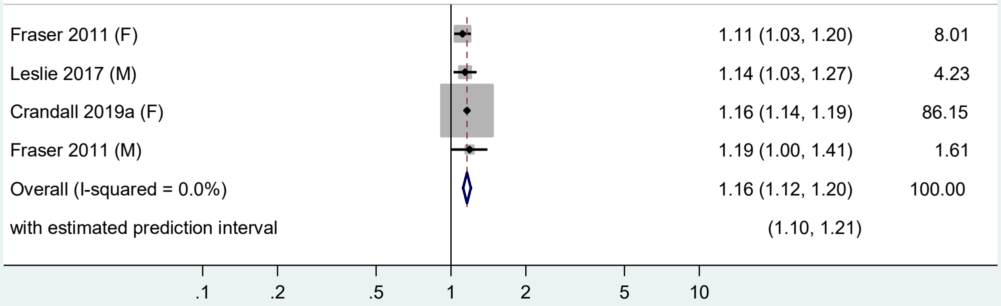

NOTE: Weights are from random-effects model

KQ2: How accurate are screening tests at predicting fractures among adults ≥40 years?

Summary of subgroup analyses

Outcome	N studies; comparisons	n	O:E ratio (95% CI)[a] or regression coefficient (95% CI)	I^2	Prediction interval
**1.0 10-year clinical fragility fractures**	**19; 22**	**110,391**	**1.38 (1.11, 1.72)[b]**	**100%**	**0.50, 3.83**
1.1 Between study subgroup: mean age (test for subgroup differences, p=0.228)					
<65 years	10; 11	79,825	1.51 (1.11, 2.06)	99%	0.53, 4.34
≥65 years	9; 10	30,566	1.26 (0.84, 1.89)	99%	0.33, 4.78
1.2 Between study subgroup: sex (test for subgroup differences, p=0.463)					
Males	7; 7	17,446	1.12 (0.72, 1.75)	97%	0.33, 3.83
Females	15; 15	92,945	1.52 (1.16, 1.99)	100%	0.52, 4.39
1.3 Between study sensitivity: risk of bias (test for subgroup differences, p=0.029)					
Unclear risk of bias	3; 4	61,156	1.16 (1.12, 1.20)	0%	1.10, 1.21
High risk of bias	16; 17	49,235	1.45 (1.09, 1.93)	99%	0.44, 4.77
1.4 Subgroup analysis using within-study data					
Females ≥65 years	7; 16	42,979	1.54 (1.27, 1.86)[b]	97%	0.75, 3.15
1.5 Meta-regressions					
Mean age	19; 22	110,391	0.97 (0.93, 1.001), p=0.109		
Mean baseline risk	19; 22	110,391	-0.03 (-0.08, 0.02), p=0.23		
**2.0 10-year hip fractures**	**16; 22**	**199,762**	**1.11 (0.81, 1.51)[b]**	**99%**	**0.27, 4.59**
2.1 Between study subgroup: mean age (test for subgroup differences, p=0.555)					
<65 years	8; 9	62,077	1.29 (0.81, 2.05)	94%	0.32, 5.17
≥65 years	9; 12	137,685	1.05 (0.64, 1.70)	100%	0.18, 5.93
2.2 Between study subgroup: sex (test for subgroup differences, p=0.686)					
Males	6; 6	15,641	1.27 (0.92, 1.99)	88%	0.40, 4.02
Females	13; 16	184,121	1.04 (0.69, 1.57)	100%	0.20, 5.45
2.3 Between study sensitivity: risk of bias (test for subgroup differences, p=0.418)					
Unclear risk of bias	3; 4	61,156	1.31 (0.81, 2.13)	93%	0.33, 5.26
High risk of bias	13; 17	138,606	1.09 (0.73, 1.63)	99%	0.21, 5.58
2.4 Subgroup analysis using within-study data					
Females ≥65 years	5; 14	97,875	1.09 (0.70, 1.70)[b]	99%	0.21, 5.71
2.5 Meta-regressions					
Mean age	16; 22	199,762	0.97 (0.93, 1.01), p=0.113		
Mean baseline risk	16; 22	199,762	-0.05 (-0.19, 0.10), p=0.51		
**3.0 5-year clinical fragility fractures**	**1; 2**	**62,275**<br>**6,455**	**F: 1.00 (0.97, 1.04)**<br>**M: 1.22 (1.07, 1.37)**	**NA**	**NA**
**4.0 5-year hip fractures**	**1; 2**	**62,275**<br>**6,455**	**F: 0.88 (0.81, 0.95)**<br>**M: 0.88 (0.65-1.10)**	**NA**	**NA**

F=female; O:E ratio=ratio of observed to expected (predicted) events; M=male; NA=not applicable
[a]Pooled using restricted maximum likelihood (REML) estimation and the Hartung-Knapp-Sidik-Jonkman (HKSJ) correction.
[b]The pooled estimate was suppressed in the summary of findings due to high unexplained heterogeneity.

Summary of data from calibration plots

Within-study data from calibration plots were inconsistent (data available on request). Ten studies provided calibration plot data for 10-year risk of clinical fragility fractures. In three studies (Tamaki 2019, Premaor 2013, Ettinger 2013), FRAX + BMD seemed well calibrated at all or most levels of baseline predicted risk. In two studies (Ettinger 2013, Crandall 2019a), FRAX + BMD over- or underestimated the observed fractures at low levels of fracture risk (≤3% in one study and 3.8% in another), but was well calibrated at higher levels of risk. In three studies (Azagra 2016a, Tebe Cordomi 2013, Bolland 2011), FRAX + BMD always underestimated the observed risk of clinical fragility fractures, but this underestimation seemed to improve at increasing levels of baseline risk in two of the studies (Tebe Cordomi 2013, Bolland 2011). One study (Iki 2015) showed

consistent overestimation of the observed fracture risk, while there was no apparent trend in the remaining studies (Fraser 2011, Melton 2012).

Eight studies provided calibration plot data for 10-year risk of hip fracture. In three studies (Fraser 2011, Ettinger 2013, Premaor 2013), FRAX + BMD always or usually underestimated the 10-year risk of hip fracture, with no clear trend related to baseline fracture risk. One study (Azagra 2016a) showed consistent underestimation with improving calibration as the level of baseline risk increased. In one study (Bolland 2011), FRAX + BMD always overestimated hip fracture risk, with no clear trend by level of baseline risk. One study (Crandall 2019a) showed acceptable calibration in the middle quintiles of baseline risk (0.6-4.1%), but underestimation at higher and lower levels of baseline risk. There was no apparent trend in the remaining two studies (Leslie 2010, Tamaki 2019).

No studies presented calibration plot data for 5-year risk of clinical fragility or hip fracture.

3. Clinical Garvan

3.1 GRADE Summary of Findings

Outcome* Studies; sample size	Findings	Certainty†	What does the evidence say?	Discrimination‡ [3] (pooled AUC, 95% CI)
**Clinical Garvan**				
10-y hip fractures<br>2 cohort; 67,923 [6, 29]	In one study, the tool substantially underestimated the observed fracture risk (O:E 3.63, 95% CI 3.31-3.97 [6]. A second study reported only the Hosmer-Lemeshow test ($p<0.0001$), indicating poor calibration [29].	VERY LOW[a]	Very uncertain for the conclusion of poor performance	F: 0.68 (NR)<br>M: 0.65 (NR)
10-y clinical fragility fractures<br>1 cohort; 5,063 [29]	In one study, the Hosmer-Lemeshow test was significant ($p=0.01014$), indicating poor calibration [29].	VERY LOW[b]	Very uncertain for the conclusion of poor performance	F: 0.66 (0.61-0.72)<br>M: NR
5-y hip fractures<br>1 cohort; 1,054,815 [21]	In one study, the tool substantially underestimated the observed fracture risk (O:E 2.17, 95% CI 2.16-2.17) [21].	LOW[c]	May underestimate by 116 to 117%	NR
5-y clinical fragility fractures<br>1 cohort; 9,393 [22]	In one study, the tool substantially underestimated the observed fracture risk (O:E 1.72, 95% CI 1.53-1.92).	VERY LOW[d]	Very uncertain for the conclusion of underestimation.	NR

BMD=bone mineral density; CI=confidence interval; F=female; M=male; NR=not reported; O:E ratio=ratio of observed to expected (predicted) events

*Rows for 5-year fractures have been omitted from the table when no studies were located that reported on this outcome.

† When our assessment of the certainty of evidence fell between levels, we assigned the level that best represented our actual certainty.

‡Extracted directly from the 2018 USPSTF systematic review by Viswanathan et al. [3]

Explanations:

[a] 10-year hip fractures: ***Serious concern about risk of bias:*** the included studies were at high risk of bias due to concerns about the predictors (missing data for a substantial portion of participants), and analysis methods (high losses to follow-up unaccounted for, length of follow-up exceeding the prediction interval, and in one study the O:E value is not provided and cannot be calculated from the available data). ***Some concern for inconsistency:*** there is minimal evidence of consistency because there are only two studies for this outcome, however both support the conclusion of poor calibration. ***Some concern about indirectness:*** in one of the studies, the enrolled population was healthier than the general primary care population. Based on other analyses, the impact on the findings is unclear. ***No serious concern about imprecision:*** the degree of underestimation appears substantial, but the range of potential underestimation is not compatible with clinically meaningful conclusions. ***No serious concerns for other considerations:*** no other concerns that would further impact certainty in the estimates.

[b] 10-year clinical fragility fractures: ***Serious concern about risk of bias:*** the one included study is at high risk of bias due to missing predictor data for several participants, length of follow-up that substantially exceeds the prediction interval, and concerns about the analysis because an O:E value is not provided and cannot be calculated from the available data. ***Serious concern for inconsistency:*** there is no evidence of consistency because there is only one study for this outcome, and demonstrated inconsistency in other analyses reinforces the need for several studies to support certainty in conclusions. ***No***

serious concerns for indirectness: the study population is well aligned with the review question. ***Some concern about imprecision:*** the precision of the estimate is unknown, thus clinically meaningful conclusions cannot be drawn. ***No serious concerns for other considerations:*** no other concerns that would further impact certainty in the estimates.

[c] <u>5-year hip fractures:</u> ***Serious concern about risk of bias:*** the analysis in the one included study (Dagan 2017) did not account for competing risk of mortality and use of the tool was not as intended (halved the 10-year risk estimate to obtain a 5-year risk). ***Serious concern for inconsistency:*** there is no evidence of consistency because there is only one study for this outcome, and demonstrated inconsistency in other analyses reinforces the need for several studies to support certainty in conclusions. ***Some concern about indirectness:*** the cohort includes an unspecified number of participants who previously used anti-osteoporosis medication. Based on other analyses, the impact on the findings is unclear. ***No serious concern about imprecision:*** the confidence interval allows for clinically meaningful conclusion about the range of potential underestimation. ***No serious concerns for other considerations:*** no other concerns that would further impact certainty in the estimates.

[d] <u>5-year clinical fragility fractures:</u> ***Serious concern about risk of bias:*** the analysis in the one included study did not have adequate predictor data nor account for competing risk of mortality, and use of the tool was not as intended (halved the 10-year risk estimate to obtain a 5-year risk). ***Serious concern for inconsistency:*** there is no evidence of consistency because there is only one study for this outcome, and demonstrated inconsistency in other analyses reinforces the need for several studies to support certainty in conclusions. ***No serious concern about indirectness:*** the study used the FRAX tool calibrated for Canada and the population is quite representative. ***Serious concern about imprecision:*** the confidence interval does not allow for a clinically meaningful conclusion about the range of potential underestimation. ***No serious concerns for other considerations:*** no other concerns that would further impact certainty in the estimates.

3.2 Contributing data

Summary of data from calibration plots

Within-study data from calibration plots were unavailable for 10-year clinical fragility fractures and 10-year hip fractures. One study (Desbiens 2020) reported on 5-year clinical fragility fractures, finding underestimation at lower predicted probabilities, good calibration at moderate risk (5%) and overestimation at higher risk. One study (Dagan 2017) provided calibration plot data for 5-year hip fractures. This study showed substantial underestimation of observed 5-year hip fracture risk in females, with calibration improving as the level of baseline risk increased. A similar trend was observed in males, though the degree of underestimation was lesser, with prediction of 5-year hip fractures being in the acceptable range at a baseline risk of 4.0-5.8% (deciles 8-9), and overestimated in the highest decile (10.4% baseline risk).

4. Garvan + BMD

4.1 GRADE Summary of Findings

Outcome* Studies; sample size	Findings	Certainty†	What does the evidence say?	Discrimination‡ [3] (pooled AUC, 95% CI)
**Garvan + BMD**				
10-y hip fractures<br>5 cohort; 11,869 [5, 11, 29-31]	Most studies show poor calibration and are inconsistent. Most often, the tool overestimated fracture risk to an important magnitude, though the degree of overestimation is highly variable (n = 3 studies, 4 comparisons, O:E 0.10 to 0.66). Inconsistency was not well explained by subgroup analyses.	VERY LOW[a]	Very uncertain for the conclusion of poor performance	F: 0.73 (0.66-0.79)<br>M: 0.79 (NR)
10-y clinical fragility fractures<br>5 cohort; 11,733 [5, 11, 29-31]	Most studies show poor calibration and are inconsistent. Most often, the tool over- (n = 2 studies, 2 comparisons; O:E 0.34 to 0.74) or underestimated (n = 1 study, 1 comparison; O:E 1.65) the observed fracture risk. One study reported only the Hosmer-Lemeshow test (p=0.0001) [29], indicating poor calibration. Inconsistency was not well explained by subgroup analyses.	VERY LOW[b]	Very uncertain for the conclusion of poor performance	F: 0.68 (0.64-0.71)<br>M: 0.75 (NR)

BMD=bone mineral density; CI=confidence interval; F=female; M=male; NR=not reported; O:E ratio=ratio of observed to expected (predicted) events

*Rows for 5-year fractures have been omitted from the table when no studies were located that reported on this outcome.

KQ2: How accurate are screening tests at predicting fractures among adults ≥40 years?

† When our assessment of the certainty of evidence fell between levels, we assigned the level that best represented our actual certainty.
‡Extracted directly from the 2018 USPSTF systematic review by Viswanathan et al. [3]

Explanations:
[b] <u>10-year hip fractures</u>: ***Serious concern about risk of bias:*** all studies were at high risk of bias primarily due to concerns about the predictors (missing for several participants), outcome ascertainment (fractures not verified), and appropriateness of the analysis methods (losses to follow up and deaths not accounted for, follow-up shorter or longer than the prediction interval, competing risks not accounted for. ***Some concern about inconsistency***: most point estimates agree with conclusions of poor performance (overestimating risk by an important magnitude), though 2 studies suggest acceptable calibration. Rated down 0.5 because it is believed that this inconsistency is at least partly related to risk of bias concerns. ***No serious concern about indirectness:*** the study populations overall seem to align with the review question. ***Serious concerns about imprecision:*** The confidence intervals of 4 studies cross the upper and lower threshold for being well calibrated, and others are too wide to indicate a clinically meaningful overestimation.. ***No serious concerns for other considerations:*** no other concerns that would further impact certainty in the estimates.

[a] <u>10-year clinical fragility fractures:</u> ***Serious concern about risk of bias:*** all studies were at high risk of bias primarily due to concerns about the predictors (missing for several participants), outcome ascertainment (fractures not verified), and appropriateness of the analysis methods (losses to follow up and deaths not accounted for, follow-up shorter or longer than the prediction interval, competing risks not accounted for). ***Some concern about inconsistency***: most point estimates agree with conclusions of poor performance (either under- or overestimating risk by an important magnitude), though 2 studies suggest acceptable calibration, and the direction of poor performance (under- or overestimating) is unclear. Rated down 0.5 because it is believed that this inconsistency is at least partly related to risk of bias concerns. ***No serious concern about indirectness:*** the study populations overall seem to align with the review question. ***Serious concerns about imprecision:*** The confidence intervals of 2 studies cross the upper and lower threshold for being well calibrated, and most others are too wide to indicate a clinically meaningful under- or overestimation. ***No serious concerns for other considerations:*** no other concerns that would further impact certainty in the estimates.

4.2 Contributing data

Calibration for the prediction of 10-year hip fractures

Study or Subgroup	log[Prediction Performance]	SE	Prediction Performance IV, Random, 95% CI
Pluskiewicz 2015	-2.26436392	0.49869961	0.10 [0.04, 0.28]
Langsetmo (women) 2011	-0.69849002	0.09143773	0.50 [0.42, 0.59]
Reyes Dominguez 2017	-0.59608547	0.70123857	0.55 [0.14, 2.18]
Bolland 2011	-0.41129603	0.12977143	0.66 [0.51, 0.85]
Langsetmo (men) 2011	-0.10285739	0.15912797	0.90 [0.66, 1.23]
Gourlay 2017 (men)	-0.04445176	0.07475675	0.96 [0.83, 1.11]

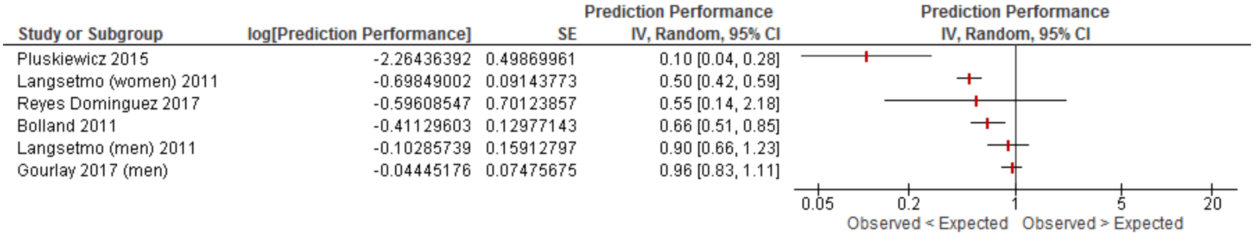

Calibration for the prediction of 10-year clinical fragility fractures

Study or Subgroup	log[O-E Ratio]	SE	O-E Ratio IV, Random, 95% CI
Pluskiewicz 2015	-1.0804778	0.14297003	0.34 [0.26, 0.45]
Langsetmo (men) 2011	-0.3005302	0.08083566	0.74 [0.63, 0.87]
Langsetmo (women) 2011	-0.1235278	0.03529684	0.88 [0.82, 0.95]
Bolland 2011	0.0108109	0.0536749	1.01 [0.91, 1.12]
Reyes Dominguez 2017	0.5025268	0.15833152	1.65 [1.21, 2.25]

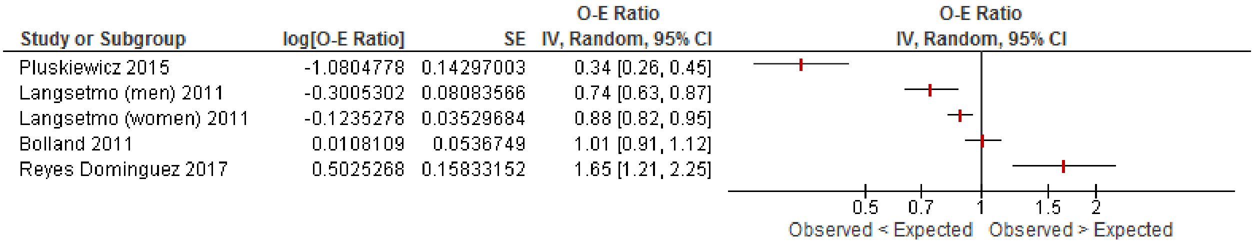

KQ2: How accurate are screening tests at predicting fractures among adults ≥40 years?

Summary of data from calibration plots

Within-study data from calibration plots were inconsistent (data available on request). Two studies provided calibration plot data for 10-year risk of clinical fragility fractures. In one (Langsetmo 2011), Garvan + BMD had acceptable calibration at all levels of baseline risk. In another, the tool underestimated the risk of fracture at lower levels of baseline risk (≤13.2%), but seemed well calibrated in higher risk groups, except in the highest decile where risk was overestimated.

Three studies provided calibration plot data for 10-year risk of hip fractures. In one study (Gourlay 2017), Garvan + BMD overestimated the observed fracture risk in the lower deciles of predicted risk but underestimated in the upper deciles. Conversely, another study (Bolland 2011) showed the opposite findings. Finally, one study (Langsetmo 2011 showed no clear trend in males, but that the tool consistently overestimated the risk of hip fracture in females.

No studies presented calibration plot data for 5-year risk of clinical fragility or hip fracture.

5. Canadian Association of Radiologists and Osteoporosis Canada Risk Assessment tool (CAROC)

5.1 GRADE Summary of Findings

Outcome* Studies; sample size	Findings	Certainty†	What does the evidence say?	Discrimination‡ [3] (pooled AUC, 95% CI)
**CAROC (includes BMD)**				
10-y hip fractures	No studies reported this outcome.	Not applicable		NR
10-y clinical fragility fractures 1 cohort; 34,060 [32]	One study did not report an O:E ratio. Observed fracture risk (95% CI) was 6.4 (6.0-6.8)% in the low risk (<10%) group, 13.8 (13.1-14.5)% in the moderate risk group (10-20%), and 23.8 (22.5-25.0)% in the high risk group (>20%).	LOW[a]	May be adequately calibrated to predict a category of risk.	NR

BMD=bone mineral density; CI=confidence interval; NR=not reported; O:E ratio=ratio of observed to expected (predicted) events

*Rows for 5-year fractures have been omitted from the table when no studies were located that reported on this outcome.

† When our assessment of the certainty of evidence fell between levels, we assigned the level that best represented our actual certainty.

‡Extracted directly from the 2018 USPSTF systematic review by Viswanathan et al. [3]

Explanations:

[a] 10-year clinical fragility fractures: ***Some concern about risk of bias:*** the one included study was at high risk of bias primarily due to concerns about outcome ascertainment (may include non-clinical vertebral fractures) and about the analysis because an O:E value is not provided and cannot be calculated from the available data. ***Serious concern for inconsistency:*** there is no evidence of consistency because there is only one study for this outcome, and demonstrated inconsistency in other analyses reinforces the need for several studies to support certainty in conclusions. ***Some concern about indirectness:*** all participants are those who were referred for BMD testing (higher risk). Based on other analyses, the impact on the conclusion is unclear. ***No serious concern about imprecision:*** though imprecision cannot be adequately assessed using the available data, this is already accounted for in concerns about risk of bias. ***No serious concerns for other considerations:*** no other concerns that would further impact certainty in the estimates.

6. QFracture

6.1 GRADE Summary of Findings

Outcome* Studies; sample size	Findings	Certainty†	What does the evidence say?	Discrimination‡ [3] (pooled AUC, 95% CI)
**QFracture (no BMD)**				
10-y hip fractures 1 cohort; 5,200 [29]	In one study, the O:E was not reported. The Hosmer-Lemeshow test was significant (p<0.0001), indicating poor calibration.	VERY LOW[a]	Very uncertain for the conclusion of poor performance.	NR
10-y clinical fragility fractures 1 cohort; 5,063 [29]	In one study, the O:E was not reported. The Hosmer-Lemeshow test was significant (p=0.0001), indicating poor calibration.	VERY LOW[b]	Very uncertain for the conclusion of poor performance.	NR

KQ2: How accurate are screening tests at predicting fractures among adults ≥40 years?

5-y hip fractures<br>1 cohort; 1,054,815 [21]	In one study, the tool underestimated the observed fracture risk (O:E 1.42, 95% CI 1.41-1.42).	LOW[c]	May underestimate by 40 to 42%.	NR
5-y clinical fragility fractures<br>1 cohort; 9,393 [22]	In one study, the tool substantially underestimated the observed fracture risk (O:E 2.03, 95% CI 1.71-2.42).	VERY LOW[d]	Very uncertain for the conclusion of underestimation.	NR

BMD=bone mineral density; CI=confidence interval; NR=not reported; O:E ratio=ratio of observed to expected (predicted) events
*Rows for 5-year fractures have been omitted from the table when no studies were located that reported on this outcome.
† When our assessment of the certainty of evidence fell between levels, we assigned the level that best represented our actual certainty.
‡Extracted directly from the 2018 USPSTF systematic review by Viswanathan et al. [3]

Explanations:

[a] 10-year hip fractures: ***Very serious concern about risk of bias:*** the one included study was at high risk of bias due to missing predictor data for several participants, length of follow-up that substantially exceeds the prediction interval, and concerns about the analysis because an O:E value is not provided and cannot be calculated from the available data. ***Serious concern for inconsistency:*** there is no evidence of consistency because there is only one study for this outcome, and demonstrated inconsistency in other analyses reinforces the need for several studies to support certainty in conclusions. ***No serious concern for indirectness:*** the study population is well aligned with the review question. ***Some concern for imprecision:*** the precision cannot be ascertained from the available data. ***No serious concerns for other considerations:*** no other concerns that would further impact certainty in the estimates.

[b] 10-year clinical fragility fractures: ***Very serious concern about risk of bias:*** the one included study was at high risk of bias due to missing predictor data for several participants, length of follow-up that substantially exceeds the prediction interval, and concerns about the analysis because an O:E value is not provided and cannot be calculated from the available data. ***Serious concern for inconsistency:*** there is no evidence of consistency because there is only one study for this outcome, and demonstrated inconsistency in other analyses reinforces the need for several studies to support certainty in conclusions. ***No serious concern for indirectness:*** the study population is well aligned with the review question. ***Some concern for imprecision:*** the precision cannot be ascertained from the available data. ***No serious concerns for other considerations:*** no other concerns that would further impact certainty in the estimates.

[c] 5-year hip fractures: ***Serious concern about risk of bias:*** the analysis in the one included study (Dagan 2017) did not account for competing risk of mortality and use of the tool was not as intended (halved the 10-year risk estimate to obtain a 5-year risk). ***Serious concern for inconsistency:*** there is no evidence of consistency because there is only one study for this outcome, and demonstrated inconsistency in other analyses reinforces the need for several studies to support certainty in conclusions. ***Some concern about indirectness:*** the cohort includes an unspecified number of participants who previously used anti-osteoporosis medication. Based on other analyses, the impact on the findings is unclear. ***No serious concern about imprecision:*** the precision is adequate to draw clinically meaningful conclusions. ***No serious concerns for other considerations:*** no other concerns that would further impact certainty in the estimates.

[d] 5-year clinical fragility fractures: ***Serious concern about risk of bias:*** the analysis in the one included study did not have adequate predictor data nor account for competing risk of mortality, and use of the tool was not as intended (halved the 10-year risk estimate to obtain a 5-year risk). ***Serious concern for inconsistency:*** there is no evidence of consistency because there is only one study for this outcome, and demonstrated inconsistency in other analyses reinforces the need for several studies to support certainty in conclusions. ***No serious concern about indirectness:*** the study used the FRAX tool calibrated for Canada and the population is quite representative. ***Serious concern about imprecision:*** the confidence interval does not allow for a clinically meaningful conclusion about the range of potential underestimation. ***No serious concerns for other considerations:*** no other concerns that would further impact certainty in the estimates.

KQ2: How accurate are screening tests at predicting fractures among adults ≥40 years?

6.2 Contributing data

Summary of data from calibration plots

One study (Gourlay 2017) presented calibration plot data for 10-year hip fractures (data available on request), showing that QFracture overestimated the observed risk of fracture at the lower deciles of predicted baseline risk, but underestimated in the upper deciles. One study (Dagan 2017) presented calibration data for 5-year hip fractures, showing substantial overestimation of fracture risk, with this overestimation being most prominent at the lower deciles of baseline risk.

One study (Desbiens 2020) reported calibration plot data for 5-year clinical fragility fracture, finding underestimation at all baseline levels of risk. No studies presented calibration plot data for 10-year clinical fragility fractures.

7. Fracture and Immobilization Score (FRISC, includes BMD)

7.1 GRADE Summary of Findings

Outcome* Studies; sample size	Findings	Certainty†	What does the evidence say?	Discrimination‡ [3] (pooled AUC, 95% CI)
**Fracture and Immobilization Score (FRISC; includes BMD)**				
10-y hip fractures	No studies reported this outcome.	Not applicable		NR
10-y clinical fragility fractures [26]<br>1 cohort; 400	In one study, FRISC was imprecise for overestimation of the 10-year risk of clinical fragility fracture (O:E 0.74, 95% CI 0.59-0.93).	VERY LOW[a]	Very uncertain for the conclusion of poor performance	F: 0.73 (NR)

BMD=bone mineral density; CI=confidence interval; F=female; NR=not reported; O:E ratio=ratio of observed to expected (predicted) events
*Rows for 5-year fractures have been omitted from the table when no studies were located that reported on this outcome.
† When our assessment of the certainty of evidence fell between levels, we assigned the level that best represented our actual certainty.
‡Extracted directly from the 2018 USPSTF systematic review by Viswanathan et al. [3]

Explanations:

[a] 10-year clinical fragility fractures: ***Serious concern about risk of bias:*** the one included study was at high risk of bias due unclear ascertainment of the outcome (may include non-clinical vertebral fractures) and competing risk of mortality not accounted for in the analysis. ***Serious concern for inconsistency:*** there is no evidence of consistency because there is only one study for this outcome, and demonstrated inconsistency in other analyses reinforces the need for several studies to support certainty in conclusions. ***No serious concerns about indirectness***: the study population is well aligned with the review question. ***Serious concern about imprecision:*** the confidence interval includes values aligned with overestimation and good calibration. ***No serious concerns for other considerations:*** no other concerns that would further impact certainty in the estimates.

8. Clinical Fracture Risk Calculator (FRC)

8.1 GRADE Summary of Findings

Outcome* Studies; sample size	Findings	Certainty†	What does the evidence say?	Discrimination‡ [3] (pooled AUC, 95% CI)
**Clinical Fracture Risk Calculator (FRC)**				
10-y hip fractures<br>2 cohort; 100,382 [33, 34]	Calibration findings were inconsistent. In one study the clinical FRC underestimated the observed 10-year risk of hip fracture in women (O:E 1.44, 95% CI not reported). In another study, FRC was well calibrated for the 10-year prediction of hip fracture in men (O:E 0.97, 95% CI not reported).	VERY LOW[a]	Very uncertain for the conclusion of poor performance	F: 0.83 (0.82-0.84)<br>M: 0.71 (NR)
10-y clinical fragility fractures<br>1 cohort; 5,893 [33]	In one study, the clinical FRC was well calibrated for the 10-year prediction of clinical fragility fracture (O:E 0.95, 95% CI not reported) in men.	VERY LOW[b]	Very uncertain for the conclusion of acceptable calibration in men	F: NR<br>M: 0.66 (NR)

BMD=bone mineral density; CI=confidence interval; F=female; M=male; NR=not reported; O:E ratio=ratio of observed to expected (predicted) events
*Rows for 5-year fractures have been omitted from the table when no studies were located that reported on this outcome.
† When our assessment of the certainty of evidence fell between levels, we assigned the level that best represented our actual certainty.
‡Extracted directly from the 2018 USPSTF systematic review by Viswanathan et al. [3]

KQ2: How accurate are screening tests at predicting fractures among adults ≥40 years?

Explanations:

[a] <u>10-year hip fractures:</u> ***Serious concern about risk of bias:*** the included studies are at high risk of bias, primarily due to inappropriate use of predictors or missing predictor values, losses to follow-up and deaths were excluded from the analysis or not adequately account for, and participants who started bisphosphonates after baseline were censored. ***Serious concern about inconsistency:*** there is some evidence of inconsistency since the two included studies show differing conclusions. In addition, demonstrated inconsistency in other analyses reinforces the need for several studies to support certainty in conclusions. ***Some concern about indirectness***: the enrolled participants were healthier than the general population in one study (Ettinger 2012) and were all referred for BMD testing in the other (Lo 2011; higher risk). ***Some concern about imprecision:*** no indication of precision is provided in the contributing studies, thus it is not possible to draw clinically meaningful conclusions. ***No serious concerns for other considerations:*** no other concerns that would further impact certainty in the estimates.

[b] <u>10-year clinical fragility fractures:</u> ***Serious concern about risk of bias:*** the one included study is at high risk of bias because predictor values were missing for 27% of participants, losses to follow-up and deaths were excluded from the analysis (32%), did not account for competing risk of mortality in the analysis, and participants who started bisphosphonates after baseline were censored. ***Serious concern about inconsistency:*** there is no evidence of consistency because there is only one study for this outcome, and demonstrated inconsistency in other analyses reinforces the need for several studies to support certainty in conclusions. ***Some concern about indirectness:*** the enrolled participants were healthier than the general population. Based on other analyses, the impact on the findings is unclear. ***Some concern about imprecision:*** no indication of precision is provided in the study, thus it is not possible to draw clinically meaningful conclusions. ***No serious concerns for other considerations:*** no other concerns that would further impact certainty in the estimates.

8.2 Contributing data

Summary of data from calibration plots

One study (Ettinger 2012) presented calibration plot data for 10-year clinical fragility fractures (data available on request), showing acceptable calibration at all levels of baseline risk. Two studies provided calibration data for 10-year hip fractures. One (Ettinger 2012) showed acceptable calibration at all levels of baseline risk, while the other showed acceptable calibration only in the lowest two sextiles of baseline risk, with underestimation of risk in the remaining sextiles.

No studies presented calibration plot data for 5-year clinical fragility or hip fractures.

9. Fracture Risk Calculator (FRC) + BMD

9.1 GRADE Summary of Findings

Outcome* Studies; sample size	Findings	Certainty†	What does the evidence say?	Discrimination‡ [3] (pooled AUC, 95% CI)
**FRC + BMD**				
10-y hip fractures<br>2 cohort; 100,382 [33, 34]	Calibration findings were inconsistent. In one study, the FRC + BMD underestimated the observed 10 year risk of hip fracture in women (O:E 1.50, 95% CI not reported). In another study, FRC was well calibrated for the 10-year prediction of hip fractures in men (O:E 1.0, 95% CI not reported).	VERY LOW[a]	Very uncertain for the conclusion of poor performance	F: 0.85 (0.84-0.86)<br>M: 0.79 (NR)
10-y clinical fragility fractures<br>1 cohort; 5,893 [33]	In one study, the FRC + BMD was well calibrated for the 10-year prediction of clinical fragility fracture in men (O:E 0.96, 95% CI not reported).	VERY LOW[b]	Very uncertain for the conclusion of acceptable calibration	F: NR<br>M: 0.70 (NR)

BMD=bone mineral density; CI=confidence interval; F=female; M=male; NR=not reported; O:E ratio=ratio of observed to expected (predicted) events

*Rows for 5-year fractures have been omitted from the table when no studies were located that reported on this outcome.

† When our assessment of the certainty of evidence fell between levels, we assigned the level that best represented our actual certainty.

‡Extracted directly from the 2018 USPSTF systematic review by Viswanathan et al. [3]

KQ2: How accurate are screening tests at predicting fractures among adults ≥40 years?

Explanations:

[a] 10-year hip fractures: **Serious concern about risk of bias:** the included studies are at high risk of bias, primarily due to inappropriate use of predictors or missing predictor values, losses to follow-up and deaths were excluded from the analysis or not adequately account for, and participants who started bisphosphonates after baseline were censored. ***Serious concern about inconsistency:*** there is some evidence of inconsistency since the two included studies show differing conclusions. In addition, demonstrated inconsistency in other analyses reinforces the need for several studies to support certainty in conclusions. ***Some concern about indirectness***: the enrolled participants were healthier than the general population in one study (Ettinger 2012) and were all referred for BMD testing in the other (Lo 2011; higher risk). ***Some concern about imprecision:*** no indication of precision is provided in the contributing studies, thus it is not possible to draw clinically meaningful conclusions. ***No serious concerns for other considerations:*** no other concerns that would further impact certainty in the estimates.

[b] 10-year clinical fragility fractures: ***Serious concern about risk of bias:*** the one included study is at high risk of bias because predictor values were missing for 27% of participants, losses to follow-up and deaths were excluded from the analysis (32%), did not account for competing risk of mortality in the analysis, and participants who started bisphosphonates after baseline were censored. ***Serious concern about inconsistency:*** there is no evidence of consistency because there is only one study for this outcome, and demonstrated inconsistency in other analyses reinforces the need for several studies to support certainty in conclusions. ***Some concern about indirectness:*** the enrolled participants were healthier than the general population. Based on other analyses, the impact on the findings is unclear. ***Some concern about imprecision:*** no indication of precision is provided in the study, thus it is not possible to draw clinically meaningful conclusions. ***No serious concerns for other considerations:*** no other concerns that would further impact certainty in the estimates.

9.2 Contributing data

Summary of data from calibration plots: One study (Ettinger 2012) presented calibration plot data for 10-year clinical fragility fractures (data available on request), showing acceptable calibration at all levels of baseline risk. Two studies provided calibration data for 10-year hip fractures. One (Ettinger 2012) showed acceptable calibration at most levels of baseline risk, while the other showed acceptable calibration only in the lowest two sextiles of baseline risk, with underestimation of risk in the remaining sextiles.

No studies presented calibration plot data for 5-year clinical fragility or hip fractures.

KQ3a: What are the benefits of pharmacologic treatments to prevent fragility fractures among adults ≥ 40 years?

EVIDENCE SUMMARY FOR KQ3a ON THE BENEFITS OF PHARMACOLOGIC TREATMENTS

1. Alendronate vs. placebo

1.1 GRADE Summary of Findings

Outcome & Study approach	Studies; sample size	Follow-up (y)	Assumed population risk*	Absolute effects (95% CI)	Certainty	What happens?
**Alendronate vs. placebo (postmenopausal females)**						
Hip fractures<br>Intention to treat	7 RCT; 9,226 [35-42]	1 to 4	Study data:<br>8 in 1000	2.1 fewer in 1000<br>(4.5 fewer to 1.9 more)	LOW[a]	May not reduce
			General F ≥65 y:<br>20 in 1000	5.3 fewer in 1000<br>(11.3 fewer to 4.7 more)		
Clinical fragility fractures<br>Intention to treat	8 RCT; 8,854 [35, 37-40, 42-47]	1 to 4	Study data:<br>96 in 1000	14.7 fewer in 1000<br>(24.5 fewer to 2.6 fewer)	MODERATE[b]	Probably reduces
			General F ≥65 y:<br>202 in 1000	28.4 fewer in 1000<br>(47.8 fewer to 4.9 fewer)		
Clinical vertebral fractures	The evidence from 5 RCTs (n=6,324) [35-38, 41, 46] is very uncertain.				VERY LOW[c]	Very uncertain
All-cause mortality	The evidence from 4 RCTs (n=5,272) [36, 37, 41, 47, 48] is very uncertain.				VERY LOW[d]	Very uncertain

CI=confidence interval; RCT=randomized controlled trial; y=years

* The effects without screening for the general risk population are estimated from PRIOR et al. [49], based on 10 year follow-up. Data for the general population <65 years is not included in the summary table (available on request).

Explanations:

[a] ***No major concerns about risk of bias***: no major risk of bias concerns for main contributing study (Cummings 1998). ***Some concern about inconsistency:*** lack of evidence of consistency because the analysis hinges primarily on the one large (adequately powered) trial (Cummings 1998). ***Serious concern about indirectness:*** in one of the trials (Liberman 1995) it is possible that the fractures were the result of trauma (i.e., not fragility fractures); in all but Cummings 1998, it is unclear how the outcome is defined. The one adequately powered trial used a dose (5 mg/day for 24 months followed by 10 mg/day for 24 months) that is not approved for use in Canada (lower than the approved dose and showing no difference). ***Some concern about imprecision:*** the number of events does not meet the optimal information size (<300), though the sample size is large. The confidence interval includes the potential for important benefit or no difference. ***No serious concern for other considerations:*** no other concerns that would further reduce our confidence in the findings.

[b] ***No major concerns about risk of bias***: no major risk of bias concerns for main contributing studies (Cummings 1998, Liberman 1995, Pols 1999). ***Some concerns about inconsistency:*** Presence of an effect is inconsistent among the trials. ***Some concern about indirectness:*** the prevalence of prior fracture in two of the larger trials (Liberman 1995, Pols 1999) is unknown and in one of the trials (Liberman 1995) it is possible that the fractures were the result of trauma (i.e., not fragility fractures). In 3 trials (Bell 2002, Hosking 2003, Lewiecki 2007) the outcome was self-reported and may include non-clinical vertebral fractures, but the findings were robust to sensitivity analysis removing these trials (did not rate down). The largest trial used a dose (5 mg/day for 24 months followed by 10 mg/day for 24 months) that is not approved for use in Canada (lower than the approved dose), but the pooled estimate still shows benefit (did not rate down). ***No serious concern about imprecision:*** the sample size is adequate and confidence interval is precise for benefit of alendronate. ***No serious concern for other considerations:*** no other concerns that would further reduce our confidence in the findings.

[c] ***Some concerns about risk of bias***: no major risk of bias concerns for the one study contributing events (Cummings 1998). The findings could be biased because several of the trials of alendronate did not report on this outcome specifically. ***Some concerns about inconsistency:*** there is no evidence of consistency because only one of the trials was large enough to show any events. ***Serious concern about indirectness:*** The largest trial used a dose (5 mg/day for 24 months followed by 10 mg/day for 24 months) that is not approved for use in Canada (lower than the approved dose). ***Some concern about imprecision:*** the number of events does not meet the optimal information size (<300), though the sample size is large. The confidence interval includes the potential for important benefit or no difference. ***No serious concern for other considerations:*** no other concerns that would further reduce our confidence in the findings.

[d] ***Serious concern about risk of bias***: in the one adequately powered trial (Cummings 1998), it is unclear how the outcome was collected (may not have been collected systematically). Selective reporting strongly suspected, since only 4 of the 11 trials of alendronate (36%) reported on this outcome, and appears to be collected passively in these trials. ***Some concern about inconsistency:*** lack of evidence of consistency because the analysis hinges primarily on the one large trial (Cummings 1998). ***Some concerns about indirectness:*** the main contributing trial (Cummings 1998) provided a dose of alendronate (5 mg/day for 24 months followed by 10 mg/day for 24 months) that is not approved for use in Canada (lower than the approved dose and showing no difference). ***Serious concern about imprecision:*** the number of events does not meet the optimal information size (n<300), though the sample size is large. The confidence interval is very wide and includes the potential for both important benefit and harm. ***No serious concern for other considerations:*** no other concerns that would further reduce our confidence in the findings.

1.2 Contributing data
Alendronate vs. placebo – hip fractures

Females

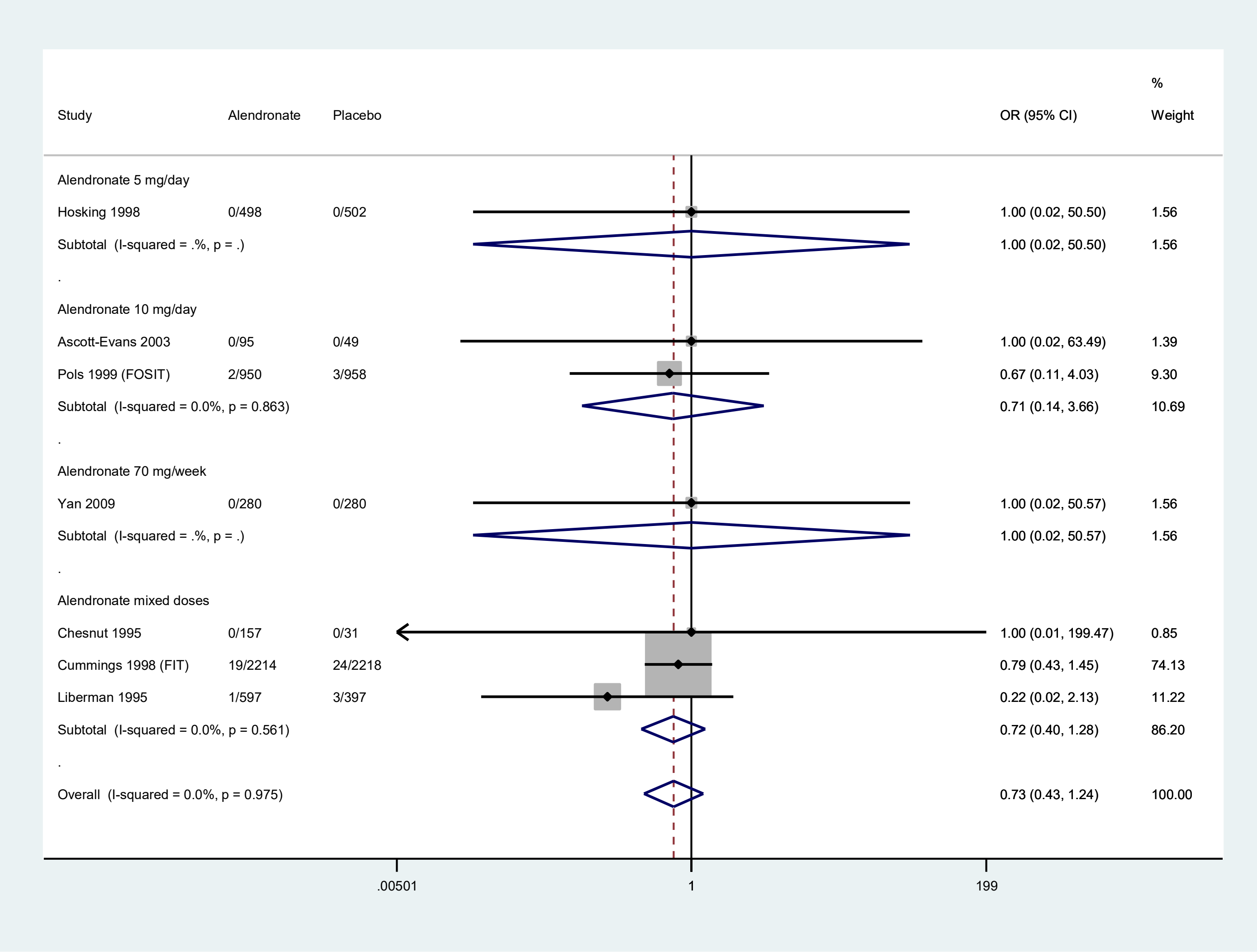

Length of treatment was 12 months in Ascott-Evans 2003, Pols 1999, Yan 2009, and Chesnut 1995 (20 and 40 mg/day doses); 24 months in Hosking 1998 and Chesnut 1995 (5 or 10 or 40 followed by 2.5 mg/day doses); 36 months in Liberman 1995 (5 or 10 or 20 followed by 5 mg/day); and 48 months in Cummings 1998 (5 mg/day for 24 months followed by 10 mg/day for 24 months). In all studies, follow-up was to the end of the treatment period, except the Chesnut 1995 20 and 40 mg/day doses, where follow-up extended 12 months beyond the end of treatment.

KQ3a: What are the benefits of pharmacologic treatments to prevent fragility fractures among adults ≥40 years?

Alendronate vs. placebo – clinical fragility fractures

Females

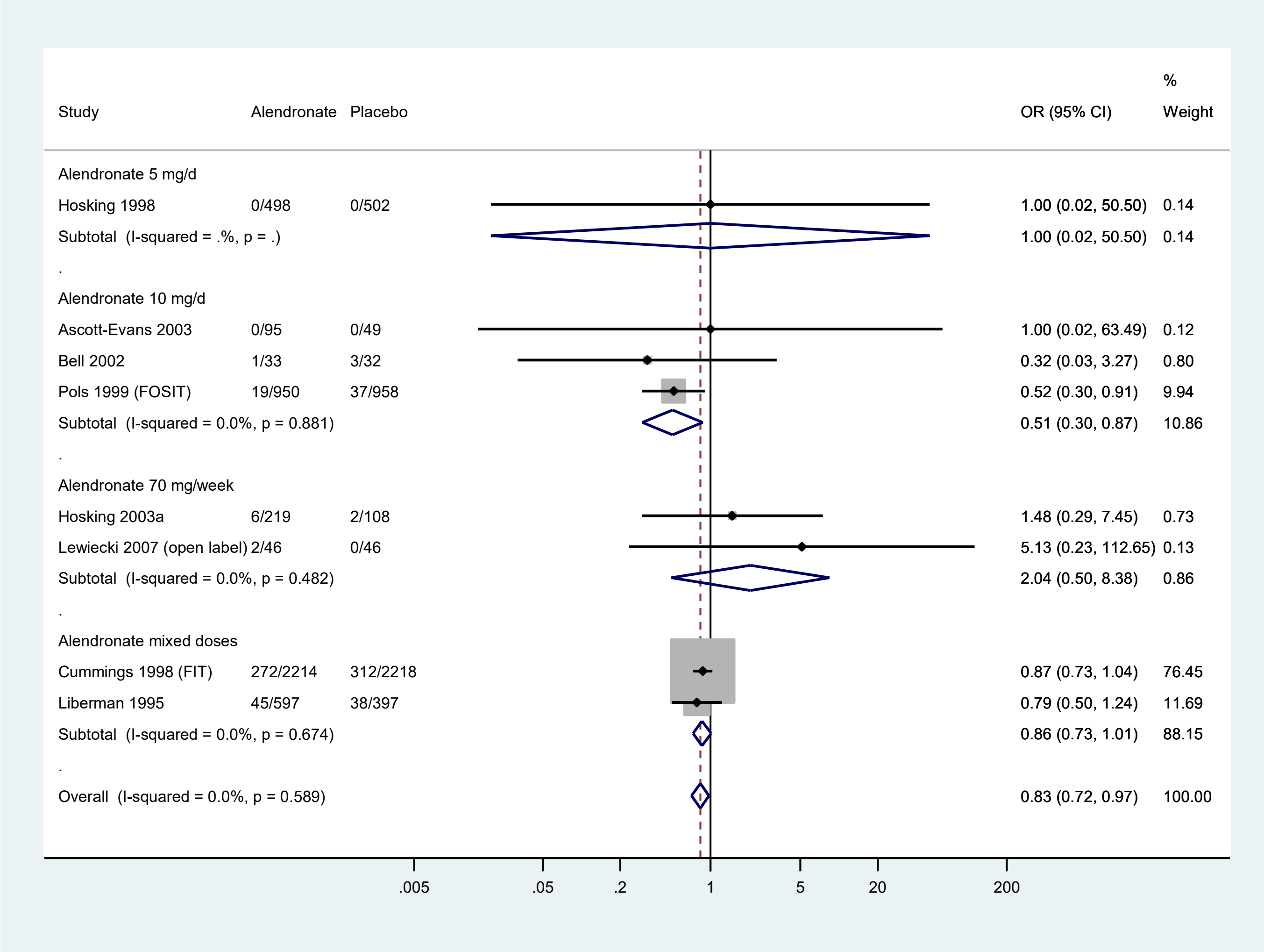

Length of treatment was 12 months in Ascott-Evans 2003, Hosking 2003, and Pols 1999; 24 months in Bell 2002, Hosking 1998, and Lewiecki 2007; 36 months in Liberman 1995 (5 or 10 or 20 followed by 5 mg/day); and 48 months in Cummings 1998 (5 mg/day for 24 months followed by 10 mg/day for 24 months). In all studies, follow-up was to the end of the treatment period.

KQ3a: What are the benefits of pharmacologic treatments to prevent fragility fractures among adults ≥40 years?

Summary of within-study subgroup data for clinical fragility fractures

Population	Within-study subgroup data
Femoral neck BMD T-score [37]	**≤-2.5:** 107/819 [13.1%] vs. 159/812 [19.6%]; HR 0.64 (0.50, 0.82)<br>**≤-2.0:** 199/1545 [12.9%] vs. 246/1522 [16.2%]; HR 0.78 (0.65, 0.94)<br>**>-2.5:** 73/699 [10.9%] vs. 66/696 [9.5%]; HR 1.08 (0.87, 1.35)
Age [46]	**≥75 years:** 88/1235 [7.9%] vs. 102/1236 [9.0%], HR 0.84 (0.63, 1.12)<br>**<75 years:** 150/2667 [5.9%] vs. 191/2670 [7.6%], HR 0.78 (0.63-0.96)
FRAX alone [45]	**Tertile 1 (3.48-18.75%):** 101/1082 [9.3%] vs. 116/1071 [10.8%], RD -0.41 (-1.1, 0.30)<br>**Tertile 2 (18.76-31.06%):** 128/1077 [11.9%] vs. 143/1076 [13.3%], RD -0.41 (-1.3, 0.44)<br>**Tertile 3 (31.07-76.23%):** 155/1077 [14.4%] vs. 182/1076 [16.9%], RD -0.85 (-1.9, 0.18)<br>Note: data also available by decile in Donaldson 2012
FRAX + BMD [45]	**Tertile 1 (4.75-22.06%):** 91/1083 [8.5%] vs. 104/1071 [9.7%], RD -0.34 (-1.0, 0.33)<br>**Tertile 2 (22.07-34.19%):** 129/1087 [11.9%] vs. 140/1066 [13.1%], RD -0.41 (-1.3, 0.43)<br>**Tertile 3 (34.2-85.36%):** 164/1066 [15.4%] vs. 197/1086 [18.1%], RD -0.91 (-2.0, 0.17)

Alendronate vs. placebo – clinical vertebral fractures

Females

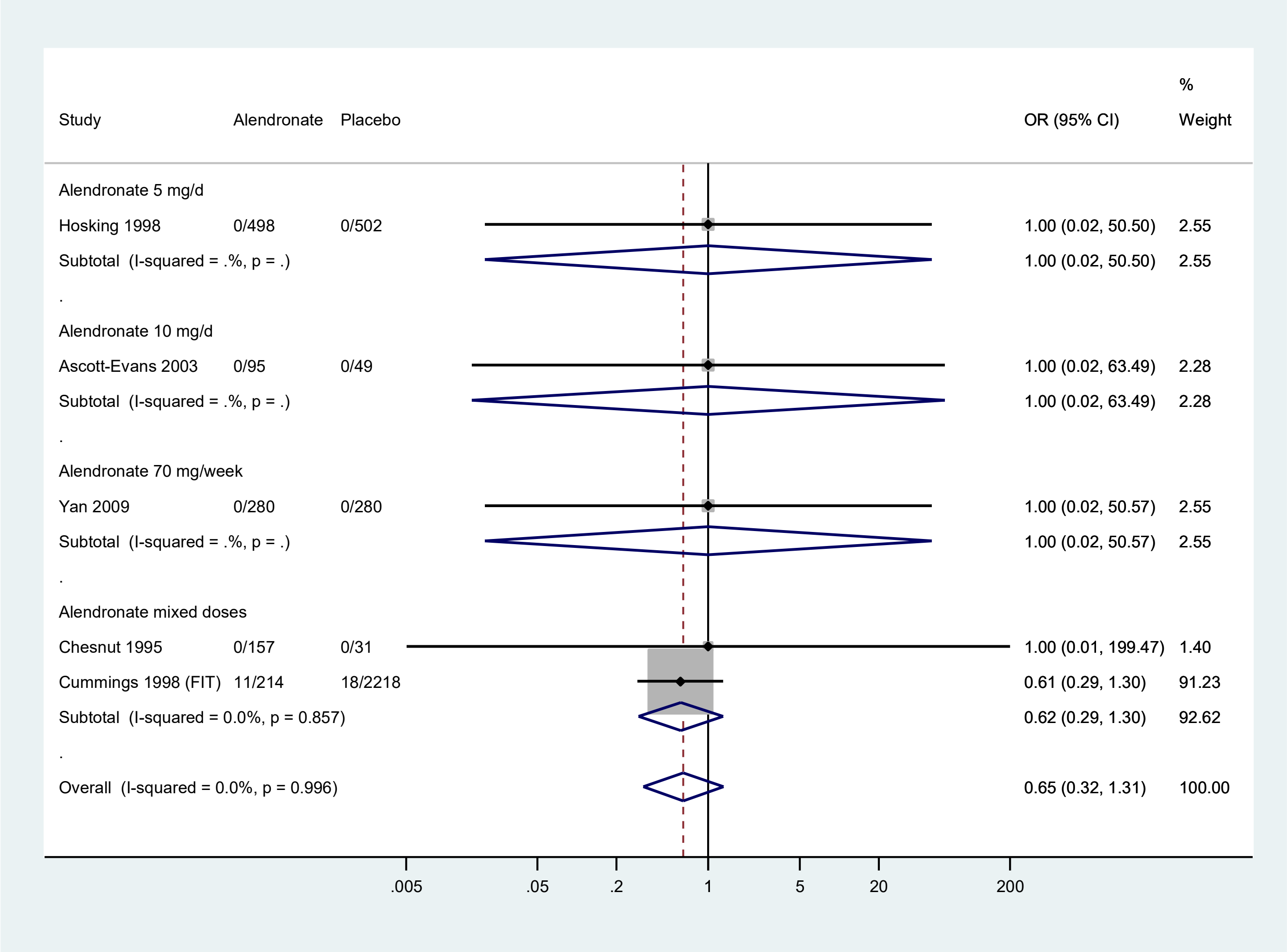

Length of treatment was 12 months in Ascott-Evans 2003, Yan 2009, and Chesnut 1995 (20 and 40 mg/day doses); 24 months in Hosking 1998 and Chesnut 1995 (5 or 10 or 40 followed by 2.5 mg/day doses); and 48 months in Cummings 1998 (5 mg/day

for 24 months followed by 10 mg/day for 24 months). In all studies, follow-up was to the end of the treatment period, except the Chesnut 1995 20 and 40 mg/day doses, where follow-up extended 12 months beyond the end of treatment.

Alendronate vs. placebo – all-cause mortality

Females

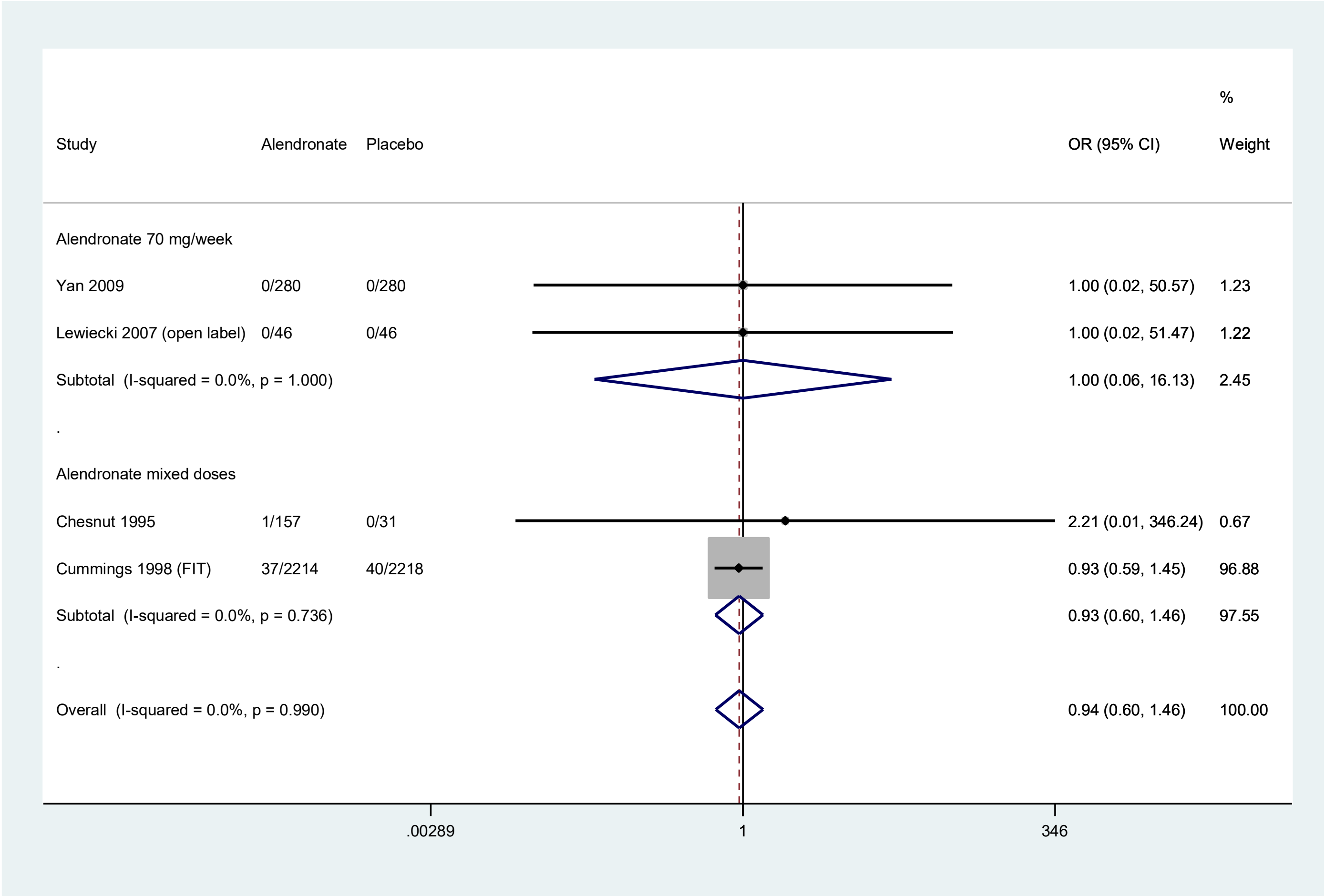

Length of treatment was 12 months in Chesnut 1995 (20 and 40 mg/day doses) and Yan 2009; 24 months Chesnut 1995 (5 or 10 or 40 followed by 2.5 mg/day doses) and Lewiecki 2007; and 48 months in Cummings 1998 (5 mg/day for 24 months followed by 10 mg/day for 24 months). In all studies, follow-up was to the end of the treatment period, except the Chesnut 1995 20 and 40 mg/day doses, where follow-up extended 12 months beyond the end of treatment.

KQ3a: What are the benefits of pharmacologic treatments to prevent fragility fractures among adults ≥40 years?

2. Risedronate vs. placebo

2.1 GRADE Summary of Findings

Outcome & Study approach	Studies; sample size	Follow-up (y)	Assumed population risk*	Absolute effects (95% CI)	Certainty	What happens?
**Risedronate vs. placebo (postmenopausal females)**						
Hip fractures<br>Intention to treat	4 RCT; 9,672 [50-53]	1 to 3	Study data:<br>30 in 1000	7.9 fewer in 1000<br>(13.0 fewer to 1.5 fewer)	LOW[a]	May reduce
			General F ≥65 y:<br>20 in 1000	5.3 fewer in 1000<br>(8.7 fewer to 1.0 fewer)		
Clinical fragility fractures<br>Intention to treat or exposed to ≥1 dose	7 RCT; 10,572 [44, 50-55]	1 to 3	Study data:<br>48 in 1000	7.8 fewer in 1000<br>(12.5 fewer to 2.3 fewer)	LOW[b]	May reduce
			General F ≥65 y:<br>202 in 1000	28.4 fewer in 1000<br>(46.0 fewer to 8.1 fewer)		
Clinical vertebral fractures	The evidence from 2 RCTs (n=230) [53, 54] is very uncertain.				VERY LOW[c]	Very uncertain
All-cause mortality	The evidence from 1 RCTs (n=170) [53] is very uncertain.				VERY LOW[d]	Very uncertain

CI=confidence interval; RCT=randomized controlled trial; y=years

* The effects without screening for the general risk population are estimated from PRIOR et al. [49], based on 10 year follow-up. Data for the general population <65 years is not included in the summary table (available on request).

Explanations:

[a] ***Serious concern about risk of bias***: the one adequately powered trial (McClung 2001) is at high risk of bias due to a high rate of attrition (>30%). ***Some concern about inconsistency:*** lack of evidence of consistency because the analysis hinges primarily on the one adequately powered trial (McClung 2001). ***Some concern about indirectness:*** in the one adequately powered trial, the rate of prior fracture is unknown; 41% of those with known vertebral fracture status (n=6876, 74% of participants) had a prevalent vertebral fracture at baseline. This trial combined 2.5 mg/day and 5 mg/day doses in their analysis; the 2.5 mg/day is not an approved dosage in Canada (lower than the approved dose but still showing benefit; did not rate down). ***No serious concern about imprecision:*** the sample size is large. ***No serious concern for other considerations:*** no other concerns that would further reduce our confidence in the findings.

[b] ***Serious concern about risk of bias***: the largest trial (McClung 2001) is at high risk of bias due to a high rate of attrition (>30%). ***Some concern about inconsistency:*** limited evidence of consistency because the analysis hinges primarily on the one large trial (McClung 2001); however, most point estimates are in the same direction. ***Some concerns about indirectness:*** in the one adequately powered trial, the rate of prior fracture is unknown; 41% of those with known vertebral fracture status (n=6876, 74% of participants) had a prevalent vertebral fracture at baseline. This trial combined 2.5 mg/day and 5 mg/day doses in their analysis; the 2.5 mg/day is not an approved dosage in Canada (lower than the approved dose but still showing benefit; did not rate down). In Hosking 2003 the outcome was self-reported and may include non-clinical vertebral fractures, but the findings were robust to sensitivity analysis removing this trial (did not rate down). ***No serious concern about imprecision:*** the sample size is adequate and confidence interval is precise for benefit of risedronate. ***No serious concern for other considerations:*** no other concerns that would further reduce our confidence in the findings.

[c] ***Serious concern about risk of bias***: Li 2005 is at high risk of bias due to imbalance in rates of attrition between groups (14% in the placebo group and 7% in the risedronate group). The findings could be biased because several of the trials of risedronate did not report on this outcome specifically. ***No serious concern about inconsistency. Some concern about indirectness:*** in both of the included studies the rate of prior fracture is not known. ***Very serious concern about imprecision:*** the number of events does not meet the optimal information size (n<300), and the sample size is small. The confidence interval is very wide and includes the potential for both important benefit and harm. ***No serious concern for other considerations:*** no other concerns that would further reduce our confidence in the findings.

[d] ***Serious concern about risk of bias***: in the one included trial (Välimäki 2007), it is unclear how the outcome was collected (may not have been collected systematically). Selective reporting strongly suspected, since only 1 of the 6 trials of alendronate (17%) reported on this outcome, and appears to be collected passively. ***Some concern about inconsistency:*** lack of evidence of consistency because there is only one trial reporting on this outcome. ***No major concerns about indirectness:*** the one included trial is well aligned with the review question. ***Serious concern about imprecision:*** the number of events does not

meet the optimal information size (n<300), and the sample size is small. The confidence interval is very wide and includes the potential for both important benefit and harm. ***No serious concern for other considerations:*** no other concerns that would further reduce our confidence in the findings.

2.2 Contributing data

Risedronate vs. placebo – hip fractures

Females

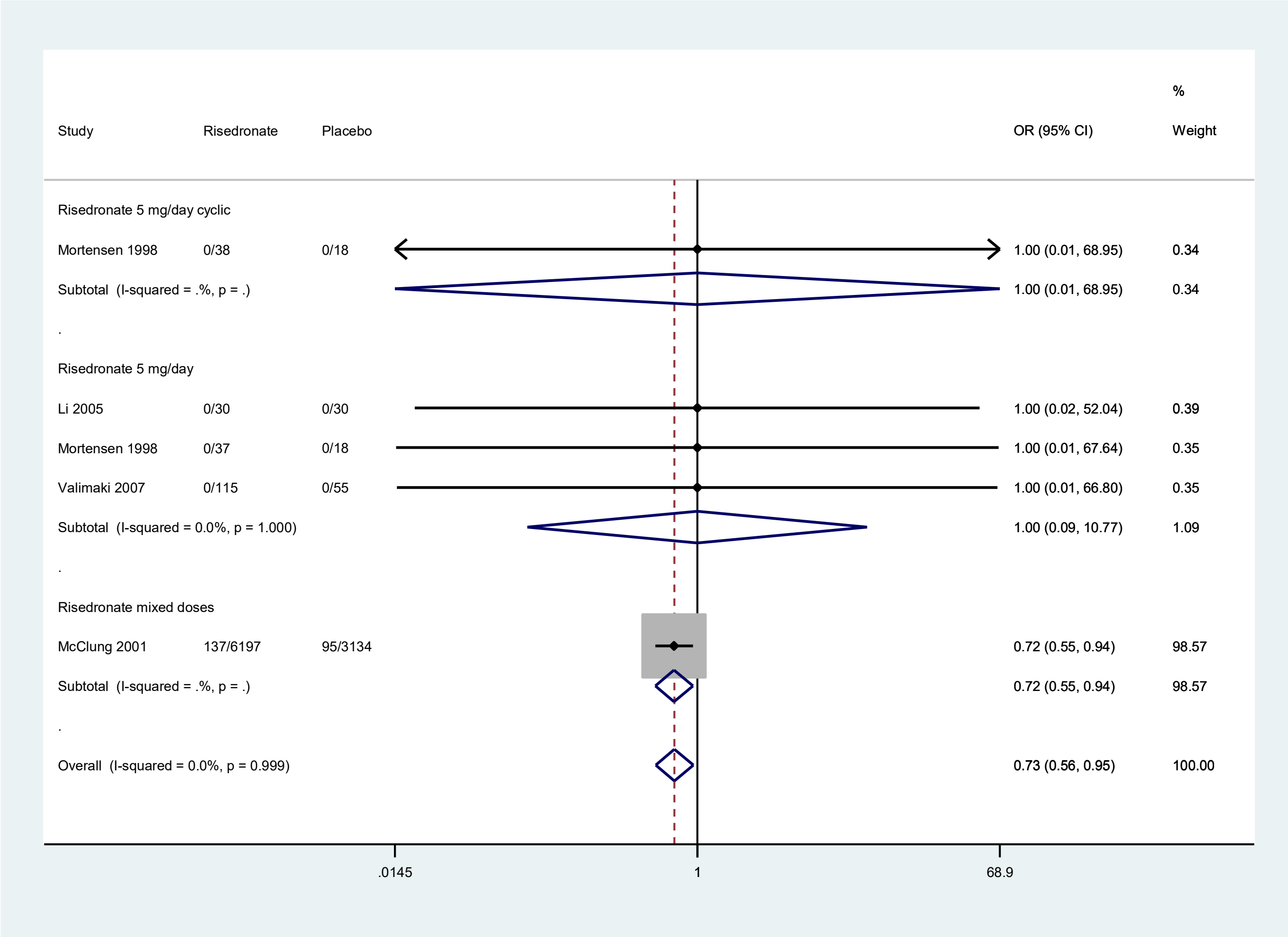

Length of treatment was 12 months in Li 2005; 24 months in Mortensen 1998 (cyclic treatment was 2 weeks on, 2 weeks off) and Valimäiki 2007; 36 months in McClung 2001 (2.5 or 5 mg/day). In all studies, follow-up was to the end of the treatment period.

KQ3a: What are the benefits of pharmacologic treatments to prevent fragility fractures among adults ≥ 40 years?

Risedronate vs. placebo – clinical fragility fractures

Females

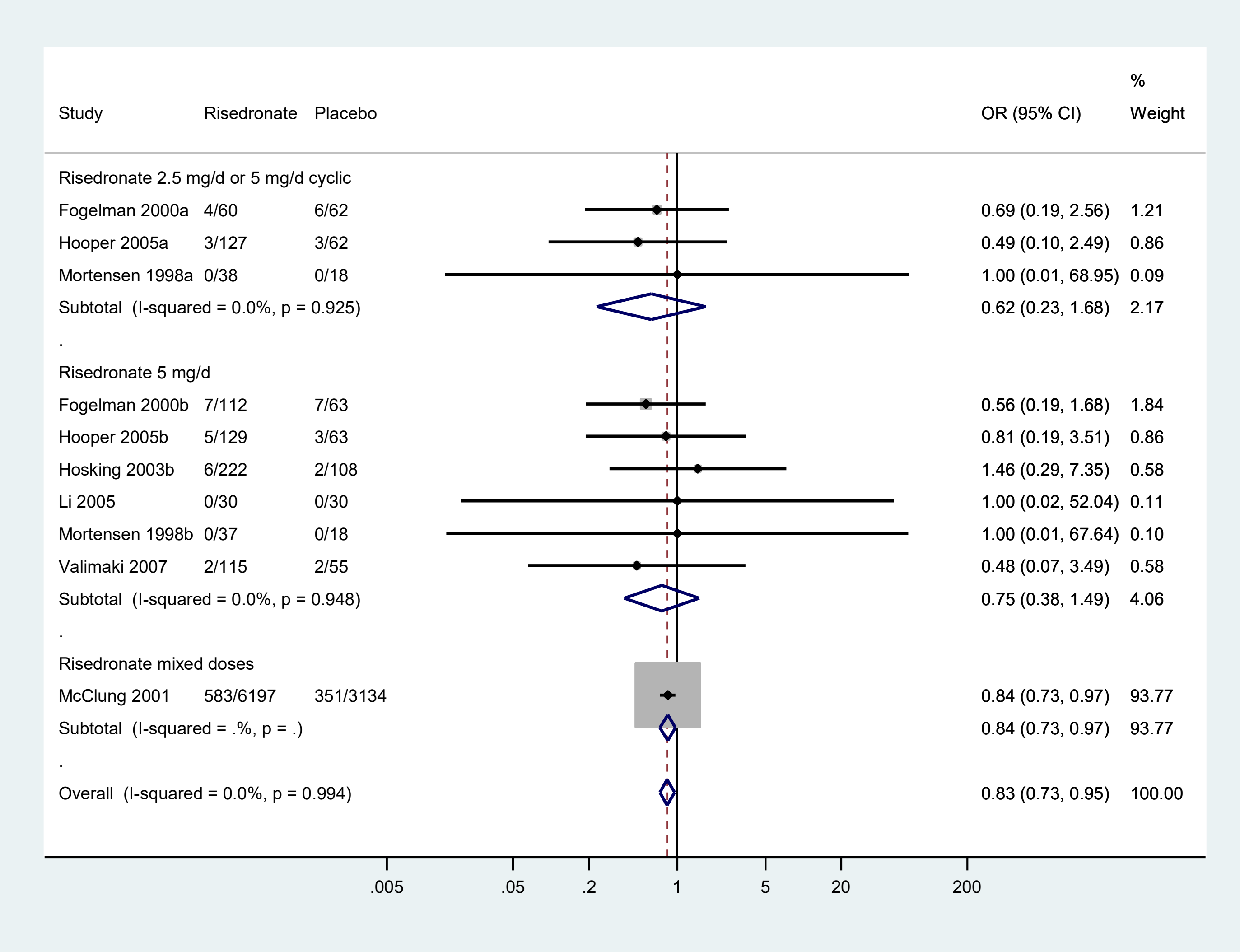

Length of treatment was 12 months in Hosking 2003 and Li 2005; 24 months in Fogelman 2000 (2.5 mg/day or 5 mg/day), Hooper 2005 (2.5 mg/day or 5 mg/day) Mortensen 1998 (cyclic treatment was 2 weeks on, 2 weeks off) and Valimäiki 2007; 36 months in McClung 2001 (2.5 or 5 mg/day). In all studies, follow-up was to the end of the treatment period except Mortensen 1998, which extended one year beyond the end of treatment.

Summary of within-study subgroup data for clinical fragility fractures

Population	Within-study subgroup data
Risk factors/BMD [51]	**70-79 years with osteoporosis (BMD T-score <-4 or -3 with at least 1 risk factor):** 304/3624 [8.4%] vs. 195/1821 [10.7%]; RR 0.8 (0.7, 1.0)<br>**70-79 years with osteoporosis + vertebral fracture at baseline:** 116/1128 [10.3%] vs. 141/875 [16.1%]; RR 0.7 (0.5, 0.9)<br>**≥80 years with ≥1 clinical risk factors:** 278/2573 [10.8%] vs. 156/1313 [11.9%]; p=0.43 (relative effect not provided)

KQ3a: What are the benefits of pharmacologic treatments to prevent fragility fractures among adults ≥ 40 years?

Risedronate vs. placebo – clinical vertebral fractures

Females

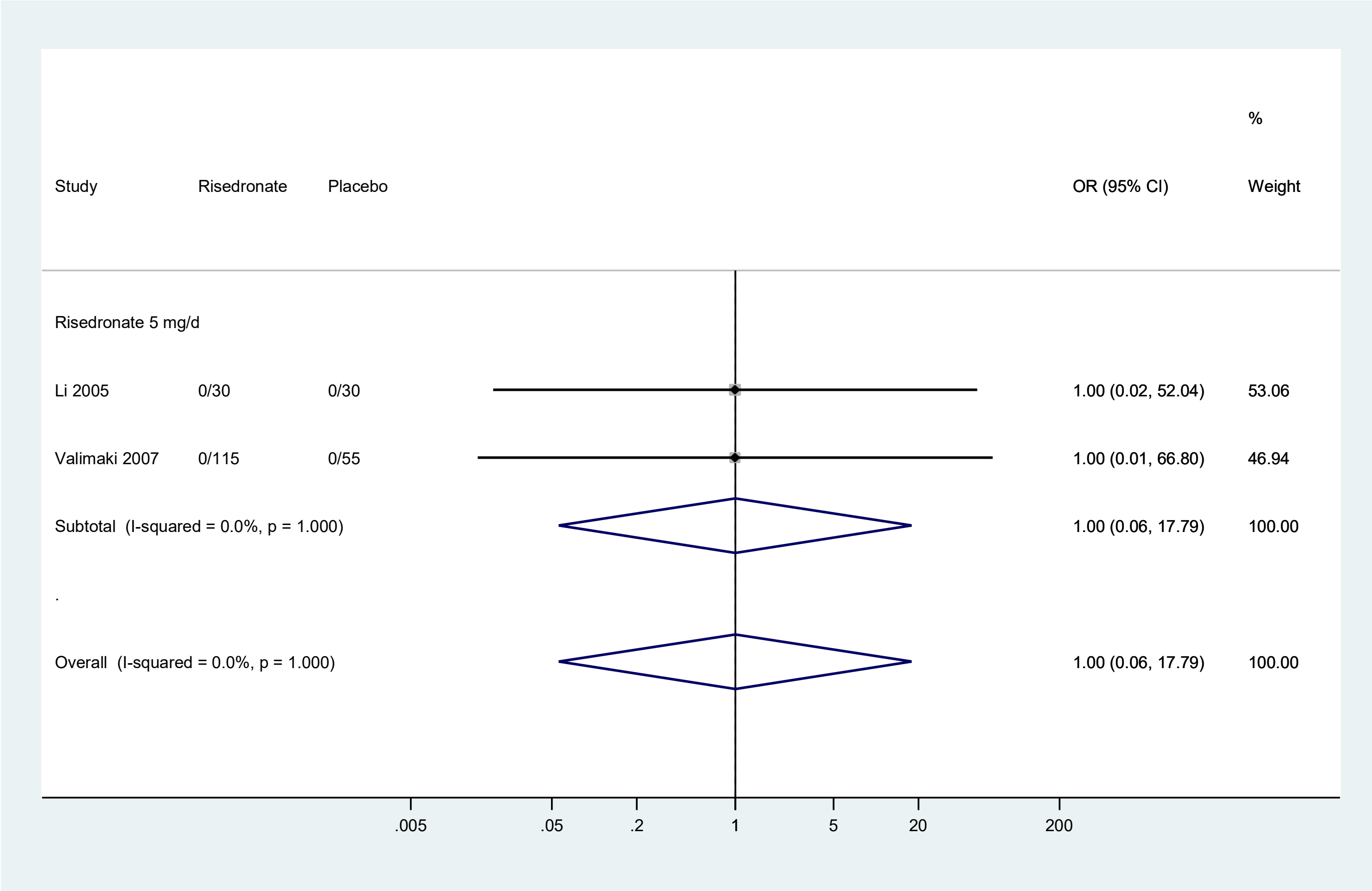

Length of follow up was 12 months in Li 2005 and 24 months in Välimäiki 2007. Follow-up was to the end of treatment.

KQ3a: What are the benefits of pharmacologic treatments to prevent fragility fractures among adults ≥ 40 years?

Risedronate vs. placebo – all-cause mortality

Females

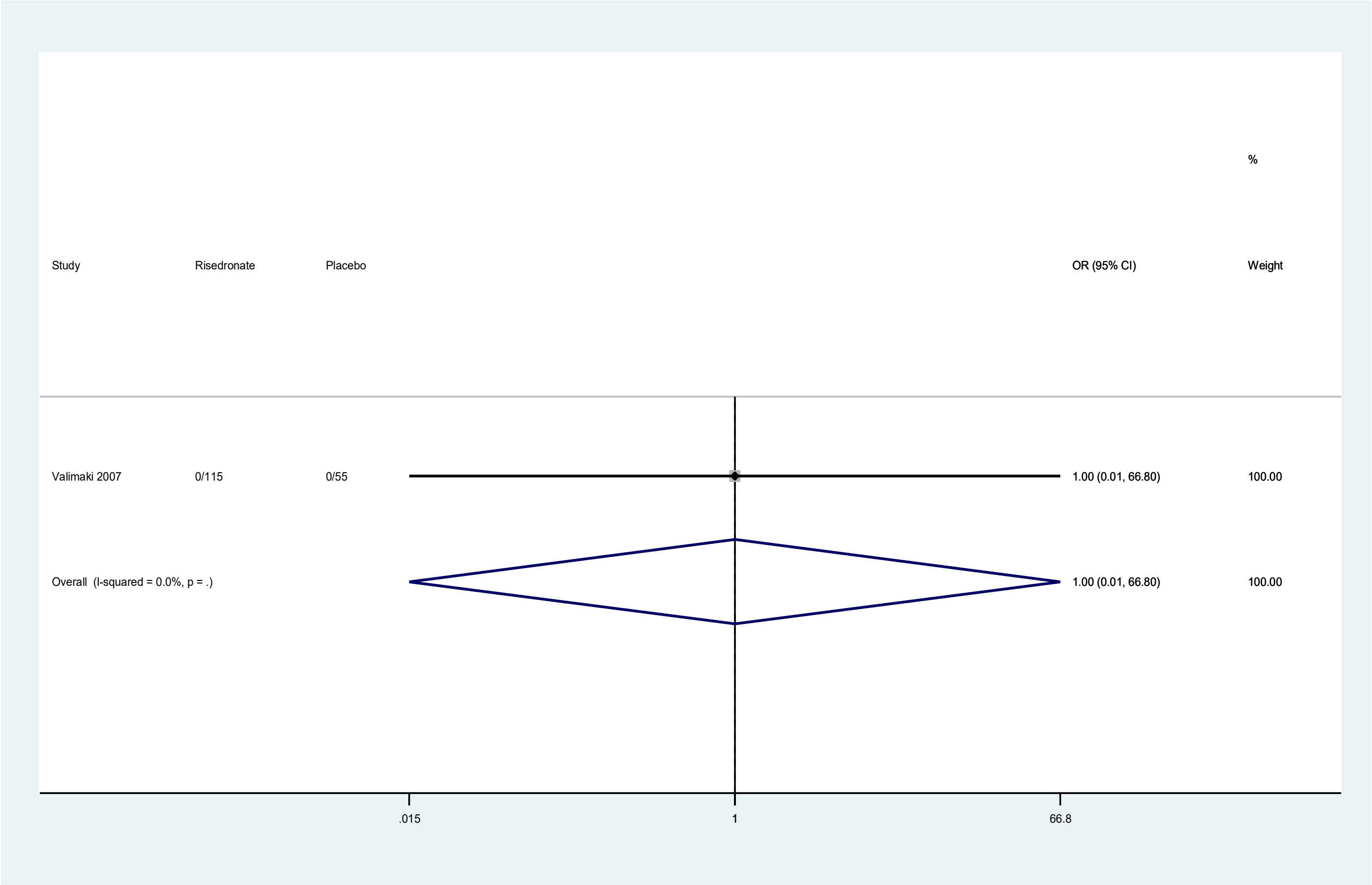

Length of treatment was 24 months. Follow-up was to the end of treatment.

KQ3a: What are the benefits of pharmacologic treatments to prevent fragility fractures among adults ≥40 years?

3. Zoledronic acid vs. placebo
3.1 GRADE Summary of Findings

Outcome & Study approach	Studies; sample size	Follow-up (y)	Assumed population risk*	Absolute effects (95% CI)	Certainty	What happens?
**Zoledronic acid vs. placebo (post-menopausal females)**						
Hip fractures<br>Intention to treat	3 RCT; 2,200 [56-59]	1 to 6	Study data:<br>12 in 1000	3.7 fewer in 1000<br>(8.5 fewer to 7.4 more)	LOW[a]	May not reduce
			General F ≥65 y:<br>20 in 1000	6.1 fewer in 1000<br>(14.1 fewer to 12.2 more)		
Clinical fragility fractures<br>Intention to treat	5 RCT; 3,218 [56-61]	1 to 6	Study data:<br>58 in 1000	20.1 fewer in 1000<br>(27.6 fewer to 9.9 fewer)	MODERATE[b]	Probably reduces
			General F ≥65 y:<br>202 in 1000	62.6 fewer in 1000<br>(87.7 fewer to 30.1 fewer)		
Clinical vertebral fractures<br>Intention to treat	4 RCT; 2,367 [56-59, 61]	1 to 6	Study data:<br>34 in 1000	18.7 fewer in 1000<br>(25.6 fewer to 6.6 fewer)	LOW[c]	May reduce
			General F ≥65 y:<br>27 in 1000	14.9 fewer in 1000<br>(20.4 fewer to 5.3 fewer)		
All-cause mortality	The evidence from 3 RCTs (n=2,656) [56, 58, 60, 62] is very uncertain.				VERY LOW[d]	Very uncertain
**Zoledronic acid vs. placebo (males)**						
Hip fractures<br>Intention to treat	1 RCT; 1,199 [63]	2	Study data:<br>2 in 1000	2.2 more in 1000<br>(1.6 fewer to 42.0 more)	LOW[e]	May not reduce
			General M ≥65 y:<br>16 in 1000	16.7 more in 1000<br>(12.9 fewer to 256.0 more)		
Clinical fragility fractures<br>Intention to treat	1 RCT; 1,199 [63]	2	Study data:<br>18 in 1000	7.7 fewer in 1000<br>(14.2 fewer to 9.5 more)	LOW[f]	May not reduce
			General M ≥65 y:<br>105 in 1000	42.3 fewer in 1000<br>(81.0 fewer to 48.0 more)		
Clinical vertebral fractures	No study reported on this outcome.					
All-cause mortality	The evidence from 1 RCT (n=1,199) [63] is very uncertain				VERY LOW[g]	Very uncertain

CI=confidence interval; RCT=randomized controlled trial; y=years

* The effects without screening for the general risk population are estimated from PRIOR et al. [49], based on 10 year follow-up. Data for the general population <65 years is not included in the summary table (available on request).

Explanations:

[a] ***No serious concern about risk of bias***: no serious risk of bias concerns in the large trial (Reid 2018). ***Some concern about inconsistency:*** lack of evidence of consistency because the analysis hinges primarily on the one adequately powered trial (Reid 2018). ***Some concern about indirectness:*** the one adequately powered trial (Reid 2018) used a 5mg/18 months dose which is not an approved dosage in Canada (lower than the approved dose and showing no difference). ***Serious concern about imprecision:*** the number of events does not meet the optimal information size (<300), and sample size is <4,000. ***No serious concern for other considerations:*** no other concerns that would further reduce our confidence in the findings.

[b] ***No serious concern about risk of bias***: no serious risk of bias concerns in the large trial (Reid 2018). ***Some concern about inconsistency:*** lack of evidence of consistency because the analysis hinges primarily on the one adequately powered trial (Reid 2018). ***No serious concern about indirectness:*** the one adequately powered trial (Reid 2018) used a 5mg/18 months dose which is not an approved dosage in Canada (lower than the approved dose) but the pooled estimate still shows benefit (did not rate down). In McClung 2009 the outcome is self-reported and could include non-clinical vertebral fractures, and in Reid 2018 clinical vertebral and nonvertebral fractures were added to determine the number of events. The analysis was robust to sensitivity analysis removing McClung 2009 and using nonvertebral fractures for Reid 2018 (did not rate down). ***No serious concern about imprecision:*** the sample size is adequate and confidence interval is precise for benefit of zoledronic acid. ***No serious concern for other considerations:*** no other concerns that would further reduce our confidence in the findings.

[c] ***Some concern about risk of bias***: no serious risk of bias concerns in the one large trial (Reid 2018). The findings could be biased because several of the trials of zoledronic acid did not report on this outcome specifically. ***Some concern about inconsistency:*** lack of evidence of consistency because the analysis hinges primarily on the one large trial (Reid 2018). ***No serious concern about indirectness:*** the one adequately powered trial (Reid 2018) used a 5mg/18 months dose which is not an

approved dosage in Canada (lower than the approved dose) but the pooled estimate still shows benefit (did not rate down). ***Serious concern about imprecision:*** the number of events does not meet the optimal information size (n<300). ***No serious concern for other considerations:*** no other concerns that would further reduce our confidence in the findings.

[d] ***Serious concern about risk of bias***: selective reporting suspected, since only 3 of the 5 studies of zoledronic acid (60%) in females reported on this outcome, and appears to be collected passively in all but one (Reid 2018) of these trials. ***Some concern about inconsistency:*** some concern about lack of evidence of consistency because the analysis hinges primarily on the one larger trial (Reid 2018). ***Serious concerns about indirectness:*** The main contributing study (Reid 2018) provided a dose of zoledronic acid (5mg/18 months) that is not approved for use in Canada (lower than the approved dose and showing no difference). ***Serious concern about imprecision:*** the number of events does not meet the optimal information size (n<300) and the sample size is <4,000. ***No serious concern for other considerations:*** no other concerns that would further reduce our confidence in the findings.

[e] ***No serious concern about risk of bias:*** no serious risk of bias concerns in the on included trial. ***Some concern about inconsistency:*** no evidence of consistency because there is only one included trial. ***Serious concern about imprecision:*** the number of events does not meet the optimal information size. ***No serious concern for indirectness, or other considerations:*** no other concerns that would further reduce our confidence in the findings.

[f] ***No serious concern about risk of bias:*** no serious risk of bias concerns in the on included trial. ***Some concern about inconsistency:*** no evidence of consistency because there is only one included trial. ***Serious concern about imprecision:*** the number of events does not meet the optimal information size. ***No serious concern for indirectness, or other considerations:*** no other concerns that would further reduce our confidence in the findings.

[g] ***Serious concern about risk of bias***: it is unclear how the outcome was collected (may not have been collected systematically), appears to be collected passively. ***Some concern about inconsistency:*** some concern about lack of evidence of consistency because there is only one trial in the analysis. ***Serious concern about imprecision:*** the number of events does not meet the optimal information size (n<300) and the sample size is <4,000. ***No serious concern for indirectness or other considerations:*** no other concerns that would further reduce our confidence in the findings.

3.2 Contributing data
Zoledronic acid vs. placebo – hip fractures

Females

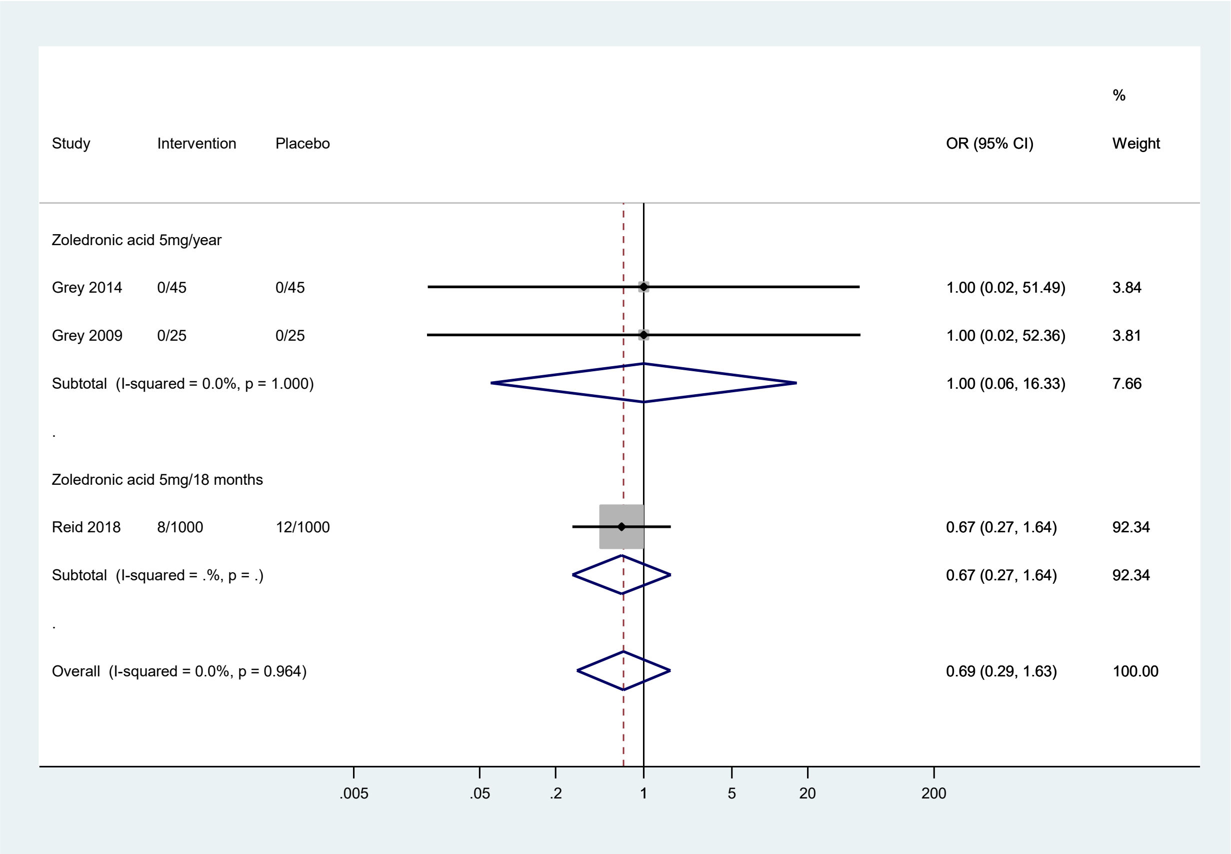

Length of follow-up was to the end of the treatment period for all studies, corresponding to 12 months (1 infusion) for Grey 2009 and Grey 2014; 72 months (4 infusions) for Reid 2018.

KQ3a: What are the benefits of pharmacologic treatments to prevent fragility fractures among adults ≥ 40 years?

Males

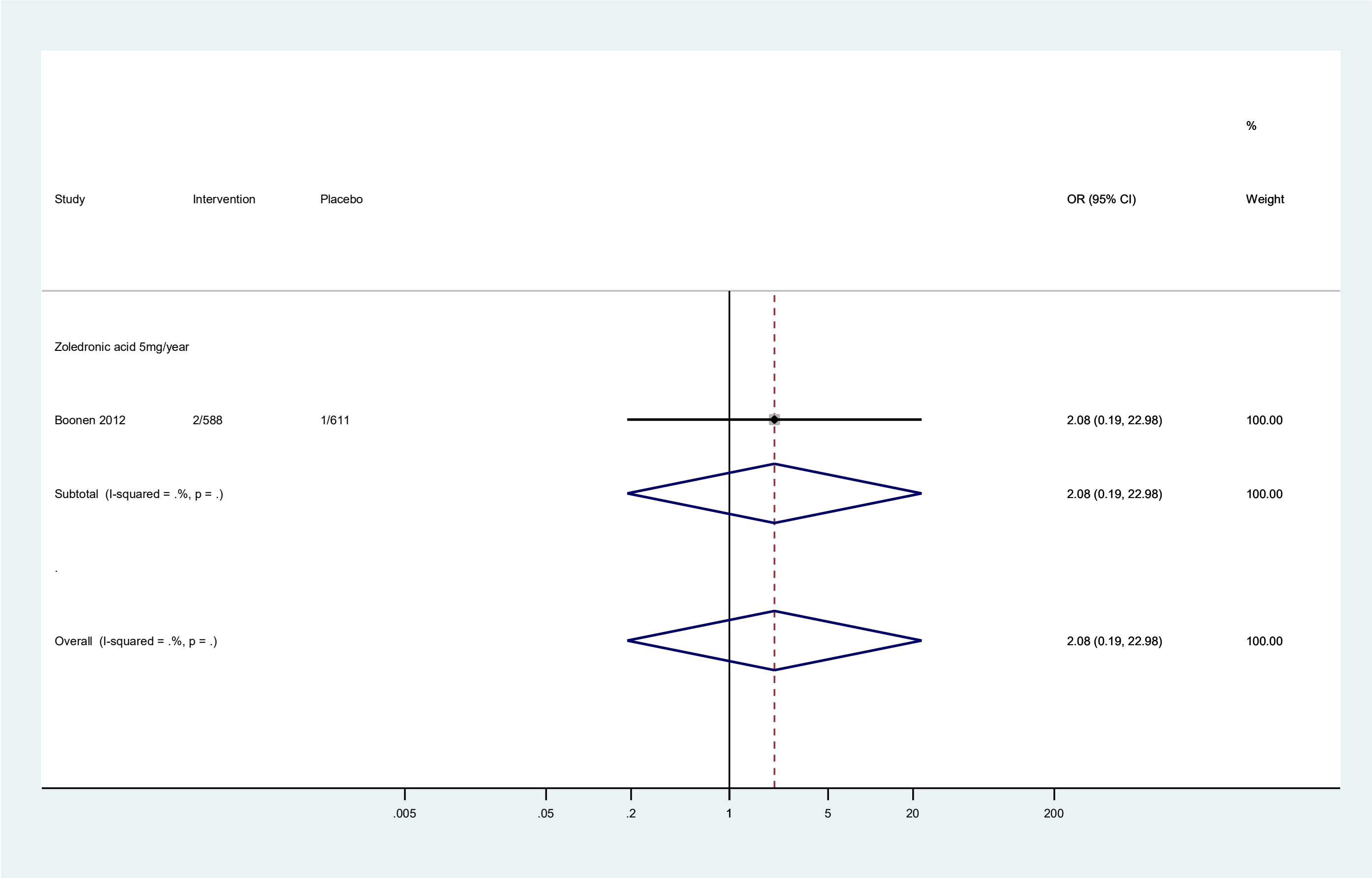

Length of follow-up was to the end of the treatment, corresponding to 24 months (2 infusions).

KQ3a: What are the benefits of pharmacologic treatments to prevent fragility fractures among adults ≥40 years?

Zoledronic acid vs. placebo – clinical fragility fractures

Females

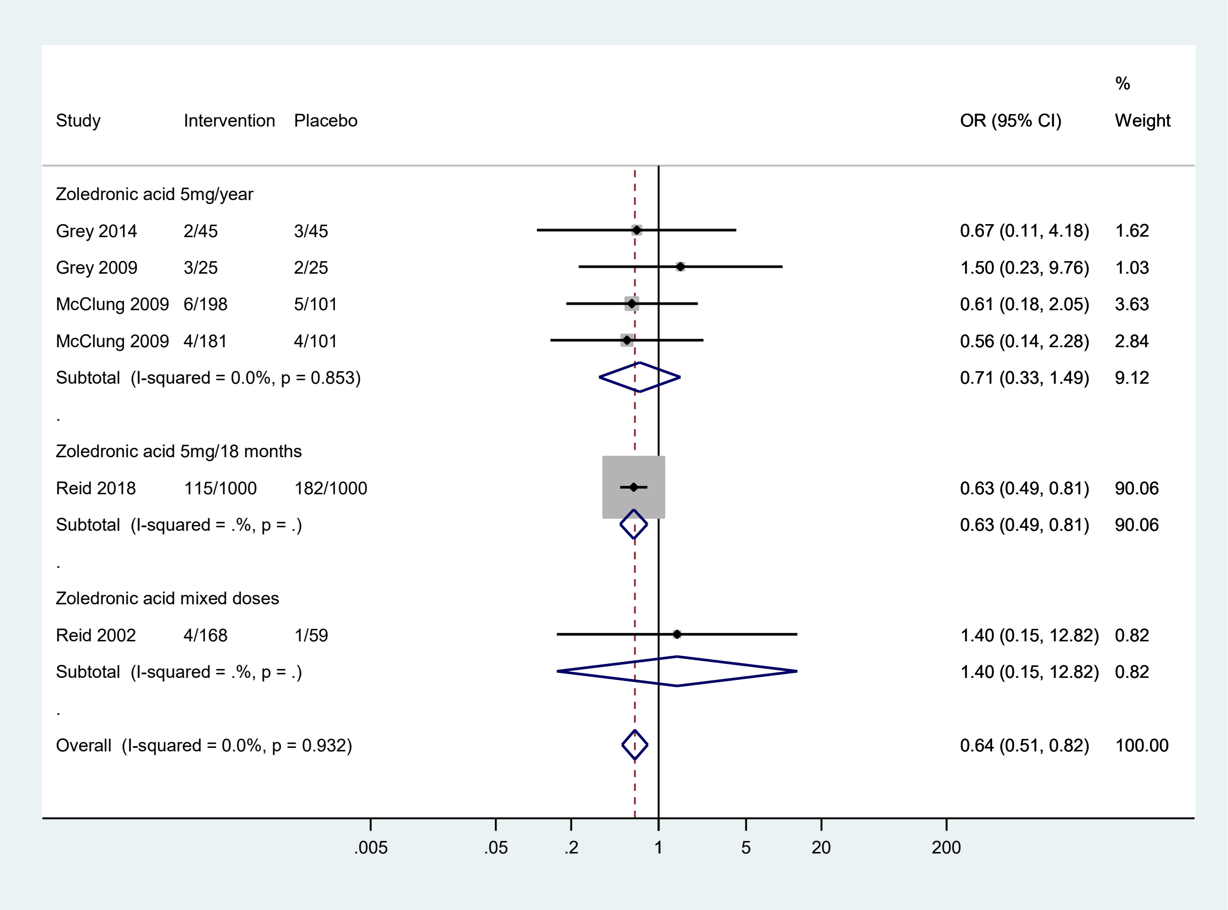

Length of follow-up was to the end of the treatment period for all studies, corresponding to 12 months for Reid 2002 (4 mg/year in 1, 2, or 4 infusions); 12 months (1 infusion) for Grey 2009 and Grey 2014; 72 months (4 infusions) for Reid 2018.

KQ3a: What are the benefits of pharmacologic treatments to prevent fragility fractures among adults ≥ 40 years?

Females – sensitivity analysis

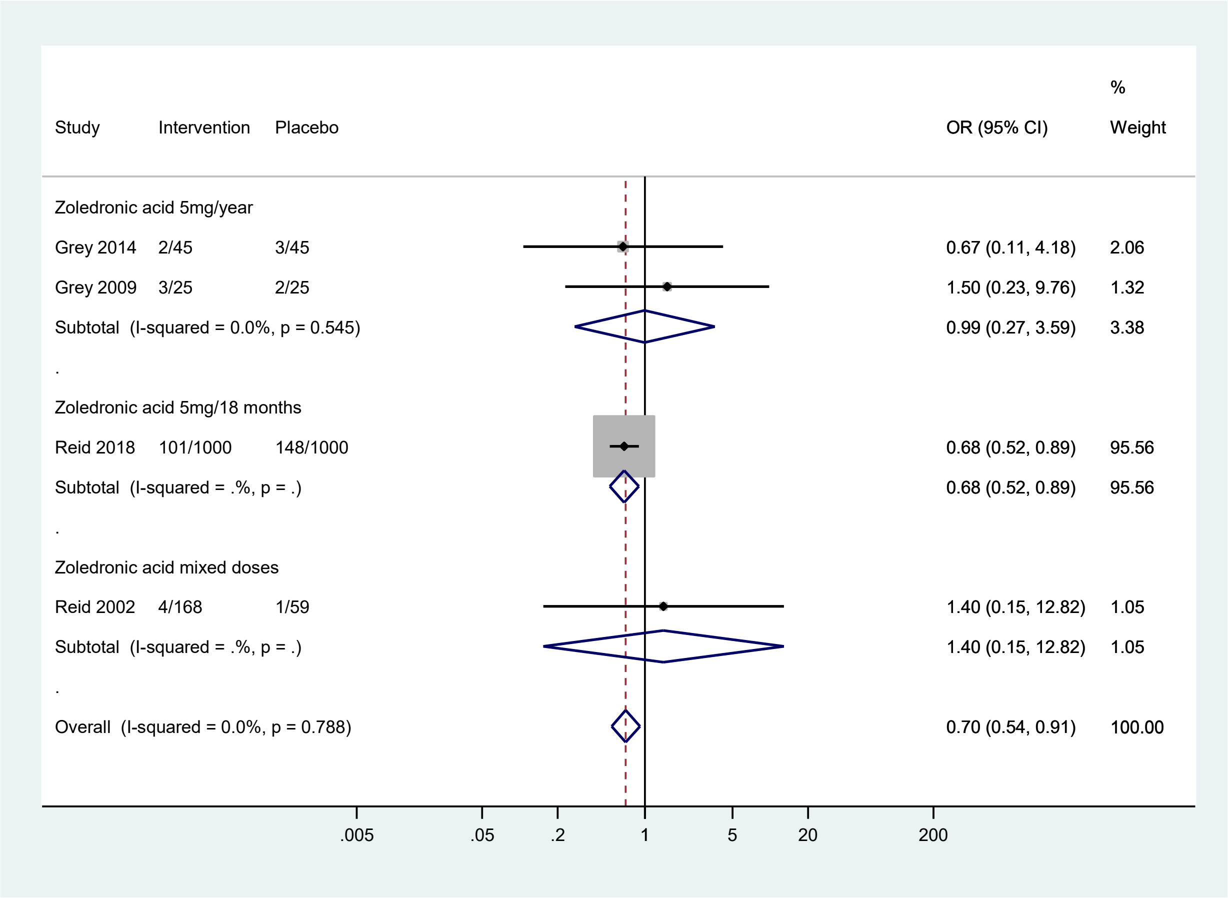

KQ3a: What are the benefits of pharmacologic treatments to prevent fragility fractures among adults ≥ 40 years?

Males

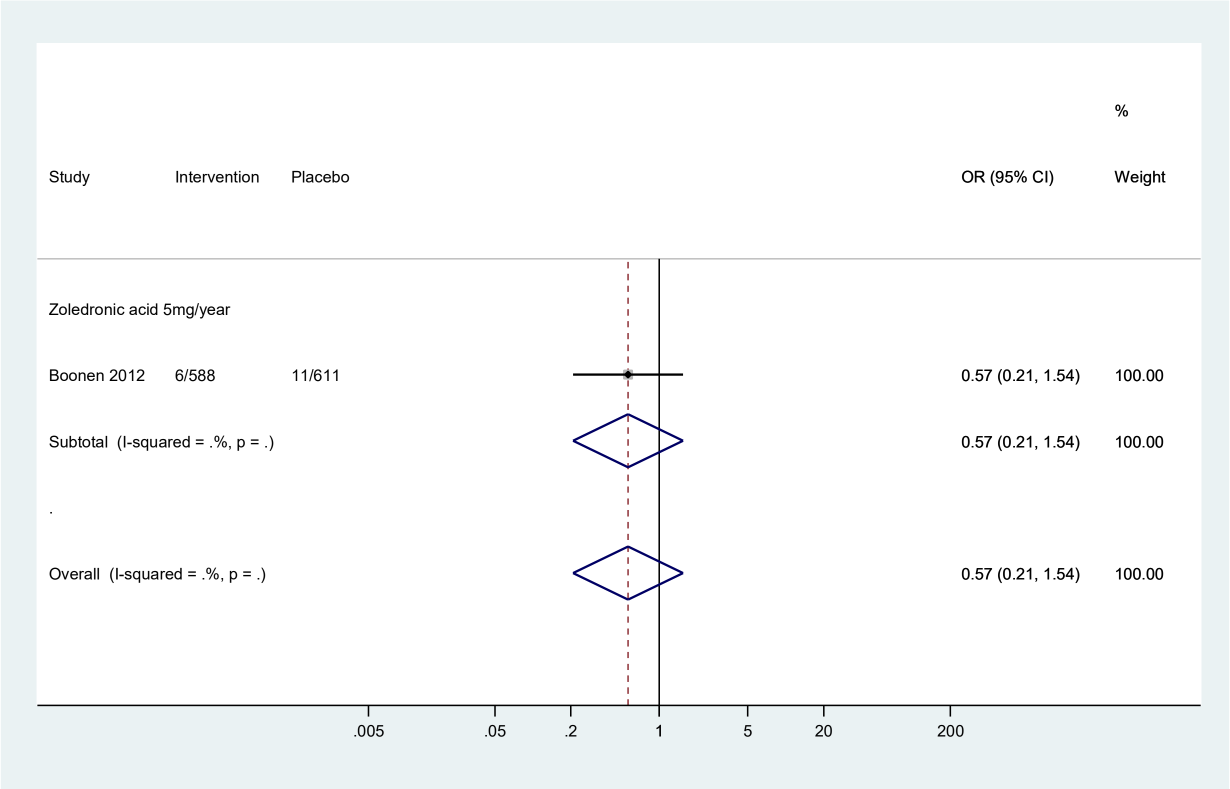

Length of follow-up was to the end of the treatment period, corresponding to 24 months (2 infusions).

KQ3a: What are the benefits of pharmacologic treatments to prevent fragility fractures among adults ≥ 40 years?

Zoledronic acid vs. placebo – clinical vertebral fractures

Females

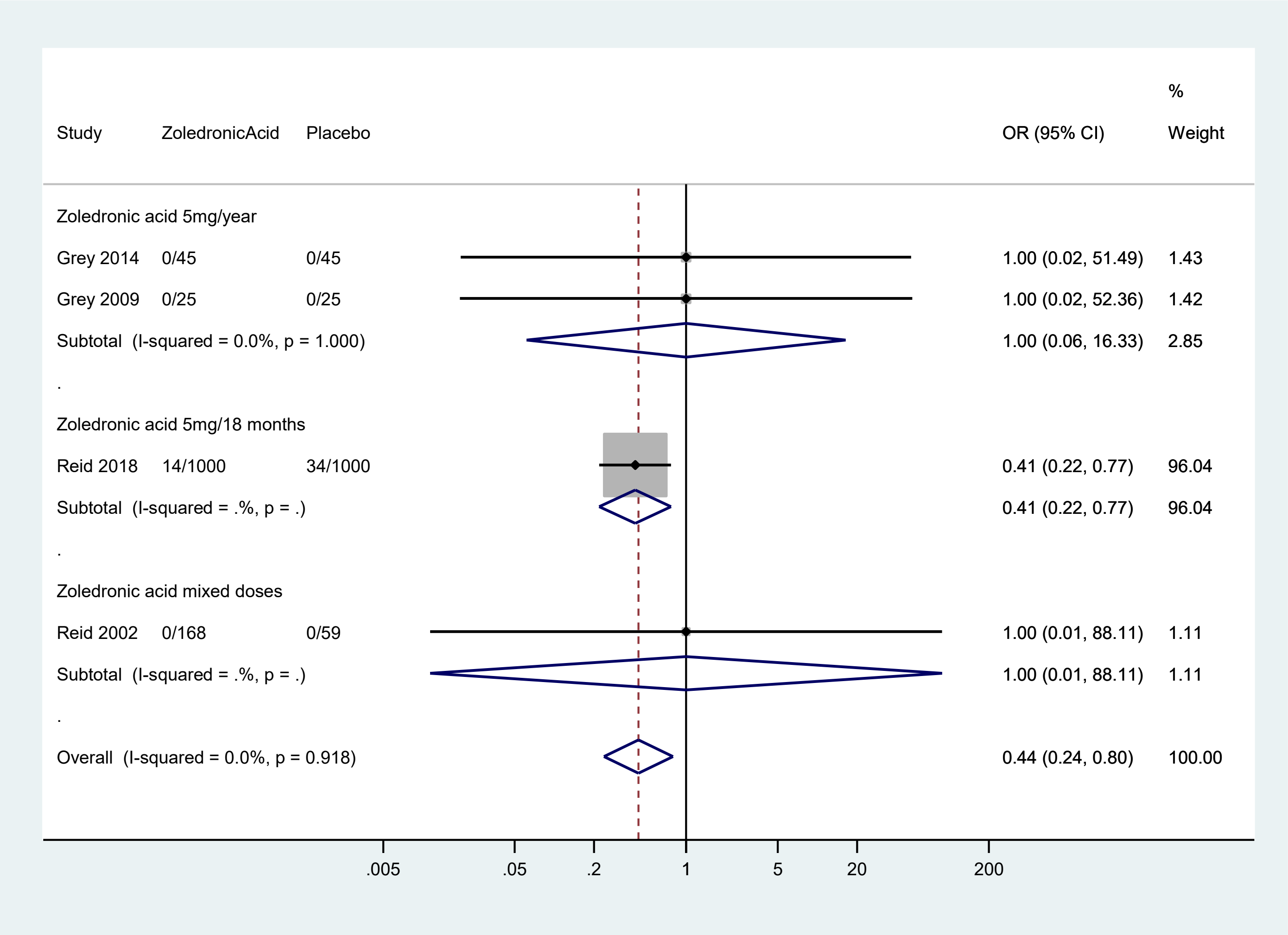

Length of follow-up was to the end of the treatment period for all studies, corresponding to 12 months for Reid 2002 (4 mg/year in 1, 2, or 4 infusions); 12 months (1 infusion) for Grey 2009 and Grey 2014; 72 months (4 infusions) for Reid 2018.

KQ3a: What are the benefits of pharmacologic treatments to prevent fragility fractures among adults ≥ 40 years?

Zoledronic acid vs. placebo – all-cause mortality

Females

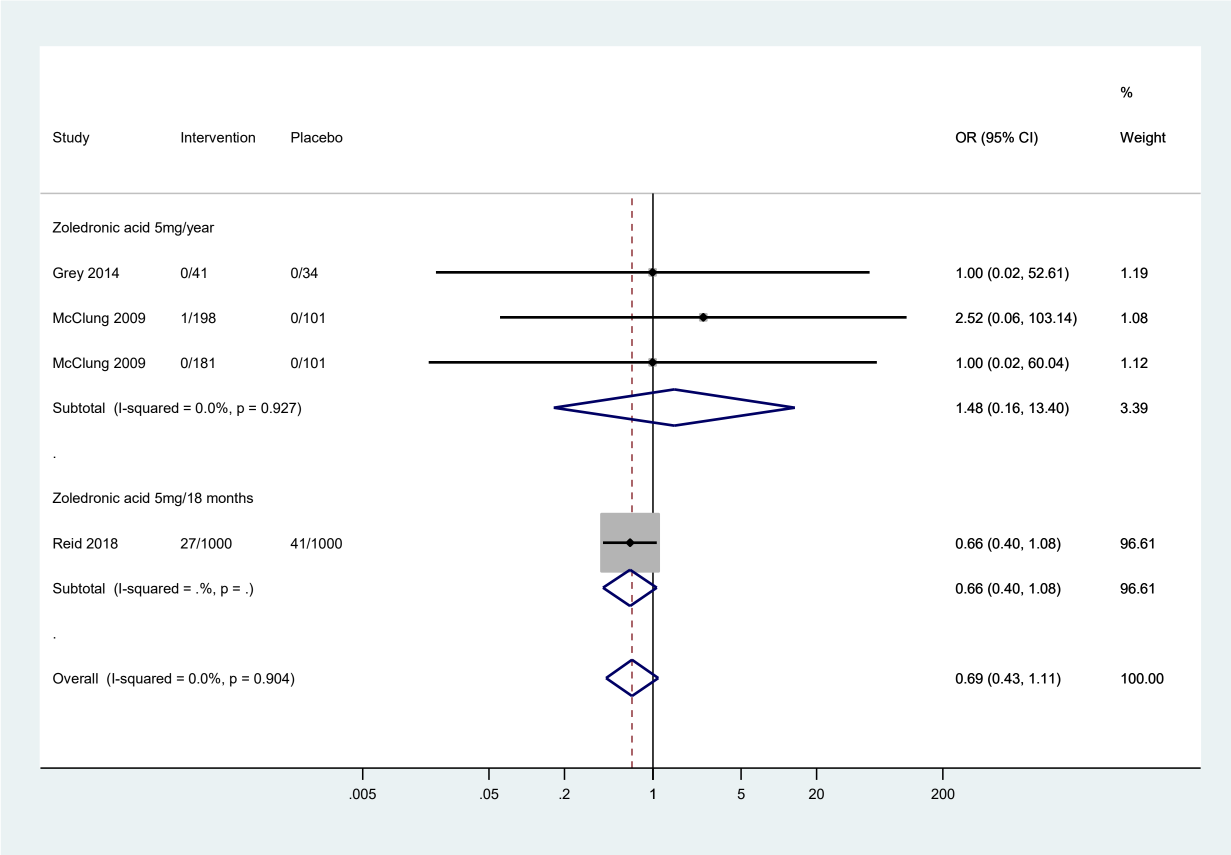

Treatment was 5mg/year for 12 months (1 infusion) for 12 months in Grey 2014 and McClung 2009b; 5 mg/year for 24 months (2 infusions) in McClung 2009a; 5 mg/1.5 years for 72 months (4 infusions) in Reid 2018. Follow-up was to the end of the treatment period for all studies, except McClung 2009b, where follow-up extended for 12 months beyond the end of the treatment period (i.e., 1 infusion at baseline with 2 year follow-up).

KQ3a: What are the benefits of pharmacologic treatments to prevent fragility fractures among adults ≥ 40 years?

Males

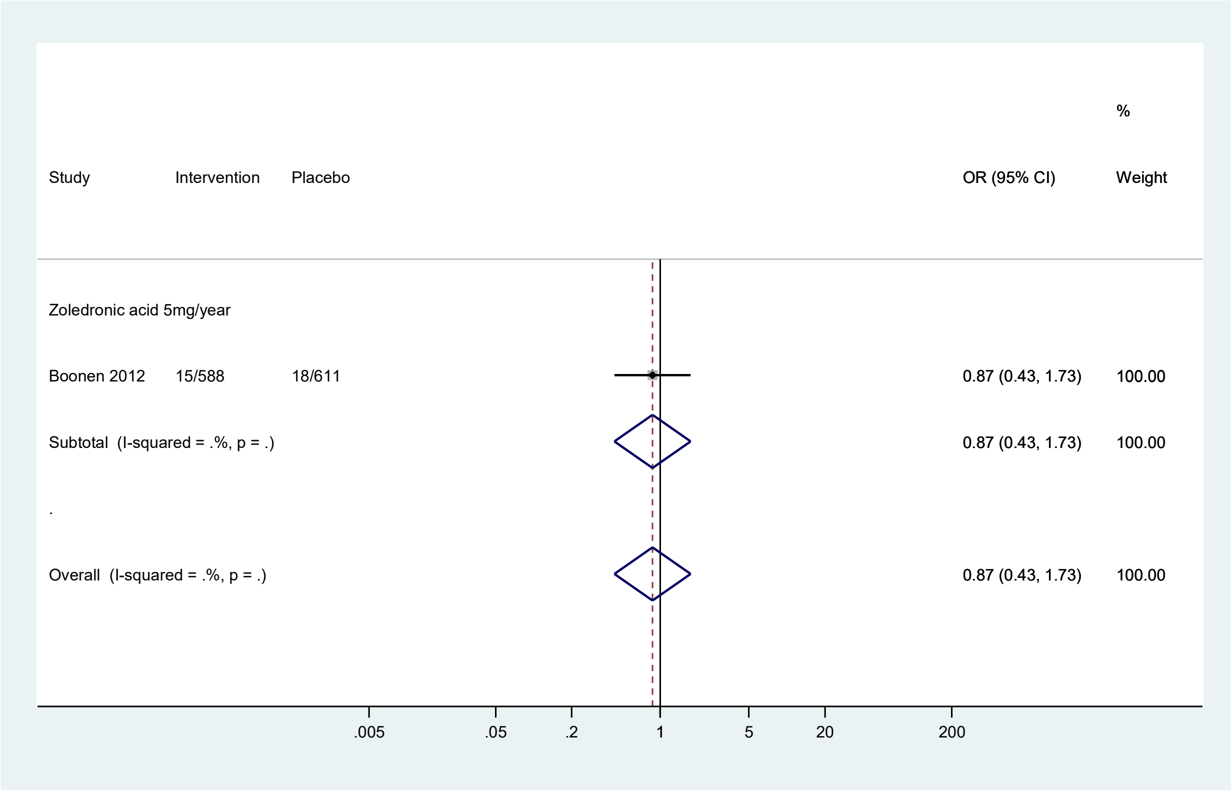

Treatment was 5 mg/year for 24 months (2 infusions), with follow-up to the end of the treatment period.

KQ3a: What are the benefits of pharmacologic treatments to prevent fragility fractures among adults ≥40 years?

4. Bisphosphonates (alendronate, risedronate, zoledronic acid) vs. placebo

4.1 GRADE Summary of Findings

Outcome & Study approach	Studies; sample size	Follow-up (y)	Assumed population risk*	Absolute effects (95% CI)	Certainty	What happens?
**Bisphosphonates (alendronate, risedronate, or zoledronic acid) vs. placebo (postmenopausal females)**						
Hip fractures<br>Intention to treat	14 RCT; 21,038 [35-42, 50-53, 56-59]	1 to 6	Study data:<br>11 in 1000	2.9 fewer in 1000<br>(4.6 fewer to 0.9 fewer)	LOW[a]	May reduce
			General F ≥65 y:<br>20 in 1000	5.3 fewer in 1000<br>(8.3 fewer to 1.6 fewer)		
Clinical fragility fractures<br>Intention to treat or exposed to ≥1 dose	19 RCT; 22,482 [35, 37-40, 42-47, 50-61]	1 to 6	Study data:<br>58 in 1000	11.1 fewer in 1000<br>(15.0 fewer to 6.6 fewer)	MODERATE[b]	Probably reduces
			General F ≥65 y:<br>202 in 1000	33.6 fewer in 1000<br>(46.0 fewer to 19.8 fewer)		
Clinical vertebral fractures;<br>Intention to treat or exposed to ≥1 dose	11 RCT; 8,921 [35-38, 41, 46, 53, 54, 56-59, 61]	1 to 6	Study data:<br>21 in 1000	10.0 fewer in 1000<br>(14.0 fewer to 3.9 fewer)	LOW[c]	May reduce
			General F ≥65 y:<br>27 in 1000	12.8 fewer in 1000<br>(17.9 fewer to 5.0 fewer)		
All-cause mortality<br>Intention to treat or exposed to ≥1 dose	8 RCT; 8,542 [36, 37, 41, 47, 48, 53, 56, 58, 60, 62]	1 to 6	Study data:<br>30 in 1000	5.5 fewer in 1000<br>(11.9 fewer to 3.4 more)	LOW[d]	May not reduce
			General F >65 y:<br>57 in 1000	10.3 fewer in 1000<br>(22.6 fewer to 6.4 more)		

CI=confidence interval; RCT=randomized controlled trial; y=years

* The effects without screening for the general risk population are estimated from PRIOR et al. [49], based on 10 year follow-up. Data for the general population <65 years is not included in the summary table (available on request).

Explanations:

[a] ***Some concern about risk of bias***: the largest contributing trial (McClung 2001) was at high risk of bias due to high (>30%) loss to follow-up. There were no major concerns about the other adequately powered studies. ***No serious concern about inconsistency:*** the adequately powered trials appear to be showing the same direction of effect, with varying degrees of precision. ***Serious concern about indirectness:*** the prevalence of prior fracture in several of the larger trials (Liberman 1995, McClung 2001, Pols 1999) is unknown, and in one of these trials (Liberman 1995) it is possible that the fractures were the result of trauma (i.e., not fragility fractures). The adequately powered trial for each drug provided doses that are not approved for use in Canada (in all cases lower than the approved dose). The clinical utility of findings from this analysis are unclear, since they do not provide information about which bisphosphate might provide benefit. ***No major concerns about imprecision:*** the sample size is large. ***No serious concern for other considerations:*** no other concerns that would further reduce our confidence in the findings.

[b] ***Some concern about risk of bias***: the largest contributing trial (McClung 2001) was at high risk of bias due to high (>30%) loss to follow-up. There were no major concerns about the other adequately powered studies. ***No serious concern about inconsistency:*** the adequately powered trials appear to be showing the same direction of effect, with varying degrees of precision. ***Some concern about indirectness:*** the prevalence of prior fracture in several of the larger trials (Liberman 1995, McClung 2001, Pols 1999) is unknown, and in one of these trials (Liberman 1995) it is possible that the fractures were the result of trauma (i.e., not fragility fractures). The adequately powered trial for each drug provided doses that are not approved for use in Canada (in all cases lower than the approved dose) but the pooled effect still shows a benefit for bisphosphonates (did not rate down). Fractures were self-reported and undefined in four trials and could have included non-clinical vertebral fractures (Bell 2002, Hosking 2003, Lewiecki 2007, McClung 2001); in two trials clinical vertebral and nonvertebral fractures were added to determine the number of events (McClung 2009, Reid 2018). The findings were robust to sensitivity analysis removing self-reports and using only nonvertebral fractures (did not rate down). ***No major concerns about imprecision:*** the sample size is large and the pooled estimate is precise for benefit of bisphosphonates. ***No serious concern for other considerations:*** no other concerns that would further reduce our confidence in the findings.

[c] ***Some concern about risk of bias***: no major risk of bias concerns for the larger contributing trials. The findings could be biased because several of the available trials for each drug did not report on this outcome specifically. ***Some concern about inconsistency:*** lack of evidence of consistency since the pooled effect hinges primarily on two trials, one for alendronate and one for zoledronic acid. There were no adequately powered trials for risedronate. ***No serious concern about indirectness:*** the

adequately powered trials for alendronate and zoledronic acid provided doses that are not approved for use in Canada (in all cases lower than the approved dose) but the pooled effect still shows a benefit for bisphosphonates (did not rate down). ***Some concerns about imprecision:*** the number of events does not meet the optimal information size but the sample size is large. ***No serious concern for other considerations:*** no other concerns that would further reduce our confidence in the findings.

[d] ***Serious concern about risk of bias***: in one of the larger trials (Cummings 1998), it is unclear how the outcome was defined and how it was collected (may not have been collected systematically). Selective reporting strongly suspected, since only 8 of the 22 trials of bisphosphonates in females (36%) reported on this outcome, and appears to be collected passively in the majority of trials. ***Some concern about inconsistency:*** lack of evidence of consistency because the analysis hinges primarily on 0-1 large trials per drug (n=1 alendronate, n=0 risedronate, n=1 zoledronic acid). ***Serious concern about indirectness:*** the main contributing studies provided doses of the drugs that are not approved for use in Canada (lower than the approved dose and showing no difference). ***No serious concern about imprecision:*** the sample size is large. ***No serious concern for other considerations:*** no other concerns that would further reduce our confidence in the findings.

4.2 Contributing data
Bisphosphonates vs. placebo – hip fractures

Females

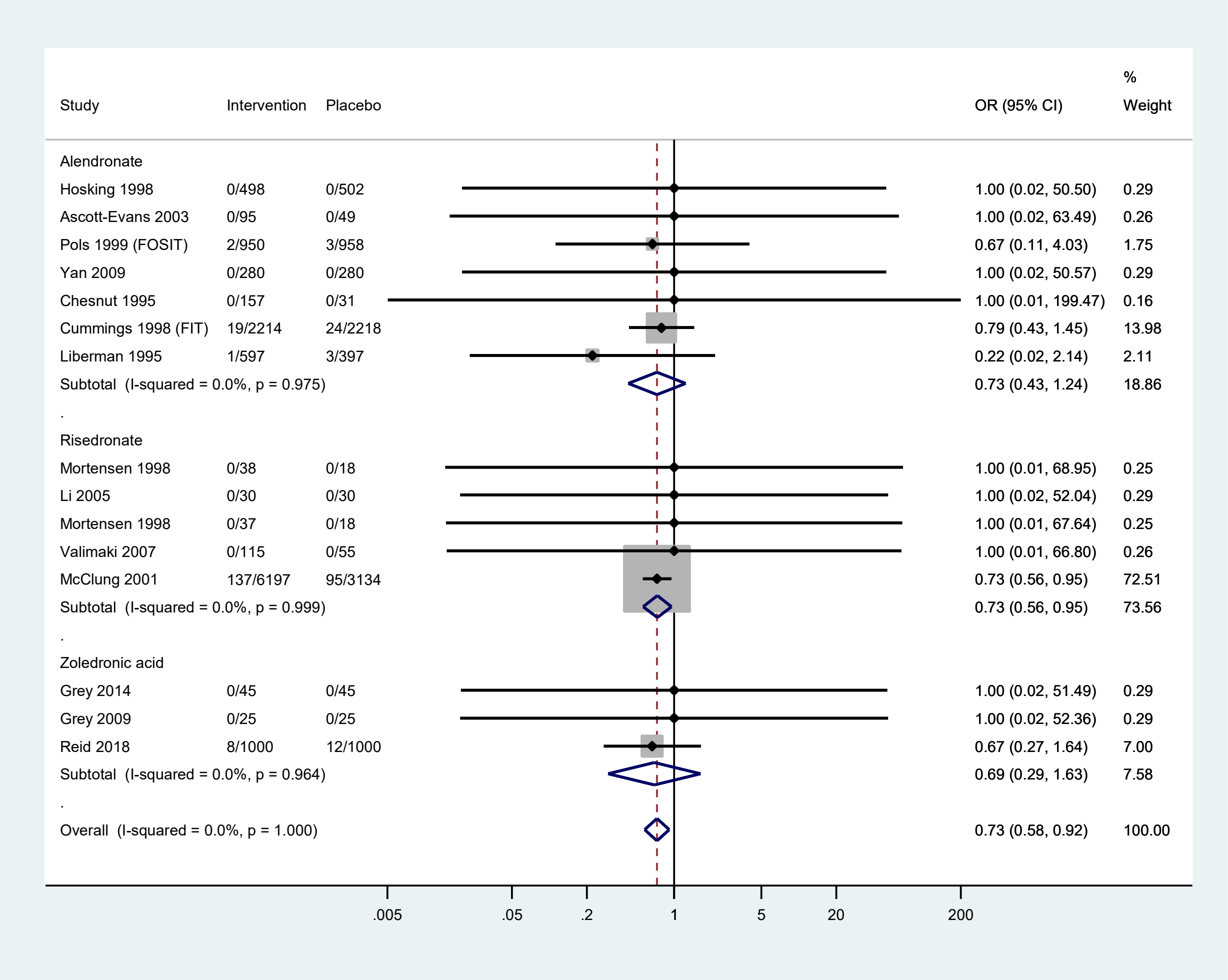

See analyses of individual drugs for details on treatment dosage and duration.

KQ3a: What are the benefits of pharmacologic treatments to prevent fragility fractures among adults ≥40 years?

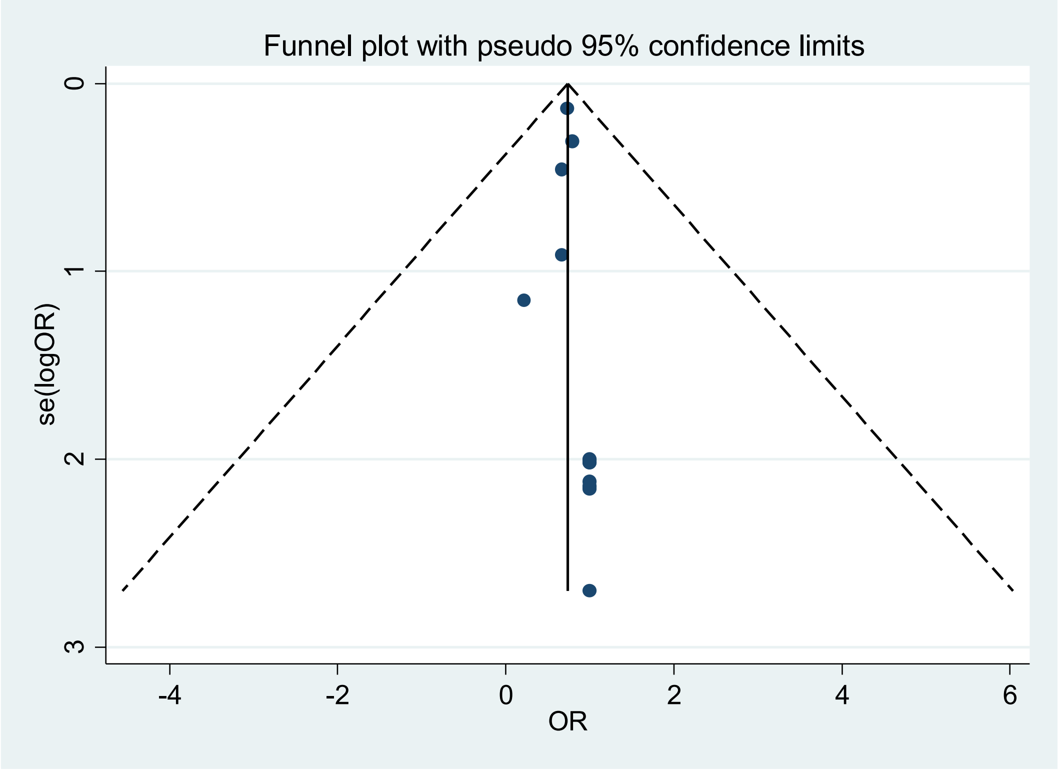

No statistically significant evidence of small study bias (Harbord p-value: 0.669)

KQ3a: What are the benefits of pharmacologic treatments to prevent fragility fractures among adults ≥40 years?

Bisphosphonates vs. placebo – clinical fragility fractures

Females

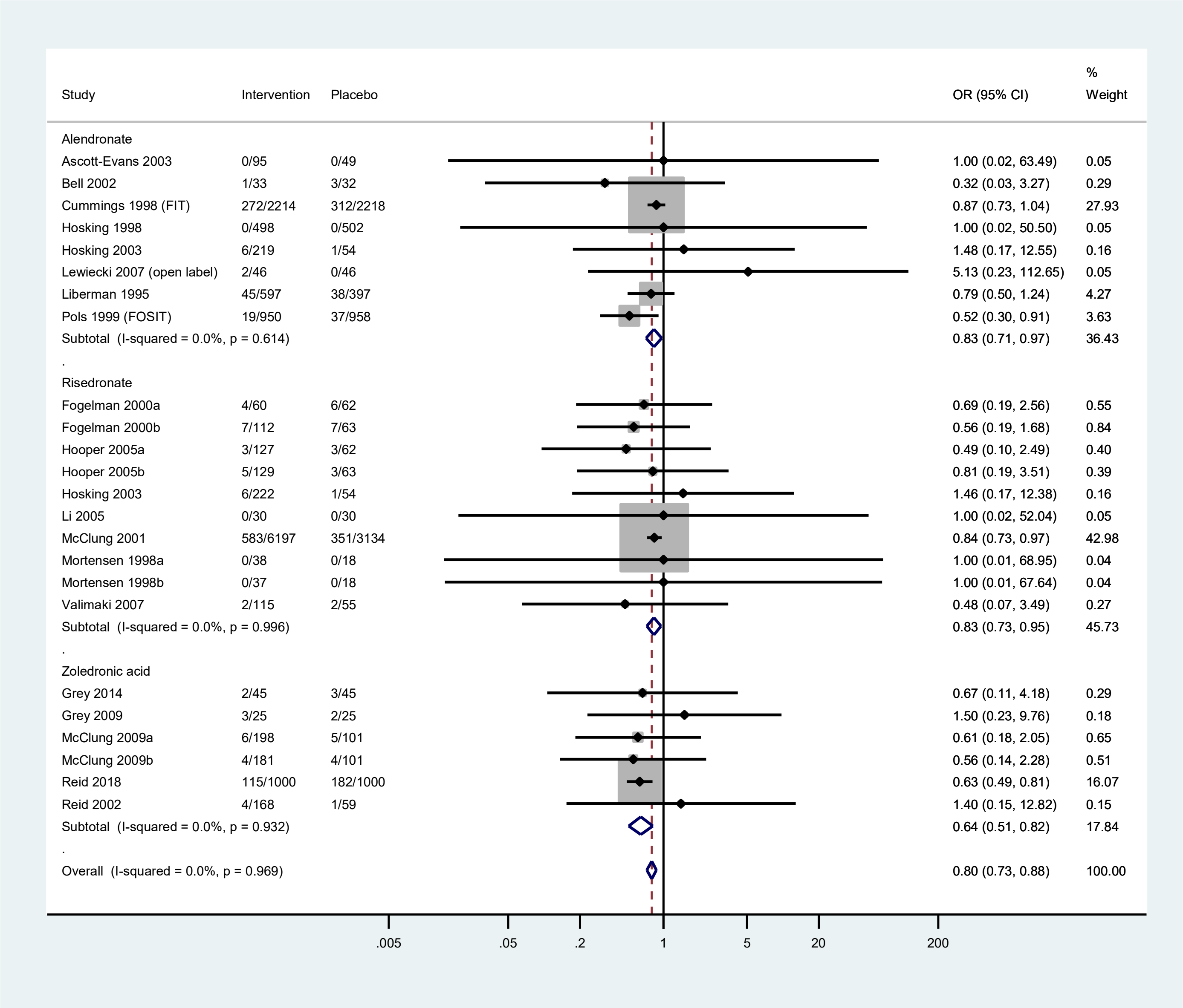

See analyses of individual drugs for details on treatment dosage and duration.

KQ3a: What are the benefits of pharmacologic treatments to prevent fragility fractures among adults ≥ 40 years?

Females - sensitivity analysis

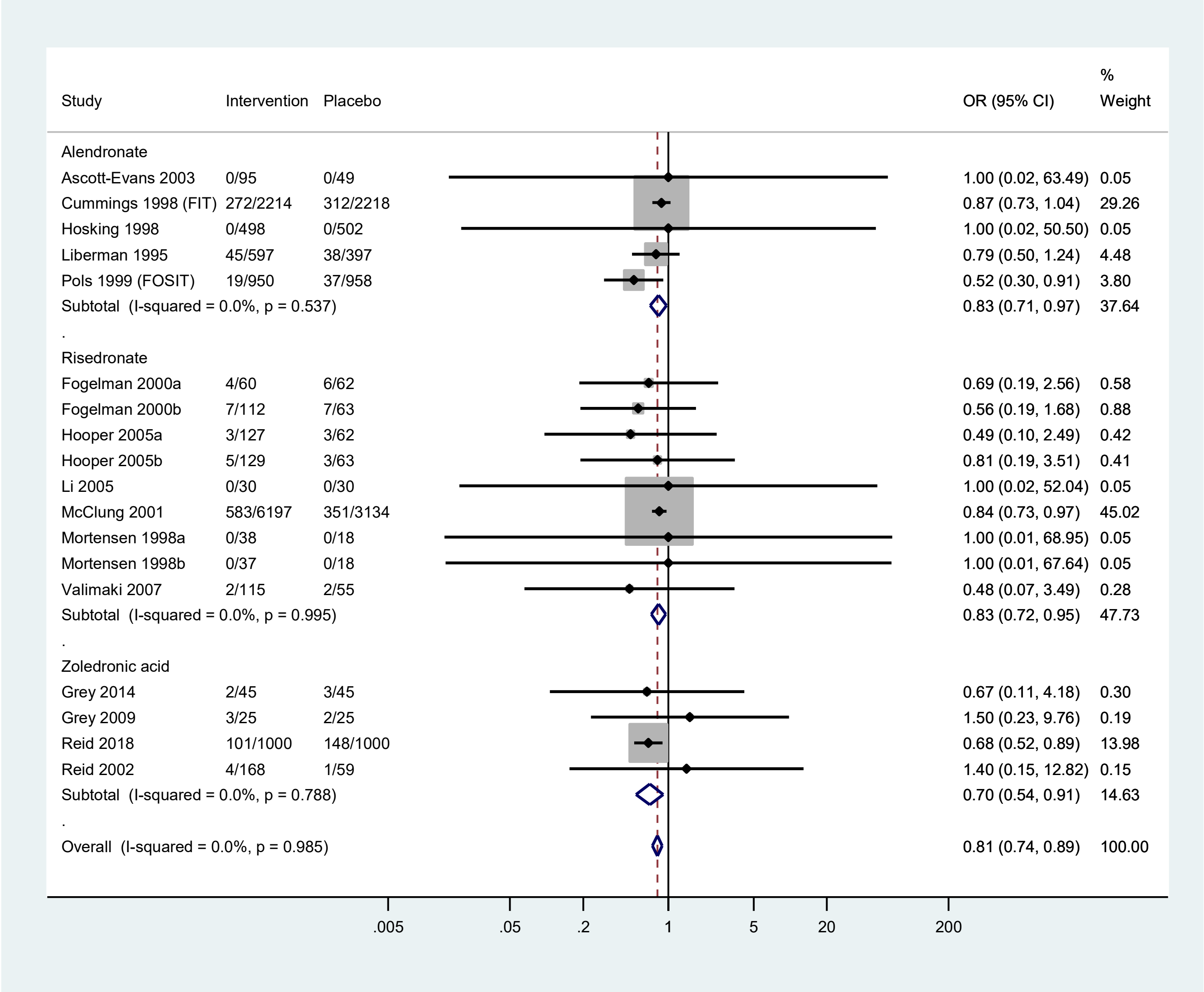

KQ3a: What are the benefits of pharmacologic treatments to prevent fragility fractures among adults ≥40 years?

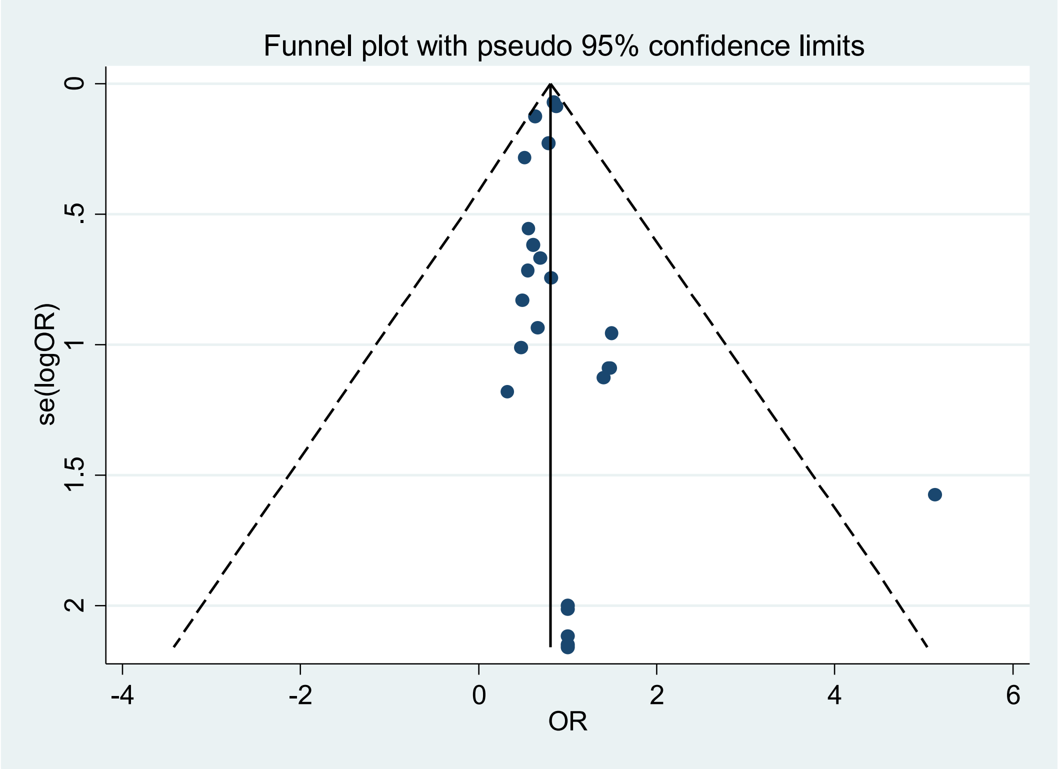

No statistically significant evidence of small study bias (Harbord p-value: 0.674).

KQ3a: What are the benefits of pharmacologic treatments to prevent fragility fractures among adults ≥40 years?

Bisphosphonates vs. placebo – clinical vertebral fractures

Females

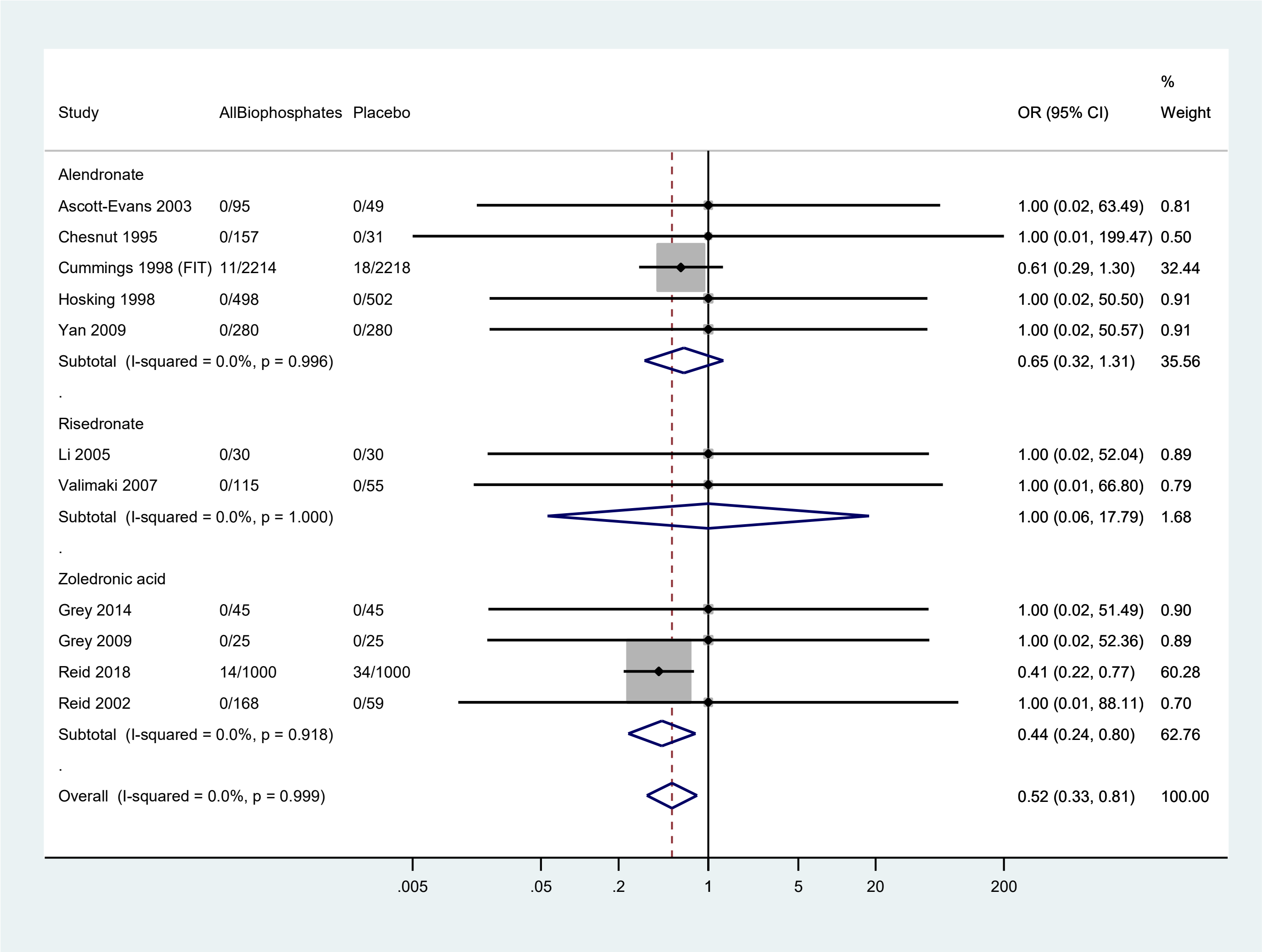

See analyses of individual drugs for details on treatment dosage and duration.

KQ3a: What are the benefits of pharmacologic treatments to prevent fragility fractures among adults ≥ 40 years?

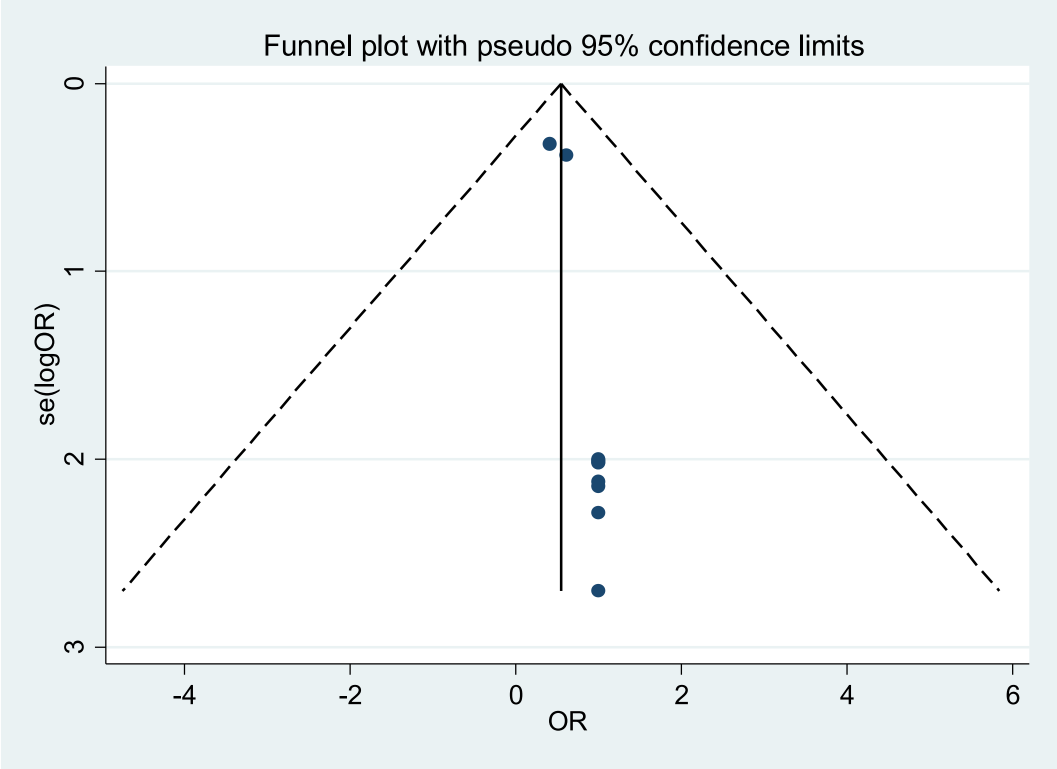

Harbord p-value = 0.002; all the small studies had no events compared to the two larger studies with OR<1. This causes the effect shown in the Harbord test, but from the funnel plot we can clearly see that any bias is inconsequential.

KQ3a: What are the benefits of pharmacologic treatments to prevent fragility fractures among adults ≥ 40 years?

Bisphosphonates vs. placebo – all-cause mortality

Females

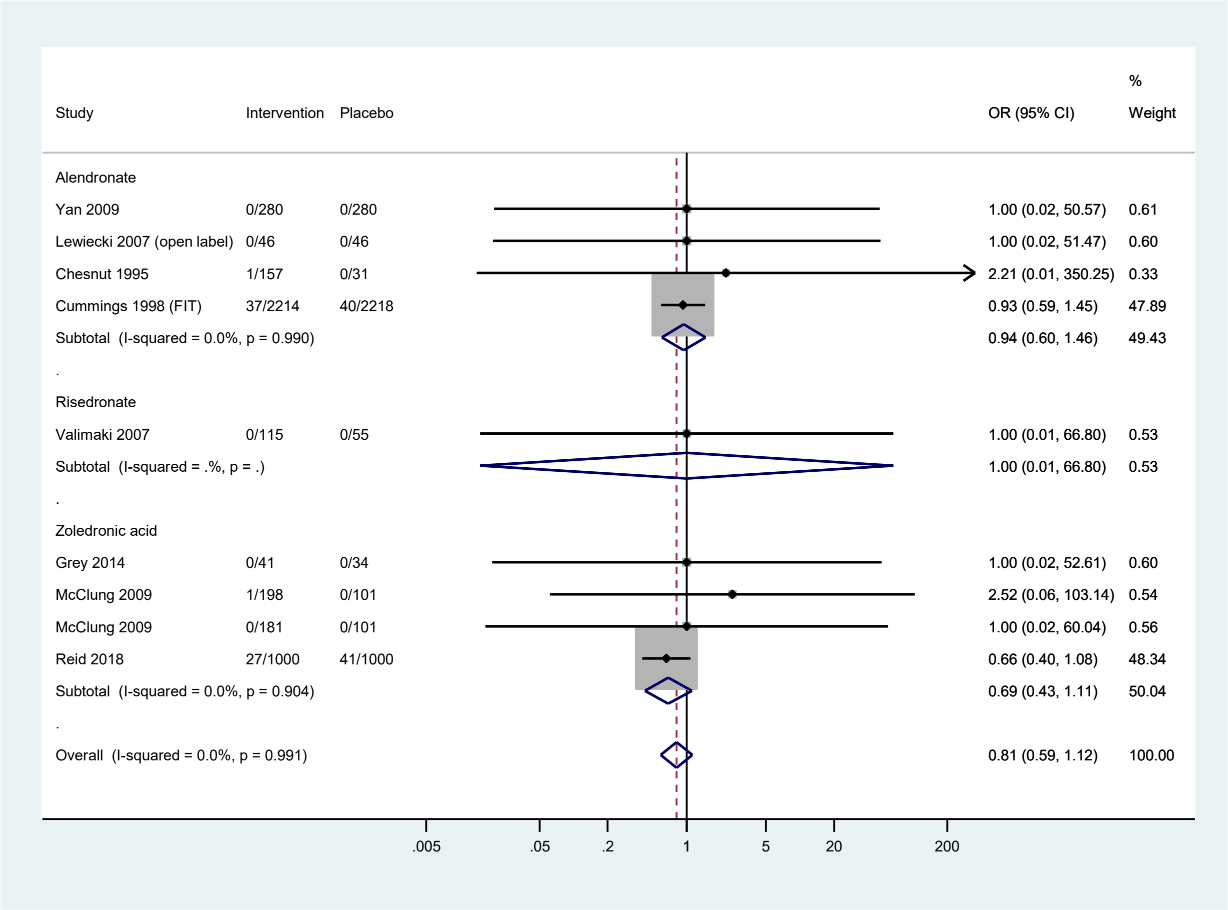

See analyses of individual drugs for details on treatment dosage and duration.

KQ3a: What are the benefits of pharmacologic treatments to prevent fragility fractures among adults ≥40 years?

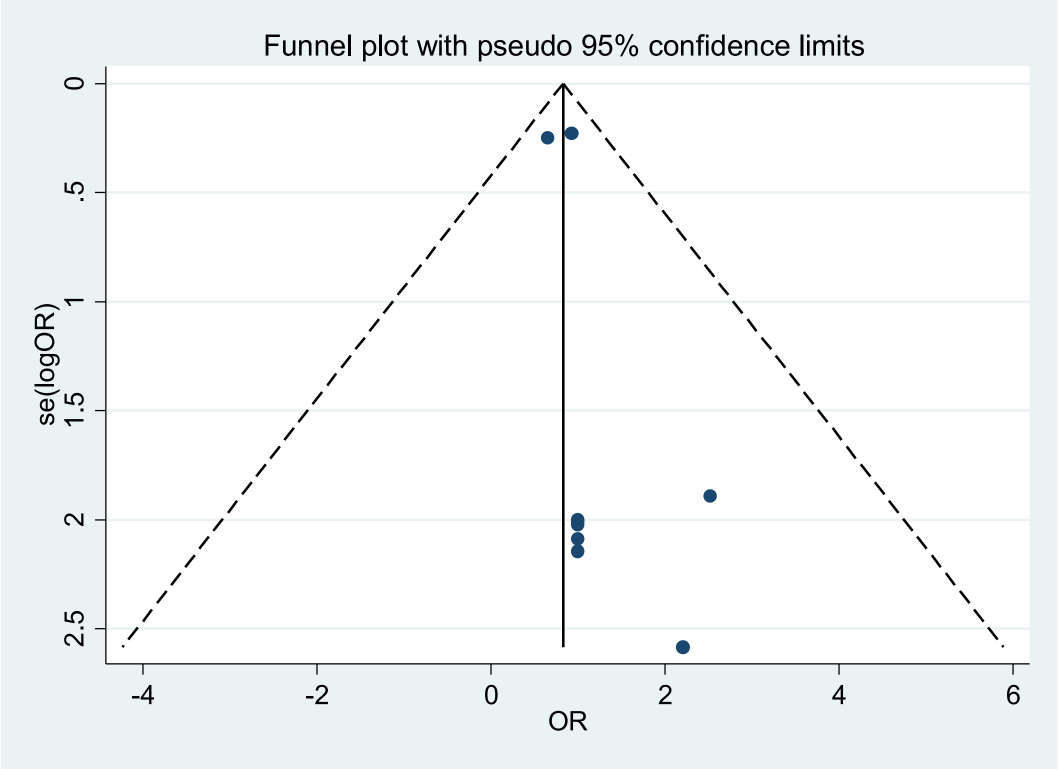

No statistically significance evidence of small study bias (Harbord p-value = 0.225).

KQ3a: What are the benefits of pharmacologic treatments to prevent fragility fractures among adults ≥ 40 years?

5. Denosumab vs. placebo

5.1 GRADE Summary of Findings

Outcome & Study approach	Studies; sample size	Follow-up (y)	Assumed population risk*	Absolute effects (95% CI)	Certainty	What happens?
**Denosumab vs. placebo (postmenopausal females)**						
Hip fractures<br>Intention to treat	3 RCT; 8,542 [64-68]	0.5 to 3	Study data:<br>11 in 1000	3.9 fewer in 1000<br>(6.7 fewer to 0.2 more)	LOW[a]	May not reduce
			General F ≥65 y:<br>20 in 1000	7.1 fewer in 1000<br>(12.1 fewer to 0.4 more)		
Clinical fragility fractures<br>Intention to treat or exposed to ≥1 dose	5 RCT; 9,231 [47, 48, 64-67, 69]	0.5 to 3	Study data:<br>42 in 1000	12.2 fewer in 1000<br>(16.8 fewer to 7.3 fewer)	MODERATE[b]	Probably reduces
			General F ≥65 y:<br>202 in 1000	51.5 fewer in 1000<br>(72.1 fewer to 30.1 fewer)		
Clinical vertebral fractures<br>Intention to treat or exposed to ≥1 dose	3 RCT; 8,397 [64, 67, 69-71]	0.5 to 3	Study data:<br>24 in 1000	16.2 fewer in 1000<br>(18.9 fewer to 12.1 fewer)	MODERATE[c]	Probably reduces
			General F ≥65 y:<br>27 in 1000	18.2 fewer in 1000<br>(21.2 fewer to 13.6 fewer)		
All-cause mortality<br>Intention to treat or exposed to ≥1 dose	5 RCT; 9,185 [47, 48, 64-67, 69, 71]	0.5 to 3	Study data:<br>23 in 1000	4.7 fewer in 1000<br>(9.5 fewer to 1.8 more)	MODERATE[d]	Probably does not reduce
			General F >65 y:<br>57 in 1000	11.4 fewer in 1000<br>(23.1 fewer to 4.3 more)		
Health-related quality of life (OPAQ-SV; 0-100; higher = better) after 3-y of treatment	1 RCT; 6,481 postmenopausal females [72]	3	Change from baseline: physical function (-1.3 vs. -1.2), emotional status (-1.4 vs. -1.6), and back pain (4.1 vs. 4.3) for denosumab vs. placebo.		MODERATE[e]	Probably does not change
**Denosumab vs. placebo (males)**						
Hip fractures<br>Intention to treat	1 RCT; 242 [73]	1	Study data:<br>0 in 1000	No difference in 1000	VERY LOW[f]	Very uncertain
			General M ≥65 y:<br>16 in 1000	No difference in 1000<br>(15.7 fewer to 436.4 more)		
Clinical fragility fractures<br>Intention to treat	1 RCT; 242 [73]	1	Study data:<br>16 in 1000	8.4 fewer in 1000<br>(16.3 fewer to 71.2 more)	VERY LOW[g]	Very uncertain
			General M ≥65 y:<br>105 in 1000	49.6 fewer in 1000<br>(100.3 fewer to 291.1 more)		
Clinical vertebral fractures<br>Intention to treat	1 RCT; 242 [73]	1	Study data:<br>0 in 1000	No difference in 1000	VERY LOW[h]	Very uncertain
			General M ≥65 y:<br>10 in 1000	No difference in 1000<br>(9.8 fewer to 329.1 more)		
All-cause mortality<br>Exposed to ≥1 dose	1 RCT; 240 [73]	1	Study data:<br>8 in 1000	No difference in 1000<br>(7.5 fewer to 107.4 more)	VERY LOW[i]	Very uncertain
			General M >65 y:<br>76 in 1000	No difference in 1000<br>(71.1 fewer to 494.8 more)		

CI=confidence interval; OPAQ-SV=Osteoporosis Assessment Questionnaire - Short Version; RCT=randomized controlled trial; vs=versus; y=years
* The effects without screening for the general risk population are estimated from PRIOR et al. [49], based on 10 year follow-up. Data for the general population <65 years is not included in the summary table (available on request).

Explanations:

[a] ***No serious concern about risk of bias***: the one adequately powered trial (Cummings 2009) is at low risk of bias. ***Serious concern about inconsistency:*** no evidence of consistency because the analysis hinges on one large trial (Cummings 2009); the remaining trials were inadequately powered to show a reduction in hip fractures. ***Some concern about indirectness:*** hip fracture outcome in the Zhu 2017 trial is undefined, so it is unclear if these are fragility fractures. Though the contribution to the analysis is small, the pooled estimate is altered (becomes non-significant) when this study is included. ***Serious concern about imprecision:*** the number of events does not meet the optimal information size (<300), but the sample size is large. The 95% confidence interval for the pooled effect includes the potential for a benefit or for no difference. ***No serious concern for other considerations:*** no other concerns that would further reduce our confidence in the findings.

[b] ***No serious concern about risk of bias***: the one adequately powered trial (Cummings 2009) is at low risk of bias. ***Some concern about inconsistency:*** no evidence of consistency because the analysis hinges on one large trial (Cummings 2009); the remaining trials contribute very little to the analysis. ***No serious concern about indirectness:*** The one large trial uses a dosage lower than the one approved in Canada, but the pooled effect still shows a benefit of denosumab (did not rate down). In one trial the fracture outcome is self-reported and could include non-clinical vertebral fractures (Lewiecki 2007), in another clinical vertebral and nonvertebral fractures were added to determine the number of events (Cummings 2009). The findings were robust to sensitivity analysis removing Lewiecki 2007 and using only nonvertebral fractures for Cummings 2009 (did not rate down). ***No serious concern about imprecision:*** The sample size is adequate and the pooled estimate is precise for a benefit of denosumab. ***No serious concern for other considerations:*** no other concerns that would further reduce our confidence in the findings.

[c] ***No serious concern about risk of bias***: the one adequately powered trial (Cummings 2009) is at low risk of bias. ***Some concern about inconsistency:*** no evidence of consistency because the analysis hinges on one large trial (Cummings 2009); remaining trials are underpowered and have 0 events. ***No serious concern about indirectness:*** The adequately powered trial uses a dosage lower than the one approved in Canada, but the pooled effect still shows a benefit of denosumab (did not rate down). ***No serious concern about imprecision:*** The sample size is adequate and the pooled estimate is precise for a benefit of denosumab. ***No serious concern for other considerations:*** no other concerns that would further reduce our confidence in the findings.

[d] ***No serious concern about risk of bias***: no serious risk of bias concerns for the main contributing trial (Cummings 2009). ***Some concern about inconsistency:*** lack of evidence of consistency because the analysis hinges primarily on one large trial (Cummings 2009). The remaining trials were likely underpowered to detect a difference in mortality. ***No serious concern about indirectness:*** the main contributing studies are well aligned with the review question. ***Serious concern about imprecision:*** the number of events does not meet the optimal information size (n<300), though the sample size is large. The confidence interval includes the potential for both important benefit and no difference. ***No serious concern for other considerations:*** no other concerns that would further reduce our confidence in the findings.

[e] ***No serious concern about risk of bias***: no serious risk of bias concerns for the one contributing trial (Cummings 2009). ***Some concern about inconsistency:*** lack of evidence of consistency because there is only one study reporting on this outcome. ***Some concerns about indirectness:*** analysis includes a selected population of participants who completed all 3 years of the trial (82% placebo, 84% denosumab; 99% of these completed the questionnaire); it is unclear if ratings would be different for those who did not complete the trial. ***No serious concern about imprecision:*** the sample size is large. ***No serious concern for other considerations:*** no other concerns that would further reduce our confidence in the findings.

[f] ***Serious concern about risk of bias:*** the trial was at high risk of reporting bias as the outcome was not mentioned in the trial registration nor methods, and it is unclear whether it was collected systematically. ***Some concern about inconsistency:*** lack of evidence of consistency because there is only one study reporting on this outcome. ***Serious concern about imprecision:*** the study was not adequately powered to detect a difference in fractures, and no events were reported. ***No serious concern for indirectness or other considerations:*** no other concerns that would further reduce our confidence in the findings.

[g] ***Serious concern about risk of bias:*** the trial was at high risk of reporting bias as the outcome was not mentioned in the trial registration nor methods, and it is unclear whether it was collected systematically. ***Some concern about inconsistency:*** lack of evidence of consistency because there is only one study reporting on this outcome. ***Serious concern about imprecision:*** the number of events does not meet the optimal information size (n<300). ***No serious concern for indirectness or other considerations:*** no other concerns that would further reduce our confidence in the findings.

[h] ***Serious concern about risk of bias:*** the trial was at high risk of reporting bias as the outcome was not mentioned in the trial registration nor methods, and it is unclear whether it was collected systematically. ***Some concern about inconsistency:*** lack of evidence of consistency because there is only one study reporting on this outcome. ***Serious concern about imprecision:*** the

study was not adequately powered to detect a difference in fractures, and no events were reported. ***No serious concern for indirectness or other considerations:*** no other concerns that would further reduce our confidence in the findings.

[i] ***Serious concern about risk of bias:*** it is unclear how the outcome was collected (may not have been collected systematically). ***Some concern about inconsistency:*** lack of evidence of consistency because there is only one study reporting on this outcome. ***Serious concern about imprecision:*** the number of events does not meet the optimal information size (n<300), and the sample size is very small. ***No serious concern for indirectness or other considerations:*** no other concerns that would further reduce our confidence in the findings.

5.2 Contributing data
Denosumab vs. placebo – hip fractures

Females

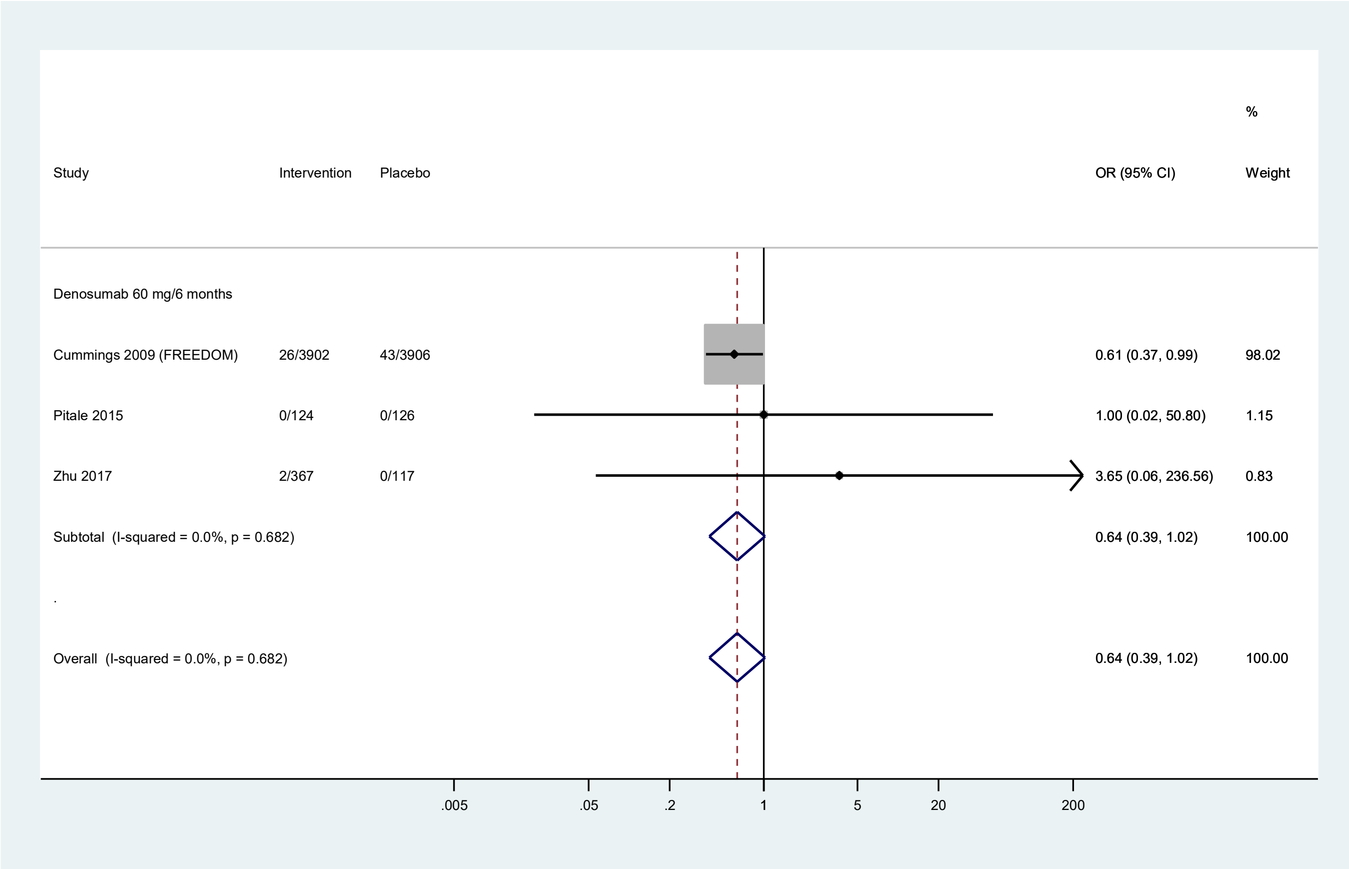

Treatment was as follows: 60mg/6 months for 36 months (6 infusions) in Cummings 2009; 60 mg/6 months for 12 months (2 infusions) in Zhu 2017; 60 mg/6 months for 6 months in Pitale 2015. Follow-up was to the end of treatment.

KQ3a: What are the benefits of pharmacologic treatments to prevent fragility fractures among adults ≥ 40 years?

Males

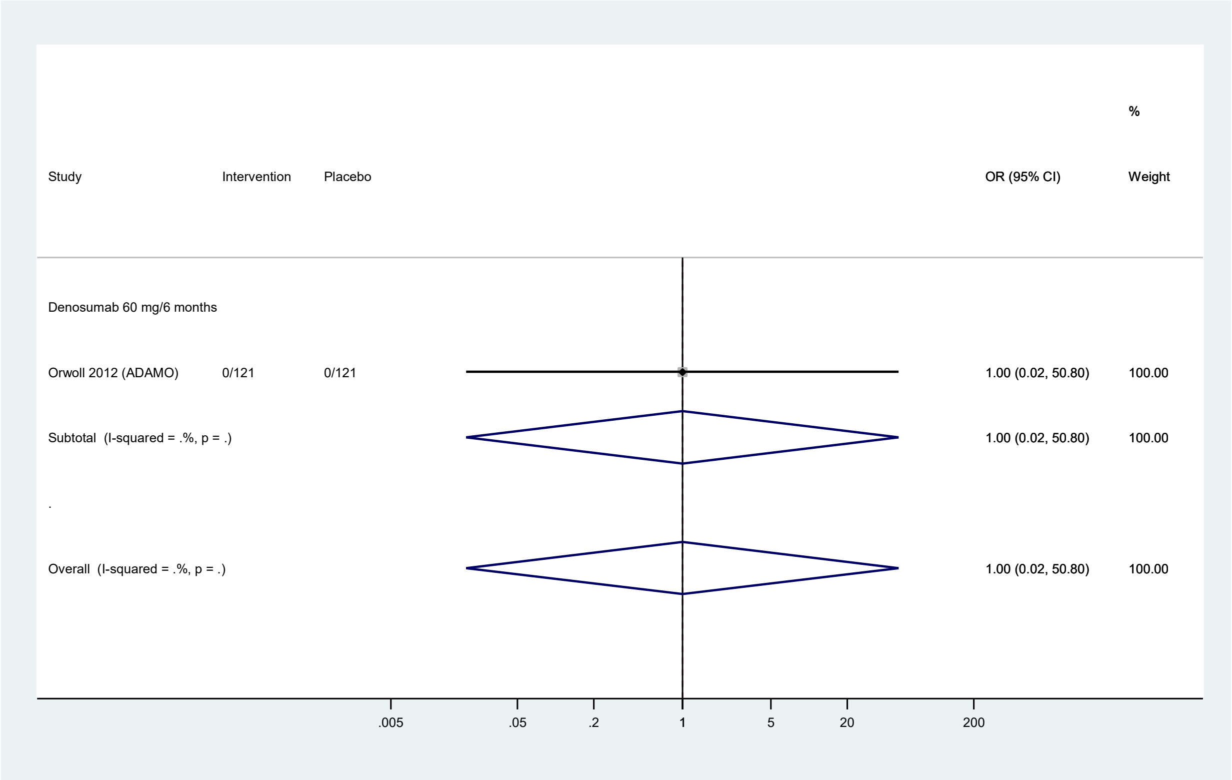

Treatment was 60 mg/6 months for 12 months (2 infusions), with follow-up to end of treatment.

KQ3a: What are the benefits of pharmacologic treatments to prevent fragility fractures among adults ≥40 years?

Denosumab vs. placebo – clinical fragility fractures

Females

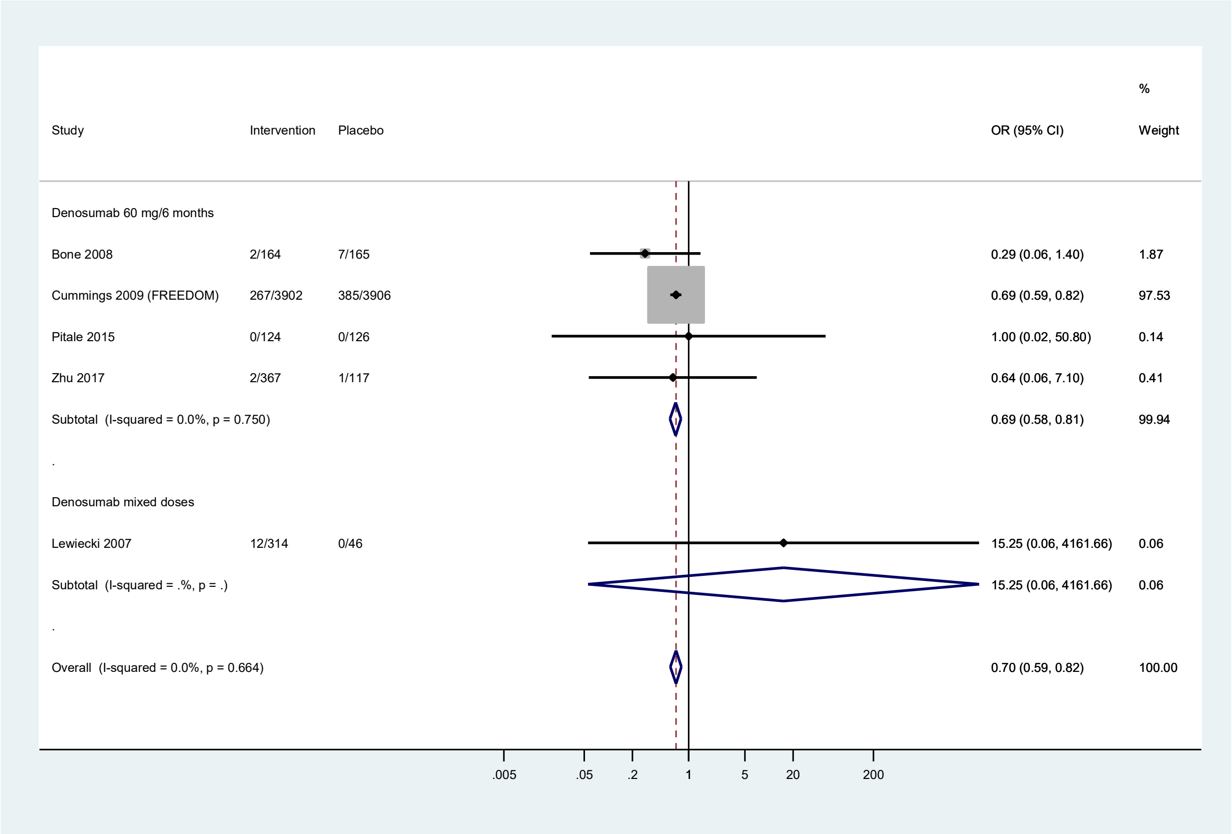

Treatment was as follows: 60 mg/6 months for 12 months (2 infusions) in Zhu 2017; 60 mg/6 months for 24 months (4 infusions) in Bone 2008; 60mg/6 months for 36 months (6 infusions) in Cummings 2009; and 6 or 14 or 30 mg/3 months or 14 or 60 or 100 or 210 mg/6 months for 24 months (4 or 8 infusions) in Lewiecki 2007. Follow-up was to the end of treatment.

Summary of within-study subgroup data for clinical fragility fractures in females

Population	Within-study subgroup data
Femoral neck BMD T-score [64, 68]	**≤-2.5:** 105/1384 [8.1%] vs. 159/1406 [12.3%]; HR 0.65 (0.51, 0.83)<br>**>2.5:** 128/2495 [5.5%] vs. 131/2484 [5.6%]; HR 0.97 (0.76 to 1.23)
Age [64, 68]	**≥75 years:** 88/1235 [7.9%] vs. 102/1236 [9.0%]; HR 0.84 (0.63, 1.12)<br>**<75 years:** 150/2667 [5.9%] vs. 191/2671 [7.6%]; HR 0.78 (0.63, 0.96)
Prior nonvertebral fracture [64, 68]	**Yes:** 103/1163 [9.4%] vs. 121/1177 [11.2%]; HR 0.84 (0.65, 1.09)<br>**No:** 135/2737 [5.3%] vs. 172/2724 [6.6%]; HR 0.77 (0.62, 0.97)
Prevalent vertebral fracture [64, 68]	**Yes:** 84/929 [9.6%] vs. 77/915 [9.2%]; HR 1.06 (0.78, 1.44)<br>**No:** 151/2864 [5.7%] vs. 209/2854 [7.7%]; HR 0.71 (0.62, 0.97)
FRAX ± BMD [64, 68]	*"[…] increasing efficacy of denosumab as baseline probability increased from 5% to 18%. At fracture probabilities higher than 18%, no further increase in efficacy with higher probabilities was observed. For example, at 10% probability (23rd percentile), denosumab decreased fracture risk by 11% (p=0.629), whereas at 20% (70th percentile) the reduction was 71% (p<0.001) and at 30% (90th percentile) it was 50% (p=0.001). A similar pattern was observed if major fracture probability was calculated without the input of femoral neck BMD, or if hip fracture probabilities were used."*

KQ3a: What are the benefits of pharmacologic treatments to prevent fragility fractures among adults ≥ 40 years?

Males

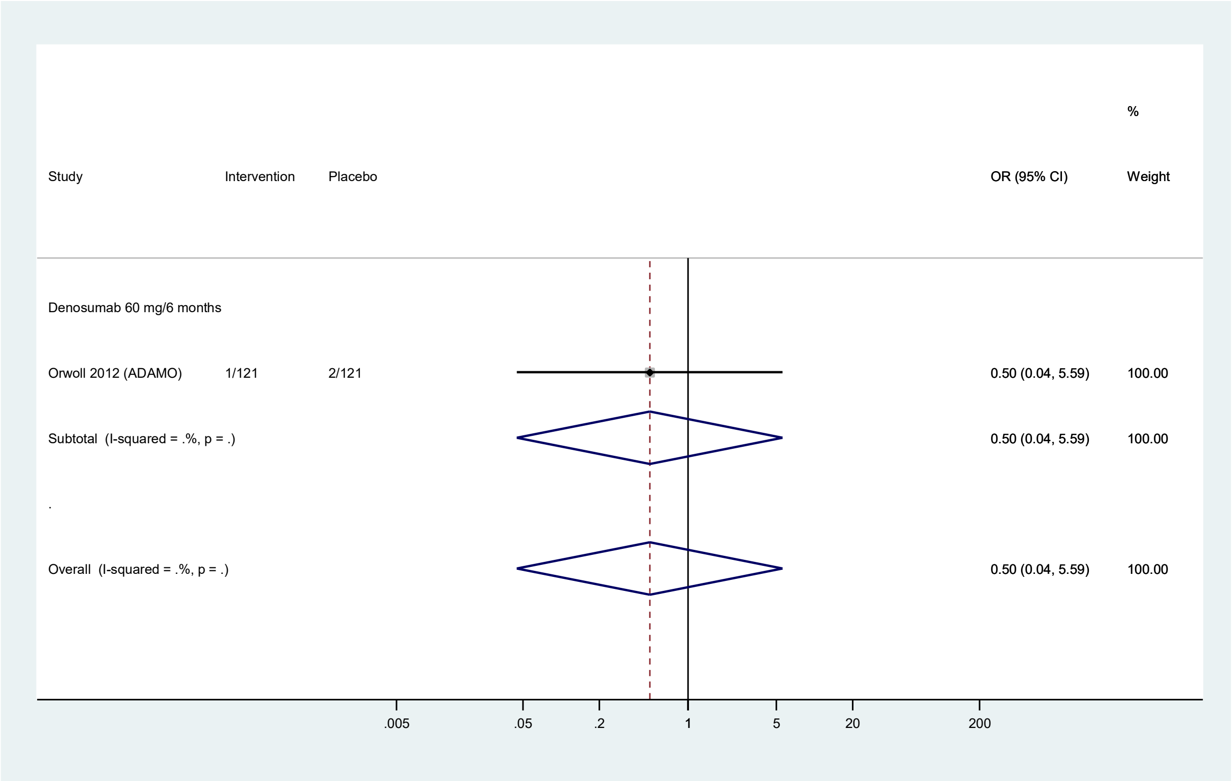

Treatment was 60 mg/6 months for 12 months (2 infusions). Follow-up was to the end of treatment.

KQ3a: What are the benefits of pharmacologic treatments to prevent fragility fractures among adults ≥ 40 years?

Denosumab vs. placebo – clinical vertebral fractures

Females

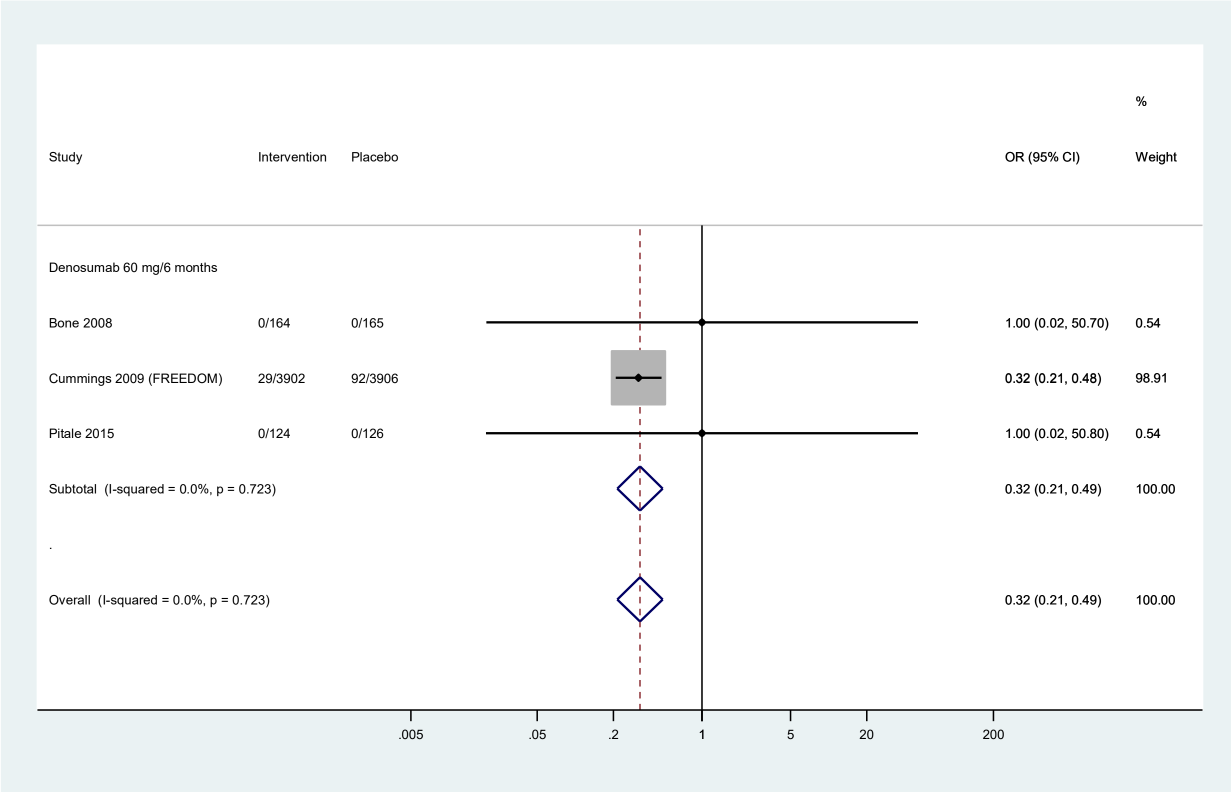

Treatment was as follows: 60 mg/6 months for 6 months (1 infusion) for Pitale 2015; 60 mg/6 months for 24 months (4 infusions) in Bone 2008; 60mg/6 months for 36 months (6 infusions) in Cummings 2009. Follow-up was to the end of treatment.

KQ3a: What are the benefits of pharmacologic treatments to prevent fragility fractures among adults ≥ 40 years?

Males

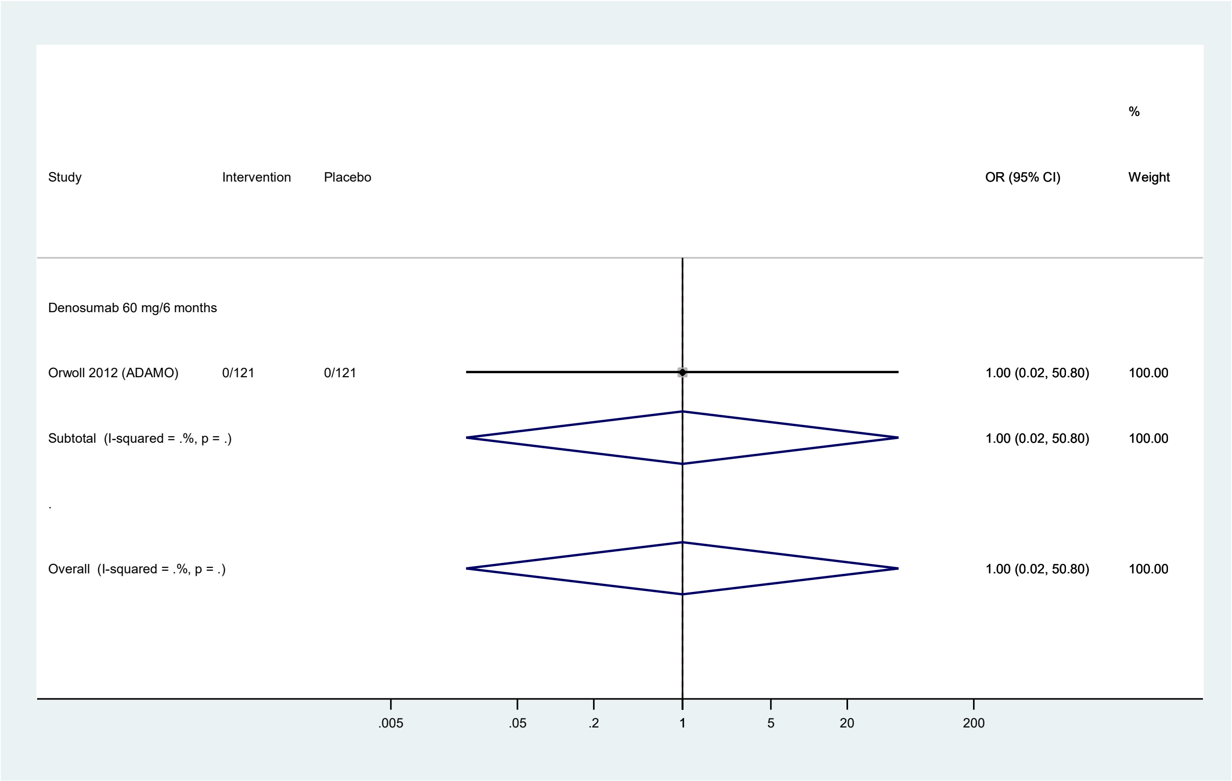

Treatment was 60 mg/6 months for 12 months (2 infusions). Follow-up was to the end of treatment.

KQ3a: What are the benefits of pharmacologic treatments to prevent fragility fractures among adults ≥40 years?

Denosumab vs. placebo – all-cause mortality

Females

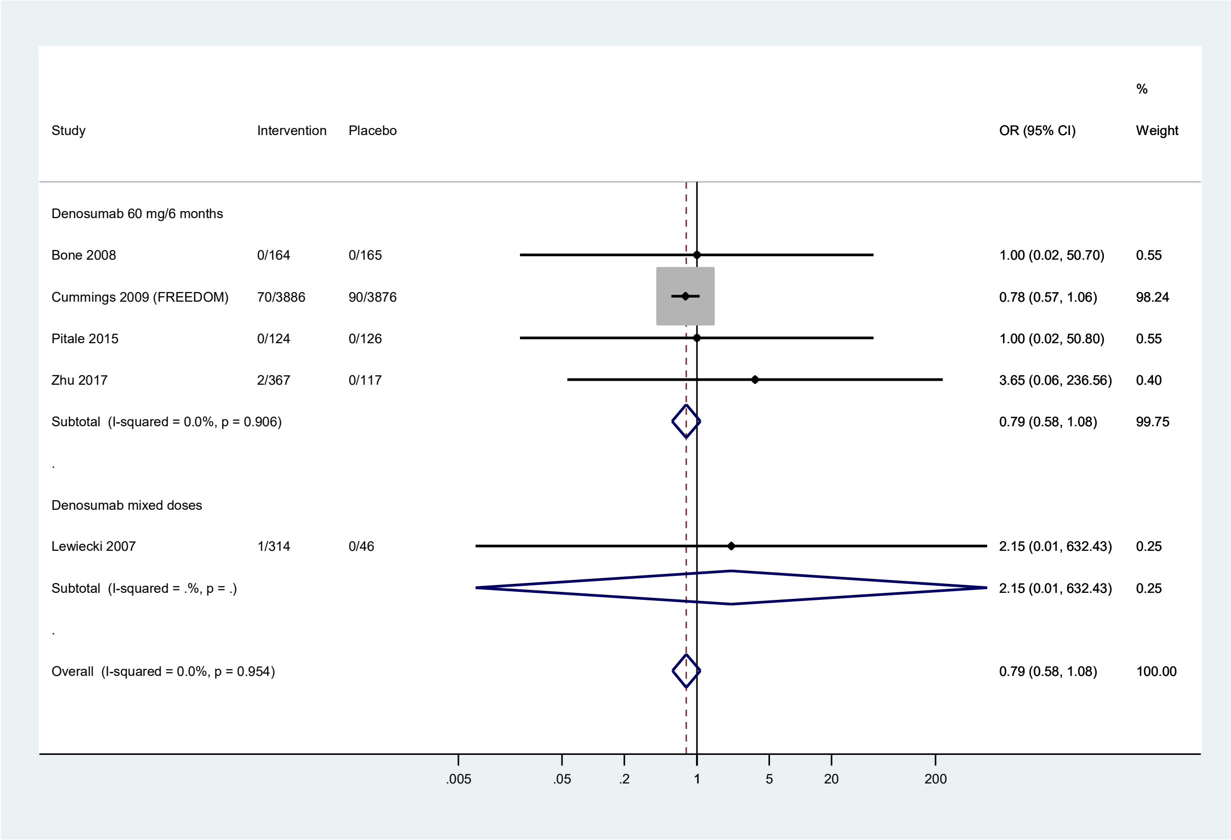

Treatment was as follows: 60 mg/6 months for 6 months in Pitale 2015; 60 mg/6 months for 12 months (2 infusions) in Orwoll 2012 and Zhu 2017; 60 mg/6 months for 24 months in Bone 2008; 60mg/6 months for 36 months (6 infusions) in Cummings 2009; 6 mg or 14 mg or 30 mg/3 months, or 14 mg or 60 mg or 100 mg or 210 mg/6 months for 24 months (8 or 4 infusions) in Lewiecki 2007. Follow-up was to the end of treatment.

KQ3a: What are the benefits of pharmacologic treatments to prevent fragility fractures among adults ≥ 40 years?

Males

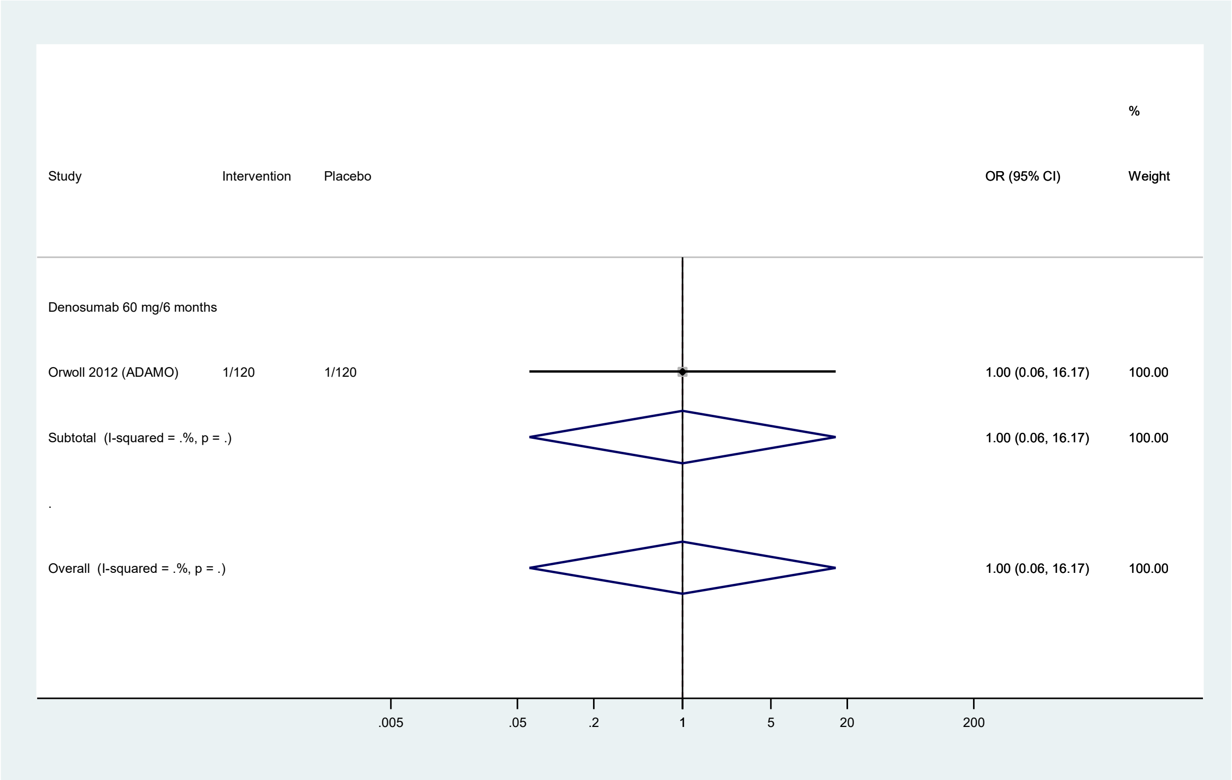

Treatment was 60 mg/6 months for 12 months (2 infusions). Follow-up was to the end of treatment.

KQ3b: What are the harms of pharmacologic treatments to prevent fragility fractures among adults ≥40 years?

EVIDENCE SUMMARY FOR KQ3b ON THE HARMS OF PHARMACOLOGIC TREATMENTS

Background and approach to GRADE

- We appraised the evidence based on presence/direction of an effect, not magnitude (no thresholds)
- Preferentially used GRADE assessments as reported by the authors. In some cases we altered these as appropriate.
- When authors did not report GRADE appraisals, we perform these using data available within the systematic review.
- In several cases, the information needed to perform GRADE appraisals was inadequately reported, thus we made the following assumptions:
    a) Risk of bias: For RCTs, rate down 1 level in all cases where ROB has not been assessed for harms outcomes (i.e., no evidence that risk of bias is low). For observational studies, start at low certainty to capture the likely bias in assessment and reporting of harms data.
    b) Imprecision & inconsistency: When inadequate information, these were combined into a single rating. For imprecision, relied primarily on sample size (i.e., 300 events; n=4000 for rare outcomes), while consistency accounted for the width of the confidence interval if a forest plot was not provided.
    c) Indirectness: In most cases did not rate down, because selection of SRs means that most of the evidence is direct. Rated down when systematic reviews reported composite outcomes and these did not represent the number of patients with one or more of the outcomes.
- Where it was reasonable (i.e., at least low certainty of evidence, statistically significant effect, 5 or fewer included studies), we looked to the included studies to provide absolute effects when these were not reported by the included SRs. For larger analyses, we looked at the five largest contributing studies.

1. Alendronate vs. placebo or no treatment

1.1 GRADE Summary of Findings

Outcome	Studies; sample size	Assumed pop. risk*	Absolute effects (95% CI)	Certainty	What happens?
**Alendronate vs. placebo or no treatment**					
**Serious adverse events**					
Atypical femoral fractures (subtrochanteric) [74]	1 cohort; 220,360	0.06 per 1000	0.08 more per 1000 (0.05 more to 0.14 more)	LOW[a]	May increase
Atypical femoral fractures (femoral shaft) [74]	1 cohort; 220,360	0.03 per 1000	0.06 more per 1000 (0.03 more to 0.10 more)	LOW[b]	
Osteonecrosis of the jaw [74]	1 cohort; 220,360	0.1 per 1000	0.22 more per 1000 (0.04 more to 0.59 more)	LOW[c]	
Any serious AE [3, 75]	5 RCT; 1,955	106 per 1000	5.7 fewer per 1000 (31.9 fewer to 29.4 more)	LOW[d]	May not increase
GI perforations, ulcers, bleeds [76-78]	10 RCT; 137 events	NR	Cannot be calculated; NS difference	LOW[e]	
Serious esophageal AE [76-78]	5 RCT; 499,062	NR	Cannot be calculated; NS difference	LOW[f]	
Atrial fibrillation [76-78]	1 RCT; NR	14 per 1000	3.6 more per 1000 (0.6 fewer to 9.0 more)	LOW[g]	
	1 SR of 32 RCT; 17,291		2.2 more per 1000 (1.8 fewer to 7.7 more)		
Very uncertain: serious GI AEs (any)[h] [76-78], GI cancer (colorectal[i], gastric[j], esophageal[k], liver[l], pancreatic[m], oral[n], bile duct[o], small intestinal[p]) [79], serious cardiovascular AE (acute coronary syndrome[q], cerebrovascular death[r], thromboembolic events[s]) [76-78], and atypical femoral fractures (any[t], with long term treatment [>3 years][u]) [74].<br>No evidence: serious stroke, pulmonary embolism					
**Non-serious adverse events and discontinuation due to AE**					
Non-serious GI AE [76-78]	50 RCT; 22,549	589 per 1000	16.3 more per 1000 (2.4 more to 31.3 more)	MODERATE[v]	Probably increases
Discontinuation due to AE [3, 75]	9 RCT; 9,160	68 per 1000	1.4 fewer per 1000 (10.0 fewer to 8.3 more)	MODERATE[w]	Probably does not increase
Any non-serious AE [80]	5 RCT; 4,720	815 per 1000	16.3 fewer per 1000 (81.5 fewer to 48.9 more)	LOW[x]	May not increase

KQ3b: What are the harms of pharmacologic treatments to prevent fragility fractures among adults ≥40 years?

Outcome	Studies; sample size	Assumed pop. risk*	Absolute effects (95% CI)	Certainty	What happens?
Very uncertain: influenza-like symptoms[y] [80] and musculoskeletal (arthritis and arthralgia[z]; myalgia, cramps, and limb pain[aa]) AEs [76-78].					

AE=adverse event; GI=gastrointestinal; NR=not reported; NS=not statistically significant; RCT=randomized controlled trial; SR=systematic review

* The control event rate is the median rate in the control group for studies in the analysis. These were extracted directly from the systematic reviews when possible. Otherwise, we extracted these data from the included primary studies when there were ≤5 in the analysis or used the 5 largest studies from larger analyses to calculate the control event rate.

Explanations:

[a] Atypical femoral fractures (subtrochanteric): **Rated by Fink 2019.** Authors note moderate risk of bias, unknown consistency (1 study), imprecise, direct, reporting bias undetected, large magnitude of effect (+1). Revised to not rate down for imprecision, as the sample size is large and the entire confidence interval is showing harm.

[b] Atypical femoral fractures (femoral shaft): **Rated by Fink 2019.** Authors note moderate risk of bias, unknown consistency (1 study), imprecise, direct, reporting bias undetected, large magnitude of effect (+1). Revised to not rate down for imprecision, as the sample size is large and the entire confidence interval is showing harm.

[c] Osteonecrosis of the jaw: **Rated by Fink 2019.** Authors note moderate risk of bias, unknown consistency (1 study), imprecise, direct, reporting bias undetected, large magnitude of effect (+1). Revised to not rate down for imprecision, as the sample size is large and the entire confidence interval is showing harm.

[d] Any serious adverse event: ***serious concerns about risk of bias**:* risk of bias not assessed for harm outcomes, rated down one level due to likely bias in harms ascertainment and reporting; ***very serious concerns about imprecision:*** sample size may be adequate (>1000), but the confidence interval is close to the threshold for harm and benefit; ***no serious concerns about inconsistency, indirectness, or other considerations***.

[e] Gastrointestinal perforations, ulcers, and bleeds: ***serious concerns about risk of bias**:* risk of bias not assessed for harm outcomes, rated down one level due to likely bias in harms ascertainment and reporting; ***serious concerns about imprecision:*** the number of events does not meet the optimal information size (<300); ***no serious concerns about inconsistency, indirectness, or other considerations***.

[f] Serious esophageal adverse events: ***serious concerns about risk of bias**:* risk of bias not assessed for harm outcomes, rated down one level due to likely bias in harms ascertainment and reporting; ***serious concerns about inconsistency/imprecision:*** no information on the sample size, confidence interval includes both important harm and no effect; ***no serious concerns about indirectness or other considerations***.

[g] Atrial fibrillation: **Rated by Crandall 2014.** ***Based on independent rating, serious concerns about risk of bias**:* risk of bias not assessed for harm outcomes, rated down one level due to likely bias in harms ascertainment and reporting; ***some concerns about inconsistency:*** some inconsistency, the large trials show both benefit and harm; ***serious concerns about imprecision:*** the sample size is large but the confidence interval is wide, including both important benefit and no effect; ***no serious concerns about indirectness or other considerations***.

[h] All serious gastrointestinal adverse events: ***serious concerns about risk of bias**:* risk of bias not assessed for harm outcomes, rated down one level due to likely bias in harms ascertainment and reporting; ***serious concerns about imprecision:*** confidence interval includes both benefit and no effect; ***serious concerns about indirectness:*** because the number of events is unlikely to reflect the number of people with ≥1 event; ***no serious concerns about inconsistency or other considerations***.

[i] Colorectal cancer: ***no serious concerns about risk of bias:*** the systematic review authors did not report major risk of bias concerns; ***serious concerns about inconsistency:*** presence of unexplained heterogeneity, I^2=80%; ***no serious concerns for imprecision, indirectness or other considerations***.

[j] Gastric cancer: ***no serious concerns about risk of bias:*** the systematic review authors did not report major risk of bias concerns; ***serious concerns about imprecision:*** the sample size is large, but confidence interval includes harm and no difference; ***no serious concerns for inconsistency (I^2=24%), indirectness or other considerations***.

[k] Esophageal cancer: ***no serious concerns about risk of bias:*** the systematic review authors did not report major risk of bias concerns; serious concerns about inconsistency: studies show differing directions of effect, I^2=52%; ***serious concerns about imprecision:*** the sample size is large, but confidence interval includes harm and benefit (related to inconsistency); ***no serious concerns for indirectness or other considerations***.

[l] Liver cancer: ***no serious concerns about risk of bias:*** the systematic review authors did not report major risk of bias concerns; ***serious concerns about inconsistency:*** presence of unexplained heterogeneity, I^2=65%; ***no serious concerns for imprecision, indirectness, or other considerations***.

[m] Pancreatic cancer: ***no serious concerns about risk of bias:*** the systematic review authors did not report major risk of bias concerns; ***serious concerns about imprecision:*** the sample size is large, but confidence includes both harm and no effect; ***no serious concerns for inconsistency (I^2=7%), indirectness, or other considerations***.

[n] Oral cancer: ***no serious concerns about risk of bias:*** the systematic review authors did not report major risk of bias concerns; ***some concerns about inconsistency:*** no evidence of consistency since there is only one study in the analysis; ***very serious concerns about imprecision:*** the sample size is large, but confidence interval is very wide, including both important benefit and harm; ***no serious concerns for indirectness or other considerations***.

[o] Bile duct cancer: ***no serious concerns about risk of bias:*** the systematic review authors did not report major risk of bias concerns; ***some concerns about inconsistency:*** no evidence of consistency since there is only one study in the analysis; ***serious concerns about imprecision:*** the sample size is large, but confidence interval includes harm and no effect; ***no serious concerns for indirectness or other considerations***.

[p] Small intestinal cancer: ***no serious concerns about risk of bias:*** the systematic review authors did not report major risk of bias concerns; ***some concerns about inconsistency:*** no evidence of consistency since there is only one study in the analysis; ***very serious concerns about imprecision:*** the sample size is large, but confidence interval is very wide, including both important benefit and harm; ***no serious concerns for indirectness or other considerations***.

[q] Acute coronary syndrome: ***serious concerns about risk of bias:*** risk of bias not assessed for harm outcomes, rated down one level due to likely bias in harms ascertainment and reporting; ***very serious concerns about inconsistency/imprecision:*** the sample size is not reported and there is no forest plot, but the confidence interval is very wide, including both important benefit and harm; ***no serious concerns about indirectness or other considerations***.

[r] Cerebrovascular death: ***serious concerns about risk of bias:*** risk of bias not assessed for harm outcomes, rated down one level due to likely bias in harms ascertainment and reporting; ***very serious concerns about inconsistency/imprecision:*** the sample size is not reported and there is no forest plot, but the confidence interval is very wide, including both important benefit and harm; ***no serious concerns about indirectness or other considerations***.

[s] Thromboembolic events: ***serious concerns about risk of bias****:* risk of bias not assessed for harm outcomes, rated down one level due to likely bias in harms ascertainment and reporting; ***some concerns about inconsistency:*** no evidence of consistency since there is only one trial in the analysis; ***very serious concerns about imprecision:*** the confidence interval is very wide, including both important benefit and harm; ***no serious concerns about indirectness or other considerations***.

[t] Atypical femoral fractures (any; vs. placebo): **Rated by Fink 2019.** Authors note low risk of bias (however not rated specifically for harm outcomes), unknown consistency (1 study), highly imprecise, direct, reporting bias undetected.

[u] Atypical femoral fractures (any; vs. no treatment): **Rated by Fink 2019.** Authors note moderate risk of bias, unknown consistency (1 study), highly imprecise, direct, reporting bias undetected. Revised to not rate down for imprecision, as the sample size is large and the entire confidence interval is showing harm.

[v] Non-serious gastrointestinal adverse events: ***serious concerns about risk of bias****:* risk of bias not assessed for harm outcomes, rated down one level due to likely bias in harms ascertainment and reporting; ***no serious concerns about inconsistency, imprecision, indirectness, or other considerations***.

[w] Discontinuation due to adverse events: ***serious concerns about risk of bias****:* risk of bias not assessed for harm outcomes, rated down one level due to likely bias in harms ascertainment and reporting; ***no serious concerns about inconsistency, imprecision, indirectness, or other considerations***.

[x] Any non-serious adverse event: ***serious concerns about risk of bias****:* risk of bias not assessed for harm outcomes, rated down one level due to likely bias in harms ascertainment and reporting; ***serious concerns about inconsistency:*** studies differ in direction of effects (one larger study shows harm while others are close to no effect) and I^2=85%; ***no serious concerns about imprecision, indirectness, or other considerations***.

[y] Influenza-like symptoms: ***serious concerns about risk of bias****:* risk of bias not assessed for harm outcomes, rated down one level due to likely bias in harms ascertainment and reporting; ***some concerns about inconsistency:*** there is no evidence of consistency because the analysis includes only one trial; ***serious concerns about imprecision***: small sample size (n=241), number of events cannot reach the optimal information size; ***no serious concerns about indirectness or other considerations***.

[z] Arthritis and arthralgia: ***serious concerns about risk of bias****:* risk of bias not assessed for harm outcomes, rated down one level due to likely bias in harms ascertainment and reporting; ***very serious concerns about inconsistency and imprecision:*** the sample size is not reported and there is no forest plot, however the confidence interval is very wide, including both serious harm and benefit; ***no serious concerns about indirectness or other considerations***.

[aa] Myalgia, cramps, and limb pain: ***serious concerns about risk of bias****:* risk of bias not assessed for harm outcomes, rated down one level due to likely bias in harms ascertainment and reporting; ***very serious concerns about inconsistency and imprecision:*** the sample size is not reported and there is no forest plot, however the confidence interval is very wide, including both serious harm and benefit; ***no serious concerns about indirectness or other considerations***.

KQ3b: What are the harms of pharmacologic treatments to prevent fragility fractures among adults ≥40 years?

2. Risedronate vs. placebo

2.1 GRADE Summary of Findings

Outcome	Studies; sample size	Assumed pop. risk*	Absolute effects (95% CI)	Certainty	What happens?
**Risedronate vs. placebo**					
**Serious adverse events**					
Any serious AE [3, 75]	5 RCT; 7,195	11 per 1000	2.6 fewer per 1000 (10.2 fewer to 5.7 more)	MODERATE[a]	Probably does not increase
Very uncertain: serious GI AEs (all[b]; GI perforations, ulcers bleeds[c]; serious esophageal AE[d]) [76-78], GI cancer[e] [76-78], acute coronary syndrome[f], cerebrovascular death[g], pulmonary embolism[h] [76-78], atrial fibrillation[i] [76-78].<br>No evidence: serious stroke, thromboembolic events [76-78], atypical femoral fractures, or osteonecrosis of the jaw.					
**Non-serious adverse events and discontinuation due to AE**					
Any non-serious AE [80]	6 RCT; 9,575	915 in 1000	45.8 fewer in 1000 (146.4 fewer to 73.2 more)	MODERATE[j]	Probably does not increase
Non-serious GI AE [76-78]	21 RCT; 3,474 events	223 in 1000	5.2 more in 1000 (8.8 fewer to 20.3 more)	MODERATE[k]	
Discontinuation due to AE [3, 75]	5 RCT; 7,159	111 in 1000	1.0 fewer in 1000 (11.8 fewer to 10.9 more)	MODERATE[l]	
Very uncertain: influenza-like symptoms[m] [80], pharyngitis[n] [80], and arthritis and arthralgia[o] [76-78].<br>No evidence: myalgia, cramps, and limb pain [76-78].					

AE=adverse event; GI=gastrointestinal; NR=not reported; NS=not statistically significant; RCT=randomized controlled trial; SR=systematic review

* The control event rate is the median rate in the control group for studies in the analysis. These were extracted directly from the systematic reviews when possible. Otherwise, we extracted these data from the included primary studies when there were ≤5 in the analysis or used the 5 largest studies from larger analyses to calculate the control event rate.

Explanations:

[b] Any serious adverse event: ***serious concerns about risk of bias:*** risk of bias not assessed for harm outcomes, rated down one level due to likely bias in harms ascertainment and reporting; ***no serious concerns about inconsistency, imprecision, indirectness, or other considerations***.

[b] All serious gastrointestinal adverse events: ***serious concerns about risk of bias:*** risk of bias not assessed for harm outcomes, rated down one level due to likely bias in harms ascertainment and reporting; ***serious concerns about indirectness:*** because the number of events is unlikely to reflect the number of people with ≥1 event; ***no serious concerns about inconsistency, imprecision, or other considerations***.

[c] Gastrointestinal perforations, ulcers, and bleeds: ***serious concerns about risk of bias:*** risk of bias not assessed for harm outcomes, rated down one level due to likely bias in harms ascertainment and reporting; ***very serious concerns about inconsistency/imprecision:*** sample size not reported and there is no forest plot, confidence interval is very wide and includes both benefit and harm***; no serious concerns for indirectness or other considerations***.

[d] Serious esophageal adverse events: ***serious concerns about risk of bias:*** risk of bias not assessed for harm outcomes, rated down one level due to likely bias in harms ascertainment and reporting; ***very serious concerns about inconsistency/imprecision:*** sample size not reported and there is no forest plot, confidence interval is very wide and includes both benefit and harm***; no serious concerns for indirectness or other considerations***.

[e] Gastrointestinal cancer: ***serious concerns about risk of bias:*** risk of bias not assessed for harm outcomes, rated down one level due to likely bias in harms ascertainment and reporting; ***serious concerns about inconsistency:*** no evidence of consistency since there is only one trial in the analysis; ***very serious concerns about imprecision:*** the sample size is very small, thus the optimal information size cannot be met***; no serious concerns for indirectness or other considerations***.

[f] Acute coronary syndrome: ***serious concerns about risk of bias:*** risk of bias not assessed for harm outcomes, rated down one level due to likely bias in harms ascertainment and reporting; ***very serious concerns about inconsistency/imprecision:*** sample size not reported and there is no forest plot, confidence interval is very wide and includes both benefit and harm; ***no serious concerns for indirectness or other considerations***.

[g] Cerebrovascular death: ***serious concerns about risk of bias:*** risk of bias not assessed for harm outcomes, rated down one level due to likely bias in harms ascertainment and reporting; ***very serious concerns about inconsistency/imprecision:*** sample size not reported and there is no forest plot, confidence interval is very wide and includes both benefit and harm; ***no serious concerns for indirectness or other considerations***.

[h] Pulmonary embolism: ***serious concerns about risk of bias:*** risk of bias not assessed for harm outcomes, rated down one level due to likely bias in harms ascertainment and reporting; ***very serious concerns about inconsistency/imprecision:*** sample size not reported and there is no forest plot, confidence interval is very wide and includes both benefit and harm; ***no serious concerns for indirectness or other considerations***.

[i] Atrial fibrillation: **Rated by Crandall 2014.** ***Based on independent assessment, serious concerns about risk of bias:*** risk of bias not assessed for harm outcomes, rated down one level due to likely bias in harms ascertainment and reporting; ***serious concerns about inconsistency:*** no evidence of consistency since there is only one trial in the analysis; ***very serious concerns about imprecision:*** the sample size not reported, but the confidence interval is very wide and includes both benefit and harm***; no serious concerns for indirectness or other considerations***.

[j] Any non-serious adverse event: ***serious concerns about risk of bias:*** risk of bias not assessed for harm outcomes, rated down one level due to likely bias in harms ascertainment and reporting; ***no serious concerns about inconsistency, imprecision, indirectness, or other considerations***.

[k] Non-serious gastrointestinal adverse events: ***serious concerns about risk of bias:*** risk of bias not assessed for harm outcomes, rated down one level due to likely bias in harms ascertainment and reporting; ***no serious concerns about inconsistency, imprecision, indirectness, or other considerations***.

[l] Discontinuation due to adverse events: ***serious concerns about risk of bias:*** risk of bias not assessed for harm outcomes, rated down one level due to likely bias in harms ascertainment and reporting; ***no serious concerns about inconsistency***: limited evidence of consistency because the evidence is dominated by one large trial, but consistent with results for alendronate; ***no serious concerns about imprecision, indirectness, or other considerations***.

[m] Influenza-like symptoms: ***serious concerns about risk of bias:*** risk of bias not assessed for harm outcomes, rated down one level due to likely bias in harms ascertainment and reporting; ***some concerns about inconsistency:*** there is no evidence of consistency because the analysis includes only one trial; ***very serious concerns about imprecision***: small sample size (n=284), the optimal information size is not met; ***no serious concerns about indirectness or other considerations***.

[n] Pharyngitis: ***serious concerns about risk of bias:*** risk of bias not assessed for harm outcomes, rated down one level due to likely bias in harms ascertainment and reporting; ***some concerns about inconsistency:*** there is no evidence of consistency because the analysis includes only one trial; ***very serious concerns about imprecision***: small sample size (n=284), the optimal information size is not met; ***no serious concerns about indirectness or other considerations***.

[o] Arthritis and arthralgia: ***serious concerns about risk of bias:*** risk of bias not assessed for harm outcomes, rated down one level due to likely bias in harms ascertainment and reporting; ***very serious concerns about inconsistency/imprecision:*** the sample size is not reported and there is no forest plot, however the confidence interval is very wide, including benefit and harm; ***no serious concerns about indirectness or other considerations***.

3. Zoledronic acid vs. placebo

3.1 GRADE Summary of Findings

Outcome	Studies; sample size	Assumed pop. risk*	Absolute effects (95% CI)	Certainty	What happens?
**Zoledronic acid vs. placebo**					
**Serious adverse events**					
Any serious AE [3, 75]	3 RCT; 1,950	114 in 1000	0.9 fewer in 1000 (19.8 fewer to 21.8 more)	MODERATE[a]	Probably does not increase
Acute coronary syndrome [76-78]	2 RCT; NR	NR	Cannot be calculated; NS difference	LOW[b]	May not increase
Serious stroke [76-78]	2 RCT; NR	NR	Cannot be calculated; NS difference	LOW[c]	
Very uncertain: cerebrovascular death[d] [76-78], atrial fibrillation [76-78][e], atypical femoral fractures[f] [76-78], osteonecrosis of the jaw[g] [76-78]. No evidence: serious GI AE (any; GI perforations, ulcers, bleeds; serious esophageal AE), GI cancer, pulmonary embolism, thromboembolic events.					
**Non-serious adverse events and discontinuation due to AE**					
Any non-serious AE [80]	6 RCT; 9,575	915 in 1000	51.8 more per 1000 (no difference to 112.2 more)	MODERATE[h]	Probably increases
Pyrexia [80]	5 RCT; 11,823	38 in 1000	127.7 more in 1000 (34.6 more to 337.4 more)	MODERATE[i]	
Headache [80]	4 RCT; 9,712	53 in 1000	60.4 more in 1000 (19.1 more to 126.7 more)	MODERATE[j]	
Influenza-like symptoms [76-78]	5 RCT; 10,695	44 in 1000	142.5 more in 1000 (105.5 more to 188.4 more)	MODERATE[k]	
Arthritis and arthralgia [76-78]	6 RCT; 11,171	145 in 1000	178.5 more in 1000 (137.4 more to 224.1 more)	MODERATE[l]	
Myalgia [76-78]	5 RCT; 11,065	17 in 1000	70.7 more in 1000 (54.6 more to 90.8 more)	MODERATE[m]	
Arthralgia, myalgia, pyrexia, chills, & influenza-like symptoms [76-78]	6 RCT; 11,676	219 in 1000	422.8 more in 1000 (398.6 more to 446.3 more)	LOW[n]	May increase
Chills [80]	2 RCT; 799	12 in 1000	33.7 more in 1000 (3.0 more to 127.2 more)	LOW[o]	
Non-serious GI AE [76-78]	3 RCT; 840	79 in 1000	30.9 more in 1000 (11.8 fewer to 97.6 more)	LOW[p]	May not increase
Very uncertain: discontinuation due to AE[q] [3, 75].					

AE=adverse event; GI=gastrointestinal; MI=myocardial infarction; NR=not reported; NS=not statistically significant; RCT=randomized controlled trial; SR=systematic review

* The control event rate is the median rate in the control group for studies in the analysis. These were extracted directly from the systematic reviews when possible. Otherwise, we extracted these data from the included primary studies when there were ≤5 in the analysis or used the 5 largest studies from larger analyses to calculate the control event rate.

Explanations:

[a] Any serious adverse event: ***serious concerns about risk of bias:*** risk of bias not assessed for harm outcomes, rated down one level due to likely bias in harms ascertainment and reporting; ***no serious concerns about inconsistency, imprecision, indirectness, or other considerations***.

[b] Acute coronary syndrome: ***serious concerns about risk of bias:*** risk of bias not assessed for harm outcomes, rated down one level due to likely bias in harms ascertainment and reporting; ***serious concerns about inconsistency/imprecision:*** the sample size is not reported and there is no forest plot, but the confidence interval includes benefit and no effect; ***no serious concerns for indirectness or other considerations***.

[c] Serious stroke: ***serious concerns about risk of bias:*** risk of bias not assessed for harm outcomes, rated down one level due to likely bias in harms ascertainment and reporting; ***serious concerns about inconsistency/imprecision:*** the sample

size is not reported and there is no forest plot, but the confidence interval includes harm and no effect; ***no serious concerns for indirectness or other considerations***.

[d] Cerebrovascular death: ***serious concerns about risk of bias:*** risk of bias not assessed for harm outcomes, rated down one level due to likely bias in harms ascertainment and reporting; ***very serious concerns about inconsistency/imprecision:*** the sample size is not reported and there is no forest plot, but the confidence interval is wide and includes benefit and harm; ***no serious concerns for indirectness or other considerations***.

[e] Atrial fibrillation: **Rated by Crandall 2014.** Reasoning not provided, but there are likely to be concerns about risk of bias, and the analysis relies on only two trials (likely small).

[f] Atypical femoral fractures: **Rated by Fink 2019.** No explanation provided but there were no events reported in the one included trial. Probable concerns about risk of bias, lack of evidence of consistency, and a small sample size to assess a rare event (<4,000).

[g] Osteonecrosis of the jaw: **Rated by Fink 2019.** No explanation provided but there were no events reported in the one included trial. Probable concerns about risk of bias, lack of evidence of consistency, and a small sample size to assess a rare event (<4,000).

[h] Any non-serious adverse event: ***serious concerns about risk of bias:*** risk of bias not assessed for harm outcomes, rated down one level due to likely bias in harms ascertainment and reporting; ***no serious concerns about inconsistency:*** the findings are heterogeneous in terms of effect size, but the direction of effect is homogeneous across studies; ***no serious concerns about imprecision, indirectness, or other considerations***.

[i] Pyrexia: ***serious concerns about risk of bias:*** risk of bias not assessed for harm outcomes, rated down one level due to likely bias in harms reporting; ***no serious concerns about inconsistency, imprecision, indirectness, or other considerations***.

[j] Headache: ***serious concerns about risk of bias:*** risk of bias not assessed for harm outcomes, rated down one level due to likely bias in harms ascertainment and reporting; ***no serious concerns about inconsistency, imprecision, indirectness, or other considerations***.

[k] Influenza-like symptoms: **Rated by Crandall 2014.** ***Based on independent rating, serious concerns about risk of bias:*** risk of bias not assessed for harm outcomes, rated down one level due to likely bias in harms ascertainment and reporting; ***no serious concerns about inconsistency, imprecision, indirectness, or other considerations***.

[l] Arthritis and arthralgia: ***serious concerns about risk of bias:*** risk of bias not assessed for harm outcomes, rated down one level due to likely bias in harms ascertainment and reporting; ***no serious concerns about inconsistency, imprecision, indirectness, or other considerations***.

[m] Myalgia: ***serious concerns about risk of bias:*** risk of bias not assessed for harm outcomes, rated down one level due to likely bias in harms ascertainment and reporting; ***no serious concerns about inconsistency, imprecision, indirectness, or other considerations***.

[n] Composite of arthralgia, myalgia, pyrexia, chills, and influenza-like symptoms: **Rated by Crandall 2014.** ***Based on independent rating, serious concerns about risk of bias:*** risk of bias not assessed for harm outcomes, rated down one

level due to likely bias in harms ascertainment and reporting; ***serious concerns about indirectness:*** composite outcome that is unlikely to represent the number of people with ≥1 event; ***no serious concerns about inconsistency, imprecision, or other considerations***.

[o] Chills: ***serious concerns about risk of bias:*** risk of bias not assessed for harm outcomes, rated down one level due to likely bias in harms ascertainment and reporting; ***serious concerns about inconsistency/imprecision:*** there is no forest plot or indication of heterogeneity, but the sample size is small (<4000 for a rare event); ***no serious concerns for indirectness or other considerations***.

[p] Non-serious gastrointestinal adverse events: ***serious concerns about risk of bias:*** risk of bias not assessed for harm outcomes, rated down one level due to likely bias in harms ascertainment and reporting; ***serious concerns about inconsistency/imprecision:*** forest plot not provided, the number of events does not meet the optimal information size (n=83); ***no serious concerns about indirectness or other considerations***.

[q] Discontinuation due to adverse events: ***serious concerns about risk of bias:*** risk of bias not assessed for harm outcomes, rated down one level due to likely bias in harms ascertainment and reporting; ***some concerns about inconsistency:*** lack of evidence of consistency since there is only one trial in the analysis; ***serious concerns about imprecision:*** the sample size is small for a rare event (<4,000); ***no serious concerns for indirectness or other considerations***.

4. Bisphosphonates vs. placebo or no treatment

4.1 GRADE Summary of Findings

Outcome	Studies; sample size	Assumed pop. risk[*]	Absolute effects (95% CI)	Certainty	What happens?
**Bisphosphonate vs. placebo or no treatment**					
**Serious adverse events**					
Atypical femoral fracture (any, with long-term treatment, >3 years) [74]	1 cohort; ~2.8 mill	0.3 in 1000[†]	11 (7 to 14) in 10,000 in-years	LOW[a]	May increase
	1 case-control; 1,368		NA; OR 93 (66 to 132) for >5 years		
	1 case-control; 290		NA; OR 25.65 (10.74 to 61.28)		
Atypical femoral fracture (subtrochanteric) [76-78]	3 RCT; NR	0.3 in 1000[†]	1.0 more in 1000 (2.6 fewer to 41.1 more)	LOW[b]	
	1 SR of 11 observational; NR		0.2 more in 1000 (0.1 more to 0.4 more)		
	Pooled: safety databases; NR		1.1 more in 1000 (0.7 more to 1.5 more)		
Osteonecrosis of the jaw [76-78]	Case series, SRs; NR	NR	Inconsistent, 0.3 to 43.0 in 1000	LOW[c]	
Stroke [81]	2 RCT; 9,825	33 in 1000	2.0 more in 1000 (5.9 fewer to 11.6 more)	MODERATE[d]	Probably does not increase
Myocardial infarction [81]	5 RCT; 10,4040	12 in 1000	2.2 fewer in 1000 (5.2 fewer to 2.0 more)	MODERATE[e]	
Nonfatal stroke, MI, death - vascular cause [81]	12 RCT; 16,888	67 in 1000	3.4 fewer in 1000 (8.7 fewer to 3.4 more)	LOW[f]	May not increase
Cardiovascular mortality [81]	5 RCT; 10,165	22 in 1000	2.6 fewer in 1000 (8.4 fewer to 5.1 more)	LOW[g]	
Very uncertain: esophageal cancer[h] [76-78] and atrial fibrillation[i] [76-78].<br>No evidence: effect of long-term bisphosphonates (>3 years) on the risk of osteonecrosis of the jaw.					

AE=adverse event; GI=gastrointestinal; MI=myocardial infarction; NR=not reported; NS=not statistically significant; RCT=randomized controlled trial; SR=systematic review

[*] The control event rate is the median rate in the control group for studies in the analysis. These were extracted directly from the systematic reviews when possible. Otherwise, we extracted these data from the included primary studies when there were ≤5 in the analysis or used the 5 largest studies from larger analyses to calculate the control event rate.

KQ3b: What are the harms of pharmacologic treatments to prevent fragility fractures among adults ≥40 years?

Explanations:

[a] Atypical femoral fracture (long term bisphosphonates vs. no treatment): **Rated by Fink 2019.** Authors note moderate risk of bias, precise, consistent, direct, reporting bias undetected, large magnitude of effect (+1).

[b] Atypical femoral fracture (subtrochanteric): **Rated by Crandall 2014.** Evidence is inconsistent and dominated by observational studies.

[c] Osteonecrosis of the jaw (bisphosphonate vs. placebo): **Rated by Crandall 2014.** Evidence is inconsistent and dominated by observational studies.

[d] Stroke: ***no serious concerns about risk of bias****:* authors note no serious risk of bias concerns for the included studies; ***some concerns about imprecision:*** sample size is large but the confidence interval includes harm and no effect; ***no serious concerns about inconsistency, indirectness, or other considerations***.

[e] Myocardial infarction ***no serious concerns about risk of bias****:* authors note no serious risk of bias concerns for the included studies; ***some concerns about imprecision:*** sample size is large but the confidence interval includes harm and no effect; ***no serious concerns about inconsistency, indirectness, or other considerations***.

[f] Composite of nonfatal stroke, myocardial infarction, death from vascular cause: ***some concerns about risk of bias****:* authors note that 5 of 12 studies are at high or unclear risk of bias***; serious concerns about indirectness:*** composite outcome which does not represent the number of participants with ≥1 event; ***no serious concerns about inconsistency, imprecision, indirectness, or other considerations***.

[g] Cardiovascular mortality: ***some concerns about risk of bias****:* authors note that 2 of 5 studies are at high or unclear risk of bias; ***some concerns about imprecision:*** sample size is large but the confidence interval includes benefit and no effect; ***no serious concerns about inconsistency, indirectness, or other considerations***.

[h] Esophageal cancer: **Rated by Crandall 2014.** Reasoning not reported but the evidence is inconsistent and dependent on observational studies.

[i] Atrial fibrillation: **Rated by Viswanathan 2018.** Authors note fair study quality, inconsistent evidence dominated by one study per drug (no evidence of consistency), imprecise despite large sample size, no evidence of reporting bias.

KQ3b: What are the harms of pharmacologic treatments to prevent fragility fractures among adults ≥40 years?

5. Denosumab vs. placebo

5.1 GRADE Summary of Findings

Outcome	Studies; sample size	Assumed pop. risk*	Absolute effects (95% CI)	Certainty	What happens?
**Denosumab vs. placebo**					
**Serious adverse events**					
Any serious AE [3, 75]	4 RCT; 8,663	81 per 1000	9.8 more per 1000 (10.0 fewer to 35.2 more)	LOW[a]	May not increase
Serious cardiac events [76-78]	3 RCT; NR	NR	Cannot be calculated; NS difference	LOW[b]	
Stroke [82]	2 RCT; 7,733	NR	Cannot be calculated; NS difference	LOW[c]	
Cardiovascular death + MI + stroke [83]	4 RCT; 9,066	NR	Cannot be calculated; NS difference	LOW[d]	
Cardiovascular death + MI + stroke + heart failure [83]	4 RCT; 9,066	NR	Cannot be calculated; NS difference	LOW[e]	
Very uncertain: serious infections[f] [3, 75], venous thromboembolism[g] [82]; composite of stroke, atrial fibrillation, heart failure, coronary artery disease[h] [83]; atrial fibrillation[i] [76-78], atypical femoral fractures[j] [74, 82], and osteonecrosis of the jaw[k] [74, 82].<br>No evidence: serious GI AE (any; GI perforations, ulcers, bleeds; serious esophageal) [76-78], GI cancer [76-78], thromboembolic events [76-78], cardiac death [76-78].					
**Non-serious adverse events and discontinuation due to AE**					
Non-serious GI AE [76-78]	3 RCT; 8,454	105 in 1000	64.5 more in 1000 (26.4 more to 113.3 more)	MODERATE[l]	Probably increases
Rash or eczema [3, 75]	3 RCT; 8,454	17 in 1000	15.8 more in 1000 (7.6 more to 27.0 more)	MODERATE[m]	
Infections (any) [76-78]	4 RCT; 8,691	7 in 1000	1.8 more in 1000 (0.1 more to 4.0 more)	MODERATE[n]	
Eczema [3, 75]	1 RCT; 7,762	17 in 1000	13.8 more in 1000 (5.8 more to 24.5 more)	LOW[o]	May increase
Any non-serious AE [82]	5 RCT; 9,201	907 in 1000	No difference in 1000 (9.1 fewer to 9.1 more)	MODERATE[p]	Probably does not increase
Discontinuation due to AE [3, 75]	3 RCT; 8,451	21 in 1000	Cannot be calculated; NS difference	LOW[q]	May not increase
Very uncertain: arthralgia[r] [84], injection-site reactions[s] [3, 75], and rash[t] [3, 75].<br>No evidence: influenza-like symptoms.					
**Rebound fractures with discontinuation (discontinuation of denosumab vs. discontinuation of placebo)**					
Very uncertain: non-vertebral fractures[u] [85], clinical vertebral fractures[v] [85], and multiple clinical vertebral fractures[w] [85].<br>No evidence: hip fracture.					

AE=adverse event; GI=gastrointestinal; MI=myocardial infarction; NR=not reported; NS=not statistically significant; RCT=randomized controlled trial; SR=systematic review

* The control event rate is the median rate in the control group for studies in the analysis. These were extracted directly from the systematic reviews when possible. Otherwise, we extracted these data from the included primary studies when there were ≤5 in the analysis or used the 5 largest studies from larger analyses to calculate the control event rate.

Explanations:

[a] Any serious adverse event: **Rated by Viswanathan 2018**. Authors note fair study quality, consistent (but a single large trial dominates the results), imprecision, no evidence of reporting bias.

[b] Serious cardiac events: ***serious concerns about risk of bias:*** risk of bias not assessed for harm outcomes, rated down one level due to likely bias in harms ascertainment and reporting; ***some concerns for inconsistency/imprecision:*** limited information to judge (no sample size), but seems relatively precise; ***no serious concerns for indirectness or other considerations***.

[c] Stroke: ***serious concerns about risk of bias:*** risk of bias not assessed for harm outcomes, rated down one level due to likely bias in harms ascertainment and reporting; ***some concerns for inconsistency:*** limited evidence of consistency since there are only two trials in the analysis; ***some concerns for imprecision:*** the sample size is large but difference may be somewhat imprecise; ***no serious concerns for indirectness or other considerations***.

[d] Composite of cardiovascular death, myocardial infarction, heart failure: ***serious concerns about risk of bias****:* risk of bias not assessed for harm outcomes, rated down one level due to likely bias in harms ascertainment and reporting; ***serious concerns about indirectness:*** the composite outcome is not representative of the number of people with ≥1 event; ***no serious concerns for inconsistency, imprecision, or other considerations***.

[e] Composite of cardiovascular death, myocardial infarction, stroke, heart failure: ***serious concerns about risk of bias****:* risk of bias not assessed for harm outcomes, rated down one level due to likely bias in harms ascertainment and reporting; ***serious concerns about indirectness:*** the composite outcome is not representative of the number of people with ≥1 event; ***no serious concerns for inconsistency, imprecision, or other considerations***.

[f] Serious infections: **Rated by Viswanathan 2018**. Authors note fair study quality, consistent (but a single large trial dominates the results), imprecision (despite large sample size), no evidence of reporting bias.

[g] Venous thromboembolism: ***serious concerns about risk of bias****:* risk of bias not assessed for harm outcomes, rated down one level due to likely bias in harms ascertainment and reporting; ***some concerns for inconsistency:*** limited evidence of consistency since there are only two trials in the analysis; ***serious concerns for imprecision:*** the sample size is inadequate for assessment of a rare event (<4,000); ***no serious concerns for indirectness or other considerations***.

[h] Composite of stroke, atrial fibrillation, heart failure, coronary artery disease: ***serious concerns about risk of bias****:* risk of bias not assessed for harm outcomes, rated down one level due to likely bias in harms ascertainment and reporting; ***serious concerns about inconsistency/imprecision:*** sample size is large, but confidence interval includes both harm and no effect; ***serious concerns about indirectness:*** the composite outcome is not representative of the number of people with ≥1 event; ***no serious concerns for other considerations***.

[i] Atrial fibrillation: ***serious concerns about risk of bias****:* risk of bias not assessed for harm outcomes, rated down one level due to likely bias in harms ascertainment and reporting; ***some concerns for inconsistency:*** limited evidence of consistency since there is only one trial in the analysis; ***very serious concerns for imprecision:*** sample size not provided, but confidence interval is very wide, including both benefit and harm; ***no serious concerns for indirectness or other considerations***.

[j] Atypical femoral fractures: ***serious concerns about risk of bias****:* risk of bias not assessed for harm outcomes, rated down one level due to likely bias in harms ascertainment and reporting; ***some concerns for inconsistency:*** cannot be adequately assessed because there were no events reported in any trial; ***serious concerns for imprecision:*** the sample size is inadequate as no events were reported in either group; ***no serious concerns for indirectness or other considerations***.

[k] Osteonecrosis of the jaw: ***serious concerns about risk of bias****:* risk of bias not assessed for harm outcomes, rated down one level due to likely bias in harms ascertainment and reporting; ***some concerns for inconsistency:*** cannot be adequately assessed because there were no events reported in any trial; ***serious concerns for imprecision:*** the sample size is inadequate as no events were reported in either group; ***no serious concerns for indirectness or other considerations***.

[l] Non-serious gastrointestinal adverse events: ***serious concerns about risk of bias****:* risk of bias not assessed for harm outcomes, rated down one level due to likely bias in harms ascertainment and reporting; ***no serious concerns for inconsistency, imprecision, indirectness, or other considerations***.

[m] Rash or eczema: **Rated by Crandall 2014 as high certainty.** ***Independent reassessment indicates serious concerns about risk of bias****:* risk of bias not assessed for harm outcomes, rated down one level due to likely bias in harms ascertainment and reporting; ***no serious concerns for inconsistency, imprecision, indirectness, or other considerations.***

[n] Infections: **Rated by Crandall 2014.** ***Independent reassessment indicates serious concerns about risk of bias****:* risk of bias not assessed for harm outcomes, rated down one level due to likely bias in harms ascertainment and reporting; ***no serious concerns for inconsistency, imprecision, indirectness, or other considerations***.

[o] Eczema: ***Serious concerns about risk of bias****:* risk of bias not assessed for harm outcomes, rated down one level due to likely bias in harms ascertainment and reporting; ***some concerns about inconsistency:*** no evidence of consistency since there is only one study in the analysis; ***no serious concerns for imprecision, indirectness, or other considerations***.

[p] Any non-serious adverse event: ***serious concerns about risk of bias****:* risk of bias not assessed for harm outcomes, rated down one level due to likely bias in harms ascertainment and reporting; ***no serious concerns for inconsistency, imprecision, indirectness, or other considerations***.

[q] Discontinuation due to adverse events: ***serious concerns about risk of bias****:* risk of bias not assessed for harm outcomes, rated down one level due to likely bias in harms ascertainment and reporting; ***serious concerns for imprecision:*** the sample size is adequate, but the confidence interval includes both no effect and harm; ***no serious concerns for inconsistency, indirectness, or other considerations***.

[r] Arthralgia: ***serious concerns about risk of bias****:* risk of bias not assessed for harm outcomes, rated down one level due to likely bias in harms ascertainment and reporting; ***some concerns about inconsistency:*** no evidence of consistency since there is only one study in the analysis; ***serious concerns about imprecision:*** sample size not provided, confidence interval may be wide (authors only report "not statistically significant"); ***no serious concerns for indirectness or other considerations***.

[s] Injection site reactions: **Rated by Crandall 2014 as high certainty.** ***Independent reassessment indicates serious concerns about risk of bias****:* risk of bias not assessed for harm outcomes, rated down one level due to likely bias in harms ascertainment and reporting; ***some concerns about inconsistency:*** no evidence of consistency since there is only one study in the analysis; ***serious concerns about imprecision:*** the number of events does not meet the optimal information size (n=2), and the confidence interval is very wide, including benefit and harm; ***no serious concerns for indirectness or other considerations***.

[t] Rash: ***Serious concerns about risk of bias****:* risk of bias not assessed for harm outcomes, rated down one level due to likely bias in harms ascertainment and reporting; ***some concerns about inconsistency:*** no evidence of consistency since there is only one study in the analysis; ***serious concerns about imprecision:*** the sample size is small for assessment of a rare event (<4,000); ***no serious concerns for indirectness or other considerations***.

[u] Nonvertebral fractures (denosumab discontinuation vs. placebo discontinuation): ***serious concerns about risk of bias****:* the one included trial is non-randomized; ***some concerns for inconsistency:*** there is no evidence of consistency; ***serious concerns for imprecision:*** the number of events does not meet the optimal information size (<300); ***serious concerns for indirectness:*** the outcome may include non-clinical fractures, the follow-up is short (median 2-6 months), and the fractures cannot be attributed to rebound effects with confidence.

KQ3b: What are the harms of pharmacologic treatments to prevent fragility fractures among adults ≥40 years?

[v] Clinical vertebral fractures (denosumab discontinuation vs. placebo discontinuation): ***serious concerns about risk of bias****:* the one included trial is non-randomized; ***some concerns for inconsistency:*** there is no evidence of consistency; ***serious concerns for imprecision:*** the number of events does not meet the optimal information size (<300); ***serious concerns for indirectness:*** the outcome includes non-clinical fractures (though number with clinically recognized fractures is provided), the follow-up is short (median 2-6 months), and the fractures cannot be attributed to rebound effects with confidence.

[w] Multiple vertebral fractures (denosumab discontinuation vs. placebo discontinuation): ***serious concerns about risk of bias****:* the one included trial is non-randomized; ***some concerns for inconsistency:*** there is no evidence of consistency; ***serious concerns for imprecision:*** the number of events does not meet the optimal information size (<300); ***serious concerns for indirectness:*** the outcome includes non-clinical fractures (though number with clinically recognized fractures is provided), the follow-up is short (median 2-6 months), and the fractures cannot be attributed to rebound effects with confidence.

6. Denosumab discontinuation vs. non-discontinuation
6.1 GRADE Summary of Findings

Outcome	Studies; sample size	Assumed Risk in those remaining on denosumab*	Absolute effects (95% CI)	Certainty	What happens?
Rebound fractures (i.e. multiple vertebral fractures) [86]	1 cohort; 3,110	0.01 per 1000	0.07 more per 1000	VERY LOW[a]	Uncertain

Explanation:

[a] Rebound fractures: ***some concerns about risk of bias***: the study did not control for confounders in their analysis for this (rare) outcomes, but the groups were very similar across a large number of variables; ***some concerns for inconsistency:*** there is no evidence of consistency; ***some concerns about imprecision***: the sample size was likely inadequate for the low (<1%) incidence of the outcome; ***some concerns for indirectness***; most (>90%) of the participants were on second or greater line therapy and a large proportion (>40%) had previous fractures

KQ4: For patients ≥40 years, what is the acceptability of screening and/or initiating treatment to prevent fragility fractures when considering the possible benefits and harms from screening and/or treatment?

EVIDENCE SUMMARY FOR KQ4 ON THE ACCEPTABILITY OF SCREENING AND/OR TREATMENT

1. GRADE Summary of Findings

Studies; sample size	Findings	Certainty*	What does the evidence say?
**Acceptance of screening**			
Females 50-65 y<br>1 observational; 258	– Females (57% previously screened; low risk based on age) had moderate-to-strong intentions to be screened (mean (SD) intention score 3.74 (0.96)/5) [87].<br>– Reading information on the benefits (2 fewer hip fractures in 1000/10 y or 'very few') and harms (osteonecrosis of the jaw – 1-10/1000 or 'very few', atypical fractures – 5/1000 or 'very few', overdiagnosis – incidence disease rate exceeding important outcomes), was not associated with any important change in the intention to accept screening [87].<br>– There was no difference by subgroups of patients defined by previous screening or worry about health [87].	LOW[a]	Females aged 50-65 years (low risk) may have a high intention to be screened, and this intention may not be changed after reading a 1-page decision support sheet (1 study, n=258) [87].
**Acceptance of treatment with information**			
Adults (predominantly female) ≥50 y, mean 63-73 y<br>3 observational, 2 RCT; 1,010	– In two studies (n=593) [88, 89], 19 to 39% of patients who were aware of their fracture risk and received information on benefits (and harms in one study)[88] were willing to initiate treatment.<br>– In one small study (n=30) [90], 57% of females unaware of their fracture risk were acceptors or cautious acceptors of bisphosphonate treatment. More females (80%) would accept treatment of some form, with an indication females are concerned about the harms of bisphosphonates.<br>– In two studies (n=387; ~50% at high risk of fracture or with osteoporosis) [91, 92], at various levels of efficacy (5% to 50%) women preferred treatment over no treatment, with significant variability in the strength of those preferences.<br>– One study (n=267) [92] showed that females and those with osteoporosis had a stronger preference for being treated, but not those with a prior fracture.	LOW[b]	Patients' preference for treatment vs. no treatment may be highly variable (3 studies, n=317) [90-92]. After receiving information on their personal fracture risk, relatively few (19 to 39%) patients may be willing to accept treatment (2 studies, n=593) [88, 89].
**Acceptance of treatment with decision aids**			
Postmenopausal females ≥45 y, mean 64-69 y<br>4 observational (5 reports); ~324	– In two studies (n~240) [93-95], postmenopausal women with osteoporosis or osteopenia who were aware of their fracture risk were provided decision aids outlining potential benefits and harms treatment. Overall, few (20.2%) decided to initiate treatment, and many were undecided. Of those with prior fracture or at high risk (i.e. common treatment thresholds of 20% and 3% for clinical and hip fracture) 32-45% accepted treatment [93, 95]. In one study, only 5.3% were taking anti-osteoporosis drugs at 6 months follow-up.[94]<br>– In two studies (n=84) [96, 97] clinicians used the Osteoporosis Choice decision aid during a clinical encounter, after which less than half (41 to 44%) decided to initiate treatment. Most (>80%) of these patients had actually initiated treatment at follow-up.	MODERATE[c]	Few (5-20%) postmenopausal females with osteoporosis or osteopenia who read decision aids and are aware of their fracture risk are willing to initiate treatment (2 studies, n~240) [93-95]. Somewhat more may be willing to start treatment when the decision aid is used during a clinical encounter (41-44%; 2 studies, n=84) [96, 97] or when they have had a previous fracture or are at higher fracture risk (32-45%; 1 study, n=208). Overall, a minority of postmenopausal females at increased risk for fracture may accept treatment.
**Minimum acceptable benefit of treatment**			
Adults ≥50 y, mean 60-72 y<br>3 observational; 741	– In one study (n=354, 44% female, unaware of fracture risk) [98], 64% of adults required 100 to 1000 fewer hip fractures per 5000 people for a treatment with no major side effects to be acceptable (50 assumed to be 'correct').<br>– Overestimation of benefits did not vary significantly by age, sex, diagnosis of osteoporosis, and use of medications for osteoporosis [98].<br>– In one study (n=267)[92] patients preferred a treatment with higher clinical efficacy and were willing to pay 3689 Yuan (~700 CAD) per annum for 1%	LOW[d]	About two-thirds (64%) of adults ≥50 years may have overly optimistic views of the benefits of treatment (1 study, n=354) [98]; these views may be highly variable (3 studies, n=741) [91, 92, 98]. Patients may require a reduction of 20 to 200 fractures per 1000 to

KQ4: For patients ≥40 years, what is the acceptability of screening and/or initiating treatment to prevent fragility fractures when considering the possible benefits and harms from screening and/or treatment?

Studies; sample size	Findings	Certainty*	What does the evidence say?
	improvement in medication efficacy, but this was variable (~385 to 1250 CAD). In a second study (n=120) [91], patients were willing to pay up to 338 Euro (~500 CAD) for treatment if the fracture risk reduction was at ≥ 12%.		consider 10 y of bisphosphonate treatment acceptable (1 study, n=354) [98].
**Level of risk at which treatment is acceptable**			
Adults (predominantly female) ≥45 y<br>6 observational; 1091	– In one study (n=200) [89], the median (IQR) 5-y risk threshold for oral medication was 50 (25, 70)% for osteoporotic and 50 (30, 75)% for hip fracture. Information on benefits had little impact on these thresholds.<br>– In a second study (n=241) [99] those with osteoporosis or higher estimate of risk were more likely to accept treatment, but 28% to 38% of females would not accept treatment in high risk scenarios (≥3% hip, 12% other fracture).<br>– Two studies (n=445) [88, 97] showed low risk females may be just as willing to accept treatment as higher risk females. In a third study (n=85) [93] acceptance of treatment was higher among those with a high (≥3%) risk of hip fracture (32% vs. 19%, p=0.012) but not a high (≥20%) risk of MOF (47% vs. 23%, p=0.11) compared to those at lower risk.<br>– In one study (n=120) [91], patients at higher fracture risk were willing to accept less effective treatments than those at lower risk.	LOW[e]	Among adults ≥45 years (97% female; aware of personal risk) there is large heterogeneity in the level of risk at which treatment would be considered [88, 89, 91, 93, 97, 99]. Many (19 to 51%) are willing to accept treatment at low levels of fracture risk (5 to 20%), but a large proportion (44 to 68%) of high-risk females (≥3% hip or ≥20% osteoporotic fracture risk; ≥30% in one study) would choose not to be treated (3 studies, n=378) [88, 93, 97].

CAD=Canadian dollars; IQR=interquartile range; MOF=major osteoporotic fracture; RCT=randomized controlled trial; SD=standard deviation; y=years

* When our assessment of the certainty of evidence fell between levels, we assigned the level that best represented our actual certainty.

Explanations:

[a] Acceptance of screening: Some concern about potential for selection bias (24% of the eligible population participated); some concern about lack of evidence of consistency; some concern about indirectness due to the intervention (hypothetical scenario rather than actual decision about being screened); some concern about imprecision due to small sample size (<300).

[b] Acceptance of treatment with information: Serious concern about risk of bias due to potential selection bias in two studies (36 to 66% of eligible population participated in Hudson 2011 and de Bekker-Grob 2008, respectively), unclear participant understanding of the information provided in all studies, and lack of information on harms in Kalluru 2017; serious concern about indirectness due to population (many participants were lower risk and would not be eligible for treatment) and the intervention (hypothetical scenario rather than actual decision about treatment).

[c] Acceptance of treatment with decision aids: Some concern about risk of bias due to lack of controlling (or subgroup analysis) for important confounders (namely baseline risk) in 2 studies; though risk of bias is high in the Smallwood study, this was a minor contributor to the analysis so we did not rate down further; some concern about imprecision with sample size near minimum of 300.

[d] Minimum acceptable benefit of treatment: Serious concern about risk of bias due to potential selection bias in two studies (36 to 66% of eligible population participated in Hudson 2011 and de Bekker-Grob 2008, respectively), unclear participant understanding of the information provided in all studies, and inaccurate information on harms in Hudson 2012 ("no major side effects"); serious concern about indirectness due to population (in the Hudson 2012 and Si 2019 studies were not aware of their own risk) and the intervention (hypothetical scenario rather than actual decision about treatment).

[e] Level of risk at which treatment is acceptable: Serious concern about risk of bias due to potential selection bias in four studies (31 to 66% of eligible population participated in Neuner 2014, Hudson 2011, and de Bekker-Grob 2008, unclear in Billington 2019), unclear participant understanding of the information provided in all studies except those using decision aids (Billington 2019 and Montori 2011), and lack of information on harms in Kalluru 2017; some concern about indirectness due to the intervention (hypothetical scenario rather than actual decision about treatment in all studies except those using decision aids).

REFERENCES

1. Debray TPA, Damen JAAG, Snell KIE, Ensor J, Hooft L, Reitsma JB, et al. A guide to systematic review and meta-analysis of prediction model performance. BMJ. 2017;356.
2. Murad MH, Mustafa RA, Schünemann HJ, Sultan S, Santesso N. Rating the certainty in evidence in the absence of a single estimate of effect. Evid Based Med. 2017;22(3):85-7.
3. Viswanathan M, Reddy S, Berkman N, Cullen K, Middleton JC, Nicholson WK, et al. Screening to prevent osteoporotic fractures: An evidence review for the US Preventive Services Task Force. Rockville, MD: Agency for Healthcare Research and Quality; 2018.
4. Azagra R, Zwart M, Encabo G, Aguye A, Martin-Sanchez JC, Puchol-Ruiz N, et al. Rationale of the Spanish FRAX model in decision-making for predicting osteoporotic fractures: an update of FRIDEX cohort of Spanish women. BMC Musculoskeletal Disorders. 2016;17:262.
5. Bolland MJ, Siu AT, Mason BH, Horne AM, Ames RW, Grey AB, et al. Evaluation of the FRAX and Garvan fracture risk calculators in older women. Journal of bone and mineral research : the official journal of the American Society for Bone and Mineral Research. 2011;26(2):420-7.
6. Crandall CJ, Larson J, LaCroix A, Cauley JA, LeBoff MS, Li W, et al. Predicting Fracture Risk in Younger Postmenopausal Women: Comparison of the Garvan and FRAX Risk Calculators in the Women's Health Initiative Study. Journal of General Internal Medicine. 2019;34(2):235-42.
7. Czerwinski E, Borowy P, Kumorek A, Amarowicz J, Gorkiewicz M, Milert A. Fracture risk prediction in outpatients from Krakow Region using FRAX tool versus fracture risk in 11-year follow-up. Ortopedia, traumatologia, rehabilitacja. 2013;15(6):617-28.
8. Ettinger B, Ensrud KE, Blackwell T, Curtis JR, Lapidus JA, Orwoll ES, et al. Performance of FRAX in a cohort of community-dwelling, ambulatory older men: the Osteoporotic Fractures in Men (MrOS) study. Osteoporosis International. 2013;24(4):1185-93.
9. Goldshtein I, Gerber Y, Ish-Shalom S, Leshno M. Fracture Risk Assessment With FRAX Using Real-World Data in a Population-Based Cohort From Israel. American Journal of Epidemiology. 2018;187(1):94-102.
10. Marques A, Lucas R, Simoes E, Verstappen SMM, Jacobs JWG, da Silva JAP. Do we need bone mineral density to estimate osteoporotic fracture risk? A 10-year prospective multicentre validation study. RMD Open. 2017;3(2):e000509.
11. Pluskiewicz W, Adamczyk P, Czekajlo A, Grzeszczak W, Drozdzowska B. High fracture probability predicts fractures in a 4-year follow-up in women from the RAC-OST-POL study. Osteoporosis international : a journal established as result of cooperation between the European Foundation for Osteoporosis and the National Osteoporosis Foundation of the USA. 2015;26(12):2811-20.
12. Premaor M, Parker RA, Cummings S, Ensrud K, Cauley JA, Lui LY, et al. Predictive value of FRAX for fracture in obese older women. Journal of bone and mineral research : the official journal of the American Society for Bone and Mineral Research. 2013;28(1):188-95.
13. Pressman AR, Lo JC, Chandra M, Ettinger B. Methods for assessing fracture risk prediction models: experience with FRAX in a large integrated health care delivery system. Journal of clinical densitometry : the official journal of the International Society for Clinical Densitometry. 2011;14(4):407-15.
14. Sornay-Rendu E, Munoz F, Delmas PD, Chapurlat RD. The FRAX tool in French women: How well does it describe the real incidence of fracture in the OFELY cohort? Journal of bone and mineral research : the official journal of the American Society for Bone and Mineral Research. 2010;25(10):2101-7.
15. Tamaki J, Iki M, Sato Y, Winzenrieth R, Kajita E, Kagamimori S, et al. Does Trabecular Bone Score (TBS) improve the predictive ability of FRAX for major osteoporotic fractures according to the Japanese Population-Based Osteoporosis (JPOS) cohort study? Journal of Bone & Mineral Metabolism. 2019;37(1):161-70.
16. Yin MT, Shiau S, Rimland D, Gibert CL, Bedimo RJ, Rodriguez-Barradas MC, et al. Fracture Prediction With Modified-FRAX in Older HIV-Infected and Uninfected Men. Journal of Acquired Immune Deficiency Syndromes: JAIDS. 2016;72(5):513-20.
17. Crandall CJ, Schousboe JT, Morin SN, Lix LM, Leslie W. Performance of FRAX and FRAX-Based Treatment Thresholds in Women Aged 40 Years and Older: The Manitoba BMD Registry. Journal of Bone & Mineral Research. 2019;28:28.

18. Fraser LA, Langsetmo L, Berger C, Ioannidis G, Goltzman D, Adachi JD, et al. Fracture prediction and calibration of a Canadian FRAX(R) tool: a population-based report from CaMos. Osteoporosis international : a journal established as result of cooperation between the European Foundation for Osteoporosis and the National Osteoporosis Foundation of the USA. 2011;22(3):829-37.
19. Leslie WD, Majumdar SR, Morin SN, Lix LM, Johansson H, Oden A, et al. FRAX for fracture prediction shorter and longer than 10 years: the Manitoba BMD registry. Osteoporosis International. 2017;28(9):2557-64.
20. Li G, Thabane L, Papaioannou A, Adachi JD. Comparison between frailty index of deficit accumulation and fracture risk assessment tool (FRAX) in prediction of risk of fractures. Bone. 2015;77:107-14.
21. Dagan N, Cohen-Stavi C, Leventer-Roberts M, Balicer RD. External validation and comparison of three prediction tools for risk of osteoporotic fractures using data from population based electronic health records: retrospective cohort study. BMJ. 2017;356:i6755.
22. Desbiens L-C, Mac-Way F, Sidibe A, Beaudoin C, Jean S. Comparison of Fracture Prediction Tools in Individuals Without and With Early Chronic Kidney Disease: A Population-Based Analysis of CARTaGENE. Journal of Bone and Mineral Research. 2020;35(6):1048-57.
23. Holloway KL, Mohebbi M, Betson AG, Hans D, Hyde NK, Brennan-Olsen SL, et al. Prediction of major osteoporotic and hip fractures in Australian men using FRAX scores adjusted with trabecular bone score. Osteoporosis International. 2018;29(1):101-8.
24. Melton LJ, 3rd, Atkinson EJ, Achenbach SJ, Kanis JA, Therneau TM, Johansson H, et al. Potential Extensions of the US FRAX Algorithm. Journal of osteoporosis. 2012;2012:528790.
25. Iki M, Fujita Y, Tamaki J, Kouda K, Yura A, Sato Y, et al. Trabecular bone score may improve FRAX(R) prediction accuracy for major osteoporotic fractures in elderly Japanese men: the Fujiwara-kyo Osteoporosis Risk in Men (FORMEN) Cohort Study. Osteoporosis international : a journal established as result of cooperation between the European Foundation for Osteoporosis and the National Osteoporosis Foundation of the USA. 2015;26(6):1841-8.
26. Tanaka S, Yoshimura N, Kuroda T, Hosoi T, Saito M, Shiraki M. The Fracture and Immobilization Score (FRISC) for risk assessment of osteoporotic fracture and immobilization in postmenopausal women--A joint analysis of the Nagano, Miyama, and Taiji Cohorts. Bone. 2010;47(6):1064-70.
27. Tebe Cordomi C, Del Rio LM, Di Gregorio S, Casas L, Estrada MD, Kotzeva A, et al. Validation of the FRAX predictive model for major osteoporotic fracture in a historical cohort of Spanish women. Journal of clinical densitometry : the official journal of the International Society for Clinical Densitometry. 2013;16(2):231-7.
28. Tremollieres FA, Pouilles JM, Drewniak N, Laparra J, Ribot CA, Dargent-Molina P. Fracture risk prediction using BMD and clinical risk factors in early postmenopausal women: sensitivity of the WHO FRAX tool. Journal of bone and mineral research : the official journal of the American Society for Bone and Mineral Research. 2010;25(5):1002-9.
29. Gourlay ML, Ritter VS, Fine JP, Overman RA, Schousboe JT, Cawthon PM, et al. Comparison of fracture risk assessment tools in older men without prior hip or spine fracture: the MrOS study. Archives of Osteoporosis. 2017;12(1):91.
30. Langsetmo L, Nguyen TV, Nguyen ND, Kovacs CS, Prior JC, Center JR, et al. Independent external validation of nomograms for predicting risk of low-trauma fracture and hip fracture. CMAJ : Canadian Medical Association journal = journal de l'Association medicale canadienne. 2011;183(2):E107-14.
31. Reyes Dominguez AI, Sosa Cabrera N, Saavedra Santana P, de Tejada Romero MJG, Jodar Gimeno E, Sosa Henriquez M. Assessment of the predictive capacity of the garvan calculator of 10 year risk of fracture in a Spanish population. Revista de Osteoporosis y Metabolismo Mineral. 2017;9(2):55-61.
32. Leslie WD, Majumdar SR, Lix LM, Josse RG, Johansson H, Oden A, et al. Direct comparison of FRAX(R) and a simplified fracture risk assessment tool in routine clinical practice: a registry-based cohort study. Osteoporosis international : a journal established as result of cooperation between the European Foundation for Osteoporosis and the National Osteoporosis Foundation of the USA. 2016;27(9):2689-95.
33. Ettinger B, Liu H, Blackwell T, Hoffman AR, Ensrud KE, Orwoll ES. Validation of FRC, a fracture risk assessment tool, in a cohort of older men: the Osteoporotic Fractures in Men (MrOS) Study. Journal of clinical densitometry : the official journal of the International Society for Clinical Densitometry. 2012;15(3):334-42.
34. Lo JC, Pressman AR, Chandra M, Ettinger B. Fracture risk tool validation in an integrated healthcare delivery system. The American journal of managed care. 2011;17(3):188-94.

35. Ascott-Evans BH, Guañabens N, Kivinen S, Stuckey BGA, Magaril CH, Vandormael K, et al. Alendronate Prevents Loss of Bone Density Associated With Discontinuation of Hormone Replacement Therapy: A Randomized Controlled Trial. Archives of internal medicine. 2003;163(7):789-94.
36. Chesnut CH, McClung MR, Ensrud KE, Bell NH, Genant HK, Harris ST, et al. Alendronate treatment of the postmenopausal osteoporotic woman: Effect of multiple dosages on bone mass and bone remodeling. The American Journal of Medicine. 1995;99(2):144-52.
37. Cummings SR, Black DM, Thompson DE, Applegate WB, Barrett-Connor E, Musliner TA, et al. Effect of Alendronate on Risk of Fracture in Women With Low Bone Density but Without Vertebral FracturesResults From the Fracture Intervention Trial. JAMA. 1998;280(24):2077-82.
38. Hosking D, Chilvers CE, Christiansen C, Ravn P, Wasnich R, Ross P, et al. Prevention of bone loss with alendronate in postmenopausal women under 60 years of age. Early Postmenopausal Intervention Cohort Study Group. The New England journal of medicine. 1998;338(8):485-92.
39. Liberman UA, Weiss SR, Bröll J, Minne HW, Quan H, Bell NH, et al. Effect of Oral Alendronate on Bone Mineral Density and the Incidence of Fractures in Postmenopausal Osteoporosis. New England Journal of Medicine. 1995;333(22):1437-44.
40. Pols HAP, Felsenberg D, Hanley DA, Štepán J, Muñoz-Torres M, Wilkin TJ, et al. Multinational, Placebo-Controlled, Randomized Trial of the Effects of Alendronate on Bone Density and Fracture Risk in Postmenopausal Women with Low Bone Mass: Results of the FOSIT Study. Osteoporosis International. 1999;9(5):461-8.
41. Yan Y, Wang W, Zhu H, Li M, Liu J, Luo B, et al. The efficacy and tolerability of once-weekly alendronate 70 mg on bone mineral density and bone turnover markers in postmenopausal Chinese women with osteoporosis. Journal of bone and mineral metabolism [Internet]. 2009; 27(4):[471-8 pp.]. Available from: https://www.cochranelibrary.com/central/doi/10.1002/central/CN-00718986/full

https://link.springer.com/content/pdf/10.1007/s00774-009-0057-7.pdf.
42. Tucci JR, Tonino RP, Emkey RD, Peverly CA, Kher U, Santora AC, 2nd. Effect of three years of oral alendronate treatment in postmenopausal women with osteoporosis. The American journal of medicine. 1996;101(5):488-501.
43. Bell NH, Bilezikian JP, Bone Iii HG, Kaur A, Maragoto A, Santora AC. Alendronate Increases Bone Mass and Reduces Bone Markers in Postmenopausal African-American Women. The Journal of Clinical Endocrinology & Metabolism. 2002;87(6):2792-7.
44. Hosking D, Adami S, Felsenberg D, Andia JC, Valimaki M, Benhamou L, et al. Comparison of change in bone resorption and bone mineral density with once-weekly alendronate and daily risedronate: a randomised, placebo-controlled study. Current medical research and opinion. 2003;19(5):383-94.
45. Donaldson MG, Palermo L, Ensrud KE, Hochberg MC, Schousboe JT, Cummings SR. Effect of alendronate for reducing fracture by FRAX score and femoral neck bone mineral density: the Fracture Intervention Trial. Journal of bone and mineral research : the official journal of the American Society for Bone and Mineral Research. 2012;27(8):1804-10.
46. Hochberg MC, Thompson DE, Black DM, Quandt SA, Cauley J, Geusens P, et al. Effect of alendronate on the age-specific incidence of symptomatic osteoporotic fractures. Journal of bone and mineral research : the official journal of the American Society for Bone and Mineral Research. 2005;20(6):971-6.
47. McClung MR, Lewiecki EM, Cohen SB, Bolognese MA, Woodson GC, Moffett AH, et al. Denosumab in postmenopausal women with low bone mineral density. The New England journal of medicine. 2006;354(8):821-31.
48. Lewiecki EM, Miller PD, McClung MR, Cohen SB, Bolognese MA, Liu Y, et al. Two-year treatment with denosumab (AMG 162) in a randomized phase 2 study of postmenopausal women with low BMD. Journal of bone and mineral research : the official journal of the American Society for Bone and Mineral Research. 2007;22(12):1832-41.
49. Prior JC, Langsetmo L, Lentle BC, Berger C, Goltzman D, Kovacs CS, et al. Ten-year incident osteoporosis-related fractures in the population-based Canadian Multicentre Osteoporosis Study — Comparing site and age-specific risks in women and men. Bone. 2015;71:237-43.
50. Li Y, Zhang Z, Deng X, Chen L. Efficacy and safety of risedronate sodium in treatment of postmenopausal osteoporosis. Hua zhong ke ji da xue xue bao Yi xue Ying De wen ban [Journal of Huazhong University of Science and Technology Medical sciences] [Internet]. 2005; 25(5):[527-9 pp.]. Available from: https://www.cochranelibrary.com/central/doi/10.1002/central/CN-00749866/full

https://link.springer.com/content/pdf/10.1007/BF02896007.pdf.
51. McClung MR, Geusens P, Miller PD, Zippel H, Bensen WG, Roux C, et al. Effect of Risedronate on the Risk of Hip Fracture in Elderly Women. New England Journal of Medicine. 2001;344(5):333-40.
52. Mortensen L, Charles P, Bekker PJ, Digennaro J, Johnston CC, Jr. Risedronate increases bone mass in an early postmenopausal population: two years of treatment plus one year of follow-up. The Journal of clinical endocrinology and metabolism. 1998;83(2):396-402.
53. Välimäiki MJ, Farrerons-Minguella J, Halse J, Kröger H, Maroni M, Mulder H, et al. Effects of risedronate 5 mg/d on bone mineral density and bone turnover markers in late-postmenopausal women with osteopenia: A multinational, 24-month, randomized, double-blind, placebo-controlled, parallel-group, phase III trial. Clinical Therapeutics. 2007;29(9):1937-49.
54. Fogelman I, Ribot C, Smith R, Ethgen D, Sod E, Reginster JY. Risedronate reverses bone loss in postmenopausal women with low bone mass: results from a multinational, double-blind, placebo-controlled trial. BMD-MN Study Group. The Journal of clinical endocrinology and metabolism. 2000;85(5):1895-900.
55. Hooper MJ, Ebeling PR, Roberts AP, Graham JJ, Nicholson GC, D'Emden M, et al. Risedronate prevents bone loss in early postmenopausal women: a prospective randomized, placebo-controlled trial. Climacteric : the journal of the International Menopause Society. 2005;8(3):251-62.
56. Grey A, Bolland M, Mihov B, Wong S, Horne A, Gamble G, et al. Duration of antiresorptive effects of low-dose zoledronate in osteopenic postmenopausal women: A randomized, placebo-controlled trial. Journal of Bone and Mineral Research. 2014;29(1):166-72.
57. Grey A, Bolland MJ, Wattie D, Horne A, Gamble G, Reid IR. The antiresorptive effects of a single dose of zoledronate persist for two years: A randomized, placebo-controlled trial in osteopenic postmenopausal women. Obstetrical and Gynecological Survey. 2009;64(7):462-3.
58. Reid IR, Horne AM, Mihov B, Stewart A, Garratt E, Wong S, et al. Fracture Prevention with Zoledronate in Older Women with Osteopenia. The New England journal of medicine. 2018;379(25):2407-16.
59. Grey A, Bolland M, Wong S, Horne A, Gamble G, Reid IR. Low-dose zoledronate in osteopenic postmenopausal women: A randomized controlled trial. Journal of Clinical Endocrinology and Metabolism. 2012;97(1):286-92.
60. McClung M, Miller P, Recknor C, Mesenbrink P, Bucci-Rechtweg C, Benhamou CL. Zoledronic acid for the prevention of bone loss in postmenopausal women with low bone mass: a randomized controlled trial. Obstetrics & Gynecology. 2009;114(5):999-1007.
61. Reid IR, Brown JP, Burckhardt P, Horowitz Z, Richardson P, Trechsel U, et al. Intravenous Zoledronic Acid in Postmenopausal Women with Low Bone Mineral Density. New England Journal of Medicine. 2002;346(9):653-61.
62. Grey A, Bolland MJ, Horne A, Mihov B, Gamble G, Reid IR. Duration of antiresorptive activity of zoledronate in postmenopausal women with osteopenia: a randomized, controlled multidose trial. CMAJ Canadian Medical Association Journal. 2017;189(36):E1130-E6.
63. Boonen S, Reginster J-Y, Kaufman J-M, Lippuner K, Zanchetta J, Langdahl B, et al. Fracture Risk and Zoledronic Acid Therapy in Men with Osteoporosis. New England Journal of Medicine. 2012;367(18):1714-23.
64. Cummings SR, Martin JS, McClung MR, Siris ES, Eastell R, Reid IR, et al. Denosumab for Prevention of Fractures in Postmenopausal Women with Osteoporosis. New England Journal of Medicine. 2009;361(8):756-65.
65. Nct. Denosumab China Phase III Study. https://clinicaltrialsgov/show/NCT02014467 [Internet]. 2016. Available from: https://www.cochranelibrary.com/central/doi/10.1002/central/CN-01479832/full.
66. Zhu HM, Tang H, Cheng Q, He L, Li PQ, Xue QY, et al. Efficacy and safety of denosumab in Chinese postmenopausal women with osteoporosis at increased risk of fracture: Results from a 12-month, randomized, double-blind, placebo-controlled phase III study. Journal of Bone and Mineral Research Conference. 2017;31(Supplement 1).
67. Pitale S, Thomas M, Rathi G, Deshmukh V, Kumar P, Reddy S, et al. A randomized placebo-controlled trial of the efficacy of denosumab in Indian postmenopausal women with osteoporosis. Indian Journal of Endocrinology and Metabolism. 2015;19(1):148-54.
68. Boonen S, Adachi JD, Man Z, Cummings SR, Lippuner K, Törring O, et al. Treatment with Denosumab Reduces the Incidence of New Vertebral and Hip Fractures in Postmenopausal Women at High Risk. The Journal of Clinical Endocrinology & Metabolism. 2011;96(6):1727-36.

69. Bone HG, Bolognese MA, Yuen CK, Kendler DL, Wang H, Liu Y, et al. Effects of denosumab on bone mineral density and bone turnover in postmenopausal women. The Journal of clinical endocrinology and metabolism. 2008;93(6):2149-57.
70. McCloskey EV, Johansson H, Oden A, Austin M, Siris E, Wang A, et al. Denosumab reduces the risk of osteoporotic fractures in postmenopausal women, particularly in those with moderate to high fracture risk as assessed with FRAX. Journal of Bone and Mineral Research. 2012;27(7):1480-6.
71. McClung MR, Boonen S, Törring O, Roux C, Rizzoli R, Bone HG, et al. Effect of denosumab treatment on the risk of fractures in subgroups of women with postmenopausal osteoporosis. Journal of Bone and Mineral Research. 2012;27(1):211-8.
72. Silverman S, Viswanathan HN, Yang YC, Wang A, Boonen S, Ragi-Eis S, et al. Impact of clinical fractures on health-related quality of life is dependent on time of assessment since fracture: results from the FREEDOM trial. Osteoporosis International. 2012;23(4):1361-9.
73. Orwoll E, Teglbjaerg CS, Langdahl BL, Chapurlat R, Czerwinski E, Kendler DL, et al. A randomized, placebo-controlled study of the effects of denosumab for the treatment of men with low bone mineral density. Journal of Clinical Endocrinology & Metabolism. 2012;97(9):3161-9.
74. Fink HA, MacDonald R, Forte ML, Rosebush CE, Ensrud KE, Schousboe JT, et al. Long-Term Drug Therapy and Drug Holidays for Osteoporosis Fracture Prevention: A Systematic Review2019 2019.
75. Viswanathan M, Reddy S, Berkman N, Cullen K, Middleton JC, Nicholson WK, et al. Screening to Prevent Osteoporotic Fractures: Updated Evidence Report and Systematic Review for the US Preventive Services Task Force. JAMA. 2018;319(24):2532-51.
76. Crandall CJ, Newberry SJ, Diamant A, Lim YW, Gellad WF, Booth MJ, et al. Comparative effectiveness of pharmacologic treatments to prevent fractures: an updated systematic review. Annals of internal medicine. 2014;161(10):711-23.
77. Crandall CJ, Newberry SJ, Diamant A, Lim YW, Gellad WF, Suttorp MJ, et al. Treatment To Prevent Fractures in Men and Women With Low Bone Density or Osteoporosis: Update of a 2007 Report. Rockville (MD): Agency for Healthcare Research and Quality 2012.
78. Qaseem A, Forciea M, McLean RM, Denberg TD, for the Clinical Guidelines Committee of the American College of P. Treatment of low bone density or osteoporosis to prevent fractures in men and women: A clinical practice guideline update from the american college of physicians. Annals of internal medicine. 2017;166(11):818-39.
79. Chen LX, Ning GZ, Zhou ZR, Li YL, Zhang D, Wu QL, et al. The carcinogenicity of alendronate in patients with osteoporosis: evidence from cohort studies. PloS one. 2015;10(4):e0123080.
80. Davis S, Martyn-St James M, Sanderson J, Stevens J, Goka E, Rawdin A, et al. A systematic review and economic evaluation of bisphosphonates for the prevention of fragility fractures. Health Technology Assessment (Winchester, England). 2016;20(78):1-406.
81. Kranenburg G, Bartstra JW, Weijmans M, de Jong PA, Mali WP, Verhaar HJ, et al. Bisphosphonates for cardiovascular risk reduction: A systematic review and meta-analysis. Atherosclerosis. 2016;252:106-15.
82. Davis S, Simpson E, Hamilton J, Martyn-St James M, Rawdin A, Wong R, et al. Denosumab, raloxifene, romosozumab and teriparatide to prevent osteoporotic fragility fractures: a systematic review and economic evaluation. Southampton, UK; 2020.
83. Lv F, Cai X, Yang W, Gao L, Chen L, Wu J, et al. Denosumab or romosozumab therapy and risk of cardiovascular events in patients with primary osteoporosis: Systematic review and meta- analysis. Bone. 2020;130:115121.
84. Diedhiou D, Cuny T, Sarr A, Norou Diop S, Klein M, Weryha G. Efficacy and safety of denosumab for the treatment of osteoporosis: A systematic review. Annales d Endocrinologie. 2015;76(6):650-7.
85. Tsourdi E, Zillikens MC, Meier C, Body J-J, Rodriguez EG, Anastasilakis AD, et al. Fracture risk and management of discontinuation of denosumab therapy: a systematic review and position statement by ECTS. The Journal of Clinical Endocrinology & Metabolism. 2020.
86. Tripto-Shkolnik L, Fund N, Rouach V, Chodick G, Shalev V, Goldshtein I. Fracture incidence after denosumab discontinuation: Real-world data from a large healthcare provider. Bone. 2020;130:115150.

87. Sheridan SL, Sutkowi-Hemstreet A, Barclay C, Brewer NT, Dolor RJ, Gizlice Z, et al. A Comparative Effectiveness Trial of Alternate Formats for Presenting Benefits and Harms Information for Low-Value Screening Services: a Randomized Clinical Trial. JAMA internal medicine. 2016;176(1):31-41.
88. Hudson B, Toop L, Mangin D, Pearson J. Risk communication methods in hip fracture prevention: a randomised trial in primary care. British journal of general practice. 2011;61(589):e469-76.
89. Kalluru R, Petrie KJ, Grey A, Nisa Z, Horne AM, Gamble GD, et al. Randomised trial assessing the impact of framing of fracture risk and osteoporosis treatment benefits in patients undergoing bone densitometry. BMJ open. 2017;7(2):e013703.
90. Fuzzell LN, Fraenkel L, Stark SL, Seehra SS, Nelson C, Keleman A, et al. A Mixed Methods Study Exploring Older Womens' Attitudes Toward Osteoporosis Medications: Adapting a Health Communication Framework. Women's health reports (New Rochelle, NY). 2020;1(1):102-13.
91. de Bekker-Grob EW, Essink-Bot ML, Meerding WJ, Pols HA, Koes BW, Steyerberg EW. Patients' preferences for osteoporosis drug treatment: a discrete choice experiment. Osteoporosis International. 2008;19(7):1029-37.
92. Si L, Tu L, Xie Y, Palmer AJ, Gu Y, Zheng X, et al. Chinese patients’ preference for pharmaceutical treatments of osteoporosis: a discrete choice experiment. Archives of Osteoporosis. 2019;14(1):85.
93. Billington EO, Feasel AL, Kline GA. At Odds About the Odds: Women’s Choices to Accept Osteoporosis Medications Do Not Closely Agree with Physician-Set Treatment Thresholds. Journal of General Internal Medicine. 2019.
94. Smallwood AJ, Schapira MM, Fedders M, Neuner JM. A pilot randomized controlled trial of a decision aid with tailored fracture risk tool delivered via a patient portal. Osteoporosis international. 2017;28(2):567-76.
95. Liu CS, Feasel AL, Kline GA, Billington EO. Pharmacotherapy decisions among postmenopausal women attending a group medical consultation or a one-on-one specialist consultation at an osteoporosis center: an observational cohort study. Osteoporosis international : a journal established as result of cooperation between the European Foundation for Osteoporosis and the National Osteoporosis Foundation of the USA. 2021.
96. LeBlanc A, Wang AT, Wyatt K, Branda ME, Shah ND, Van Houten H, et al. Encounter Decision Aid vs. Clinical Decision Support or Usual Care to Support Patient-Centered Treatment Decisions in Osteoporosis: The Osteoporosis Choice Randomized Trial II. PLoS ONE [Electronic Resource]. 2015;10(5):e0128063.
97. Montori VM, Shah ND, Pencille LJ, Branda ME, Van Houten HK, Swiglo BA, et al. Use of a decision aid to improve treatment decisions in osteoporosis: the osteoporosis choice randomized trial. American journal of medicine. 2011;124(6):549-56.
98. Hudson B, Zarifeb A, Young L, Wells J. Patients' expectations of screening and preventive treatments. Annals of Family Medicine. 2012;10(6):495-502.
99. Neuner JM, Schapira MM. Patient perceptions of osteoporosis treatment thresholds. Journal of Rheumatology. 2014;41(3):516-22.
