# Supplementary material for: Screening for the primary prevention of fragility fractures among adults aged 40 years and older in primary care: systematic reviews of the effects and acceptability of screening and treatment, and the accuracy of risk prediction tools
Source: Syst Rev. 2023 Mar 21;12:51. doi: 10.1186/s13643-023-02181-w (PMC10029308; doi:10.1186/s13643-023-02181-w)
Supplement: Supplementary file 5 — Additional file 5. Excluded studies list. This file lists all studies excluded after full text appraisal, with reasons. [file 13643_2023_2181_MOESM5_ESM.pdf]

Screening for the primary prevention of fragility fractures among adults aged 40 years and older in primary care: systematic reviews of the effects and acceptability of screening and treatment, and the accuracy of risk prediction tools

Michelle Gates, Jennifer Pillay (corresponding), Megan Nuspl, Aireen Wingert, Ben Vandermeer, Lisa Hartling

Affiliation: Alberta Research Centre for Health Evidence, Department of Pediatrics, University of Alberta. Edmonton Clinic Health Academy, 11405-87 Avenue NW, Edmonton, Alberta, Canada T6G 1C9. E-mail: MG: michellegates85@gmail.com; JP: jpillay@ualberta.ca; MN: megan.nuspl@ualberta.ca; AW: awingert@ualberta.ca; BV: ben.vandermeer@ualberta.ca; LH: hartling@ualberta.ca

Additional file 5. Excluded studies list

Not primary research:

KQ1 (n=9)

Anonymous. Osteoporosis Prevention, Screening, and Diagnosis: ACOG Clinical Practice Guideline No. 1. Obstet Gynecol. 2021;138(3):494-506.

Chaouat D. New osteoformative agents for osteoporosis. Rev Med Interne. 2002;23(9):759-67.

Fuggle NR, Curtis EM, Ward KA, Harvey NC, Dennison EM, Cooper C. Fracture prediction, imaging and screening in osteoporosis. Nat Rev Endocrinol. 2019;15(9):535-47.

ISRCTN registry [Internet]. London: BMC. [date unknown]. Identifier ISRCTN55814835, A pragmatic randomised controlled trial of the effectiveness and cost-effectiveness of screening for osteoporosis in older women for the prevention of fractures; 2007 Jun 19 [cited 2020 Nov 12]. Available from: https://apps.who.int/trialsearch/Trial2.aspx?TrialID=ISRCTN55814835.

Kwok T, Choy D. A case for osteoporosis screening and treatment in older people to prevent hip fracture. Hong Kong Med J. 2017;23(4):421.

Leslie WD, Morin SN. New Developments in Fracture Risk Assessment for Current Osteoporosis Reports. Curr Osteoporos Rep. 2020;18(3):115-29.

Lim LS, Hoeksema LJ, Sherin K. Screening for Osteoporosis in the Adult U.S. Population. ACPM Position Statement on Preventive Practice. Am J Prev Med. 2009;36(4):366-75.

Merlijn T, Swart KMA, van der Horst HE, Netelenbos JC, Elders PJM. Fracture prevention by screening for high fracture risk: a systematic review and meta-analysis. Osteoporos Int. 2020;31(2):251-7.

Netherlands Trial Register [Internet]. Amsterdam: Academic Medical Center (The Netherlands). 2004 Oct 26. Identifier NTR2430, Stepped screening of fracture risk. A case finding and treatment program for women of 65 years of age and older in primary care; 2010 Jul 26 [cited 2020 Nov 12]. Available from: http://apps.who.int/trialsearch/Trial2.aspx?TrialID=NTR2430.

KQ2 (n=12)

Azagra R, Gabriel P, Aguye A, Morina D, en representacion de GROIMAP. The application of the FRIDEX calibration of the frax tool to determine the absolute risk of osteoporotic fracture among spanish women. Reumatologia Clinica. 2017;15(5):e68-69.

Azagra R, Gabriel P, Aguye A, Morina D, en representacion de G. The application of the FRIDEX calibration of the FRAX tool to determine the absolute risk of osteoporotic fracture among Spanish women. La aplicacion de la calibracion FRIDEX de la herramienta FRAX R para determinar el riesgo absoluto de fractura osteoporotica en mujeres espanolas. 2019;15(5):e68-e9.

Brewer L, Mellon L, Duggan J. Ability of fracture risk assessment tool and national osteoporosis guideline group guidance to stratify people appropriately before fracture. J Am Geriatr Soc. 2013;61(9):1633-4.

Buzkova P, Barzilay JI. Comment on Davis et al. Development and validation of a simple hip fracture risk prediction tool for type 2 diabetes: The fremantle diabetes study phase I. Diabetes care 2018;42:102-109. Diabetes Care. 2019;42(6):E100.

Dagan N, Barda N, Balicer R, Elnekave E, Bachmat E. Automatic evaluation of routine computed tomography scans for prediction of osteoporotic fractures. Isr J Health Policy Res. 2019;8(Suppl 1).

Harvey NC, Orwoll E, Kwok T, Karlsson MK, Rosengren BE, Ribom E, et al. Sarcopenia Definitions as Predictors of Fracture Risk Independent of FRAX, Falls, and BMD in the Osteoporotic Fractures in Men (MrOS) Study: A Meta-Analysis. J Bone Miner Res. 2021.

Kanis JA, McCloskey E, Johansson H, Oden A, Leslie WD. FRAX® with and without bone mineral density. Calcif Tissue Int. 2012;90(1):1-13.

Kanis JA. Tools forestimating fracture probability. Osteoporos Int. 2019;30(Suppl 2):S160.

Leslie WD, Lix LM. Comparison between various fracture risk assessment tools. Osteoporos Int. 2014;25(1):1-21.

Moppett I. Accuracy of possum and nottingham hip fracture score. Acta Anaesthesiol Scand. 2018;62(9):1333-4.

Silverman SL, Calderon AD. The utility and limitations of FRAX: a US perspective. Curr Osteoporos Rep. 2010;8(4):192-7.

Silverman SL, Komm BS, Mirkin S. Use of FRAX®-based fracture risk assessments to identify patients who will benefit from osteoporosis therapy. Maturitas. 2014;79(3):241-7.

KQ3a (n=7)

Adami S, Prizzi R, Colapietro F. Alendronate for the treatment of osteoporosis in men. Calcif Tissue Int. 2001;69(4):239-41.

Bianchi G, Giusti A, Palummeri E, Girasole G. Hot topics from the 4th international conference on osteoporosis in men. Int J Clin Rheumtol. 2009;4(1):41-4.

Donggrell S. New horizons for zoledronic acid: results of the HORIZON trials in postmenopausal women with osteoporosis and after hip fracture. Expert Opin Pharmacother. 2008; 9(4):663-8.

Donnelly E, Lane JM, Boskey AL. Research perspectives: the 2013 AAOS/ORS research symposium on bone quality and fracture prevention. J Orthop Res. 2014;32(7):855-64.

Ensrud KE, Crandall CJ. In older postmenopausal women with osteopenia, zoledronate reduced fragility fractures at 6 years. Ann Intern Med. 2019;170(8):JC42.

Ferrari S. Benefits/risk of long-term treatment of osteoporosis. Osteoporos Int. 2019;30(Suppl 1):S32.

Lewiecki EM, Bilezikian JP, Giangregorio L, Greenspan SL, Khosla S, Kostenuik P, et al. Proceedings of the 2018 Santa Fe Bone Symposium: advances in the management of osteoporosis. J Clin Densitom. 2019;22(1):1-19.

KQ4 (n=2)

DeJesus RS. Use of a clinical decision support system to increase osteoporosis screening. J Eval Clin Pract. 2012;18(4):926.

Prihar BJ, Katz S. Patient education as a tool to increase screening for osteoporosis. J Am Geriatr Soc. 2008;56(5):961-2.

Ineligible study design:

KQ1 (n=28)

Alvarado MA, Harrison A. Improving osteoporosis screening at MacNeal Family Medicine Center. J Womens Health. 2016;25(4):A3-A4.

Anonymous. Abstracts for 15th World Congress on Menopause. Climacteric. 2016;19(Suppl 1): iii-cxxix.

Anonymous. Abstracts of Osteoporosis Conference 2016. Osteoporos Int. 2016;27(Suppl 2):S609-S685.

Anonymous. World Congress on Osteoporosis, Osteoarthritis and Musculoskeletal Diseases (WCO-IOF-ESCEO 2017): Poster Abstracts. Osteoporos Int. 2017;28(Suppl 1): 127-636.

Borgstrom F, Jonsson E, Harvey N, Shepstone L, Lenaghan E, Clarke S, et al. Long-term cost-effectiveness of screening for fracture risk in a UK primary care setting. In: World Congress on Osteoporosis, Osteoarthritis and Musculoskeletal Diseases (WCO-IOF-ESCEO 2018): Oral Communication Abstracts. Osteoporos Int. 2018;29(Suppl 1):S68-S9.

Chen Y, Harrold LR, Yood RA, Field TS, Briesacher BA. Identifying patients with osteoporosis or at risk for osteoporotic fractures. Am J Manag Care. 2012;18(2):e61-7.

Croswell J. Screening for osteoporosis. Am Fam Physician. 2011;83(10):1201-2.

Dargent-Molina P, Piault S, Breart G, EPIDOS Study Group. A comparison of different screening strategies to identify elderly women at high risk of hip fracture: results from the EPIDOS prospective study. Osteoporos Int. 2003;14(12):969-77.

Edwards FD, Grover ML, Cook CB, Chang YH. Use of FRAX as a determinant for risk-based osteoporosis screening may decrease unnecessary testing while improving the odds of identifying treatment candidates. Womens Health Issues. 2014;24(6):629-34.

Ettinger B. Appropriate intervention through fracture risk assessment. Manag Care. 2005;14(Suppl 8):9-12.

Green AD, Colón-Emeric CS, Bastian L, Drake MT, Lyles KW. Does this woman have osteoporosis?. JAMA. 2004;292(23):2890-900.

Garduño-García JD, Pérez-Espejel I, Huitrón-Bravo G, Romero-Figueroa MD. Osteoporotic fracture risk evaluation. Options when central densitometry is not available. Rev Med Inst Mex Seguro Soc. 2014;52(6):674-9.

Jiang X, Good LE, Spinka R, Schnatz PF. Osteoporosis screening in postmenopausal women aged 50–64 years: BMI alone compared with current screening tools. Maturitas. 2016;83:59-64.

Japan Primary Registries Network (JPRN) [Internet]. Tokyo: University hospital Medical Information Network (UMIN) Center. [date unknown]. Identifier JPRN-UMIN000018306, Osteoporosis screening using FRAX by community pharmacists and Collaborative pharmacotherapy in the osteoporotic treatment; 2015 May 1 [cited 2020 Nov 12]. Available from: https://apps.who.int/trialsearch/Trial2.aspx?TrialID=JPRN-UMIN000018306.

Jung E, Kadri O, Taliaferro K, Buraimoh M, Bartol S. Osteopenia screening utilizing a novel CT-based dexa equivalent algorithm. Spine J. 2016;16(10):S299-S300.

Kearns AE. Osteoporosis secondary fracture prevention: A united voice. Endocr Pract. 2020;26(5):571-2.

Khalid S, Hawley S, Ali MS, Cooper C, Javaid K, Judge A, et al. Comparative fracture risk amongst users of different anti-osteoporosis drugs available in the UK in the NHS: a propensity-matched cohort study using data from the Clinical Practice Research Datalink. Osteoporos Int. 2016;27(Suppl 2):S631.

Lewiecki EM, Bilezikian JP, Giangregorio L, Greenspan SL, Khosla S, Kostenuik P, et al. Proceedings of the 2018 Santa Fe Bone Symposium: advances in the management of osteoporosis. J Clin Densitom. 2019;22(1):1-19.

McCloskey EV. Screening for high fracture risk in primary care. Osteoporos Int. 2022;32(Suppl 1):S91-S2.

Michaëlsson K, Bergström R, Mallmin H, Holmberg L, Wolk A, Ljunghall S. Screening for osteopenia and osteoporosis: selection by body composition. Osteoporos Int. 1996;6(2):120-6.

Pawar P, Malkauthekar M. Using machine learning to predict fracture risk in large U.S. population: An Analysis of NHANES 2005-2014. J Allergy Clin Immunol. 2022;149(2 Suppl):AB44.

Rajan R, Paul J, Cherian KE, Asha HS, Kapoor N, Paul TV. FRAX R with or without BMD and TBS predicts fragility fractures in community-dwelling rural southern Indian postmenopausal women. Arch Osteoporos. 2020;15(1):82.

Rendl S, Lapa C, Blumel C, Bundschuh RA, Schneider P. Decision making for osteoporotic treatment using FRAX or DVO risk algorithms in a clinical setting. J Musculoskelet Neuronal Interact. 2013;13(3):339-45.

Rothmann MJ, Huniche L, Ammentorp J, Barkmann R, Glüer CC, Hermann AP. Women's perspectives and experiences on screening for osteoporosis (Risk-stratified Osteoporosis Strategy Evaluation, ROSE). Arch Osteoporos. 2014;9(1):192.

Subasinghe HWAS, Lekamwasam S, Ball P, Morrissey H, Morrissey EI. Performance of Sri Lankan FRAX algorithm without bone mineral density and with Quantitative Ultrasound data input. Ceylon Med J. 2019;64(1):17-24.

Teede HJ, Renouf DA, Jayasuriya IA, Barrie D. STOP fracture study: southern health osteoporotic fracture screening project. Intern Med J. 2012;42(5):e74-9.

Turner DA, Khioe RF, Shepstone L, Lenaghan E, Cooper C, Gittoes N, et al. The cost-effectiveness of screening in the community to reduce osteoporotic fractures in older women in the UK: economic evaluation of the SCOOP Study. J Bone Miner Res. 2018;33(5):845-51.

Weinstein L, Ullery B. Identification of at-risk women for osteoporosis screening. Am J Obstet Gynecol. 2000;183(3):547-9.

KQ2 (n=20)

Abe Y, Nishimura T, Mizukami S, Aoyagi K. Efficiency of the combining use of Osteoporosis Screening Tool (OST) and FRAX in screening women with low bone mass. J Bone Miner Res. 2019;34(Suppl 1):236.

Altai Z, Qasim M, Li X, Viceconti M. The effect of boundary and loading conditions on patient classification using finite element predicted risk of fracture. Clin Biomech (Bristol, Avon). 2019;68:137-43.

Anonymous. Can frax help portuguese men? J Clin Densitom. 2016;19(4):529.

Azagra R, Zwart M, Aguye A, Moreno N, Gabriel P, Manresa JM, et al. Analysis of using FRAX with fridex risk thresholds in the application of DXA-scan in the Spanish female population over 45 years old. Osteoporosis International. 2016;27(Suppl 1):S356-S7.

Blanchard R, Morin C, Malandrino A, Vella A, Sant Z, Hellmich C. Patient-specific fracture risk assessment of vertebrae: A multiscale approach coupling x-ray physics and continuum micromechanics. Int J Numer Method Biomed Eng. 2016;32(9):e02760.

Brennan-Olsen SL, Holloway KL, Kotowicz MA, Pasco JA, Leslie WD. Associations between regular physical activity and exceeding intervention thresholds for fracture using frax calculated with trabecular bone scores. Osteoporos Int. 2016;27(Suppl 1):S434-S5.

Elnekave E, Bar A, Bregman-Amitai O, Orlovsky M, Dagan N, Barda N, et al. Automatic opportunistic ct based risk assessment of osteoporotic fracture: Results from a 48,744 subject, 5-year retrospective cohort study. Osteoporos Int. 2020;31(Suppl 1):S217.

Gladkova EN, Lesnyak OM. Frax-based intervention thresholds in the five countries of Eastern Europe. Osteoporos Int. 2020;31(Suppl 1):S433.

Harvey N, Cooper C, Kanis JA, Liu E, Lorentzon M, Bea JW, et al. DXA appendicular lean mass, FRAX, BMD and incident fractures: Findings from the Women's Health Initiative (WHI). J Bone Miner Res. 2019;34(Suppl 1):142.

Harvey NC, Kanis JA, Liu E, Johansson H, Lorentzon M, McCloskey E. Appendicular lean mass and fracture risk assessment: implications for FRAX R and sarcopenia. Osteoporos Int. 2019;30(3):537-9.

Holloway-Kew KL, Betson AG, Hyde NK, Kotowicz MA, Pasco JA. Prediction of fractures in men with dysglycaemia using FRAX (AUS). Osteoporos Int. 2019;30(Suppl 1):S74.

Lee SH, Lee JY, Lim K-H, Lee Y-S, Kim S-H, Choi S, et al. High Circulating Sphingosine 1-Phosphate is a Risk Factor for Osteoporotic Fracture Independent of Fracture Risk Assessment Tool. Calcif Tissue Int. 2020;107(4):362-70.

Leslie W, Lix L, Nguyen T, Eisman J, Morin S. Independent Performance Evaluation of the Garvan Fracture Risk Calculator: A Registry-Based Cohort Study. J Bone Miner Res. 2020;35(Suppl 1):197-8.

Li G, Zeng J, Leslie W, Papaioannou A, Thabane L, Levine M, et al. Combining frailty and trabecular bone score in predicting risk of major osteoporotic fractures. Osteoporos Int. 2019;30(Suppl 2):S320-S1.

Marco-Pascual C, Gonzalez-Gimenez X, Mora M, Medina P, Bianchi M-M, Santo P, et al. Applicability of frax in clinical practice: 10-year results. Arthritis and Rheumatol. 2019;71(Suppl 10):3921-2.

Nikitinskaya O, Toroptsova N, Demin N. Risk assessment of osteoporotic fractures: BMD vs . FRAX (10-year prospective cohort study). Osteoporos Int. 2019;30(Suppl 2):S485.

Rymar O, Mustafina S, Malyutina S, Mazurenko E, Bobak M. Validation of the 10-year risk of osteoporotic fractures score (FRAX) for the caucasian population of siberia. Osteoporos Int. 2019;30(Suppl 2):S429-S30.

Sandhu SK, Nguyen ND, Center JR, Pocock NA, Eisman JA, Nguyen TV. Prognosis of fracture: evaluation of predictive accuracy of the FRAX algorithm and garvan nomogram. Osteoporos Int. 2010;21(5):863-71.

Sornay-Rendu E, Duboeuf F, Chapurlat R. FRAX probabilities generally do not change much over time. A prospective analysis from the OFELY cohort. J Bone Miner Res. 2020;35(Suppl 1):216.

Wright NC, Daigle SG, Melton ME, Delzell ES, Balasubramanian A, Curtis JR. The Design and Validation of a New Algorithm to Identify Incident Fractures in Administrative Claims Data. J Bone Miner Res. 2019;34(10):1798-807.

KQ3a (n=8)

Bilezikian JP, Lin CJF, Brown JP, Wang AT, Yin X, Ebeling PR, et al. Long-term denosumab treatment restores cortical bone loss and reduces fracture risk at the forearm and humerus: analyses from the FREEDOM Extension cross-over group. Osteoporos Int. 2019;30(9):1855-64.

Boonen S, Orwoll E, Magaziner J, Colon-Emeric CS, Adachi JD, Bucci-Rechtweg C, et al. Once-yearly zoledronic acid in older men compared with women with recent hip fracture. J Am Geriatr Soc. 2011;59(11):2084-90.

Inoue D, Muraoka R, Okazaki R, Nishizawa Y, Sugimoto T. Efficacy and safety of risedronate in osteoporosis subjects with comorbid diabetes, hypertension, and/or dyslipidemia: a post hoc analysis of phase III trials conducted in Japan. Calcif Tissue Int. 2016;98(2):114-22.

Neviaser AS, Lane JM, Lenart BA, Edobor-Osula F, Lorich DG. Low-energy femoral shaft fractures associated with alendronate use. J Orthop Trauma. 2008;22(5):346-50.

Reid IR, Black DM, Eastell R, Bucci-Rechtweg C, Su G, Hue TF, et al. Reduction in the risk of clinical fractures after a single dose of zoledronic Acid 5 milligrams. J Clin Endocrinol Metab. 2013;98(2):557-63.

Siris ES, Simon JA, Barton IP, McClung MR, Grauer A. Effects of risedronate on fracture risk in postmenopausal women with osteopenia. Osteoporos Int. 2008;19(5):681-6.

Um MJ, Cho EA, Jung H. Combination therapy of raloxifene and alendronate for treatment of osteoporosis in elderly women. J Menopausal Med. 2017;23(1):56-62.

Watts NB, Josse RG, Hamdy RC, Hughes RA, Manhart MD, Barton I, et al. Risedronate prevents new vertebral fractures in postmenopausal women at high risk. J Clin Endocrinol Metab. 2003;88(2):542-9.

KQ3b - Not a systematic review (n=7)

Aung K, Htay T. 2016 - Review: Different bisphosphonates are equally effective and safe for preventing fragility fractures. ACP J Club. 2017;166:JC34.

Fadda V, Maratea D, Trippoli S, Messori A. Gastrointestinal and renal side effects of bisphosphonates: differentiating between no proof of difference and proof of no difference. J Endocrinol Invest. 2015;38:189-92.

Invernizzi M, Cisari C, Carda S. The potential impact of new effervescent alendronate formulation on compliance and persistence in osteoporosis treatment. Aging Clin Exp Res. 2015;27:107-13.

Iranikhah M, Deas C, Murphy P, Freeman MK. Effects of denosumab after treatment discontinuation : a review of the literature. Consult Pharm. 2018;33:142-51.

Khominsky A, Lim M. "Spontaneous" medication-related osteonecrosis of the jaw; two case reports and a systematic review. Aust Dent J. 2018;63:441-54.

Kim HY, Kim JW, Kim SJ, Lee SH, Lee HS. Uncertainty of current algorithm for bisphosphonate-related osteonecrosis of the jaw in population-based studies: a systematic review. J Bone Miner Res. 2017;32:584-91.

Mungan Z, Pinarbasi Simsek B. Which drugs are risk factors for the development of gastroesophageal reflux disease? Turk J Gastroenterol. 2017;28:S38-S43.

KQ4 (n=2)

Jensen AL, Wind G, Langdahl BL, Lomborg K. The impact of multifaceted osteoporosis group education on patients' decision-making regarding treatment options and lifestyle changes. J Osteoporos. 2018;2018:9703602.

Swart KMA, van Vilsteren M, van Hout W, Draak E, van der Zwaard BC; van der Horst HE. Factors related to intentional non-initiation of bisphosphonate treatment in patients with a high fracture risk in primary care: a qualitative study. BMC Fam Pract. 2018;19(1):141.

<u>Ineligible population:</u>

KQ1 (n=11)

Abou-Raya S, Abou-Raya A, Khadrawi T. AB0627 A randomized controlled trial of early initiation of osteoporosis assessment and management in the acute setting of the fracture clinic. Ann Rheum Dis. 2013;72(Suppl 3):A981.

Beaton DE, Mamdani M, Zheng H, Jaglal S, Cadarette SM, Bogoch ER, et al. Improvements in osteoporosis testing and care are found following the wide scale implementation of the Ontario Fracture Clinic Screening Program: an interrupted time series analysis. Medicine. 2017;96(48):e9012.

Buist DS, LaCroix AZ, Brenneman SK, Abbott III T. A population-based osteoporosis screening program: who does not participate, and what are the consequences? J Am Geriatr Soc. 2004;52(7):1130-7.

Clarke S, Bradley R, Simmonds B, Salisbury C, Benger J, Marques E, et al. Can paramedics use FRAX (the WHO Fracture Risk Assessment Tool) to help GPs improve future fracture risk in patients who fall? Protocol for a randomised controlled feasibility study. BMJ open. 2014;4(9):e005744.

Clarke S, Simmonds B, Gooberman-Hill R, Robinson M, Greenwood R, Marques et al. O32. Can paramedics use frax to identify patients at greatest risk of future fracture among those who fall? A feasibility study. Rheumatology. 2015;54(Suppl 1):i38.

Ikegami S, Kamimura M, Uchiyama S, Mukaiyama K, Kato H. Unilateral vs bilateral hip bone mineral density measurement for the diagnosis of osteoporosis J Clin Densitom. 2014;17(1):84-90.

Leung PC, Qin L, Au SK. Prevention of osteoporotic fractures among high-risk groups of post-menopausal women. Hong Kong Med J. 2006;12(4 Suppl 2):36-9.

NCT registry [internet]. Evaluation of Alerts in Promoting Bone Densitometry Scans. Available from: https://clinicaltrialsgov/show/NCT04955652.

Rothmann MJ, Möller S, Holmberg T, Højberg M, Gram J, Bech M, et al. Non-participation in systematic screening for osteoporosis—the ROSE trial. Osteoporos Int. 2017;28(12):3389-99.

Silverman S, Miller P, Sebba A, Weitz M, Wan X, Alam J, et al. The Direct Assessment of Nonvertebral Fractures in Community Experience (DANCE) study: 2-year nonvertebral fragility fracture results. Osteoporos Int. 2013;24(8):2309-17.

Zaman M, Shah A, Singal R, Kirmani A, Bhat AR, Singal RP. Role of dual energy x-ray absorptiometry (DEXA) scan in the diagnosis of chronic low back pain–a prospective randomized controlled study in osteoporotic patients hospitalized in a tertiary care institute. Maedica (Bucur). 2018;13(2):120-124.

KQ2 (n=29)

Adachi JD, Berger C, Barron R, Weycker D, Anastassiades TP, Davison KS, et al. Predictors of imminent non-vertebral fracture in elderly women with osteoporosis, low bone mass, or a history of fracture, based on data from the population-based Canadian Multicentre Osteoporosis Study (CaMos). Arch Osteoporos. 2019;14(1):53.

Akyea RK, McKeever TM, Gibson J, Scullion JE, Bolton CE. Predicting fracture risk in patients with chronic obstructive pulmonary disease: a UK-based population-based cohort study. BMJ Open. 2019;9(4):e024951.

Bisson EJ, Finlayson ML, Ekuma O, Marrie RA, Leslie WD. Accuracy of FRAX® in people with multiple sclerosis. J Bone Miner Res. 2019;34(6):1095-1100.

Casado E, Arevalo M, Profitos J, Lira A, Del Rio L, Larrosa M, et al. Osteoporosis and fractures in patients with cirrhosis: can FRAX be useful for screening? Osteoporos Int. 2018;29(Suppl 1):S420.

Casado E, Arevalo M, Profitos J, Lira A, Del Rio L, Valero O, et al. Osteoporosis and fractures in patients with cirrhosis. Can FRAX be useful for screening? Ann Rheum Dis. 2018;77(Suppl 2):1617.

Couraud G, Souffir C, Gaigneux E, Kolta S, Roux C, Briot K. Adjusting FRAX® on TBS for identification of subjects at high risk of fractures. Bone. 2017;101:214-8.

Davis WA, Hamilton EJ, Bruce DG, Davis TME. A hip fracture risk equation for type 2 diabetes: The fremantle diabetes study. Diabetologia. 2017;60 (Suppl 1):S112.

Davis WA, Hamilton EJ, Bruce DG, Davis TME. Development and validation of a simple hip fracture risk prediction tool for type 2 diabetes: the fremantle diabetes study phase I. Diabetes Care. 2019;42(1):102-9.

De Sousa Braz A, Falcao MV, De Carvalho Motta JA, Gama ALH, De Oliveira JCS, De Campos KVM, et al. Risk assessment of osteoporotic fracture in patients from 65 years attended in a reumathology clinic. Adv Rheumatol. 2018;58(Suppl 1):94.

Gomez-Vaquero C, Fabregas-Canales D, Seoane-Mato D, Sanchez-Piedra C, Bustabad-Reyes S, Diaz-Gonzalez F, et al. Fracture risk assessment in the general population in Spain by FRAX algorithm. EPISER2016 study. Medicina Clinica. 2020;154(5):163-70.

Kats A, Langsetmo L, Ensrud K, Boyd C, Diem S, Taylor B, et al. Impact of comorbidity and prognosis on hip fracture and mortality incidence among women in late life. J Bone Miner Res. 2018;33(Suppl 1):391-2.

Kim D, Cho SK, Park HR, Choi YY, Sung YK. Trabecular bone score combined with clinical risk factors can predict incident fracture in rheumatoid arthritis patients. Ann Rheum Dis. 2017;76 (Suppl 2):1447-8.

Kozhemyakina EV, Lesnyak OM, Gracheva TV, Gladkova EN, Sannikova OY. The discriminative ability of bmd, trabecular bone score and frax® for non-vertebral fractures in patients with rheumatoid arthritis. Osteoporosis International. 2017;28 (Suppl 1):S513-S4.

Leslie WD, Morin SN, Lix LM, McCloskey EV, Johansson H, Harvey NC, et al. Fracture prediction from FRAX for Canadian ethnic groups: a registry-based cohort study. Osteoporos Int. 2021;32(1):113-22.

Leslie WD, Whitlock R, Shaw J, Rigatto C, Thorlacius L, Komenda P, et al. FRAX predicts fracture risk in patients with non-dialysis chronic kidney disease. Osteoporos Int. 2017;28(Suppl 1):S284.

Li G, Papaioannou A, Thabane L, Adachi J. Frailty in combination with trabecular bone score for enhancing predictive accuracy of major osteoporotic fracture risk. J Bone Miner Res. 2019;34(Suppl 1):234-5.

Longobardi V, Zanchetta MB, Bogado C, Silveira F, Zanchetta JR. In young women with low bone mass, can bone microarchitecture discriminate between those with and without fractures? Osteoporos Int. 2020;31(Suppl 1):S280.

Lu H, Murphy WA, Ebede C, Karim E, Manocha S, Don-Pedro D, et al. Osteoporotic fracture risk assessment using FRAX following hematopoietic stem cell transplantation. Ann Rheum Dis. 2017;76 (Suppl 2):82-3.

Martinez-Laguna D, Tebe C, Nogues X, Kassim Javaid M, Cooper C, Moreno V, et al. Fracture risk in type 2 diabetic patients: a clinical prediction tool based on a large population-based cohort. PLoS ONE. 2018;13(9):e0203533.

Merilainen M, Oikarinen P, Mikkonen K, Kaakinen P. Predictors of fragility fractures and osteoporosis among people over 50 years old - a retrospective cohort study. Int J Orthop Trauma Nurs. 2020;36:100709.

Nickolas T, Shiau S, Nishiyama K, Cortez N, Shane E, Rodriquez-Barradas M, et al. FRAX underestimates hip fracture risk in older men with CKD. J Bone Miner Res. 2015;30(Suppl 1):S46.

Pluskiewicz W, Drozdzowska B, Adamczyk P. Ten-year fracture risk in the assessment of osteoporosis management efficacy in postmenopausal women: a pilot study. Climacteric. 2013;16(1):117-26.

Rotondi NK, Beaton DE, Elliot-Gibson V, Sujic R, Josse RG, Sale JEM, et al. Comparison of CAROC and FRAX in fragility fracture patients: agreement, clinical utility, and implications for clinical practice. J Rheumatol. 2016;43(8):1593-9.

Smith EE, Fang J, Alibhai SM, Cram P, Cheung AM, Casaubon LK, et al. Derivation and external validation of a scoring system for predicting fracture risk after ischemic stroke in a Canadian cohort. JAMA Neurol. 2019;76(8):925-931.

Shah V, Champakanath A, Keshawarz A, Snell-Bergeon J. Fracture Risk Assessment (FRAX) without BMD and Risk of Major Osteoporotic Fractures in Adults with Type 1 Diabetes. J Bone Miner Res. 2020;35(Suppl 1):205.

Shuler FD, Scott K, Wilson-Byrne T, Morgan L, Olajide OB. Improving rural bone health and minimizing fracture risk in west virginia: Validation of the world health organization frax assessment tool as a phone survey for osteoporosis detection. W V Med J. 2016;112(3):84-8.

Silverman S, Chien A. Clinical utility of trabecular bone score (TBS) in osteoporosis patient management. Osteoporos Int. 2020;31(Suppl 1):S601.

Su Y, Kwok TCY, Cummings SR, Yip BHK, Cawthon PM. Can Classification and Regression Tree Analysis Help Identify Clinically Meaningful Risk Groups for Hip Fracture Prediction in Older American Men (The MrOS Cohort Study)? JBMR Plus. 2019;3(10):e10207.

Whitlock RH, Leslie WD, Shaw J, Rigatto C, Thorlacius L, Komenda P, et al. The Fracture Risk Assessment Tool (FRAX) predicts fracture risk in patients with chronic kidney disease. Kidney Int. 2019;95(2):447-54.

KQ3a

Population is low risk or <40 years (n=3)

Chen Q, Hu C, Liu Y, Song R, Zhu W, Zhao H, et al. Pharmacokinetics, pharmacodynamics, safety, and tolerability of single-dose denosumab in healthy Chinese volunteers: a randomized, single-blind, placebo-controlled study. PLoS ONE. 2018;13(6):e0197984.

Frankel B, Krishna V, Vandergrift A, Bauer DC, Nicholas J. Natural history and risk factors for adjacent vertebral fractures in the fracture intervention trial. Spine. 2013;38(25):2201-7

Mersereau JE, Khouri C, Jaffe RB. The role of alendronate in mitigating perimenopausal bone loss: a double-blind randomized controlled pilot study. Fertil Steril. 2010;94(3):1138-40.

Population has >50% prior fractures or condition impacting bone metabolism (n=21)

Black DM, Cummings SR, Karpf DB, Cauley JA, Thompson DE, Nevitt MC, et al. Randomised trial of effect of alendronate on risk of fracture in women with existing vertebral fractures. Lancet. 1996;348(9041):1535-41.

Black DM, Delmas PD, Eastell R, Reid IR, Boonen S, Cauley JA, et al. Once-yearly zoledronic acid for treatment of postmenopausal osteoporosis. N Engl J Med. 2007;356(18):1809-22.

Bolland MJ, Horne AM, Briggs SE, Thomas MG, Reid I, Gamble GD, et al. Effects of intravenous zoledronate on bone turnover and bone density persist for at least 11 Years in HIV-infected men. J Bone Miner Res. 2019;34(7):1248-53.

Boonen S, Black DM, Colon-Emeric CS, Eastell R, Magaziner JS, Eriksen EF, et al. Efficacy and safety of a once-yearly intravenous zoledronic acid 5 mg for fracture prevention in elderly postmenopausal women with osteoporosis aged 75 and older. J Am Geriatr Soc. 2010;58(2):292-9.

Boonen S, Eastell R, Su G, Mesenbrink P, Cosman F, Cauley JA, et al. Time to onset of antifracture efficacy and year-by-year persistence of effect of zoledronic acid in women with osteoporosis. J Bone Miner Res. 2012;27(7):1487-93.

Boonen S, Orwoll ES, Wenderoth D, Stoner KJ, Eusebio R, Delmas PD. Once-weekly risedronate in men with osteoporosis: results of a 2-year, placebo-controlled, double-blind, multicenter study. J Bone Miner Res. 2009;24(4):719-25.

Cauley JA, Black D, Boonen S, Cummings SR, Mesenbrink P, Palermo L, et al. Once-yearly zoledronic acid and days of disability, bed rest, and back pain: randomized, controlled HORIZON Pivotal Fracture Trial. J Bone Miner Res. 2011;26(5):984-92.

Clemmesen B, Ravn P, Zegels B, Taquet AN, Christiansen C, Reginster JY. A 2-year phase II study with 1-year of follow-up of risedronate (NE-58095) in postmenopausal osteoporosis. Osteoporos Int. 1997;7(5):488-95.

Colón-Emeric C, Mesenbrink P, Lyles K, Pieper C, Boonen S, Delmas P, et al. Potential mediators of the mortality reduction with zoledronic acid after hip fracture. J Bone Miner Res. 2010; 25(1):91-7.

Ensrud KE, Black DM, Palermo L, Bauer DC, Barrett-Connor E, Quandt SA, et al. Treatment with alendronate prevents fractures in women at highest risk: results from the Fracture Intervention Trial. Arch Intern Med. 1997;157(22):2617-24.

Harris ST, Watts NB, Genant HK, McKeever CD, Hangartner T, Keller M, et al. Effects of risedronate treatment on vertebral and nonvertebral fractures in women with postmenopausal osteoporosis: a randomized controlled trial. JAMA. 1999;282(14):1344-52.

Jacques RM, Boonen S, Cosman F, Reid IR, Bauer DC, Black DM, et al. Relationship of changes in total hip bone mineral density to vertebral and nonvertebral fracture risk in women with postmenopausal osteoporosis treated with once-yearly zoledronic acid 5 mg: the HORIZON-Pivotal Fracture Trial (PFT). J Bone Miner Res. 2012;27(8):1627-34.

Masud T, McClung M, Geusens P. Reducing hip fracture risk with risedronate in elderly women with established osteoporosis. Clin Interv Aging. 2009;4:445-9.

McCloskey EV, Johansson H, Oden A, Austin M, Siris E, Wang A, et al. Denosumab reduces the risk of osteoporotic fractures in postmenopausal women, particularly in those with moderate to high fracture risk as assessed with FRAX. J Bone Miner Res. 2012;27(7):1480-6.

Miller PD, Schnitzer T, Emkey R, Orwoll E, Rosen C, Ettinger M, et al. Weekly oral alendronic acid in male osteoporosis. Clin Drug Investig. 2004;24(6):333-41.

Nakamura T, Matsumoto T, Sugimoto T, Hosoi T, Miki T, Gorai I, et al. Clinical Trials Express: fracture risk reduction with denosumab in Japanese postmenopausal women and men with osteoporosis: Denosumab fracture Intervention Randomized placebo Controlled Trial (DIRECT). J Clin Endocrinol Metab. 2014; 99(7):2599-607.

Orwoll E, Ettinger M, Weiss S, Miller P, Kendler D, Graham J, et al. Alendronate for the Treatment of Osteoporosis in Men. N Engl J Med. 2000;343(9):604-10.

Qi WX, Tang LN, He AN, Yao Y, Shen Z. Risk of osteonecrosis of the jaw in cancer patients receiving denosumab: a meta-analysis of seven randomized controlled trials. Int J Clin Oncol. 2014;19(2):403-10.

Reid IR, Black DM, Eastell R, Bucci-Rechtweg C, Su G, Hue TF, et al. Reduction in the risk of clinical fractures after a single dose of zoledronic acid 5 milligrams. J Clin Endocrinol Metab. 2013;98(2):557-63.

Sorensen OH, Crawford GM, Mulder H, Hosking DJ, Gennari C, Mellstrom D, et al. Long-term efficacy of risedronate: a 5-year placebo-controlled clinical experience. Bone. 2003;32:120-6.

Ste-Marie LG, Sod E, Johnson T, Chines A. Five years of treatment with risedronate and its effects on bone safety in women with postmenopausal osteoporosis. Calcif Tissue Int. 2004;75(6):469-76.

KQ3b (n=3)

Boquete-Castro A, Gomez-Moreno G, Calvo-Guirado JL, Aguilar-Salvatierra A, Delgado-Ruiz RA. Denosumab and osteonecrosis of the jaw. A systematic analysis of events reported in clinical trials. Clin Oral Implants Res. 2016;27:367-75.

Ottesen C, Schiodt M, Gotfredsen K. Efficacy of a high-dose antiresorptive drug holiday to reduce the risk of medication-related osteonecrosis of the jaw (MRONJ): a systematic review. Heliyon. 2020;6:e03795.

Yarom N, Shapiro CL, Peterson DE, Van Poznak CH, Bohlke K, Ruggiero SL, et al. Medication-related osteonecrosis of the jaw: MASCC/ISOO/ASCO clinical practice guideline. J Clin Oncol. 2019;37:2270-90.

KQ4 (n=21)

Baleanu F, Iconaru L, Kinnard V, Surquin M, Karmali R, Bergmann P, et al. Osteoporosis treatment gap in a prospective cohort of volunteer women. Osteoporos Int. 2020;31(Suppl 1):S184.

Chaine C, Ray P, Timoshanko J. Perspectives of women with experience of a fragility fracture in Europe: Attitudes towards future fracture risk, osteoporosis and pharmacotherapy. Osteoporos Int. 2019;30(Suppl 2):S631-S2.

Cornelissen D, Boonen A, Bours S, Evers S, Dirksen C, Hiligsmann M. Understanding patients' preferences for osteoporosis treatment: the impact of patients' characteristics on subgroups and latent classes. Osteoporos Int. 2020;31(1):85-96.

Danila MI, Rahn EJ, Mudano AS, Outman RC, Li P, Redden DT, et al. Factors associated with readiness for adopting osteoporosis treatment change. Arthritis Rheumatol. 2017;69(Suppl 10).

Darba J, Restovic G, Kaskens L, Balbona MA, Carbonell A, Cavero P, et al. Patient preferences for osteoporosis in Spain: a discrete choice experiment. Osteoporos Int. 2011;22(6):1947-54.

Deo P, Nayak R, Rajpura J. Women's attitudes and health beliefs toward osteoporosis screening in a community pharmacy. J Osteoporos. 2013;2013:650136.

Feldstein A, Elmer PJ, Smith DH, Herson M, Orwoll E, Chen C, et al. Electronic medical record reminder improves osteoporosis management after a fracture: a randomized, controlled trial. J Am Geriatr Soc. 2006;54(3):450-7.

Fernandez-Avila DG, Rincon-Riano DN, Pinzon DF, Gutierrez Davila JM. Low rate of densitometric diagnosis and treatment in patients with severe osteoporosis in Colombia. Arch Osteoporos. 2019;14(1):95.

Fraenkel L, Gulanski B, Wittink D. Patient treatment preferences for osteoporosis. Arthritis Rheum. 2006;55(5):729-35.

Gold DT, Safi W, Trinh H. Patient preference and adherence: comparative US studies between two bisphosphonates, weekly risedronate and monthly ibandronate. Curr Med Res Opin. 2006;22(12):2383-91.

Goldshtein I, Rouach V, Shamir-Stein N, Yu J, Chodick G. Role of side effects, physician involvement, and patient perception in non-adherence with oral bisphosphonates. Adv Ther. 2016;33(8):1374-84.

Halvorsen PA, Kristiansen IS. Decisions on drug therapies by numbers needed to treat: a randomized trial. Arch Intern Med. 2005;165(10):1140-6.

Jose D, Yu M, Torralba K, Downey C, Dunn J, Cabido V. Fracture Liaison Service at an Academic Center with an Open Health System: 30-month Outcomes. Arthritis Rheum. 2020;72(Suppl 10):216-8.

Linton DN, Inrig TM, Frankel L, Porteous J, Eatson H, Chepesiuk R, et al. Educational booklet reinforces knowledge of osteoporosis and influences intentions to improve bone health in previously diagnosed and treated patients. Osteoporosis Int. 2020;31(9):1703-11.

Majumdar SR, Johnson JA, McAlister FA, Bellerose D, Russell AS, Hanley DA, et al. Multifaceted intervention to improve diagnosis and treatment of osteoporosis in patients with recent wrist fracture: a randomized controlled trial. CMAJ. 2008;178(5):569-75.

Majumdar SR, McAlister FA, Johnson JA, Bellerose D, Siminoski K, Hanley DA, et al. Interventions to increase osteoporosis treatment in patients with 'incidentally' detected vertebral fractures. Am J Med. 2012;125(9):929-36.

Miller MJ, Jou T, Danila MI, Mudano AS, Rahn EJ, Outman RC, et al. Use of path modeling to inform a clinical decision support application to encourage osteoporosis medication use. RSAP. 2020.

Roux S, Beaulieu M, Beaulieu MC, Cabana F, Boire G. Priming primary care physicians to treat osteoporosis after a fragility fracture: an integrated multidisciplinary approach. J Rheumatol. 2013;40(5):703-11.

Silverman S, Calderon A, Kaw K, Childers TB, Stafford BA, Brynildsen W, et al. Patient weighting of osteoporosis medication attributes across racial and ethnic groups: a study of osteoporosis medication preferences using conjoint analysis. Osteoporos Int. 2013;24(7):2067-77.

Solimeo SL, Nguyen VTT, Edmonds SW, Lou Y, Roblin DW, Saag KG, et al. Sex differences in osteoporosis self-efficacy among community-residing older adults presenting for DXA. Osteoporos Int. 2019;30(5):1033-41.
Tso LS, Loi D, Mosley DG, Yi D, Stockl KM, Lew HC, et al. Evaluation of a nationwide pharmacist-led phone outreach program to improve osteoporosis management in older women with recently sustained fractures. J Manag Care Spec Pharm. 2015;21(9):803-10.

Intervention:

KQ1

Intervention not screening (n=18)

Actrn registry [internet]. Falls Assessment Clinical Trial. [no date] Available from: https://trialsearchwhoint/Trial2aspx?TrialID=ACTRN12605000054617. 2005.
Barker A, Cameron P, Flicker L, Arendts G, Brand C, Etherton-Beer C, et al. Evaluation of RESPOND, a patient-centred program to prevent falls in older people presenting to the emergency department with a fall: A randomised controlled trial. PLoS Med. 2019;16(5):e1002807.
Bruce J, Hossain A, Lall R, Withers EJ, Finnegan S, Underwood M, et al. Fall prevention interventions in primary care to reduce fractures and falls in people aged 70 years and over: The PreFIT three-arm cluster RCT. Health Technol Assess. 2021;25(34):1-113.
Chang SF, Hong CM, Yang RS. Global computer-assisted appraisal of osteoporosis risk in Asian women: an innovative study. J Clin Nurs. 2011;20(9-10):1357-64.
ClinicalTrials.gov [Internet]. Bethesda (MD): National Library of Medicine (US). 2000 Feb 29. Identifier NCT00285168, A Randomized Controlled Trial of a Bone Density Decision Aide. 2006 Feb 1 [cited 2020 Nov 12]. Available from: https://clinicaltrials.gov/ct2/show/NCT00285168.
Cockayne S, Pighills A, Adamson J, Fairhurst C, Drummond A, Hewitt C, et al. Can occupational therapist-led home environmental assessment prevent falls in older people? A modified cohort randomised controlled trial protocol. BMJ Open. 2018;8(9):e022488.
Hill K, Barker A, Cameron P, Flicker L, Arendts G, Brand C, et al. Responding to the first fall to prevent the second: Successful RCT in reducing falls using a person centred approach for older fallers presenting to emergency departments. Age Ageing. 2019;48(2).
ISRCTN registry [internet]. A randomised trial of a community pharmacist-initiated screening and intervention program for osteoporosis: the OsteoPharm Study. Available from: https://trialsearchwhoint/Trial2aspx?TrialID=ISRCTN54746861.
ISRCTN registry [internet]. The Chaos Clinic for prevention of falls and related injuries: a randomised, controlled trial. Available from: https://trialsearchwhoint/Trial2aspx?TrialID=ISRCTN48015966.
Lamb SE, Bruce J, Hossain A, Ji C, Longo R, Lall R, et al. Screening and Intervention to Prevent Falls and Fractures in Older People. N Engl J Med. 2020;383(19):1848-59.
Larson EB. In older adults, adding fall-risk screening with targeted interventions to mailed advice did not reduce fractures. Ann Intern Med. 2021;174(4):JC46.
Levy BT, Hartz A, Woodworth G, Xu Y, Sinift S. Interventions to improving osteoporosis screening: an Iowa Research Network (IRENE) study. J Am Board Fam Med. 2009;22(4):360-7.
McCloskey EV, Johansson H, Odén A, Vasireddy S, Kayan K, Pande K, et al. Ten-year fracture probability identifies women who will benefit from clodronate therapy—additional results from a double-blind, placebo-controlled randomised study. Osteoporos Int. 2009;20(5):811-7.
NTR registry [internet]. Preventive health survey in elderly people. Available from: https://trialsearchwhoint/Trial2aspx?TrialID=NTR664. 2006.
Outman RC, Warriner AH, Saag KG, Curtis JR. Improving osteoporosis screening through patient activation and self-scheduling. Arthritis Rheum. 2009;60:309.
Rapp K, Lamb SE, Roigk P, Becker C, Konnopka C, Konig H-H, et al. Effect of an osteoporotic fracture prevention program on fracture incidence in routine care: a cluster-randomized trial. BMC Med. 2022;20(1):49.
Schapira M, Neuner J, Smallwood A, Fedders M. A pilot randomized controlled trial of a decision aid with tailored fracture risk tool delivered via a patient portal. Osteoporos Int. 2017;28(2):567-76.

Warriner AH, Outman RC, Saag KG, Morgan SL, Kitchin E, Curtis JR. Self-scheduling of DXA scans to improve osteoporosis screening in women. Arthritis Rheum. 2010;62:1006.

Ineligible screening strategy (n=3)

Barr RJ, Stewart A, Torgerson DJ, Seymour DG, Reid DM. Screening elderly women for risk of future fractures—participation rates and impact on incidence of falls and fractures. Calcif Tissue Int. 2005;76(4):243-8.

Clark EM, Gould V, Morrison L, Ades AE, Dieppe P, Tobias JH. Randomized controlled trial of a primary care–based screening program to identify older women with prevalent osteoporotic vertebral fractures: cohort for Skeletal Health in Bristol and Avon (COSHIBA). J Bone Miner Res. 2012;27(3):664-71.

Schneider DL, Worley K, Beard MK, Iannini M, Ko M, McCallum J, et al. The primary care osteoporosis risk of fracture screening (POROS) study: design and baseline characteristics. Contemp Clin Trials. 2010;31(4):336-44.

KQ2

Not screening, or ineligible screening method (n=27)

Adams AL, Fischer H, Kopperdahl DL, Lee DC, Black DM, Bouxsein ML, et al. Osteoporosis and hip fracture risk from routine computed tomography scans: the Fracture, Osteoporosis, and CT Utilization Study (FOCUS). J Bone Miner Res. 2018;33(7):1291-301.

Almog Y, Rai A, Mishra A, Moulaison A, Powell R, Weinberg K, et al. Crystal Bone: Personalized, Short-term Fracture Risk Prediction with Natural Language Processing Methods. J Bone Miner Res. 2020;35(Suppl 1):200.

Beaudoin C, Jean S, Moore L, Gamache P, Bessette L, Ste-Marie LG, et al. History of fractures for prediction of a future fracture in individuals from the general population. J Bone Miner Res. 2017;32(Suppl 1):S17.

Bhattacharya P, Altai Z, Qasim M, Viceconti M. A multiscale model to predict current absolute risk of femoral fracture in a postmenopausal population. Biomech Model Mechanobiol. 2019;18(2):301-18.

Borowy P, Czerwinski E. The predictive value of densitometry of the spine and proximal femur in predicting fracture in postmenopausal women. Osteoporos Int. 2016;27(Suppl 1):S416-S7.

Chan MY, Nguyen ND, Center JR, Eisman JA, Nguyen TV. Absolute fracture-risk prediction by a combination of calcaneal quantitative ultrasound and bone mineral density. Calcif Tissue Int. 2012;90(2):128-36.

Chan MY, Nguyen ND, Center JR, Eisman JA, Nguyen TV. Quantitative ultrasound and fracture risk prediction in non-osteoporotic men and women as defined by who criteria. Osteoporos Int. 2013;24(3):1015-22.

Chapurlat R, Sornay-Rendu E, Zebaze R, Bui M, Seeman E, Lespessailles E. Deterioration of bone microstructure identifies women at imminent risk of fragility fractures. J Bone Miner Res. 2018;33(Suppl 1):38.

Crandall C, Larson J, Wright N, Laddu D, Stefanick M, Kaunitz A, et al. Does Serial Bone Density Measurement Meaningfully Improve Incident Fracture Risk Prediction in Postmenopausal Women? Results from the Women's Health Initiative Observational Study and Clinical Trials. J Bone Miner Res. 2020;35(Suppl 1):213-4.

De Laet CE, Van Hout BA, Burger H, Weel AE, Hofman A, Pols HA. Hip fracture prediction in elderly men and women: validation in the rotterdam study. J Bone Miner Res. 1998;13(10):1587-93.

Desbiens L-C, Goupil R, Mac-Way F. Predictive value of quantitative ultrasound parameters in individuals with chronic kidney disease: A population-based analysis of CARTaGENE. Bone. 2020;130:115120.

Gonnelli S, Caffarelli C, Bianchi G, Girasole G, Brandi ML, Casciaro S, et al. Identification of patients at high risk for osteoporotic fractures through radio frequency echographic multispectrometry (REMS): Results of a 5-year follow-up study. Osteoporos Int. 2019;30(Suppl 2):S638.

Jazinizadeh F, Quenneville CE. Enhancing hip fracture risk prediction by statistical modeling and texture analysis on DXA images. Med Eng Phys. 2020;78:14-20.

Ladang A, Beaudart C, Locquet M, Reginster J, Bruyere O, Cavalier E. Fragility fracture risk prediction in elderly people based on a microrna panel. Clinica Chimica Acta. 2019;493 (Suppl 1):S168-S9.

Lui L-Y, Cummings S, Kim KM, Cauley J, Ensrud K, Orwoll E, et al. Red cell distribution width (RDW), A widely available simple measure of cell aging, strongly predicts hip fracture. J Bone Miner Res. 2019;34(Suppl 1):22-3.

Miller PD, Siris ES, Barrett-Connor E, Faulkner KG, Wehren LE, Abbott TA, et al. Prediction of fracture risk in postmenopausal white women with peripheral bone densitometry: evidence from the national osteoporosis risk assessment. J Bone Miner Res. 2002;17(12):2222-30.

Prokopiou D, Rogers K, Evans P, Elarnaut F, Lyburn I, Wilson M, et al. Point of care fracture prediction for osteoporosis using a conical shell x-ray beam. Osteoporos Int. 2020;31(Suppl 1):S412.

Sarvi MN, Luo Y. Improving the prediction of sideways fall-induced impact force for women by developing a female-specific equation. J Biomech. 2019;88:64-71.

Sheer R, Jiang Y, Sudharshan L, Pasquale M. Risk predictors for seniors at imminent risk of fracture. J Bone Miner Res. 2019;34(Suppl 1):103.

Sheer RL, Barron RL, Sudharshan L, Pasquale MK. Validated prediction of imminent risk of fracture for older adults. The Am J Manag Care. 2020;26(3):e91-e7.

Szulc P, Chapurlat R, Sornay-Rendu E, Bouxsein M, Boyd S, Biver E, et al. HIGH RESOLUTION PERIPHERAL QCT (HR-pQCT) PREDICTS FRACTURES for ALL AGE GROUPS in OLDER ADULTS - The BOMIC CONSORTIUM. J Bone Miner Res. 2020;35(Suppl 1):201.

Valentinitsch A, Trebeschi S, Kaesmacher J, Lorenz C, Loffler MT, Zimmer C, et al. Opportunistic osteoporosis screening in multi-detector ct images via local classification of textures. Osteoporos Int. 2019;30(6):1275-85.

Wright NC, Daigle SG, Balasubramanian A, Arora T, Delzell ES, Curtis JR. Validating updated claims-based algorithms to identify incident fragility fractures. Pharmacoepidemiol Drug Saf. 2018;27 (Suppl 2):128.

Wright NC, Daigle SG, Melton ME, Delzell ES, Balasubramanian A, Curtis JR. The design and validation of a new algorithm to identify incident fractures in administrative claims data. J Bone Miner Res. 2019;06.

Wright N, Delzell E, Daigle S, Melton M, Curtis J, Balasubramanian A. The design and validation of a new algorithm to identify initial incident and recurrent incident fragility fractures in administrative claims data. J Bone Miner Res. 2018;33(Suppl 1):267.

Yang S, Leslie WD, Luo Y, Goertzen AL, Ahmed S, Ward LM, et al. Automated dxa-based finite element analysis for hip fracture risk stratification: a cross-sectional study. Osteoporos Int. 2018;29(1):191-200.

Yang S, Luo Y, Yang L, Dall'Ara E, Eastell R, Goertzen AL, et al. Comparison of femoral strength and fracture risk index derived from dxa-based finite element analysis for stratifying hip fracture risk: a cross-sectional study. Bone. 2018;110:386-91.

Risk assessment tool not used to predict fractures (n=22)

Almog YA, Rai A, Zhang P, Moulaison A, Powell R, Mishra A, et al. Deep Learning With Electronic Health Records for Short-Term Fracture Risk Identification: Crystal Bone Algorithm Development and Validation. J Med Internet Res. 2020;22(10):e22550.

Cadarette SM, Jaglal SB, Kreiger N, McIsaac WJ, Darlington GA, Tu JV. Development and validation of the osteoporosis risk assessment instrument to facilitate selection of women for bone densitometry. CMAJ. 2000;162(9):1289-94.

Cadarette SM, Jaglal SB, Murray TM. Validation of the simple calculated osteoporosis risk estimation (SCORE) for patient selection for bone densitometry. Osteoporos Int. 1999;10(1):85-90.

Cass AR, Shepherd AJ, Carlson CA. Osteoporosis risk assessment and ethnicity: validation and comparison of 2 clinical risk stratification instruments. J Gen Intern Med. 2006;21(6):630-5.

Donaldson MG, Palermo L, Schousboe JT, Ensrud KE, Hochberg MC, Cummings SR. FRAX and risk of vertebral fractures: the fracture intervention trial. J Bone Miner Res. 2009;24(11):1793-9.

Fujiwara S, Masunari N, Suzuki G, Ross PD. Performance of osteoporosis risk indices in a Japanese population. 2001;62(8):586-94.

Gourlay ML, Miller WC, Richy F, Garrett JM, Hanson LC, Reginster JY. Performance of osteoporosis risk assessment tools in postmenopausal women aged 45-64 years. Osteoporos Int. 2005;16(8):921-7.

Gourlay ML, Powers JM, Lui LY, Ensrud KE. Clinical performance of osteoporosis risk assessment tools in women aged 67 years and older. Osteoporos Int. 2008;19(8):1175-83.

Hamdy RC, Seier E, Whalen K, Clark WA, Hicks K, Piggee TB. FRAX calculated without BMD does not correctly identify caucasian men with densitometric evidence of osteoporosis. Osteoporos Int. 2018;29(4):947-52.

Inderjeeth CA, Raymond WD. Case finding with GARVAN fracture risk calculator in primary prevention of fragility fractures in older people. Arch Gerontol Geriatr. 2019:103940.

Jiang X, Good LE, Spinka R, Schnatz PF. Osteoporosis screening in postmenopausal women aged 50-64 years: BMI alone compared with current screening tools. Maturitas. 2016;83:59-64.

Kirilova E, Kirilov N, Bischoff F. Predictive ability of the osteoporosis self-assessment tool for assessing the risk of osteoporosis. Revmatologiia (Bulgaria). 2019;27(3):3-9.

Leslie W, Yan L, Lix L, Moller S, Abrahamsen B, Rubin K, et al. External Validation of FREM in 74,828 Patients from Manitoba, Canada: A Registry-Based Cohort Study. J Bone Miner Res. 2020;35(Suppl 1):202.
Li G, Prior JC, Leslie WD, Thabane L, Papaioannou A, Josse RG, et al. Frailty and Risk of Fractures in Patients With Type 2 Diabetes. Diabetes Care. 2019;42(4):507-13.
McArthur C, Adachi JD, Ioannidis G, Papaioannou A, Jantzi M, Hirdes J, et al. Validation of the Fracture Risk Scale Home Care (FRS-HC) Across 4 Canadian Provinces. J Am Med Dir Assoc. 2021;22(5):1114-6.
Oka R, Ohira M, Suzuki S, Yoshida T, Koide H, Tanaka T, et al. Fracture Risk Assessment Tool (FRAX) and for the diagnosis of osteoporosis in japanese middle-aged and elderly women: Chiba bone survey. Endocr J. 2018;65(2):193-202.
Pang WY, Inderjeeth CA. FRAX without bone mineral density versus osteoporosis self-assessment screening tool as predictors of osteoporosis in primary screening of individuals aged 70 and older. J Am Geriatr Soc. 2014;62(3):442-6.
Robbins J, Aragaki AK, Kooperberg C, Watts N, Wactawski-Wende J, Jackson RD, et al. Factors associated with 5-year risk of hip fracture in postmenopausal women. JAMA. 2007;298(20):2389-98.
Sung KH, Choi Y, Cho GH, Chung CY, Park MS, Lee KM. Peripheral dxa measurement around ankle joint to diagnose osteoporosis as assessed by central dxa measurement. Skeletal Radiol. 2018;47(8):1111-7.
Ungar WJ, Josse R, Lee S, Ryan N, Adachi R, Hanley D, et al. The Canadian SCORE questionnaire: optimizing the use of technology for low bone density assessment. Simple calculated osteoporosis risk estimate. J Clin Densitom. 2000;3(3):269-80.
Von Muhlen D, Visby Lunde A, Barrett-Connor E, Bettencourt R. Evaluation of the simple calculated osteoporosis risk estimation (SCORE) in older Caucasian women: the Rancho Bernardo Study. Osteoporos Int. 1999;10(1):79-84.
Weinstein L, Ullery B. Identification of at-risk women for osteoporosis screening. Am J Obstet Gynecol. 2000;183(3):547-9.

Risk assessment tool does not predict 5- or 10-year fracture risk (n=7)

Ensrud KE, Ewing SK, Taylor BC, Fink HA, Cawthon PM, Stone KL, et al. Comparison of 2 frailty indexes for prediction of falls, disability, fractures, and death in older women. Arch Intern Med. 2008;168(4):382-9.
Ensrud KE, Lui L-Y, Taylor BC, Schousboe JT, Donaldson MG, Fink HA, et al. A comparison of prediction models for fractures in older women: Is more better? Arch Intern Med. 2009;169(22):2087-94.
Gonzalez-Macias J, Marin F, Vila J, Diez-Perez A. Probability of fractures predicted by FRAX® and observed incidence in the spanish ECOSAP study cohort. Bone. 2012;50(1):373-7.
Kim HY, Jang EJ, Park B, Kim TY, Shin SA, Ha YC, et al. Development of a Korean Fracture Risk Score (KFRS) for predicting osteoporotic fracture risk: analysis of data from the Korean national health insurance service. PLoS ONE. 2016;11(7):e0158918.
Li G, Papaioannou A, Thabane L, Levine MAH, Ioannidis G, Wong AKO, et al. Modifying the phenotypic frailty model in predicting risk of major osteoporotic fracture in the elderly J Am Med Dir Assoc. 2017;18(5):414-9.
Morin S, Tsang JF, Leslie WD. Weight and body mass index predict bone mineral density and fractures in women aged 40 to 59 years. Osteoporos Int. 2009;20(3):363-70.
Zaslavsky O, Zelber-Sagi S, Gray SL, LaCroix AZ, Brunner RL, Wallace RB, et al. Comparison of frailty phenotypes for prediction of mortality, incident falls, and hip fracture in older women. J Am Geriatr Soc. 2016;64(9):1858-62.

Risk assessment tool not readily available to clinicians (n=3)

Ettinger B, Hillier TA, Pressman A, Che M, Hanley DA. Simple computer model for calculating and reporting 5-year osteoporotic fracture risk in postmenopausal women J Womens Health. 2005;14(2):159-71.
Leslie WD, Tsang JF, Lix LM. Validation of ten-year fracture risk prediction: a clinical cohort study from the Manitoba Bone Density Program. Bone. 2008;43(4):667-71.
Melton Iii LJ, Atkinson EJ, Khosla S, Oberg AL, Riggs BL. Evaluation of a prediction model for long-term fracture risk. J Bone Miner Res. 2005;20(4):551-6.

Risk assessment tool not externally validated (n=30)

Adams A, Johnson E, Zhou H, Platt R, Wenkert D, Jacobsen S, et al. Prediction of two-year risk of fracture among older us women. J Bone Miner Res. 2016;31(Suppl 1):S94.

Beaudoin C, Jean S, Moore L, Gamache P, Bessette L, Ste-Marie LG, et al. Number, location, and time since prior fracture as predictors of future fracture in the elderly from the general population. J Bone Miner Res. 2018;33(11):1956-66.

Black DM, Cauley JA, Wagman R, Ensrud K, Fink HA, Hillier TA, et al. The ability of a single bmd and fracture history assessment to predict fracture over 25 years in postmenopausal women: the study of osteoporotic fractures. J Bone Miner Res. 2018;33(3):389-95.

Bon J, Nouraie SM, Smith KJ, Dransfield MT, McDonald M-L, Hoffman EA, et al. Lung-Specific Risk Factors Associated With Incident Hip Fracture in Current and Former Smokers. J Bone Miner Res. 2020;35(10):1952-61.

Boutin E, Natella PA, Schott AM, Bastuji-Garin S, David JP, Paillaud E, et al. Interrelations between body mass index, frailty, and clinical adverse events in older community-dwelling women: the EPIDOS cohort study. Clin Nutr. 2018;37(5):1638-44.

Cauley JA, Cawthon PM, Peters KE, Cummings SR, Ensrud KE, Bauer DC, et al. Risk factors for hip fracture in older men: the osteoporotic fractures in men study (MrOS). J Bone Miner Res. 2016;31(10):1810-9.

Cummings SR, Nevitt MC, Browner WS, Stone K, Fox KM, Ensrud KE, et al. Risk factors for hip fracture in white women. Study of Osteoporotic Ffractures Research Group. N Engl J Med. 1995;332(12):767-73.

Czerwinski E, Kanis JA, Osieleniec J, Kumorek A, Milert A, Johansson H, et al. Evaluation of frax to characterise fracture risk in poland. Osteoporos Int. 2011;22(9):2507-12.

Giangregorio LM, Leslie WD. Time since prior fracture is a risk modifier for 10-year osteoporotic fractures. J Bone Miner Res. 2010;25(6):1400-5.

Harvey NC, Oden A, Orwoll E, Lapidus J, Kwok T, Karlsson M, et al. Physical performance or function, but not appendicular lean mass, predict incident fractures independently of FRAX probability and BMD: results from the osteoporotic fractures in men (MROS) cohort. Osteoporos Int. 2018;29(1 Suppl 1):S69-S70.

Hedlin H, Weitlauf J, Crandall CJ, Nassir R, Cauley JA, Garcia L, et al. Development of a comprehensive health-risk prediction tool for postmenopausal women. Menopause (NY). 2019;26(12):1385-94.

Henry MJ, Pasco JA, Sanders KM, Nicholson GC, Kotowicz MA. Fracture risk (frisk) score: Geelong osteoporosis study. Radiology. 2006;241(1):190-6. Epub 2006/08/25.

Jang EJ, Lee YK, Choi HJ, Ha YC, Jang S, Shin CS, et al. Osteoporotic fracture risk assessment using bone mineral density in korean: a community-based cohort study. J Bone Metab. 2016;23(1):34-9.

Kamiya K, Kajita E, Tachiki T, Ikehara S, Kouda K, Sato Y, et al. Association between hand-grip strength and site-specific risks of major osteoporotic fracture: Results from the Japanese Population-based Osteoporosis Cohort Study. Maturitas. 2019;130:13-20.

Kilpatrick RD, Chiuve SE, Leslie WD, Wegrzyn LR, Gao W, Yang H, et al. Estimating the Effect of Elagolix Treatment for Endometriosis on Postmenopausal Bone Outcomes: A Model Bridging Phase III Trials to an Older Real-World Population. JBMR Plus. 2020;4(12):e10401.

Lalmohamed A, Welsing PM, Lems WF, Jacobs JW, Kanis JA, Johansson H, et al. Calibration of FRAX® 3.1 to the Dutch population with data on the epidemiology of hip fractures. Osteoporos Int. 2012;23(3):861-9.

Leslie WD, Lix LM, Johansson H, Oden A, McCloskey E, Kanis JA. Spine-hip discordance and fracture risk assessment: A physician-friendly FRAX enhancement. Osteoporos Int. 2011;22(3):839-47.

Marques A, Mota A, Canhao H, Romeu JC, Machado P, Ruano A, et al. A FRAX model for the estimation of osteoporotic fracture probability in Portugal. Acta Reumatol Port. 2013;38(2):104-12.

Martinez-Laguna D, Carbonell-Abella C, Reyes C, Tebe C, Pallares N, Nogues X, et al. A clinical tool for automated prediction of hip and major osteoporotic at five-and one-years fractures using electronic medical records data: The epic study. Osteoporos Int. 2020;31(Suppl 1):S53-S4.

Merlijn T, Swart KM, van Schoor NM, Heymans MW, van der Zwaard BC, van der Heijden AA, et al. The effect of a screening and treatment program for the prevention of fractures in older women: a randomized pragmatic trial. J Bone Miner Res. 2019;34(11):1993-2000.

Nguyen ND, Frost SA, Center JR, Eisman JA, Nguyen TV. Development of a nomogram for individualizing hip fracture risk in men and women. Osteoporos Int. 2007;18(8):1109-17.

Nguyen ND, Frost SA, Center JR, Eisman JA, Nguyen TV. Development of prognostic nomograms for individualizing 5-year and 10-year fracture risks. Osteoporos Int. 2008;19(10):1431-44.

Nguyen ND, Pongchaiyakul C, Center JR, Eisman JA, Nguyen TV. Identification of high-risk individuals for hip fracture: a 14-year prospective study. J Bone Miner Res. 2005;20(11):1921-8.

Skripnikova IA, Myagkova MA, Shalnova SA, Vygodin VA, Drapkina OM, Redko AN, et al. Assessment of the absolute risk

of fractures among urban and rural populations in 4 regions of the Russian federation. Profilakticheskaya Meditsina. 2020;23(5):33-9.
Swart K, Elders P, Netelenbos C. Screening of high fracture risk in primary care not effective Thomas Merlijn. J Bone Miner Res. 2018;33(Suppl 1):8.
Taylor BC, Schreiner PJ, Stone KL, Fink HA, Cummings SR, Nevitt MC, et al. Long-term prediction of incident hip fracture risk in elderly white women: Study of osteoporotic fractures. J Am Geriatr Soc. 2004;52(9):1479-86.
Tebe C, Pallares N, Carbonell-Abella C, Reyes C, Martinez-Laguna D, Nogues X, et al. A clinical tool for automated prediction of hip and major osteoporotic fractures using electronic medical records data: the epic study. Ann Rheum Dis. 2019;78(Suppl 2):1389.
Tebe C, Pallares N, Diez-Perez A, Nogues-Solan X, Martinez-Laguna D, Prieto-Alhambra D. Total hip BMD combined with age and fracture history are high ly predictive of hip fracture risk in actual practice settings: A realworld cohort including 21,278 postmenopausal women from catalonia. Osteoporos Int. 2019;30(Suppl 2):S453-S4.
Wang Y, Mehrabi S, Sohn S, Atkinson EJ, Amin S, Liu H. Natural language processing of radiology reports for identification of skeletal site-specific fractures. BMC medical informatics and decision making. 2019;19(Suppl 3):73.
Zhao J-H, Shi H-P, Jiang D-J, Wang L-T, Chen S-B, Jia W-T. Analysis of Combined Indicators for Risk of Osteoporotic Hip Fracture in Elderly Women. Orthop Surg. 2021.

KQ3a

Wrong drug or complex intervention (n=6)

Clinical Trials Peruvian Registry [Internet].]. Lima: National Institute of Health. [date unknown]. Identifier PER-089-12, A multicenter, international, randomized, double-blind, placebo-controlled, parallel-group study to assess the efficacy and safety of amg 785 treatment in postmenopausal women with osteoporosis; 2013 Jan 7 [cited 2020 Jul 27]. Available from: https://apps.who.int/trialsearch/Trial2.aspx?TrialID=PER-089-12.
Elders PJM, Merlijn T, Swart KMA, van Hout W, van der Zwaard BC, Niemeijer C, et al. Design of the SALT Osteoporosis Study: a randomised pragmatic trial, to study a primary care screening and treatment program for the prevention of fractures in women aged 65 years or older. BMC Musculoskelet Disord. 2017;18(1):424.
Ito M, Sone T, Shiraki M, Tanaka S, Irie C, Ota Y, et al. The effect of once-yearly zoledronic acid on hip structural and biomechanical properties derived using computed tomography (CT) in Japanese women with osteoporosis. Bone. 2018;106:179-86.
Khanizadeh F, Rahmani A, Asadollahi K, Ahmadi MRH. Combination therapy of curcumin and alendronate modulates bone turnover markers and enhances bone mineral density in postmenopausal women with osteoporosis. Arch Endocrinol Metab. 2018;62(4):438-45.

KQ4

Ineligible screening or treatment approach (n=7)

Christell H, Gullberg J, Nilsson K, Heidari Olofsson S, Lindh C, Davidson T. Willingness to pay for osteoporosis risk assessment in primary dental care. Health Econ Rev. 2019;9(1):14.
Edmonds SW, Solimeo SL, Lu X, Roblin DW, Saag KG, Cram P. Developing a bone mineral density test result letter to send to patients: a mixed-methods study. Patient Prefer Adherence. 2014;8:827-41.
Leonardi Reyes F, Florez S, Hauber B, Fernandez MM, Boeri M, Graham-Clarke P, et al. PATIENT PREFERENCES FOR BIOLOGIC AND BIOSIMILAR OSTEOPOROSIS TREATMENTS IN COLOMBIA: A DISCRETE-CHOICE EXPERIMENT. ViHRI. 2019;19(Supplement):S54.
Rothert ML, Holmes-Rovner M, Rovner D, Kroll J, Breer L, Talarczyk G, et al. An educational intervention as decision support for menopausal women. Res Nurs Health. 1997;20(5):377-87.
Urano T, Shiraki M, Saito M, Sasaki N, Ouchi Y, Inoue S. Polymorphism of SLC25A32, the folate transporter gene, is associated with plasma folate levels and bone fractures in Japanese postmenopausal women. Geriatr Gerontol Int. 2014;14(4):942-6.
Wang C, O'Neill SM, Rothrock N, Gramling R, Sen A, Acheson LS, et al. Comparison of risk perceptions and beliefs across common chronic diseases. Prev Med. 2009;48(2):197-202.

Zikmund-Fisher BJ, Couper MP, Singer E, Levin CA, Fowler FJ, Ziniel S, et al. The DECISIONS Study: a nationwide survey of United States adults regarding 9 common medical decisions. Med Decis Making. 2010;30(5 suppl):20S-34S.

Inadequate information on benefits or harms (n=56)

Akarırmak Ü, Koçyiğit H, Eskiyurt N, Esmaeilzadeh S, Kuru Ö, Yalçinkaya EY, et al. Influence of patient training on persistence, compliance, and tolerability of different dosing frequency regimens of bisphosphonate therapy: an observational study in Turkish patients with postmenopausal osteoporosis. Acta Orthop Traumatol Turc. 2016;50(4):415-23.

Ambahe G. Implementation of an osteoporosis risk assessment and patient education protocol in primary care. Diss Abstr Int. 2020;81(6-A):No-Specified.

Aree-Ue S, Pothiban L, Belza B, Sucamvang K, Panuthai S. Osteoporosis preventive behavior in Thai older adults: feasibility and acceptability. J Gerontol Nurs. 2006;32(7):23-30.

Baseer A, Raqeebuddin MA, Salman S, Badri V, Lankeswar Rao K. Community based screening and intervention to optimize preventive care of osteoporosis. J Pharm Sci. 2020;12(11):1437-47.

Bianchi ML, Duca P, Vai S, Guglielmi G, Viti R, Battista C, et al. Improving adherence to and persistence with oral therapy of osteoporosis. Osteoporos Int. 2015;26(5):1629-38.

Brask-Lindemann D, Cadarette SM, Eskildsen P, Abrahamsen B. Osteoporosis pharmacotherapy following bone densitometry: importance of patient beliefs and understanding of DXA results. Osteoporos Int. 2011;22(5):1493-501.

Cram P, Schlechte J, Christensen A. A randomized trial to assess the impact of direct reporting of DXA scan results to patients on quality of osteoporosis care. J Clin Densitom. 2006;9(4):393-8.

Dashkova I, Dashkova A, Kozikowski A, Gupta A, Sison C, Ibrahim R, et al. Differences according to sex in perspectives and beliefs on osteoporosis: a cross-sectional survey study. J Am Geriatr Soc. 2016;64(10):e85-e6.

DeJesus RS, Chaudhry R, Angstman KB, Cha SS, Tulledge-Scheitel SM, Kesman RL, et al. Predictors of osteoporosis screening completion rates in a primary care practice. Popul Health Manag. 2011;14(5):243-7.

Dugard MN, Jones TJ, Davie MWJ. Uptake of treatment for osteoporosis and compliance after bone density measurement in the community. J Epidemiol Community Health. 2010;64(6):518-22.

Gaines JM, Narrett M, Parrish JM. The effect of the addition of osteoporosis education to a bone health screening program for older adults. Geriatr Nurs. 2010;31(5):348-60.

Goldshtein I, Shamai-Lubovitz O, Guindy M, Shalev V, Chodick G, Lerner U, et al. Development and efficacy of a computerized decision support system for osteoporosis management in the community. Arch Osteoporos. 2020;15(1):27.

Gonnelli S, Caffarelli C, Rossi S, Di Munno O, Malavolta N, Isaia G, et al. How the knowledge of fracture risk might influence adherence to oral therapy of osteoporosis in Italy: the ADEOST study. Aging Clin Exp Res. 2016;28(3):459-68.

Guilera M, Fuentes M, Grifols M, Ferrer J, Badia X. Does an educational leaflet improve self-reported adherence to therapy in osteoporosis? The OPTIMA study. Osteoporos Int. 2006;17(5):664-71.

Hall S, Edmonds S, Lou Y, Cram P, Roblin D. Reasons for patient non-adherence to recommended osteoporosis pharmacotherapy. J Bone Miner Res. 2015;30:S170.

Hall SF, Edmonds SW, Lou Y, Cram P, Roblin DW, Saag KG, et al. Patient-reported reasons for nonadherence to recommended osteoporosis pharmacotherapy. J Am Pharm Assoc. 2017;57(4):503-9.

Heyworth L, Kleinman K, Oddleifson S, Bernstein L, Frampton J, Lehrer M, et al. Comparison of interactive voice response, patient mailing, and mailed registry to encourage screening for osteoporosis: a randomized controlled trial. Osteoporos Int. 2014;25(5):1519-26.

Heyworth L, Kleinman K, Oddleifson S, Bernstein L, Frampton J, Salvato K, et al. Screening for osteoporosis in high risk, menopausal women: a randomized trial of interactive voice response. J Gen Intern Med. 2011;26:S207.

Hoiberg MP, Rubin KH, Holmberg T, Rothmann MJ, Moller S, Gram J, et al. Use of antiosteoporotic medication in the

Danish ROSE population-based screening study. Osteoporos Int. 2019;30(6):1223-33.
Jones ASK, Fernandez J, Grey A, Petrie KJ. The impact of 3-D models versus animations on perceptions of osteoporosis and treatment totivation: a randomised trial. Ann Behav Med. 2017;51(6):899-911.
Jones SMW, Gell NM, Roth JA, Scholes D, LaCroix AZ. The relationship of perceived risk and biases in perceived risk to fracture prevention behavior in older women. Ann Behav Med. 2015;49(5):696-703.
Kalisch Ellett LM, Pratt NL, Sluggett JK, Ramsay EN, Kerr M, LeBlanc VT, et al. Patient-specific prescriber feedback can increase the rate of osteoporosis screening and treatment: results from two national interventions. Arch Osteoporos. 2017;12(1):17.
Kastner M, Sawka AM, Hamid J, Chen M, Thorpe K, Chignell M, et al. A knowledge translation tool improved osteoporosis disease management in primary care: an interrupted time series analysis. Implement Sci. 2014;9:109.
Kingwell E. The effects of bone mineral density testing on health related behaviours in a randomly selected population of Canadians. Dissertation Abstracts International: Section B: The Sciences and Engineering. 2008;68(10-B):6615.
Lacroix AZ, Buist DS, Brenneman SK, Abbott TA. Evaluation of three population-based strategies for fracture prevention: results of the osteoporosis population-based risk assessment (OPRA) trial. Med Care. 2005;43(3):293-302.
Law AV, Shapiro K. Impact of a community pharmacist-directed clinic in improving screening and awareness of osteoporosis. J Eval Clin Pract. 2005;11(3):247-55.
Levinson JB, Jina N, Beldowski K, Hwang C, Zirker W. Patient barriers to bisphosphonate treatment. J Am Geriatr Soc. 2019;67(Suppl 1):S108.
McCloskey E, Rathi J, Heijmans S, Blagden M, Koukakis R, O'Kelly J, et al. Osteoporosis (OP) diagnosis and treatment of women aged >=70 years in primary care: Results from a large Europeal cross-sectional study. Swiss Med Wkly. 2019;149(Suppl 238):10S.
Mora AN, Blazar PE, Rogers JC, Earp BE. Patient Perceptions and Preferences for Osteoporosis Treatment. J Hand Surg. 2020;45(2):153.
Nayak S, Roberts MS, Greenspan SL. Osteoporosis screening preferences of older adults. J Clin Densitom. 2009;12(3):279-86.
Nguyen T, Edmonds S, Solimeo S, Wolinsky F, Roblin D, Saag K, et al. Sex and racial differences of osteo porosis knowledge among patients presenting for DXA. Osteoporos Int. 2013;24:S436-S7.
Polinski JM, Cadarette SM, Arnold M, Katz JN, Finkelstein JS, Brookhart MA, et al. High-risk patients' readiness to undergo BMD testing for osteoporosis diagnosis in Pennsylvania. Int Q Community Health Educ. 2008;29(3):223-40.
Pressman A, Forsyth B, Ettinger B, Tosteson AN. Initiation of osteoporosis treatment after bone mineral density testing. Osteoporos Int. 2001;12(5):337-42.
Puvanendran R, Lee MLJJ, Chua MT, Cheng SJ, Ang SB. Primary prevention of osteoporotic fractures in post menopausal women - utilizing structured patient education and case managers to improve adherence. Giorn It Ost Gin. 2014;36(1):250-3.
Richards JB, Cherkas LF, Spector TD. An analysis of which anti-osteoporosis therapeutic regimen would improve compliance in a population of elderly adults. Curr Med Res Opin. 2007;23(2):293-9.
Roh YH, Lee ES, Ahn J, Kim HS, Gong HS, Baek KH, et al. Factors affecting willingness to get assessed and treated for osteoporosis. Osteoporos Int. 2019;30(7):1395-401.
Rukshini P. Can a structured program of patient education and follow up with case managers prevent osteoporotic fractures in post menopausal women? Med J Malaysia. 2017;72(Suppl 1):52.
Saag KG, Mudano AS, Rahn EJ, Wright NC, Outman RC, Ladores S, et al. Factors associated with the contemplative stage of readiness to initiate osteoporosis treatment. Osteoporos Int. 2020;31(7):1283-90.
Seuffert P, Sagebien CA, McDonnell M, O'Hara DA. Evaluation of osteoporosis risk and initiation of a nurse practitioner intervention program in an orthopedic practice. Arch Osteoporos. 2016;11:10.

Shu AD, Stedman MR, Polinski JM, Jan SA, Patel M, Truppo C, et al. Adherence to osteoporosis medications after patient and physician brief education: post hoc analysis of a randomized controlled trial. Am J Manag Care. 2009;15(7):417-24.

Siris ES, Gehlbach S, Adachi JD, Boonen S, Chapurlat RD, Compston JE, et al. Failure to perceive increased risk of fracture in women 55 years and older: the Global Longitudinal Study of Osteoporosis in Women (GLOW). Osteoporos Int. 2011;22(1):27-35.

Solomon DH, Brookhart MA, Polinski J, Katz JN, Cabral D, Licari A, et al. Osteoporosis action: design of the healthy bones project trial. Contemp Clin Trials. 2005;26(1):78-94.

Solomon DH, Finkelstein JS, Polinski JM, Arnold M, Licari A, Cabral D, et al. A randomized controlled trial of mailed osteoporosis education to older adults. Osteoporos Int. 2006;17(5):760-7.

Solomon DH, Katz JN, Finkelstein JS, Polinski JM, Stedman M, Brookhart MA, et al. Osteoporosis improvement: a large-scale randomized controlled trial of patient and primary care physician education. J Bone Miner Res. 2007;22(11):1808-15.

Stephens MH, Grey A, Fernandez J, Kalluru R, Faasse K, Horne A, et al. 3-D bone models to improve treatment initiation among patients with osteoporosis: a randomised controlled pilot trial. Psychol Health. 2016;31(4):487-97.

Sujic R, Beaton D, Bogoch E. Patient acceptance of osteoporosis treatment: application of the stages of change model. Maturitas. 2016;88:70-5.

Umar A, Olanipenkun T, Kwong HS, Harris C, Bilal M, Ivonye C, et al. Osteoporosis screening in a resident-driven clinic of a large academic hospital. Arthritis Rheum. 2019;71(Supplement 10):3926-7.

Waalen JB, A. L.; Peters, M. J.; Blau, E. M. A telephone-based intervention for increasing the use of osteoporosis medication: a randomized controlled trial. Am J Manag Care. 2009;15(8):e60-70.

Walsh ME, Nerdrum M, Fahey T, Moriarty F. Factors associated with initiation of bone-health medication among older adults in primary care in Ireland. Age Ageing. 2021.

Warriner AH, Outman RC, Feldstein AC, Roblin DW, Allison JJ, Curtis JR, et al. Effect of self-referral on bone mineral density testing and osteoporosis treatment. Med Care. 2014;52(8):743-50.

Warriner AH, Outman RC, Kitchin E, Chen L, Morgan S, Saag KG, et al. A randomized trial of a mailed intervention and self-scheduling to improve osteoporosis screening in postmenopausal women. J Bone Miner Res. 2012;27(12):2603-10.

Weiss TW, McHorney CA. Osteoporosis medication profile preference: Results from the PREFER-US study. Health Expect. 2007;10(3):211-23.

Wu F, Laslett LL, Wills K, Oldenburg B, Jones G, Winzenberg T. Effects of individualized bone density feedback and educational interventions on osteoporosis knowledge and self-efficacy: a 12-yr prospective study. J Clin Densitom. 2014;17(4):466-72.

Yood RA, Mazor KM, Andrade SE, Emani S, Chan W, Kahler KH. Patient decision to initiate therapy for osteoporosis: the influence of knowledge and beliefs. J Gen Intern Med. 2008;23(11):1815-21.

Yuksel N, Majumdar SR, Biggs C, Tsuyuki RT. Community pharmacist-initiated screening program for osteoporosis: randomized controlled trial. Osteoporosis Int. 2010;21(3):391-8.

Yuksel N, Tsuyuki RT, Majumdar SR. Predictors of bone mineral density testing in patients at high risk of osteoporosis: secondary analyses from the OSTEOPHARM randomized trial. J Clin Densitom. 2012;15(1):61-6.

<u>Ineligible comparator:</u>

KQ1 (n=1)

Crandall C, Larson J, LaCroix A, Cauley J, LeBoff M, Li W, et al. Screening young postmenopausal women for osteoporosis: comparison of hip fracture risk prediction by the garvan and FRAX risk calculators in the women's health initiative observational study and clinical trials. J Bone Miner Res. 2017;32 (Suppl 1):S249.

KQ3a

Not placebo (n=32)

Atmaca A, Gedik O. Effects of alendronate and risedronate on bone mineral density and bone turnover markers in late postmenopausal women with osteoporosis. Adv Ther. 2006;23:842-53.

Bai H, Jing D, Guo A, Yin S. Randomized controlled trial of zoledronic acid for treatment of osteoporosis in women. J Int Med Res. 2013;41(3):697-704.

Black D, Reid I, Boonen S, Bucci-Rechtweg C, Cauley J, Cosman F, et al. The effect of 3 versus 6 years of zoledronic acid treatment of osteoporosis: a randomized extension to the HORIZON-Pivotal Fracture Trial (PFT). J Bone Miner Res. 2012;27(2):243-54.

Black D, Reid I, Cauley J, Cosman F, Leung P, Lakatos P, et al. The effect of 6 versus 9 years of zoledronic acid treatment in osteoporosis: a randomized second extension to the HORIZON-Pivotal Fracture Trial (PFT). J Bone Miner Res. 2015;30(5):934-44.

Bliuc D, Tran T, Van Geel T, Eisman JA, Nguyen TV, Langsetmo L, et al. The effect of bisphosphonates on all-cause and post-fracture mortality risk in the population-based Canadian multicentre osteoporosis study (CaMos). J Bone Miner Res. 2016;31(Suppl 1):S3.

Bone HG, Brandi ML, Brown JP, Chapurlat R, Cummings SR, Czerwinski E, et al. Ten years of denosumab (DMAB) treatment in postmenopausal women with osteoporosis: results from the Freedom Extension trial. Osteoporos Int. 2016;27(SUPPL. 1):S135-S6.

Bonnick S, Broy S, Kaiser F, Teutsch C, Rosenberg E, DeLucca P, et al. Treatment with alendronate plus calcium, alendronate alone, or calcium alone for postmenopausal low bone mineral density. Curr Med Res Opin. 2007;23(6):1341-9.

Boonen S, Lorenc R, Wenderoth D, Stoner K, Eusebio R, Orwoll E. Evidence for safety and efficacy of risedronate in men with osteoporosis over 4 years of treatment: results from the 2-year, open-label, extension study of a 2-year, randomized, double-blind, placebo-controlled study. Bone. 2012;51(3):383-8.

Brown JP, Kendler DL, McClung MR, Emkey RD, Adachi JD, Bolognese MA, et al. The efficacy and tolerability of risedronate once a week for the treatment of postmenopausal osteoporosis. Calcif Tissue Int. 2002;71(2):103-11.

Chang S, Bauer D, Black D. Change in bone mineral density (BMD) or bone turnover markers (BTM) did not predict risk of vertebral fracture after discontinuation ofalendronate in the flex study. Osteoporos Int. 2017;28(Suppl 1):S377.

Chao M, Hua Q, Yingfeng Z, Guang W, Shufeng S, Yuzhen D, et al. Study on the role of zoledronic acid in treatment of postmenopausal osteoporosis women. Pak J Med Sci. 2013;29(6):1381-4.

ClinicalTrials.gov [Internet]. Bethesda (MD): National Library of Medicine (US). 2000 Feb 29. Identifier NCT00729651, Efficacy and Safety Study of Fosamax Plus D in Postmenopausal Women With Osteoporosis (0217A-263); 2008 Aug 7 [cited 2020 Jul 27]. Available from: https://clinicaltrials.gov/ct2/show/NCT00729651.

ClinicalTrials.gov [Internet]. Bethesda (MD): National Library of Medicine (US). 2000 Feb 29. Identifier NCT00097825, Efficacy and Safety of Zoledronic Acid for the Treatment of Osteoporosis in Men; 2004 Dec 1 [cited 2020 Jul 27]. Available from: https://clinicaltrials.gov/ct2/show/NCT00097825.

Delaney M, Hurwitz S, Shaw J, LeBoff M. Bone density changes with once weekly risedronate in postmenopausal women. J Clin Densitom. 2003; 6(1):45-50.

Eastell R, Vrijens B, Cahall DL, Ringe JD, Garnero P, Watts NB. Bone turnover markers and bone mineral density response with risedronate therapy: relationship with fracture risk and patient adherence. J Bone Miner Res. 2011;26(7):1662-9.

Gonnelli S, Cepollaro C, Montagnani A, Bruni D, Caffarelli C, Breschi M, et al. Alendronate treatment in men with primary osteoporosis: a three-year longitudinal study. Calcif Tissue Int. 2003;73(2):133-9.

Grey A, Horne A, Gamble G, Mihov B, Reid IR, Bolland M. Ten years of very infrequent zoledronate therapy in older women: an open-label extension of a randomized trial. J Clin Endocrinol Metab. 2020;105(4):1-7.

Gu JM, Wang L, Lin H, Chen DC, Tang H, Jin XL, et al. The efficacy and safety of weekly 35-mg risedronate dosing regimen for Chinese postmenopausal women with osteoporosis or osteopenia: 1-year data. Acta Pharmacol Sin. 2015;36(7):841-6.

Ho A, Kung A. Efficacy and tolerability of alendronate once weekly in Asian postmenopausal osteoporotic women. Ann Pharmacother. 2005;39(9):1428-33.

Hwang JS, Liou MJ, Ho C, Lin JD, Huang YY, Wang CJ, et al. The effects of weekly alendronate therapy in Taiwanese males with osteoporosis. J Bone Miner Metab. 2010;28(3):328-33.
McClung MR, Balske A, Burgio DE, Wenderoth D, Recker RR. Treatment of postmenopausal osteoporosis with delayed-release risedronate 35 mg weekly for 2 years. Osteoporos Int. 2013;24(1):301-10.
McClung M, Clemmesen B, Daifotis A, Gilchrist NL, Eisman J, Weinstein RS, et al. Alendronate prevents postmenopausal bone loss in women without osteoporosis. A double-blind, randomized, controlled trial. Alendronate Osteoporosis Prevention Study Group. Ann Intern Med. 1998;128(4):253-61.
McClung MR, Lewiecki EM, Geller ML, Bolognese MA, Peacock M, Weinstein RL, et al. Effect of denosumab on bone mineral density and biochemical markers of bone turnover: 8-year results of a phase 2 clinical trial. Osteoporos Int. 2013;24(1):227-35.
Muscoso E, Puglisi N, Mamazza C, Lo Giudice F, Testai M, Abbate S, et al. Antiresorption therapy and reduction in fracture susceptibility in the osteoporotic elderly patient: open study. Eur Rev Med Pharmacol Sci. 2004;8(2):97-102.
Naranjo JFT, Garcia-Hernandez P, Flores-Moreno C, De La Pena-Rodriguez P, Gutierrez-Hermosillo H, Gonzalez-Mendoza R, et al. Bone mineral density changes in compliant and adherent denosumab treated patients over a 24 months period in real clinical setting. Two years preliminary data. Calcif. Tissue Int. 2017;100(1 Suppl 1):S77.
Ravn P, Weiss SR, Rodriguez-Portales JA, McClung MR, Wasnich RD, Gilchrist NL, et al. Alendronate in early postmenopausal women: effects on bone mass during long-term treatment and after withdrawal. Alendronate Osteoporosis Prevention Study Group. J Clin Endocrinol Metab. 2000;85(4):1492-7.
Ringe JD, Dorst A, Faber H, Ibach K. Alendronate treatment of established primary osteoporosis in men: 3-year results of a prospective, comparative, two-arm study. Rheumatol Int. 2004;24(2):110-3.
Ringe JD, Faber H, Dorst A. Alendronate treatment of established primary osteoporosis in men: results of a 2-year prospective study. J Clin Endocrinol Metab. 2001;86(11):5252-5.
Ringe JD, Faber H, Farahmand P, A D. Efficacy of risedronate in men with primary and secondary osteoporosis: results of a 1-year study. Rheumatol Int. 2006;26(5):427-31.
Ringe JD, Farahmand P, Faber H, A D. Sustained efficacy of risedronate in men with primary and secondary osteoporosis: results of a 2-year study. Rheumatol Int. 2009;29(3):311-5.
Rizzoli R, Greenspan SL, Bone G, 3rd, Schnitzer TJ, Watts NB, Adami S, et al. Two-year results of once-weekly administration of alendronate 70 mg for the treatment of postmenopausal osteoporosis. J Bone Miner Res. 2002;17(11):1988-96.
Sugimoto T, Matsumoto T, Hosoi T, Miki T, Gorai I, Yoshikawa H, et al. Three-year denosumab treatment in postmenopausal Japanese women and men with osteoporosis: results from a 1-year open-label extension of the Denosumab Fracture Intervention Randomized Placebo Controlled Trial (DIRECT). Osteoporos Int. 2015;26(2):765-74.

Inadequate length of follow-up (<6 months; n = 2)

Ilter E, Karalok H, Tufekci EC, Batur O. Efficacy and acceptability of risedronate 5 mg daily compared with 35 mg once weekly for the treatment of postmenopausal osteoporosis. Climacteric. 2006;9(2):129-34.
Nagashima H, Hirayama M, Ohishi H, Oikawa I. Optimizing a single monthly dose of risedronate based on its weekly dose in healthy Japanese postmenopausal women. Jpn J Clin Pharmacol Ther. 2017;48(4):121-9.

KQ3b (n=10)

Aljohani S, Fliefel R, Ihbe J, Kuhnisch J, Ehrenfeld M, Otto S. What is the effect of anti-resorptive drugs (ARDs) on the development of medication-related osteonecrosis of the jaw (MRONJ) in osteoporosis patients: a systematic review. J Craniomaxillofac Surg. 2017;45:1493-502.
Aljohani S, Fliefel R, Otto S, Ehrenfeld M, Kühnisch J, Ihbe J. Medication-related osteonecrosis of the jaw associated with antiresorpative drugs in osteoporosis: a systematic review protocol. PROSPERO [Internet]. 2016 Nov 22 [cited 2020 Nov 4]. Available from: https://www.crd.york.ac.uk/prospero/display_record.php?ID=CRD42016052011.
Aulestia-Viera P, Rocha A, Santos-Silva A, Machado G. Medication-related osteonecrosis of the jaw in denosumab users: systematic review. PROSPERO [Internet]. 2020 Apr 28 [cited 2020 Nov 4]. Available from: https://www.crd.york.ac.uk/prospero/display_record.php?ID=CRD42020153720.

Beaudoin C, Jean S, Bessette L, Ste-Marie LG, Moore L, Brown JP. Denosumab compared to other treatments to prevent or treat osteoporosis in individuals at risk of fracture: a systematic review and meta-analysis. Osteoporos Int. 2016;27:2835-44.

Benjamin B, Benjamin M, Swe M, Sugathan S. Systematic review on the comparison of effectiveness between denosumab and bisphosphonates in post-menopausal osteoporosis. PROSPERO [Internet]. 2015 Apr 15 [cited 2020 Nov 4]. Available from: https://www.crd.york.ac.uk/prospero/display_record.php?ID=CRD42015019464.

Fliefel R, Troltzsch M, Kuhnisch J, Ehrenfeld M, Otto S. Treatment strategies and outcomes of bisphosphonate-related osteonecrosis of the jaw (BRONJ) with characterization of patients: a systematic review. Int J Oral Maxillofac Surg. 2015;44:568-85.

McGowan K, McGowan T, Ivanovski S. Systematic review of medical and dental risk factors associated with Osteonecrosis of the Jaws (ONJ). PROSPERO Record.

McGowan K, McGowan T, Ivanovski S. Risk factors for medication-related osteonecrosis of the jaws: a systematic review. Oral Dis. 2018;24:527-36.

Wang T, Xuan Z, Li R, Song L, Dou Y, Ren J, et al. Efficacy and safety of denosumab and teriparatide treatment for osteoporosis: a systematic review and meta-analysis. Int J Clin Exp Med. 2017;10:5949-56.

Wu J, Zhang Q, Yan G, Jin X. Denosumab compared to bisphosphonates to treat postmenopausal osteoporosis: a meta-analysis. J Orthop Surg Res. 2018;13:194.

No outcomes of interest:

KQ1 (n=17)

Buist DS, LaCroix AZ, Manfredonia D, Abbott T. Identifying postmenopausal women at high risk of fracture in populations: a comparison of three strategies. J Am Geriatr Soc. 2002;50(6):1031-8.

Condurache C, Shepstone L, Cooper C, Harvey N, Kanis J, Johansson H, et al. Does screening for high hip fracture risk impact on falls risk? A post hoc analysis from the SCOOP study. Calcif Tiss Int. 104(Suppl 1):S19.

Condurache CI, Chiu S, Chotiyarnwong P, Johansson H, Shepstone L, Lenaghan E, et al. Screening for high hip fracture risk does not impact on falls risk: a post hoc analysis from the SCOOP study. Osteoporos Int. 2020;31(3):457-64.

Cooper C, Parsons C, Harvey NC, Shepstone L, Kanis JA, Lenaghan E, et al. Effect of fracture risk assessment on anti-osteoporosis medication use and adherence: findings from the scoop trial. J Bone Miner Res. 2017;32:S108.

Cooper C, Pearosn C, Harvey NC, Shepstone L, Kanis JA, Lenaghan E, et al. Effect of fracture risk assessment on anti-osteoporosis medication use and adherence: findings from the scoop trial. J Bone Miner Res. 2017;32(Suppl 1):S108.

Elders PJ, Merlijn T, Swart KM, Van Hout W, Van der Zwaard BC, Niemeijer C, et al. Design of the SALT Osteoporosis Study: a randomised pragmatic trial, to study a primary care screening and treatment program for the prevention of fractures in women aged 65 years or older. BMC Musculoskelet Disord. 2017;18(1):424.

Høiberg MP, Rubin KH, Holmberg T, Rothmann MJ, Möller S, Gram J, et al. Use of antiosteoporotic medication in the Danish ROSE population-based screening study. Osteoporos Int. 2019;30(6):1223-33.

McCloskey E, Chiu S, Lenaghan E, Shepstone L, Birrell F, Harvey N. Impact of falls on fractures and mortality-an opportunity for intervention and enhancement of fracture prediction?. Osteoporos Int. 2016;27(Suppl 2):S617.

NCT registry [internet]. Wiser Choices in Osteoporosis Choice II: A Decision Aid for Patients and Clinicians. Available from: https://clinicaltrialsgov/show/NCT00949611. 2009.

NCT registry [internet]. Understanding and Discouraging Overuse of Potentially Harmful Screening Tests. Available from: https://clinicaltrialsgov/show/NCT01694784. 2012.

Parsons C, Harvey N, Shepstone L, Kanis JA, Lenaghan E, Clarke S, et al. Systematic fracture risk screening using FRAX results in greater use of, and adherence to, anti-osteoporosis medications: the UK SCOOP Trial. JBMR Plus. 2018;2(Suppl 1):S6-S7.

Parsons C, Harvey N, Shepstone L, Kanis J, Lenaghan E, Clarke S, et al. Systematic screening using FRAX leads to increased use of, and adherence to, antiosteoporosis medications: the UK scoop trial. Osteoporos Int. 2018;29(Suppl 2):S613.

Parsons C, Harvey N, Shepstone L, Kanis J, Lenaghan E, Clarke S, et al. Systematic screening using FRAX leads to increased

use of, and adherence to, antiosteoporosis medications: the UK scoop trial. Osteoporos Int. 2018;29:S613.
Parsons CM, Harvey N, Shepstone L, Kanis JA, Lenaghan E, Clarke S, et al. Systematic screening using FRAX R leads to increased use of, and adherence to, anti-osteoporosis medications: an analysis of the UK SCOOP trial. Osteoporos Int. 2020;31(1):67-75.
Rubin KH, Holmberg T, Rothmann MJ, Høiberg M, Barkmann R, Gram J, et al. The risk-stratified osteoporosis strategy evaluation study (ROSE): a randomized prospective population-based study. Design and baseline characteristics. Calcif Tissue Int. 2015;96(2):167-79.
Soreskog E, Borgstrom F, Shepstone L, Clarke S, Cooper C, Harvey I, et al. Long-term cost-effectiveness of screening for fracture risk in a UK primary care setting: the SCOOP study. Osteoporos Int. 2020;31(8):1499-506.
Women's Health Initiative Study Group. Design of the Womens Health Initiative clinical trial and observational study. Control Clin Trials. 1998;19(1):61-109.

KQ2

No calibration outcomes (n=85)

Adami G, Gatti D, Rossini M, Orsolini G, Pollastri F, Bertoldo E, et al. Risk of fragility fractures in obesity and diabetes: a retrospective analysis on a nation-wide cohort. Osteoporos Int. 2020;31(11):2113-22.
Alajlouni D, Bliuc D, Tran T, Pocock N, Nguyen TV, Eisman JA, et al. Nonstandard lumbar region in predicting fracture risk. J Clin Densitom. 2018;21(2):220-6.
Albaba M. Modified Hip Fracture Risk Assessment Index (MHFRAI), an algorithm of demographic risk factors, can identify risk for osteoporotic hip fracture in community dwelling older adults. J Am Geriatr Soc. 2016;64(Suppl 1):S96.
Alibasic E, Ljuca F, Brkic S, Fazlic M, Husic D. Secondary Prevention of Osteoporosis Through Assessment of Individual and Multiple Risk Factors. Materia socio-medica. 2020;32(1):10-4.
Amin S, Atkinson E, Khosla S. Identifying women and men at high fracture risk by leveraging the electronic medical records to estimate FRAX treatment thresholds. Ann Rheum Dis. 2017;76 (Suppl 2):705-6.
Billington EO, Gamble GD, Reid IR. Reasons for discrepancies in hip fracture risk estimates using FRAX and Garvan calculators. Maturitas. 2016;85:11-8.
Black DM, Steinbuch M, Palermo L, Dargent-Molina P, Lindsay R, Hoseyni MS, et al. An assessment tool for predicting fracture risk in postmenopausal women. Osteoporos Int. 2001;12(7):519-28.
Boutroy S, Hans D, Sornay-Rendu E, Vilayphiou N, Winzenrieth R, Chapurlat R. Trabecular bone score improves fracture risk prediction in non-osteoporotic women: the OFELY study. Osteoporos Int. 2013;24(1):77-85.
Buist DSM, LaCroix AZ, Manfredonia D, Abbott T. Identifying postmenopausal women at high risk of fracture in populations: a comparison of three strategies. J Am Geriatr Soc. 2002;50(6):1031-8.
Cetin B, Cetin EA, Arikan H, Velioglu A, Alibaz-Oner F, Direskeneli H, et al. FRAX scores are increased in patients with ANCA-associated vasculitis. Int Urol Nephrol. 2021.
Chen P, Krege JH, Adachi JD, Prior JC, Tenenhouse A, Brown JP, et al. Vertebral fracture status and the World Health Organization risk factors for predicting osteoporotic fracture risk. J Bone Miner Res. 2009;24(3):495-502.
Chun SH, Cho B, Yang HK, Ahn E, Han MK, Oh B, et al. Performance on physical function tests and the risk of fractures and admissions: findings from a national health screening of 557,648 community-dwelling older adults. Arch Gerontol Geriatr. 2017;68:174-80.
Clark P, Denova-Gutierrez E, Zerbini C, Sanchez A, Messina O, Jaller JJ, et al. FRAX-based intervention and assessment thresholds in seven Latin American countries. Osteoporos Int. 2018;29(3):707-15.
Colon-Emeric CS, Pieper CF, Artz MB. Can historical and functional risk factors be used to predict fractures in community-dwelling older adults? Development and validation of a clinical tool. Osteoporos Int. 2002;13(12):955-61.
Couris CM, Chapurlat RD, Kanis JA, Johansson H, Burlet N, Delmas PD, et al. FRAX® probabilities and risk of major osteoporotic fracture in france. Osteoporos Int. 2012;23(9):2321-7.
Crandall CJ, Larson J, Manson JE, Cauley JA, LaCroix AZ, Wactawski-Wende J, et al. A comparison of US and Canadian osteoporosis screening and treatment strategies in postmenopausal women. J Bone Miner Res. 2019;34(4):607-15.
Crandall CJ, Larson J, Manson JE, Cauley JA, LaCroix AZ, Wactawski-Wende J, et al. A Comparison of US and Canadian Osteoporosis Screening and Treatment Strategies in Postmenopausal Women. J Bone Miner Res. 2019;34(4):607-15.

Diem SJ, Peters KW, Gourlay ML, Schousboe JT, Taylor BC, Orwoll ES, et al. Screening for osteoporosis in older men: operating characteristics of proposed strategies for selecting men for BMD testing. J Gen Intern Med. 2017;32(11):1235-41.

Esmaeilzadeh S, Cesme F, Oral A, Yaliman A, Sindel D. The utility of dual-energy X-ray absorptiometry, calcaneal quantitative ultrasound, and fracture risk indices (FRAX® and Osteoporosis Risk Assessment Instrument) for the identification of women with distal forearm or hip fractures: a pilot study. Endocrine Research. 2016;41(3):248-60.

Ettinger B, Black DM, Dawson-Hughes B, Pressman AR, Melton LJ, 3rd. Updated fracture incidence rates for the US version of FRAX. Osteoporos Int. 2010;21(1):25-33.

Ferrari S, Rizzoli R, Chapurlat R, Brandi ML, Martinez H, Herrero M, et al. OP0294 Fracture prediction using a genetic markers algorithm compared to FRAX in three European cohorts. Ann Rheum Dis. 2017;76(Suppl 2):178.

Fujiwara S, Nakamura T, Orimo H, Hosoi T, Gorai I, Oden A, et al. Development and application of a Japanese model of the WHO fracture risk assessment tool (FRAX). Osteoporos Int. 2008;19(4):429-35.

Gadam RK, Schlauch K, Izuora KE. FRAX prediction without BMD for assessment of osteoporotic fracture risk. Endocr Pract. 2013;19(5):780-4.

Galassi A, Martin-Guerrero JD, Villamor E, Monserrat C, Ruperez MJ. Risk Assessment of Hip Fracture Based on Machine Learning. Appl Bionics Biomech. 2020;2020:8880786.

Garton M. Comparative performance of frax and qfracture in a real-world setting. Osteoporos Int. 2018;29(Suppl 2):S638.

Gladkova EN, Lesnyak OM, Anoshenkova ON, Tavluev VA, Belova KY, Belousova IB, et al. Application of FRAX for the development of specific intervention thresholds in osteoporosis treatment in Russian federation. Osteoporos Int. 2019;30(Suppl 2):S627-S8.

Glaser Y, Sadowski P, Wolfgruber T, Shepherd J, Lui L-Y, Cummings S. Hip Fracture Risk Modeling using DXA and Artificial Intelligence. J Bone Miner Res. 2020;35(Suppl 1):200.

Goldshtein I, Ish-Shalom S, Leshno M. Impact of FRAX-based osteoporosis intervention using real world data. Bone. 2017;103:318-24.

Goldshtein I, Leshno M, Ish-Shalom S, Gerber Y. Application of FRAX in Israel: validation and impact assessment using real-world medical databases. Osteoporos Int. 2016;27(Suppl 1):S414.

Hamdy RC, Kiebzak GM. Variance in 10-year fracture risk calculated with and without T-scores in select subgroups of normal and osteoporotic patients. J Clin Densitom. 2009;12(2):158-61.

Henry MJ, Pasco JA, Merriman EN, Zhang Y, Sanders KM, Kotowicz MA, et al. Fracture risk score and absolute risk of fracture. Radiology. 2011;259(2):495-501.

Ho-Le TP, Tran HTT, Center JR, Eisman JA, Nguyen HT, Nguyen TV. Assessing the clinical utility of genetic profiling in fracture risk prediction: a decision curve analysis. Osteoporos Int. 2021;32(2):271-80.

Hoggard K, Hart S, Birchall J, Kirk S, Goff I, Grove M, et al. Fracture prevention: a population-based intervention delivered in primary care. QJM. 2020;113(5):313-9.

Inderjeeth C, Raymond W. GARVAN fracture risk calculator without BMD is accurate and reduces reliance on BMD in older people with poor access. Int J Rheum Dis. 2019;22(Suppl 3):94-5.

Inderjeeth C, Raymond W. Utilising GARVAN fracture risk calculator without BMD to identify who to treat vs. who to investigate with BMD in people over 70 years of age. Osteoporos Int. 2017;28(Suppl 1):S239-S40.

Inderjeeth C, Raymond W. GARVAN fracture risk calculator without BMD is accurate and reduces reliance on BMD in older people with poor access. Int J Rheum Dis. 2019;22(Suppl 3):94-5.

Inderjeeth CA, Raymond WD. Case finding with GARVAN fracture risk calculator in primary prevention of fragility fractures in older people. Arch Gerontol Geriatr. 2020;86:103940.

Jiang X, Westermann LB, Galleo GV, Demko J, Marakovits KA, Schnatz PF. Age as a predictor of osteoporotic fracture compared with current risk-prediction models. Obstet Gynecol. 2013;122(5):1040-6.

Johansson H, Azizieh F, Al Ali N, Alessa T, Harvey NC, McCloskey E, et al. FRAX- vs. T-score-based intervention thresholds for osteoporosis. Osteoporos Int. 2017;28(11):3099-105.

Johansson H, Oden A, McLoskey E, Hans D, Kanis J. Improved risk assessment using lumbar spine trabecular bone score (TBS) to adjust fracture probability: the Manitoba BMD cohort. J Bone Miner Res. 2015;30(Suppl 1):S103.

Kalvesten J, Lui LY, Brismar TB, Cummings SR. Association of digital X-ray radiogrammetry with FRAX and dual-energy X-ray absorptiometry: the study of osteoporotic fractures. Osteoporos Int. 2016;27(Suppl 1):S113.

Kanis JA, Johansson H, Oden A, Dawson-Hughes B, Melton LJ 3rd, McCloskey EV. The effects of a FRAX revision for the USA. Osteoporos Int. 2010;21(1):35-40.

Kanis JA, Oden A, Johnell O, Johansson H, De Laet C, Brown J, et al. The use of clinical risk factors enhances the performance of BMD in the prediction of hip and osteoporotic fractures in men and women. Osteoporos Int. 2007;18(8):1033-46.

Kharroubi A, Saba E, Ghannam I, Darwish H. Evaluation of the validity of osteoporosis and fracture risk assessment tools (IOF One Minute Test, SCORE, and FRAX) in postmenopausal Palestinian women. Arch of Osteoporos. 2017;12(1):6.

Kim BY, Choi ST, Kim SH, Kwon SR, Suh CH. Validation of FRAX for osteoporosis treatment decision in patients with osteoarthritis: a multicenter comparative study of FRAX and WHO criteria. Int J Rheum Dis. 2018;21(Suppl 1):107.

Kim H, Kim JH, Kim MJ, Hong AR, Lee JH, Ku E, et al. Predictive value of FRAX adjusted for trabecular bone score for osteoporotic fracture in Korean: a community-based cohort study. Endocr Rev. 2018;39(Suppl 1).

Kim JW, Koh JM, Park JH, Chang JS. Validation of FRAX without BMD: an age-related analysis of the fifth Korean National Health and Nutrition Examination Survey (KNHANES v-1, 2010). Bone. 2015;75:27-31.

Kranjc T, Trajanoska K, Medina-Gomez C, Rivadeneira F. A machine learning approach to predict non-vertebral fracture risk from dual X-ray absorptiometry images. Calcif Tissue Int. 2019;104 (Suppl 1):S18-S9.

Kyriakos G, Vidal-Casariego A, Quiles-Sanchez LV, Calleja-Fernandez A, Avila-Turcios D, Urosa-Maggi JA, et al. A comparative study between the implementation of the FRIDEX calibration and the NOGG guideline in the management of osteoporosis in routine clinical practice. Reumatol Clin. 2017;13(5):258-63.

Leslie WD, Brennan-Olsen SL, Morin SN, Lix LM. Fracture prediction from repeat bmd measurements in clinical practice. Osteoporos Int. 2016;27(1):203-10.

Leslie WD, Lix LM, Langsetmo L, Berger C, Goltzman D, Hanley DA, et al. Construction of a FRAX® model for the assessment of fracture probability in Canada and implications for treatment. Osteoporos Int. 2011;22(3):817-27.

Leslie WD, Lix LM. Absolute fracture risk assessment using lumbar spine and femoral neck bone density measurements: derivation and validation of a hybrid system. J Bone Miner Res. 2011;26(3):460-7.

Leslie W, Luo Y, Yang S, Goertzen AL, Ahmed S, Delubac I, et al. Fracture risk indices from DXA-based finite element analysis predict incident fractures independently from FRAX: the Manitoba BMD registry. J Clin Densitom. 2019;22(3):338-345.

Leslie WD, Luo Y, Yang S, Goertzen AL, Ahmed S, Delubac I, et al. Fracture Risk Indices From DXA-Based Finite Element Analysis Predict Incident Fractures Independently From FRAX: The Manitoba BMD Registry. J Clin Densitom. 2019;22(3):338-45.

Leslie WD, Metge C, Ward L. Contribution of clinical risk factors to bone density-based absolute fracture risk assessment in postmenopausal women. Osteoporos Int. 2003;14(4):334-8.

Leslie WD, Morin SN, Lix LM, Majumdar SR. Does diabetes modify the effect of FRAX risk factors for predicting major osteoporotic and hip fracture? Osteoporos Int. 2014;25(12):2817-24.

Leslie WD, Morin SN, Lix LM, Binkley N. Targeted bone density testing for optimizing fracture prevention in Canada. Osteoporos Int. 2020;31(7):1291-7.

Leslie WD, Morin SN, Lix LM, Martineau P, Bryanton M, McCloskey EV, et al. Reassessment Intervals for Transition From Low to High Fracture Risk Among Adults Older Than 50 Years. JAMA. 2020;3(1):e1918954.

Martel DR, Lysy M, Laing AC. Predicting population level hip fracture risk: a novel hierarchical model incorporating probabilistic approaches and factor of risk principles. Comput Methods Biomech Biomed Engin. 2020;23(15):1201-14.

Myasoutova L, Vasilyev A, Abdulganieva D, Lapshina S. Influence of bone mineral density at the femoral neck on the probability of osteoporotic fractures. Ann Rheum Dis. 2015;74(Suppl 2):1197.

Nakatoh S, Takemaru Y. Application of the fracture risk assessment tool (frax®) and determination of suitable cut-off values during primary screening in specific health check-ups in Japan. J Bone Miner Metab. 2013;31(6):674-80.

Nguyen T, Sambrook P, Kelly P, Jones G, Lord S, Freund J, et al. Prediction of osteoporotic fractures by postural instability and bone density. BMJ. 1993;307(6912):1111-5.

Pasco JA, Mohebbi M, Tembo MC, Holloway-Kew KL, Hyde NK, Williams LJ, et al. Repurposing a fracture risk calculator (FRAX) as a screening tool for women at risk for sarcopenia. Osteoporos Int. 2020;31(7):1389-94.

Pfister AK, Welch CA, Emmett MK, Gessford AK. An approach to identify rural women aged 60 to 64 for osteoporosis treatment. South Med J. 2012;105(1):11-7.
Pluskiewicz W, Adamczyk P, Franek E, Leszczynski P, Sewerynek E, Wichrowska H, et al. Ten-year probability of osteoporotic fracture in 2012 Polish women assessed by FRAX and nomogram by Nguyen et al.-Conformity between methods and their clinical utility. Bone. 2010;46(6):1661-7.
Sanfelix-Genoves J, Peiro S, Sanfelix-Gimeno G, Giner V, Gil V, Pascual M, et al. Development and validation of a population-based prediction scale for osteoporotic fracture in the region of valencia, spain: the ESOSVAL-R study. BMC Public Health. 2010;10:153.
Schousboe JT, Lix LM, Morin SN, Derkatch S, Bryanton M, Alhrbi M, et al. Prevalent vertebral fracture on bone density lateral spine (VFA) images in routine clinical practice predict incident fractures. Bone. 2019;121:72-9.
Schousboe JT, Vo T, Taylor BC, Cawthon PM, Schwartz AV, Bauer DC, et al. Prediction of incident major osteoporotic and hip fractures by trabecular bone score (TBS) and prevalent radiographic vertebral fracture in older men. J Bone Miner Res. 2016;31(3):690-7.
Sengul I, Askin A, Tosun A, Aydin E, Nas-Kirdar N. Comparison of screening strategies for bone mineral density measurement. Turk J Endocrinol Metab.2019;23(4):206-12.
Shevroja E, Koromani F, Leslie WD, Lamy O, Rivadeneira F, Hans D. The utility of TBS-adjusted BMD T-score in the discrimination of the major osteoporotic fractures in the postmenopausal women of the Rotterdam and the Osteolaus studies. J Clin Densitom. 2018;21(4):610.
Si L, Eisman JA, Winzenberg T, Sanders KM, Center JR, Nguyen TV, et al. Development and validation of the risk engine for an Australian Health Economics Model of Osteoporosis. Osteoporos Int. 2021.
Skowronska-Jozwiak E, Wojcicka A, Lorenc RS, Lewinski A. Comparison of selected methods for fracture risk assessment in postmenopausal women: analysis of the Lodz population in the EPOLOS study. Pol Arch Med Wewn. 2010;120(5):197-202.
Sosa M, Sosa-Cabrera N, Sablon N, Rivero L, Gomez De Tejada MJ, Reyes-Dominguez AI, et al. Validation of the Qfracture scale in Spain. Osteoporos Int. 2016;27(Suppl 1):S420-S1.
Sosa-Cabrera N, Reyes-Dominguez AI, Rivero L, Gomez De Tejada MJ, Saavedra P, Sosa M. Comparison of the scales FRAX and Qfracture in the Spanish population. Osteoporos Int. 2016;27(Suppl 1):S427.
Szulc P, Boutroy S, Chapurlat R. Prediction of fractures in men using bone microarchitectural parameters assessed by high-resolution peripheral quantitative computed tomography-the prospective strambo study. J Bone Miner Res. 2018;33(8):1470-9.
Tanaka S, Kuroda T, Saito M, Shiraki M. Urinary pentosidine improves risk classification using fracture risk assessment tools for postmenopausal women. J Bone Miner Res. 2011;26(11):2778-84.
Tembo MC, Holloway-Kew KL, Mohebbi M, Sui SX, Hosking SM, Brennan-Olsen SL, et al. The association between a fracture risk tool and frailty: Geelong Osteoporosis Study. BMC Geriatrics. 2020;20(1):196.
Tripto-Shkolnik L, Vered I, Peltz-Sinvani N, Kowal D, Goldshtein I. Bone Mineral Density of the 1/3 Radius Refines Osteoporosis Diagnosis, Correlates With Prevalent Fractures, and Enhances Fracture Risk Estimates. Endocr Pract. 2021;27(5):408-12.
Tripto-Shkolnik L, Vered I, Tirosh A, Zacay G, Cohen S. What is your risk? Patient self-perceived fracture risk and its comparison to FRAX assessment. Osteoporos Int. 2019;30(Suppl 2):S557.
Unni S, Yao Y, Milne N, Gunning K, Curtis JR, LaFleur J. An evaluation of clinical risk factors for estimating fracture risk in postmenopausal osteoporosis using an electronic medical record database. Osteoporos Int. 2015;26(2):581-7.
van Geel TA, Eisman JA, Geusens PP, van den Bergh JP, Center JR, Dinant GJ. The utility of absolute risk prediction using FRAX® and Garvan fracture risk calculator in daily practice. Maturitas. 2014;77(2):174-9.
Xu G, Yamamoto N, Hayashi K, Takeuchi A, Miwa S, Igarashi K, et al. The accuracy of different FRAX tools in predicting fracture risk in Japan: A comparison study. J Orthop Surg (Hong Kong). 2020;28(2):2309499020917276.
Yalochkina T, Tarbaeva N, Rozhinskaya L, Melnichenko G, Dzerabova K, Belaya Z. Diagnostic accuracy of FRAX and diagnostic performance of FRAX thresholds to identify patients with fractures as eligible for antiosteoporotic treatment among patients with and without type 2 diabetes mellitus. Osteoporos Int. 2018;29(Suppl 1):S467-S8.
Yun H, Delzell E, Ensrud KE, Kilgore ML, Becker D, Morrisey MA, et al. Predicting hip and major osteoporotic fractures using administrative data. Arch Intern Med. 2010;170(21):1940-2.

Zagorski P, Tabor E, Martela-Tomaszek K, Adamczyk P, Pluskiewicz W. Five-year fracture risk assessment in postmenopausal women, using both the POL-RISK calculator and the Garvan nomogram: the Silesia Osteo Active Study. Arch Osteoporos. 2021;16(1):32.

KQ3a (n=62)

Adachi JD, Bone HG, Daizadeh NS, Dakin P, Papapoulos S, Hadji P, et al. Influence of subject discontinuation on long-term nonvertebral fracture rate in the denosumab FREEDOM Extension study. BMC Musculoskelet Disord. 2017;18(1):174.

Adami S, Baroni MC, Broggini M, Carratelli L, Caruso I, Gnessi L, et al. Treatment of postmenopausal osteoporosis with continuous daily oral alendronate in comparison with either placebo or intranasal salmon calcitonin. Osteoporos Int. 1993;3(Suppl 3):S21-7.

Adami S, Libanati C, Boonen S, Cummings SR, Ho PR, Wang A, et al. Denosumab treatment in postmenopausal women with osteoporosis does not interfere with fracture-healing: results from the FREEDOM trial. J Bone Joint Surg Am. 2012;94(23):2113-9.

Austin M, Yang YC, Vittinghoff E, Adami S, Boonen S, Bauer DC, et al. Relationship between bone mineral density changes with denosumab treatment and risk reduction for vertebral and nonvertebral fractures. J Bone Miner Res. 2012;27(3):687-93.

Bekker PJ, Holloway DL, Rasmussen AS, Murphy R, Martin SW, Leese PT, et al. A single-dose placebo-controlled study of AMG 162, a fully human monoclonal antibody to RANKL, in postmenopausal women. J Bone Miner Res. 2004;19(7):1059-66.

Black DM, Thompson DE, Bauer DC, Ensrud K, Musliner T, Hochberg MC, et al. Fracture risk reduction with alendronate in women with osteoporosis: the Fracture Intervention Trial. FIT Research Group. J Clin Endocrinol Metab. 2000;85(11):4118-24.

Boivin GY, Chavassieux PM, Santora AC, Yates J, Meunier PJ. Alendronate increases bone strength by increasing the mean degree of mineralization of bone tissue in osteoporotic women. Bone. 2000;27(5):687-94.

Bone HG, Bolognese MA, Yuen CK, Kendler DL, Miller PD, Yang YC, et al. Effects of denosumab treatment and discontinuation on bone mineral density and bone turnover markers in postmenopausal women with low bone mass. J Clin Endocrinol Metab. 2011;96(4):972-80.

Bone HG, Greenspan SL, McKeever C, Bell N, Davidson M, Downs RW, et al. Alendronate and estrogen effects in postmenopausal women with low bone mineral density. J Clin Endocrinol Metab. 2000;85(2):720-6.

Bone HG, Hosking D, Devogelaer JP, Tucci JR, Emkey RD, Tonino RP, et al. Ten years' experience with alendronate for osteoporosis in postmenopausal women. N Engl J Med. 2004;350(12):1189-99.

Bone HG, Wagman RB, Brandi ML, Brown JP, Chapurlat R, Cummings SR, et al. 10 years of denosumab treatment in postmenopausal women with osteoporosis: results from the phase 3 randomised FREEDOM trial and open-label extension. Lancet Diabetes Endocrinol. 2017;5(7):513-23.

Brown JP, Roux C, Torring O, Ho PR, Beck Jensen JE, Gilchrist N, et al. Discontinuation of denosumab and associated fracture incidence: analysis from the Fracture Reduction Evaluation of Denosumab in Osteoporosis Every 6 Months (FREEDOM) trial. J Bone Miner Res. 2013;28(4):746-52.

Cai G, Laslett L, Aitken D, Halliday A, Pan F, Otahal P, et al. Effect of zoledronic acid and denosumab in patients with lowback pain and modic change: a proof of principle trial. Osteoarthritis Cartilage. 2018;26 (Suppl 1):S416-S7.

Carfora E, Sergio F, Bellini P, Sergio C, Falco D, Zarcone R. Effect of treatment of postmenopausal osteoporosis with continuous daily oral alendronate and the incidence of fractures. Gazz Med Ital. 1998;157(4):105-9.

Chavassieux P, Portero-Muzy N, Roux JP, Horlait S, Dempster DW, Wang A, et al. Reduction of cortical bone turnover and erosion depth after 2 and 3 years of denosumab: Iliac bone histomorphometry in the FREEDOM trial. J Bone Miner Res. 2019;34(4):626-31.

Cryer B, Binkley N, Simonelli C, Lewiecki EM, Lanza F, Chen E, et al. A randomized, placebo-controlled, 6-month study of once-weekly alendronate oral solution for postmenopausal osteoporosis. Am J Geriatr Pharmacother. 2005;3(3):127-36.

Cummings SR, Ferrari S, Eastell R, Gilchrist N, Jensen JB, McClung M, et al. Vertebral fractures after discontinuation of denosumab: a post hoc analysis of the randomized placebo-controlled FREEDOM trial and its extension. J Bone Miner Res. 2018;33(2):190-8.

Dempster DW, Brown JP, Fahrleitner-Pammer A, Kendler D, Rizzo S, Valter I, et al. Effects of long-term denosumab on bone histomorphometry and mineralization in women with postmenopausal osteoporosis. J Clin Endocrinol Metab. 2018;103(7):2498-509.

Dempster DW, Brown J, Yue S, Rizzo S, Farlay D, Wagman RB, et al. Effects of up to 10 years of denosumab treatment on bone matrix mineralization: results from the freedom extension. Calcif Tissue Int. 2017;100 (Suppl 1):S47-S8.

Dempster DW, Brown JP, Yue S, Rizzo S, Farlay D, Wagman RB, et al. Effects of up to 10 years of denosumab treatment on bone matrix mineralization: results from the freedom extension (ext). Osteoporos Int. 2017;28(Suppl 1):S177.

Devogelaer JP, Broll H, Correa-Rotter R, Cumming DC, De Deuxchaisnes CN, Geusens P, et al. Oral alendronate induces progressive increases in bone mass of the spine, hip, and total body over 3 years in postmenopausal women with osteoporosis. Bone. 1996;18(2):141-50.

Eastell R, Black DM, Boonen S, Adami S, Felsenberg D, Lippuner K, et al. Effect of once-yearly zoledronic acid five milligrams on fracture risk and change in femoral neck bone mineral density. J Clin Endocrinol Metab. 2009;94(9):3215-25.

Eastell R, Christiansen C, Grauer A, Kutilek S, Libanati C, McClung MR, et al. Effects of denosumab on bone turnover markers in postmenopausal osteoporosis. J Bone Miner Res. 2011;26(3):530-7.

Felsenberg D, Alenfeld F, Bock O, Hammermeister C, W G. Placebo-controlled multicenter study of oral alendronate in postmenopausal osteoporotic women. FOSIT-Study-Group. Fosamax International Trial. Maturitas. 1998;31(1):35-44.

Ferrari S, Eastell R, Napoli N, Schwartz A, Hofbauer LC, Chines A, et al. Denosumab in postmenopausal women with osteoporosis and diabetes: subgroup analysis of FREEDOM and FREEDOM extension. Bone. 2020;134:115268.

Grey A, Bolland MJ, Horne A, Mihov B, Gamble G, Reid IR. Duration of antiresorptive activity of zoledronate in postmenopausal women with osteopenia: a randomized, controlled multidose trial. CMAJ. 2017;189(36):E1130-E6.

Ho YV, Frauman AG, Thomson W, E S. Effects of alendronate on bone density in men with primary and secondary osteoporosis. Osteoporos Int. 2000;11(2):98-101.

Hochberg MC, Ross PD, Black D, Cummings SR, Genant HK, Nevitt MC, et al. Larger increases in bone mineral density during alendronate therapy are associated with a lower risk of new vertebral fractures in women with postmenopausal osteoporosis. Arthritis Rheum. 1999;42(6):1246-54.

Johansson H, McCloskey E, Oden A, Harvey NC, Black DM, Cauley J, et al. The effect of alendronate on vertebral fracture risk is independent of baseline frax fracture probability: a post hoc analysis of the FIT study. Osteoporos Int. 2017;28(Suppl 1):S619.

Kendler DL, Chines A, Brandi ML, Papapoulos S, Lewiecki EM, Reginster JY, et al. The risk of subsequent osteoporotic fractures is decreased in subjects experiencing fracture while on denosumab: results from the FREEDOM and FREEDOM Extension studies. Osteoporos Int. 2019;30(1):71-8.

Khan A, Dubois S, Khan AA, Zohair Rahman M, Ahmed Khan O, Syed HT, et al. A randomized, double-blind, placebo-controlled study to evaluate the effects of alendronate on bone mineral density and bone remodelling in perimenopausal women with low bone mineral density. J Obstet Gynaecol Can. 2014;36(11):976-82.

Koh JM, Chung DJ, Chung YS, Kang MI, Kim IJ, Min YK, et al. Assessment of denosumab in Korean postmenopausal women with osteoporosis: randomized, double-blind, placebo-controlled trial with open-label extension. Yonsei Med J. 2016;57(4):905-14.

Koivisto K, Karppinen J, Haapea M, Jarvinen J, Kyllonen E, Tervonen O, et al. The effect of zoledronic acid on serum biomarkers among patients with chronic low back pain and modic changes in lumbar magnetic resonance imaging. Diagnostics. 2019;9(4):212.

Kumagai Y, Hasunuma T, Padhi D. A randomized, double-blind, placebo-controlled, single-dose study to evaluate the safety, tolerability, pharmacokinetics and pharmacodynamics of denosumab administered subcutaneously to postmenopausal Japanese women. Bone. 2011;49(5):1101-7.

Kung AW, Yeung SS, Chu LW. The efficacy and tolerability of alendronate in postmenopausal osteoporotic Chinese women: a randomized placebo-controlled study. Calcif Tissue Int. 2000;67(4):286-90.

Kuroda T, Shiraki M, Ueda S, Takeuchi Y, Sugimoto T, Nakamura T. Acute phase reactions after intravenous infusion of zoledronic acid in Japanese patients with osteoporosis: sub-analysis of the phase III (ZONE) study. J Bone Miner Res. 2017;32(Suppl 1):S163.

Levis S, Quandt SA, Thompson D, Scott J, Schneider DL, Ross PD, et al. Alendronate reduces the risk of multiple symptomatic fractures: results from the Fracture Intervention Trial. J Am Geriatr Soc. 2002;50(3):409-15.

Liang BC, Shi ZY, Wang B, Wu P, Kong LC, Yao JL, et al. Intravenous zoledronic acid 5 mg on bone turnover markers and bone mineral density in East China subjects with newly diagnosed osteoporosis: a 24-month clinical study. Orthop Surg. 2017;9(1):103-9.

Majima T, Shimatsu A, Komatsu Y, Satoh N, Fukao A, Ninomiya K, et al. Efficacy of risedronate in Japanese male patients with primary osteoporosis. Intern Med. 2008;47(8):717-23.

McClung MR, Lippuner K, Brandi ML, Zanchetta JR, Bone HG, Chapurlat R, et al. Effect of denosumab on trabecular bone score in postmenopausal women with osteoporosis. Osteoporos Int. 2017;28(10):2967-73.

Nakamura T, Matsumoto T, Sugimoto T, Shiraki M. Dose-response study of denosumab on bone mineral density and bone turnover markers in Japanese postmenopausal women with osteoporosis. Osteoporos Int. 2012;23(3):1131-40.

Napoli N, Pannacciulli N, Vittinghoff E, Crittenden D, Yun J, Wang A, et al. Effect of denosumab on fasting glucose in women with diabetes or prediabetes from the FREEDOM trial. Diabetes Metab Res Rev. 2018;34(4):e2991.

Palacios S, Kalouche-Khalil L, Rizzoli R, Zapalowski C, Resch H, Adachi JD, et al. Treatment with denosumab reduces secondary fracture risk in women with postmenopausal osteoporosis. Climacteric. 2015;18(6):805-12.

Patel DV, Bolland M, Nisa Z, Al-Abuwsi F, Singh M, Horne A, et al. Incidence of ocular side effects with intravenous zoledronate: secondary analysis of a randomized controlled trial. Osteoporos Int. 2015;26(2):499-503.

Patel DV, Horne A, House M, Reid IR, McGhee CN. The incidence of acute anterior uveitis after intravenous zoledronate. Ophthalmology. 2013;120(4):773-6.

Quandt SA, Thompson DE, Schneider DL, Nevitt MC, Black DM. Effect of alendronate on vertebral fracture risk in women with bone mineral density T scores of-1.6 to -2.5 at the femoral neck: the Fracture Intervention Trial. Mayo Clin Proc. 2005;80(3):343-9.

Reginster J, Minne HW, Sorensen OH, Hooper M, Roux C, Brandi ML, et al. Randomized trial of the effects of risedronate on vertebral fractures in women with established postmenopausal osteoporosis. Vertebral Efficacy with Risedronate Therapy (VERT) Study Group. Osteoporos Int. 2000;11(1):83-91.

Reid IR, Horne AM, Mihov B, Stewart A, Garratt E, Bastin S, et al. Effects of zoledronate on cancer, cardiac events, and mortality in osteopenic older women. J Bone Miner Res. 2020;35(1):20-7.

Reid IR, Horne AM, Mihov B, Stewart A, Garratt E, Wiessing KR, et al. Anti-fracture efficacy of zoledronate in subgroups of osteopenic postmenopausal women: secondary analysis of a randomized controlled trial. J Intern Med. 2019;286(2):221-9.

Reid IR, Miller PD, Brown JP, Kendler DL, Fahrleitner-Pammer A, Valter I, et al. Effects of denosumab on bone histomorphometry: the FREEDOM and STAND studies. J Bone Miner Res. 2010;25(10):2256-65.

Rossini M, Gatti D, Zamberlan N, Braga V, Dorizzi R, Adami S. Long-term effects of a treatment course with oral alendronate of postmenopausal osteoporosis. J Bone Miner Res. 1994;9(11):1833-7.

Shapses SA, Kendler DL, Robson R, Hansen KE, Sherrell RM, Field MP, et al. Effect of alendronate and vitamin D3 on fractional calcium absorption in a double-blind, randomized, placebo-controlled trial in postmenopausal osteoporotic women. J Bone Miner Res. 2011;26(8):1836-44.

Simon JA, Recknor C, Moffett Jr AH, Adachi JD, Franek E, Lewiecki EM, et al. Impact of denosumab on the peripheral skeleton of postmenopausal women with osteoporosis: bone density, mass, and strength of the radius, and wrist fracture. Menopause. 2013;20(2):130-7.

Sone T, Kon N, Gaither KW, Okubo N, Osakabe T, Nakayama Y. Effects of 3-year denosumab treatment on hip structure in Japanese postmenopausal women and men with osteoporosis. Bone Rep. 2017;7:164-171.

Sone T, Kon N, Okubo N, Fukunaga M, Ito M, Nakamura T. Effect of 3 year denosumab treatment on hip structure in Japanese postmenopausal women and men with osteoporosis. J Clin Densitom. 2018;21(4):602-603.

Sri Lanka Clinical Trials Registry [Internet]. Colombo: Sri Lanka Medical Association (Sri Lanka). 2007 Feb 1. Identifier SLCTR/2018/038, Effect of alendronate and vitamin D on selected bone turnover markers in women with postmenopausal osteoporosis and a high fracture risk; a randomized controlled trial; 2018 Nov 14 [cited 2020 Jul 27]. Available from: https://apps.who.int/trialsearch/Trial2.aspx?TrialID=SLCTR/2018/038.

Tanaka S, Shiraki M, Ohashi M, Ueda S, Nakamura T. Acute phase reactions after intravenous infusion of zoledronic acid in Japanese patients with osteoporosis: subanalyses of the phase III (ZONE) study. J Bone Miner Res. 2016;31(Suppl 1):S382.

Watts N, Roux C, Modlin J, Brown J, Daniels A, Jackson S, et al. Infections in postmenopausal women with osteoporosis treated with denosumab or placebo: coincidence or causal association? Osteoporos Int. 2012;23(1):327-37.

Yen ML, Yen BL, Jang MH, Hsu SH, Cheng WC, Tsai KS. Effects of alendronate on osteopenic postmenopausal Chinese women. Bone. 2000;27(5):681-5.

Zysset P, Pahr D, Engelke K, Genant HK, McClung MR, Kendler DL, et al. Comparison of proximal femur and vertebral body strength improvements in the FREEDOM trial using an alternative finite element methodology. Bone. 2015;81:122-30.

KQ3b (n=11)

Bastounis A, Leonardi-Bee J, Langley T, Paskins Z, Davis S, Sahota O. Comparing treatment adherence in patients receiving alendronate and alternative bisphosphonates for preventing fragility fractures: a comprehensive systematic review and meta-analysis. PROSPERO [Internet]. 2020 Mar 30 [cited 2020 Nov 4]. Available from: https://www.crd.york.ac.uk/prospero/display_record.php?ID=CRD42020177166.

Billington E, Aghajafari F, Dunnewold N. Effects of bisphosphonates on fracture incidence, bone mineral density, and bone turnover in perimenopausal and early postmenopausal women: A systematic review and meta-analysis. PROSPERO [Internet]. 2020 Apr 28 [cited 2020 Nov 4]. Available from: https://www.crd.york.ac.uk/prospero/display_record.php?ID=CRD42020169109.

Hiligsmann M, Evers SM, Ben Sedrine W, Kanis JA, Ramaekers B, Reginster JY, et al. A systematic review of cost-effectiveness analyses of drugs for postmenopausal osteoporosis. Pharmacoeconomics. 2015;33:205-24.

Hu Y, Ke Z, Han J. Effectiveness of pharmacological therapies on bone mineral density and fracture at various sites: a network meta-analysis of randomized controlled trials. PROSPERO [Internet]. 2020 Apr 28 [cited 2020 Nov 4]. Available from: https://www.crd.york.ac.uk/prospero/display_record.php?ID=CRD42020158203.

Nayak S, Greenspan SL. A systematic review and meta-analysis of the effect of bisphosphonate drug holidays on bone mineral density and osteoporotic fracture risk. Osteoporos Int. 2019;30:705-20.

Saito T, Sterbenz JM, Malay S, Zhong L, MacEachern MP, Chung KC. Effectiveness of anti-osteoporotic drugs to prevent secondary fragility fractures: systematic review and meta-analysis. Osteoporos Int. 2017;28:3289-300.

Salmerón MZ, Prieto-Alhambra D. The anti fracture efficacy of different anti osteoporosis medications in the male population: a systematic review and meta-analysis. PROSPERO; 2017. https://www.crd.york.ac.uk/prospero/display_record.php?ID=CRD42017057309.

Simoes R, Soares-Jr JM, Baracat E, Filho EF, Hayashida S. Denosumab for the treatment of osteoporosis and osteopenia in postmenopausal women: a systematic review. PROSPERO [Internet]. 2018 Mar 7 [cited 2020 Nov 4]. Available from: https://www.crd.york.ac.uk/prospero/display_record.php?ID=CRD42018090230.

Sanderson J, Martyn-St James M, Stevens J, Goka E, Wong R, Campbell F, et al. Clinical effectiveness of bisphosphonates for the prevention of fragility fractures: a systematic review and network meta-analysis. Bone. 2016;89:52-8.

Xu Z. Alendronate for the treatment of osteoporosis in men: a meta-analysis of randomized controlled trials. Am J Ther. 2017;24:e130-e8.

Zeng LF, Pan BQ, Liang GH, Luo MH, Cao Y, Guo D, et al. Does routine anti-osteoporosis medication lower the risk of fractures in male subjects? An updated systematic review with meta-analysis of clinical trials. Front Pharmacol. 2019;10:882.

KQ4 (n=15)

Amer M, Noor S, Kashif SM, Nazir SUR, Ghazanfar T, Yousaf S. Evaluation of Disease Related Knowledge in Patients of Osteoporosis: An Observational Study. Altern Ther Health Med. 2021;27(S1):97-103.

El Hage C, Hallit S, Akel M, Dagher E. Osteoporosis awareness and health beliefs among Lebanese women aged 40 years and above. Osteoporosis Int. 2019;30(4):771-86.

Fourmy J, Begum J, Nisar MK. Can we manage osteoporosis without patients ever seeing a consultant? Rheumatology (United Kingdom). 2020;59(Suppl 2).

Funnell L, Chepesiuk R, Djekic-Ivankovic M, Giangregorio L, Braganca Rodrigues I, Ridout R, et al. Patient engagement in clinical guidelines development: Input from >1000 members of the canadian osteoporosis patient network. Osteoporos Int. 2019;30(Suppl 2):S171-S2.

Gasperini B, Montanari R, Lamanna P, Espinosa E, Cherubini A. How much do you know about osteoporosis? a survey among patients referred to the DXA exam by general practitioners in Italy. Osteoporos Int. 2020;31(Suppl 1):S531.

Gasperini B, Montanari R, Lamanna P, Martinez V, Espinosa E, Cherubini A, et al. How much do patients know about osteoporosis? A survey among patients referred to the dual-energy X-ray absorptiometry exam. Aging Clin Exp Res. 2021.

Hawarden A, Jinks C, Mahmood W, Gwilym S, Paskins Z. Patient priorities in osteoporosis research: generating meaningful research questions. Rheumatology (United Kingdom). 2019;58(Suppl 3).

Iranian Registry of Clinical Trials [Internet]. Tehran: IRCT. [2013]. IRCT2013060310661N3, Education on the prevention of osteoporosis in postmenopausal women; 2013 Dec 18 [cited 2020 Nov 27]. Available from: https://apps.who.int/trialsearch/Trial2.aspx?TrialID=IRCT2013060310661N3.

Karimiankakolaki Z, Gerayllo S, Khadibi M, Motaghi B, Heidari F. The impact of training through the telegram's virtual network on promoting women's knowledge and perceptions in preventing osteoporosis. Indian J Rheumatol. 2019;14(3):194-9.

McHorney CA, Schousboe JT, Cline RR, Weiss TW. The impact of osteoporosis medication beliefs and side-effect experiences on non-adherence to oral bisphosphonates. Curr Med Res Opin. 2007;23(12):3137-52.

Pencille LJ, Campbell ME, Van Houten HK, Shah ND, Mullan RJ, Swiglo BA, et al. Protocol for the Osteoporosis Choice trial. A pilot randomized trial of a decision aid in primary care practice. Trials. 2009;10:113.

Ranbhise N, Talsandekar A, Rathod SA. Evaluating the effectiveness of informational booklet on knowledge of osteoporosis among working women of, kolhapur information booklet and osteoporosis. Indian J Public Health Res Dev. 2020;11(8):78-83.

Ryan P, Brown RL, Csuka ME, Papanek P. Efficacy of Osteoporosis Prevention Smartphone App. Nurs Res. 2020;69(1):31-41.

Sitati FC, Obimbo MM, Gichangi P. Knowledge and Beliefs on Osteoporosis among African Postmenopausal Women in a Kenyan Semi-Rural County of Kiambu. J Bone Metab. 2021;28(1):91-8.

Winzenberg T, Oldenburg B, Frendin S, De Wit L, Riley M, Jones G. The effect on behavior and bone mineral density of individualized bone mineral density feedback and educational interventions in premenopausal women: a randomized controlled trial [NCT00273260]. BMC Public Health. 2006;6:12.

<u>Duplicate or abstract of another study</u>

KQ1 (n=37)

Cooper C, Parsons C, Harvey NC, Shepstone L, Kanis JA, Lenaghan E, et al. Effect of fracture risk assessment on anti-osteoporosis medication use and adherence: findings from the SCOOP trial. J Bone Miner Res. 2017;32:S108.

Hoiberg MP, Rubin KH, Holmberg T, Rothmann MJ, Moller S, Gram J, et al. Use of antiosteoporotic medication in the Danish ROSE population-based screening study. Osteoporos Int. 2019;30(6):1223-33.

ISRCTN registry [Internet]. Preventive health survey in elderly people. [date unknown]. Available from: https://trialsearchwhoint/Trial2aspx?TrialID=ISRCTN21267689. 2006.

ISRCTN registry [Internet]. London: BMC. [date unknown]. Identifier ISRCTN11021925, A pragmatic randomised clinical trial of the effectiveness and cost effectiveness of targeted population screening for low bone mineral density (BMD) in the prevention of neck of femur fractures; 2005 Jun 17 [cited 2020 Nov 12]. Available from: http://www.isrctn.com/ISRCTN11021925.

Kern L, Powe N, Levine M, Harris T, Robbins J, Fitzpatrick A, et al. Is screening for osteoporosis associated with fewer hip fractures? J Gen Intern Med. 2002;17(Suppl 1):144.

LaCroix AZ, Buist DS, Brenneman SK, Abbott III TA. Evaluation of three population-based strategies for fracture prevention: results of the osteoporosis population-based risk assessment (OPRA) trial. Med Care. 2005;43(3):293-302.

McCloskey E, Harvey N, Johansson H, Shepstone L, Lenaghan E, Cooper C, et al. Women identified at high risk of hip fracture based on FRAX are responsive to appropriate osteoporosis management: results from the SCOOP study of population screening. J Bone Miner Res. 2017;32(Suppl 1):S30.

McCloskey E, Harvey N, Johansson H, Shepstone L, Lenaghan E, Cooper C, et al. Women identified at high risk of hip fracture based on FRAX are responsive to appropriate osteoporosis management: analysis from the SCOOP study of population screening. Osteoporos Int. 2018;29(Suppl 2):S613-S4.
McCloskey E, Johansson H, Harvey NC, Shepstone L, Lenaghan E, Fordham R, et al. Management of patients with high baseline hip fracture risk by FRAX reduces hip fractures—a post hoc analysis of the SCOOP Study. J Bone Miner Res. 2018;33(6):1020-6.
McCloskey E, Harvey N, Johansson H, Shepstone L, Lenaghan E, Cooper C, et al. Women identified at high risk of hip fracture based on FRAX are responsive to appropriate osteoporosis management: results from the SCOOP study of population screening. J Bone Miner Res. 2017;32:S30.
McCloskey E, Harvey N, Johansson H, Shepstone L, Lenaghan E, Cooper C, et al. Women identified at high risk of hip fracture based on FRAX are responsive to appropriate osteoporosis management: analysis from the SCOOP study of population screening. Osteoporos Int. 2018;29:S613-S4.
McCloskey EV, Harvey NC, Johansson H, Shepstone L, Lenaghan E, Cooper C, et al. Women identified at high risk based on FRAX hip fracture probability are responsive to appropriate osteoporosis management: analysis from the scoop study of population screening. Osteoporos Int. 2017;28(Suppl 1):S60.
McCloskey E, Harvey N, Johansson H, Shepstone L, Lenaghan E, Cooper C, et al. Women at high risk of hip fracture based on FRAX respond to appropriate osteoporosis management: analysis from the SCOOP study of population screening. JBMR Plus. 2018;2(Suppl 1):S12.
McCloskey EV, Lenaghan E, Clarke S, Cooper C, Fordham R, Gittoes N, et al. Screening based on FRAX fracture risk assessment reduces the incidence of hip fractures in older community dwelling women-results from the scoop study in the UK. J Bone Miner Res. 2016;31(Suppl 1).
McCloskey E, Lenaghan E, Clarke S, Fordham R, Gittoes N, Harvey I, et al. Screening based on FRAX fracture risk assessment reduces the incidence of hip fractures in older community-dwelling women-results from the SCOOP study. Osteoporos Int. 2016;27(2).
McCloskey E, Lenaghan E, Clarke S, Fordham R, Gittoes N, Harvey I, et al. Screening based on FRAX fracture risk assessment reduces the incidence of hip fractures in older community-dwelling women-results from the SCOOP study. Osteoporos Int. 2016;27(2):S624.
Merlijn T, Swart K, Netelenbos C, Elders P. Screening of high fracture risk in primary care to reduce fractures: the SALT Osteoporosis Study a randomized trial. Calcif Tissue Int. 2018;102(1 Suppl 1):S17.
Merlijn T, Swart KM, van Schoor NM, Heymans MW, van der Zwaard BC, van der Heijden AA, et al. The Effect of a Screening and Treatment Program for the Prevention of Fractures in Older Women: A Randomized Pragmatic Trial. J Bone Miner Res. 2019;34(11):1993-2000.
Parsons C, Curtis EM, Shepstone L, Kanis JA, Lenaghan E, Clarke S, et al. Fracture risk screening using FRAX is effective for secondary fracture prevention: A post hoc analysis of the scoop trial. Osteoporos Int. 2020;31(Suppl 1):S480-S1.
Parsons C, Harvey N, Shepstone L, Kanis JA, Lenaghan E, Clarke S, et al. Systematic fracture risk screening using FRAX results in greater use of, and adherence to, anti-osteoporosis medications: the UK SCOOP Trial. JBMR plus. 2018;2(Suppl 1):S6-S7.
Parsons C, Harvey N, Shepstone L, Kanis J, Lenaghan E, Clarke S, et al. Systematic screening using FRAX leads to increased use of, and adherence to, antiosteoporosis medications: the UK SCOOP Trial. Osteoporos Int. 2018;29(Suppl 1):S54-S5.
Parsons C, Harvey N, Shepstone L, Kanis J, Lenaghan E, Clarke S, et al. Systematic screening using FRAX leads to increased use of, and adherence to, antiosteoporosis medications: the UK SCOOP trial. Osteoporos Int. 2018;29(Suppl 2):S613.
Parsons C, Harvey N, Shepstone L, Kanis JA, Lenaghan E, Clarke S, et al. Systematic screening using FRAX leads to increased use of, and adherence to, antiosteoporosis medications: the UK SCOOP trial. Osteoporos Int. 2018;29(Suppl 1):S54-S5.
Shepstone L, Lenaghan E, Clarke S, Fordham R, Gittoes N, Harvey I, et al. A randomized controlled trial of screening in the community to reduce fractures in older women in the UK (the scoop study). Osteoporos Int. 2016;(Suppl 1):S42-S3.

Shepstone L, Lenaghan E, Clarke S, Fordham R, Gittoes N, Harvey I, et al. A randomized controlled trial of screening in the community to reduce fractures in older women in the UK (the SCOOP study). Osteoporos Int. 2016;27(1 Suppl 1):S42-S3.

Shepstone L, Lenaghan E, Cooper C, Clarke S, Fong-Soe-Khioe R, Fordham R, et al. Screening in the community to reduce fractures in older women (SCOOP): a randomised controlled trial. Lancet. 2018;391(10122):741-7.

Rajan R, Paul J, Cherian KE, Asha HS, Kapoor N, Paul TV. FRAX with or without BMD and TBS predicts fragility fractures in community-dwelling rural southern Indian postmenopausal women. Arch Osteoporos. 2020;15(1):82.

Rapp K, Lamb S, Roigk P, Becker C, Schulz C, Konig HH, et al. Effect of an osteoporotic fracture prevention program on fracture incidence in routine care. A cluster-randomized trial. Eur Geriatr Med. 2021;12(Suppl 1):S239.

Rasmussen OWR, Lauszus FFL. Effectiveness of a two-step population-based osteoporosis screening program using FRAX: The randomized risk-stratified osteoporosis strategy evaluation (ROSE) study. Osteoporos Int. 2019;30(Suppl 1):S70-S1.

Rothmann MJ, Möller S, Holmberg T, Højberg M, Gram J, Bech M, et al. Non-participation in systematic screening for osteoporosis-the ROSE trial. Osteoporos Int. 2017;28(12):3389-99.

Rothmann MJ, Möller S, Holmberg T, Højberg M, Gram J, Bech M, et al. Non-participation in systematic screening for osteoporosis—the ROSE trial. Osteoporos Int. 2017;28(12):3389-99.

Rubin KH, Holmberg T, Rothmann MJ, Høiberg M, Möller S, Brakmann R, et al. Effectiveness of a two-step population-based osteoporosis screening program using FRAX: the randomized Risk-stratified Osteoporosis Strategy Evaluation (ROSE) study. Osteoporos Int. 2018:29(3)567-578.

Rubin KH, Holmberg T, Rothmann MJ, Brixen K, Hoejberg M, Gram, J, et al. The ROSE study: Risk-stratified Osteoporosis Strategy Evaluation Effectiveness of a two-step population based osteoporosis screening programme using FRAX: a randomised prospective population-based study. J Bone Miner Res. 2017;32(Suppl 1):S29-S30.

Rubin KH, Holmberg T, Rothmann MJ, Brixen K, Hoejberg M, Gram J, et al. The ROSE study: risk-stratified Osteoporosis Strategy Evaluation Effectiveness of a two-step population based osteoporosis screening programme using FRAX®: a randomised prospective population-based study. J Bone Miner Res. 2017;32:S29-S30.

Rubin KH, Holmberg T, Rothmann MJ, Høiberg M, Barkmann R, Gram J, et al. The risk-stratified osteoporosis strategy evaluation study (ROSE): a randomized prospective population-based study. Design and baseline characteristics. Calcif Tissue Int. 2015;96(2):167-79.

Rubin KH, Rothmann MJ, Holmberg T, Høiberg M, Möller S, Barkmann R, et al. Effectiveness of a two-step population-based osteoporosis screening program using FRAX: the randomized Risk-stratified Osteoporosis Strategy Evaluation (ROSE) study. Osteoporos Int. 2018;29(3):567-78.

Turner DA, Khioe RF, Shepstone L, Lenaghan E, Cooper C, Gittoes N, et al. The cost-effectiveness of screening in the community to reduce osteoporotic fractures in older women in the UK: economic evaluation of the SCOOP Study. J Bone Miner Res. 2018;33(5):845-51.

KQ2 (n=33)

Adams A, Balasubramanian A, Zhou H, Platt R, Wenkert D, Jacobsen S, et al. Prediction model for the two-year risk of fracture among older us women. Arthritis Rheumatol. 2017;69(Suppl 10).

Amin S, Atkinson EJ, Khosla S. Identifying women and men at high fracture risk by leveraging the electronic medical record to estimate FRAX treatment thresholds. Osteoporos Int. 2017;28(Suppl 1):S514-S5.

Azagra R, Gabriel P, Aguye A, Morina D. The application of the FRIDEX calibration of the FRAX tool to determine the absolute risk of osteoporotic fracture among Spanish women. Reumatol Clin. 2017.

Azagra R, Roca G, Encabo G, Prieto D, Aguye A, Zwart M, et al. Prediction of absolute risk of fragility fracture at 10 years in a Spanish population: validation of the WHO FRAX tool in Spain. BMC Musculoskelet Disord. 2011;12:30.

Bolton J, Morin S, Majumdar S, Lix LM, Johansson H, Oden A, et al. Are psychiatric illnesses and the medications used to treat them FRAX-independent risk factors? The Manitoba BMD cohort. J Bone Miner Res. 2015;30(Suppl 1):S22.

Buehring B, Lewis BL, Hansen KE, Cummings SR, Lane NE, Binkley N, et al. Association of dysmobility syndrome with fracture risk in the MROS cohort. Ann Rheum Dis. 2017;76(Suppl 2):690.

Crandall C, Larson J, LaCroix A, Cauley J, LeBoff M, Li W, et al. Screening young postmenopausal women for osteoporosis: comparison of hip fracture risk prediction by the Garvan and FRAX risk calculators in the Women's Health Initiative observational study and clinical trials. J Bone Miner Res. 2017;32(Suppl 1):S249.

Crandall CJ, Larson J, LaCroix A, Cauley JA, LeBoff MS, Li W, et al. Predicting Fracture Risk in Younger Postmenopausal Women: Comparison of the Garvan and FRAX Risk Calculators in the Women's Health Initiative Study. J Gen Intern Med. 2019;34(2):235-42.

Crandall CJ, Schousboe JT, Morin SN, Lix LM, Leslie W. Performance of FRAX and FRAX-Based Treatment Thresholds in Women Aged 40 Years and Older: The Manitoba BMD Registry. J Bone Miner Res. 2019;34(8):1419-27.

Goldshtein I, Gerber Y, Ish-Shalom S, Leshno M. Validation of fracture risk assesment tool using real-world data. Pharmacoepidemiol Drug Saf. 2016;25(Suppl 3):250-1.

Goldshtein I, Leshno M, Gerber Y, Ish-Shalom S. Application of frax in Israel: Validation and impact assessment using real-world medical databases. Osteoporos Int. 2016;27(Suppl 1):S413-S4.

Gonzalez-Macias J, Marin F, Vlia J, Diez-Perez A. Probability of fractures predicted by FRAX® and observed incidence in the Spanish ECOSAP Study cohort. Bone. 2012;50(1):373-7.

Holloway K, Mohebbi M, Hans D, Brennan- Olsen SL, Kotowicz MA, Pasco JA. Prediction of hip fractures in Australian men using FRAX scores adjusted with trabecular bone score. Osteoporos Int. 2016;27(Suppl 1):S351.

Holloway KL, Mohebbi M, Hans D, Brennan-Olsen SL, Kotowicz MA, Pasco JA. Prediction of hip fractures in Australian men using frax scores adjusted with trabecular bone score. Osteoporos Int. 2016;27(Suppl 1):S350-S1.

Jonasson GB, Sundh V, Hakeberg M, Ahlqwist M, Lissner L, Hange D. Evaluation of clinical and radiographic indices as predictors of osteoporotic fractures: a 10-year longitudinal study. Oral Surg Oral Med Oral Pathol Oral Radiol. 2018;125(5):487-94.

Kalvesten J, Lui LY, Cummings SR, Brismar TB. Association of digital X-ray radiogrammetry with frax and dual-energy X-ray absorptiometry: The study of osteoporotic fractures. Osteoporos Int. 2016;27(Suppl 1):S113.

Kuzma M, Koller T, Nemethova E, Killinger Z, Payer J, Hans D, et al. Less strict high- versus low-risk thresholds for the FRAX and TBS-adjusted FRAX predict clinical fractures in osteopenic postmenopausal women with no prior fractures: A 5-year longitudinal cohort study. J fur Mineralstoffwechsel. 2016;23(Suppl 1):5.

Leslie W, Majumdar S, Morin S, Lix L, Johansson H, Oden A, et al. FRAX and the confounding effect of hip area in Canadian white women: the Manitoba BMD cohort. J Bone Miner Res. 2016;31(Suppl 1):236.

Leslie W, Majumdar S, Morin S, Lix L, Schousboe J, Ensrud K, et al. Performance of FRAX in clinical practice according to who and nof definitions of osteoporosis in Canadian women and men: the Manitoba BMD cohort. J Bone Miner Res. 2016;31(Suppl 1):S143-4.

Leslie W, Morin S, Majumdar S, Lix L, Johansson H, Oden A, et al. Longer duration of diabetes strongly impacts fracture risk assessment: the Manitoba BMD cohort. J Bone Miner Res. 2016;31(Suppl 1):S25.

Leslie W, Yang S, Goertzen A, Lix L, Luo Y. Fracture risk indices from DXA-based finite element analysis stratify incident fracture risk independently from FRAX: the Manitoba BMD registry. J Bone Miner Res. 2017;32(Suppl 1):S28.

Leslie WD, Majumdar SR, Morin SN, Lix LM, Johansson H, Oden A, et al. FRAX for fracture prediction shorter and longer than 10 years: the Manitoba BMD registry. Osteoporos Int. 2017;28(9):2557-64.

Leslie WD, Majumdar SR, Morin SN, Lix LM, Schousboe JT, Ensrud KE, et al. Performance of FRAX in clinical practice according to sex and osteoporosis definitions: the Manitoba BMD registry. Osteoporos Int. 2018;29(3):759-67.

Leslie WD, Majumdar SR, Morin SN, Lix LM, Schousboe JT, Ensrud KE, et al. Performance of FRAX in clinical practice according to sex and osteoporosis definitions: the Manitoba BMD registry. Osteoporos Int. 2018;29(3):759-67. (same study as above)

Leslie WD, Tsang JF, Lix LM. Validation of ten-year fracture risk prediction: a clinical cohort study from the Manitoba Bone Density Program. Bone. 2008;43(4):667-71.

Merlijn T, Swart K, Netelenbos C, Elders P. Screening of high fracture risk in primary care to reduce fractures: the SALT Osteoporosis Study a randomized trial. Calcif Tissue Int. 2018;102(Suppl 1):S17.

Morin S, Majumdar S, Lix L, Johansson H, Oden A, McLoskey E, et al. Net reclassification improvement with FRAX versus a simpler risk assessment system: more is more. J Bone Miner Res. 2015;30(Suppl 1):S103.

Sosa M, Sablon N, Reyes-Dominguez AI, Saavedra P, Sosa-Cabrera N, Rivero L, et al. Validation of the qfracture scale in spain. Osteoporos Int. 2016;27(Suppl 1):S420.

Sosa-Cabrera N, Reyes-Dominguez AI, Saavedra P, Sosa M, Rivero L, Gomez De Tejada MJ. Comparison of the scales frax and qfracture in the spanish population. Osteoporos Int. 2016;27(Suppl 1):S426-S7.

Sosa-Henriquez M, Reyes-Dominguez AI, Sosa-Cabrera N, Hernandez-Hernandez D, Diaz-Curiel M, De Tejada MJG. Validation of Garvan scale in a Spanish population. Osteoporos Int. 2017;28(Suppl 1):S255-S6.

Sund R, Honkanen R, Johansson J, Oden A, McCloskey E, Kanis J, et al. Evaluation of the FRAX model for hip fracture predictions in the population-based Kuopio Osteoporosis Risk Factor and Prevention Study (OSTPRE). Calcif Tissue Int. 2014;95(1):39-45.

Tamaki J, Iki M, Kadowaki E, Sato Y, Kajita E, Kagamimori S, et al. Fracture risk prediction using FRAX®: a 10-year follow-up survey of the Japanese Population-Based Osteoporosis (JPOS) Cohort Study. Osteoporos Int. 2011;22(12):3037-45.

Yang S, Leslie WD, Morin SN, Lix LM. Administrative healthcare data applied to fracture risk assessment. Osteoporos Int. 2019;30(3):565-71.

KQ3a (n=3)

Cummings SR, Ferrari S, Eastell R, Gilchrist N, Jensen JE, McClung M, et al. Vertebral fractures after discontinuation of denosumab: a post hoc analysis of the randomized placebo-controlled FREEDOM trial and its extension. J Bone Miner Res. 2018;33(2):190-198.

Nakamura T, Matsumoto T, Sugimoto T, Shiraki M. Dose-response study of denosumab on bone mineral density and bone turnover markers in Japanese postmenopausal women with osteoporosis. Osteoporos Int. 2012;23(3):1131-40.

Sato Y, Iwamoto J, Honda Y. Once-weekly risedronate for prevention of hip fracture in women with Parkinson's disease: a randomised controlled trial. J Neurol Neurosurg Psychiatry. 2011;82(12):1390-3.

Sone T, Kon N, Gaither KW, Okubo N, Osakabe T, Nakayama Y, et al. Effects of 3-year denosumab treatment on hip structure in Japanese postmenopausal women and men with osteoporosis. Bone Rep. 2017;7:164-71.

Um MJ, Cho EA, Jung H. Combination therapy with raloxifene and alendronate for treatment of osteoporosis in elderly women. J Menopausal Med. 2017;23(1):56-62.

KQ3b (n=4)

Davis S, Simpson E, Martyn-St-James Marrissa, Wong R. Non-bisphosphonates for the prevention of osteoporotic fragility fractures (ID901). PROSPERO [Internet]. 2018 Aug 22 [cited 2020 Nov 4]. Available from: https://www.crd.york.ac.uk/prospero/display_record.php?ID=CRD42018107651.

Fink H, MacDonald R, Forte ML, Rosebush CE, Ensrud KE, Schousboe JT, et al. Long-term drug therapy and drug holidays for osteoporosis fracture prevention: a systematic review. AHRQ Comparative Effectiveness Review, No. 218. Rockville (MD): Agency for Healthcare Research and Quality; 2019.

Viswanathan M, Reddy S, Berkman N, Cullen K, Middleton JC, Nicholson WK, et al. Screening to prevent osteoporotic fractures: an evidence review for the U.S. Preventive Services Task Force. AHRQ Evidence Synthesis, No. 162. Rockville (MD): Agency for Healthcare Research and Quality; 2018.

Wen F, Du H, Ding L, Hu J, Huang Z, Huang H, et al. Clinical efficacy and safety of drug interventions for primary and secondary prevention of osteoporotic fractures in postmenopausal women: network meta-analysis followed by factor and cluster analysis. PloS One. 2020;15:e0234123.

KQ4 (n=5)

de Bekker-Grob EW, Essink-Bot M-L, Meerding WJ, Koes BW, Steyerberg EW. Preferences of GPs and patients for preventive osteoporosis drug treatment: a discrete-choice experiment. Pharmacoeconomics. 2009;27(3):211-9.

Gonnelli S, Caffarelli C, Rossi S, Di Munno O, Malavolta N, Isaia G, et al. How the knowledge of fracture risk might influence adherence to oral therapy of osteoporosis in Italy: the ADEOST study. Aging Clin Exp Res. 2016;28(3):459-68.

Hiligsmann M, Dellaert B, Dirksen C, Van d W, Watson V, Goemaere S, et al. Are Patients' Preferences Transferable Between Countries? A Cross-European Discrete-Choice Experiment to Elicit Patients' Preferences for Osteoporosis Drug Treatment. Value Health. 2014;17(7):A385.

Kastner M, Sawka A, Thorpe K, Chignel M, Marquez C, Newton D, et al. Evaluation of a clinical decision support tool for osteoporosis disease management: protocol for an interrupted time series design. Implement Sci. 2011;6:77.

Neuner J, Schapira MM, Smallwood AJ. Randomized controlled trial of a decision aid with tailored fracture risk tool delivered via a patient portal. J Gen Intern Med. 2015;30:S243.

Si L, Gu J, Palmer AJ, Tu L, Xie Y, Gu Y, et al. What is Chinese patients' preference for osteoporosis treatment? a discrete choice experiment. Osteoporos Int. 2018;29(Suppl 1):S325.

Setting:

KQ2 (n=56)

Ahmed LA, Nguyen ND, Bjornerem A, Joakimsen RM, Jorgensen L, Stormer J, et al. External validation of the Garvan nomograms for predicting absolute fracture risk: the Tromso study. PLoS One. 2014;9(9):e107695.

Ahmed LA, Schirmer H, Fonnebo V, Joakimsen RM, Berntsen GK. Validation of the cummings' risk score; how well does it identify women with high risk of hip fracture: the Tromso study. Eur J Epidemiol. 2006;21(11):815-22.

Akyea RK, McKeever TM, Gibson J, Scullion JE, Bolton CE. Predicting fracture risk in patients with chronic obstructive pulmonary disease: a UK-based population-based cohort study. BMJ Open. 2019;9(4):e024951.

Akyea RK, McKeever TM, Gibson JE, Scullion J, Bolton CE. Osteoporosis and fracture risk in patients with chronic obstructive pulmonary disease (COPD): a UK based population-based cohort study. Osteoporos Int. 2018;29(Suppl 1):S172.

Albertsson D, Mellström D, Petersson C, Thulesius H, Eggertsen R. Hip and fragility fracture prediction by 4-item clinical risk score and mobile heel BMD: a women cohort study. BMC Musculoskelet Disord. 2010;11(1):55.

Aw D, Thain J, Ali A, Aung T, Chua WM, Sahota O, et al. Predicting fracture risk in osteoporosis: the use of fracture prediction tools in an osteoporosis clinic population. Postgrad Med J. 2016;92(1087):267-70.

Bhattacharya P, Altai Z, Qasim M, Viceconti M. A multiscale model to predict current absolute risk of femoral fracture in a postmenopausal population. Biomech Model Mechanobiol. 2019;18(2):301-18.

Biver E, Durosier-Izart C, Chevalley T, van Rietbergen B, Rizzoli R, Ferrari S. Evaluation of radius microstructure and areal bone mineral density improves fracture prediction in postmenopausal women. J Bone Miner Res. 2018;33(2):328-37.

Blackburn TD, Howard DB, Leib ES. Utility of spine bone mineral density in fracture prediction within FRAX. J Clin Densitom. 2013;16(1):81-6.

Bonaccorsi G, Fila E, Messina C, Maietti E, Ulivieri FM, Caudarella R, et al. Comparison of trabecular bone score and hip structural analysis with FRAX® in postmenopausal women with type 2 diabetes mellitus. Aging Clin Exp Res. 2017;29(5):951-7.

Bonaccorsi G, Messina C, Cervellati C, Maietti E, Medini M, Rossini M, et al. Fracture risk assessment in postmenopausal women with diabetes: comparison between DeFRA and FRAX tools. Gynecol Endocrinol. 2018;34(5):404-8.

Briot K, Paternotte S, Kolta S, Eastell R, Felsenberg D, Reid DM, et al. Frax®: prediction of major osteoporotic fractures in women from the general population: the OPUS study. PloS one. 2013;8(12):e83436.

Byberg L, Gedeborg R, Cars T, Sundstrom J, Berglund L, Kilander L, et al. Prediction of fracture risk in men: a cohort study. J Bone Miner Res. 2012;27(4):797-807.

Chen J-F, Yu S-F, Hsu C-Y, Chiu W-C, Wu C-H, Lai H-M, et al. The role of bone mineral density in therapeutic decision-making using the Fracture Risk Assessment Tool (FRAX): a sub-study of the Taiwan OsteoPorosis Survey (TOPS). Arch Osteoporos. 2019;14(1):101.

Chen SJ, Chen YJ, Cheng CH, Hwang HF, Chen CY, Lin MR. Comparisons of different screening tools for identifying fracture/osteoporosis risk among community-dwelling older people. Medicine. 2016;95(20):e3415.

Cheung C-L, Lam M-T, Sing C-W, Li G, Kung A, Tan K. Development and validation of a risk score to predict the first hip fracture in the oldest old: Retrospective cohort studies. J Bone Miner Res. 2019;34(Suppl 1):344.

Edwards MH, Jameson K, Denison H, Harvey NC, Sayer AA, Dennison EM, et al. Clinical risk factors, bone density and fall history in the prediction of incident fracture among men and women. Bone. 2013;52(2):541-7.

Engels A, Reber KC, Lindlbauer I, Rapp K, Buchele G, Klenk J, et al. Osteoporotic hip fracture prediction from risk factors available in administrative claims data - A machine learning approach. PloS ONE. 2020;15(5):e0232969.

FitzGerald G, Compston JE, Chapurlat RD, Pfeilschifter J, Cooper C, Hosmer DW, Jr., et al. Empirically based composite fracture prediction model from the Global Longitudinal Study of Osteoporosis in Postmenopausal Women (GLOW). J Clin Endocrinol Metab. 2014;99(3):817-26.

Forgetta V, Keller-Baruch J, Forest M, Durand A, Bhatnagar S, Kemp JP, et al. Development of a polygenic risk score to improve screening for fracture risk: A genetic risk prediction study. PLoS Med. 2020;17(7):e1003152.

Forti P, Rietti E, Pisacane N, Olivelli V, Maltoni B, Ravaglia G. A comparison of frailty indexes for prediction of adverse health outcomes in an elderly cohort. Arch Gerontol Geriatr. 2012;54(1):16-20.

Francesco L, Elisa B, Raffaella M, Alessandro P, Iacopo C, Giampiero M, et al. Assessing risk of osteoporotic fractures in primary care: development and validation of the FRA-HA algorithm. Calcif Tissue Int. 2017;100(6):537-49.

Gudmundsson HT, Hansen KE, Halldorsson BV, Ludviksson BR, Gudbjornsson B. Clinical decision support system for the management of osteoporosis compared to NOGG guidelines and an osteology specialist: a validation pilot study. BMC Med Inform Decis Mak. 2019;19(1):27.

Hars M, Biver E, Chevalley T, Herrmann F, Rizzoli R, Ferrari S, et al. Low lean mass predicts incident fractures independently from FRAX: a prospective cohort study of recent retirees. J Bone Miner Res. 2016;31(11):2048-56.

Hippisley-Cox J, Coupland C. Derivation and validation of updated Qfracture algorithm to predict risk of osteoporotic fracture in primary care in the United Kingdom: prospective open cohort study. BMJ. 2012;344:e3427.

Hoff M, Meyer HE, Skurtveit S, Langhammer A, Sogaard AJ, Syversen U, et al. Validation of FRAX and the impact of self-reported falls among elderly in a general population: the HUNT study, Norway. Osteoporos Int. 2017;28(10):2935-44.

Johansson H, Kanis JA, Oden A, Johnell O, McCloskey E. BMD, clinical risk factors and their combination for hip fracture prevention. Osteoporos Int. 2009;20(10):1675-82.

Jonasson GB, Sundh V, Hakeberg M, Ahlqwist M, Lissner L, Hange D. Evaluation of clinical and radiographic indices as predictors of osteoporotic fractures: a 10-year longitudinal study. Oral Surg Oral Med Oral Pathol Oral Radio. 2018;125(5):487-94.

Kral R, Osima M, Borgen TT, Vestgaard R, Richardsen E, Bjornerem A. Increased cortical porosity and reduced cortical thickness of the proximal femur are associated with nonvertebral fracture independent of FRAX and Garvan estimates in postmen opausal women. Osteoporos Int. 2018;29(Suppl 1):S465-S6.

Kuzma M, Hans D, Koller T, Nemethova E, Jackuliak P, Killinger Z, et al. Different thresholds for the FRAX and TBS-adjusted FRAX used for prediction of clinical fractures in osteopenic post-menopausal women without previous fractures. Osteoporos Int. 2017;28(Suppl 1):S352.

Kuzma M, Hans D, Koller T, Nemethova E, Jackuliak P, Killinger Z, et al. Less strict intervention thresholds for the FRAX and TBS-adjusted FRAX predict clinical fractures in osteopenic postmenopausal women with no prior fractures. J Bone Miner Metab. 2018;36(5):580-8.

Kuzma M, Hans D, Koller T, Nemethova E, Killinger Z, Resch H, et al. Less strict high- versus low-risk thresholds for the FRAX and TBS-adjusted FRAX predict clinical fractures in osteopenic postmenopausal women with no prior fractures: a 5-year longitudinal cohort study. J Miner Stoffwechs Muskuloskelet Erkrank. 2016;23(Suppl 1):5.

Lundin H, Saaf M, Strender LE, Nyren S, Johansson SE, Salminen H. Gait speed and one-leg standing time each add to the predictive ability of FRAX. Osteoporos Int. 2017;28(1):179-87.

McCloskey E, Compston JE, Cooper C, Harvey NC, Johansson H, Oden A, et al. Impact of fracture risk assessment tools on proportions of women without prior fracture identified at risk and treated-lessons from the sign guidance. Osteoporos Int. 2016;27(Suppl 1):S382-S3.

McCloskey E, Johansson H, Oden A, Harvey N, Eastell R, Gossiel F, et al. Serum CTX predicts hip fracture risk independently of FRAX (without BMD) but not of BMD: a post hoc analysis from the Sheffield hip facture study. Osteoporos Int. 2018;29(Suppl 1):S355-S6.

Morales-Torres J, Morales-Vargas J, Tinajero-Patino C. Case finding in osteoporosis: is there still a role for osteorisk? Osteoporos Int. 2017;28(Suppl 1):S562-S3.

Muratore M, Casciaro S, Pisani P, Peccarisi M, Greco A, Renna MD, et al. Fracture risk prediction: comparative evaluation of ultrasound-based fragility score and DXA-measured BMD against FRAX. Ann Rheum Dis. 2015;74(Suppl 2):65.

Osima M, Shigdel R, Joakimsen RM, Eriksen EF. Why are additional women with fracture identified by measurement of cortical porosity than identified by FRAX? J Bone Miner Res. 2015;30(Suppl 1):S413.

Popp AW, Meer S, Krieg MA, Perrelet R, Hans D, Lippuner K. Bone mineral density (BMD) and vertebral trabecular bone score (TBA) for the identification of elderly women at high risk for fracture: the SEMOF cohort study. Eur Spine J. 2016;25(11):3432-8.

Reber KC, Konig HH, Becker C, Rapp K, Buchele G, Machler S, et al. Development of a risk assessment tool for osteoporotic fracture prevention: a claims data approach. Bone. 2018;110:170-6.

Rubin KH, Abrahamsen B, Friis-Holmberg T, Hjelmborg JV, Bech M, Hermann AP, et al. Comparison of different screening tools (FRAX®, OST, ORAI, OSIRIS, SCORE and age alone) to identify women with increased risk of fracture. A population-based prospective study. Bone. 2013;56(1):16-22.

Rubin KH, Holmberg T, Rothmann MJ, Brixen K, Hoejberg M, Gram J, et al. The ROSE study: Risk-stratified Osteoporosis Strategy Evaluation effectiveness of a two-step population based osteoporosis screening programme using FRAX: a randomised prospective population-based study. J Bone Miner Res. 2017;32(Suppl 1):S29-S30.

Rubin KH, Moller S, Holmberg T, Bliddal M, Sondergaard J, Abrahamsen B. A new fracture risk assessment tool (FREM) based on public health registries. J Bone Miner Res. 2018;33(11):1967-79.

Sambrook PN, Flahive J, Hooven FH, Boonen S, Chapurlat R, Lindsay R, et al. Predicting fractures in an international cohort using risk factor algorithms without BMD. J Bone Miner Res. 2011;26(11):2770-7.

Shahla A. Validity of bone mineral density and who fracture risk assessment thresholds in hip fractures. Arch Iran Med. 2011;14(5):352-4.

Su FM, Liu DH, Chen JF, Yu SF, Chiu WC, Hsu CY, et al. Development and validation of an Osteoporosis Self-assessment Tool for Taiwan (OSTAi) postmenopausal women-a sub-study of the Taiwan Osteoporosis Survey (TOPS). PLoS One. 2015;10(6):e0130716.

Subasinghe HWAS, Lekamwasam S, Ball P, Morrissey H, Morrissey EI. Performance of Sri Lankan FRAX algorithm without bone mineral density and with Quantitative Ultrasound data input. Ceylon Med J. 2019;64(1):17-24.

Sund R, Honkanen R, Johansson H, Oden A, McCloskey E, Kanis J, et al. Evaluation of the FRAX model for hip fracture predictions in the population-based Kuopio osteoporosis risk factor and prevention study (OSTPRE). Calcif Tissue Int. 2014;95(1):39-45.

Sundh V, Hange D, Ahlqwist M, Hakeberg M, Lissner L, Jonasson G. FRAX and mandibular sparse trabeculation as fracture predictors: a longitudinal study from 1980 to 2002. Eur J Oral Sci. 2017;125(2):135-40.

Sundh V, Jonasson G. FRAX and mandibular sparse trabeculation as fracture predictors: a longitudinal study 1980-2002. Osteoporos Int. 2016;27(Suppl 1):S257.

Supawattanabodee B, Wiriyasirivaj B. Utility of bayesian logistic regression model in the development of a clinical risk score index for screening of osteoporosis in menopausal women. J Med Assoc Thai. 2018;101(Suppl 8):S193-S9.

Thuy Trinh LT, Achat H, Loh SM, Pascoe R, Assareh H, Stubbs J, et al. Validity of routinely collected data in identifying hip fractures at a major tertiary hospital in Australia. Health Inf Manag. 2018;47(1):38-45.

Tortora R, Imperatore N, Capone P, Gerbino N, Rea M, Affinito G, et al. FRAX score can be used to avoid superfluous dxa scans in detecting osteoporosis in celiac disease: accuracy of the FRAX score in celiac patients. J Clin Densitom. 2018;21(3):315-21.

van Staa TP, Geusens P, Kanis JA, Leufkens HG, Gehlbach S, Cooper C. A simple clinical score for estimating the long-term risk of fracture in post-menopausal women. QJM. 2006;99(10):673-82.

Wilson C, Loughrey PB, Cummings B, McNally C, Lindsay JR. Does implementation of fracture risk thresholds for access to dual energy densitometry (dxa), using frax, impact management outcomes? Ir J Med Sci. 2016;185(Suppl 7):S385.

Wilczek ML, Bhatta L, Brumpton BM, Freyschuss B, Brismar TB. Screening for women with increased risk of fragility fractures in a general female population using digital X-ray radiogrammetry (DXR). Maturitas. 2021;144:60-7.

Abstracts, inadequate to assess risk of bias

KQ2 (n=6)

Amin S, Atkinson E, Achenbach S, Therneau T, Khosla S. A comprehensive assessment of comorbidities to predict hip fracture using a population-based cohort. J Bone and Miner Res. 2016;31(Suppl 1):S362.

Azagra Ledesma R, Prieto-Alhambra D, Encabo Duro G, Casado Burgos E, Aguye Batista A, Diez-Perez A. [Usefulness of frax tool for the management of osteoporosis in the Spanish female population]. Med Clin (Barc). 2011;136(14):613-9.

Battalora L, Armon C, Buchacz K, Hammer J, Spear J, Brooks JT, et al. FRAX-tool fracture-risk comparison: have a little backbone! Top Antivir Med. 2017;25(Suppl 1):289s.

Leslie W, Duerksen D, Lix L, Morin S, Majumdar S, Johansson H, et al. A population-based assessment of the performance of FRAX in celiac disease: the Manitoba BMD cohort. J Bone Miner Res. 2016;31(Suppl 1):S235.

Leslie W, Majumdar S, Morin S, Lix L, Johansson H, Oden A, et al. The effect of White vs Asian ethnicity on the performance of FRAX in Canadian women: the Manitoba BMD cohort. J Bone Miner Res. 2016;31(Suppl 1):S235-6.

Olmos JM, Hernandez JL, Gonzalez JL, Martinez J, Pariente E, Sierra I, et al. Predictive and discriminatory capacity of the frax tool in Spanish postmenopathic women: a preliminary study. Calcif Tissue Int. 2017;100 (Suppl 1):S86.

KQ4 (n=3)

Blalock SJ, Dixon M. Effect of quantitative information concerning medication side-effects on risk perception. Arthritis Rheumatol. 2016;68(Suppl 10):1387-8.

Gopisetty P, Gupta A. Understanding patient preferences for osteoporosis treatments. Osteoporos Int. 2017;28(Suppl 1):S326.

Jiang C, Peters E, Fraenkel L. The influence of testimonials on patient decision-making. Arthritis Rheumatol [Internet]. 2017[cited 2020 Nov 27];69(Suppl 10). Available from: https://acrabstracts.org/abstract/the-influence-of-testimonials-on-patient-decision-making/.

Conference proceedings with no relevant abstracts

KQ2 (n=3)

Anonymous. Abstracts of osteoporosis conference 2016. Osteoporos Int. 2016;27(Suppl 2):609–85.

Anonymous. CAEP/ACMU 2015 scientific abstracts. CJEM. 2015;17(Suppl 2):S92-S96.

Anonymous. ISS 2017 annual meeting New York, New York. Skeletal Radiol. 2017;46:1305-14.

KQ3a (n=4)

Anonymous. Conference highlights. Mol Diagn Ther. 2010;14(4):251-8.

Anonymous. Osteoporosis conference 2014. Osteoporos Int. 2014;25(Suppl 6):S659-S726.

Anonymous. Osteoporosis conference 2018 Birmingham, UK 2 – 4 December 2018 Abstracts. Osteoporos Int. 2018;29(Suppl 2):S601-S649.

World congress on osteoporosis, osteoarthritis and musculoskeletal diseases (WCO-IOF-ESCEO 2017): Poster Abstracts. Osteoporos Int. 2017;28(Suppl 1):S127-S636.

KQ4 (n=12)

Anonymous. 2013 Annual meeting of the American Society for Bone and Mineral Research. J Bone Miner Res. 2013;28(Suppl 1).

Anonymous. 2016 ACR/ARHP annual meeting abstract supplement. Arthritis Rheumatol. 2016;68(Suppl 10).

Anonymous. 65th ASTMH annual meeting. Am J Trop Med Hyg. 2016;95(Suppl 5).

Anonymous. 20th Annual meeting of the North American Menopause Society. Menopause. 2009;16(6):1231-1266.

Anonymous. Annual European congress of rheumatology EULAR Abstracts 2013. Ann Rheum Dis. 2013;72(Suppl 3).

Anonymous. APhA2010 abstracts of contributed papers. J Am Pharm Assoc. 2010;50(2):219-313.

Anonymous. Canadian Association for Population Therapeutics 2010 conference. J Popul Ther Clin Pharmacol; 2010 Mar 28-30; Toronto, ON. 2010;17(1).

Anonymous. 44th annual meeting of the Cervical Spine Research Society. Neurosurg Focus. 2017;42(2):A1-38

Anonymous. Interdisciplinary symposium on osteoporosis 2013 - patient-centered care: developing successful bone health teams. Osteoporos Int. 2013;24(Suppl 2):S419-441.

Anonymous. ISPOR 12th Annual European Congress. Abstracts. Value Health. 2009;12(7):A221-481.

Anonymous. Proceedings of the SHBC 2013: Singapore health and biomedical congress. Ann Acad Med. 2013;42(Suppl 9).

D'Orazio AI, Maung K. 2001 Highlights from: 37th annual meeting of the American Society of Clinical Oncology Meeting. Clin Breast Cancer. 2001;2(2):252-257.

Protocols, not published or screened the full text

KQ1 (n=9)

Colon-Emeric CS, Lee R, Pieper CF, Lyles KW, Zullig LL, Nelson RE, et al. Protocol for the models of primary osteoporosis screening in men (MOPS) cluster randomized trial. Contemp Clin Trials. 2022;112:106634.
ISRCTN registry [internet]. A pragmatic randomised clinical trial of the effectiveness and cost effectiveness of targeted population screening for low bone mineral density (BMD) in the prevention of neck of femur fractures. Available from: https://trialsearchwhoint/Trial2aspx?TrialID=ISRCTN11021925.
ISRCTN registry [internet]. A pragmatic randomised controlled trial of the effectiveness and cost-effectiveness of screening for osteoporosis in older women for the prevention of fractures. Available from: https://trialsearchwhoint/Trial2aspx?TrialID=ISRCTN55814835.
JPRN registry [internet]. Osteoporosis screening using FRAX by community pharmacists and Collaborative pharmacotherapy in the osteoporotic treatment. Available from: https://trialsearchwhoint/Trial2aspx?TrialID=JPRN-UMIN000018306.
NCT registry [internet]. Risk-stratified Osteoporosis Strategy Evaluation Study (ROSE). Available from: https://clinicaltrialsgov/show/NCT01388244.
NCT registry [internet]. Models of Primary Osteoporosis Screening in Male Veterans. Available from: https://clinicaltrialsgov/show/NCT04079868. 2019.
NCT registry [internet]. Comprehensive Risk Assessment and Prevention Plan of Osteoporosis and Sarcopenia for the Elderly Participating in Community-based Long-term Care Services. Available from: https://clinicaltrialsgov/show/NCT05104034. 2021.
NCT registry [internet]. Effects of FRAX+SARC-F Pre-screening on Preventing Fragility Fracture and Fall in Community-dwelling Older Adults. Available from: https://clinicaltrialsgov/show/NCT04709393.
NTR registry [internet]. Stepped screening of fracture risk. A case finding and treatment program for women of 65 years of age and older in primary care. Available from: https://trialsearchwhoint/Trial2aspx?TrialID=NTR2430.

KQ3b (n=8)

Bastounis A, Leonardi-Bee J, Langley T, Paskins Z, Davis S, Sahota O. Assessing the effectiveness of bisphosphonates for the prevention of fragility fractures: an updated systematic review. PROSPERO [Internet]. 2020 Mar 30 [cited 2020 Nov 4]. Available from: https://www.crd.york.ac.uk/prospero/display_record.php?ID=CRD42020177155.
Cai X, Lv F, Yang W, Ji L. Association between denosumab or romosozumab therapy and risk of cardiovascular events in patients with primary osteoporosis: a meta-analysis of randomized clinical trials. PROSPERO [Internet]. 2019 Aug 8 [cited 2020 Nov 4]. Available from: https://www.crd.york.ac.uk/prospero/display_record.php?ID=CRD42019137850.
He X, Yang X, Jiangtao F, Lin G, Yongcheng H. Long-term use of bisphosphonates in postmenopausal women with osteopenia versus osteoporosis: a systematic review and network meta-analysis. PROSPERO [Internet]. 2018 Jul 20 [cited 2020 Nov 4]. Available from: https://www.crd.york.ac.uk/prospero/display_record.php?ID=CRD42018100861.
Ie-Wen Sim, Ebeling P, Buchbinder R. Bisphosphonates for preventing and treating osteoporosis in men. Cochrane Database of Systematic Reviews [Internet]. 2014 [cited 2020 Nov 4]; Issue 4. Art. No.: CD011074. Available from: https://www.cochranelibrary.com/cdsr/doi/10.1002/14651858.
CD011074/full.
Li M, Zhong M. Does the use of bisphosphonates increase the risk of esophageal cancer in osteoporosis patients compared to normal people? PROSPERO [Internet]. 2020 Apr 28 [cited 2020 Nov 4]. Availabe from: https://www.crd.york.ac.uk/prospero/display_record.php?ID=CRD42020169168.
Seeto A, Rodriguez A, Abrahamsen B, Ebeling P. Cardiovascular adverse events in patients treated with denosumab for bone related outcomes. PROSPERO [Internet]. 2019 Jul 12 [cited 2020 Nov 4]. Available from: https://www.crd.york.ac.uk/prospero/display_record.php?ID=CRD42019135414.
Zhao X, Tang M, Yan S, Zhao G. Meta-analysis of denosumab versus placebo for osteoporotic patients. PROSPERO [Internet]. 2016 Dec 25 [cited 2020 Nov 4]. Available from: https://www.crd.york.ac.uk/prospero/display_record.php?ID=CRD42017054159.
Zheng C, Hsieh S, Peterson J, Tugwell P, Singh J, Wells G. Interventions for the treatment and prevention of osteoporosis in postmenopausal women. PROSPERO [Internet]. 2019 Nov 26 [cited 2020 Nov 4]. Available from: https://www.crd.york.ac.uk/prospero/display_record.php?ID=CRD42019147448.

Registry for studies included or excluded by full text

KQ3a (n=23)

ClinicalTrials.gov [Internet]. Bethesda (MD): National Library of Medicine (US). 2000 Feb 29. Identifier NCT00043186, Determine the Efficacy, Safety and Tolerability of Denosumab (AMG 162) in the Treatment of Postmenopausal Women With Low Bone Mineral Density; 2002 Aug 7 [cited 2020 Jul 27]. Available from: https://clinicaltrials.gov/ct2/show/NCT00043186.

ClinicalTrials.gov [Internet]. Bethesda (MD): National Library of Medicine (US). 2000 Feb 29. Identifier NCT00089791, A Study to Evaluate Denosumab in the Treatment of Postmenopausal Osteoporosis; 2004 Aug 16 [cited 2020 Jul 27]. Available from: https://clinicaltrials.gov/show/NCT00089791.

ClinicalTrials.gov [Internet]. Bethesda (MD): National Library of Medicine (US). 2000 Feb 29. Identifier NCT00091793, Study to Evaluate AMG 162 in the Prevention of Postmenopausal Osteoporosis; 2004 Sept 21 [cited 2020 Jul 27]. Available from: https://clinicaltrials.gov/ct2/show/NCT00091793.

ClinicalTrials.gov [Internet]. Bethesda (MD): National Library of Medicine (US). 2000 Feb 29. Identifier NCT00092027, A Study to Evaluate the Safety and Tolerability of MK0217 in Women (0217-219); 2004 Sept 24 [cited: 2020 Jul 27]. Available from: https://clinicaltrials.gov/ct2/show/NCT00092027.

ClinicalTrials.gov [Internet]. Bethesda (MD): National Library of Medicine (US). 2000 Feb 29. Identifier NCT00132808, Zoledronic Acid in the Prevention of Bone Loss in Postmenopausal Women With Osteopenia, 45 Years of Age and Older; 2005 Aug 22 [cited 2020 Jul 27]. Available from: https://clinicaltrials.gov/ct2/show/NCT00132808.

ClinicalTrials.gov [Internet]. Bethesda (MD): National Library of Medicine (US). 2000 Feb 29. Identifier NCT00306189, A Randomized, Double-blind, Placebo-controlled, Dose Response Study of AMG 162 (Denosumab) in Japanese Postmenopausal Osteoporotic Subjects; 2006 Mar 23 [cited 2020 Jul 27]. Available from: https://clinicaltrials.gov/ct2/show/NCT00306189.

ClinicalTrials.gov [Internet]. Bethesda (MD): National Library of Medicine (US). 2000 Feb 29. Identifier NCT00353080, Risedronate In The Prevention Of Osteoporosis In Osteopenic Postmenopausal Women; 2006 Jul 17 [cited 2020 Jul 27]. Available from: https://clinicaltrials.gov/ct2/show/NCT00353080.

ClinicalTrials.gov [Internet]. Bethesda (MD): National Library of Medicine (US). 2000 Feb 29. Identifier NCT00402441, Risedronate in the Prevention of Osteoporosis in Postmenopausal Women; 2006 Nov 22 [cited 2020 Jul 27]. Available from: https://clinicaltrials.gov/ct2/show/NCT00402441.

ClinicalTrials.gov [Internet]. Bethesda (MD): National Library of Medicine (US). 2000 Feb 29. Identifier NCT00439647, Efficacy in Reducing Fractures and Safety of Zoledronic Acid in Men With Osteoporosis; 2007 Feb 23 [cited 2020 Jul 27]. Available from: https://clinicaltrials.gov/ct2/show/NCT00439647

ClinicalTrials.gov [Internet]. Bethesda (MD): National Library of Medicine (US). 2000 Feb 29. Identifier NCT00471237, A Phase II Study Evaluating SB-751689 in Post-Menopausal Women With Osteoporosis; 2007 May 9 [cited 2020 Jul 27]. Available from: https://clinicaltrials.gov/ct2/show/NCT00471237.

ClinicalTrials.gov [Internet]. Bethesda (MD): National Library of Medicine (US). 2000 Feb 29. Identifier NCT00523341, Extension Study to Evaluate the Long Term Safety and Efficacy of Denosumab in the Treatment of Osteoporosis; 2007 Aug 31 [cited 2020 Jul 27]. Available from: https://clinicaltrials.gov/ct2/show/NCT00523341.

ClinicalTrials.gov [Internet]. Bethesda (MD): National Library of Medicine (US). 2000 Feb 29. Identifier NCT00619957, Efficacy and Safety of Risedronate Therapy Administered 35 mg Once A Week in Men With Osteoporosis; 2008 Feb 21 [cited 2020 Jul 27]. Available from: https://clinicaltrials.gov/ct2/show/NCT00619957.

ClinicalTrials.gov [Internet]. Bethesda (MD): National Library of Medicine (US). 2000 Feb 29. Identifier NCT00980174, Study to Compare the Efficacy and Safety of DenosumAb Versus Placebo in Males With Osteoporosis; 2009 Sept 18 [cited 2020 Jul 27]. Available from: https://clinicaltrials.gov/ct2/show/NCT00980174.

ClinicalTrials.gov [Internet]. Bethesda (MD): National Library of Medicine (US). 2000 Feb 29. Identifier NCT01457950, A Study in Korean Postmenopausal Women With Osteoporosis to Evaluate the Efficacy and Safety of Denosumab; 2011 Oct 24 [cited 2020 Jul 27]. Available form: https://clinicaltrials.gov/ct2/show/NCT01457950.

ClinicalTrials.gov [Internet]. Bethesda (MD): National Library of Medicine (US). 2000 Feb 29. Identifier NCT01495000, A Study in Indian Postmenopausal Women With Osteoporosis to Evaluate the Efficacy and Safety of Denosumab; 2011 Dec 19 [cited 2020 Jul 27]. Available from: https://clinicaltrials.gov/ct2/show/NCT01495000.

ClinicalTrials.gov [Internet]. Bethesda (MD): National Library of Medicine (US). 2000 Feb 29. Identifier NCT03822078, Study to Evaluate the Safety, Tolerability, Pharmacokinetics, and Pharmacodynamics of AMG 162 in Japanese

Postmenopausal Women; 2019 Jan 30 [cited 2020 Jul 27]. Available from: https://clinicaltrials.gov/ct2/show/NCT03822078.
Clinical Trials Registry India [Internet]. New Delhi: database publisher (India). 2007 Jun 20. Identifier CTRI/2011/12/002257, A six month randomized, double-blind, placebo controlled study across centres in India to study the safety and efficacy of Denosumab in post menopausal women with osteoporosis; 2011 Dec 19 [cited 2020 Jul 27]. Available from: https://apps.who.int/trialsearch/Trial2.aspx?TrialID=CTRI/2011/12/002257.
Clinical Trials Registry India [Internet]. New Delhi: database publisher (India). 2007 Jun 20. Identifier CTRI/2016/10/007385, ROLE OF ZOLEDRONIC ACID IN OSTEOPROTIC PATIENTS. 2016 Oct 10 [cited 2020 Jul 27]. Available from: https://apps.who.int/ trialsearch/Trial2.aspx?TrialID=CTRI/2016/10/007385.
EU Clinical Trials Register [Internet]. Amsterdam: European Medicines Agency (Netherlands). 1995. Identifier EUCTR2004-004131-57-FI, A two year multicenter, randomized, double-blind, placebo-controlled, parallel group study to evaluate the fracture efficacy and safety of intravenous zoledronic acid 5 mg annually for the treatment of osteoporosis in men; 2006 Nov 6 [cited 2020 Jul 27]. Available from: https://apps.who.int/trialsearch/Trial2.aspx? TrialID=EUCTR2004-004131-57-FI.
EU Clinical Trials Register [Internet]. Amsterdam: European Medicines Agency (Netherlands). 1995. Identifier EUCTR2006-005893-37-IT, A one year multicenter, randomized, double-blind, placebocontrolled, parallel group study to evaluate the efficacy and safety of a single intravenous 5 mg dose zoledronic acid for the treatment of osteoporosis in men – ND; 2007 April 3 [cited 2020 Jul 27]. Available from: https://apps.who.int/trialsearch/Trial2.aspx? TrialID=EUCTR2006-005893-37-IT
EU Clinical Trials Register [Internet]. Amsterdam: European Medicines Agency (Netherlands). 1995. Identifier EUCTR2006-000635-94-GB, A Phase II, randomized, double-blind, parallel group study to examine bone quality and bone mineral density changes following treatment with SB-462795 (40mg, 80mg, 120mg or 160mg), alendronate or placebo in postmenopausal women with osteoporosis; 2006 April 13 [cited 2020 Jul 27]. Available from: https://apps.who.int/trialsearch/Trial2.aspx?TrialID=EUCTR2006-000635-94-GB.
EU Clinical Trials Register [Internet]. Amsterdam: European Medicines Agency (Netherlands). 1995. Identifier EUCTR2008-005991-28-ES, A Randomized, Placebo-controlled, Multi-dose Phase 2 Study to Determine the Efficacy, Safety and Tolerability of AMG 785 in the Treatment of Postmenopausal Women with Low Bone Mineral Density / Estudio de fase 2 aleatorizado, controlado con placebo, de dosis múltiple para determinar la eficacia, la seguridad y la tolerabilidad de AMG 785 en el tratamiento de mujeres posmenopáusicas con baja densidad mineral ósea; 2009 May 25 [cited 2020 Jul 27]. Available from: https://apps.who.int/trialsearch/ Trial2.aspx?TrialID=EUCTR2008-005991-28-ES.
EU Clinical Trials Register [Internet]. Amsterdam: European Medicines Agency (Netherlands). 1995. Identifier EUCTR2009-010205-37-FR, A Multicenter, Randomized, Double-Blind, Placebo Controlled Study to Compare the Efficacy and Safety of Denosumab versus Placebo in Males with Low Bone Mineral Density; 2009 Sept 7 [cited 2020 Jul 27]. Available from: https://apps.who.int/trialsearch/Trial2.aspx?TrialID=EUCTR2009-010205-37-FR.

<u>Excluded at second level screening – selection of most relevant, up-to-date, and comprehensive</u>

KQ2 – Not a Canadian study, for update (n=12)

Chapurlat R, Bui M, Sornay-Rendu E, Zebaze R, Delmas PD, Liew D, et al. Deterioration of Cortical and Trabecular Microstructure Identifies Women With Osteopenia or Normal Bone Mineral Density at Imminent and Long-Term Risk for Fragility Fracture: A Prospective Study. J Bone Miner Res. 2020;35(5):833-44.
Crandall CJ, Larson J, Cauley JA, Schousboe JT, LaCroix AZ, Robbins JA, et al. Do Additional Clinical Risk Factors Improve the Performance of Fracture Risk Assessment Tool (FRAX) Among Postmenopausal Women? Findings From the Women's Health Initiative Observational Study and Clinical Trials. JBMR Plus. 2019;3(12):e10239.
Dagan N, Elnekave E, Barda N, Bregman-Amitai O, Bar A, Orlovsky M, et al. Automated opportunistic osteoporotic fracture risk assessment using computed tomography scans to aid in FRAX underutilization. Nat Med. 2020;26(1):77-82.
de Abreu LLF, Holloway-Kew KL, Sajjad MA, Kotowicz MA, Pasco JA. FRAX (Australia) scores in women with impaired fasting glucose and diabetes. Bone Rep. 2019;11:100223.

Harvey NC, Kanis JA, Liu E, Cooper C, Lorentzon M, Bea JW, et al. Predictive Value of DXA Appendicular Lean Mass for Incident Fractures, Falls, and Mortality, Independent of Prior Falls, FRAX, and BMD: Findings from the Women's Health Initiative (WHI). J Bone Miner Res. 2021;36(4):654-61.

Holloway-Kew KL, Zhang Y, Betson AG, Anderson KB, Hans D, Hyde NK, et al. How well do the FRAX (Australia) and Garvan calculators predict incident fractures? Data from the Geelong Osteoporosis Study. Osteoporos Int. 2019;30(10):2129-39.

Kim H, Kim JH, Kim MJ, Hong AR, Choi H, Ku E, et al. Low Predictive Value of FRAX Adjusted by Trabecular Bone Score for Osteoporotic Fractures in Korean Women: A Community-Based Cohort Study. Endocrinol Metab (Seoul, Korea). 2020;35(2):359-66.

Lu T, Forgetta V, Keller-Baruch J, Nethander M, Bennett D, Forest M, et al. Improved prediction of fracture risk leveraging a genome-wide polygenic risk score. Genome Med. 2021;13(1):16.

Pickhardt PJ, Graffy PM, Zea R, Lee SJ, Liu J, Sandfort V, et al. Automated Abdominal CT Imaging Biomarkers for Opportunistic Prediction of Future Major Osteoporotic Fractures in Asymptomatic Adults. Radiology. 2020;297(1):64-72.

Pluskiewicz W, Adamczyk P, Drozdzowska B. Height loss in postmenopausal women-do we need more for fracture risk assessment? Results from the GO Study. Osteoporos Int. 2021.

Tamaki J, Iki M, Sato Y, Winzenrieth R, Kajita E, Kagamimori S. Does Trabecular Bone Score (TBS) improve the predictive ability of FRAX for major osteoporotic fractures according to the Japanese Population-Based Osteoporosis (JPOS) cohort study? J Bone Miner Res. 2019;37(1):161-70.

Xiao X, Wu Q. The Utility of Genetic Risk Score to Improve Performance of FRAX for Fracture Prediction in US Postmenopausal Women. Calcif Tissue Int. 2021.

KQ2 – Canadian study in update but overlapping population with included study (n=6)

Bisson EJ, Finlayson M, Ekuma O, Marrie RA, Leslie WD. Accuracy of FRAX in people with multiple sclerosis: A Manitoba BMD registry-based cohort study. J Bone Miner Res. 2018;33(Supplement 1):284.

Leslie WD, Lix LM, Binkley N. Targeted vertebral fracture assessment for optimizing fracture prevention in Canada. Arch Osteoporos. 2020;15(1):65.

Leslie WD, Morin SN, Lix LM, Binkley N. Comparison of treatment strategies and thresholds for optimizing fracture prevention in Canada: a simulation analysis. Arch Osteoporos. 2019;15(1):4.

Leslie WD, Morin SN, Lix LM, Binkley N. Impact of spine-hip discordance on fracture risk assessment and treatment qualification in Canada: the Manitoba BMD registry. Arch Osteoporos. 2020;15(1):85.

Leslie WD, Morin SN, Lix LM, Martineau P, Bryanton M, McCloskey EV, et al. Fracture prediction from self-reported falls in routine clinical practice: a registry-based cohort study. Osteoporos Int. 2019;30(11):2195-203.

Li G, Leslie WD, Kovacs CS, Prior J, Josse RG, Towheed T, et al. Combining Frailty and Trabecular Bone Score Did Not Improve Predictive Accuracy in Risk of Major Osteoporotic Fractures. J Bone Miner Res. 2020;35(6):1058-64.

KQ3b (n=18)

Anagnostis P, Paschou SA, Mintziori G, Ceausu I, Depypere H, Lambrinoudaki I, et al. Drug holidays from bisphosphonates and denosumab in postmenopausal osteoporosis: EMAS position statement. Maturitas. 2017;101:23-30.

Cai D, Qin J, Chen G, Feng W, Liu J. Bisphosphonates use and risk of gastric cancer: an updated meta-analysis of cohort and case-control studies. Minerva Med. 2017;108:464-72.

Deng Y, Zhang Z, Jia X, Cheng W, Zhou X, Liu Y, et al. Oral bisphosphonates and incidence of cancers in patients with osteoporosis: a systematic review and meta-analysis. Arch Osteoporos. 2018;14:1.

Dennison EM, Cooper C, Kanis JA, Bruyere O, Silverman S, McCloskey E, et al. Fracture risk following intermission of osteoporosis therapy. Osteoporos Int. 2019;30:1733-43.

Diker-Cohen T, Rosenberg D, Avni T, Shepshelovich D, Tsvetov G, Gafter-Gvili A. Risk for infections during treatment with denosumab for osteoporosis: a systematic review and meta-analysis. J Clin Endocrinol Metab. 2020;105:01.

Ding LL, Wen F, Wang H, Wang DH, Liu Q, Mo YX, et al. Osteoporosis drugs for prevention of clinical fracture in white postmenopausal women: a network meta-analysis of survival data. Osteoporos Int. 2020;31:961-71.

Gu HF, Gu LJ, Wu Y, Zhao XH, Zhang Q, Xu ZR, et al. Efficacy and safety of denosumab in postmenopausal women with osteoporosis: a meta-analysis. Medicine. 2015;94:e1674.

Huang BT, Huang FY, Gui YY, Peng Y, Liu W, Huang KS, et al. Association between bisphosphonates therapy and incident myocardial infarction: meta-analysis and trial sequential analysis. J Cardiovasc Pharmaco 2015;66:468-77.
Jin YZ, Lee JH, Xu B, Cho M. Effect of medications on prevention of secondary osteoporotic vertebral compression fracture, non-vertebral fracture, and discontinuation due to adverse events: a meta-analysis of randomized controlled trials. BMC Musculoskelet Disord. 2019;20:399.
Khow KS, Shibu P, Yu SC, Chehade MJ, Visvanathan R. Epidemiology and postoperative outcomes of atypical femoral fractures in older adults: a systematic review. J Nutr Health Aging. 2017;21:83-91.
Lee S, Yin RV, Hirpara H, Lee NC, Lee A, Llanos S, et al. Increased risk for atypical fractures associated with bisphosphonate use. Fam Pract. 2015;32:276-81.
Liu GF, Wang ZQ, Liu L, Zhang BT, Miao YY, Yu SN. A network meta-analysis on the short-term efficacy and adverse events of different anti-osteoporosis drugs for the treatment of postmenopausal osteoporosis. J Cell Biochem. 2018;119:4469-81.
Liu L, Li C, Yang P, Zhu J, Gan D, Bu L, et al. Association between alendronate and atypical femur fractures: a meta-analysis. Endocr Connect. 2015;4:58-64.
Wang G, Sui L, Gai P, Li G, Qi X, Jiang X. The efficacy and safety of vertebral fracture prevention therapies in post-menopausal osteoporosis treatment: which therapies work best? A network meta-analysis. Bone Joint Res. 2017;6:452-63.
Wen F, Du H, Ding L, Hiu J, Huang Z, Huang H, et al. Clinical efficacy and safety of drug interventions for primary and secondary prevention of osteoporotic fractures in postmenopausal women: a network meta-analysis followed by a factor and cluster analysis. PloS One. 2020;15:e0234123.
Wright E, Schofield PT, Molokhia M. Bisphosphonates and evidence for association with esophageal and gastric cancer: a systematic review and meta-analysis. BMJ Open. 2015;5:e007133.
Yang XC, Deng ZH, Wen T, Luo W, Xiao WF, Zhao RB, et al. Network meta-analysis of pharmacological agents for osteoporosis treatment and fracture prevention. Cell Physiol Biochem. 2016;40:781-95.
Zhou M, Zheng Y, Li J, Wu J, Xu W, Cui L, et al. Upper gastrointestinal safety and tolerability of oral alendronate: a meta-analysis. Exp Ther Med. 2016;11:289-96.

KQ4 (n=2)

Billington EO, Feasel AL, VanDyke JL, Kline GA. Group medical consultation for osteoporosis: a prospective pilot study of patient experience in Canadian tertiary care. Br J Gen Pract. 2020;70(700):e801-e8.
Wilton-Clark MS, Feasel AL, Kline GA, Billington EO. Autonomy begets adherence: decisions to start and persist with osteoporosis treatment after group medical consultation. Archives of osteoporosis. 2020;15(1):138.

Full text not retrievable:

KQ1 (n=6)

Borgstrom F, Jonsson E, Harvey N, Shepstone L, Lenaghan E, Clarke S, et al. Cost-effectiveness evaluation of a screening programme for fracture risk in UK. J Bone Miner Res. 2018;33(Suppl 1):398-9.
Condurache C, Shepstone L, Cooper C, Harvey N, Kanis J, Johansson H, et al. Does screening for high hip fracture risk impact on falls risk? A post hoc analysis from the SCOOP study. Calcif Tissue Int. 2019;104(Suppl 1):S19.
Cooper C. Osteoporosis screening and fracture risk reduction. Osteoporos Int. 2019;30(Suppl 2):S161.
Curtis EM. Is screening for osteoporosis useful? Osteoporos Int. 2022;32(Suppl 1):S80.
Merlijn T, Swart K, Netelenbos C, Elders P. The effect of screening of high fracture risk and subsequent treatment on osteoporotic fractures: A systematic review and meta-analysis. J Bone Miner Res. 2018;33(Suppl 1):436-7.
Swart K, Netelenbos C, Elders P. Screening of high fracture risk in primary care not effective Thomas Merlijn. J Bone Miner Res. 2018;33(Suppl 1):8.

KQ3a (n=1)

Anonymous. Osteologie Conference: OSTEOLOGIE. 2012;21(1).

KQ4 (n=1)

Duarte JWB, Susan C.; Sen, Shuvayu S. An evaluation of patients' preferences for osteoporosis medications and their attributes: The PREFER-International study. Clin Ther. 2007;29(3):488-503.

Language (not English or French):

KQ2 (n=3)

Lesnyak OM, Gladkova EN, Ershova OB, Belova KY, Bolshakova EV, Ilyin MV, et al. The ten-year probability of fractures with the FRAX tool: Which threshold for intervention to be used and how? Nauchno-Prakticheskaya Revmatologiya. 2019;57(6):626-35.

Yamamoto N, Tsuchiya H. [Development of Novel Osteoporosis Screening Methods Using FRAX]. Yakugaku Zasshi. 2019;139(1):35-8.

Ye YJ, Ge JR, Xu HJ, Chen SN, Xie LH, Li JY, et al. Combination of hip geometry mechanics and fracture risk assessment tool to predict fracture risk in middle-aged and elderly women. Chin J Tissue Eng Res. 2020;24(26):4101-5.

KQ3a (n=3)

Hadji P, Bock O, Resch H, Kraenzlin M, Wang A, Steinle T, et al. Reduction of fracture risk with denosumab among women with osteoporosis with or without need for treatment according to DVO 2009 guideline: a subgroup analysis of the FREEDOM trial. [German]. Osteologie. 2013;22(1):39-45.

Ringe J, Nitschmann S. Zoledronic acid for preventing fractures. HORIZON trial (Health Outcomes and Reduced Incidence with Zoledronic acid ONce yearly). Internist. 2008;49(4):502-4.

Skripnikova IA, Kosmatova OV, Abirova ES, Novikov VE, Murashko LM. [Experience in using Prolia in patients with postmenopausal osteoporosis in clinical practice]. Ter Arkh. 2017;89(12. Vyp. 2):190-6.
