# Supplementary material for: Screening for the primary prevention of fragility fractures among adults aged 40 years and older in primary care: systematic reviews of the effects and acceptability of screening and treatment, and the accuracy of risk prediction tools
Source: Syst Rev. 2023 Mar 21;12:51. doi: 10.1186/s13643-023-02181-w (PMC10029308; doi:10.1186/s13643-023-02181-w)
Supplement: Supplementary file 6 — Additional file 6. KQ 2-4 study characteristics. This file shows the descriptive characteristics of the included studies for key questions 2 (accuracy of screening tests), 3a (benefits of treatment), 3b (harms of treatment), and 4 (acceptability of screening/treatment). [file 13643_2023_2181_MOESM6_ESM.pdf]

Screening for the primary prevention of fragility fractures among adults aged 40 years and older in primary care: systematic reviews of the effects and acceptability of screening and treatment, and the accuracy of risk prediction tools

Michelle Gates, Jennifer Pillay (corresponding), Megan Nuspl, Aireen Wingert, Ben Vandermeer, Lisa Hartling

Affiliation: Alberta Research Centre for Health Evidence, Department of Pediatrics, University of Alberta. Edmonton Clinic Health Academy, 11405-87 Avenue NW, Edmonton, Alberta, Canada T6G 1C9. E-mail: MG: michellegates85@gmail.com; JP: jpillay@ualberta.ca; MN: megan.nuspl@ualberta.ca; AW: awingert@ualberta.ca; BV: ben.vandermeer@ualberta.ca; LH: hartling@ualberta.ca

Additional file 6. Characteristics of the included studies

Additional Table 6.1. Characteristics of cohort studies included in KQ2 on the predictive accuracy of screening tests

Author & year, Country<br>Design<br>Funding source	Source of data and participant eligibility	Participant characteristics<br>Baseline predicted risk<br>Length of follow-up	Screening tool(s)<br>Included predictors & ascertainment<br>Risk prediction & handling of missing data	Outcomes predicted & ascertainment<br>Consideration of competing risk	Calibration outcomes & analyses
Azagra 2016a [1], Spain<br><br>Prospective cohort<br><br>**Funding:** government, industry<br><br>**Related studies:** Azagra 2012 [2], Azagra 2015 [3]	**FRIDEX cohort:** random sample of 3397 Caucasian women ≥40 and ≤90 years (mean (SD) 57.2 (8.2)) referred for bone density scanning for initial study of osteoporosis or treatment follow-up from 2000 to 2010.<br><br>**Exclusion:** prior treatment with anti-osteoporosis medication, Paget's disease, bone cancer; <10 years of follow-up, died, unable to contact at follow-up	**Analyzed sample:** n = 1308 (38.5% of eligible); 100% F; mean (SD) 57.2 (8.2) years; menopausal status NR; no treatment with anti-osteoporosis drugs at baseline, some (% NR) may have been treated during follow-up<br><br>**Predicted 10-y risk:** 3.6% MOF; 0.9% hip<br><br>**Follow-up:** 10 years	FRAX-Spain (3.2) ± BMD<br><br>**Predictors:** at time of DXA scan (baseline visit), participants self-reported age, sex, height, weight (BMI), family history of hip fracture (father/mother), history of fragility fracture, smoking, alcohol risk intake, history of glucocorticoids intake, history of anti-osteoporosis medication. BMD measured at the femoral neck via DXA with T-score determination using NHANES III reference.<br><br>**Prediction:** blinded investigators used official FRAX website; unclear how many participants had missing data nor how missing data were handled	**10-year MOF (hip, humerus, forearm, clinical spine), hip fractures:** self-reported at 10-y follow-up and confirmed with medical records; fractures that could not be confirmed were excluded.<br><br>**Competing risk:** not considered; participants who died during follow-up (5.8%) were excluded.	Expected and observed fractures; O:E ratio; calibration plot<br><br>**Subgroups:** data available by quintile of predicted risk and by age category (≥ and < 65 years)<br><br>This study updates Azagra 2012 in an expanded cohort; Azagra 2015 provide similar data in slightly different (overlapping) cohort
Azagra 2016b [4], Spain<br><br>Prospective cohort<br><br>**Funding:** government<br><br>**Related studies:** none	**FROCAT cohort:** random sample (stratified by age) of 1434 Caucasian women aged ≥40 and ≤90 years who were patients of participating family physicians in Catalonia in 2001.<br><br>**Exclusion:** developed cancer during follow-up, refused participation, moved outside the study area, died, unable to contact at follow-up	**Analyzed sample:** n = 1090 (76.0% of eligible); 100% F; mean (SD) 59.1 (12.4) years; menopausal status NR; 206 (18.9%) used anti-osteoporosis drugs<br><br>**Predicted 10-y risk:** 70.2% low risk (FRAX <5%), 11.2% intermediate risk (FRAX 5 to <7.5%), 18.6% high risk (FRAX ≥7.5%) for MOF	FRAX-Spain ± BMD (FRAX with BMD calculated in a subset of 234 [21.5%] women who had a DXA scan following general practice)<br><br>**Predictors:** not reported; may be assumed to align with the FRAX tool. BMD measured via DXA, site NR.	**10-year MOF (hip, humerus, forearm, clinical spine):** self-reported during follow-up and confirmed with hospital and electronic records; fractures that could not be confirmed were excluded.	Observed fracture probability by category of predicted risk<br><br>**Subgroups:** data available by category (low, intermediate, high) of predicted risk

Author & year, Country<br>Design<br>Funding source	Source of data and participant eligibility	Participant characteristics<br>Baseline predicted risk<br>Length of follow-up	Screening tool(s)<br>Included predictors & ascertainment<br>Risk prediction & handling of missing data	Outcomes predicted & ascertainment<br>Consideration of competing risk	Calibration outcomes & analyses
		**Follow-up:** 10 years	**Prediction:** used FRAX-Spain; unclear how many participants had missing data nor how missing data were handled	**Competing risk:** not considered; participants who died during follow-up (4.7%) were excluded.	
Bolland 2011 [5], New Zealand<br><br>Retrospective cohort (RCT extension)<br><br>**Funding:** government<br><br>**Related studies:** none	1471 healthy postmenopausal women >55 years who participated in a 5-y RCT of calcium supplements, starting in 1998. Participants were free of major medical conditions, had normal lumbar spine BMD for their age (Z-score >-2), were not taking anti-osteoporosis medications (including HRT or vitamin D supplements in doses >1000 IU/day), had serum 25(OH)D levels 25 nmol/L.<br><br>**Exclusion:** missing baseline BMD, no follow-up data available	**Analyzed sample:** n = 1422 (96.7% of eligible); 100% F; mean (SD) 74.2 (4.2) years; all postmenopausal; no use of anti-osteoporosis drugs at baseline (exclusion criteria), NR during follow-up<br><br>**Predicted 10-y risk:** FRAX-BMD 8.5% (95% CI 8.2-8.8%) MOF, 3.0% (2.8-3.2%) hip; FRAX (no BMD) 11.7% (11.3-12.1%)MOF, 5.5% (5.2-5.8%) hip; Garvan 19.4% (18.7-20.1%) osteoporotic, 6.0% (5.6-6.5%) hip<br><br>**Follow-up:** mean 8.8 years	FRAX-New Zealand ± BMD<br>Garvan + BMD<br><br>**Predictors:** ascertainment unclear but appear to be self-reported on a baseline questionnaire. ***FRAX:*** age, sex, BMI, history of personal fracture, history of parental hip fracture, smoking status, glucocorticoid use, alcohol intake, presence of rheumatoid arthritis or secondary osteoporosis, femoral neck BMD T-score (ascertainment NR); ***Garvan:*** age, sex, number of falls in the past year, and number of fractures since age 50 years, femoral neck BMD T-score (ascertainment NR)<br><br>**Prediction:** Used FRAX-New Zealand and Garvan; unclear how many participants had missing data nor how missing data were handled	**10-y MOF (FRAX – shoulder, hip, forearm, clinical vertebral), osteoporotic fractures (Garvan – hip, symptomatic vertebral, forearm, metacarpal, humerus, scapula, clavicle, distal femur, proximal tibia, patella, pelvis, sternum), hip fractures:** during 5-y RCT, self-reported every 6 months and confirmed using radiographs or reports; thereafter all fractures were self-reported at 10-y follow-up.<br><br>**Competing risk:** not considered. Participants were censored at death.	Expected and observed fractures, expected fracture probability, O:E ratio, calibration plot, Hosmer-Lemeshow test<br><br>**Subgroups:** data available by decile and quintile of predicted risk, and by age category (≤70, 70-75, 75-80, >80 years)
Crandall 2019b [6], USA<br><br>Prospective cohort<br><br>**Funding:** government<br><br>**Related studies:** Crandall 2014 [7]	**Women's Health Initiative Observational Study (WHI-OS) and Clinical Trials (WHI-CT):** 90,764 postmenopausal women aged 50-79 years at baseline (1993-1998) enrolled at 40 clinical centres. The WHI-CT evaluated three clinical interventions: a low-fat eating pattern, menopausal hormone therapy, calcium + vitamin D supplementation<br><br>**Exclusion:** serious medical conditions, no information on medication use at baseline, medications known to influence osteoporosis (bisphosphonates, calcitonin, parathyroid hormone, selective	**Analyzed sample:** n = 62,723; 62,621; 64,739 for hip, MOF, and clinical fractures respectively (69.1%, 69.0%, and 71.3% of full sample); 100% F; all postmenopausal; mean (SD) 57.9 (4.1) years; 55.6% were using HRT at baseline, those on other anti-osteoporosis drugs were excluded; 46% used HRT at any time during follow-up<br><br>**Baseline 10-year risk MOF and clinical fracture (mean):** 6.3% for 50-54y to 9.9% for 60-64y MOF, 15.8% for 50-54y to 19.0% for 60-64y clinical fracture<br><br>**Baseline 10-year risk hip (mean):** FRAX (only) 0.7%; Garvan (only) 0.2%	FRAX-US (3.0) (no BMD)<br>Garvan (no BMD)<br><br>**Predictors:** at baseline, questionnaires were used to collect self-reported age, race/ethnicity, medical history (previous fractures, rheumatoid arthritis, falls in previous 12 months), medication use, parental hip fractures, smoking, alcohol intake, use of supplemental calcium and vitamin D; height and weight were measured.<br><br>**Prediction:** FRAX values were calculated by the World Health Organization Collaborating Centre for Metabolic Bone Disease (online); Garvan using published formulas. Only participants with complete data were included.	**Hip fractures:** self-reported annually (WHI-OS) or semi-annually (WHI-CT) using questionnaires. All hip fractures were confirmed by physician adjudicators using medical records.<br><br>**Competing risk:** not considered. Participants who died during follow-up were excluded from the analysis.	Observed and expected fracture probability, observed and expected fractures<br><br>**Subgroups:** data available by age category (50-54 y, 55-59 y, 60-64 y)<br><br>Crandall 2014 provides data by category of risk for FRAX (< and ≥9.3% for MOF), as annualized rates

Author & year, Country<br>Design<br>Funding source	Source of data and participant eligibility	Participant characteristics<br>Baseline predicted risk<br>Length of follow-up	Screening tool(s)<br>Included predictors & ascertainment<br>Risk prediction & handling of missing data	Outcomes predicted & ascertainment<br>Consideration of competing risk	Calibration outcomes & analyses
	estrogen receptor modulators, luteinizing hormone-releasing hormone agents somatostatins (n = 1,111), incomplete FRAX or Garvan risk factors, <10 years follow-up	**Follow-up:** 10 years			
Czerwinski 2013 [8], Poland<br><br>Retrospective cohort<br><br>**Funding:** NR<br><br>**Related studies:** none	**Cracow Medical Centre:** 5092 women aged 50-80 years from the Malopolska region, randomly selected from 100,000 patients who attended for densitometric examination between 1997 and 2001 and were capable of answering a 15-minute telephone questionnaire. To be included, women required complete medical records (n = 3350).<br><br>**Exclusion:** dementia, hearing loss, memory loss, aphasia impeding communication, questionnaire incomplete or refused further participation	**Analyzed sample:** n = 1024 (30.6% of eligible); 100% F; mean (SD) 63.8 (6.66) years; menopausal status NR; 41.7% taking anti-osteoporosis drugs at baseline, NR during follow-up<br><br>**Predicted 10-y risk (median (IQR)):** FRAX+BMD (n = 886) 5.3 (3.5-8.5)% MOF, 1.3 (0.7-2.4)% hip; FRAX (no BMD) 4.9 (3.3-7.9)%, 0.9 (0.3-2.3)% hip<br><br>**Follow-up:** mean 11 years	FRAX-Poland ± BMD<br><br>**Predictors:** at baseline, an interview questionnaire was used to collect self-reported risk factors including age, sex, personal history of fractures, hip fractures in parents, smoking, use of glucocorticoids, rheumatoid arthritis, alcohol intake, and secondary osteoporosis. Definitions were based on the online nomogram. BMD measured at the spine and/or hip via DXA, with T-score based on NHANES III reference data.<br><br>**Prediction:** NR - appear to have used the online nomogram; unclear how many participants had missing data nor how missing data were handled	**MOF (spine, distal radius, humerus, proximal femur), hip (proximal femur) fractures:** self-reported at 11-year follow-up; included all fractures, even if there were more than one per person<br><br>**Competing risk:** not considered; participants who died during follow-up (4.3%) or were lost for other reasons were excluded	Observed and expected fracture probability, observed fractures<br><br>**Subgroups:** none
Dagan 2017 [9], Israel<br><br>Retrospective cohort<br><br>**Funding:** academic<br><br>**Related studies:** none	**Clalit Health Services:** 1,054,815 members 50-90 years with at least 3 years of continuous membership to the Clalit Health Services national health fund.<br><br>**Exclusion:** lost to follow-up (but deaths were included)	**Analyzed sample:** n = 1,054,815 (100% of eligible); 54.6% F; 38.0% 50-59 y, 28.4% 60-69 y, 21.1% 70-79 y, 12.5% 80-89 y; menopausal status NR; 0.8% were on HRT at baseline, other anti-osteoporosis medications at baseline and follow-up NR<br><br>**Predicted 5-y hip fracture risk (mean (SD)):** variable by age (NR for full cohort); FRAX 0.2 (0.002)% in women 50-54 y to 6.8 (0.037)% in women 85-89 y, 0.1 (0.001)% in men 50-54 y to 3.8 (0.020)% in men 85-89 y; QFracture 0.3 (0.004)% in women 40-44 y to 18.12 (0.152)% in women 95-99 y, 0.05 (0.007)% in men 40-44 y to 18.30	FRAX-Israel (2012 version)<br>QFracture<br>Garvan<br><br>**Predictors:** electronic record data were used to collect variables at the index date or most recent documentation for chronic conditions. ***FRAX:*** age, sex, alcoholism, smoking status, parental hip fracture history, MOF history, secondary osteoporosis (type 1 diabetes, osteogenesis imperfecta, hyperthyroidism, hypogonadism, malabsorption, chronic liver disease), rheumatoid arthritis, glucocorticoid use (90 days), BMI (measured height/weight); ***QFracture:*** age, sex, BMI, alcoholism, smoking status, parental hip fracture and MOF history, major osteoporotic fracture history, history of falls,	**Hip fracture:** ascertained via record review for clinical diagnoses<br><br>**Competing risk:** not considered; participants who died during follow-up were censored at death.	Observed and expected fracture probability, observed fractures, O:E ratio, calibration plot<br><br>**Subgroups:** data available in 5-year age/sex categories and by decile of predicted risk

Author & year, Country<br>Design<br>Funding source	Source of data and participant eligibility	Participant characteristics<br>Baseline predicted risk<br>Length of follow-up	Screening tool(s)<br>Included predictors & ascertainment<br>Risk prediction & handling of missing data	Outcomes predicted & ascertainment<br>Consideration of competing risk	Calibration outcomes & analyses
		(0.192)% in men 95-99 y; Garvan 0.20 (0.004)% in women 60-64 y to 7.84 (0.134)% in women 90-94 y, 0.40 (0.004)% in men 60-64 y to 25.29 (0.160)% in men 90-94 y.<br><br>**Follow-up:** mean 4.73 years	dementia, Parkinson's, epilepsy, diabetes, other endocrine disorders, cancer history, obstructive airway disorders, cardiovascular disease, malabsorption, chronic liver or renal disorders, purchase of glucocorticoids, antidepressants, or HRT; ***Garvan:*** age, sex, BMI, fractures after age 50y, falls in past year.<br><br>**Prediction:** used full tool equations for QFracture and Garvan via their websites. Used FRAX 10-y probability charts and multiplied by 0.5 to obtain 5-y probabilities. Multiple imputation was used to impute data for those with no documentation of BMI, weight, or smoking status.		
Desbiens 2020 [10], Canada<br><br>Retrospective cohort<br><br>**Funding**: NR<br><br>**Related studies**: None	**CARTaGENE:** a population-based survey of 40 to 69-year olds, recruited between 2009-2010 (25.6% response rate from random selection of 1% of province's population)<br><br>**Exclusion:** no renal function data, advanced kidney disease (stage 4 or 5), lived in nursing homes, correctional facilities, and First Nation Reserves	**Analyzed sample:** n=19,393 (9522 non-CKD; 9114 CKD stage 2; 757 CKD stage 3); 51% F; 54 years; menopausal status NR; 2.6% on HRT and 3.6% on bisphosphonates at baseline<br><br>**Predicted 5-year risk:**<br>FRAX MOF: no-CKD 1.5 (1.0–2.2), stage 2 CKD 2.0 (1.2–2.8), stage 3 CKD 2.4 (1.8–3.6)<br>QFracture MOF: no-CKD 0.5 (0.3–0.8), stage 2 0.6 (0.3–1.1), stage 3 0.8 (0.5–1.7)<br>Garvan any fracture: no-CKD 1.8 (0.4–3.0), stage 2 CKD 2.0 (0.7–3.7), stage 3 CKD 2.3 (1.3–5.0)<br><br>**Follow-up:** 5 years	FRAX-Canadian version 4.0 (without BMD)<br>QFracture<br>Garvan (without BMD)<br><br>**Predictors**: survey included recruitment interview including a health questionnaire, undertook physical measurements (weight and BMI), and had blood samples drawn. Previous fracture via administrative database. Previous falls and parental history of fractures were not available and were set at zero. Otherwise, data was complete except for alcohol consumption (0.7% missing), smoking (0.6%), and BMI (6.4%).<br><br>**Prediction**:<br>QFracture 5-year MOF probabilities computed using 2012 version.<br>Garvan probabilities of any fracture at 5 years were computed using the full published equation.<br>FRAX - Obtained FRAX 10-year MOF probabilities were then multiplied by 0.5 to obtain 5-year MOF probabilities	**MOF (hip, wrist, shoulder, clinical spine):** provincial physician claims databases using a previously validated algorithm specifically developed for Quebec databases<br><br>**Competing risk:** not considered	Observed and predicted 5-year fracture risk by CKD stage; calibration plots.<br><br>Subgroups: age and sex

Author & year, Country<br>Design<br>Funding source	Source of data and participant eligibility	Participant characteristics<br>Baseline predicted risk<br>Length of follow-up	Screening tool(s)<br>Included predictors & ascertainment<br>Risk prediction & handling of missing data	Outcomes predicted & ascertainment<br>Consideration of competing risk	Calibration outcomes & analyses
			Analysis for calibration using a direct modeling approach, with missing data treated using 10 multiple imputation datasets generated by predictive mean matching.		
Ettinger 2012 [11], USA<br><br>Prospective cohort<br><br>**Funding:** NR<br><br>**Related studies:** none	**Osteoporotic Fracture in Men (MrOS) study:** 5994 community dwelling men ≥65 years recruited between March 2000 and April 2002 at 6 clinical centres in Birmingham, Alabama; Minneapolis, Minnesota; Palo Alto, California; the Monongahela Valley near Pittsburgh, Pennsylvania; Portland, Oregon; and San Diego, California<br><br>**Exclusion:** 101 men who used bisphosphonates in the 30 days prior to the baseline visit	**Analyzed sample:** n = 5893 (98.3% of eligible); 0% F; mean (SD) 73.6 (5.9) years; no use of bisphosphonates at baseline (exclusion criteria), 7.1% during follow-up (were censored); other anti-osteoporosis drugs NR<br><br>**Baseline 10-y risk:** 6.0% MOF, 2.4% hip in the middle quintile<br><br>**Follow-up:** mean 8.4 years	Fracture risk calculator (FRC) ± BMD<br><br>**Predictors:** at baseline, participants completed a questionnaire including age, sex, BMI, race/ethnicity, history of fracture after 45 years (excluding from a motor vehicle accident or fall from greater than standing height), parental history of hip fracture, smoking, alcohol consumption, rheumatoid arthritis; data on use of corticosteroids, medications for secondary osteoporosis (insulin or history of hypothyroidism) in past 30 days obtained from the Iowa Drug Information Service; BMD at femoral neck measured via DXA and T- and Z-scores calculated using NHANES III reference data.<br><br>**Prediction:** data were complete for all predictors for 72.9% of men. Missing data were set to null (with sensitivity analysis removing these men also conducted). Those who started bisphosphonates during follow-up were censored at initiation of treatment.	**MOF (hip, wrist, shoulder, clinical spine), hip fractures:** Self-reported on a questionnaire every 4 months (>99% response) with confirmation by radiology reports or radiographic images. Fractures caused by excessive trauma were excluded.<br><br>**Competing risk:** not considered; appears that participant observations were censored at death	Expected and observed fracture probability for the middle quintile, observed fractures, calibration plot.<br><br>**Subgroups:** data available by quintile of predicted risk
Ettinger 2013 [12], USA<br><br>Prospective cohort<br><br>**Funding:** government<br><br>**Related studies:** Gourlay 2017 [13], Orwoll 2017 [14], Harvey 2018 [15], Langsetmo 2018 [16], Buehring 2018 [17]	**Osteoporotic Fracture in Men (MrOS) study:** 5994 community dwelling men ≥65 years recruited between March 2000 and April 2002 at 6 clinical centres in Birmingham, Alabama; Minneapolis, Minnesota; Palo Alto, California; the Monongahela Valley near Pittsburgh, Pennsylvania; Portland, Oregon; and San Diego, California	**Analyzed sample:** n = 5891 (98.3% of original sample); 0% F; mean (SD) 73.6 (5.9) years; no use of bisphosphonates at baseline (exclusion criteria), 7.1% during follow-up (were censored); other anti-osteoporosis drugs NR<br><br>**Baseline 10-y risk (mean (SD)):** FRAX+BMD 7.6 (4.3)% MOF, 2.3 (3.1)% hip; FRAX (no BMD) 8.9 (4.6)% MOF, 3.5 (3.6)% hip	FRAX-US (3.3) ± BMD<br><br>**Predictors:** at baseline, participants completed a questionnaire including age, sex, ethnicity, history of fractures after age 50, rheumatoid arthritis, parental hip fracture, smoking, alcohol consumption; height and weight were measured; prescription and non-prescription medication in the past 30 days were identified using an electronic medications inventory database; BMD of total hip and subregions measured via DXA	**MOF (hip, clinical spine, forearm, shoulder), hip fractures:** self-reported on a questionnaire every 4 months (>99% response) with confirmation by radiology reports or radiographic images. All fractures were included regardless of the degree of trauma.	Expected and observed fracture probability, observed fractures, O:E ratio, calibration plot<br><br>**Subgroups:** data available by quintile of baseline risk<br><br>Gourlay 2017 direcatly compares FRAX to Garvan and QFracture. Other

Author & year, Country<br>Design<br>Funding source	Source of data and participant eligibility	Participant characteristics<br>Baseline predicted risk<br>Length of follow-up	Screening tool(s)<br>Included predictors & ascertainment<br>Risk prediction & handling of missing data	Outcomes predicted & ascertainment<br>Consideration of competing risk	Calibration outcomes & analyses
	**Exclusion:** 101 men who used bisphosphonates in the 30 days prior to the baseline visit	**Follow-up:** mean (SD) 8.4 (2.3) years	and T- and Z-scores calculated using NHANES III reference data.<br><br>**Prediction:** Calculated at the World Health Organization Collaborating Centre for Metabolic Bone Disease using online algorithm version 3.3; input values for secondary osteoporosis were set to null due to lack of information on conditions associated with bone loss. 23.9% were missing parental hip fracture information, 4.1% on corticosteroid use and secondary osteoporosis.	**Competing risk:** Kaplan-Meier product-limit method was used to calculate 10-year cumulative incidence probabilities in the presence of competing risk of mortality. Note, observations censored at the start of bisphosphonate use.	related studies do not provide any additional data of interest.
Fraser 2011 [18], Canada<br><br>Prospective cohort<br><br>**Funding:** government, foundation, industry<br><br>**Related studies:** Leslie 2011a[19]	**Canadian Multicentre Osteoporosis Study:** people living within a 50-km radius of nine Canadian cities and aged ≥50 years at study entry randomly selected from a list of residential phone numbers. 43% agreed to participate and had a baseline interview.<br><br>**Exclusion:** participants without follow-up data, who did not agree to participate, Indigenous peoples residing in northern regions of the country	**Analyzed sample:** n = 6697 (100% of those who agreed to participate); 71.3% F; mean (SD) 65.7 (8.9) years; menopausal status NR; use of anti-osteoporosis medications NR<br><br>**Predicted 10-y risk (mean (SD)) in women:** FRAX+BMD 10.8 (7.8)% MOF, 2.7 (4.8)% hip; FRAX (no BMD) 10.6 (7.1)% MOF, 2.9 (4.2)% hip<br><br>**Predicted 10-y risk (mean (SD)) in men:** FRAX+BMD 5.4 (3.2)% MOF, 1.3 (2.0)% hip; FRAX (no BMD) 5.4 (2.7)% MOF, 1.4 (1.8)% hip<br><br>**Follow-up:** 10 years	FRAX-Canada ± BMD<br><br>**Predictors:** at baseline, height and weight were measured. A baseline questionnaire was used to collect self-reported age, history of osteoporotic fractures since age 50. Rheumatoid arthritis was self-reported with treatment ascertained using drug codes for methotrexate, hydroxychloroquine or corticosteroids. Corticosteroid use ascertained using drug codes for oral or IV glucocorticoids. History of parental hip fracture self-reported for those with 5-year data, or history of any parental osteoporotic fracture used from baseline questionnaire in those without 5-year data. BMD measured at the lumbar spine and femoral neck via DXA, and T-scores calculated using NHANES III reference data.<br><br>**Prediction:** the WHO Coordinating Centre used the Canadian FRAX tool calibrated using national hip fracture and mortality data along with the FRAX predictor variables from CaMos to calculate 10-year fracture probability. Unclear how many participants may have had missing data or how this was handled.	**MOF (hip, humerus, forearm/wrist, clinical spine), hip fractures:** self-reported on a yearly postal questionnaire and structured interview, with consent to contact the treating physician of hospital for verification.<br><br>**Competing risk:** Survival methods were used to control for incomplete follow-up (18.7%). 10-y estimates of observed fractures derived using Kaplan-Meier method with incomplete observations censored and death treated as a competing risk.	Observed and expected fracture probability, calibration plot, calibration slope<br><br>**Subgroups:** data available by sex and by quintile of baseline risk<br><br>Leslie 2011a provides data for the whole population (not stratified by sex) and by category of baseline risk (high, moderate, low)

Author & year, Country<br>Design<br>Funding source	Source of data and participant eligibility	Participant characteristics<br>Baseline predicted risk<br>Length of follow-up	Screening tool(s)<br>Included predictors & ascertainment<br>Risk prediction & handling of missing data	Outcomes predicted & ascertainment<br>Consideration of competing risk	Calibration outcomes & analyses
Goldshtein 2018 [20], Israel<br><br>Retrospective cohort<br><br>**Funding:** none<br><br>**Related studies:** none	**Maccabi Healthcare Services:** 141,320 female members of a government-funded health maintenance organization (MHS) aged 50-90 years in 2004 who had at least 3 years of prior membership. BMD is included in the membership package for those ≥60 years, those ≥50 years with prior fragility fracture, family history of osteoporosis, BMI <19, use of bisphosphonates or SERMS, or use of glucocorticoids ≥3 months.<br><br>**Exclusion:** missing data on height and weight (5%) required for the FRAX calculator	**Analyzed sample:** n = 141,320 (100% of eligible); 100% F; median (IQR) 58 (54-67) years; menopausal status NR; 19% were prescribed any anti-osteoporosis drugs before the index date, 20% were ever treated for >3 years (both pre- and post-index date)<br><br>**Predicted 10-y risk (mean):** FRAX-BMD 7.0% MOF, 1.8% hip; FRAX (no BMD) 6.9% MOF, 2.2% hip<br><br>**Follow-up:** 10 years	FRAX-Israel ± BMD<br><br>**Predictors:** electronic record data were used to collect variables at the last data point available on the index data, except for smoking and BMI, for which missing baseline data were replaced by the last available status up to the end of baseline data collection. Collected age, sex, BMI, previous fracture (defined as MOF), family history of hip fracture (used history of osteoporosis as a proxy), prolonged exposure to glucocorticoids (dispensations of medication), rheumatoid arthritis, secondary osteoporosis, and high alcohol consumption defined by diagnostic codes. BMD at the femoral neck extracted from data maintained by 7 medical centres and converted to T-scores using NHANES III reference standards.<br><br>**Prediction:** used downloadable paper charts from the FRAX website. For patients with missing data on smoking status (1.5%) the default value was used.	**MOF (femoral neck, clinical spine, forearm, proximal humerus), hip (femoral neck) fracture:** ascertained using clinical diagnosis and procedure codes; fracture that occurred 6 months follow-up a motor vehicle accident and all events including multiple fracture diagnosis codes with the same date were considered more likely to be trauma-related and excluded from analysis.<br><br>**Competing risk:** not considered; participants who died during follow-up were censored at death.	Expected and observed fracture probability, observed fractures, calibration plot, Hosmer-Lemeshow test<br><br>**Subgroups:** data available by age category (≥ and < 70 years), treatment status, presence of diabetes, and by decile of baseline risk
Gourlay 2017 [13], USA<br><br>Prospective cohort<br><br>**Funding:** government, academic<br><br>**Related studies:** none (see Ettinger 2013 [12] for FRAX outcomes)	**Osteoporotic Fracture in Men (MrOS) study:** 5994 community dwelling men ≥65 years recruited between March 2000 and April 2002 at 6 clinical centres in Birmingham, Alabama; Minneapolis, Minnesota; Palo Alto, California; the Monongahela Valley near Pittsburgh, Pennsylvania; Portland, Oregon; and San Diego, California<br><br>**Exclusion:** history of hip or clinical vertebral fracture, past or current antifracture treatment (bisphosphonate, calcitonin,	**Analyzed sample:** n = 4808-5200 (80.2-86.8% of total sample, depending on outcome); 0% F; mean (SD) 73.4 (5.8) years among men with BMD data; no use of anti-osteoporosis drugs at baseline (exclusion criteria), <1% during follow-up<br><br>**Baseline 10-y risk:** NR<br><br>**Follow-up:** mean 15.8 years	Garvan ± BMD<br>QFracture (no BMD)<br><br>**Predictors:** predictors used and method of ascertainment NR, assumed to be self-reported and included age, height, weight (BMI), race, previous fracture after age 50 years, smoking, alcohol use, history of parental hip fracture, rheumatoid arthritis, oral glucocorticoid use; BMD at femoral neck measured via DXA and T- and Z-scores calculated using NHANES III reference data.<br><br>**Prediction:** risk scores calculated using externally generated parameter estimates provided by the	**MOF (clinical spine, forearm, hip, shoulder), hip fractures:** Self-reported on a questionnaire every 4 months (>99% response) with confirmation by radiology reports or radiographic images.<br><br>**Competing risk:** not fully considered; only men with a MOF or hip fracture developing before a competing risk (anti-fracture treatment, death, incident osteoporosis) were considered.	Observed fractures, Hosmer-Lemeshow test, calibration plot (hip fractures only)<br><br>**Subgroups:** data available by decile of predicted risk for each tool (in calibration plot) for hip fractures only

Author & year, Country<br>Design<br>Funding source	Source of data and participant eligibility	Participant characteristics<br>Baseline predicted risk<br>Length of follow-up	Screening tool(s)<br>Included predictors & ascertainment<br>Risk prediction & handling of missing data	Outcomes predicted & ascertainment<br>Consideration of competing risk	Calibration outcomes & analyses
	teriparatide), osteoporosis by BMD criteria at baseline		respective algorithms. 54% missing data for rheumatoid arthritis, 29% for parental history of hip fracture, 21% for glucocorticoid use. Handling of missing data NR.<br><br>*data also available for FRAX-US, but this is not the main FRAX study for analysis in this cohort; see Ettinger 2013		
Holloway 2018 [21], Australia<br><br>Prospective cohort<br><br>**Funding:** government, foundation<br><br>**Related studies:** none	**Geelong Osteoporosis Study:** 769 men 40-90 years randomly selected from Commonwealth electoral rolls in the Barwon Statistical Division (captures almost all adults in the region), south-eastern Australia, between 2001 and 2006.<br><br>**Exclusion:** bone densitometry performed by Lunar DPX-L (does not allow calculation of trabecular bone score), missing femoral neck or lumbar spine BMD, missing one or more FRAX variable	**Analyzed sample:** n = 591 (76.9% of eligible); 0% F; 70 (60-79) years; 1.4% taking anti-osteoporosis drugs at baseline, NR during follow-up.<br><br>**Predicted 10-y risk (median (IQR)):** 3.7 (2.1-5.9)% MOF, 1.2 (0.3-2.4)% hip<br><br>**Follow-up:** median (IQR) 9.5 (7.5-11.4) years	FRAX-Aus + BMD<br><br>**Predictors:** at baseline, height and weight were measured. Participants self-reported age, sex, previous fractures, current smoking, alcohol consumption, oral glucocorticoid use, rheumatoid arthritis, secondary osteoporosis (insulin-treated diabetes, osteogenesis imperfect, untreated longstanding hyperthyroidism, malabsorption, chronic liver disease, chronic malnutrition [BMI <18.5 km/m^2]), use of anti-osteoporosis medication; BMD at the femoral neck and lumbar spine via DXA.<br><br>**Prediction:** data were entered into the FRAX online tool. Participants with missing FRAX data were excluded at baseline.	**MOF (clinical spine, hip, wrist, proximal humerus), hip fractures:** identified by examining radiological records from all imaging centres across the study region<br><br>**Competing risk:** not considered; endpoint considered to be first MOF, death, or end of study follow-up	Expected fracture probability, observed fractures<br><br>**Subgroups:** data available for high (≥20% MOF, 3% hip) vs. (<20% MOF, 3% hip) low baseline risk
Iki 2015 [22], Japan<br><br>Prospective cohort<br><br>**Funding:** government, academic<br><br>**Related studies:** none	**FORMEN cohort:** ancilliary study including a subset of 2012 men who completed the Fujiwara-kyo cohort study, which enrolled 4427 men ≥65 years in 2007 from four cities in Nara Prefecture. Men were living at home, able to walk without assistance from another person, and able to provide self-reported information and provide consent.	**Analyzed sample:** n = 1805 (89.7% of enrolled); 0% F; mean (SD) 73.0 (5.1) years; anti-osteoporosis drugs at baseline NR, 17 (0.9%) during follow-up (bisphosphonates for ≥6 months or activated vitamin D or other drugs for ≥2 years)<br><br>**Predicted 10-y risk (mean (SD)):** 5.9 (1.4)% MOF<br><br>**Follow-up:** median 4.5 years	FRAX-Japan (3.8) + BMD<br><br>**Predictors:** at baseline, in-person interviews using a structured questionnaire were used to collect age, history of disease (rheumatoid arthritis, conditions associated with osteoporosis) and medications related to disease (e.g., glucocorticoids), smoking, drinking, diet, prior fragility fracture, maternal hip fracture at ≥50 years (substituted for parental history). Height and weight were measured; BMD at the spine, hip, and femoral neck were measured via	**10-y MOF (femoral neck, spine, distal forearm, or proximal humerus):** Self-reported in follow-up interviews with trained nurses, or in telephone or mail surveys; only included fractures that occurred without a strong external force<br><br>**Competing risk:** not considered, appears that those who died during follow-up or were lost for	Expected fracture probability, observed and expected fractures<br><br>**Subgroups:** data available by tertile of baseline risk

Author & year, Country<br>Design<br>Funding source	Source of data and participant eligibility	Participant characteristics<br>Baseline predicted risk<br>Length of follow-up	Screening tool(s)<br>Included predictors & ascertainment<br>Risk prediction & handling of missing data	Outcomes predicted & ascertainment<br>Consideration of competing risk	Calibration outcomes & analyses
	**Exclusion:** missing information required for FRAX calculation		DXA and T-scores calculated according to Japanese reference data.<br><br>**Prediction:** appear to have used online calculator. Participants with missing data were excluded.	other reasons (10.3%), were excluded.	
Langsetmo 2011 [23], Canada<br><br>Prospective cohort<br><br>**Funding:** government, foundation, industry<br><br>**Related studies:** none	**Canadian Multicentre Osteoporosis Study:** 9424 people living within a 50-km radius of nine Canadian cities and aged 55-95 years at study entry randomly selected from a list of residential phone numbers.<br><br>**Exclusion:** missing data, <1 year of follow-up data	**Analyzed sample:** n = 5758 (61.1% of eligible); 72.1% F; mean (SD) 67.7 (7.6) years; menopausal status NR; 21.5% used anti-osteoporosis drugs at baseline, NR during follow-up<br><br>**Predicted 10-y risk (mean (SD)) in women:** 18.33 (14.04)% low trauma, 5.63 (10.31)% hip<br><br>**Predicted 10-y risk (mean (SD)) in men:** 11.75 (12.74)% low trauma, 2.66 (6.16)% hip<br><br>**Follow-up:** mean 8.5 years	Garvan (with BMD)<br><br>**Predictors:** at baseline participants completed a questionnaire to self-report age, presence of prior fractures after age 50 years, falls in the past year (falls in past month used as a proxy)<br><br>**Prediction:** NR, used the Dubbo nomogram previously derived via model selection. Unclear how many participants had missing data or how this was handled.	**Low trauma fractures, hip fractures:** self-reported on yearly follow-up questionnaires or in-person. Included fractures without trauma or caused by a fall from standing height or less, excluding skull, face, hands, ankles, feet.<br><br>**Competing risk:** not considered; participants were censored at death or loss to follow-up, and Kaplan-Meier methods used to account for varying lengths of follow-up, but consideration of death as a competing hazard NR	Observed and expected fracture probability, observed fractures, calibration plot<br><br>**Subgroups:** data available by sex and by quintile of baseline risk
Leslie 2016 [24], Canada<br><br>Retrospective cohort<br><br>**Funding:** none<br><br>**Related studies:** Leslie 2009 [25], Leslie 2010b [26]	**Manitoba Bone Density Program:** 34,060 women and men ≥50 years at baseline with BMD recorded in the Manitoba Bone Mineral Density Database (which records all BMD testing conducted in the province of Manitoba) from January 1, 1996 onward. Criteria for screening were women ≥65 years without risk factors, and men or women <65 years with risk factors.<br><br>**Exclusion:** BMD measured prior to January 1, 1996, receiving anti-osteoporosis therapy, <5 years of observation time	**Analyzed sample:** n = 34,060 (NR% of eligible); 91% F; mean (SD) 66.6 (9.8) years; menopausal status NR; no use of anti-osteoporosis drugs at baseline (exclusion criteria); NR during follow-up<br><br>**Predicted 10-y risk (mean (SD)):** 44.3% low risk (<10%), 37.9% moderate risk (10-20%), 17.8% high risk (>20%)<br><br>**Follow-up:** mean 9.8 years	CAROC<br><br>**Predictors:** age, sex, femoral neck BMD, prior fragility fracture, and systemic glucocorticoid use (3-month cumulative therapy in past year at a prednisone-equivalent dose of ≥7.5 mg/day) assessed through a combination of hospital discharge abstracts, diagnoses, and procedures (ICD-9-CM or ICD-10-CA codes), physician billing claims (ICD-9-CM) and information collected directly from participants at the time of DXA scanning. BMD of lumbar spine and femoral assessed by DXA and total hip T-scores calculated from NHANES III white female reference values.	**MOF (hip, clinical vertebral, forearm, humerus):** health records assess for the presence of incidence of relevant non-traumatic fracture codes. Hip and forearm fractures need to be associated with site-specific fracture reduction, fixation, or casting codes.<br><br>**Competing risk:** For each category of risk, the observed incidence of mortality was compared, adjusting for the competing risk of mortality	Expected and observed fracture probability, observed fractures<br><br>**Subgroups:** data available by category (low, moderate, high) of baseline risk.<br><br>Leslie 2009 and 2010b provide data for similar overlapping cohorts, but with shorter follow-up. The Leslie 2010b cohort includes those on anti-osteoporosis treatment.

Author & year, Country<br>Design<br>Funding source	Source of data and participant eligibility	Participant characteristics<br>Baseline predicted risk<br>Length of follow-up	Screening tool(s)<br>Included predictors & ascertainment<br>Risk prediction & handling of missing data	Outcomes predicted & ascertainment<br>Consideration of competing risk	Calibration outcomes & analyses
			**Prediction:** calculation NR; unclear how many participants had missing data and how this was handled<br><br>*data also available for FRAX-Canada, but this is not the main FRAX study for analysis in this cohort		
Leslie 2017b [27], Canada<br><br>Retrospective cohort<br><br>**Funding:** NR<br><br>**Related studies:** Leslie 2010a [28], Leslie 2011b [29], Leslie 2012a [3], Leslie 2012b [31], Leslie 2013 [32], Leslie 2014 [33], Brennan 2014 [34], Majumdar 2016 [35], Martineau 2017 [36], Leslie 2017a [37], Leslie 2018 [38], Bolton 2017 [39], Lix 2018 [40], Yang 2019 [41], Crandall 2019a [42]	**Manitoba Bone Density Program:** 62,275 women and 6,455 men ≥50 years at baseline with BMD recorded in the Manitoba Bone Mineral Density Database (which records all BMD testing conducted in the province of Manitoba) from January 1, 1996 to 2013. Criteria for screening were women ≥65 years without risk factors, and men or women <65 years with risk factors.<br><br>**Exclusion:** incomplete FRAX data	**Analyzed sample:** 68,730 (100% of eligible with complete data); 90.6% F; mean(SD) 64.1 (11.1) years for females and 66.0 (12.2) years for males; menopausal status NR; use of anti-osteoporosis drugs NR<br><br>**Predicted 5-y risk:** NR (but outcome data available)<br><br>**Predicted 10-y risk in women (mean (SD)):** FRAX+BMD 10.9 (8.0)% MOF, 2.6 (4.5)% hip; FRAX (no BMD) 11.8 (9.0)% MOF, 3.4 (5.3)% hip<br><br>**Predicted 10-y risk in men (mean (SD)):** FRAX+BMD 8.2 (5.2)% MOF, 3.6 (3.6)% hip; FRAX (no BMD) 11.8 8.0 (5.0)% MOF, 2.8 (3.8)% hip<br><br>**Follow-up:** mean 7.1 (4.2) years. Estimated fracture probabilities at 5- and 10-years using simple linear rescaling	FRAX-Canada (3.7) ± BMD<br><br>**Predictors:** height and weight were self-reported pre-2000, measured thereafter (BMI); linkage to hospital discharge abstracts and billing claims used to assess prior fracture (non-traumatic), prolonged oral corticosteroid used (>90 days dispensed in the past year), parental hip fractures (self-report from 2005-onward and by linkage to hospitalization records in earlier years), current smoking (self-report from 2005 onwards and using chronic obstructive pulmonary disease codes in earlier years), alcohol use (self-reported from 2012 onwards and using alcohol substance abuse codes in earlier years), secondary osteoporosis via records (hyperthyroidism, chronic malnutrition, chronic liver disease, inflammatory bowel disease, Parkinson's disease, cerebrovascular disease, multiple sclerosis, ankylosing spondylitis, organ transplant)<br><br>**Prediction:** used online FRAX calculator; included participants with complete data	**MOF (humerus, hip, clinical vertebral, forearm), hip fractures:** extracted relevant ICD-9-CM or ICD-10-CA codes and physician billing claims for fractures not associated with codes indicative of severe trauma. For hip and forearm fractures, site-specific fracture reduction, fixation, or casting code was required.<br><br>**Competing mortality:** the cumulative incidence function for MOF and hip fracture was constructed following a competing mortality framework	Observed and expected fracture probability, observed fractures, O:E<br><br>**Subgroups:** data available by sex<br><br>Crandall 2019a is the main study for analysis in women (also provides data in 10-year age groups from 40-80+ years). Leslie 2010a provides calibration plots. Majumdar 2016 provides data for those with and without diabetes. Remaining studies offer limited additional information.
Li 2015 [43], Canada<br><br>Prospective cohort<br><br>**Funding:** academic, industry	**GLOW cohort:** 4000 Canadian (Hamilton, Ontario) women ≥55 years enrolled between May 2008 and March 2009 from an international cohort bringing together data from 17 sites in 10 countries. Participants were stratified such that	**Analyzed sample:** n = 3985 (99.6% of eligible); 100% F; mean (SD) 69.4 (8.9) years; menopausal status NR; use of anti-osteoporosis drugs NR<br><br>**Baseline 10-y risk:** mean (SD) 16 (9.9)% MOF	FRAX-Canada without BMD<br><br>**Predictors:** at baseline, a mailed questionnaire or telephone interview was used to collect data on age, sex, weight, height, history of fragility fracture, parental hip fracture, smoking, alcohol	**MOF (spine, upper arm or shoulder, wrist, hip):** self-reported on a mailed annual questionnaire or telephone interview in the case of non-response	Expected fracture probability, observed fractures<br><br>**Subgroups:** data available by category of predicted risk (low, moderate, high)

Author & year, Country<br>Design<br>Funding source	Source of data and participant eligibility	Participant characteristics<br>Baseline predicted risk<br>Length of follow-up	Screening tool(s)<br>Included predictors & ascertainment<br>Risk prediction & handling of missing data	Outcomes predicted & ascertainment<br>Consideration of competing risk	Calibration outcomes & analyses
**Related studies:** none	approximately two-thirds were ≥65 years<br><br>**Exclusion:** cognitive impairment, language barrier, institutionalized, too ill to complete the survey	**Follow-up:** mean 3.01 years	intake, oral glucocorticoids, rheumatoid arthritis, secondary osteoporosis<br><br>**Prediction:** NR, likely used online algorithm. Used the multiple imputation approach to impute missing data if the percentage was more than 10%. When less than 10% of data on a variable were missing, the median or mean of that variable was used for imputation.	**Competing risk:** not considered. Appears that participant observations were censored at death.	
Lo 2011 [44], USA<br><br>Retrospective cohort<br><br>**Funding:** academic<br><br>**Related studies:** none<br><br>*same population as Pressman 2011 [45	**Kaiser Permanente Northern California:** 116,962 women 50-85 years who underwent a hip BMD scan during 1997-2003 who were members of a large integrated healthcare delivery system in Northern California serving >3 million members<br><br>**Exclusion:** <1 year of continuous (<90-day gap) membership prior and following the DXA scan, DXA not electronically accessible, missing race/ethnicity, women who had filled a bisphosphonate prescription in the year prior to DXA; excluded during follow-up after the 4th bisphosphonate prescription	**Analyzed sample:** n = 94,489 (80.8% of available cohort; 100% F; 41.4% 50-59 years, 34.8% 60-69 years, 20.2% 70-79 years, 3.6% 80+ years; menopausal status NR; 42% taking HRT at baseline (other anti-osteoporosis medications NR and bisphosphonates excluded), NR during follow-up<br><br>**Baseline 10-y risk:** NR – participants categorized and predicted risk compared to observed risk<br><br>**Follow-up:** mean (IQR) 6.6 (3.6-8.3) years	Fracture Risk Calculator (FRC; with BMD)<br><br>**Predictors:** age, race/ethnicity, and body mass index (BMI) were determined at the index BMD scan date. Used ambulatory care, hospitalization, and pharmacy databases to obtain glucocorticoid use (≥1825 mg of cumulative prednisone dose equivalent in the prior year), rheumatoid arthritis, and secondary causes of bone loss (diabetes mellitus with insulin use, malabsorption syndrome, chronic liver disease, osteogenesis imperfecta). Prior history of fracture after age 45 years based on hospitalization and outpatient diagnoses of fracture (ICD codes). Femoral neck BMD measured via DXA and calculated Z-scores using NHANES III reference ranges.<br><br>**Prediction:** Entered data into the Foundation for Osteoporosis Research and Education FRC website. Information on alcohol consumption and parental history of hip fracture unavailable and smoking status not uniformly available. Missing input values assumed to be null. Those with missing BMI were assigned the median value in the cohort.	**Hip fracture:** extracted from patient records using relevant ICD-9 codes, excluding open fractures and those associated with major trauma.<br><br>**Competing risk:** not considered; used Kaplan-Meier product-limit estimates to calculate observed fracture probability with participants censored at death, loss to follow-up, or 4th bisphosphonate prescription	Observed fractures, O:E ratio, calibration plot<br><br>**Subgroups:** data available by category of baseline risk (low, moderate, high)
Marques 2017 [46], Portugal	Combined 3 population-based cohorts (n = 5049):	**Analyzed sample:** n = 2626 (52.0% of eligible); 73% F; mean (SD) 58.2 (10.2) years; menopausal status NR; use of	FRAX-Portugal ± BMD	**MOF (hip, wrist, shoulder, clinical spine), hip fracture:** self-reported, with confirmation by	Expected fracture probability, observed and expected fractures,

Author & year, Country<br>Design<br>Funding source	Source of data and participant eligibility	Participant characteristics<br>Baseline predicted risk<br>Length of follow-up	Screening tool(s)<br>Included predictors & ascertainment<br>Risk prediction & handling of missing data	Outcomes predicted & ascertainment<br>Consideration of competing risk	Calibration outcomes & analyses
Prospective cohort<br><br>**Funding:** academic, industry<br><br>**Related studies:** none	**SAOL cohort:** 1745 people >18 years were randomly selected from the Santo António dos Olivais country electoral register between March 1998 and April 2000<br>**IPR cohort:** 819 people ≥40 years who were referred for a DXA scan performed between December 1999 and July 2001 at Instituto Português de Reumatologia, Lisbon.<br>**EPIPorto cohort:** 2485 people >18 years randomly selected from 1999 to 2003. FRAX was completed at the second follow-up (2005-2006) and considered to be baseline.<br><br>**Exclusion:** incomplete FRAX data	anti-osteoporosis drugs at baseline NR; 7.6% during follow-up<br><br>**Predicted 10-y risk (median (IQR)):** FRAX-BMD 3.4 (1.8-6.9)% MOF, 0.7 (0.2-2.5)% hip; FRAX (no BMD) 2.9 (1.7-5.8)% MOF, 0.5 (0.2-1.6)% hip<br><br>**Follow-up:** mean (SD) 9.12 (1.5) years	**Predictors:** at baseline, participants completed questionnaires including age, BMI, previous fracture, parental hip fracture, current smoking, oral glucocorticoids, rheumatoid arthritis, secondary osteoporosis, alcohol use; BMD was measured via DXA at the femoral neck and lumbar spine, with hip T-scores calculated according to NHANES III reference data. All variables were defined exactly as prescribed by FRAX.<br><br>**Prediction:** appear to have used the online calculator. Participants with missing FRAX data were excluded at baseline.	clinical file review in the SAOL cohort only. For those who died during follow-up, fracture data were collected from family members.<br><br>**Competing risk:** not considered. Participants who died during follow-up were included in the analysis.	 **Subgroups:** data available by age category (<60, 60-75, >75 years) and sex
Melton 2012 [47], USA<br><br>Prospective cohort<br><br>**Funding:** academic<br><br>**Related studies:** none	**Rochester Epidemiology Project:** 503 women and men recruited from an age-stratified sample of Rochester, Minnesota women ≥40 years at baseline that was selected using the medical records linkage system of the Rochester Epidemiology Project for patients seen in 1980 ± 1 year (almost all of population is seen within a 3-y period).<br><br>**Exclusion:** NR	**Analyzed sample:** n = 499 (99.2% of eligible); 50% F; mean age NR, range 40-93 years; menopausal status NR; treatment with anti-osteoporosis drugs NR<br><br>**Baseline 10-y risk (median (range):** 7 (0-45)% MOF; hip fracture NR<br><br>**Follow-up:** 74% followed for at least 10 years	FRAX-US (3.1) + BMD<br><br>**Predictors:** participants were interviewed to collect personal history of fracture after 35 years, rheumatoid arthritis, oral glucocorticoid use, current smoking, heavy alcohol use (>2 drinks/day), parental history of hip fracture. Community medical records were used to confirm prior fractures and collect information on conditions predisposing to falls or secondary osteoporosis. Femoral neck BMD was measured via DXA and T-score calculated from national reference data for women.<br><br>**Prediction:** calculated by the World Health Organization Collaborating Centre for Metabolic Bone Diseases using FRAX 3.1 models.	**MOF (hip, clinical spine, distal forearm, proximal humerus), hip fractures:** self-reported in periodic interviews and confirmed with medical record review. Original x-rays were not available for review so diagnosis of vertebral fracture was accepted based on a radiologist's report. Categorized incidentally noted vertebral fractures separately from those reported as symptomatic.<br><br>**Competing risk:** Computations for observed probability based on the method of Berry, which accounts for both incomplete follow-up and the competing risk of death (O:E ratio)	Expected fracture probability, expected and observed fractures, O:E ratio<br><br>**Subgroups:** data available by sex and by quartile of predicted risk

Author & year, Country<br>Design<br>Funding source	Source of data and participant eligibility	Participant characteristics<br>Baseline predicted risk<br>Length of follow-up	Screening tool(s)<br>Included predictors & ascertainment<br>Risk prediction & handling of missing data	Outcomes predicted & ascertainment<br>Consideration of competing risk	Calibration outcomes & analyses
Pluskiewicz 2015 [48], Poland<br><br>Prospective cohort<br><br>**Funding:** NR<br><br>**Related studies:** none	**RAC-OST-POL study cohort:** 625 postmenopausal women randomly selected from the Racibórz district, and 353 women invited by post in May 2010 for an epidemiological study on osteoporosis.<br><br>**Exclusion:** changed address or phone number during follow-up, refused to cooperate, died	**Analyzed sample:** n = 770 (78.7% of eligible); 100% F; mean (SD) 65.6 (7.3) years; all postmenopausal; use of anti-osteoporosis drugs NR<br><br>**Predicted 10-y risk (mean (SD)):** FRAX+BMD 5.7 (3.8)% non-traumatic fractures, 1.4 (2.3)% hip fractures; FRAX (no BMD) 7.0 (5.1)% nontraumatic fractures, 2.0 (2.4)% hip fractures; Garvan+BMD 17.6 (12.6)% nontraumatic fractures, 5.0 (8.7)% hip fractures<br><br>**Follow-up:** 4 years	FRAX-Poland (3.9) ± BMD<br>Garvan + BMD<br><br>**Predictors:** ascertainment unclear, assumed to be self-reported. At baseline, collected information on prior fracture, hip fracture in parents, smoking, rheumatoid arthritis, steroid or anticonvulsant use, alcohol intake, diabetes, thyroid disease, early menopause (before 45 years) malabsorption, renal or liver failure. The authors do not report how these were used in the tools.<br><br>**Prediction:** appear to have used online nomograms; unclear how many participants had missing data nor how missing data were handled	**All fractures of nontraumatic origin, hip fractures of nontraumatic origin:** self-reported at yearly follow-up and confirmed by a doctor.<br><br>**Competing risk:** not considered; participants who died during follow-up (3.1%) or were lost for other reasons were excluded	Expected fracture probability, observed fractures<br><br>**Subgroups:** data available by baseline high (>10%) and low (≤10%) FRAX probability
Premaor 2013 [49], USA<br><br>Prospective cohort<br><br>**Funding:** government<br><br>**Related studies:** Hillier 2011 [50], Kalvesten 2016 [51]	**Study of Osteoporotic Fractures (SOF):** 8098 community-based ambulatory women recruited between September 1986 and October 1988 from population-based listings at four clinical centres in Portland, Oregon; Minneapolis, Minnesota; Baltimore, Maryland; and Monongahela Valley near Pittsburgh, Pennsylvania. This analysis included women who attended the 2-year follow-up visit.<br><br>**Exclusion:** women unable to walk without assistance, with bilateral hip replacements, black women, missing FRAX variables	**Analyzed sample:** n = 6049 (74.7% of eligible); 100% F; mean (SD) 72.2 (5.3) years; menopausal status NR; use of anti-osteoporosis drugs NR<br><br>**Baseline 10-y risk:** FRAX+BMD 18.2% MOF, 7.1% hip in obese, 23.3% MOF, 10.9% hip in non-obese; FRAX (no BMD) 17.6% MOF, 5.8% hip in obese, 23.6% MOF, 11.4% hip in non-obese<br><br>**Follow-up:** mean (SD) 9.03 (2.22) years	FRAX-US (3.0) ± BMD<br><br>**Predictors:** at second (baseline) visit, participants completed a self-administered questionnaire including age, smoking habits, alcohol, family history of fractures, personal history of fractures after 50 years, medical conditions such as diabetes mellitus, rheumatoid arthritis, glucocorticoid use; weight and height (BMI) were measured; BMD of proximal femur (total hip and subregions) measured via DXA.<br><br>**Prediction:** used the FRAX algorithm for Caucasian women. Excluded any participants with missing FRAX data.	**MOF (hip, clinical spine, wrist, humerus), hip fractures:** self-reported on a questionnaire every 4 months (98% response) with confirmation by radiology reports. Pathological fractures (including periprosthetic) and fractures secondary to extreme trauma were excluded.<br><br>**Competing risk:** not considered. Appears that participant observations were censored at death.	Expected fracture probability, observed and expected fractures, Hosmer-Lemeshow test<br><br>**Subgroups:** data available by quartile of predicted risk, category of risk (low vs. high < and ≥3% or 20%)<br><br>Kalvesten 2016 provides data by decile of risk in 5-year age categories. Hillier 2011 does not provide additional data of interest.
Pressman 2011 [45], USA<br><br>Retrospective cohort<br><br>**Funding:** academic	**Kaiser Permanente Northern California:** 116,962 women 50-85 years who underwent a hip BMD scan during 1997-2003 who were members of a large integrated	**Analyzed sample:** n = 94,489 (80.8% of available cohort; 100% F; 41.4% 50-59 years, 34.8% 60-69 years, 20.2% 70-79 years, 3.6% 80+ years; menopausal status NR; 42% taking HRT at baseline	FRAX-US (3.0) ± BMD<br><br>**Predictors:** age, race/ethnicity, and body mass index (BMI) were determined at the index BMD scan date. Used health plan administrative	**Hip fracture:** extracted from patient records using relevant ICD-9 codes, excluding open fractures and those associated with major trauma.	Expected and observed fracture probability, observed fractures, O:E ratio

Author & year, Country<br>Design<br>Funding source	Source of data and participant eligibility	Participant characteristics<br>Baseline predicted risk<br>Length of follow-up	Screening tool(s)<br>Included predictors & ascertainment<br>Risk prediction & handling of missing data	Outcomes predicted & ascertainment<br>Consideration of competing risk	Calibration outcomes & analyses
**Related studies:** none<br><br>*same population as Lo 2011 [44]	healthcare delivery system in Northern California serving >3 million members<br><br>**Exclusion:** <1 year of continuous (<90-day gap) membership prior and following the DXA scan, DXA not electronically accessible, missing race/ethnicity, women who had filled a bisphosphonate prescription in the year prior to DXA; excluded during follow-up after the 4th bisphosphonate prescription	(other anti-osteoporosis medications NR and bisphosphonates excluded), NR during follow-up<br><br>**Baseline 10-y risk:** FRAX-BMD 0.25% for 50-59y, 0.68% for 60-69y, 2.80% for 70-79y, 4.90% for 80-85y hip; FRAX (without BMD) 0.34% for 50-59y, 1.11% for 60-69y, 4.03% for 70-79y, 9.21% for 80-85y hip<br><br>**Follow-up:** mean 6.6 years	databases to obtain data for current smoking, use of glucocorticoids (>1825 mg prednisone equivalents in prior year), rheumatoid arthritis, secondary causes of bone loss (diabetes mellitus with insulin use, malabsorption syndrome, chronic liver disease), prior fracture after age 45 years. Femoral neck BMD measured via DXA and calculated Z-scores using NHANES III reference ranges.<br><br>**Prediction:** Risk estimates obtained from the WHO Collaborating Centre for Metabolic Bone Diseases via the International Osteoporosis Foundation website. Information on alcohol consumption and parental history of hip fracture unavailable and smoking status not uniformly available. Missing input values assumed to be null. Those with missing BMI were assigned the median value in the cohort.	**Competing risk:** not considered; used Kaplan-Meier product-limit estimates to calculate observed fracture probability with participants censored at death, loss to follow-up, or 4th bisphosphonate prescription	**Subgroups:** data available by age category (60-69y, 70-79y, 80+y) and by category of baseline risk (low, moderate, high)
Reyes Domínguez 2017 [52], Spain<br><br>Prospective cohort<br><br>**Funding:** foundation<br><br>**Related studies:** none	400 people from the Canary Islands who attended for densitometry and had no osteoporotic values.<br><br>**Exclusion:** did not attend any follow-up visits, started anti-osteoporosis treatment during follow-up	**Analyzed sample:** n = 121 (30.3% of eligible); 90.5% F (in eligible sample); mean (SD) 59.3 (6.8) years; menopausal status NR; no use of anti-osteoporosis drugs (exclusion criteria)<br><br>**Predicted 10-y risk:** median (IQR) 15 (10;28)% MOF; 3 (1;8)% hip<br><br>**Follow-up:** 10 years	Garvan + BMD<br><br>**Predictors:** age, sex, presence of fragility fractures beyond 50 years, falls in the past 12 months appear to be self-reported (NR); BMD measured via densitometry, site NR<br><br>**Prediction:** used the online Garvan calculator; participants with less than complete follow-up were excluded at baseline	**10-y fragility fractures (not defined), hip fractures:** self-reported during follow-up<br><br>**Competing risk:** not considered; participants who died during follow-up were excluded.	Expected fracture probability, observed fractures<br><br>**Subgroups:** none
Sornay-Rendu 2010 [53], France<br><br>Prospective cohort<br><br>**Funding:** industry<br><br>**Related studies:** none	**OFELY cohort:** 867 randomly selected volunteer women from a large health insurance registry from the Rhône district (Lyon and its surroundings) recruited between February 1992 and December 1993<br><br>**Exclusion:** <40 years at inclusion in the cohort	**Analyzed sample:** 867 (100% of enrolled); 100% F; mean (SD) 58.8 (10.3) years; 680 (78.4%) postmenopausal; 127 (14.6%) took HRT for ≥5 years (including during baseline), none took bisphosphonates<br><br>**Predicted 10-y risk (mean (SD)):** FRAX+BMD 5.9 (6.3)% MOF, 1.8 (4.3)%	FRAX ± BMD<br><br>**Predictors:** at baseline, a questionnaire was used to collect parental history of hip fracture, prior fragility fracture (low trauma fractures of wrist, humerus, vertebrae, hip after 40 years), current tobacco smoking, daily consumption of alcohol of more than 2 units, ever long-term use of oral glucocorticoids, rheumatoid arthritis, and other	**10-y MOF (clinical vertebral, hip, shoulder, forearm), hip fractures:** self-reported at each annual follow-up or by mail if did not attend. All fractures confirmed with radiographs or surgical report. Only included low-trauma fractures and	Expected fracture probability, observed fractures<br><br>**Subgroups:** data available by quartile of baseline risk, by 5-year age group between 40 and 89 years,

Author & year, Country<br>Design<br>Funding source	Source of data and participant eligibility	Participant characteristics<br>Baseline predicted risk<br>Length of follow-up	Screening tool(s)<br>Included predictors & ascertainment<br>Risk prediction & handling of missing data	Outcomes predicted & ascertainment<br>Consideration of competing risk	Calibration outcomes & analyses
		hip; FRAX (no BMD) 6.6 (7.3)% MOF, 2.4 (5.1)% hip<br><br>**Follow-up:** 10 years	secondary causes of osteoporosis. Height and weight were measured; BMD measured at the femoral neck via DXA and T-score calculated using NHANES III reference values.<br><br>**Prediction:** used the FRAX tool; unclear how many participants had missing data nor how missing data were handled	symptomatic vertebral fractures that came to clinical attention.<br><br>**Competing risk:** not considered for cohort-level data, participants appear to be censored at death. Authors state that they 'corrected for mortality' by providing data by 1000 P-Y for data by quartile of risk.	and for those untreated at baseline
Tamaki 2019 [54], Japan<br><br>**Funding:** government, industry<br><br>**Related studies:** Tamaki 2011 [55]	4550 women were randomly selected in 5-year age groups (15-79 years) using resident registrations from seven municipalities. Of these, 3985 women (87.6%) completed the baseline survey in 1996. The participants from five municipalities were selected for the cohort study.<br><br>**Exclusion:** use of anti-osteoporosis drugs at baseline, death, missing data	**Analyzed sample:** n = 1541 (33.9% of eligible); 100% F; mean (SD) 58.1 (10.6) years; menopausal status NR; 127 (8.0%) used anti-osteoporosis drugs during follow-up<br><br>**Predicted 10-y risk (mean (SD)):** FRAX+BMD 6.9 (6.2%) MOF; FRAX (no BMD) 7.1 (6.6)%<br><br>**Follow-up:** median 10 years (10 years in Myakojima, 15-16 years in the other municipalities)	FRAX-Japan (3.8) ± BMD<br><br>**Predictors:** at baseline, trained public health nurses collected self-reported age, history of fractures, disease history, prescribed medications, smoking and drinking habits (daily alcohol consumption substituted for >3 units per day), mother's history of fractures after age 50y (substituted for parental history). Height and weight were measured. BMD was measured at the lumbar spine using DXA.<br><br>**Prediction:** Used the online FRAX-Japan tool. Participants with missing data or who changed address were excluded.	**10-y MOF (clinical fracture of hip, vertebra, disal forearm, proximal humerus):** self-reported in interviews with public health nurses or on mailed surveys during follow-up; only included fractures that occurred without a strong external force<br><br>**Competing risk:** not considered; participants who died during follow-up were excluded.	Expected fracture probability, observed fractures<br><br>**Subgroups:** none.<br><br>Tamaki 2011 provides data for hip fractures and by quartile of expected risk (MOF and hip fracture).
Tanaka 2010 [56], Japan<br><br>Prospective cohort<br><br>**Funding:** NR<br><br>**Related studies:** none	Of 1453 inhabitants aged 40-79 years in Miyama village listed in the resident registration in December 1988, 200 women were recruited. This cohort was combined with 200 women recruited from a list of 2261 inhabitants aged 40-79 years in Taiji Town in June 1992.<br><br>**Exclusion:** NR	**Analyzed sample:** n = 400 (100% of selected from cohorts); 100% F; mean (SD) 59.5 (11.3) years; menopausal status NR; proportion using anti-osteoporosis drugs NR<br><br>**Predicted 10-y risk:** FRAX: 9.5% MOF; FRISC 20.3% MOF<br><br>**Follow-up:** 10 years	FRAX-Japan + BMD<br>FRISC + BMD (developed within a separate cohort in the same study)<br><br>**Predictors:** self-reported on a self-administered questionnaire in the Miyama cohort, and a mix of self-reported questionnaire and interview-administered questionnaire in the Taiji cohort.<br>***FRAX:*** age, sex, weight, height, previous fracture, parental history of hip fracture (Taiji cohort only), current smoking status, glucocorticoid use, rheumatoid arthritis, alcohol intake and femoral	**10-y MOF (hip, surgical neck of humerus, distal forearm, clinical vertebral):** ascertainment NR, other than radiographs were used to ascertain morphometric vertebral fractures in the Miyama cohort during follow-up.<br><br>**Competing risk:** not considered. Participants were censored at death.	Expected and observed fractures, O:E ratio, Hosmer-Lemeshow test<br><br>**Subgroups:** none

Author & year, Country<br>Design<br>Funding source	Source of data and participant eligibility	Participant characteristics<br>Baseline predicted risk<br>Length of follow-up	Screening tool(s)<br>Included predictors & ascertainment<br>Risk prediction & handling of missing data	Outcomes predicted & ascertainment<br>Consideration of competing risk	Calibration outcomes & analyses
			neck BMD; ***FRISC:*** age, weight, lumbar BMD, prior fracture, presence of back pain.<br><br>**Prediction:** entered into the online FRAX tool or used self-developed FRISC algorithm. For the Miyama cohort, it was assumed that participants had no parental history of hip fracture. Unclear how many other participants had missing data or how these were handled. Participants who moved or were lost to follow-up were treated as censored.		
Tebé Cordomí 2013 [57], Spain<br><br>Retrospective cohort<br><br>**Funding:** government<br><br>**Related studies:** none	**CETIR cohort:** random sample of 2086 women aged 40-90 years with a first visit for bone densitometry at the CETIR Medical Centre in Barcelona at the request of a general practitioner or specialist between January 1992 and February 2008.<br><br>**Exclusion:** did not have at least one follow-up survey or earlier report of MOF, or did not consent to the study	**Analyzed sample:** n = 1231 (59.0% of eligible); 100% F; mean (SD) 56.8 (7.8) years; menopausal status NR; 436 (35.4%) used anti-osteoporosis drugs during follow-up (78% bisphosphonates)<br><br>**Predicted 10-y risk:** 4.6% MOF<br><br>**Follow-up:** median (IQR) 10.95 (0.52) years	FRAX-Spain + BMD<br><br>**Predictors:** at baseline visit (or by telephone), trained technicians collected self-reported age, sex, BMI, personal and family history of MOF, history of other comorbidities (likely to affect bone density: rheumatoid arthritis, hyperparathyroidism, diabetes mellitus, anorexia nervosa, hyperthyroidism, secondary osteoporosis), use of drugs with potential effects on BMD (glucocorticoids, anticonvulsants, diuretics), smoking status, alcohol intake in units per day<br><br>**Prediction:** used FRAX-Spain; unclear how many participants had missing data nor how missing data were handled	**10-y MOF (forearm, proximal humerus, clinical spine, hip):** self-reported and confirmed by imaging studies for some but not all participants. Included only fractures resulting from low-intensity trauma.<br><br>**Competing risk:** not considered; participants who died during follow-up were excluded.	Expected and observed fractures; O:E ratio<br><br>**Subgroups:** data available by decile of predicted risk; age category (40-55, 55-65, 65-75, ≥75 years)
Trémollieres 2010 [58], France<br><br>Prospective cohort<br><br>**Funding:** industry<br><br>**Related studies:** none	**MENOS cohort:** 4024 women >45 years who were consecutively referred to the Menopause Centre at Toulouse University Hospital between 1988 and 1991 for a systematic 'menopause checkup'.<br><br>**Exclusion:** past or current use (any time during follow-up) of anti-osteoporosis drugs for >3 months	**Analyzed sample:** 956 (41.0% of eligible); 100% F; mean (SD) 53.5 (4.2) years; menopausal status NR; no use of anti-osteoporosis drugs (including HRT; exclusion criteria)<br><br>**Predicted 10-y risk (mean (SD)):** 3.8 (2.4)%<br><br>**Follow-up:** mean (SD) 13.4 (1.4) years	FRAX + BMD<br><br>**Predictors:** at baseline, participants a computer-assisted standardized questionnaire was completed and a trained research nurse extracted Age, weight, height, BMI, reproductive history, self-reported history of low-trauma fractures after age 45, parental history of hip fracture, history of medical conditions and use of medications known to impair bone mass,	**MOF (clinical spine, hip, distal forearm, proximal humerus):** self-reported at follow-up and confirmed using radiographs or medical/surgical reports. Systematic radiographs of the spine were not performed and only minimal or no trauma fractures and symptomatic spine fractures were considered.	Expected fracture probability, observed and expected fractures<br><br>**Subgroups:** none

Author & year, Country<br>Design<br>Funding source	Source of data and participant eligibility	Participant characteristics<br>Baseline predicted risk<br>Length of follow-up	Screening tool(s)<br>Included predictors & ascertainment<br>Risk prediction & handling of missing data	Outcomes predicted & ascertainment<br>Consideration of competing risk	Calibration outcomes & analyses
	(with the exception of calcium or vitamin D supplements) (n = 1695), missing femoral neck BMD measurement (measured lumbar spine only pre-1989), did not attend at follow-up		smoking and drinking status, dietary calcium intake, physical activity level. Height and weight were measured. BMD was measured at the lumbar spine (pre-1989) or femoral neck (1989 onward) via DXA, with T-scores calculated using the author's personal normative data.<br><br>**Prediction:** calculated using the FRAX website; unclear how many participants had missing data nor how missing data were handled	**Competing risk:** not considered; participants who died during follow-up (3.1%) or were lost for other reasons were excluded	
Yin 2016 [59], USA<br><br>Prospective cohort<br><br>**Funding:** government<br><br>**Related studies:** none	**Veterans Aging Cohort Study Virtual Cohort (VACS-VC):** 25,720 HIV-infected veterans matched with uninfected veterans by age, sex, race-ethnicity, and geographic region who enrolled for care in the Veterans Health Administration in the same calendar year. Veterans aged 50-70 years at year 2000 were included in the analysis.<br><br>**Exclusion:** weight exceeding 125 kg limit of the FRAX tool; missing data for FRAX variables	**Analyzed sample:** 24,451 (95% of original sample); 0% F; mean (SD) 55.6 (5.4) years; use of anti-osteoporosis drugs NR<br><br>**Baseline 10-y risk (mean):** 2.8% MOF and 0.3% hip for HIV+; 2.7% MOF and 0.2% hip for HIV-<br><br>**Follow-up:** 10 years	FRAX-US (modified; no BMD)<br><br>**Predictors:** extracted nine FRAX variables that were available in the VACS-VC database – age, race/ethnicity, weight, height (BMI), history of previous fragility fracture, ever glucocorticoid use, rheumatoid arthritis, alcohol use, current smoking.<br><br>**Prediction:** entered data into the FRAX website. Did not use parental history of hip fracture or secondary osteoporosis in the calculation because this information was not collected in the VACS-VC. Instead, a 'no' response was imputed for all.	**MOF (hip, shoulder, forearm, clinical vertebral), hip fractures:** collected via chart review using relevant ICD-9-CM codes, previously validated by chart review of 400 randomly selected radiology reports<br><br>**Competing risk:** not considered. Appears that participant observations were censored at death.	Expected fracture probability (by HIV status), observed fracture probability, observed fractures, O:E ratio<br><br>**Subgroups:** data available by level of risk (< and ≥3%) for hip fractures

BMD: bone mineral density; BMI (body mass index); DXA: dual-energy x-ray absorptiometry; F: female; HRT: hormone replacement therapy; MOF: major osteoporotic fracture; NHANES: National Health and Nutrition Examination Survey; P-Y: person-years; RCT: randomized controlled trial; y: year

REFERENCES

1. Azagra R, Zwart M, Encabo G, Aguye A, Martin-Sanchez J C, Puchol-Ruiz N, et al. Rationale of the Spanish FRAX model in decision-making for predicting osteoporotic fractures: An update of FRIDEX cohort of Spanish women. BMC Musculoskel Disord. 2016;17:262.
2. Azagra R, Roca G, Encabo G, Aguye A, Zwart M, Guell S, et al. FRAX(R) tool, the WHO algorithm to predict osteoporotic fractures: The first analysis of its discriminative and predictive ability in the Spanish FRIDEX cohort. BMC Musculoskelet Disord. 2012;13:204.
3. Azagra R, Roca G, Martin-Sanchez JC, Casado E, Encabo G, Zwart M, et al. FRAX(R) thresholds to identify people with high or low risk of osteoporotic fracture in Spanish female population. Med Clin (Barc). 2015;144(1):1-8.
4. Azagra R, Zwart M, Aguye A, Martin-Sanchez JC, Casado E, Diaz-Herrera MA, et al. Fracture experience among participants from the FROCAT study: What thresholding is appropriate using the FRAX tool? Maturitas. 2016;83:65-71.
5. Bolland MJ, Siu AT, Mason BH, Horne AM, Ames RW, Grey AB, et al. Evaluation of the FRAX and Garvan fracture risk calculators in older women. J Bone Miner Res. 2011;26(2):420-7.
6. Crandall CJ, Larson J, LaCroix A, Cauley JA, LeBoff MS, Li W, et al. Predicting fracture risk in younger postmenopausal women: Comparison of the Garvan and FRAX risk calculators in the Women's Health Initiative Study. J Gen Intern Med. 2019;34(2):235-42.
7. Crandall CJ, Larson JC., Watts NB, Gourlay ML, Donaldson MG, LaCroix A, et al. Comparison of fracture risk prediction by the US Preventive Services Task Force strategy and two alternative strategies in women 50-64 years old in the Women's Health Initiative. J Clin Endocrinol Metab. 2014;99(12):4514-22.
8. Czerwinski E, Borowy P, Kumorek A, Amarowicz J, Gorkiewicz M, Milert A. Fracture risk prediction in outpatients from Krakow Region using FRAX tool versus fracture risk in 11-year follow-up. Ortopedia, Traumatologia, Rehabilitacja. 2013;15(6):617-28.
9. Dagan N, Cohen-Stravi C, Leventer-Roberts M, Balicer RD. External validation and comparison of three prediction tools for risk of osteoporotic fractures using data from population based electronic health records: Retrospective cohort study. BMJ. 2017;356:i6755.
10. Desbiens L, Sidibe A, Beaudoin C, Jean S, Mac-Way F. Comparison of fracture prediction tools in individuals without and with early chronic kidney disease: a population-based analysis of CARTaGENE. J Bone Mineral Res. 2020;35(6): 1048–57.
11. Ettinger B, Liu H, Blackwell T, Hoffman AR, Ensrud KE, Orwoll ES. Validation of FRC, a fracture risk assessment tool, in a cohort of older men: the Osteoporotic Fractures in Men (MrOS) Study. J Clin Densitom. 2012;15(3):334-42.
12. Ettinger B, Ensrud KE, Blackwell T, Curtis JR, Lapidus JA, Orwoll ES, for the Osteoporotic Fracture in Men Study Research Group. Performance of FRAX in a cohort of community-dwelling, ambulatory older men: the Osteoporotic Fractures in Men (MrOS) study. Osteoporos Int. 2013;24(4):1185-93.
13. Gourlay ML, Ritter VS, Fine JP, Overman RA, Schousboe JT, Cawthon PM, et al. Comparison of fracture risk assessment tools in older men without prior hip or spine fracture: the MrOS study. Arch Osteoporos. 2017;12(1):91.
14. Orwoll ES, Lapidus J, Wang PY, Vandenput L, Hoffman A, Fink HA, et al. The limited clinical utility of testosterone, estradiol, and sex hormone binding globulin measurements in the prediction of fracture risk and bone loss in older men. J Bone Miner Res. 2017;32(3):633-40.
15. Harvey NC, Oden A, Orwoll E, Lapidus J, Kwok T, Karlsson MK, et al. Falls predict fractures independently of FRAX probability: A meta-analysis of the Osteoporotic Fractures in Men (MrOS) Study. J Bone Min Res. 2018;33(3):510-6.
16. Langsetmo L, Peters KW, Burghardt AJ, Ensrud KE, Fink HA, Cawthon PM, et al. Volumetric bone mineral density and failure load of distal limbs predict incident clinical fracture independent HR-pQCT BMD and failure load predicts incident clinical fracture of FRAX and clinical risk factors among older men. J Bone Miner Res. 2018;33(7):1302-11.
17. Buehring B, Hansen KE, Lewis BL, Cummings SR, Lane NE, Binkley N, et al. Dysmobility syndrome independently increases fracture risk in the Osteoporotic Fractures in Men (MrOS) prospective cohort study. J Bone Miner Res. 2018;33(9):1622-9.
18. Fraser LA, Langsetmo L, Berger C, Ioannidis G, Goltzman D, Adachi JD, et al. Fracture prediction and calibration of a Canadian FRAX(R) tool: A population-based report from CaMos. Osteoporos Int. 2011;22(3):829-37.
19. Leslie WD, Berger C, Lansetmo L, Lix LM, Adachi JD, Hanley DA, et al. Construction and validation of a simplified fracture risk assessment tool for Canadian women and men: Results from the CaMos and Manitoba cohorts. Osteoporos Int. 2011a;22(6):1873-83.
20. Goldshtein I, Gerber Y, Ish-Shalom S, Leshno M. Fracture risk assessment With FRAX using real-world data in a population-based cohort from Israel. Am J Epidemiol. 2018;187(1):94-102.
21. Holloway KL, Mohebbi M, Betson AG, Hans D, Hyde NK, Brennan-Olsen SL, et al. Prediction of major osteoporotic and hip fractures in Australian men using FRAX scores adjusted with trabecular bone score. Osteoporos Int. 2018;29(1):101-8.

22. Iki M, Fujita Y, Tamaki J, Douda K, Yura A, Sato Y, et al. Trabecular bone score may improve FRAX(R) prediction accuracy for major osteoporotic fractures in elderly Japanese men: The Fujiwara-kyo Osteoporosis Risk in Men (FORMEN) Cohort Study. Osteoporos Int. 2015;26(6):1841-8.
23. Langsetmo L, Nguyen TV, Nguyen ND, Kovacs CS, Prior JC, Center JR. Independent external validation of nomograms for predicting risk of low-trauma fracture and hip fracture. CMAJ. 2011;183(2):E107-14.
24. Leslie WD, Majumdar SR, Lix LM, Josse RG, Johansson H, Oden A, McCLoskey EV, Kanis JA. Direct comparison of FRAX(R) and a simplified fracture risk assessment tool in routine clinical practice: a registry-based cohort study. Osteoporos Int. 2016;27(9):2689-95.
25. Leslie WD, Tsang JF, Lix LM. Simplified system for absolute fracture risk assessment: Clinical validation in Canadian women. J Bone Miner Res. 2009;24(2):353-60.
26. Leslie WD, Lix LM. Simplified 10-Year absolute fracture risk assessment: A comparison of men and women. J Clin Densitom. 2010b;13(2):141-6.
27. Leslie WD, Majumdar SR, Morin SN, Lix LM, Johansson H, Oden A, McCloskey EV, et al. FRAX for fracture prediction shorter and longer than 10 years: The Manitoba BMD registry. Osteoporos Int. 2017b;28(9):2557-64.
28. Leslie WD, Lix LM, Johansson H, Oden A, McCloskey E, Kanis JA.. Independent clinical validation of a Canadian FRAX tool: Fracture prediction and model calibration. J Bone Miner Res. 2010a;25(11):2350-8.
29. Leslie WD, Majumdar S. Fracture burden in relation to low bone mineral density and FRAX((R)) probability. J Clin Densitom. 2011b;14(3):279-85.
30. Leslie WD, Lix LM, Johansson H, Oden A, McCloskey E, Kanis JA. A comparative study of using non-hip bone density inputs with FRAX(R). Osteoporos Int. 2012a;23(3):853-60.
31. Leslie WD, Majumdar S, Lix LM, Johansson H, Oden A, McCloskey E, Kanis JA. Fracture risk assessment without bone density measurement in routine clinical practice. Osteoporos Int. 2012b;23(1):75-85.
32. Leslie WD, Brennan SL, Langsetmo L, Lix LM, Adachi JD, Hanley DA, et al. Direct comparison of eight national FRAX(R) tools for fracture prediction and treatment qualification in Canadian women. Arch Osteoporos. 2013;8:145.
33. Leslie WD, Johansson H, Kanis JA, Lamy O, Oden A, McCloskey EV, et al. Lumbar spine texture enhances 10-year fracture probability assessment. Osteoporos Int. 2014;25(9):2271-7.
34. Brennan SL, Leslie WD, Lix LM, Johansson H, Oden A, McCloskey E, Kanis JA. FRAX provides robust fracture prediction regardless of socioeconomic status. Ostoeporos Int. 2014;25(1):61-9.
35. Majumdar SR, Leslie WD, Lix LM, Morin SN, Johansson HL, Oden A, et al. Longer duration of diabetes strongly impacts fracture risk assessment: The Manitoba BMD cohort. J Clin Endocrinol Metab. 2016;101(11):4489-96.
36. Martineau PL, W. D.; Johansson, H.; Oden, A.; McCloskey, E. V.; Hans, D.; Kanis, J. A. Clinical Utility of Using Lumbar Spine Trabecular Bone Score to Adjust Fracture Probability: The Manitoba BMD Cohort. J Bone Miner Res. 2017;32(7):1568-74.
37. Leslie WD, Lix LM, Majumdar SR, Morin SN, Johansson H, Oden A, McCloskey EV, Kanis JA. Total hip bone area affects fracture prediction with FRAX in Canadian white women. J Clin Endocrinol Metab. 2017a;102(11):4242-9.
38. Leslie WD, Majumdar SR, Morin SN, Lix LM, Schousboe JT, Ensrud KE, et al. Performance of FRAX in clinical practice according to sex and osteoporosis definitions: The Manitoba BMD registry. Osteoporos Int. 2018;29(3):759-67.
39. Bolton JM, Morin SN, Majumdar SR, Sareen J, Lix LM, Johansson H, et al. Association of mental disorders and related medication use with risk for major osteoporotic fractures. JAMA Psychiatry. 2017;74(6):641-8.
40. Lix LM, Leslie WD, Majumdar SR. Measuring improvement in fracture risk prediction for a new risk factor: a simulation. BMC Res Notes. 2018;11(1):62.
41. Yang S, Leslie WD, Morin SN, Lix LM. Administrative healthcare data applied to fracture risk assessment. Osteoporos Int. 2019;30(3):565-71.
42. Crandall CJ, Schousboe JT, Morin SN, Lix L, Leslie W. Performance of FRAX and FRAX-based treatment thresholds in women aged 40 years and older: The Manitoba BMD registry. J Bone Miner Res. 2019;28:28.
43. Li G, Thabane L, Papaioannou A, Adachi JD. Comparison between frailty index of deficit accumulation and fracture risk assessment tool (FRAX) in prediction of risk of fractures. Bone. 2015;77:107-14.
44. Lo JC, Pressman AR, Chandra M, Ettinger B. Fracture risk tool validation in an integrated healthcare delivery system. Am J Manag Care. 2011;17(3):188-94.
45. Pressman AR, Lo JC, Chandra M, Ettinger B. Methods for assessing fracture risk prediction models: experience with FRAX in a large integrated health care delivery system. J Clin Denistom. 2011;14(4):407-15.
46. Marques A, Lucas R, Simoes E, Verstappen SMM, Jacobs JWG, da Silva JAP. Do we need bone mineral density to estimate osteoporotic fracture risk? A 10-year prospective multicentre validation study. RMD Open. 2017;3(2):e000509.
47. Melton III LJ, Atkinson EJ, Achenbach SJ. Kanis JA, Therneau Tm, Johansson H, et al. Potential Extensions of the US FRAX Algorithm. J Osteoporos. 2012;2012:528790.

48. Pluskiewicz W, Adamczyk P, Czekajlo A, Grzeszczak W, Drozdzowska B. High fracture probability predicts fractures in a 4-year follow-up in women from the RAC-OST-POL study. Osteoporos Int. 2015;26(12):2811-20.
49. Premaor M, Parker RA, Cummings S, Ensrud K, Cauley JA, Lui LY, et al. Predictive value of FRAX for fracture in obese older women. J Bone Miner Res. 2013;28(1):188-95.
50. Hillier TA, Cauley JA, Rizzo JH, Pedula KL, Ensrud KE, Bauer DC, et al. WHO absolute fracture risk models (FRAX): do clinical risk factors improve fracture prediction in older women without osteoporosis? J Bone Miner Res. 2011;26(8):1774-82.
51. Langsetmo L, Peters KW, Burghardt AJ, Ensrud KE, Fink HA, Cawthon PM, et al. Volumetric bone mineral density and failure load of distal limbs predict incident clinical fracture independent HR-pQCT BMD and failure load predicts incident clinical fracture of FRAX and clinical risk factors among older men. J Bone Miner Res. 2018;33(7):1302-11.
52. Reyes Dominguez AI, Sosa Cabrera N, Saavedra Santana P, de Tejada Romero MJG, Jodar Gimeno E, Sosa Henriquez M. Assessment of the predictive capacity of the garvan calculator of 10 year risk of fracture in a Spanish population. Revista de Osteoporosis y Metabolismo Mineral. 2017;9(2):55-61.
53. Premaor M, Parker RA, Cummings S, Ensrud K, Cauley JA, Lui LY, et al. Predictive value of FRAX for fracture in obese older women. J Bone Miner Res. 2013;28(1):188-95.
54. Tamaki J, Iki M, Sato Y, Winzenrieth R, Kajita E, Kagamimori S, et al. Does Trabecular Bone Score (TBS) improve the predictive ability of FRAX for major osteoporotic fractures according to the Japanese Population-Based Osteoporosis (JPOS) cohort study? J Bone Miner Metab. 2019;37(1):161-70.
55. Tamaki J, Iki M, Kadowaki E, Sato Y, Kajita E, Kagamimori S, et al. Fracture risk prediction using FRAX(R): a 10-year follow-up survey of the Japanese Population-Based Osteoporosis (JPOS) Cohort Study. Osteoporos Int. 2011;22(12):3037-45.
56. Tanaka S, Yoshimura N, Kuroda T, Josoi T, Saito M, Shiraki M. The Fracture and Immobilization Score (FRISC) for risk assessment of osteoporotic fracture and immobilization in postmenopausal women--A joint analysis of the Nagano, Miyama, and Taiji Cohorts. Bone. 2010;47(6):1064-70.
57. Tebé Cordomí C, del Río LM, Di Gregorio S, Casas L, Estrada M-D, Kotzeva A, Espallargues M. Validation of the FRAX predictive model for major osteoporotic fracture in a historical cohort of Spanish women. J Clin Densitom. 2013;16(2):231-7.
58. Trémollieres FA, Pouilles JM, Drewniak N, Laparra J, Ribot CA, Dargent-Molina P. Fracture risk prediction using BMD and clinical risk factors in early postmenopausal women: sensitivity of the WHO FRAX tool. J Bone Miner Res. 2010;25(5):1002-9.
59. Yin MT, Shiau S, Riland D, Gibert CL, Bedimo RJ, Rodriguez-Barradas MC, et al. Fracture prediction with modified-FRAX in older HIV-infected and uninfected men. JAIDS. 2016;72(5):513-20.

Additional Table 6.2. Characteristics of trials included for KQ3a on the benefits of pharmacologic treatments

**Author & year; Setting**<br>**Design; Funding source**<br>**Length of follow-up**	**Population characteristics**	**Treatment(s) & Comparators (s) of interest**<br>**Adherence**	**Outcomes & Ascertainment**<br>**Available subgroups**
Ascott-Evans 2003 [1]<br>18 centres in 9 countries (Argentina, Australia, Austria, Brazil, Finland, Germany, New Zealand, Spain, South Africa)<br><br>2-arm RCT (parallel)<br><br>Industry<br><br>Follow-up: 1 year	144 postmenopausal females (% of eligible NR) with low lumbar spine BMD (T-score between -3.5 and -1.5), previously treated with hormone replacement therapy and stopped within 3 months before the study; mean (SD) 57.3 (6.6) years old; no prior osteoporotic fractures (exclusion criteria); baseline fracture risk NR<br><br>Exclusion: history of metabolic bone disease, osteoporotic fracture, or recent use of bisphosphonates and/or drugs known to affect bone metabolism	a) Oral alendronate 10 mg/day for 1 year (n = 95)<br>b) Oral placebo for 1 year (n = 49)<br><br>+ calcium 500 mg/day<br><br>Adherence NR	**Hip fractures** (not defined): self-reported as AEs<br>**Clinical fractures** (not defined): self-reported as AEs<br><br>Subgroups: none
Bell 2002 [2]<br>8 centres geographically distributed across USA<br><br>2-arm RCT (parallel)<br><br>Funding NR<br><br>Follow-up: 2 years	65 African-American postmenopausal females (% of eligible NR) with low lumbar spine BMD (≤0.86 g/cm^2); mean (SD) 66.2 (8.8) years old; prior fracture NR; baseline fracture risk NR<br><br>Exclusion: disease or drug therapy affecting bone metabolism; >1 lumbar spine fracture; abnormal renal function or a history of cancer or major upper gastrointestinal mucosal erosive disease	a) Oral alendronate 10 mg/day for 2 years (n = 33)<br>b) Oral placebo for 2 years (n = 33)<br><br>+ calcium 500 mg/day, vitamin D 500 IU/day<br><br>Adherence NR	**Clinical fractures** (not defined): self-reported as AEs<br><br>Subgroups: none
Bone 2008 [3]<br>21 centres in the USA and Canada<br><br>2-arm RCT (parallel)<br><br>Industry<br><br>Follow-up: 2 years	332 postmenopausal ambulatory females (100% of eligible) with low lumbar spine BMD (T-scores -1.0 to -2.5); mean (SD) 59.4 (7.5) years old; no prior fractures (inclusion criteria); baseline fracture risk NR<br><br>Exclusion: oral bisphosphonates use for ≥ 3 years; recent treatment with anti-osteoporosis drugs; underlying condition that might result in abnormal bone metabolism	a) Subcutaneous denosumab, 60 mg every 6 months for 2 years (n = 166)<br>b) Subcutaneous placebo for 2 years (n = 166)<br><br>+ calcium ≥1000 mg/day, vitamin D ≥400 mg/day<br><br>Adherence: 329 (99%) received at least one dose of study medication; 86% completed treatment	**Clinical fractures** (new vertebral or nonvertebral fractures; excluded skull, facial bones, mandible, metacarpals, phalanges of the fingers/toes or if they were the result of severe trauma): self-reported and confirmed radiographically<br>**All-cause mortality**: NR<br><br>Subgroups: none
Boonen 2012 [4]<br>Europe, South America, Africa, and Australia<br><br>2-arm RCT (parallel)<br><br>Industry<br><br>Follow-up: 2 years	1199 males (% of eligible NR) with low BMD (T score ≤–1.5) at the total hip or femoral neck for those with between 1 and 3 prevalent mild/moderate vertebral fractures or low BMD (T-score of ≤ -2.5) at the hip, femoral neck, or lumbar spine for those without prior fractures; median (range) 66 (50-85) years old; prior osteoporotic fracture NR (32% had prevalent vertebral fractures); baseline fracture risk NR<br><br>Exclusion: ≥4 prevalent vertebral fractures; low serum 25-hydroxyvitamin D; renal insufficiency; hyper/hypocalcemia; treatment with anti-osteoporosis	a) Intravenous zoledronic acid 5 mg at baseline and 1 year (n = 588)<br>b) Intravenous placebo at baseline and 1 year (n = 611)<br><br>+ calcium 1000 to 1500 mg/day, vitamin D 800 to 1200 IU/day	**Hip fractures** (not defined): self-reported at each visit and verified centrally by means of a radiographic report or surgical notes<br>**Clinical fractures** (vertebral and nonvertebral): self-reported at each visit and verified centrally by means of a radiographic report or surgical notes<br>**All-cause mortality**: NR

Author & year; Setting<br>Design; Funding source<br>Length of follow-up	Population characteristics	Treatment(s) & Comparators (s) of interest<br>Adherence	Outcomes & Ascertainment<br>Available subgroups
	drugs if washout period not met; testosterone in prior year; anabolic steroids or growth hormone in prior 6 months; bilateral hip replacement; hyperthyroidism; primary hyperparathyroidism	Adherence: 52 men who received zoledronic acid (8.8%) and 53 men who received placebo (8.7%) did not receive the second infusion	Subgroups: none
Chesnut 1995 [5]<br>7 centres geographically distributed across the US<br><br>6-arm RCT (parallel)<br><br>Industry<br><br>Follow-up: 2 years	188 postmenopausal females (100% of eligible) with low lumber spine BMD (≤0.88 g/cm^2); mean (SD) 63.0 (6.3) years old; no prior spine or hip fractures (exclusion criteria); baseline fracture risk NR<br><br>Exclusion: any disease or drug therapy potentially affecting bone metabolism; presence of spine or hip fractures attributable to osteoporosis	a) Oral alendronate groups (n = 157):<br>i. 5 mg/day for 2 years;<br>ii. mg/day for 2 years;<br>iii. 40 mg/day for 3 months followed by 2.5 mg/day for 21 months;<br>iv. 20 mg/day for 1 year then placebo for 1 year;<br>v. 40 mg/day for 1 year then placebo for 1 year<br>b) Oral placebo for 2 years<br><br>+ calcium 500 mg/day	**Hip fracture** (not defined): self-reported as AEs and assessed by physician investigator<br>**Clinical fractures** (nonvertebral fractures): ascertainment NR<br>**All-cause mortality** (deaths during the study - considered an AE): ascertainment NR<br><br>Subgroups: NR
Cummings 1998 [6]<br>11 clinical centres in the USA<br><br>2-arm RCT (parallel)<br><br>Industry<br><br>Follow-up: 4 years<br><br>Associated publications: Hochberg 2005 [7]; Donaldson 2012 [8]	4432 postmenopausal females (100% of eligible) with low femoral neck BMD (≤0.68 g/cm^2); mean (SD) 67.7 (6.1) years old; 35.5% prior fracture; mean (SD) FRAX 10-y MOF 27.0 (12.3) with BMD, 24.3 (12.2) without BMD<br><br>Exclusion: recent peptic ulcers; dyspepsia requiring daily treatment; renal or hepatic dysfunction; severe malabsorption; hypertension; myocardial infarction within 6 months; unstable angina; hypothyroidism or hyperparathyroidism; estrogen or calcitonin use in prior 6 months; bisphosphonates or sodium fluoride use (>1 mg/d) at any time; vertebral fracture in the alendronate group	a) Oral alendronate 5 mg/day for 2 years, then 10 mg/day for 2 years (n = 2214)<br>b) Placebo for 4 years (n = 2218)<br><br>+ 500 mg calcium, 250 IU vitamin D if dietary intake was low<br><br>Adherence: At closeout, 82.5% of surviving placebo participants and 81.3% of alendronate participants were still taking study medication	**Hip fractures** (excluded pathologic fractures or fractures due to trauma): diagnosed by a physician and self-reported, confirmed by written reports of radiographs or other tests<br>**Clinical fractures** (excluded pathologic fractures or fractures due to trauma, facial and skull fractures): diagnosed by a physician and confirmed by written reports of radiographs or other tests<br>**All-cause mortality**: NR<br><br>Subgroups: baseline BMD, FRAX score; age
Cummings 2009 [9]<br>International study centres<br><br>2-arm RCT (parallel)<br><br>Industry<br><br>Follow-up: 3 years<br><br>Associated publications: Boonen 2011 [10]; McClung 2012 [11]; McCloskey 2012 [12]; Silverman 2012 [13]	7868 females (100% of eligible) with low BMD (T-score <2.5) at the lumbar spine or total hip; mean (SD) 72.3 (5.2) years old; 34% had a prior nonvertebral fracture; baseline 10 year major osteoporotic fracture risk assessed with FRAX for those with BMD in the treatment group was median (IQR) 15.1 (10.4-21.7) and 15.1 (10.4-21.4) in the control group. Without BMD was 16.9 (11.2-24.0) for the treatment group and 16.7 (11.4-24.3) for the control group; baseline 10 year hip fracture risk assessed with FRAX for those with BMD was 4.8 (2.5-8.7) in the treatment group and 4.8 (2.5-8.7) in the control group. Without BMD was 6.2 (3.5-10.6) in the treatment group and 6.1 (3.5-10.7) in the control group<br><br>Exclusion: conditions that influence bone metabolism; oral bisphosphonates use for >3 years (but were eligible after 12 months without treatment);	a) Subcutaneous denosumab 60 mg every 6 months for 36 months (n = 3922)<br><br>b) Subcutaneous placebo every 6 months for 36 months (n = 3935)<br><br>+ At least calcium 1000 mg/day. Those with a baseline 25-hydroxyvitamin D level of 12-20 ng/ml were given at least vitamin D 800 IU/day, and those with a baseline level above 20 ng/ml were given at least 400 IU/day<br><br>Adherence: 5979 (76%) received all injections	**Hip fractures** (femur neck, femur intertrochanter, and femur subtrochanter; excluded pathologic and traumatic fractures): self-reported, confirmed by diagnostic imaging or a radiologist's report<br>**Clinical fractures** (nonvertebral fractures excluding the skull, face, mandible, metacarpals, fingers, toes, pathologic and traumatic fractures): self-reported, confirmed by diagnostic imaging or a radiologist's report<br>**All-cause mortality**: recorded as AEs at physician study sites

Author & year; Setting<br>Design; Funding source<br>Length of follow-up	Population characteristics	Treatment(s) & Comparators (s) of interest<br>Adherence	Outcomes & Ascertainment<br>Available subgroups
	intravenous bisphosphonates, fluoride, or strontium use for osteoporosis within the past 5 years; use of parathyroid hormone or its derivatives, corticosteroids, systemic hormone-replacement therapy, selective estrogen-receptor modulators, tibolone, calcitonin, or calcitriol in prior 6 weeks; BMD T score <–4.0 at the lumbar spine or total hip, severe prevalent vertebral fractures; low serum 25-hydroxyvitamin D		**Quality of life or wellbeing** (Health-related Quality of Life): self-administered Osteoporosis Assessment Questionnaire-Short-version (OPAQ-SV)<br><br>Subgroups: age, baseline BMD, baseline FRAX, prior fracture, age + BMD
Fogelman 2000 [14]<br>13 centres in France, the UK, the Netherlands, Belgium, and Germany<br><br>3-arm RCT (parallel)<br><br>Industry<br><br>Follow-up: 4 years	543 postmenopausal females (% of eligible NR) with low lumbar spine BMD (T-score ≤-2); mean (SD) 64.7 (7.2) years old; 30.1% had a prior vertebral fracture (other fractures NR); baseline fracture risk NR<br><br><u>Exclusion:</u> hyperparathyroidism, hyperthyroidism, or osteomalacia within a year before the study; history of cancer; abnormalities that would interfere with the measurement of lumbar spine BMD by dual-energy x-ray absorptiometry (DXA); use of medications (within 6–12 months before the study) known to affect bone metabolism, including an injection of vitamin D ≥ 10,000 IU.	a) Risedronate groups (n = 363):<br>i. oral risedronate 2.5 mg/day for 2 years; this group was discontinued by protocol amendment at 9 of the 13 centres;<br>ii. oral risedronate 5 mg/day for 2 years<br><br>b) Oral placebo (n = 180)<br><br>+ calcium 1000 mg/day<br><br>Adherence: 355 (65%) patients completed 24 months of treatment: 143 (79%) in the placebo group, 73 (40%) in the risedronate 2.5-mg group; 76 were withdrawn due to protocol amendment (68% of remaining completed 24 months), and 139 (78%) in the 5-mg risedronate group	**Clinical fractures** (nonvertebral fractures): self-reported as AEs and spontaneous reports<br><br>Subgroups: none
Grey 2009 [15]<br>Clinical research facility in Auckland, New Zealand<br><br>Government<br><br>Follow-up: 2 years	50 postmenopausal females (27% of eligible) with BMD T-score between -1 and -2 at the lumbar spine or total hip; mean (SD) 63.5 (8.1) years old; no prior hip or vertebral fractures (exclusion criteria), other fractures NR; baseline fracture risk NR<br><br><u>Exclusion:</u> illnesses or therapies known to affect the skeleton; low bone mass (BMD T score at lumbar spine or total hip ≤-2); prior hip or vertebral fracture; ever used bisphosphonates; any other major systemic disease	a) Intravenous zoledronic acid 5 mg single infusion (n = 25)<br><br>b) Intravenous placebo single infusion (n = 25)<br><br>Adherence: All patients received one dose of the study drug. One withdrew.	**Hip fractures** (not defined): ascertainment NR<br>**Clinical fractures** (incident fractures - not defined): ascertainment NR<br><br>Subgroups: none
Grey 2014 [16]<br>Auckland, New Zealand<br><br>4-arm RCT (parallel)<br><br>Government, industry	180 postmenopausal females (100% of eligible) with a low BMD (T-score between -1 and -2.5) at either lumbar spine or total hip, not taking medications known to affect bone health, and had a baseline serum 25(OH)D level >25 nmol/L; mean (SD) 65.3 (8.5) years old; 16.9% had a prior fracture during adulthood; baseline fracture risk NR<br><br><u>Exclusion:</u> NR	a) Zoledronic acid groups (n = 135):<br>i. intravenous zoledronic acid 1 mg single infusion<br>ii. intravenous zoledronic acid 2.5 mg single infusion<br>iii. intravenous zoledronic acid 5 mg single infusion	**Hip fractures** (not defined): ascertainment NR<br>**Clinical fractures** (incident fractures - not defined): ascertainment NR<br>**All-cause mortality** (deaths during the study): ascertainment NR<br><br>Subgroups: none

**Author & year; Setting Design; Funding source Length of follow-up**	**Population characteristics**	**Treatment(s) & Comparators (s) of interest Adherence**	**Outcomes & Ascertainment Available subgroups**
Associated publication(s): Grey 2017 (5-y open label extension) [17] & Grey 2012 (1-y follow up) [18]<br><br>Follow-up: 2 years (5 years for mortality in Grey 2017 [17])		b) Placebo (100 ml of 0.9% NaCl) single infusion<br><br>Adherence: 2 in each group did not receive the study medication	
Hooper 2005 [19]<br>11 centres in Australia<br><br>3-arm RCT (parallel)<br><br>Industry<br><br>Follow-up: 2 years	383 postmenopausal females (% of eligible NR) with a lumbar spine BMD T-score >-2.5, a serum follicle stimulating hormone concentration of at least 50 mIU/ml, and a serum estradiol concentration of no more than 20 pg/ml; mean (SD) 52.7 (3.2) years old; prior fracture NR; baseline fracture risk NR<br><br><u>Exclusion:</u> NR	a) Risedronate groups (n = 257):<br>i. oral risedronate 2.5 mg/day<br>ii. oral risedronate 5 mg/day<br><br>b) Oral placebo daily (n = 126)<br><br>+ calcium 1000 mg/day<br><br>Adherence: 296 (77%) completed the study/treatment	**Clinical fractures** (incident non-vertebral fractures): self-reported as AEs<br><br>Subgroups: none
Hosking 1998 [20]<br>4 study centres in USA, Denmark, UK<br><br>4-arm RCT (parallel) - 3 arms of interest<br><br>Industry<br><br>Follow-up: 2 years	1000 postmenopausal females (% eligible NR) in good health with no clinical or laboratory evidence of systemic disease, proportion of participants with low lumbar spine BMD (<0.8 g/cm^2) was limited to 10%; mean (SD) 53.3 (4.0) years old; prior fracture NR; baseline fracture risk NR<br><br><u>Exclusion:</u> abnormal renal function; history of cancer; peptic ulcer or esophageal disease requiring prescription medication within the previous five years; previous bisphosphonate or fluoride use; regular therapy with a phosphate-binding antacid; estrogen-replacement therapy within the previous three months; therapy with any other drug that affects the skeleton	a) Oral alendronate 5 mg/day for 2 years (n = 498)<br><br>b) Oral placebo daily for 2 years (n = 502)<br><br>+ those with a calcium intake of less than 500 mg/day were advised to increase their intake<br><br>Adherence: 905 (91%) completed all 24 months of treatment (409 in placebo, 396 in alendronate)	**Hip fractures** (not defined): self-reported AEs<br>**Clinical fractures** (not defined): self-reported AEs<br>**All-cause mortality** (deaths during the study considered a serious AE): outcome NR<br><br>Subgroups: none
Hosking 2003 [21]<br>38 sites in Europe (Belgium, Finland, France, Germany, Italy, Sweden, Spain, UK) and Brazil<br><br>3-arm RCT (parallel)<br><br>Industry<br><br>Follow-up: 1 year	549 postmenopausal females (100% of eligible) ≥ 60 and ≤ 90 years of age with osteoporosis as defined by low BMD (lumbar spine or total hip BMD T-score ≤ –2.5, or both lumbar spine and total hip BMD T-score ≤ –2.0); mean (SD) 69.2 (6.4) years old; 48.4% prior fracture; baseline fracture risk NR.<br><br><u>Exclusion:</u> history of any illness or if significant abnormalities that might compromise the patient's safety or the evaluation of the study results; patients with osteoporosis so severe participation in a placebo controlled trial was unethical; baseline 25-hydroxyvitamin D level below 9 ng/ml, or below 15 ng/ml with biochemical evidence of osteomalacia; metabolic and other bone diseases; prior concomitant oestrogen preparations (>2 weeks within 6 months), thyroid hormone (<6 weeks before the study or with abnormal	a) Oral alendronate 70 mg/week for 1 year (n = 219)<br><br>b) Oral risedronate 5 mg/day (n = 222)<br><br>c) Oral placebo for 1 year (n = 108)<br><br>Adherence: >75% over the first 3 months of the study in 95% of alendronate and risedronate groups, 99% of placebo group	**Clinical fractures** ('clinically diagnosed vertebral or nonvertebral'): self-reported as AEs<br><br>Subgroups: none

Author & year; Setting<br>Design; Funding source<br>Length of follow-up	Population characteristics	Treatment(s) & Comparators (s) of interest<br>Adherence	Outcomes & Ascertainment<br>Available subgroups
	thyroid stimulating hormone), fluoride (>1 mg/day), glucocorticoids (>1 month within 6 months), bisphosphonate (>2 weeks), supplemental calcium (except if ongoing for >4 weeks).		
Lewiecki 2007 [22]<br>29 centres in the USA<br>9-arm RCT (parallel)<br><br>Industry<br><br>Follow-up: 2 years<br><br>Associated publication: McClung 2006 (1-year follow-up) [23]	412 postmenopausal females (100% of eligible) ≤80 years old with a BMD T-score of –1.8 to –4.0 at the lumbar spine or –1.8 to –3.5 at the femoral neck or total hip; mean (SD) 62.1 (8.5) years old; no long bone fractures in past 6 months or osteoporotic fractures in past 2 years (exclusion); baseline fracture risk NR.<br><br><u>Exclusion:</u> use of bisphosphonates within 12 months or fluoride within 24 months; tibolone, PTH or any derivative, systemic glucocorticoids, inhaled glucocorticoids, anabolic steroids, or testosterone within 6 months; and estrogens, selective estrogen receptor modulators, calcitonin, or calcitriol within 3 months of enrollment; hyper- or hypoparathyroidism, hyper- or hypothyroidism, hypocalcemia, rheumatoid arthritis, Paget's disease of bone, osteomalacia, creatinine clearance <35 ml/minute, malabsorption syndrome; recent long-bone fracture (within 6 months), >1 grade 1 vertebral fracture, osteoporosis-related fracture within the last 2 years.	a) Subcutaneous denosumab groups (n = 319)<br>i. 6, 14, or 30 mg every 3 months for 2 years<br>ii. 14, 60, 100, or 210 mg every 6 months (alternating with placebo) for 2 years<br><br>b) Oral alendronate 70 mg/week (open-label) for 2 years (n = 47)<br><br>c) Subcutaneous placebo every 3 months for 2 years (n = 46)<br><br>+ calcium 1000 mg/day, vitamin D 200 IU/day<br><br>Adherence: 98.5% received at least one dose	**Clinical fractures** ('osteoporotic' fractures): self-reported as AEs<br>**All-cause mortality** (not defined): ascertainment NR
Li 2005 [24]<br>China<br><br>2-arm RCT (parallel)<br><br>Funding NR<br><br>Follow-up: 1 years	60 postmenopausal females (% of eligible NR) in good health who do not smoke or drink alcohol, without organ disease, bone metabolic diseases, do not use medications that affect bone metabolism, and had low lumbar spine BMD (T-score ≤-2.5) for at least three evaluable vertebrae in the L1—L4 region; mean (SD) age NR but participants were between 45-68 years old (inclusion criteria); prior fracture NR; baseline fracture risk NR<br><br><u>Exclusion:</u> NR	a) Oral risedronate 5mg/day for 1 year (n = 30)<br><br>b) Oral placebo daily (n = 30)<br><br>+ calcium 600 mg/day, vitamin D (Caltrate D) 125 IU/day<br><br>Adherence: 6 (10%) did not complete the study (2 in treatment, 4 in control). Appears that those who completed took the study drugs.	**Hip fractures** (new fractures): self-reported and physical examination<br>**Clinical fractures** (new fracture): self-reported and physical examination<br><br>Subgroups: none
Liberman 1995 [25]<br>18 centres in USA (one RCT); Australia, Canada, Europe, Israel, Mexico, New Zealand, South America (other RCT)<br><br>4-arm RCT (parallel)<br><br>Industry	994 postmenopausal females (% of eligible NR) with low lumbar spine BMD (2.5 SD below the mean value in premenopausal white females); mean 64 years old (SD NR); prior osteoporotic fracture NR (20.5% had prior vertebral fracture); baseline fracture risk NR<br><br><u>Exclusion:</u> other causes of osteoporosis; other disorders of bone and mineral metabolism; active peptic ulcer disease; abnormal renal or hepatic function; abnormalities of the lumbar spine precluding the assessment of bone mineral density at a minimum of three lumbar vertebrae or a history of hip fracture;	a) Alendronate groups (n = 526):<br>i. oral alendronate 5 mg/day for 3 years;<br>ii. oral alendronate 10 mg/day for 3 years;<br>iii. oral alendronate 20 mg/day for 2 years + 5 mg oral alendronate daily for 1 year<br><br>b) Oral placebo daily for 3 years<br><br>+ calcium 500 mg/day	**Hip fractures** (not defined): recorded if symptomatic at follow-up<br>**Clinical fractures** (symptomatic nonvertebral fractures): recorded if symptomatic at follow-up<br>**All-cause mortality** (not defined): ascertainment NR - 2 deaths reported by Tucci 1996 (USA subset), but the group assignment is not mentioned

Author & year; Setting<br>Design; Funding source<br>Length of follow-up	Population characteristics	Treatment(s) & Comparators (s) of interest<br>Adherence	Outcomes & Ascertainment<br>Available subgroups
Associated publication: Tucci 1996 (Fractures data from the USA trial) [26]<br><br>Follow-up: 3 years	any prior treatment with bisphosphonates, estrogen, progestin, calcitonin, fluoride, or an anabolic steroid within the preceding 12 months	Adherence: 160 (27%) discontinued treatment at some point during the study	Subgroups: none
McClung 2009 [27]<br>25 centres in France, Spain, UK, USA, Sweden<br><br>3-arm RCT (parallel)<br><br>Industry<br><br>Follow-up: 2 years	581 postmenopausal females (100% of eligible) with low BMD at the lumbar spine (T-score <–1.0 and >–2.5) and femoral neck (T-score >-2.5); mean (SD) 60.0 (7.9) years old; prior fracture NR; baseline fracture risk NR<br><br><u>Exclusion:</u> more than one grade 1 vertebral facture or with any grade 2 or 3 vertebral fracture; vitamin D level less than 15 ng/mL before randomization; renal insufficiency; hypercalcemia or hypocalcemia; use or prior treatment with oral bisphosphonates, calcitonin, SERMs, estrogen, or tibolone (except according to specified washout schedule)	a) Zoledronic groups (n = 379):<br>i. intravenous zoledronic acid 5 mg at baseline and month 12;<br>ii. intravenous zoledronic acid 5 mg at baseline followed by placebo at month 12<br><br>b) Placebo infusion at baseline and month 12<br><br>+ calcium 500-1200 mg/day, vitamin D 400-800 IU/day<br><br>Adherence: 58 (10%) did not complete the study; appears all those who completed the study received the study drug	**Clinical fractures** (not defined): self-reported AEs (assumed)<br>**All-cause mortality** (deaths during the study): regular safety monitoring of AEs<br><br>Subgroups NR
McClung 2001 [28]<br>183 study centers in North America, New Zealand, and Australia<br><br>2-arm RCT (parallel)<br><br>Industry<br><br>Follow-up: 3 years (mean follow-up for all participants was 2.3 years)	9331 postmenopausal ambulatory females (98.3% of eligible) who (a) were 70-79 y and osteoporotic with a low BMD at the femoral neck (T-score >4 SD below mean peak value in young adults or <-3) plus at least one risk factor for hip fracture or (b) were ≥ 80 y and had at least one nonskeletal risk factor for hip fracture, with a low BMD at the femoral neck (T-score <-4 or <-3 with a hip-axis length ≥11.1); mean (SD) 77.7 (5.4) years old; 30% had prior vertebral fracture (other fractures NR); baseline fracture risk NR<br><br><u>Exclusion:</u> major medical illness; recent history of cancer; another metabolic bone disease within the previous year; important abnormalities in the results of routine laboratory tests; recent use of drugs known to affect bone; allergy to any bisphosphonate; history of bilateral hip fractures; any physical or mental condition precluding participation	a) Oral risedronate 2.5 mg or 5.0 mg daily for 3 years (n = 6197)<br><br>b) Placebo tablet daily for 3 years (n = 3134)<br><br>+ calcium 1000 mg/day, vitamin D ≤500 IU/day was given if the serum 25-hydroxyvitamin D concentration at the time of screening was below 16 ng/ml (40 nmol/L)<br><br>Adherence: 3093 (50%) in the risedronate group and 1584 (51%) in the placebo group completed treatment	**Hip fractures** (all hip fractures): radiographically confirmed<br>**Clinical fractures** (nonvertebral osteoporotic fractures of the wrist, leg, humerus, hip, pelvis or clavicle): radiographically confirmed<br><br>Subgroups: age; risk factors; BMD; vertebral fractures at baseline
Mortensen 1998 [29]<br>Two study centres in USA and Denmark<br><br>3-arm RCT (parallel)<br><br>Industry	111 postmenopausal ambulatory females (% of eligible NR) with estradiol levels ≥40 pg/mL and FSH ≥20 U/L, normal lumbar spine BMD (within 2 SD of age matched mean bone mass), weigh between 45 and 90 kg and be within 25% of normal weight and height values; mean (SD) 51.5 (3.8) years old; no prior osteoporotic fractures (exclusion criteria); baseline fracture risk NR	a) Risedronate groups (n = 75)<br>i. Cyclic risedronate: oral 5 mg/day for 2 weeks, followed by 2 weeks of placebo each week for 2 years;<br>ii. daily risedronate: oral 5 mg/day for 2 years<br><br>b) Oral placebo daily for 2 years (n = 36)	**Hip fractures** (part of nonvertebral fractures): self-reported AEs (assumed)<br>**Clinical fractures** (nonvertebral fractures): self-reported AEs (assumed)<br><br>Subgroups: none

Author & year; Setting<br>Design; Funding source<br>Length of follow-up	Population characteristics	Treatment(s) & Comparators (s) of interest<br>Adherence	Outcomes & Ascertainment<br>Available subgroups
Follow-up: 3 years (2 years treatment + 1 follow-up)	Exclusion: any use of bisphosphonate, thyroid hormone therapy, glucocorticoids, anabolic agents, calcitonin, vitamin D, high-dose calcium, diuretics, or anticonvulsants for more than 1 month within the previous 6 months; estrogens and/or progestogens use for more than 1 month within the past year; fluoride use for more than 1 month ever in the past; history of any generalized bone disease; history of alcohol or drug abuse; significant organic or psychiatric disease; established osteoporosis (e.g., atraumatic vertebral deformity or a history of osteoporosis related fracture of the hip or wrist); bilateral oophorectomy or any other type of artificially induced menopause	Adherence NR	
Orwoll 2012 [30]<br>Multicentre: USA, Denmark, Sweden, France, Poland, Canada, Belgium<br><br>2-arm RCT (parallel)<br><br>Industry<br><br>Follow-up: 1 year	242 ambulatory males (% of eligible NR) with low BMD (T-score ≤-2.0 and ≥-3.5) at the lumbar spine or femoral neck, or had a previous major osteoporotic fracture and low BMD (T-score ≤-1.0 and ≥-3.5) at the lumbar spine or femoral neck; mean (SD) 65.0 (9.8); 39.3% had prior fracture (any type), and 14.9% had a prior major osteoporotic fracture; baseline 10 year major osteoporotic fracture risk assessed with FRAX was mean (SD) 9.8 (6.3)<br><br>Exclusion: any severe or more than one moderate vertebral fracture on screening spinal x-ray; any vertebral fracture or clinical fracture diagnosed within 6 months before screening; any disease known to affect bone metabolism; low serum 25(OH)-vitamin D; any bisphosphonate use ≥3 months cumulatively in the previous 2 years or for ≥1 month in the past year or any use in the 3 months before randomization; use of anabolic steroids or testosterone, glucocorticoids, calcitonin, calcitriol or vitamin D derivatives, and other bone-active drugs in the 3 months before screening	a) Subcutaneous denosumab, 60mg every 6 months for 1 year (at baseline and month 6) (n = 121)<br><br>b) Subcutaneous placebo for 1 year (at baseline and month 6) (n = 121)<br><br>+ calcium 1000 mg/day, at least vitamin D 800 IU/day<br><br>Adherence: NR (appears that those who completed the study completed the injections of denosumab)	**Hip fractures** (not defined): self-reported AEs (assumed)<br>**Clinical fractures** (not defined): self-reported AEs (assumed)<br>**All-cause mortality** (death during the study): ascertainment NR<br><br>Subgroups: none
Pitale 2015 [31]<br>11 centres in India<br><br>2-arm RCT (parallel)<br><br>Industry<br><br>Follow-up: 6 months	250 postmenopausal females (84.7% of eligible) with low BMD (T-score <-2.5 and >-4.0) at either the lumbar spine or total hip; mean (SD) 62.6 (5.0) years old; 7.2% had a prior fracture; baseline 10 year major osteoporotic fracture risk assessed with FRAX was mean (SD) 7.5 (4.4) when Hologic machine used for BMD and 7.6 (4.2) when Lunar machine used, while baseline hip fracture risk was 2.9 (2.7) when Hologic machine used for BMD and 3.0 (2.6) when Lunar machine used<br><br>Exclusion: metabolic bone diseases other than osteoporosis; hyper- or hypoparathyroidism, rheumatoid arthritis, malabsorption syndrome or prior treatment with drugs that alter bone metabolism; vitamin D deficiency; use of medications known or suspected to have activity on bone metabolism	a) Subcutaneous denosumab 60 mg at baseline (n = 124)<br><br>b) Subcutaneous placebo at baseline (n = 126)<br><br>+ at least calcium 1000 mg/day, at least vitamin D 400 IU/day<br><br>Adherence: all received 1 dose of the study drug	**Hip fractures** (not defined): self-reported AEs (assumed)<br>**Clinical fractures** (not defined): self-reported AEs (assumed)<br>**All-cause mortality** (death during the study): ascertainment NR<br><br>Subgroups: none

Author & year; Setting<br>Design; Funding source<br>Length of follow-up	Population characteristics	Treatment(s) & Comparators (s) of interest<br>Adherence	Outcomes & Ascertainment<br>Available subgroups
Pols 1999 [32]<br>153 centres in 34 countries (Europe, Latin America, Australia, Canada, South Africa, China)<br><br>2-arm RCT (parallel)<br><br>Industry<br><br>Follow-up: 12 months	1908 postmenopausal females (% of eligible NR) in good health with low lumbar spine BMD (at least 2 SD below the mean for premenopausal females; ≤0.86 g/cm² by Hologic QDR densitometry or ≤0.98 g/ cm² by Lunar DPX densitometry), and between 20% below and 50% above ideal body weight; mean (SD) 62.8 (7.4) years old; prior fracture NR; baseline fracture risk NR<br><br><u>Exclusion:</u> metabolic bone disease other than osteoporosis; disturbed parathyroid or thyroid function; major gastrointestinal disease within the year before enrollment or use of a drug to inhibit gastric acid secretion for >2 weeks within 3 months of study entry; myocardial infarction within the year prior to enrollment; uncontrolled hypertension or untreated angina; impaired renal function; end organ disease; bisphosphonate or fluoride use during the previous 6 months; estrogen, ipriflavone or calcitonin use during the previous 4 months; any anabolic steroid, glucocorticoid or progestin use for >2 weeks within the previous 6 months; use of medications that might alter bone or mineral metabolism	a) Oral alendronate 10 mg/day for 12 months<br><br>b) Placebo tablet for 12 months<br><br>+ calcium 500 mg/day<br><br>Adherence NR	**Hip fractures** (not defined): self-reported AEs<br>**Clinical fractures** (nonvertebral fractures): self-reported as AEs<br><br>Subgroups: NR
Reid 2002 [33]<br>24 centers in 10 countries<br><br>6-arm RCT (parallel)<br><br>Industry<br><br>Follow-up: 1 year	227 postmenopausal females (% of eligible NR) with low lumbar spine (L1 to L4) BMD (at least 2.0 SD below the mean value for young adults; T-score <-2), with no more than one vertebral fracture at screening; mean (SD) 64.1 (6.4) years old; prior osteoporotic fractures NR (no vertebral fractures at study entry); baseline fracture risk NR<br><br><u>Exclusion:</u> systemic estrogen treatment within the previous 3 months; evidence of secondary osteoporosis; clinical or laboratory evidence of hepatic or renal disease; disorders of the parathyroid or thyroid glands; serum 25-hydroxyvitamin D concentration of ≤15 ng/ml (37 nmol/L); history of cancer; previous bisphosphonates or fluoride use; current use of drug(s) known to affect the skeleton	a) Zoledronic acid groups (n = 168):<br>i. intravenous infusion zoledronic acid 1 mg every 3 months for 1 year;<br>ii. intravenous infusion zoledronic acid 4 mg once at the beginning of the trial;<br>iii. intravenous infusion zoledronic acid 2 mg at baseline and at 6 months<br><br>b) intravenous infusion saline placebo every 3 months for 1 year (n = 59)<br><br>+ calcium 1000 mg/day<br><br>Adherence NR	**Clinical fractures** (nonvertebral fractures): self-reported (assumed)<br><br>Subgroups: none
Reid 2018 [34]<br>Aukland region of New Zealand<br><br>2-arm RCT (parallel)<br><br>Government<br><br>Follow-up: 6 years	2000 postmenopausal ambulatory females (100% of eligible) with low BMD (T-score of -1.0 to -2.5) at either the total hip or femoral neck; mean (SD) 71 (5.0) years old; 23.8% had a prior nonvertebral fracture after age 45 y and 13.2% had a prior vertebral fracture; baseline 10 year major osteoporotic fracture risk assessed with FRAX was median (IQR) 12 (9-16)% for zoledronate group and 12 (9-15)% for the placebo group, baseline 10 year hip fracture risk was 2.4 (1.5-3.9)% for the zoledronic acid group and 2.3 (1.5-3.8)% for the placebo group	a) 4 infusions of zoledronic acid 5 mg at 18 month intervals (n = 64)<br><br>b) 4 infusions of normal saline (placebo) at 18 month intervals (n = 75)<br><br>+ vitamin D 2.5 mg (100,000 IU) single dose 1 week before first infusion followed by 1.25 mg/month	**Hip fractures** (not defined): self-reported and if hospitalized, diagnosis was confirmed from the participant's medical records; symptomatic fractures were confirmed by radiology reports or radiographs<br>**Clinical fractures** (all symptomatic vertebral fractures and all nonvertebral fractures; excluded fractures of the toes, metatarsal

Author & year; Setting<br>Design; Funding source<br>Length of follow-up	Population characteristics	Treatment(s) & Comparators (s) of interest<br>Adherence	Outcomes & Ascertainment<br>Available subgroups
	<u>Exclusion</u>: estimated glomerular filtration rate <30 ml/minute per 1.73 m2 of body-surface area; major systemic disease; cancer in the previous 2 years; metabolic bone disease; regular use of bone-active drugs in the previous year	infusion for the duration of the trial; calcium 1 mg/day was advised but not provided<br><br>Adherence: 806 (81%) in the zolendronic acid group and 825 (83%) in the placebo group received four doses of the trial regimen.	bones, fingers, metacarpal bones, skull, facial bones, mandible, and pathologic fractures): self-reported and if hospitalized, diagnosis was confirmed from the participant's medical records; symptomatic fractures were confirmed by radiology reports or radiographs<br>**All-cause mortality** (deaths during the study): vital status confirmed with the use of a national database of death records at the end of trial<br><br>Subgroups: none for outcome of interest
Välimäki 2007 [35]<br>14 study centres across Europe (Finland, Netherlands, Norway, Spain, Sweden)<br><br>2-arm RCT (parallel)<br><br>Industry<br><br>Follow-up: 2 years	171 ambulatory postmenopausal females (% of eligible NR) with a low lumbar spine BMD (between -2.5 and -1 SD below mean value for young adults), had ≥1 other risk factor for osteoporosis, presence of hip osteopenia (proximal femur T-score ≤-1), and were not taking HRT, calcitriol, or calcitonin treatment 12, 4, and 4 weeks prior to enrollment; mean (SD) 65.9 (6.8) years old; prior fracture NR; baseline fracture risk NR<br><br><u>Exclusion:</u> history of cancer within the 5 years before the study; any condition that might interfere with the evaluation of lumbar spine BMD; any disease requiring long-term treatment with systemic corticoids; bisphosphonate use within 6 months of starting the study treatment or for >14 days within 1 year before the start of the study	a) Oral risedronate 5 mg/day for 2 years (n = 114)<br><br>b) Placebo tablet for 2 years (n = 57)<br><br>+ calcium 1000 mg/day, vitamin D 400 IU/day<br><br>Adherence: >90% in both treatment groups (94% risedronate and 90% placebo)	**Hip fractures** (not defined): self-reported or investigator observed AEs<br>**Clinical fractures** (nonvertebral fractures – not defined; clinical vertebral fractures also reported): self-reported or investigator observed AEs<br>**All-cause mortality** (deaths during the study): investigator observed AEs<br><br>Subgroups: none
Yan 2009 [36]<br>7 centres in China<br><br>2-arm RCT (parallel)<br><br>Government, industry<br><br>Follow-up: 1 year	560 postmenopausal females (% of eligible NR) with low lumbar spine BMD (at least 2 SD below the mean bone mass of normal young Chinese females), no prevalent vertebral fractures on radiographs; mean (SD) 64.9 (6.2) years old; prior fracture NR; baseline fracture risk NR<br><br><u>Exclusion:</u> history of diseases that affect calcium or bone metabolism, other than postmenopausal bone loss; serious liver or heart disease, or renal dysfunction; bisphosphonate, anabolic steroid, estrogen or estrogen-related drug use within the last 12 months; glucocorticoid or fluoride use within the last 6 months; supplements with vitamin D within the last 3 months	a) Oral alendronate 70 mg/week for 12 months (n = 280)<br><br>b) Oral placebo for 12 months (n = 280)<br><br>+ 2 Calcichew/day (calcium 500 mg, vitamin D 200 IU)<br><br>Adherence: participants completed diaries which were validated with tablet counts. Data NR.	**Hip fractures** (whether or not associated with trauma): safety evaluations performed at each visit and participants also self-reported as AEs<br>**Clinical fractures** (whether or not associated with trauma): safety evaluations performed at each visit and participants also self-reported as AEs<br>**All-cause mortality** (deaths during the study): ascertainment NR<br><br>Subgroups: none

Author & year; Setting<br>Design; Funding source<br>Length of follow-up	Population characteristics	Treatment(s) & Comparators (s) of interest<br>Adherence	Outcomes & Ascertainment<br>Available subgroups
Zhu 2017 [37]<br>8 GlaxoSmithKline investigational sites in China<br><br>2-arm RCT (parallel)<br><br>Industry<br><br>Associated publication: Zhu 2016 (registration) [38]<br><br>Follow-up: 1 year	486 postmenopausal ambulatory Chinese females (99.8% of eligible) with low BMD (T-score <-2.5 and >-4.0) at either the lumbar spine or total hip, with at least one other risk factor; mean (SD) 69.0 (6.0) years old; prior fracture NR; baseline fracture risk NR<br><br>Exclusion: metabolic bone disease, hypo- or hyperparathyroidism; thyroid condition; rheumatoid arthritis; malignancy; liver disease; physical or psychiatric disorder compromising participation; human immunodeficiency virus; vitamin D deficiency; history of oral/dental conditions; prior use of bisphosphonates ≥3 years or <3 years with last dose <1 year prior to enrolment; use of drugs affecting bone metabolism in prior 6 weeks; laboratory abnormalities that could interfere with the study; abnormal serum calcium; <2 evaluable lumbar vertebrae; history of >2 vertebral fractures or very high fracture risk needing to be treated with drugs.	a) Subcutaneous denosumab 60mg at baseline and 6 months (n = 365)<br><br>b) Subcutaneous placebo at baseline and at 6 months (n = 119)<br><br>+ at least calcium 600 mg/day, vitamin D 400 IU/day<br><br>Adherence: 484 (99.8%) received at least one dose of investigational product	**Hip fractures** (femoral neck fracture): self-reported as SAEs<br>**Clinical fractures** (any event - injury, poisoning, procedural complication;, humerus, lumbar fractures reported): self-reported as SAEs<br>All-cause mortality (fatal adverse event – fatalities during the study): recorded as a AEs<br><br>Subgroups: none

AE=adverse event; BMD=bone mineral density; DXA=dual-energy x-ray absorptiometry; IU=international units; MOF=major osteoporotic fracture; NR=not reported; RCT=randomized controlled trial; SAE serious adverse event; SD=standard deviation; USA=United States of America; UK=United Kingdom

REFERENCES

1. Ascott-Evans BH, Guañabens N, Kivinen S, Stuckey BGA, Magaril CH, Vandormael K, et al. Alendronate prevents loss of bone density associated with discontinuation of hormone replacement therapy: A randomized controlled trial. Arch Intern Med. 2003;163(7):789-94.
2. Bell NH, Bilezikian JP, Bone Iii HG, Kaur A, Maragoto A, Santora AC. Alendronate increases bone mass and reduces bone markers in postmenopausal African-American women. J Clin Endocrinol Metab. 2002;87(6):2792-7.
3. Bone HG, Bolognese MA, Yuen CK, Kendler DL, Wang H, Liu Y, et al. Effects of denosumab on bone mineral density and bone turnover in postmenopausal women. J Clin Endocrinol Metab. 2008;93(6):2149-57.
4. Boonen S, Reginster J-Y, Kaufman J-M, Lippuner K, Zanchetta J, Langdahl B, et al. Fracture risk and zoledronic acid therapy in men with osteoporosis. N Engl J Med. 2012;367(18):1714-23.
5. Chesnut CH, McClung MR, Ensrud KE, Bell NH, Genant HK, Harris ST, et al. Alendronate treatment of the postmenopausal osteoporotic woman: Effect of multiple dosages on bone mass and bone remodeling. Am J Med. 1995;99(2):144-52.
6. Cummings SR, Black DM, Thompson DE, Applegate WB, Barrett-Connor E, Musliner TA, et al. Effect of alendronate on risk of fracture in women with low bone density but without vertebral fractures results from the Fracture Intervention Trial. JAMA. 1998;280(24):2077-82.
7. Hochberg MC, Thompson DE, Black DM, Quandt SA, Cauley J, Geusens P, et al. Effect of alendronate on the age-specific incidence of symptomatic osteoporotic fractures. J Bone Miner Res. 2005;20(6):971-6.
8. Donaldson MG, Palermo L, Ensrud KE, Hochberg MC, Schousboe JT, Cummings SR. Effect of alendronate for reducting fracture by FRAX score and femoral neck bone mineral density: The Fracture Intervention Trial. J Bone Miner Res. 2012;27(8):1804-10.
9. Cummings SR, Martin JS, McClung MR, Siris ES, Eastell R, Reid IR, et al. Denosumab for prevention of fractures in postmenopausal women with osteoporosis. N Engl J Med. 2009;361(8):756-65.
10. Boonen S, Adachi JD, Man Z, Cummings SR, Lippuner K, Törring O, et al. Treatment with denosumab reduces the incidence of new vertebral and hip fractures in postmenopausal women at high risk. J Clin Endocrinol Metab. 2011;96(6):1727-36.
11. McClung MR, Boonen S, Törring O, Roux C, Rizzoli R, Bone HG, et al. Effect of denosumab treatment on the risk of fractures in subgroups of women with postmenopausal osteoporosis. J Bone Miner Res. 2012;27(1):211-8.
12. McCloskey EV, Johansson H, Oden A, Austin M, Siris E, Wang A, et al. Denosumab reduces the risk of osteoporotic fractures in postmenopausal women, particularly in those with moderate to high fracture risk as assessed with FRAX. J Bone Miner Res. 2012;27(7):1480-6.
13. Silverman S, Viswanathan HN, Yang YC, Wang A, Boonen S, Ragi-Eis S, et al. Impact of clinical fractures on health-related quality of life is dependent on time of assessment since fracture: Results from the FREEDOM trial. Osteoporos Int. 2012;23(4):1361-9.
14. Fogelman I, Ribot C, Smith R, Ethgen D, Sod E, Reginster JY. Risedronate reverses bone loss in postmenopausal women with low bone mass: Results from a multinational, double-blind, placebo-controlled trial. BMD-MN study group. J Clin Endocrinol Metab. 2000;85(5):1895-900.
15. Grey A, Bolland MJ, Wattie D, Horne A, Gamble G, Reid IR. The antiresorptive effects of a single dose of zoledronate persist for two years: A randomized, placebo-controlled trial in osteopenic postmenopausal women. Obset Gynecol Surv. 2009;64(7):462-3.
16. Grey A, Bolland M, Mihov B, Wong S, Horne A, Gamble G, et al. Duration of antiresorptive effects of low-dose zoledronate in osteopenic postmenopausal women: A randomized, placebo-controlled trial. J Bone Miner Res. 2014;29(1):166-72.
17. Grey A, Bolland M, Horne A, Mihoc B, Gamble G, Reid IR. Duration of antiresorptive activity of zoledronate in postmenopausal women with osteopenia: a randomized, controlled multidose trial. CMAJ. 2017;189(36):E1130-6.
18. Grey A, Bolland M, Wong S, Horne A, Gamble G, Reid IR. Low-dose zoledronate in osteopenic postmenopausal women: A randomized controlled trial. J Clin Endocrinol Metab. 2012;97(1):286-92.
19. Hooper MJ, Ebeling PR, Roberts AP, Graham JJ, Nicholson GC, D'Emden M, et al. Risedronate prevents bone loss in early postmenopausal women: A prospective randomized, placebo-controlled trial. Climacteric. 2005;8(3):251-62.

20. Hosking D, Adami S, Felsenberg D, Andia JC, Valimaki M, Benhamou L, et al. Comparison of change in bone resorption and bone mineral density with once-weekly alendronate and daily risedronate: A randomised, placebo-controlled study. Curr Med Res Opin. 2003;19(5):383-94.
21. Hosking D, Chilvers CED, Christiansen C, Ravn P, Wasnich R, Ross P, et al. Prevention of bone loss with alendronate in postmenopausal women under 60 years of age. N Engl J Med. 1998;338(8):485-92.
22. Lewiecki EM, Miller PD, McClung MR, Cohen SB, Bolognese MA, Liu Y, et al. Two-year treatment with denosumab (amg 162) in a randomized phase 2 study of postmenopausal women with low BMD. J Bone Miner Res. 2007;22(12):1832-41.
23. McClung MR, Lewiecki EM, Cohen SB, Bolognese MA, Woodson GC, Moffett AH, et al. Denosumab in postmenopausal women with low bone mineral density. N Engl J Med. 2006;354:821-31.
24. Li Y, Zhang Z, Deng X, Chen L. Efficacy and safety of risedronate sodium in treatment of postmenopausal osteoporosis. Journal of Huszhong University of Science and Technology. 2005;25(5):527-9.
25. Liberman UI, Weiss SR, Bröll J, Minne MW, Quan H, Bell NG, et al. Effect of oral alendronate on bone mineral density and the incidence of fractures in postmenopausal osteoporosis. N Engl J Med. 1995;33(22):1437-43.
26. Tucci JR, Tonino RP, Emkey RD, Peverly CA, Kher U, Santora AC, 2nd. Effect of three years of oral alendronate treatment in postmenopausal women with osteoporosis. Am J Med. 1996;101(5):488-501.
27. McClung M, Miller P, Recknor C, Mesenbrink P, Bucci-Rechtweg C, Benhamou CL. Zoledronic acid for the prevention of bone loss in postmenopausal women with low bone mass: A randomized controlled trial. Obstet Gynecol. 2009;114(5):999-1007.
28. McClung MR, Geusens P, Miller PD, Zippel H, Bensen WG, Roux C, et al. Effect of risedronate on the risk of hip fracture in elderly women. N Engl J Med. 2001;344(5):333-40.
29. Mortensen L, Charles P, Bekker PJ, Digennaro J, Johnston CC, Jr. Risedronate increases bone mass in an early postmenopausal population: Two years of treatment plus one year of follow-up. J Clin Endocrinol Metab. 1998;83(2):396-402.
30. Orwoll E, Teglbjaerg CS, Langdahl BL, Chapurlat R, Czerwinski E, Kendler DL, et al. A randomized, placebo-controlled study of the effects of denosumab for the treatment of men with low bone mineral density. J Clin Endocrinol Metab. 2012;97(9):3161-9.
31. Pitale S, Thomas M, Rathi G, Deshmukh V, Kumar P, Reddy S, et al. A randomized placebo-controlled trial of the efficacy of denosumab in indian postmenopausal women with osteoporosis. Indian J Endocrinol Metab. 2015;19(1):148-54.
32. Pols HAP, Felsenberg D, Hanley DA, Štepán J, Muñoz-Torres M, Wilkin TJ, et al. Multinational, placebo-controlled, randomized trial of the effects of alendronate on bone density and fracture risk in postmenopausal women with low bone mass: Results of the FOSIT study. Osteoporos Int. 1999;9(5):461-8.
33. Reid IR, Brown JP, Burckhardt P, Horowitz Z, Richardson P, Trechsel U, et al. Intravenous zoledronic acid in postmenopausal women with low bone mineral density. N Engl J Med. 2002;346(9):653-61.
34. Reid IR, Horne AM, Mihov B, Stewart A, Garratt E, Wong S, et al. Fracture prevention with zoledronate in older women with osteopenia. N Engl J Med. 2018;379(25):2407-16.
35. Välimäiki MJ, Farrerons-Minguella J, Halse J, Kröger H, Maroni M, Mulder H, et al. Effects of risedronate 5 mg/d on bone mineral density and bone turnover markers in late-postmenopausal women with osteopenia: A multinational, 24-month, randomized, double-blind, placebo-controlled, parallel-group, phase III trial. Clin Ther. 2007;29(9):1937-49.
36. Yan Y, Wang W, Zhu H, Li M, Liu J, Luo B, et al. The efficacy and tolerability of once-weekly alendronate 70 mg on bone mineral density and burn turnover markers in postmenopausal Chinese women with osteoporosis. J Bone Miner Metab. 2009;27(4):471-8.
37. Zhu HM, Tang H, Cheng Q, He L, Li PQ, Xue QY, et al. Efficacy and safety of denosumab in Chinese postmenopausal women with osteoporosis at increased risk of fracture: results from a 12-month, randomized, double-blind, placebo-controlled phase III study. J Bone Miner Res. 2017;31. https://doi.org/10.1002/jbmr.3107
38. GlaxoSmithKline (responsible party). Denosumab China phase III study. NCT02014467. https://clinicaltrials.gov/ct2/show/NCT02014467

Additional Table 6.3. Characteristics of systematic reviews included for KQ3b on the harms of pharmacologic treatments

Author & year<br>Funding source	Date of search	Study eligibility	Risk of bias appraisal<br>Certainty appraisal	Outcomes & Ascertainment
**Chen 2015** [1]<br><br>No external funding	Inception to June 2014	**Design:** cohorts<br>**Population:** females and males with osteoporosis<br>**Interventions:** alendronate or bisphosphonate (any dose) vs. controls (not specified)	Newcastle-Ottawa Scale<br><br>Certainty not assessed	**GI cancer:** separate analyses for each of colorectal, gastric, esophageal, liver, pancreatic, oral, bile duct, small intestinal
**Crandall 2014 (AHRQ)** [2, 3]<br><br>Government	January 2005 to March 2014 (updating an earlier report); later updated to July 2016 for bisphosphonates [4]	**Design:** RCTs, large (n>1,000) observational studies and case reports for rare events<br>**Population:** adults with or without low bone density/osteoporosis (could be due to chronic use of glucocorticoids, but not other diseases of bone metabolism)<br>**Interventions:** alendronate, risedronate, zoledronic acid, denosumab (any FDA-approved dose) vs. placebo	ROB not assessed for harm outcomes<br><br>Strength of evidence using AHRQ methods (similar to GRADE) for selected outcomes	**Non-serious GI AE:** conditions such as acid reflux, esophageal irritation, nausea, vomiting, and heartburn<br>**Influenza-like symptoms:** separate analyses for 'influenza-like symptoms', and composite of arthralgia, myalgia, pyrexia, chills, and influenza-like symptoms<br>**Musculoskeletal pain:** separate analyses for arthritis, arthralgia; myalgia, cramps, limb pain<br>**Serious cardiovascular AE:** separate analyses for acute coronary syndrome, cerebrovascular death, serious cerebrovascular accidents, pulmonary embolism, thromboembolic events, serious cardiac events<br>**Serious cardiac rhythm disturbances:** atrial fibrillation<br>**Serious GI AE (excluding cancer):** separate analyses for all serious GI AE; GI perforations, ulcers, bleeds; serious esophageal AE; serious hepatobiliary AE<br>**GI cancer:** separate analyses for esophageal cancer, GI cancer, colon cancer<br>**Dermatologic AE:** separate analyses for injection site reactions; rash/eczema<br>**Infections:** NR; used a previously published pooled analysis<br>**Atypical femoral fractures:** atypical (low-stress) subtrochanteric or femoral fractures<br>**Osteonecrosis of the jaw:** NR
**Davis 2016 (NIHR)** [5]<br><br>Government	2008 to September 2014	**Design:** RCTs; non-randomized studies if needed<br>**Population:** females ≥65 and males ≥75 years, or younger with low BMD (T-score ≤-1) or risk factors.<br>**Interventions:** alendronate (10mg/day or 70 mg/week), risedronate (5 mg/day or 35 mg/week), zoledronic acid (5 mg/year) vs. placebo or non-active treatments	ROB not assessed for harm outcomes<br><br>Certainty not assessed	**Any non-serious AE:** any adverse event<br>**Influenza-like symptoms:** variable - upper respiratory infections, influenza, pyrexia, headache, chills, nasopharyngitis, bronchitis, pneumonia, cough, fatigue<br>The symptoms analyzed varied across drugs based on trial reporting
**Davis 2020 (NIHR)** [6]<br><br>Government	Inception to July 2018	**Design:** RCTs<br>**Population:** females ≥65 and males ≥75 years, or younger with presence of risk factors.<br>**Interventions:** denosumab (60 mg/6 months) vs. placebo or non-active treatments	ROB not assessed for harm outcomes<br><br>Certainty not assessed	**Any non-serious AE:** any adverse event<br>**Any serious AE:** number of patients experiencing any serious AE<br>Serious cardiovascular AE: separate analyses for stroke, venous thromboembolism<br>Venous thromboembolism: NR<br>**Atypical femoral fractures:** NR, as described in the included studies<br>**Osteonecrosis of the jaw:** NR, as described in the included studies
**Diedhou 2015** [7]<br><br>Funding NR	Date of search NR	**Design:** RCTs, prospective cohorts<br>**Population:** females and males treated to prevent or reduce fractures	ROB not assessed<br><br>Certainty not assessed	**Musculoskeletal pain:** arthralgia

Author & year<br>Funding source	Date of search	Study eligibility	Risk of bias appraisal<br>Certainty appraisal	Outcomes & Ascertainment
		**Interventions:** denosumab (60 mg/6 months) vs. placebo		
**Fink 2019 (AHRQ)** [8]<br><br>Government	January 1995 to October 2018	**Design:** RCTs, observational studies<br>**Population:** females and males ≥50 years on osteoporosis treatment for >3 years (rare harms);<br>**Interventions:** alendronate, zoledronic acid, denosumab (any dose) vs. placebo	ROB not assessed for harm outcomes<br><br>GRADE	**Atypical femoral fracture:** subtrochanteric or femoral fractures with atypical features (with or without radiologic confirmation). Excluded pathologic, periprosthetic, traumatic fractures.<br>**Osteonecrosis of the jaw:** defined by diagnostic codes ± clinical confirmation
**Kranenburg 2016** [9]<br><br>Funding NR	Inception to January 2016	**Design:** RCTs<br>**Population:** any patients treated for ≥1 year<br>**Interventions:** alendronate, risedronate, zoledronic acid (any dose) vs. placebo or no treatment	Cochrane ROB tool<br><br>Certainty not assessed	**Serious cardiovascular AE:** separate analyses for cardiovascular mortality, stroke, myocardial infarction, and composite or nonfatal stroke, nonfatal myocardial infarction, death due to vascular cause.
**Lv 2020** [10]<br><br>Government	Inception to June 2019	**Design:** RCTs<br>**Population:** participants with primary osteoporosis or osteopenia and without disorders likely to affect bone metabolism, with follow-up of ≥6 months<br>**Interventions:** denosumab (any market-approved dose) vs. placebo	Cochrane ROB tool<br><br>Certainty not assessed	**Serious cardiovascular AE:** separate analyses for three composite cardiovascular endpoints: 1) cardiovascular death or death, myocardial infarction, stroke; 2) [1] and heart failure, 3) stroke, atrial fibrillation, heart failure, coronary heart disease
**Tsourdi 2020** [11]<br><br>No external funding	Inception to August 2020	**Design:** RCTs, observational studies including case series<br>**Population:** studies where patients discontinued denosumab (includes cancer patients and those receiving glucocorticoid treatment). Excluded those with metastatic disease, metabolic bone disease.<br>**Interventions:** denosumab and its discontinuation vs. discontinuation of placebo	ROB not assessed<br><br>Certainty not assessed	**Rebound fractures (hip, clinical, clinical vertebral, multiple clinical vertebral):** fractures that occurred after stopping treatment.
**Viswanathan 2018 (USPSTF)** [12, 13]<br><br>Government	November 2009 to October 2016; active surveillance through March 2018	**Design:** RCTs, observational studies published since any recent systematic review<br>**Population:** studies where the majority of adults with increased risk of fracture<br>**Interventions:** alendronate, risedronate, zoledronic acid, denosumab (FDA-approved doses) vs. placebo or no treatment	ROB not assessed for harm outcomes<br><br>Strength of evidence using USPSTF methods	**Discontinuations due to AE:** discontinuation attributed to AEs, including any of: cardiovascular events, hot flashes, esophageal cancer, gastrointestinal events, osteonecrosis of the jaw, atypical fractures of the femur, and rashes.<br>**Serious AE:** NR, appears to include any serious AE<br>**Serious cardiac rhythm disturbances:** atrial fibrillation

AE=adverse event; AHRQ=Agency for Healthcare Research and Quality; BMD=bone mineral density; EPC=Evidence-based Practice Centre; FDA=United States Food and Drug Administration; GI=gastrointestinal; GRADE: Grading of Recommendations Assessment, Development and Evaluation; NIHR=National Institute for Health Research; NR=not reported; RCT=randomized controlled trial; ROB=risk of bias; USPSTF=United States Preventive Services Task Force; vs.=versus

REFERENCES

1. Chen LX, Ning GZ, Zhou ZR, Li YL, Zhang D, Wu QL, et al. The carcinogenicity of alendronate in patients with osteoporosis: evidence from cohort studies. PloS One. 2015;10(4):e0123080.
2. Crandall CJ, Newberry SJ, Diamant A, Lim YW, Gellad WF, Booth MJ, et al. Comparative effectiveness of pharmacologic treatments to prevent fractures: An updated systematic review. Ann Intern Med. 2014;161(10):711-23.
3. Crandall CJ, Newberry SJ, Diamant A, Lim YW, Gellad WF, Suttorp MJ, et al. Treatment to prevent fractures in men and women with low bone density or osteoporosis: Update of a 2007 report. Rockville, MD: Agency for Healthcare Research and Quality; 2012.
4. Qaseem A, Forciea MA, McLean RM, Denberg TD, Clinical Guidelines Committee of the American College of Physicians. Treatment of low bone density or osteoporosis to prevent fractures in men and women: A clinical practice guideline update from the American College of Physicians. Ann Intern Med. 2017;166(11):818-39.
5. Davis S, Martyn-St James M, Sanderson J, Stevens J, Goka E, Rawdin A, et al. A systematic review and economic evaluation of bisphosphonates for the prevention of fragility fractures. Health Technol Assess. 2016;20(78):1-406.
6. Davis S, Simpson E, Hamilton J, Martyn-St James M, Rawdin A, Wong R, et al. Denosumab, raloxifene, romosozumab and teriparatide to prevent osteoporotic fragility fractures: a systematic review and economic evaluation. Health Technol Assess. 2020;24(29):1-343.
7. Diedhiou D, Cuny T, Sarr A, Norou Diop S, Klein M, Weryha G. Efficacy and safety of denosumab for the treatment of osteoporosis: A systematic review. Ann Endocrinol. 2015;76(6):650-7.
8. Fink HA, MacDonald R, Forte ML, Rosebush CE, Ensrud KE, Schousboe JT, et al. Long-term drug therapy and drug holidays for osteoporosis fracture prevention: A systematic review. Rockville, MD: Agency for Healthcare Research and Quality; 2019.
9. Kranenburg G, Bartstra JW, Weijmans M, de Jong PA, Mali WP, Verhaar HJ, et al. Bisphosphonates for cardiovascular risk reduction: A systematic review and meta-analysis. Atherosclerosis. 2016;252:106-15.
10. Lv F, Cai X, Yang W, Gao L, Chen L, Wu J, et al. Denosumab or romosozumab therapy and risk of cardiovascular events in patients with primary osteoporosis: Systematic review and meta- analysis. Bone. 2020;130:115121.
11. Tsourdi E, Zillikens MC, Meier C, Body J-J, Gonzalez Rodriguez E, Anastasilakis AD. Fracture risk and management of discontinuation of denosumab therapy: A systematic review and position statement by ECTS. J Clin Endocrinol Metab. 2020; Oct 26;dgaa756.
12. Viswanathan M, Reddy S, Berkman N, Cullen K, Middleton JC, Nicholson WK, et al. Screening to prevent osteoporotic fractures: Updated evidence report and systematic review for the US Preventive Services Task Force. JAMA. 2018;319(24):2532-51.
13. Viswanathan M, Reddy S, Berkman N, Cullen K, Middleton JC, Nicholson WK, et al. Screening to prevent osteoporotic fractures: An evidence review for the US Preventive Services Task Force. Rockville, MD: Agency for Healthcare Research and Quality; 2018.

Table 6.4. Characteristics of studies included for KQ3b on the harms from discontinuation of denosumab treatment

Author & year; Setting<br>Design; Funding source<br>Length of follow-up	Population characteristics	Treatment(s) & Comparators (s) of interest	Outcomes & Ascertainment<br>Available subgroups
Tripto-Shkolnik 2020<br>State-mandated health organization in Israel<br><br>Retrospective cohort study linking healthcare system (medication purchase) with osteoporosis registry data<br><br>Follow-up: 9 (4.8-12) months after discontinuation	3110 (91% females) new initiators of denosumab with 2 or more consecutive (less than 3 refill gap) medication purchases starting from January 2012; mean (SD) 72.3 (9.2) years old; 42.4% prior fractures; 5.4% first-line therapy<br><br>Exclusion: <12 and 15 months pre and post (respectively) denosumab initiation date continuous membership in the health organization	a) Discontinuation (refill gap 3+ months) (n=1500)<br>b) Persistent users (n=1610)	**Rebound fractures (i.e. multiple clinical vertebral fractures):** registry data with adjudicated by a further manual review of electronic medical records by an expert endocrinologist; within 1 yr from discontinuation vs. sustained from the end of first treatment year and onwards (in persistence user group)<br><br>Subgroups: None for this (rare) outcome

REFERENCE

1. Tripto-Shkolnik L, Fund N, Rouach V, Chodick G, Shalev V, Goldshtein I. Fracture incidence after denosumab discontinuation: Real-world data from a large healthcare provider. *Bone*. 2020;130:115150.

Additional Table 6.5. Characteristics of studies included for KQ4 on the acceptability of screening and/or treatment

Author & Year, Country<br>Design<br>Study description	Participant characteristics	Format of information<br>Knowledge of risk<br>Information provided on benefits and harms	Outcomes of interest<br>Subgroup data
			–
De Bekker-Grob 2008 [1], Netherlands<br><br>Cross-sectional<br><br>Discrete choice experiment using hypothetical drug treatment profiles and five treatment attributes: effectiveness of treatment (reduction of risk of hip fracture), nausea as an adverse effect of treatment, total treatment duration, route of drug administration and costs.	n = 120 (66% of eligible) community dwelling women ≥60 years from 34 general practices in the area of Rotterdam who participated in a study on osteoporosis case finding<br><br>**Age, mean (SD):** 71.8 (7.9) y<br>**Menopausal status:** NR<br>**BMD:** NR<br>**Prior fracture:** NR<br>**Osteoporosis dx:** NR<br>**Medication use:** NR<br>**Concern about fractures:** NR<br>**Perceived severity of fractures:** NR<br>**Absolute fracture risk:** 60 (50%) had a hip fracture risk ≤6% (low risk) and 60 (50%) had a hip fracture risk >6% (high risk) based on a simple risk score using Dutch guidelines.<br>**Perceived fracture risk:** NR<br>**Previous screening:** NR	**Format:** Participants completed a discrete choice experiment where they chose between sets of two different treatment profiles, with the option of no treatment. Each treatment profile had different levels of five attributes: effectiveness, nausea as an adverse effect, duration, route of administration, cost.<br><br>**Knowledge of risk:** Participants were provided their lifetime fracture risk (high or low) based on a simple risk score using Dutch guidelines<br><br>**Benefits of treatment:** 10-year risk reduction in hip fracture could be 5%, 10%, 25%, or 50%. The current drug treatment was considered to be a weekly oral bisphosphonate taken for 5 years that could provide a 30% fracture risk reduction.<br><br>**Harms of treatment:** Nausea could either be present or not present. The current drug treatment was considered to have nausea as a possible adverse effect.	**Relative importance of treatment, self-reported in a telephone interview:** The positive constant term (β = 1.23, 95% CI 0.81, 1.66, p<0.001) suggests that respondents preferred drug treatment over no drug treatment when all other attributes were set to zero.<br>For bisphosphonates, respondents were willing to pay up to ~ 338 euro to receive treatment compared with no treatment. They would thus be willing to pay for this treatment if the fracture risk reduction was at least 12%.<br><br>**Preference for the current drug profile:** The positive utility value of the specific drug profile (utility = 0.46) indicates a preference for this drug treatment over no treatment.<br><br>**Subgroups:**<br>Lower levels of treatment effectiveness were more acceptable to high-risk patients than to low-risk patients (p = 0.05)
Fuzzell 2020 [2], USA<br><br>Cross-sectional<br><br>Treatment-naïve participants provided information and then interviewed with open-ended and survey questions.	n=30 (46% of eligible), females ≥65 years who had never been offered and had never taken bisphosphonates (BPs) recruited from research participant lists<br><br>**Age, mean (SD):** 72.7 (4.8)<br>**Menopausal status:** NR<br>**BMD:** NR<br>**Prior fracture:** NR<br>**Osteoporosis dx:** NR (100% treatment naïve)<br>**Medication use:** NR<br>**Concern about fractures:** NR<br>**Perceived severity of fractures:** NR<br>**Absolute fracture risk:** NR<br>**Perceived fracture risk:** NR<br>**Previous screening:** NR	**Format:** Information was textual and visual (icon arrays) on risk of outcomes for women 1 year after hip fracture, risk of further bone loss for women taking BPs, risk of fracture for women who do and do not take BPs.<br><br>**Knowledge of risk:** Actual risk for fracture/bone mineral density t-score/indication for BP therapy was not criteria for eligibility and was not collected from participants.<br><br>**Benefits of treatment**: Lowers chance of breaking a bone (by about half), 20 in 100 women with osteoporosis break a bone without taking medication, and only 10 in 100 who take this medication break a bone. Lowers chance of forward curve of the spine (kyphosis), disability, and loss of independence.<br><br>**Harms of treatment:** This medication has very rare side effects such as: A problem with the jawbone, where the lower or upper jaw is exposed. This happens in 1 in 10,000 to 1 in 100,000 people.	**Acceptors (of treatment) and cautious acceptors (accept but little worried about it) of BPs:** 17/30 (56.6%)<br><br>Many participants' responses indicated they were worried about osteoporosis overall and were willing to take medication to treat it, but were unwilling to take BPs in particular because of concerns about side effects. Eg 80% would be willing to take a medication<br><br>**Subgroups:** None

Author & Year, Country<br>Design<br>Study description	Participant characteristics	Format of information<br>Knowledge of risk<br>Information provided on benefits and harms	Outcomes of interest<br>Subgroup data
		An unusual break of the thigh bone. This happens in about 1 in 10,000 people. If asked, women would be told "Some people talk about stomach problems, but research found that people taking the medicine do not have stomach problems more than people taking a placebo (or sugar pill).	
Hudson 2011 [3], New Zealand<br><br>RCT (4-arm)<br><br>Participants were communicated information on the benefits and harms of a hypothetical treatment using absolute or relative values.	n = 393 women (34% of eligible) ≥50 years enrolled as a patient of one of 10 GPs at 4 practices in Christchurch.<br><br>**Age, mean (SD):** 63.1 (8.7) y<br>**Menopausal status:** NR<br>**BMD:** NR<br>**Prior fracture:** 57 (14.5%)<br>**Osteoporosis dx:** 17 (4.3%)<br>**Medication use:** NR<br>**Concern about fractures:** NR<br>**Perceived severity of fractures:** NR<br>**Absolute 10-year hip fracture risk (FRAX), median (IQR):** 2.2 (0.5-2.7)%<br>**Perceived fracture risk:** 321 (81.7%) believed they were unlikely to sustain a fracture<br>**Previous screening:** NR	**Format:** Participants received information on a hypothetical treatment in one of four groups:<br>– benefits and harms both described pictorially in absolute terms;<br>– benefits described pictorially in absolute terms, harms described in relative terms;<br>– benefits described in relative terms, harms described pictorially in absolute terms;<br>– benefits and harms both described in relative terms<br><br>**Knowledge of risk:** 10-year hip fracture risk calculated using FRAX.<br><br>**Benefits of treatment:** relative reduction in risk of hip fracture by 40%, or presented with a chart of 1000 women showing the number expected to have a hip fracture in 10 years and the number avoided by taking treatment (varied from 1-200/1000 according to individual fracture risk).<br><br>**Harms of treatment:** relative increase in risk of stroke by 67%, or presented with a chart of 1000 women showing the number of women expected to have a stroke in the next 10 years without treatment (12 per 1000) and the additional strokes with treatment (8 per 1000).	**Acceptance of treatment after absolute vs. relative presentation of benefits self-reported on a 4-point scale (very likely, quite likely, quite unlikely, very unlikely):** 82 (43%) vs. 71 (36%) likely, 110 (57%) vs. 129 (65%) unlikely, OR 1.73 (95% CI 1.10-2.73), p=0.018 adjusted for age, previous dx of osteoporosis, education, self-reported risk.<br><br>**Among those accepting treatment after presentation of benefits (n = 153), likelihood of still accepted after absolute vs. relative presentation of harms:** 32 (46%) vs. 12 (14%) likely, 38 (25%) vs. 71 (50%) unlikely, OR 4.89 (95% CI 2.30-11.0), p<0.001.<br><br>**Subgroups:**<br>– Predictors of acceptance of treatment after presentation of benefit included age (per decade after 10 years) (OR 1.4, 95% CI 1.05-1.78), previous diagnosis of osteoporosis (OR 5.4, 95% CI 1.52-26.12), self-reported risk (vs. very likely) (OR 1.8, 95% CI 1.1-3.0 for quite unlikely; OR 1.9, 95% CI 1.01-3.65 for quite/very likely). There was no significant effect of history of fracture, absolute 10-year hip fracture risk, or BMD.<br>– After presentation of benefits and harms, only higher self-reported risk (OR 2.7, 95% CI 1.3-5.5) and absolute presentation of harms (OR 3.8, 95% CI 1.9-8.1) increased the likelihood of accepting treatment.
Hudson 2012 [4], New Zealand<br><br>Cross-sectional<br><br>Participants completed a questionnaire about their	n = 354 (36% of eligible) patients aged 50-70 years (44% female) who were registered with 3 GPs in Christchurch<br><br>**Age, mean (SD):** 59.7 (5.7) y<br>**Menopausal status:** NR<br>**BMD:** NR<br>**Prior fracture:** NR	**Format:** Participants completed a mailed questionnaire in which they were presented with a scenario of 5,000 people aged 50-70 years undergoing treatment with alendronate or other bisphosphonates for 10 years. Participants were asked to select the number of hip fractures that they considered justified accepting treatment from 1, 5, 50, 100, 500, or 1000.	**Minimum acceptable benefit of the medication self-reported on a questionnaire:** 227 (64%) chose a minimum acceptable benefit that was greater than the actual benefit of medication (>50 hip fractures prevented), 56 (16%) matched the actual benefit, and 71 (20%) were lower than the actual benefit (<50 hip fractures prevented).

Author & Year, Country<br>Design<br>Study description	Participant characteristics	Format of information<br>Knowledge of risk<br>Information provided on benefits and harms	Outcomes of interest<br>Subgroup data
expectations of the benefits of four treatment options.	**Osteoporosis dx:** 33 (9%)<br>**Medication use:** 35 (10%)<br>**Concern about fractures:** NR<br>**Perceived severity of fractures:** NR<br>**Absolute fracture risk:** NR<br>**Perceived fracture risk:** NR<br>**Previous screening:** NR	**Knowledge of risk:** Participants were not provided with information on their individual risk, but knew if they had osteoporosis.<br><br>**Benefits of treatment:** Not provided. The authors considered 50 hip fractures avoided to be the correct answer, based on a 60-year old woman with a 10-year hip fracture risk of 2.3% and a 53% relative risk reduction with alendronate.<br><br>**Harms of treatment:** "This medication has no major side effects"	**Subgroups:**<br>Age, sex, past diagnosis of osteoporosis, and use of medications for osteoporosis were not significant predictors of overestimating the minimum acceptable benefit
Kalluru 2017 [5], New Zealand<br><br>RCT (4-arm)<br><br>Participants read text about the benefits of treatment in various ways (having or not having an event; natural frequencies or number needed to treat).	n = 200 (91% of eligible) patients >60 years (81% female) who had been referred to a public hospital clinic for bone density measurement, but were not taking any anti-osteoporosis treatments<br><br>**Age, mean:** 69 y<br>**Menopausal status:** NR<br>**BMD, femoral neck T-score:** mean across groups was in the osteopenic range<br>**Prior fracture:** 66 (33%)<br>**Osteoporosis dx:** NR<br>**Medication use:** no current use<br>**Concern about fractures:** NR<br>**Perceived severity of fractures:** NR<br>**Absolute 5-year osteoporotic fracture risk (Garvan), median (IQR):** 7.4 (5.5, 12.0)%<br>**Absolute 5-year hip fracture risk (Garvan), median (IQR):** 1.4 (0.8, 3.0)%<br>**Perceived 5-y osteoporotic fracture risk, median (IQR):** 20 (10, 50)%; estimates were 2-3 times higher than calculator<br>**Perceived 5-y hip fracture risk, median (IQR):** 19 (10, 40)%; estimates were 10-20 times higher than calculator<br>**Previous screening:** NR	**Format:** Participants were randomized to one of 4 arms which differed in their framing of the benefits and risk of a hypothetical treatment that reduces osteoporotic fractures by 33%.<br><br>**Knowledge of risk:** All participants were provided their 5-year risk of osteoporotic and hip fracture using Garvan + BMD<br><br>**Benefits of treatment:** Osteoporosis medication reduces osteoporotic fractures by 33%, and hip fractures by 40%. Framed either as the:<br>(1) chance of having an event and benefits in natural frequencies (out of 100 people, the number having an osteoporotic fracture would decrease from 20 to 13, hip fracture from 5 to 3);<br>(2) chance of not having an event in natural frequencies (out of 100 people, the number not having an osteoporotic fracture would increase from 80 to 87, hip fracture from 95 to 97);<br>(3) chance of having an event in number needed to treat (15 people would need to be treated to prevent one osteoporotic fracture, 50 would need to be treated to prevent one hip fracture);<br>(4) chance of not having an event in number needed to treat (if 15 people were treated 14 would receive no benefit in terms of osteoporotic fracture prevention and in 1 person a fracture would be prevented; if 50 people were treated 49 would receive no benefit in terms of hip fracture prevention and 1 hip fracture would be prevented).<br><br>**Harms of treatment:** Not provided.	**Perceived level of risk of osteoporotic fracture and hip fracture at which treatment would be considered self-reported on a questionnaire:** at baseline, the median (IQR) 5-y risk threshold for oral tablets was 50 (25, 70)% for osteoporotic fracture and 50 (30, 75)% for hip fracture. The threshold for intravenous medications was 60 (30, 80)% for osteoporotic fracture and 60 (40, 80)% for hip fracture. Receiving information on benefits led to no or very small changes in risk thresholds (decrease of ≤10%).<br><br>**Proportion believing that they should take osteoporosis medication:** At baseline, 30 (15%) said yes, 67 (34%) said no, 101 (51%) were unsure. After receiving information, of those originally saying yes, 67% still said yes, 27% said no, and 7% were unsure. Of those originally saying no, 4% said yes, 81% still said no, and 15% were unsure. Of those originally being unsure, 12% said yes, 39% said no, and 48% were still unsure. This means that after receiving information, 37 (18.5%) said yes, 101 (51%) said no, and 60 (30%) were unsure. At 3 months follow-up, 53 (27%) actually started or intended to start medication, while 122 (61%) did not.<br>At baseline, 46% of participants estimated their hip or total fracture risk was equal or greater than one of the thresholds they considered high enough for treatment. This decreased to 37% after they received information.<br><br>**Subgroups:**

Author & Year, Country<br>Design<br>Study description	Participant characteristics	Format of information<br>Knowledge of risk<br>Information provided on benefits and harms	Outcomes of interest<br>Subgroup data
			There were no between-group differences in perceived level of risk at which treatment would be considered (p < 0.6) at baseline or after the receipt of information in varied formats.
LeBlanc 2015 [6], USA<br><br>Prospective cohort (one arm of a RCT)<br><br>Clinicians engaged patients in shared decision making about starting bisphosphonates using the Osteoporosis Choice decision aid.	n = 32 women >50 years with a diagnosis of osteoporosis or osteopenia who were identified by their clinician as potentially eligible for bisphosphonates and had an upcoming BMD evaluation at participating primary care practices affiliated with the Mayo Clinic, Rochester, Minnesota. Women were part of a RCT where 95% of those eligible were enrolled.<br><br>**Age, mean (SD):** 69 (8) y<br>**Menopausal status:** NR<br>**BMD:** NR<br>**Prior fracture:** NR<br>**Osteoporosis dx:** All diagnosed with osteopenia or osteoporosis<br>**Medication use:** no current use.<br>**Concern about fractures:** NR<br>**Perceived severity of fractures:** NR<br>**Absolute fracture risk (FRAX), mean (SD):** 14 (8)%; 10 (31%) had a risk <10%, 16 (50%) had a risk 11-20%, and 6 (19%) had a risk >20%<br>**Perceived fracture risk:** NR<br>**Previous screening:** NR	**Format:** The Osteoporosis Choice decision aid was used by the clinician during the clinical encounter. Patients and clinicians were to review the decision aid, deliberate about whether to start bisphosphonates, and make a decision together at that time or at a later time. https://shareddecisions.mayoclinic.org/decision-aid-information/decision-aids-for-chronic-disease/other-decision-aids/<br><br>**Knowledge of risk:** Participants were provided their 10-year risk of MOF using FRAX. After the encounter, 20 (69%) correctly identified their risk without treatment, and 23 (79%) correctly identified their risk with treatment. Median (IQR) osteoporosis knowledge score (13 items, higher = more knowledge) was 7.0 (4.5, 9.0).<br><br>**Benefits of treatment:** Absolute risk reduction with bisphosphonates represented using an evidence-based pictograph and assuming a treatment-related reduction in overall fractures of 40%. For example: "Roughly 24 in 100 have a fracture within the next years. 76 will not. 16 have avoided a fracture because of the medication." (patients fill in the numbers)<br><br>**Harms of treatment:** "Abdominal problems: About 1 in 4 people will have heartburn, nausea, or belly pain. However, it may not be from medication. If the medication is the cause, the problem will go away if you stop taking it. Osteonecrosis of the jaw: If 10,000 patients are treated, we would expect fewer than 1 to have bone sores that may be painful or need surgery. For comparison, if 10,000 patients who have a tooth extracted are treated, we would expect fewer than 30 to have bone sores of the jaw that may be painful or need surgery."	**Decision to start medication reported on a survey and verified using pharmacy records:** 12 (41%) of patients decided to start taking a bisphosphonate and 10 of these (83%) decided to fill that prescription. Eight (28%) decided not to start bisphosphonates and 9 (32%) were undecided.<br><br>**Subgroups:** none
Liu 2020 [7] & Billington 2019 [8], Canada<br><br>Liu: Cohort study; Billington: Cross-sectional (smaller than Liu with same patients but additional	Liu: n= 208 females (group 1 n=125; group 2 n=85)<br>Billington: n = 85 females (overlap with Liu in group 1) ≥45 years referred by a primary care provider for age-associated osteoporosis.<br><br>Liu (whole sample unless otherwise specified):<br>**Age, median (IQR):** 63.5 (NR) y	**Format:**<br>Group 1: The group self-management program included education about osteoporosis, consequences of fragility fracture, fracture risk factors, and detailed benefits and risks of various pharmacologic treatments (raloxifene, alendronate, risedronate, zoledronic acid, denosumab, teriparatide). Prior to the self-management program, patients attended a 2-h teaching session on the basics of osteoporosis	**Plan to initiate therapy, decline therapy, or remain undecided self-reported on a questionnaire:** 20.2% chose to initiate pharmacologic therapy<br><br>**Subgroups:**

Author & Year, Country<br>Design<br>Study description	Participant characteristics	Format of information<br>Knowledge of risk<br>Information provided on benefits and harms	Outcomes of interest<br>Subgroup data
subgroup data based on risk for hip fracture)<br><br>Liu:<br>Group 1: Group (5-10 patients) self-management consult program aimed at facilitating decision-making about treatment for osteoporosis.<br>Group 2: Traditional one-on-one consultation with specialist.<br>(Combined for analysis in this review)<br><br>Billington: same as group 1 above.	**Menopausal status:** NR<br>**BMD** T-score at femoral neck, median (IQR): group 1: -1.9 (-2.3, -1.3); group 2: -1.5 (-2.2, -0.9)<br>**Prior fracture:** 64 (30.8%)<br>**Osteoporosis dx:** 100%<br>**Medication use:** 79 (38%) prior use<br>**Concern about fractures:** 36 (42%) were worried about their fracture risk (only reported in Billington)<br>**Perceived severity of fractures:** NR<br>**Absolute 10-year risk (FRAX), median (IQR):** group 1: 11.5 (8.6, 18.2)% MOF, 24 (19%) had a risk ≥20%; 2.1 (1.1, 4.5)% hip, (in Billington) 31 (37%) had a risk ≥3%; group 2: 11.5 (7.9, 16.3)% MOF, 16 (19%) had a risk ≥20%; 2.3 (1.0, 4.0)% hip<br>**Perceived fracture risk:** NR<br>**Previous screening:** NR	and lifestyle interventions such that the total teaching and decision-making process includes >4 hours of instruction and interaction.<br>Group 2: Traditional one-on-one session with similar information to group sessions and a shared decision making approach.<br><br>**Benefits of treatment:** individual absolute fracture risk reduction calculated assuming a 40% relative risk reduction from baseline<br><br>**Harms of treatment:** general information related to each drug of interest, including hot flashes (7-10%), leg cramps, blood clot (1 per 1000 in past 3 years), indigestion, heartburn, nausea (about 10%), osteonecrosis of the jaw (very rare, 1 in 10,000 to 1 in 100,000), atypical fractures of the thigh bone (very rare, 1 in 10,000 to 1 in 100,000), flu-like symptoms (3-4%) lasting 2-3 days, pain, dry skin, skin infection (rare), leg cramps.	– Of those with FRAX 10-y MOF ≥20% 18/40 (45%) accepted therapy vs. 15/85 (17.6%) of those with a moderate risk 10-19.9% vs 9/83 (11%) low risk 0-9.9%<br>– (from Billington) Of those with a 10-year hip fracture risk ≥3% 10/31 (32%) accepted therapy vs. 10/54 (19%) of those with a risk <3%, p=0.012<br>– Of those with prior fracture 26/64 (40.6%) accepted therapy<br>– (from Billington) Median (IQR) femoral neck T-score was -2.6 (-1.9, -2.9) among acceptors, -2.1 (-1.2, -2.5) among decliners, and -2.6 (-2.3, -2.7) among undecided<br><br>Worry about fracture risk was present in 12 (52%) of acceptors, 11 (28%) of decliners, and 12 (57%) of undecided
Montori 2011 [9], USA<br><br>Prospective cohort (one arm of a RCT)<br><br>Clinicians engaged patients in shared decision making about starting bisphosphonates using the Osteoporosis Choice decision aid.	n = 52 (100% of eligible) postmenopausal women ≥50 years who were patients of 10 general medicine and primary care practices affiliated with the Mayo Clinic, Rochester, Minnesota. Women had BMD levels consistent with a diagnosis of osteopenia or osteoporosis and were found by their clinician to be eligible for bisphosphonate therapy, but were not already taking prescription anti-osteoporosis medications.<br><br>**Age, median (range):** 67 (51-84) y<br>**Menopausal status:** all postmenopausal<br>**BMD T-score at left femoral neck, median (range):** -1.80 (-3.7 to -0.7)<br>**Prior fracture:** 23 (44%)<br>**Osteoporosis dx:** all had osteoporosis or osteopenia<br>**Medication use:** none (exclusion criteria)<br>**Concern about fractures:** NR<br>**Perceived severity of fractures:** NR	**Format:** The Osteoporosis Choice decision aid was used by the clinician during the clinical encounter. Patients and clinicians were to review the decision aid, deliberate about whether to start oral alendronate, and make a decision together at that time or at a later time. https://shareddecisions.mayoclinic.org/decision-aid-information/decision-aids-for-chronic-disease/other-decision-aids/<br><br>**Knowledge of risk:** Participants were provided their 10-year risk of MOF using FRAX, and were categorized into one of three arbitrary categories: <10%, 10-30%, or >30% risk.<br><br>**Benefits of treatment:** Absolute risk reduction with alendronate shown on a pictograph, assuming a reduction in overall fracture risk of 40%. For example: "Roughly 24 in 100 have a fracture within the next years. 76 will not. 16 have avoided a fracture because of the medication."	**Decision to start medication reported on a survey and verified after 6 months using pharmacy records:** 23 (44%) of patients decided to start bisphosphonates, and all of these patients had prescriptions for bisphosphonates in the pharmacy data.<br><br>**Subgroups:**<br>– 1/2 (50%) in the low risk, 18/40 (45%) in the moderate risk, and 4/10 (40%) in the high risk group started bisphosphonates

Author & Year, Country<br>Design<br>Study description	Participant characteristics	Format of information<br>Knowledge of risk<br>Information provided on benefits and harms	Outcomes of interest<br>Subgroup data
	**Absolute 10-y MOF risk (FRAX), median (range):** 19 (6.1 to 39)%<br>**Absolute 10-y hip fracture risk (FRAX), median (range):** 2.05 (0.6 to 18)%<br>**Perceived fracture risk:** NR<br>**Previous screening:** NR	**Harms of treatment:** "Abdominal problems: About 1 in 4 people will have heartburn, nausea, or belly pain. However, it may not be from medication. If the medication is the cause, the problem will go away if you stop taking it. Osteonecrosis of the jaw: Fewer than 1 in 10,000 (over next 10 y) will have bone sores of the jaw that may need surgery).	
Neuner 2014 [10], USA<br><br>Cross-sectional<br><br>Participants were provided information regarding fracture risks and treatment risks and benefits, followed by a series of vignettes depicting a 70-year-old woman at baseline fracture risks between 5–50%.	n = 241 (31% of eligible) women ≥60 years randomly selected from those seen in the past 12 months at three general internal medicine practices in Milwaukee, Wisconsin. Inclusion criteria were designed to target postmenopausal women likely to be faced with fracture preventive treatment decision-making.<br><br>**Age, mean (SD):** 69.4 (7.19) y<br>**Menopausal status:** all postmenopausal<br>**BMD:** NR<br>**Prior fracture:** 82 (34%) any fracture, 7 (3%) hip fracture after age 40 y<br>**Osteoporosis dx:** 215 (89%) had a prior bone density test and of these 65 (27%) had osteoporosis and 75 (31%) had osteopenia<br>**Medication use:** Appear to be untreated (inclusion criteria)<br>**Concern about fractures:** NR<br>**Perceived severity of fractures:** NR<br>**Absolute fracture risk:** NR. 63% had at least one major risk factor other than low bone density.<br>**Perceived fracture risk:** Those with osteoporosis estimated their 10-y fracture risk to be 43%, those without osteoporosis estimated it at 37%. Women estimated their lifetime mean (SD) fracture risk at 50 (33)%.<br>**Previous screening:** NR	**Format:** Each hypothetical vignette asked the subject to imagine that she was a 70-year-old woman whose risk of a broken hip in the next 10 years was n% and risk of other fractures was 4n%.<br><br>**Knowledge of risk:** Participants provided their 10-year fracture risk estimate using the FRACTURE Index.<br><br>**Benefits of treatment:** The effect of treatment was summarized with the statement, "if you take an osteoporosis medication once weekly, you can reduce your chance of breaking a bone" and was also depicted using 2 pictographs. Each pictograph showed 100 women and their fracture outcomes in next 10 years. The first pictograph showed the risk with no medication and the second the risk with medication. A 33% fracture risk reduction with treatment was assumed. The vignettes were displayed in order of increasing risk (1% hip/4% other, 2%/8%, 3%/12%, 6%/24%, 8%/32%, and 10%/40%). Vignette 3 represents the current treatment threshold.<br><br>**Harms of treatment:** Provided in a box next to the pictograph - stomach upset severe enough to stop therapy (5 or more out of 100 people), osteonecrosis of the jaw (1/100,000), and atrial fibrillation (1/100).	**Willingness to take medication was self-reported immediately after reading the vignettes:** Willingness to accept treatment increased with increasing level of risk. 43% accepted at 5% risk, 45% at 10% risk, 51% at 15% risk (current treatment threshold), 66% at 30% risk, 77% at 40% risk, 82% at 50% risk.<br><br>**Subgroups:**<br>– Odds of accepting treatment increased marginally with increasing 10-year fracture risk for vignette 3 only, OR 1.02, 95% CI 1.01 to 1.03. The relationship was not significant for other vignettes.<br>– Odds of accepting treatment was higher for those with vs. without osteoporosis in vignettes 1-3, but not others. Vignette 1: 64.2% with vs. 37.5% without osteoporosis would accept, OR 2.66, 95% CI 1.35 to 5.25.<br>Vignette 2: 62.3% vs. 40.2%, OR 2.23, 95% CI 1.14 to 4.36<br>Vignette 3: 71.7% vs. 45.1%, OR 2.9, 95% CI 1.4 to 5.9
Sheridan 2016 [11], USA<br><br>RCT (4-arm)	n = 258 women (% of eligible NR, but 24% of eligible cohort participated in any of the screening services; osteoporosis was a subgroup) aged 50-64 years, with a BMI ≥18, no history of fracture or family history of osteoporosis, no current use of prednisone (>30	**Format:** Participants were provided one of four 1-page written evidence-based support sheets for osteoporosis screening using BMD and treatment with bisphosphonates (such as alendronate).	**Mean (SD) intention to accept screening during the usually recommended screening interval (5 years) self-reported before and immediately after reading the information sheet (range 1-5, where higher scores indicate stronger intentions):**

Author & Year, Country<br>Design<br>Study description	Participant characteristics	Format of information<br>Knowledge of risk<br>Information provided on benefits and harms	Outcomes of interest<br>Subgroup data
Participants were presented with information on benefits and harms of screening (and associated treatment) in one of four formats: words, numbers, numbers + narrative, numbers + framed presentation	consecutive days), <3 drinks per day, nonsmokers. Women were eligible if they had an upcoming visit, and were eligible to receive information on osteoporosis screening at one of four community-based practices affiliated with the Duke Primary Care Research Consortium.<br><br>**Age, mean:** 57 y<br>**Menopausal status:** NR<br>**BMD:** NR<br>**Prior fracture:** none (exclusion criteria)<br>**Osteoporosis dx:** NR<br>**Medication use:** NR<br>**Concern about fractures:** NR<br>**Perceived severity of fractures:** NR<br>**Absolute fracture risk:** NR<br>**Perceived fracture risk:** After screening, mean (SD) risk (range 1-4 where higher scores indicate higher risk) ranged from 1.76 (0.82) to 2.27 (1.00) across groups.<br>**Previous screening:** 146 (57%)	**Knowledge of risk:** Knowledge of risk not applicable, because patients have not been screened. After reading the information, mean (SD) disease specific knowledge (range 0-2, where higher scores indicate greater knowledge) ranged from 1.13 (0.72) to 1.20 (0.59) across groups. Mean (SD) general screening knowledge (range 0-8, where higher scores indicate greater knowledge) ranged from 5.33 (1.93) to 5.74 (1.76) across groups.<br><br>**Benefits of screening and treatment:**<br>(1) Words: finding and treating osteoporosis early reduces broken hip bones in very few of the women who are screened and treated and reduces the chances of other broken bones in a few.<br>(2) Numbers: finding and treating osteoporosis early reduces broken hip bones in 2 per 1000 screened and treated over 10 years (7 per 1000 to 5 per 1000) and reduces the chances of other broken bones.<br>(3) Numbers + narrative: same as presentation of numbers, but with added narrative from women and photographs.<br>(4) Numbers + framed: same as presentation of numbers for benefits<br><br>**Harms of screening and treatment:** Finding out about osteoporosis might lead some women to worry about a broken bone. Experts are unsure how many women worry. Bisphosphonates may cause minor stomach upset in not taken according to instructions. They may also cause muscle and joint pains in some people. Most serious symptoms are rare. Overdiagnosis presented by showing that incident disease rates exceed important outcomes: "It affects 45 of every 1000 women you age. It increases the changes of broken bones, particularly in the hip and spine. In the next 10 years, about 7 of every 1000 women your age will have a broken hip."<br><br>(1) Words: Over 10 years very few women will have damage to the jaw (osteonecrosis) or atypical breaks of the bone.<br>(2) Numbers: Of every 1000 women treated over 10 years 1 to 10 will have damage to the jaw (osteonecrosis) and 5 will have atypical breaks of the bone.<br>(3) Numbers + narrative: same as presentation of numbers, but with added narrative from women and photographs.	Words: 3.64 (1.08) vs. 3.38 (1.16); MD -0.23 (-0.40, -0.06), p < 0.001<br>Numbers: 3.73 (1.08) vs. 3.73 (1.10); MD 0.02 (-0.15, 0.19), ns<br>Narrative: 3.88 (0.69) vs. 3.82 (0.85); MD -0.06 (-0.23, 0.11), ns<br>Framed: 3.69 (0.92) vs. 3.67 (1.18); MD -0.05 (-0.21, 0.12), ns<br><br>**Subgroups:**<br>– There was no difference in the change in intention to accept screening between groups (p = 0.19)<br>– Change in intention to accept screening did not differ by subgroups of patients defined by previous screening or worry about health

Author & Year, Country<br>Design<br>Study description	Participant characteristics	Format of information<br>Knowledge of risk<br>Information provided on benefits and harms	Outcomes of interest<br>Subgroup data
		(4) Numbers + framed: same as presentation of numbers, but framed as benefits of NOT being screened (e.g., avoid unnecessary treatments and side effects).	
Si 2019 [12], China<br><br>Cross-sectional<br><br>Discrete choice experiment using hypothetical drug treatment profiles and four treatment attributes: effectiveness, adverse effects, out of pocket costs, mode of administration.	n = 267 (% of eligible NR) patients (81% female) who attended the department of Rheumatology of the Third Affiliated Hospital of Sun Yat-sen University and were assessed by their clinician to be at risk for osteoporotic fracture.<br><br>**Age, mean (SD):** 63.4 (10.2) y<br>**Menopausal status:** NR<br>**BMD T-score, mean (SD):** -2.1 (0.8)<br>**Prior fracture:** 66 (23%)<br>**Osteoporosis dx:** 119 (42%) self-reported and 88 (31%) with osteoporosis defined by T-score<br>**Medication use:** NR<br>**Concern about fractures:** NR<br>**Perceived severity of fractures:** NR<br>**Absolute fracture risk:** NR<br>**Perceived fracture risk:** NR<br>**Previous screening:** NR	**Format:** Participants completed a discrete choice experiment where they chose between sets of two different treatment profiles, with the option of no treatment. Each treatment profile had different levels of four attributes: effectiveness, adverse effects, out of pocket costs, mode of administration. The attributes were based on the characteristics of alendronate, zoledronic acid, raloxifene, calcitonin, denosumab, and calcium/vitamin D.<br><br>**Knowledge of risk:** Participants were not provided with information on their individual risk, but knew if they had osteoporosis.<br><br>**Benefits of treatment:** Treatment efficacy in reducing the risk of fracture could be 20%, 30%, 40%, or 50%.<br><br>**Harms of treatment:** could be one of flu-like symptoms, skin reactions, gastrointestinal disorders - these were assumed to occur in 1 of every 50 patients undergoing treatment. Each of these effects was relatively mild, disappeared after a few days, and had no long-term or severe consequences.	**Relative importance of treatment, self-reported on a questionnaire:** The positive constant term (ASC = 9.57, 95% CI 7.51, 11.63) indicates that on average patients preferred to received treatment over no treatment. Patients significantly preferred a treatment with higher clinical efficacy. The SD of the constant was statistically significant, indicated the presence of significant preference heterogeneity for treatment.<br>Patients were willing to pay 3689 Yuan (5th and 95th percentiles 2037 and 6532 Yuan, respectively) more per annum for a 1% improvement in medication efficacy of preventing fractures.<br><br>**Subgroups:**<br>– Patients who were women and those with osteoporosis had a stronger preference for receiving osteoporosis medication ($p < 0.05$).<br>– There was no significant difference in preference for treatment by presence of prior fracture.
Smallwood 2017 [13], USA<br><br>Prospective cohort<br><br>Patients engaged with an online decision aid about treatment for osteoporosis containing a summary of medication risks and benefits	n = NR (<33) women ≥55 years (to ensure postmenopausal status) who had undergone screening at one of three primary care clinics within a Midwestern multispecialty academic group practice and were found to have a T-score of ≤-1. Women were recruited through a patient portal or by mailed invitation and were part of a RCT where 82% of those eligible were enrolled.<br><br>**Age, mean:** 68.8 y<br>**Menopausal status:** All postmenopausal<br>**BMD:** All had a T-score of ≤-1<br>**Prior fracture:** 13 (44.8%)<br>**Osteoporosis dx:** 16 (55.2%) had osteopenia and 13 (44.8%) had osteoporosis<br>**Medication use:** No past or current use of bisphosphonates (subgroup of the population)	**Format:** Patients accessed an online decision aid titled 'Healthy Bones' from the Agency for Healthcare Research and Quality (no longer appears to be available online – broken link), which was adapted to include a personalized fracture risk (FRAX-BMD) calculator and information about osteoporosis including cases, risk factors, 'how to determine if you have osteoporosis', details about prescription and non-prescription treatment, and a values elicitation exercise. Aside from medication, recommendations for getting more dietary calcium and the types of exercise that is beneficial for bones was also included.<br><br>**Knowledge of risk:** The tool enabled patients to calculate their 10-y MOF risk using FRAX-BMD. Knowledge score (about osteoporosis) was 74% at baseline, 84% post-intervention, and 82% at 3 months.	**Proportion of patients taking anti-osteoporosis medications at 6 months ascertained using a chart review:** 5.3%<br><br>**Subgroups:** None

Author & Year, Country<br>Design<br>Study description	Participant characteristics	Format of information<br>Knowledge of risk<br>Information provided on benefits and harms	Outcomes of interest<br>Subgroup data
	**Concern about fractures:** NR<br>**Perceived severity of fractures:** NR<br>**Absolute fracture risk:** NR; 89% had at least one fracture risk factor other than age and low BMD<br>**Perceived fracture risk:** NR<br>**Previous screening:** All had been screened using BMD<br><br>**Note: above data are for the entire cohort as participant characteristics are not available for the subgroup of interest (untreated patients)	**Benefits of treatment:** A medication table included information on evidence available for 7 different medications, as follows:<br>– Alendronate, risedronate, denosumab: some protection against hip, back, and other fractures<br>– Ibandronate: some protection against back fractures, unknown for hip and other fractures<br>– Teriparatide: some protection against back and other fractures, unknown for hip fractures<br>– Raloxifene: some protection against back fractures but not for hip or other fractures<br><br>**Harms of treatment:** Information provided to patients but NR	

ASC=alternative specific constant; BMD=bone mineral density; CI=confidence interval; IQR=interquartile range; MOF=major osteoporotic fracture; NR=not reported; OR=odds ratio; RCT=randomized controlled trial; SD=standard deviation; USA=United States of America; y=years

REFERENCES

1. de Bekker-Grob EW, Essink-Bot ML, Meerding WJ, Pols HA, Koes BW, Steyerberg EW. Patients' preferences for osteoporosis drug treatment: a discrete choice experiment. Osteoporos Int. 2008;19(7):1029-37.
2. Fuzell LN, Fraenkel L, Stark SL, Seehra SS, Nelson C, Keleman A, et al. A mixed methods study exploring older womens' attitudes toward osteoporosis medications: adapting a health communication framework. Women's Health Reports. 2020; 1:102-13.
3. Hudson B, Toop L, Mangin D, Pearson J. Risk communication methods in hip fracture prevention: a randomised trial in primary care. Brit J Gen Pract. 2011;61(589):e469-76.
4. Hudson B, Zarifeb A, Young L, Wells J. Patients' expectations of screening and preventive treatments. Ann Fam Med. 2012;10(6):495-502.
5. Kalluru R, Petrie KJ, Grey A, Nisa Z, Horne AM, Gamble GD, et al. Randomised trial assessing the impact of framing of fracture risk and osteoporosis treatment benefits in patients undergoing bone densitometry. BMJ Open. 2017;7(2):e013703.
6. LeBlanc A, Wang AT, Wyatt K, Branda ME, Shah ND, Van Houten H, et al. Encounter decision aid vs. clinical decision support or usual care to support patient-centered treatment decisions in osteoporosis: the Osteoporosis Choice randomized trial II. PLoS ONE. 2015;10(5):e0128063.
7. Liu CS, Feasel AL, Kline GA, Billington EO. Pharmacotherapy decisions among postmenopausal women attending a group medical consultation or a one-on-one specialist consultation at an osteoporosis center: an observational cohort study. Osteoporos Int. 2021;32:1421–27.
8. Billington EO, Feasel AL, Kline GA. At odds about the odds: women's choices to accept osteoporosis medications do not closely agree with physician-set treatment thresholds. J Gen Intern Med. 2019;35(1):275-282.
9. Montori VM, Shah ND, Pencille LJ, Branda ME, Van Houten HK, Swiglo BA, et al. Use of a decision aid to improve treatment decisions in osteoporosis: the osteoporosis choice randomized trial. Am J Med. 2011;124(6):549-56.
10. Neuner JM, Schapira MM. Patient perceptions of osteoporosis treatment thresholds. J Rheumatol. 2014;41(3):516-22.
11. Sheridan SL, Sutkowi-Hemstreet A, Barclay C, Brewer NT, Dolor RJ, Gizlice Z, et al. A comparative effectiveness trial of alternate formats for presenting benefits and harms information for low-value screening services: a randomized clinical trial. JAMA Intern Med. 2016;176(1):31-41.
12. Si L, Tu L, Xie Y, Palmer AJ, Gu Y, Zheng X, et al. Chinese patients' preference for pharmaceutical treatments of osteoporosis: a discrete choice experiment. Arch Osteoporos. 2019;14(1):85.
13. Smallwood AJ, Schapira MM, Fedders M, Neuner JM. A pilot randomized controlled trial of a decision aid with tailored fracture risk tool delivered via a patient portal. Osteoporos Int. 2017;28(2):567-76.
