# Supplementary material for: Screening for the primary prevention of fragility fractures among adults aged 40 years and older in primary care: systematic reviews of the effects and acceptability of screening and treatment, and the accuracy of risk prediction tools
Source: Syst Rev. 2023 Mar 21;12:51. doi: 10.1186/s13643-023-02181-w (PMC10029308; doi:10.1186/s13643-023-02181-w)
Supplement: Supplementary file 7 — Additional file 7. KQ 2-4 Risk of bias assessments. This file shows the risk of bias/quality appraisals for key questions 2 (accuracy of screening tests), 3a (benefits of treatment), 3b (harms of treatment), and 4 (acceptability of screening/treatment). [file 13643_2023_2181_MOESM7_ESM.pdf]

Screening for the primary prevention of fragility fractures among adults aged 40 years and older in primary care: systematic reviews of the effects and acceptability of screening and treatment, and the accuracy of risk prediction tools

Michelle Gates, Jennifer Pillay (corresponding), Megan Nuspl, Aireen Wingert, Ben Vandermeer, Lisa Hartling

Affiliation: Alberta Research Centre for Health Evidence, Department of Pediatrics, University of Alberta. Edmonton Clinic Health Academy, 11405-87 Avenue NW, Edmonton, Alberta, Canada T6G 1C9. E-mail: MG: michellegates85@gmail.com; JP: jpillay@ualberta.ca; MN: megan.nuspl@ualberta.ca; AW: awingert@ualberta.ca; BV: ben.vandermeer@ualberta.ca; LH: hartling@ualberta.ca

Additional file 7. Risk of bias assessments

Additional Table 7.1. Risk of bias assessments for studies included in KQ2 on the predictive accuracy of screening tests

Study	Tool	Domain 1. Participants				Domain 2. Predictors							Domain 3. Outcomes									Domain 4. Analysis											Overall Appraisal	
		1.1 Data sources	1.2 Eligibility	Risk of bias	Applicability	2.1 Definitions	2.2 Blinding	2.3a Available	2.3b Collected/used	2.3c Missing data	Risk of bias	Applicability	3.1a Determination (CFF)	3.1b Determination (hip)	3.2 Definition	3.3 Predictors excluded	3.4 Similar definition	3.5 Knowledge of predictor	3.6 Interval	Risk of Bias	Applicability	4.1a No. outcomes (hip)	4.1b No. outcomes (CFF)	4.2 Predictor handling	4.3 Missing participants	4.4 Deaths/competing risk	4.5 Complexities in data	4.6a Performance data (hip)	4.6b Calibration plots (hip)	4.6c Performance data (CFF)	4.6d Calibration plots (CFF)	Risk of Bias	Risk of Bias	Applicability
Azagra 2016a	FRAX	Y	Y	●Low	●High	Y	NI	Y	Y	NI	●Unclear	●Low	Y	Y	Y	Y	Y	NI	Y	●Low	●Low	N	Y	PY	N	N	N	Y	Y	Y	Y	●High	●High	●High
Azagra 2016b	FRAX	Y	Y	●Low	●Low	NI	NI	Y	NI	NI	●Unclear	●Low	Y	NA	Y	Y	Y	NI	Y	●Low	●Low	NA	Y	NI	N	N	N	NA	NA	PY	N	●High	●High	●Low
Bolland 2011	FRAX	Y	Y	●Low	●Low	Y	NI	Y	Y	NI	●Unclear	●Low	N	Y	Y	Y	N	NI	Y	●High	●Low	N	Y	NI	Y	N	N	Y	Y	Y	Y	●High	●High	●Low
	Garvan	Y	Y	●Low	●Low	Y	NI	Y	Y	NI	●Unclear	●Low	N	Y	Y	Y	N	NI	Y	●High	●Low	N	Y	NI	Y	N	N	Y	Y	Y	Y	●High	●High	●Low
Crandall 2019a	FRAX	Y	Y	●Low	●High	N	NI	Y	Y	PY	●Unclear	●Low	PN	Y	Y	Y	Y	NI	Y	●Unclear	●Low	Y	Y	PN	Y	Y	Y	Y	Y	Y	Y	●Unclear	●Unclear	●High
Crandall 2019b	FRAX	Y	Y	●Low	●High	Y	NI	Y	Y	PY	●Unclear	●Low	N	Y	Y	Y	Y	NI	Y	●Unclear	●Low	Y	Y	Y	N	N	N	Y	N	N	N	●High	●High	●High
Crandall 2019b	Garvan	Y	Y	●Low	●High	Y	NI	Y	Y	PY	●Unclear	●Low	N	Y	Y	Y	Y	NI	Y	●Unclear	●Low	Y	Y	Y	N	N	N	Y	N	N	N	●High	●High	●High
Czerwinski 2013	FRAX	PY	Y	●Low	●High	Y	NI	Y	Y	Y	●Low	●Low	N	Y	Y	Y	Y	NI	PY	●High	●Low	N	Y	Y	N	N	N	Y	N	Y	N	●High	●High	●High
Dagan 2017	FRAX	Y	Y	●Low	●Unclear	Y	NI	Y	Y	Y	●Low	●Low	PN	Y	Y	Y	Y	NI	Y	●Unclear	●Low	Y	Y	PN	Y	Y	N	Y	Y	N	N	●High	●High	●Unclear
	Garvan	Y	Y	●Low	●Unclear	Y	NI	Y	Y	Y	●Low	●Low	PN	Y	Y	Y	Y	NI	Y	●Unclear	●Low	Y	NA	PN	Y	Y	N	Y	Y	NA	NA	●High	●High	●Unclear
	QFrac-ture	Y	Y	●Low	●Unclear	Y	NI	Y	Y	Y	●Low	●Low	PN	Y	Y	Y	Y	NI	Y	●Unclear	●Low	Y	Y	PN	Y	Y	N	Y	Y	N	N	●High	●High	●Unclear

Study	Tool	Domain 1. Participants				Domain 2. Predictors							Domain 3. Outcomes									Domain 4. Analysis											Overall Appraisal	
		1.1 Data sources	1.2 Eligibility	Risk of bias	Applicability	2.1 Definitions	2.2 Blinding	2.3a Available	2.3b Collected/used	2.3c Missing data	Risk of bias	Applicability	3.1a Determination (CFF)	3.1b Determination (hip)	3.2 Definition	3.3 Predictors excluded	3.4 Similar definition	3.5 Knowledge of predictor	3.6 Interval	Risk of Bias	Applicability	4.1a No. outcomes (hip)	4.1b No. outcomes (CFF)	4.2 Predictor handling	4.3 Missing participants	4.4 Deaths/competing risk	4.5 Complexities in data	4.6a Performance data (hip)	4.6b Calibration plots (hip)	4.6c Performance data (CFF)	4.6d Calibration plots (CFF)	Risk of Bias	Risk of Bias	Applicability
Desbiens 2020	FRAX	Y	Y	●Low	●Low	Y	NI	N	PY	N	●Unclear	●Low	NI	NA	Y	Y	Y	NI	Y	●Unclear	●Low	NA	Y	PN	PY	N	N	NA	NA	Y	Y	●High	●High	●Low
	Garvan	Y	Y	●Low	●Low	Y	NI	N	PY	N	●Unclear	●Low	NI	NA	Y	Y	Y	NI	Y	●Unclear	●Low	NA	Y	PN	PY	N	N	NA	NA	Y	Y	●High	●High	●Low
	QFracture	Y	Y	●Low	●Low	Y	NI	N	PY	N	●Unclear	●Low	NI	NA	Y	Y	Y	NI	Y	●Unclear	●Low	NA	Y	PN	PY	N	N	NA	NA	Y	Y	●High	●High	●Low
Ettinger 2012	FRAX	Y	Y	●Low	●High	Y	NI	Y	Y	N	●High	●Low	Y	Y	Y	Y	Y	NI	PY	●Low	●Low	Y	Y	Y	N	N	N	Y	Y	Y	PY	●High	●High	●High
Ettinger 2013	FRAX	Y	Y	●Low	●High	Y	NI	Y	N	PN	●High	●Low	Y	Y	Y	Y	Y	NI	PY	●High	●High	Y	Y	Y	N	Y	PN	Y	Y	Y	PY	●High	●High	●High
Fraser 2011	FRAX	Y	Y	●Low	● bUnclear	N	NI	Y	Y	NI	●Unclear	●Low	PY	Y	Y	Y	Y	Y	Y	●Unclear	●Low	Y	Y	PY	Y	Y	Y	Y	Y	Y	Y	●Unclear	●Unclear	●Unclear
Goldshtein 2018	FRAX	Y	Y	●Low	●High	Y	NI	Y	PY	Y	●Low	●Low	PN	Y	Y	Y	Y	NI	Y	●Unclear	●Low	Y	Y	N	Y	Y	N	Y	PY	Y	PY	●High	●High	●High
Gourlay 2017	FRAX	Y	Y	●Low	●Low	NI	NI	Y	NI	N	●High	●Low	Y	Y	Y	Y	Y	NI	N	●High	●Low	Y	Y	PY	NI	Y	N	PY	Y	N	N	●High	●High	●Low
	Garvan	Y	Y	●Low	●Low	NI	NI	Y	NI	N	● High	●Low	Y	Y	Y	Y	Y	NI	N	●High	●Low	Y	Y	PY	NI	Y	N	PY	Y	N	N	●High	●High	●Low
	QFracture	Y	Y	●Low	●Low	NI	NI	Y	NI	N	● High	●Low	Y	Y	Y	Y	Y	NI	N	●High	●Low	Y	Y	PY	NI	Y	N	PY	Y	N	N	●High	●High	●Low
Holloway 2018	FRAX	Y	Y	●Low	●Low	Y	NI	Y	Y	Y	● Low	●Low	PN	Y	Y	Y	Y	NI	Y	●Unclear	●Low	N	N	Y	Y	Y	N	Y	N	Y	N	●High	●High	●Low
Iki 2015	FRAX	Y	Y	●Low	●High	Y	NI	Y	Y	Y	● Low	●Low	N	NA	Y	Y	Y	NI	N	●High	●Low	NA	N	N	PY	NI	N	NA	NA	Y	PY	●High	●High	●Unclear
Langsetmo 2011	Garvan	Y	Y	●Low	●Low	Y	NI	Y	Y	NI	●Unclear	●Low	PN	Y	Y	Y	N	NI	PY	●High	●Low	N	Y	PN	NI	Y	N	Y	Y	Y	Y	●High	●High	●Low
Leslie 2016	FRAX	Y	Y	●Low	●High	N	NI	Y	Y	Y	●Unclear	●Low	PN	NA	Y	Y	Y	NI	Y	●Unclear	●Low	NA	Y	PN	Y	Y	Y	NA	NA	Y	PY	●Unclear	●Unclear	●High
	CAROC	Y	Y	●Low	●High	N	NI	Y	Y	Y	●Unclear	●Low	PN	NA	Y	Y	Y	NI	Y	●Unclear	●Low	NA	Y	Y	Y	Y	Y	NA	NA	Y	PY	●Unclear	●Unclear	●High
Leslie 2017b	FRAX	Y	Y	●Low	●High	N	NI	Y	Y	PY	●Unclear	●Low	PN	Y	Y	Y	Y	NI	PY	●Unclear	●Low	Y	Y	N	Y	Y	Y	PY	Y	PY	Y	●Unclear	●Unclear	●High
Li 2015	FRAX	Y	Y	●Low	●Unclear	Y	NI	Y	Y	N	●High	●Low	N	Y	Y	Y	Y	NI	N	●High	●Low	NA	Y	NI	PY	N	N	NA	NA	Y	N	●High	●High	●Unclear

Study	Tool	Domain 1. Participants				Domain 2. Predictors							Domain 3. Outcomes									Domain 4. Analysis											Overall Appraisal	
		1.1 Data sources	1.2 Eligibility	Risk of bias	Applicability	2.1 Definitions	2.2 Blinding	2.3a Available	2.3b Collected/used	2.3c Missing data	Risk of bias	Applicability	3.1a Determination (CFF)	3.1b Determination (hip)	3.2 Definition	3.3 Predictors excluded	3.4 Similar definition	3.5 Knowledge of predictor	3.6 Interval	Risk of Bias	Applicability	4.1a No. outcomes (hip)	4.1b No. outcomes (CFF)	4.2 Predictor handling	4.3 Missing participants	4.4 Deaths/competing risk	4.5 Complexities in data	4.6a Performance data (hip)	4.6b Calibration plots (hip)	4.6c Performance data (CFF)	4.6d Calibration plots (CFF)	Risk of Bias	Risk of Bias	Applicability
Lo 2011	FRC	Y	PY	●Low	●High	Y	NI	Y	N	NI	●Unclear	●Low	NA	Y	Y	Y	Y	NI	PY	●Low	●Low	Y	NA	Y	Y	Y	N	Y	PY	NA	NA	●High	●High	●High
Marques 2017	FRAX	Y	Y	●Low	●High	Y	Y	Y	Y	Y	●Low	●Low	N	Y	Y	Y	PY	Y	PN	●High	●Low	N	Y	Y	N	Y	N	Y	N	Y	N	●High	●High	●Unclear
Melton 2012	FRAX	Y	Y	●Low	●Low	Y	Y	Y	Y	NI	●Unclear	●Low	Y	Y	Y	Y	Y	NI	PY	●Unclear	●Low	N	N	Y	Y	N	Y	N	N	Y	N	●High	●High	●Low
Pluskiewicz 2015	FRAX	Y	Y	●Low	●Low	Y	NI	Y	Y	NI	●Unclear	●Low	N	Y	Y	Y	N	NI	N	●High	●Low	N	N	PY	N	N	N	Y	N	Y	N	●High	●High	●Low
	Garvan	Y	Y	●Low	●Low	Y	NI	Y	Y	NI	●Unclear	●Low	N	Y	Y	Y	N	NI	N	●High	●Low	N	N	PY	N	N	N	Y	N	Y	N	●High	●High	●Low
Premaor 2013	FRAX	Y	Y	●Low	●Unclear	Y	NI	Y	Y	N	●High	●Low	Y	Y	Y	Y	Y	NI	PY	●Low	●Low	Y	Y	N	Y	Y	N	Y	PY	Y	PY	●High	●High	●Unclear
Pressman 2011	FRAX	Y	PY	●Low	●High	Y	NI	Y	N	NI	●Unclear	●Low	NA	Y	Y	Y	Y	NI	PY	●Low	●Low	Y	NA	Y	Y	N	N	Y	N	NA	NA	●High	●High	●High
Reyes Dominguez 2019	Garvan	Y	NI	●Unclear	●High	NI	NI	Y	PY	NI	●Unclear	●Low	N	Y	NI	Y	Y	NI	Y	●High	●Low	N	N	NI	N	Y	N	Y	N	Y	N	●High	●High	●High
Sornay-Rendu 2010	FRAX	Y	PY	●Low	●Low	Y	NI	Y	Y	NI	●Unclear	●Low	Y	Y	Y	Y	Y	NI	Y	●Low	●Low	N	N	NI	Y	Y	Y	Y	N	Y	N	●High	●High	●Low
Tamaki 2011	FRAX	Y	Y	●Low	●Low	Y	NI	Y	Y	NI	●Unclear	●Low	PN	Y	Y	Y	N	NI	Y	●High	●Low	N	N	N	N	N	N	Y	PY	Y	PY	●High	●High	●Unclear
Tamaki 2019	FRAX	Y	Y	●Low	●Low	Y	NI	Y	Y	NI	●Unclear	●Low	N	NA	Y	Y	Y	NI	Y	●High	●Low	NA	N	N	N	N	N	NA	NA	Y	N	●High	●High	●Unclear
Tanaka 2010	FRAX	Y	Y	●Low	●Low	PN	NI	Y	N	N	●High	●Low	NI	NA	Y	Y	NI	NI	Y	●Unclear	●Low	NA	N	PY	PY	N	N	NA	NA	Y	N	●High	●High	●Low
	FRISC	Y	Y	●Low	●Low	PY	NI	Y	N	N	●High	●Low	NI	NA	Y	Y	NI	NI	Y	●Unclear	●Low	NA	N	PY	PY	N	N	NA	NA	Y	N	●High	●High	●Low
Tebe Cordomi 2013	FRAX	Y	Y	●Low	●High	Y	NI	Y	PY	NI	●Unclear	●Low	N	NA	Y	Y	N	NI	Y	●High	●Low	NA	Y	NI	N	N	N	NA	NA	Y	PY	●High	●High	●High
Tremoll-ieres 2010	FRAX	Y	Y	●Low	●High	N	NI	Y	Y	NI	●Unclear	●Low	Y	NA	Y	Y	Y	NI	N	●High	●Low	NA	N	PY	N	N	N	NA	NA	Y	N	●High	●High	●High
Yin 2016	FRAX	Y	Y	●Low	●High	Y	NI	Y	N	N	●High	●Low	PN	Y	Y	Y	Y	NI	Y	●Unclear	●Low	Y	Y	PN	NI	N	N	Y	N	Y	PY	●High	●High	●High

Risk of bias assessed using the PROBAST tool: Wolff RF, Moons KGM, Riley RD, Whiting PF, Westwood M, Collins GS, et al. PROBAST: A tool to assessed risk of bias and applicability of prediction model studies. Ann Intern Med. 2019;170:51-58.

NI = No Information; N = No; PN = Probably No; PY = Probably Yes; Y = Yes; CFF = Clinical fragility fracture

Additional Table 7.2. Risk of bias assessments for trials included for KQ3a on the benefits of pharmacologic treatments

Study	Random sequence generation	Allocation concealment	Blinding – participants and personnel	Blinding – outcome assessment	Incomplete outcome data	Selective reporting	Other sources of bias	Overall
**Hip fractures**								
Ascott-Evans 2003	Low	Unclear	Low	Low	High	High	Low	High
Boonen 2012	Low	Unclear	Low	Low	Unclear	Low	Low	Unclear
Chesnut 1995	Low	Unclear	Unclear	Unclear	Unclear	High	Low	High
Cummings 1998	Low	Low	Low	Low	Low	Unclear	Low	Unclear
Cummings 2009	Low	Unclear	Low	Low	Unclear	Low	Low	Unclear
Grey 2009	Low	Low	Low	Low	Low	High	Low	High
Grey 2014	Low	Low	Low	Low	Low	High	Low	High
Hosking 1998	Unclear	Unclear	Low	Low	Low	Unclear	Low	Unclear
Li 2005	Unclear	Unclear	Unclear	Unclear	High	Unclear	Low	High
Liberman 1995	Unclear	Unclear	Unclear	Unclear	Unclear	Unclear	Low	Unclear
McClung 2001	Unclear	Unclear	Unclear	Unclear	High	Unclear	Low	High
Mortensen 1998	Low	Unclear	Unclear	Unclear	High	High	Low	High
Orwoll 2012	Low	Low	Low	Low	Low	High	Low	High
Pitale 2015	Unclear	Unclear	Unclear	Unclear	Low	High	Low	High
Pols 1999	Unclear	Unclear	Unclear	Unclear	Low	Unclear	Low	Unclear
Reid 2018	Low	Low	Low	Low	Low	Low	Low	Low
Välimäki 2007	Unclear	Unclear	Low	Low	Low	Unclear	Low	Unclear
Yan 2009	Low	Unclear	Unclear	Unclear	Unclear	High	Low	High
Zhu 2017	Unclear	Unclear	Low	Low	Low	High	Low	High
**Clinical fragility fractures**								
Ascott-Evans 2003	Low	Unclear	Low	Low	High	High	Low	High
Bell 2002	Unclear	Unclear	Unclear	Unclear	High	High	Low	High
Bone 2008	Low	Unclear	Unclear	Unclear	Unclear	Unclear	Low	Unclear
Boonen 2012	Low	Unclear	Low	Low	Unclear	Low	Low	Unclear
Chesnut 1995	Low	Unclear	Unclear	Unclear	Unclear	High	Low	High
Cummings 1998	Low	Low	Low	Low	Low	Unclear	Low	Unclear
Cummings 2009	Low	Unclear	Low	Low	Unclear	Low	Low	Unclear
Fogelman 2000	Low	Unclear	Unclear	Unclear	High	Unclear	Low	High
Grey 2009	Low	Low	Low	Low	Low	High	Low	High
Grey 2014	Low	Low	Low	Low	Low	High	Low	High
Hooper 2005	Low	Low	Unclear	Unclear	Unclear	Unclear	Low	Unclear
Hosking 1998	Unclear	Unclear	Low	Low	Low	Unclear	Low	Unclear
Hosking 2003	Low	Unclear	Unclear	Unclear	Unclear	Unclear	Low	Unclear

Study	Random sequence generation	Allocation concealment	Blinding – participants and personnel	Blinding – outcome assessment	Incomplete outcome data	Selective reporting	Other sources of bias	Overall
Lewiecki 2007 (alendronate)	● Low	● Low	● High	● High	● Unclear	● High	● Low	● High
Lewiecki 2007 (denosumab)	● Low	● Low	● Low	● Low	● Unclear	● High	● Low	● High
Li 2005	● Unclear	● Unclear	● Unclear	● Unclear	● High	● Unclear	● Low	● High
Liberman 1995	● Unclear	● Unclear	● Unclear	● Unclear	● Unclear	● Unclear	● Low	● Unclear
McClung 2001	● Unclear	● Unclear	● Unclear	● Unclear	● High	● Unclear	● Low	● High
McClung 2009	● Low	● Low	● Low	● Low	● Low	● Unclear	● Low	● Low
Mortensen 1998	● Low	● Unclear	● Unclear	● Unclear	● High	● High	● Low	● High
Orwoll 2012	● Low	● Low	● Low	● Low	● Low	● High	● Low	● High
Pitale 2015	● Unclear	● Unclear	● Unclear	● Unclear	● Low	● High	● Low	● High
Pols 1999	● Unclear	● Unclear	● Unclear	● Unclear	● Low	● Unclear	● Low	● Unclear
Reid 2002	● Unclear	● Low	● Low	● Low	● Unclear	● Unclear	● Low	● Unclear
Reid 2018	● Low	● Low	● Low	● Low	● Low	● Low	● Low	● Low
Välimäki 2007	● Unclear	● Unclear	● Low	● Low	● Low	● Unclear	● Low	● Unclear
Yan 2009	● Low	● Unclear	● Unclear	● Unclear	● Unclear	● High	● Low	● High
Zhu 2017	● Unclear	● Unclear	● Low	● Low	● Low	● High	● Low	● High
**All-cause mortality**								
Bone 2008	● Low	● Unclear	● Unclear	● Unclear	● Unclear	● Unclear	● Low	● Unclear
Boonen 2012	● Low	● Unclear	● Low	● Low	● Unclear	● Unclear	● Low	● Unclear
Chesnut 1995	● Low	● Unclear	● Unclear	● Unclear	● Unclear	● Unclear	● Low	● Unclear
Cummings 1998	● Low	● Low	● Low	● Low	● Low	● Unclear	● Low	● Unclear
Cummings 2009	● Low	● Unclear	● Low	● Low	● Unclear	● Low	● Low	● Unclear
Grey 2014[a]	● Low	● High	● Unclear	● Unclear	● High	● High	● Low	● High
Lewiecki 2007 (alendronate)	● Low	● Low	● Unclear	● Unclear	● Unclear	● Unclear	● Low	● Unclear
Lewiecki 2007 (denosumab)	● Low	● Low	● Low	● Low	● Unclear	● Unclear	● Low	● Unclear
McClung 2009	● Low	● Low	● Low	● Low	● Low	● Unclear	● Low	● Unclear
Orwoll 2012	● Low	● Low	● Low	● Low	● Low	● Unclear	● Low	● Unclear
Pitale 2015	● Unclear	● Unclear	● Low	● Low	● Low	● Unclear	● Low	● Unclear
Reid 2018	● Low	● Low	● Low	● Low	● Low	● Low	● Low	● Low
Välimäki 2007	● Unclear	● Unclear	● Low	● Low	● Low	● Unclear	● Low	● Unclear
Yan 2009	● Low	● Unclear	● Unclear	● Unclear	● Unclear	● Unclear	● Low	● Unclear
Zhu 2017	● Unclear	● Unclear	● Low	● Low	● Low	● Low	● Low	● Unclear

Study	Random sequence generation	Allocation concealment	Blinding – participants and personnel	Blinding – outcome assessment	Incomplete outcome data	Selective reporting	Other sources of bias	Overall
**Quality of life**								
Cummings 2009	● Low	● Unclear	● Low	● Low	● Unclear	● Unclear	● Low	● Unclear

Risk of bias assessed using the Cochrane Risk of Bias Tool, v. 2011: Higgins JPT, Altman DG, Gøtzsche PC, Jüni P, Moher D, Oxman AD, et al. The Cochrane Collaboration's tool for assessing risk of bias in randomized trials. BMJ;2011;343:d5928.

[a]Mortality data are from the 5-year open-label extension (participants remained in originally randomized groups)

Additional Table 7.3. Quality appraisals of the systematic reviews included for KQ3b on the harms of pharmacologic treatments

Systematic review	Intervention[a]	Components of PICO	Protocol & deviations	Selection of designs	Comprehen-sive search	Duplicate selection	Duplication data extraction	Excluded studies	Study description	ROB appraisal[b]	Funding sources	Appropriate statistics	Impact of ROB on synthesis	Impact of ROB on results	Heterogeneity explained	Publication bias	Conflict of interest	Overall Confidence
**Any non-serious AE**																		
Davis 2016	ALE	Yes	Yes	Yes	Yes	Yes	No	Yes	Yes	Yes	Yes	No	No	No	No	No	Yes	Low
Davis 2016	RIS, ZOL	Yes	Yes	Yes	Yes	Yes	No	Yes	Yes	Yes	Yes	Yes	Yes	No	Yes	No	Yes	Low
Davis 2020	DEN	Yes	Yes	No	Yes	No	No	Yes	Yes	Yes	No	NA	NA	No	Yes	NA	Yes	Low
**Non-serious GI AE**																		
Crandall 2014	ALE, DEN	Yes	Yes	No	No	Yes	No	No	PYes	No	No	Yes	No	Yes	No	Yes	Yes	Low
Crandall 2014	RIS, ZOL	Yes	Yes	No	No	Yes	No	No	PYes	No	No	Yes	No	No	No	No	Yes	Critically Low
**Influenza-like symptoms**																		
Davis 2016	ALE	Yes	Yes	Yes	Yes	Yes	No	Yes	Yes	Yes	Yes	Yes	Yes	No	Yes	No	Yes	Low
Crandall 2014	ZOL	Yes	Yes	No	No	Yes	No	No	PYes	No	No	Yes	Yes	Yes	Yes	Yes	Yes	Low
**Influenza, Pharyngitis**																		
Davis 2016	RIS	Yes	Yes	Yes	Yes	Yes	No	Yes	Yes	Yes	Yes	NA	NA	No	Yes	No	Yes	Low
**Pyrexia, Headache**																		
Davis 2016	ZOL	Yes	Yes	Yes	Yes	Yes	No	Yes	Yes	Yes	Yes	Yes	No	No	No	No	Yes	Low
**Chills**																		
Davis 2016	ZOL	Yes	Yes	Yes	Yes	Yes	No	Yes	Yes	Yes	Yes	Yes	No	No	Yes	No	Yes	Low
**Musculoskeletal pain**																		
Diedhou 2015	DEN	Yes	No	No	No	No	No	No	Yes	No	No	NA	NA	No	Yes	NA	Yes	Critically Low
**Arthritis and arthralgia**																		
Crandall 2014	ALE, RIS, ZOL	Yes	Yes	No	No	Yes	No	No	PYes	No	No	No	No	No	No	No	Yes	Critically Low
**Myalgias, cramps, or limb pain**																		
Crandall 2014	ALE, ZOL	Yes	Yes	No	No	Yes	No	No	PYes	No	No	No	No	No	No	No	Yes	Critically Low
Crandall 2014	RIS	Yes	Yes	No	No	Yes	No	No	PYes	No	No	NA	NA	Yes	Yes	No	Yes	Critically Low
**Composite: arthralgia, myalgia, pyrexia, chills, and influenza-like symptoms**																		
Crandall 2014	ZOL	Yes	Yes	No	No	Yes	No	No	PYes	No	No	Yes	Yes	Yes	Yes	Yes	Yes	Low
**Infections**																		
Crandall 2014	DEN	Yes	Yes	No	Yes	Yes	No	No	PYes	No	No	No	No	Yes	No	Yes	Yes	Low
**Dermatologic AE**																		
**Injection site reactions**																		
Crandall 2014	DEN	Yes	Yes	No	Yes	Yes	No	No	PYes	No	No	NA	NA	Yes	Yes	Yes	Yes	Low

Systematic review	Intervention[a]	Components of PICO	Protocol & deviations	Selection of designs	Comprehen-sive search	Duplicate selection	Duplication data extraction	Excluded studies	Study description	ROB appraisal[b]	Funding sources	Appropriate statistics	Impact of ROB on synthesis	Impact of ROB on results	Heterogeneity explained	Publication bias	Conflict of interest	Overall Confidence
**Rash/eczema**																		
Crandall 2014	DEN	Yes	Yes	No	Yes	Yes	No	No	PYes	No	No	Yes	Yes	Yes	Yes	Yes	Yes	Low
**Rash**																		
Viswanathan 2018	DEN	Yes	Yes	No	Yes	Yes	No	No	Yes	Yes	No	NA	NA	Yes	Yes	Yes	Yes	Moderate
**Eczema**																		
Viswanathan 2018	DEN	Yes	Yes	No	No	Yes	No	Yes	Yes	Yes	No	NA	NA	Yes	Yes	Yes	Yes	Moderate
**Any serious AE**																		
Viswanathan 2018	ALE, RIS, ZOL, DEN	Yes	Yes	No	No	Yes	No	Yes	Yes	Yes	No	Yes	Yes	Yes	Yes	Yes	Yes	Moderate
**Serious gastrointestinal AE (excluding cancers)**																		
Crandall 2014	ALE, RIS	Yes	Yes	No	No	Yes	No	No	PYes	No	No	Yes	No	No	Yes	No	Yes	Critically Low
Crandall 2014	ZOL, DEN	Yes	Yes	No	No	Yes	No	No	PYes	No	No	NA	NA	Yes	Yes	No	Yes	Critically Low
**GI perforation, ulcers, or bleeds**																		
Crandall 2014	ALE	Yes	Yes	No	No	Yes	No	No	PYes	No	No	Yes	No	No	Yes	No	Yes	Critically Low
Crandall 2014	RIS	Yes	Yes	No	No	Yes	No	No	PYes	No	No	No	No	No	No	No	Yes	Critically Low
Crandall 2014	ZOL, DEN	Yes	Yes	No	No	Yes	No	No	PYes	No	No	NA	NA	Yes	Yes	No	Yes	Critically Low
**Serious esophageal AE**																		
Crandall 2014	ALE, RIS	Yes	Yes	No	No	Yes	No	No	PYes	No	No	No	No	No	No	No	Yes	Critically Low
Crandall 2014	ZOL, DEN	Yes	Yes	No	No	Yes	No	No	PYes	No	No	NA	NA	Yes	Yes	No	Yes	Critically Low
**Serious hepatobiliary AE**																		
Crandall 2014	ALE, RIS, ZOL, DEN	Yes	Yes	No	No	Yes	No	No	PYes	No	No	NA	NA	Yes	Yes	No	Yes	Critically Low
**GI cancer**																		
Crandall 2014	RIS	Yes	Yes	No	No	Yes	No	No	PYes	No	No	NA	NA	No	Yes	No	Yes	Critically Low
**Colon or colorectal**																		
Chen 2015	ALE	Yes	No	No	No	Yes	No	Yes	No	Yes	No	No	No	Yes	No	Yes	Yes	Critically Low
Crandall 2014	DEN	Yes	Yes	No	No	Yes	No	No	PYes	No	No	NA	NA	Yes	Yes	No	Yes	Low
**Gastric**																		
Chen 2015	ALE	Yes	No	No	No	Yes	No	Yes	No	Yes	No	Yes	Yes	Yes	Yes	Yes	Yes	Critically Low
**Esophageal**																		
Chen 2015	ALE	Yes	No	No	No	Yes	No	Yes	No	Yes	No	No	No	Yes	No	Yes	Yes	Critically Low
Crandall 2014	BIS	Yes	Yes	No	No	Yes	No	No	PYes	No	No	NA	NA	No	Yes	No	Yes	Low
**Liver**																		

Systematic review	Intervention[a]	Components of PICO	Protocol & deviations	Selection of designs	Comprehen-sive search	Duplicate selection	Duplication data extraction	Excluded studies	Study description	ROB appraisal[b]	Funding sources	Appropriate statistics	Impact of ROB on synthesis	Impact of ROB on results	Heterogeneity explained	Publication bias	Conflict of interest	Overall Confidence
Chen 2015	ALE	Yes	No	No	No	Yes	No	Yes	No	Yes	No	No	No	Yes	No	Yes	Yes	Critically Low
**Pancreas**																		
Chen 2015	ALE	Yes	No	No	No	Yes	No	Yes	No	Yes	No	No	No	Yes	Yes	Yes	Yes	Critically Low
**Oral, bile duct, small intestine**																		
Chen 2015	ALE	Yes	No	No	No	Yes	No	Yes	No	Yes	No	NA	NA	Yes	Yes	Yes	Yes	Critically Low
**Serious cardiovascular AEs**																		
Kranenburg 2016	BIS, ZOL	Yes	No	No	PYes	Yes	Yes	No	Yes	PYes	No	Yes	Yes	Yes	Yes	Yes	Yes	Low
**Acute coronary syndrome**																		
Crandall 2014	ALE, RIS, ZOL	Yes	Yes	No	No	Yes	No	No	PYes	No	No	No	No	No	No	No	Yes	Critically Low
**Cerebrovascular events**																		
Crandall 2014	ALE, RIS	Yes	Yes	No	No	Yes	No	No	PYes	No	No	NA	NA	Yes	Yes	No	Yes	Critically Low
Crandall 2014	ZOL	Yes	Yes	No	No	Yes	No	No	PYes	No	No	No	No	No	No	No	Yes	Critically Low
Crandall 2014	DEN	Yes	Yes	No	No	Yes	No	No	PYes	No	No	NA	NA	No	Yes	No	Yes	Critically Low
Davis 2020 (stroke)	DEN	Yes	Yes	No	Yes	No	No	Yes	Yes	Yes	No	NA	NA	No	Yes	NA	Yes	Low
**Pulmonary embolism**																		
Crandall 2014	ALE, ZOL	Yes	Yes	No	No	Yes	No	No	PYes	No	No	NA	NA	Yes	Yes	No	Yes	Critically Low
**Thromboembolic events**																		
Crandall 2014	ALE, RIS, ZOL	Yes	Yes	No	No	Yes	No	No	PYes	No	No	NA	NA	No	Yes	No	Yes	Critically Low
Crandall 2014	DEN	Yes	Yes	No	No	Yes	No	No	PYes	No	No	NA	NA	Yes	Yes	No	Yes	Critically Low
Davis 2020 (venous thromboembolism)	DEN	Yes	Yes	No	Yes	No	No	Yes	Yes	Yes	No	NA	NA	No	Yes	NA	Yes	Low
**Cerebrovascular death**																		
Crandall 2014	ALE, RIS, ZOL	Yes	Yes	No	No	Yes	No	No	PYes	No	No	No	No	No	No	No	Yes	Critically Low
**Composite cardiovascular outcomes**																		
Lv 2020	DEN	Yes	Yes	No	No	Yes	Yes	Yes	PYes	Yes	No	Yes	Yes	Yes	Yes	Yes	Yes	Moderate
**Serious cardiac rhythm disturbances**																		
**Atrial fibrillation**																		
Viswanathan 2018	BIS	Yes	Yes	No	No	Yes	No	Yes	Yes	Yes	No	NA	NA	Yes	Yes	NA	Yes	Moderate
Crandall 2014	ALE, RIS	Yes	Yes	No	No	Yes	No	No	PYes	No	No	NA	NA	Yes	Yes	Yes	Yes	Low
Crandall 2014	ZOL	Yes	Yes	No	No	Yes	No	No	PYes	No	No	No	No	Yes	No	Yes	Yes	Low

Systematic review	Intervention[a]	Components of PICO	Protocol & deviations	Selection of designs	Comprehen-sive search	Duplicate selection	Duplication data extraction	Excluded studies	Study description	ROB appraisal[b]	Funding sources	Appropriate statistics	Impact of ROB on synthesis	Impact of ROB on results	Heterogeneity explained	Publication bias	Conflict of interest	Overall Confidence
Crandall 2014	DEN	Yes	Yes	No	No	Yes	No	No	PYes	No	No	NA	NA	No	Yes	No	Yes	Critically Low
**Atypical femoral fractures**																		
Crandall 2014	BIS	Yes	Yes	No	No	Yes	No	No	PYes	No	No	NA	NA	Yes	Yes	Yes	Yes	Low
Fink 2019	ALE, ZOL, DEN (LT)[c]	Yes	Yes	No	No	Yes	No	Yes	Yes	Yes	Yes	NA	NA	Yes	Yes	Yes	Yes	Moderate
Davis 2020	DEN	Yes	Yes	No	Yes	No	No	Yes	Yes	Yes	No	NA	NA	No	Yes	NA	Yes	Low
**Osteonecrosis of the jaw**																		
Crandall 2014	BIS	Yes	Yes	No	No	Yes	No	No	PYes	No	No	NA	NA	Yes	Yes	Yes	Yes	Low
Fink 2019	BIS, ALE, ZOL (LT)	Yes	Yes	No	No	Yes	No	Yes	Yes	Yes	Yes	NA	NA	Yes	Yes	Yes	Yes	Moderate
Davis 2020	DEN	Yes	Yes	No	Yes	No	No	Yes	Yes	Yes	No	NA	NA	No	Yes	NA	Yes	Low
Fink 2019	DEN (LT)	Yes	Yes	No	No	Yes	No	Yes	Yes	Yes	Yes	NA	NA	Yes	Yes	Yes	Yes	Moderate
**Discontinuation due to AE**																		
Viswanathan 2018	ALE	Yes	Yes	No	No	Yes	No	Yes	Yes	Yes	No	Yes	Yes	Yes	Yes	Yes	Yes	Moderate
Viswanathan 2018	RIS	Yes	Yes	No	No	Yes	No	Yes	Yes	Yes	No	Yes	Yes	Yes	Yes	Yes	Yes	Moderate
Viswanathan 2018	ZOL	Yes	Yes	No	No	Yes	No	Yes	Yes	Yes	No	NA	NA	Yes	Yes	NA	Yes	Moderate
Viswanathan 2018	DEN	Yes	Yes	No	No	Yes	No	Yes	Yes	Yes	No	Yes	Yes	Yes	Yes	Yes	Yes	Moderate
**Rebound fractures after stopping treatment**																		
Tsourdi 2020	DEN	Yes	No	Yes	No	No	No	No	PYes	No	No	NA	NA	No	Yes	NA	Yes	Critically Low

Quality assessment performed using AMSTAR-2: Shea BJ, Reeves BC, Thuku M, Hamel C, Moran J, Moher D, et al. AMSTAR 2: A critical appraisal tool for systematic reviews that include randomized or non-randomised studies of healthcare interventions or both. BMJ. 2017;358:j4008.

ALE: alendronate; LT: long-term; DEN: denosumab; NA: not applicable (no meta-analysis); PYes: probably yes; RIS: risedronate; ZOL: zoledronic acid

[a] All interventions are compared to placebo, unless otherwise denoted

[b] Note that the following reviews assessed the risk of bias of their included studies, but these were not assessed by outcome (i.e., specifically for harms): Davis 2016, Davis 2020, Fink 2019, Viswanathan 2018.

[c] compared to no treatment

Table 7.4. Risk of bias of cohort studies included for KQ3b on harms of pharmacologic therapy

Study	Newcastle Ottawa Risk of Bias Domain								
	Was the exposed cohort representative?	Was the non-exposed cohort selected appropriately?	How was the exposure ascertained?	Was the outcome of interest present at start of study?	Does the study control for confounders (age + other)?	Does the study control for additional factors?	How is the outcome assessed?	Length of follow-up sufficient for outcome?	Adequate follow-up?
Tripto-Shkolnik 2020	Yes	Yes	Yes	No	No	No	Yes	Yes	Yes

Additional Table 7.5. Risk of bias appraisals of the studies include for KQ4 on the acceptability of screening and/or treatment

Study	Participant selection	Completeness of data			Measurement instrument					Participant understanding	Data analysis	Overall risk of bias
		Adequate participation rate	Losses to follow-up	Sample size justification	Valid, reliable, administered as intended	Outcome assessors blinded	Valid representation of benefits	Valid representation of harms	Timeframe sufficient			
de Bekker-Grob 2008	Low	Unclear	Unclear	Unclear	Low	NA	Low	Low	Low	Unclear	Low	Unclear
Fuzzell 2020	Low	Unclear	Low	Unclear	Low	NA	Low	Low	Low	Unclear	Low	Unclear
Hudson 2011	Low	High	Low	Low	Low	NA	Unclear	Low	Low	Unclear	Low	High
Hudson 2012	Low	High	Low	Unclear	Low	NA	Unclear	High	Low	Unclear	Low	High
Kalluru 2017	Low	Low	Low	Low	Low	Low	Unclear	High	Low	Unclear	Low	High
LeBlanc 2015	Low	Low	High	Low	Low	NA	Unclear	Low	Low	Low	High	High
Liu 2020 & Billington 2019	Low	Unclear	Low	Unclear	Low	NA	Unclear	Low	Low	Low	Low	Unclear
Montori 2011	Low	Low	Low	Low	Low	NA	Unclear	Low	Low	Low	Low	Unclear
Neuner 2014	Low	High	Low	Unclear	Low	NA	Low	Low	Low	Unclear	Low	High
Sheridan 2016	Low	High	Low	Low	Low	Low	Low	Low	Low	Low	Low	High
Si 2019	Low	Low	Low	Low	Low	NA	Low	Low	Low	Unclear	Low	Unclear
Smallwood 2017	Low	Low	Low	High	Low	NA	High	Unclear	Low	Low	High	High

Risk of bias assessment informed by the risk of bias subdomains included within the GRADE guidance for assessing the certainty of evidence in studies of the importance of outcomes or values and preferences. We adapted the questions to be relevant to the assessment of acceptability: Zhang Y, Alonso-Coello P, Guyatt GH, Yepes-Nuñez JJ, Akl EA, Hazlewood G, et al. GRADE guidelines: 19. Assessing the certainty of evidence in the importance of outcomes or values and preferences – risk of bias and indirectness. J Clin Epidemiol. 2019;11;94-104.
NA=not applicable
