# Supplementary material for: Screening for the primary prevention of fragility fractures among adults aged 40 years and older in primary care: systematic reviews of the effects and acceptability of screening and treatment, and the accuracy of risk prediction tools
Source: Syst Rev. 2023 Mar 21;12:51. doi: 10.1186/s13643-023-02181-w (PMC10029308; doi:10.1186/s13643-023-02181-w)
Supplement: Supplementary file 8 — Additional file 8. Responses to stakeholder comments. This file contains responses to the comments submitted by stakeholder during their review of a draft of this manuscript. Written informed consent to publish was obtained from the stakeholders who provided the stakeholder reviews. A copy of the written consent is available for review by the Editors-in-Chief of this journal. The stakeholder reviews have been anonymized. [file 13643_2023_2181_MOESM8_ESM.pdf]

Screening for the primary prevention of fragility fractures among adults aged 40 years and older in primary care: systematic reviews of the effects and acceptability of screening and treatment, and the accuracy of risk prediction tools

Authors: Michelle Gates, Jennifer Pillay (corresponding), Megan Nuspl, Aireen Wingert, Ben Vandermeer, Lisa Hartling

Affiliation: Alberta Research Centre for Health Evidence, Department of Pediatrics, University of Alberta. Edmonton Clinic Health Academy, 11405-87 Avenue NW, Edmonton, Alberta, Canada T6G 1C9. E-mail: MG: michellegates85@gmail.com; JP: jpillay@ualberta.ca; MN: megan.nuspl@ualberta.ca; AW: awingert@ualberta.ca; BV: ben.vandermeer@ualberta.ca; LH: hartling@ualberta.ca

Additional file 8. Responses to stakeholder comments

Written informed consent to publish was obtained from the stakeholders who provided the stakeholder reviews. A copy of the written consent is available for review by the Editors-in-Chief of this journal. The stakeholder reviews have been anonymized.

Stakeholder	Comments	Response
Question 1 Are the objectives and methods of these evidence reviews clear?		
1	Y	Thankyou
2	Y; I particularly appreciated how all the objectives were laid out from Q1A to Q4 and how these were referenced back in Methods and Results as well.	Thankyou
3	Y; Sound methods described. Under background, I would clearly state a heading that says 'objectives'	Thankyou. We have changed the heading in the background from Scope of systematic reviews to Objectives of systematic reviews
4	N; Your title and predominant focus is on screening. However, screening does not usually include treatment and the inclusion of this additional material on treatment effects does not add to this manuscript and may dilute some of your intention. It also does not appear to be based on a fulsome review of the pharmacotherapy literature. Unclear rationale for evaluating screening starting at age 40 – most guidelines do not start this young.	Thankyou for your comments. We have added a sentence in the background to support the rationale for looking at tool calibration (KQ2) and treatment benefits and harms (KQ3) to make this clearer. As described in the methods, our review of treatment benefits followed systematic review methods whereas for treatment harms we used an overview of reviews. Both may have different eligibility criteria than some other reviews due to the interests of the task force. For instance, we only included trials of first-line treatment compared with placebo/no treatment and where most participants did not have a prior fracture or secondary cause for osteoporosis. Although the task force did not anticipate making a recommendation to screen people 40 years of age, because in practice some people this age do get screened, or seek screening, they felt it was necessary to include this age in the review so they could report on the evidence (or lack thereof)
Question 2 Were the results clearly stated?		
1	Y	Thankyou
2	Y; Definitions were clear and useful including primary prevention population. It would be helpful to create 2 additional tables in Results summarizing screening and	Thankyou

Stakeholder	Comments	Response
	treatment data (the info that's presented in Abstract (Results section).	
3	Y; Evidence is clearly stated and supported. I would suggest the headings of each section be in a larger font to define each section more.	Thank you. The journal publishing guidelines will determine this for the final product.
4	Comments: this was a tough document to get through, and we did not find that the organization/presentation of either the data or the conclusions were clearly stated.	Thank you for your comments. We have added some subheadings to help with this.
Question 3 Are the conclusions in the reviews supported by the data that were reviewed?		
1	Y	Thank you.
2	Y; Comments: Conclusions were succinct and supported by the data reviewed. I am uncertain about the need to study "mailed offer of screening" as it is not practical for most physicians. Conclusions should include "overdiagnosis" findings as well. "Probably reduces" and "may reduce" is used interchangeably and I prefer the terminology "may" over "probably".	Thank you for your comments.<br>We agree that the mailed offer of screening may be impractical and this will be considered by the task force when making recommendations. We added a comment on overdiagnosis to the conclusions. We use "may" and "probably" when we have low and moderate, respectively, certainty of the evidence. This was described at the end of the section on Rating certainty of evidence and drawing conclusions, and in other places, such as the abstract, when we use the language, "…probably (moderate certainty).. and may (low certainty)"
3	Y; Conclusions are mostly well drawn from data and clearly stated/discussed. Strengths and limitations are discussed well.<br>Further elaboration is given in the answer to question 4 below.	Thank you for your comments.
4	Comments: well – maybe for screening, although attention needed for clarity when translating this to your intended audience. Over treatment issues seem to be vastly simplified and not easy to determine from your manuscript (including the definition of over treatment). Conclusions for treatment not clearly supported by data - some of the data re pharmacotherapy seem to reflect old data and did not seem comprehensive (eg the inclusion of alendronate 5 mg which is not used in clinical practice, and is not even on formulary in most provinces).	Thank you for your comments. We did not look at overtreatment in this review. We provided a clear definition of overdiagnosis and a supplement on how this was calculated for those more interested. For treatment we followed our eligibility criteria (e.g. RCTs of primary prevention) and agree that it is unfortunate that most trials, with comparison to no treatment/placebo, are old. As stated on lines 344-347, our clinical experts informed the inclusion of 5 mg doses of alendronate, due to the apparent uncertainty about the superiority of the 10mg/day dose and the likelihood of some variability in the doses used in practice.
Question 4 Do you have any additional comments?		
1	Blank	
	In manuscript:	Thank you for your comments.
	Comment re font size, line 138, 821	We corrected these discrepancies in font size
	Addition of "the" line 1203, and "and" 1235	We corrected these typos
2	The paper answered few very important clinical questions about harms of screening – it's crucial to start looking further into this as I have witnessed many physicians ordering BMDs in females older than 50 when it wasn't indicated. Equally important was the section about overdiagnosis.	Thank you for your comments.
3	As our group had mentioned in a previous correspondence, in the section on denosumab it indicates that this medication may not reduce the risk of hip fracture in postmenopausal women, which our content expert disagreed with based on existing evidence reported in the AACE guidelines and the ENDO Society Guidelines. We would suggest this section be reviewed and revised.	We included all evidence meeting our eligibility criteria, which focused on trials having no treatment or placebo as comparators and excluded studies looking at women at high risk due to previous fractures (e.g. the Endo Society review did not J Clin Endocrinol Metab, May 2019, 104(5):1623–1630), the latter

Stakeholder	Comments	Response
		being a major feature differentiating studies that do and do not find positive effects.
4	The document is not end-user friendly; it is data dense and the organization of the conclusions did not seem clear. In addition to a few grammatical issues, some definitions / language should be changed to reflect a higher degree of accuracy. Commenting that shared decision-making may not be the standard of care in Canada could be interpreted as being pejorative - is there evidence to support this? (if so it should be provided). Reference to "current" Canadian guidelines should be changed to 2010 guidelines (update is imminent).	Thank you for the comments. We think that the publication process and added subheadings will improve the use-friendliness of the manuscript. If the lack of accuracy reflects the use of "may" and "probably", this follows current guidance for narrative summaries of evidence following GRADE as we have cited and described. We have reviewed the sentence in the background on shared decision making and revised "would" to "may" in the latter part of what providers may be doing, but otherwise did not think major revision or citation was required. We changed the "current" to 2010.
